# Supplementary material for: Somatic Mutational Landscape in Mexican Patients: CDH1 Mutations and chr20q13.33 Amplifications Are Associated with Diffuse-Type Gastric Adenocarcinoma
Source: Int J Mol Sci. 2022 Sep 21;23(19):11116. doi: 10.3390/ijms231911116 (PMC9570354; doi:10.3390/ijms231911116)
Supplement: Supplementary file 1 [file ijms-23-11116-s001.zip › ijms-1877046-supplementary.pdf]

Supplementary Material

Somatic Mutational Landscape in Mexican Patients: CDH1 Mutations and chr20q13.33 Amplifications Are Associated with Diffuse-Type Gastric Adenocarcinoma

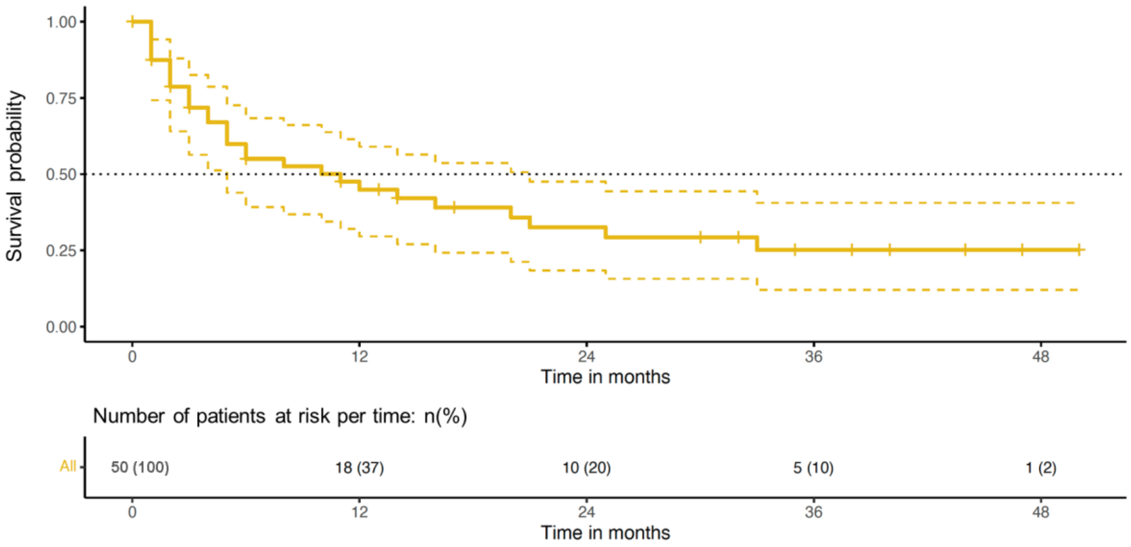

Supplementary Figure S1. Overall survival of the patients with gastric adenocarcinoma treated at the Instituto Nacional de Cancerología between January 2019 and January 2020 (n=50). Kaplan-Meier curve depicts the overall survival of all the patients included in the study. The dotted line represents the 95% confidence interval.

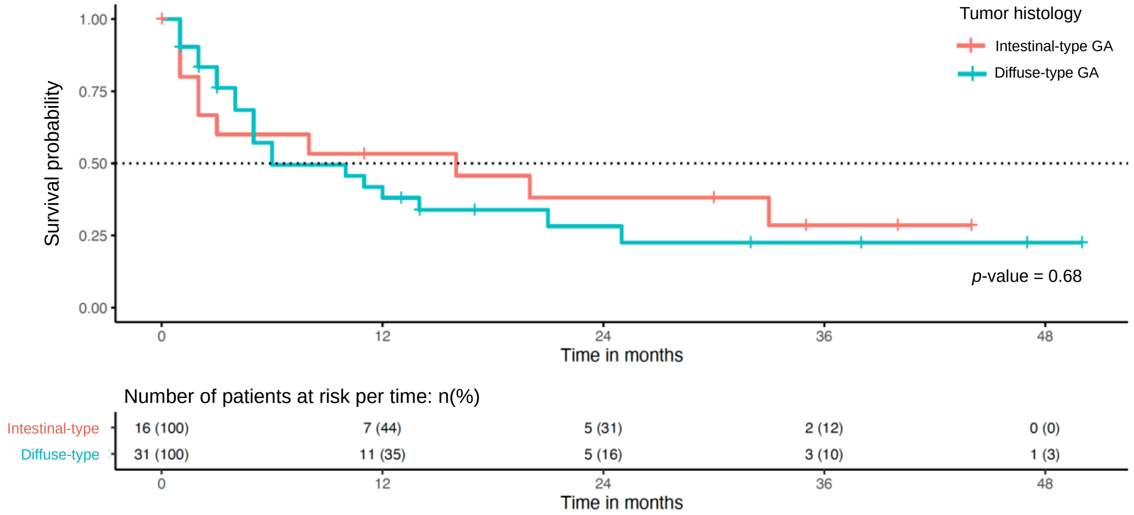

Supplementary Figure S2. Overall survival according to the tumor histology of patients with gastric adenocarcinoma treated at the Instituto Nacional de Cancerología between January 2019 and January 2020 (n=50). Kaplan-Meier curve depicts the overall survival of all the patients grouped according to their tumor histology.

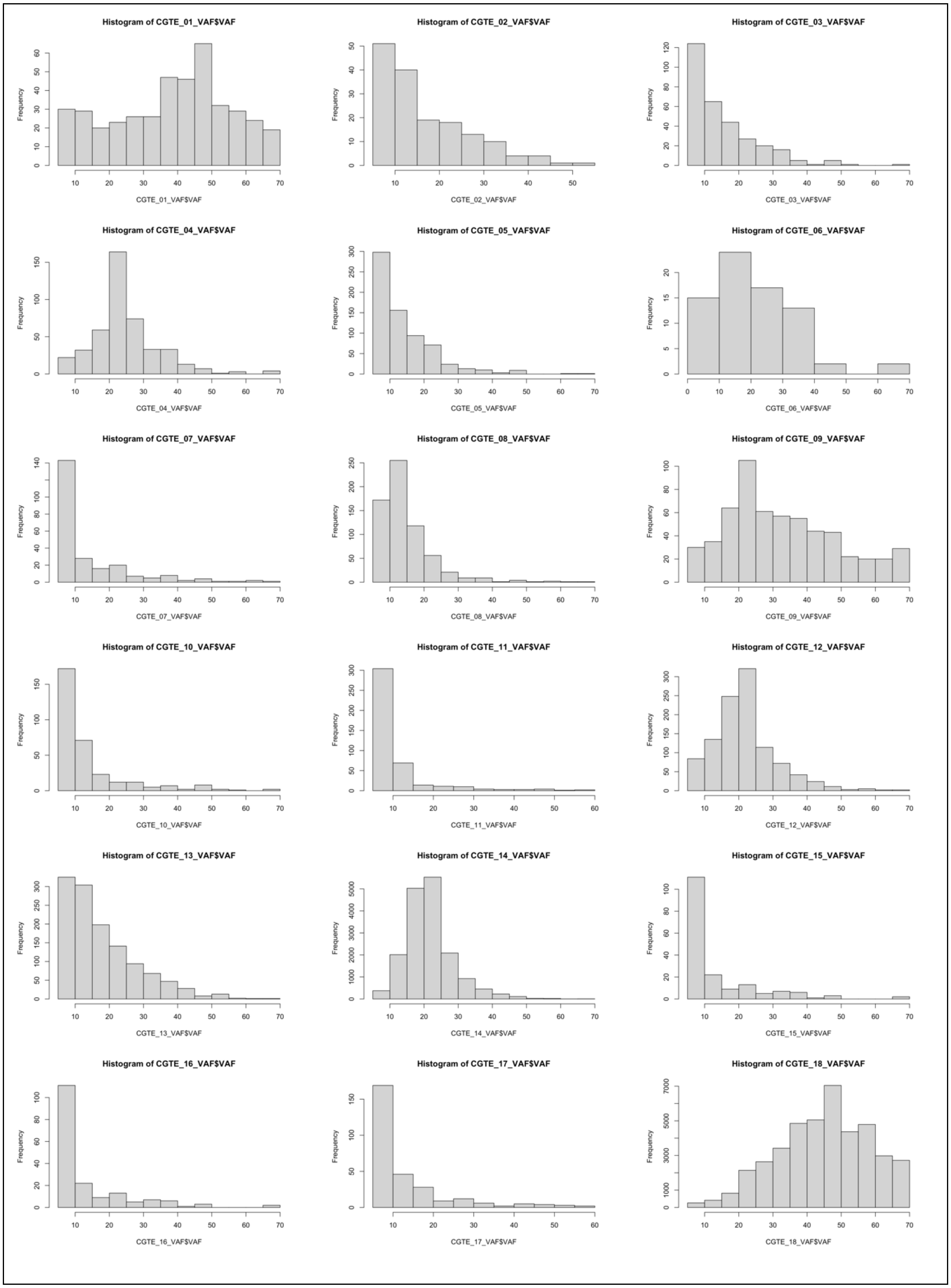
Histogram of CGTE_01_VAF$VAF
Frequency
CGTE_01_VAF$VAF
Histogram of CGTE_02_VAF$VAF
Frequency
CGTE_02_VAF$VAF
Histogram of CGTE_03_VAF$VAF
Frequency
CGTE_03_VAF$VAF
Histogram of CGTE_04_VAF$VAF
Frequency
CGTE_04_VAF$VAF
Histogram of CGTE_05_VAF$VAF
Frequency
CGTE_05_VAF$VAF
Histogram of CGTE_06_VAF$VAF
Frequency
CGTE_06_VAF$VAF
Histogram of CGTE_07_VAF$VAF
Frequency
CGTE_07_VAF$VAF
Histogram of CGTE_08_VAF$VAF
Frequency
CGTE_08_VAF$VAF
Histogram of CGTE_09_VAF$VAF
Frequency
CGTE_09_VAF$VAF
Histogram of CGTE_10_VAF$VAF
Frequency
CGTE_10_VAF$VAF
Histogram of CGTE_11_VAF$VAF
Frequency
CGTE_11_VAF$VAF
Histogram of CGTE_12_VAF$VAF
Frequency
CGTE_12_VAF$VAF
Histogram of CGTE_13_VAF$VAF
Frequency
CGTE_13_VAF$VAF
Histogram of CGTE_14_VAF$VAF
Frequency
CGTE_14_VAF$VAF
Histogram of CGTE_15_VAF$VAF
Frequency
CGTE_15_VAF$VAF
Histogram of CGTE_16_VAF$VAF
Frequency
CGTE_16_VAF$VAF
Histogram of CGTE_17_VAF$VAF
Frequency
CGTE_17_VAF$VAF
Histogram of CGTE_18_VAF$VAF
Frequency
CGTE_18_VAF$VAF

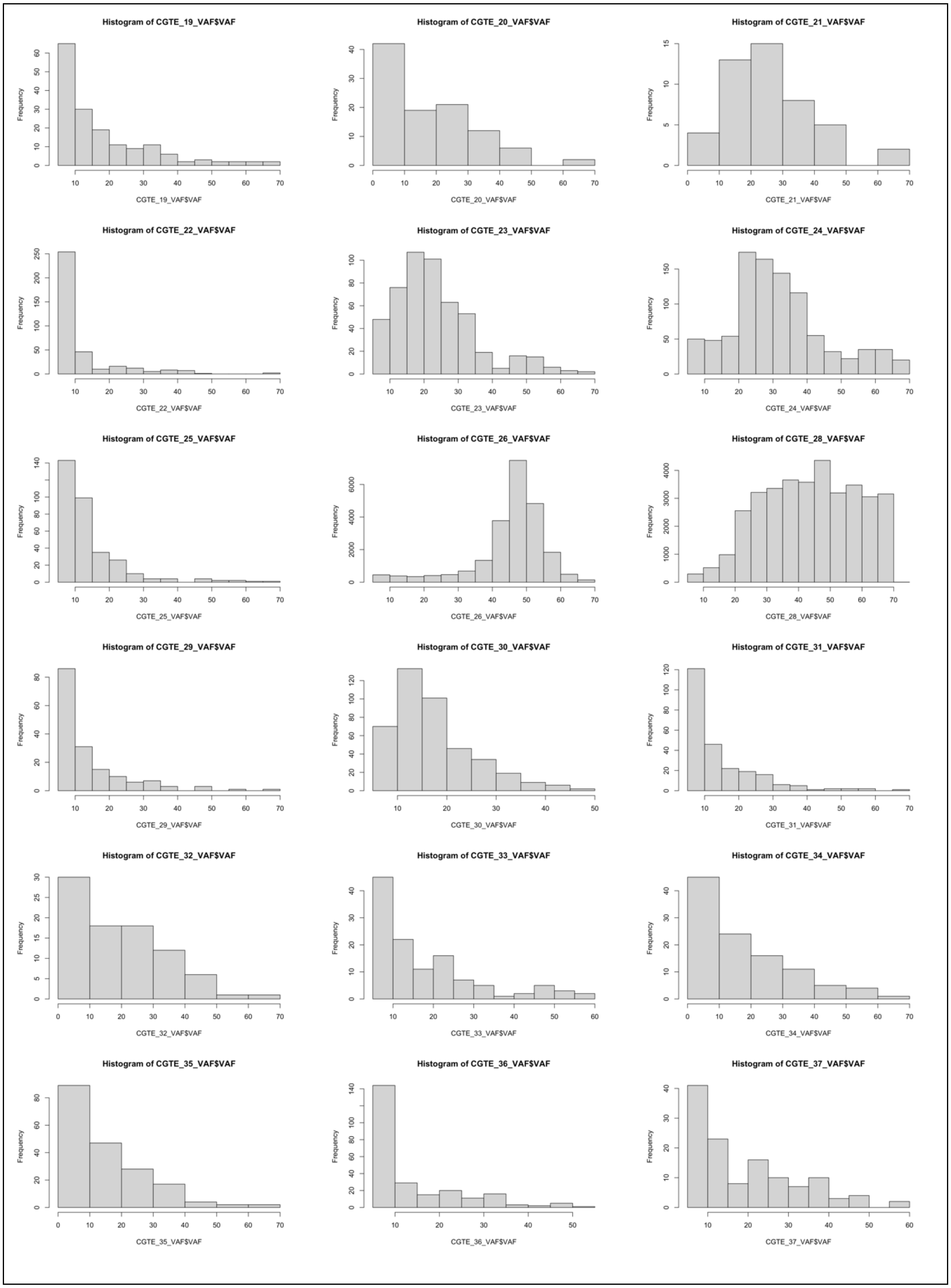
Histogram of CGTE_19_VAF$VAF
CGTE_19_VAF$VAF
Histogram of CGTE_20_VAF$VAF
CGTE_20_VAF$VAF
Histogram of CGTE_21_VAF$VAF
CGTE_21_VAF$VAF
Histogram of CGTE_22_VAF$VAF
CGTE_22_VAF$VAF
Histogram of CGTE_23_VAF$VAF
CGTE_23_VAF$VAF
Histogram of CGTE_24_VAF$VAF
CGTE_24_VAF$VAF
Histogram of CGTE_25_VAF$VAF
CGTE_25_VAF$VAF
Histogram of CGTE_26_VAF$VAF
CGTE_26_VAF$VAF
Histogram of CGTE_28_VAF$VAF
CGTE_28_VAF$VAF
Histogram of CGTE_29_VAF$VAF
CGTE_29_VAF$VAF
Histogram of CGTE_30_VAF$VAF
CGTE_30_VAF$VAF
Histogram of CGTE_31_VAF$VAF
CGTE_31_VAF$VAF
Histogram of CGTE_32_VAF$VAF
CGTE_32_VAF$VAF
Histogram of CGTE_33_VAF$VAF
CGTE_33_VAF$VAF
Histogram of CGTE_34_VAF$VAF
CGTE_34_VAF$VAF
Histogram of CGTE_35_VAF$VAF
CGTE_35_VAF$VAF
Histogram of CGTE_36_VAF$VAF
CGTE_36_VAF$VAF
Histogram of CGTE_37_VAF$VAF
CGTE_37_VAF$VAF
Frequency

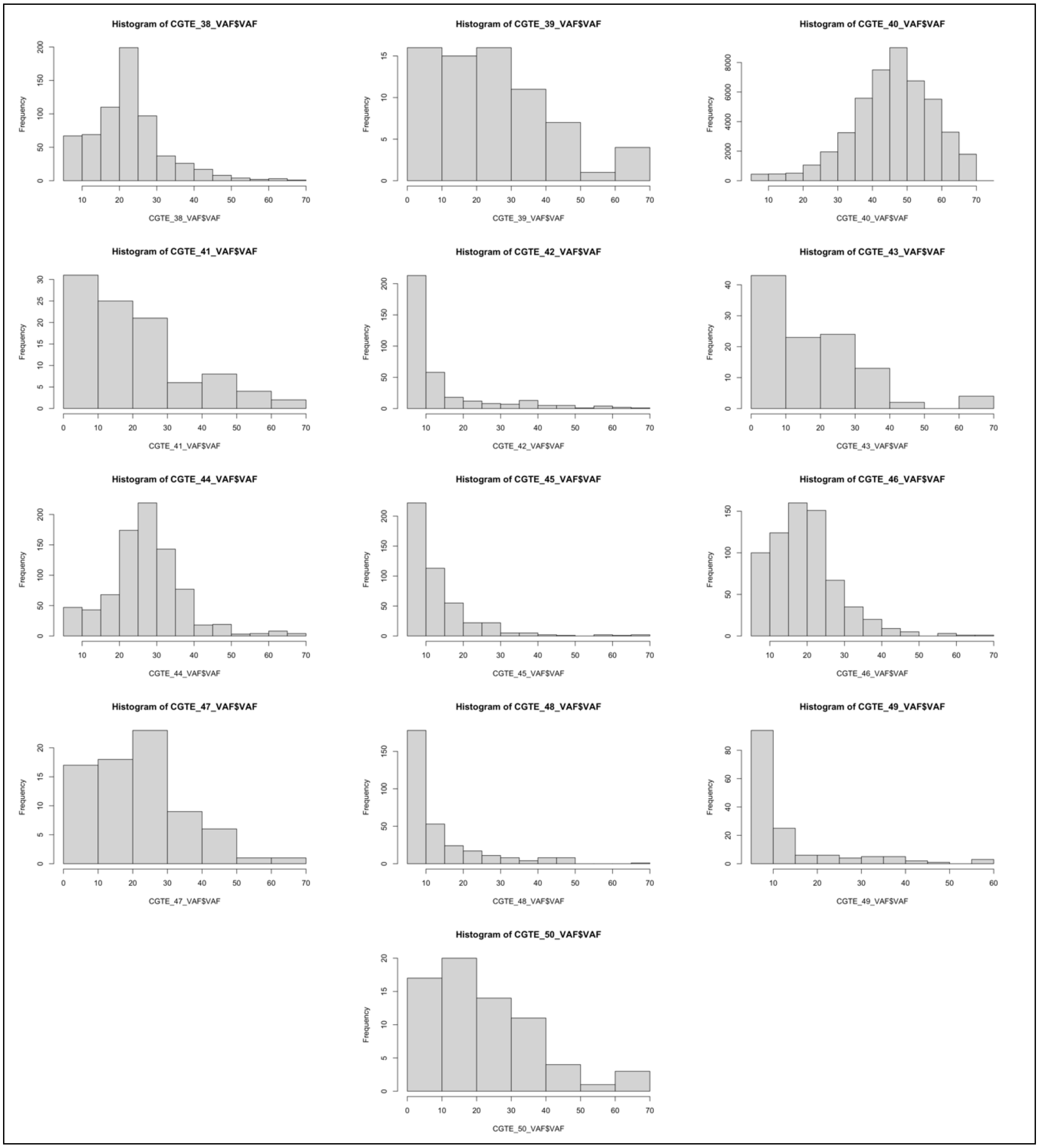

Supplementary Figure S3. Variant allele fractions (VAF) histograms for each sequenced sample

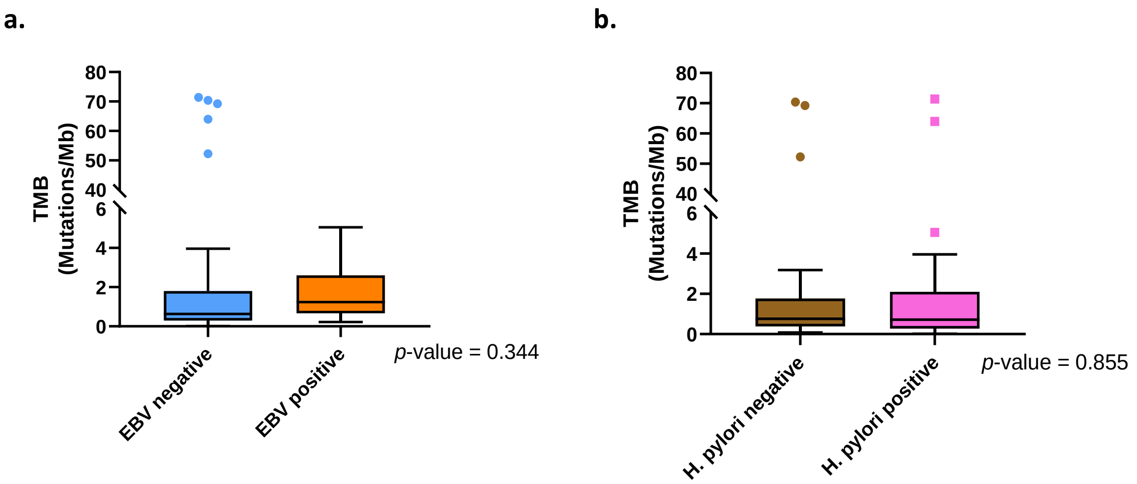

Supplementary Figure S4. Relationship between TMB value and clinical features. a) Comparison of TMB value according to the presence of Epstein-Barr Virus (EBV). b) Comparison of TMB value according to the presence of *H pylori*. p-values were calculated using Kruskal-Wallis test.

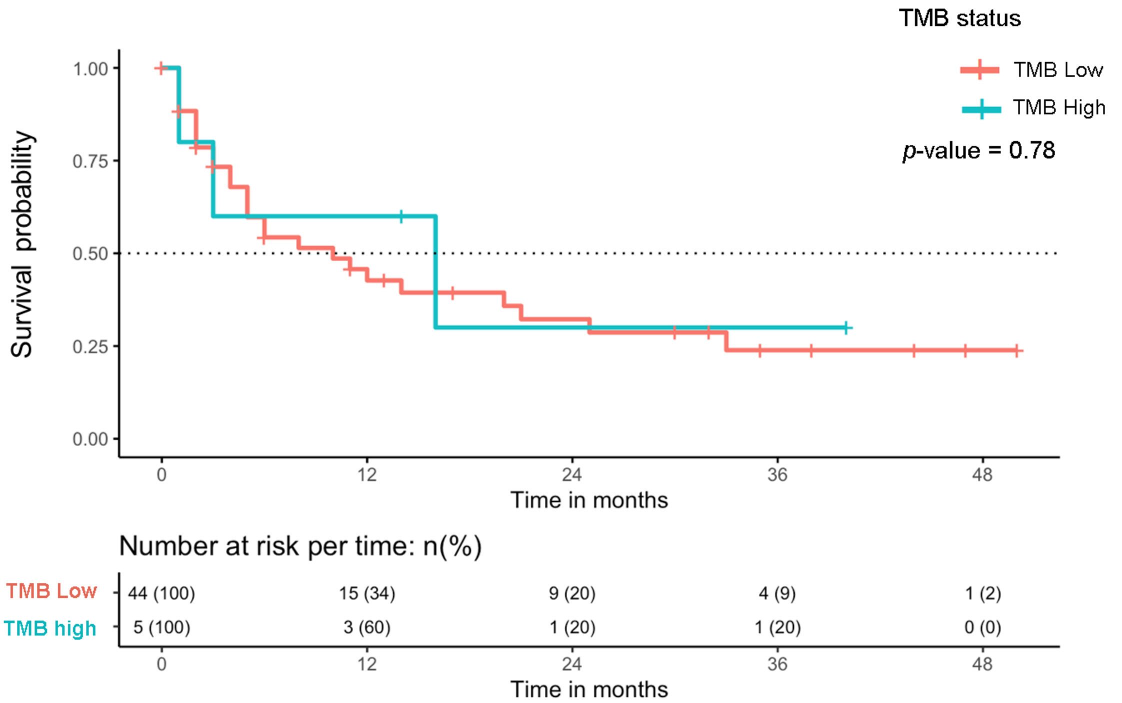

Supplementary Figure S5. Overall survival according to the Tumor mutational burden (TMB) status showed by patients with gastric adenocarcinoma treated at the Instituto Nacional de Cancerología between January 2019 and January 2020 (n=49). Lower panel indicates the number of patients at risk at different time points, grouped by TMB status

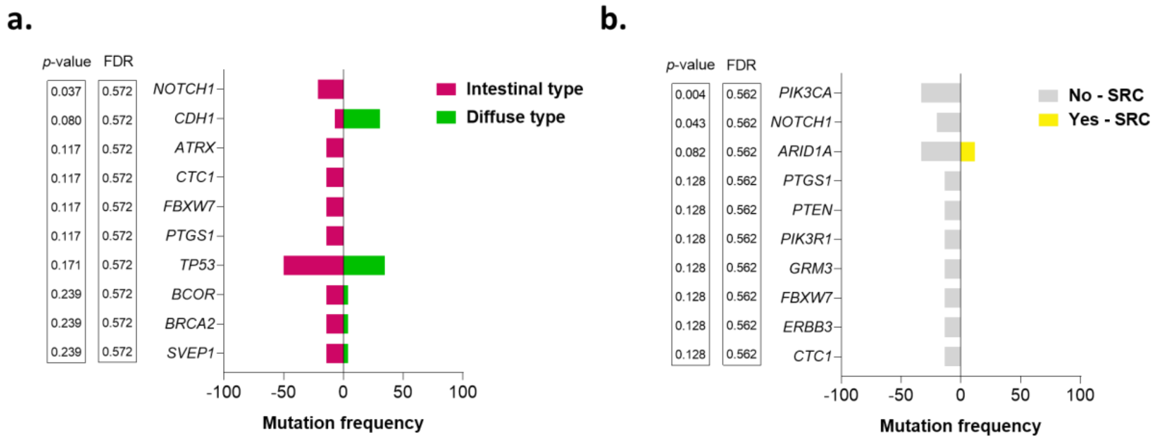

Supplementary Figure S6. Tumor histology, signet-ring cell, and frequency of driver mutations. The top-10 more differentially mutated genes are sorted from the smallest Fisher's exact test *p*-value. a) Comparison of driver mutation frequency according to tumor histology. b) Comparison of driver mutation frequency according to presence of signet-ring cell (SRC).

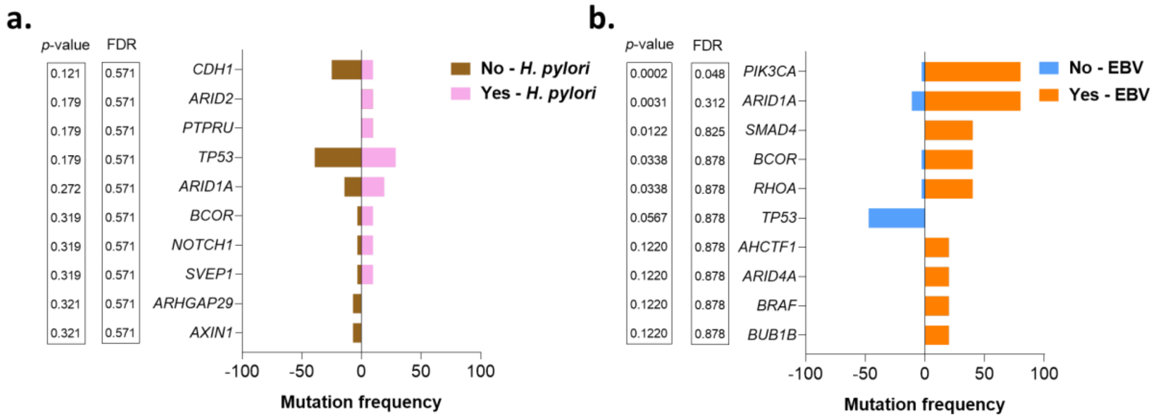

Supplementary Figure S7. *Helicobacter pylori*, EBV, and frequency of driver mutations. The top-10 more differentially mutated genes are sorted from the smallest Fisher's exact test *p*-value. a) Comparison of driver mutation frequency according to the presence of *H. pylori*. b) Comparison of driver mutation frequency according to presence of Epstein Barr Virus (EBV).

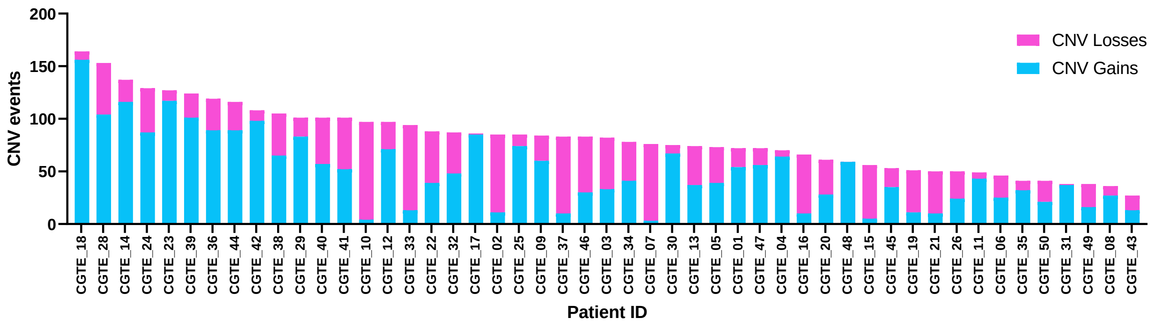

Supplementary Figure S8. Distribution of Copy number variations (CNV) events showed by patients with gastric adenocarcinoma treated at the Instituto Nacional de Cancerología between January 2019 and January 2020 (n=49).

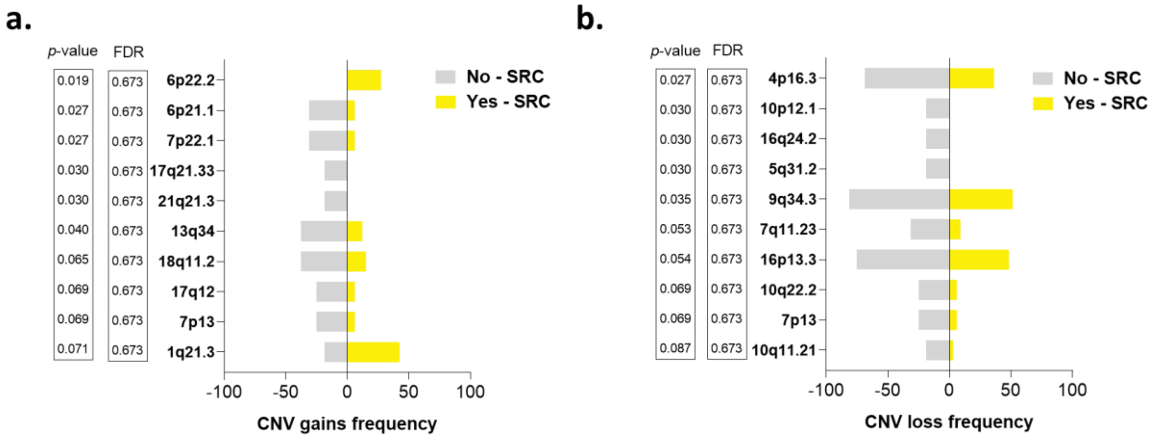

Supplementary Figure S9. Signet-ring cell and frequency of structural variants. The top-10 genes more differentially affected by copy number variations (CNV) are sorted from the smallest Fisher's exact test *p*-value. a) Comparison of CNV gains frequency according to presence of signet-ring cell (SRC). b) Comparison of CNV losses frequency according to presence of SRC.

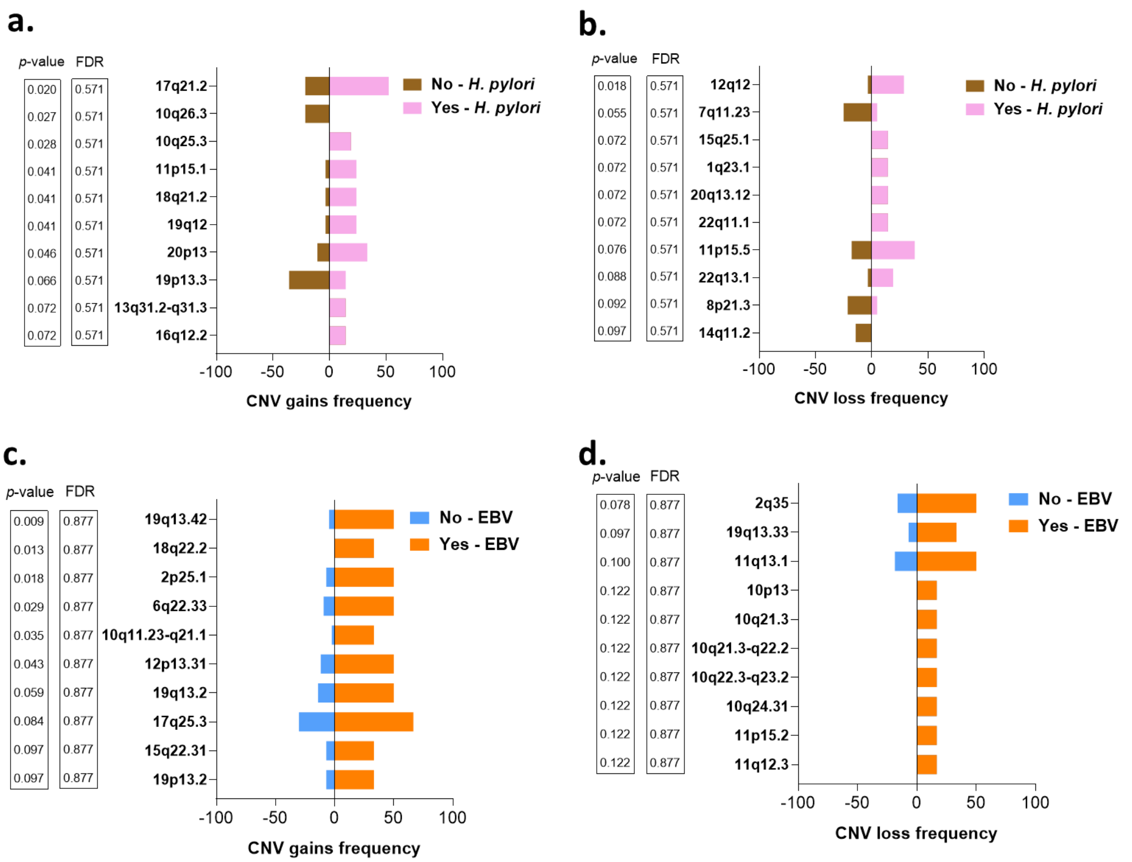

Supplementary Figure S10. *Helicobacter pylori*, EBV and frequency of structural variants. The top-10 genes more differentially affected by copy number variations (CNV) are sorted from the smallest Fisher's exact test *p*-value. a) Comparison of CNV gains frequency according to presence of *H. pylori*. b) Comparison of CNV losses frequency according to presence of *H. pylori*. c) Comparison of CNV gains frequency according to presence of Epstein-Barr Virus (EBV). d) Comparison of CNV losses frequency according to presence of EBV.

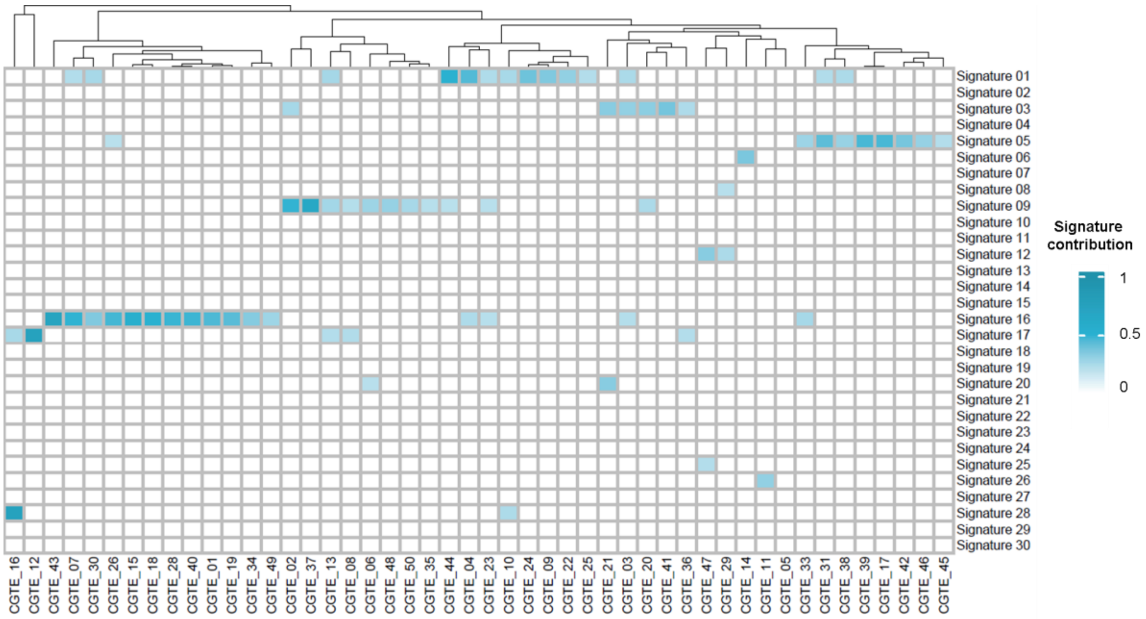

Supplementary Figure S11. Mutational signatures were present in patients with gastric adenocarcinoma treated at The Instituto Nacional de Cancerología between January 2019 and January 2020. Heatmap presenting the mutational signatures clustered by Pearson's correlation method. Signature contribution is depicted as a color gradient.

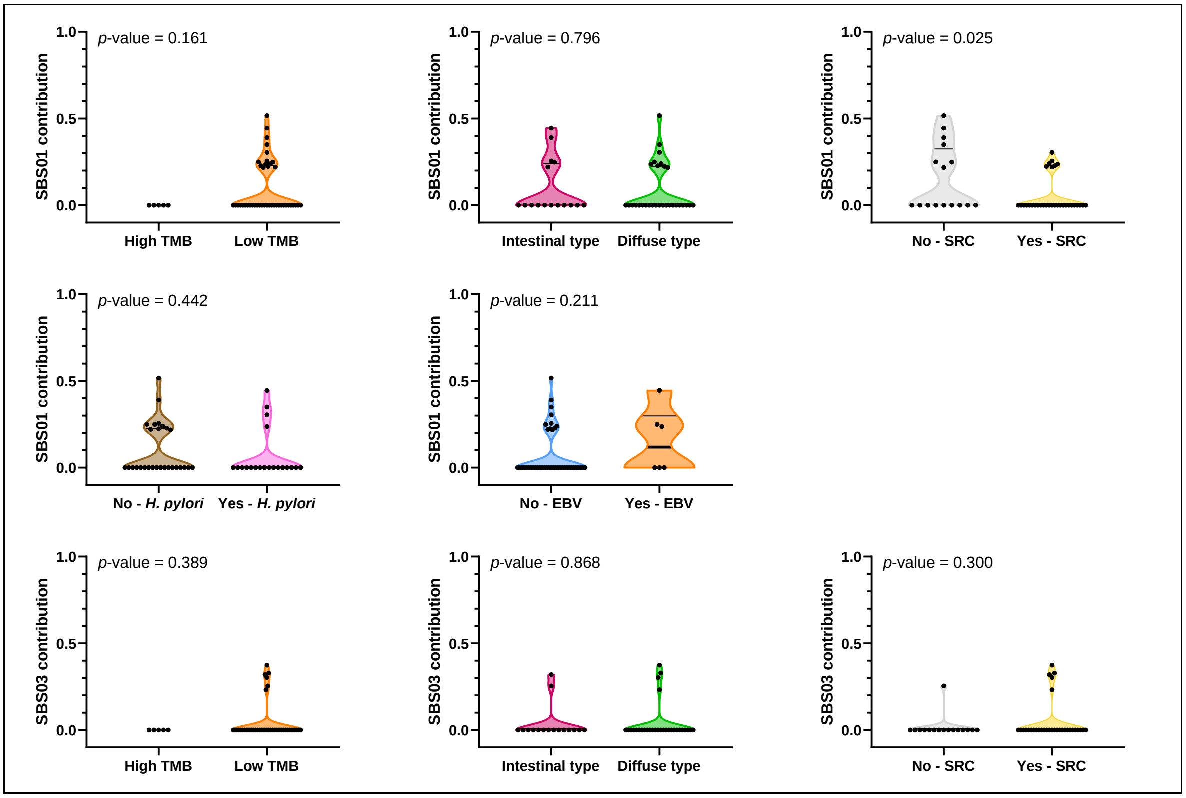

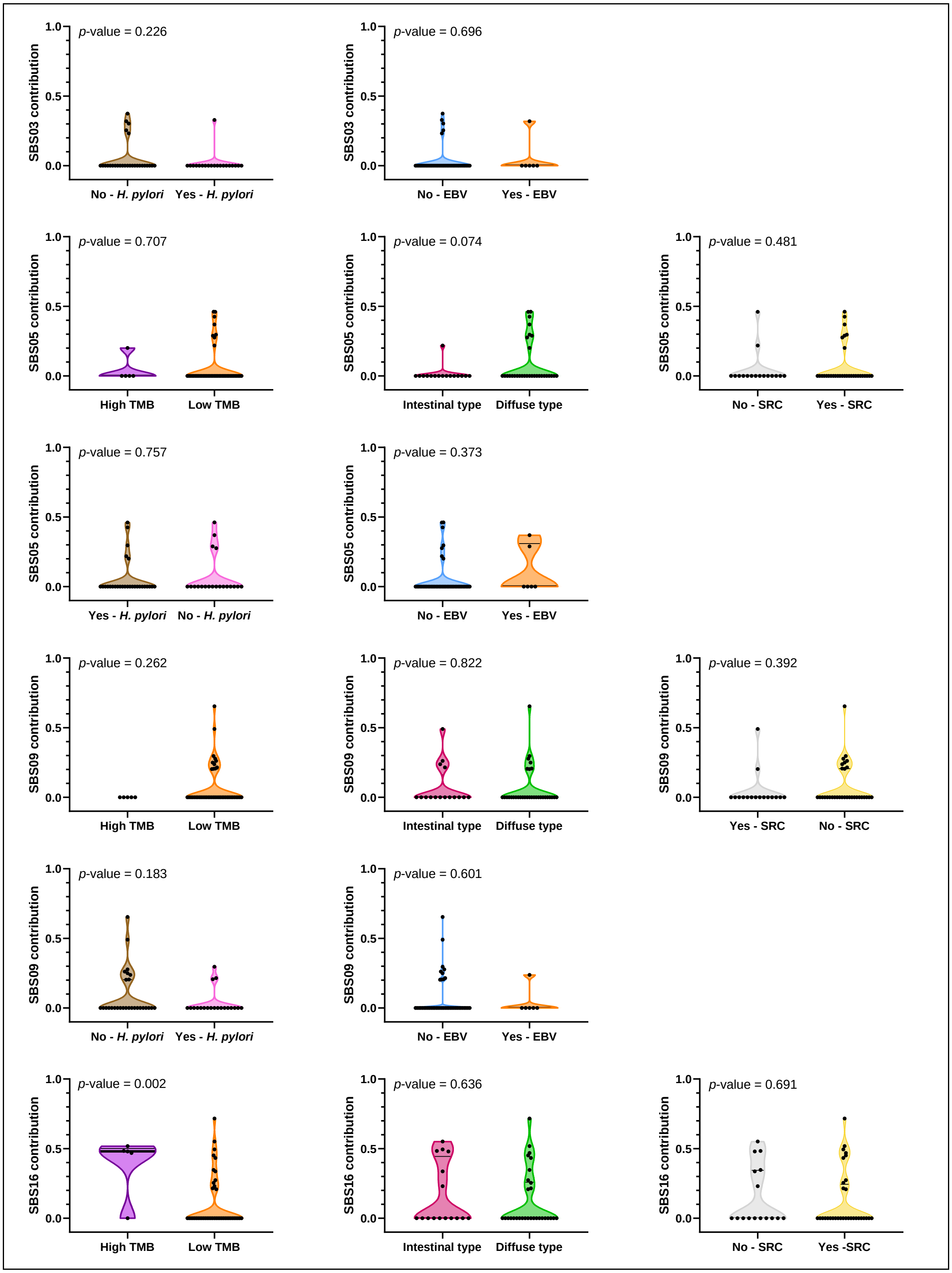
p-value = 0.226
SBS03 contribution
No - H. pylori
Yes - H. pylori
p-value = 0.696
SBS03 contribution
No - EBV
Yes - EBV
p-value = 0.707
SBS05 contribution
High TMB
Low TMB
p-value = 0.074
SBS05 contribution
Intestinal type
Diffuse type
p-value = 0.481
SBS05 contribution
No - SRC
Yes - SRC
p-value = 0.757
SBS05 contribution
Yes - H. pylori
No - H. pylori
p-value = 0.373
SBS05 contribution
No - EBV
Yes - EBV
p-value = 0.262
SBS09 contribution
High TMB
Low TMB
p-value = 0.822
SBS09 contribution
Intestinal type
Diffuse type
p-value = 0.392
SBS09 contribution
Yes - SRC
No - SRC
p-value = 0.183
SBS09 contribution
No - H. pylori
Yes - H. pylori
p-value = 0.601
SBS09 contribution
No - EBV
Yes - EBV
p-value = 0.002
SBS16 contribution
High TMB
Low TMB
p-value = 0.636
SBS16 contribution
Intestinal type
Diffuse type
p-value = 0.691
SBS16 contribution
No - SRC
Yes -SRC

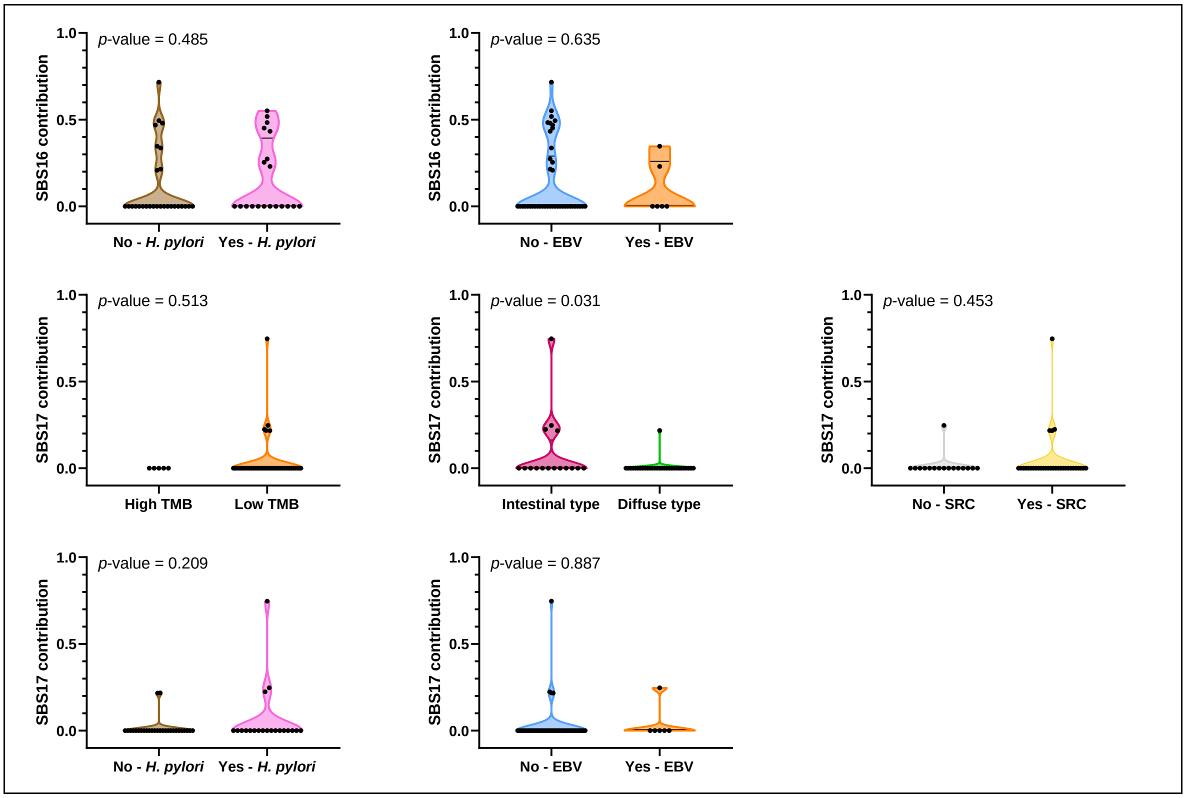

Supplementary Figure S12. Association between Mutational signature contribution and clinical-pathological characteristics of patients with gastric adenocarcinoma treated at The Instituto Nacional de Cancerología between January 2019 and January 2020. SBS: Single base substitution signature; TMB: Tumor mutational burden; EBV: Epstein-Barr Virus; SRC: Signet-ring cell

Supplementary Table S1: Driver somatic variants of patients with gastric adenocarcinoma treated at the Instituto Nacional de Cancerología between January 2019 and January 2020

PATIENT	GENE	MUTATION	CONSEQUENCE	TYPE	TRANSCRIPT	VAF	PROTEIN CHANGE
CGTE_01	CHD8	chr14:21894344-21894344 TGAATTATCAGATGAGGTATTACGTTTTCTCTTCTTACCCACTACAGGAG>-	frameshift variant	DEL	ENST00000646647	5.6	TPVVGKKRKRNTSSDNS537-553X
CGTE_01	MGA	chr15:42058202 A>G	missense variant	SNV	ENST00000219905	5.4	E2641G
CGTE_01	CDH10	chr5:24535292 C>T	missense variant	SNV	ENST00000264463	6.7	G248D
CGTE_03	AXIN1	chr16:396541 G>T	missense variant	SNV	ENST00000262320	6.9	P162Q
CGTE_03	CDH1	chr16:68855984 C>T	stop gained	SNV	ENST00000611625	11.1	R619*
CGTE_03	TP53	chr17:7578413 C>G	missense variant	SNV	ENST00000269305	22.6	V173L
CGTE_03	ELF3	chr1:201981116-201981117 ->T	frameshift variant	INS	ENST00000359651	17.1	-65-66X
CGTE_03	STK4	chr20:43610573 C>T	stop gained	SNV	ENST00000372806	15.6	R117*
CGTE_03	RHOA	chr3:49405968 A>G	missense variant	SNV	ENST00000679208	18.4	L57S
CGTE_04	HERC2	chr15:28408319 G>A	missense variant	SNV	ENST00000261609	25.1	S3556L
CGTE_04	SMARCA4	chr19:11123788 C>T	missense variant	SNV	ENST00000413806	22.7	S877L
CGTE_04	ARID1A	chr1:27087942-27087943 ->TC	frameshift variant	INS	ENST00000324856	19.3	-743-744X
CGTE_04	ARID1A	chr1:27088733-27088734 ->AC	frameshift variant	INS	ENST00000324856	37.1	I781IX
CGTE_04	PIK3CA	chr3:178936082 G>A	missense variant	SNV	ENST00000263967	30.2	E542K
CGTE_04	BCOR	chrX:39921430 C>A	stop gained	SNV	ENST00000378444	45.5	E1464*
CGTE_05	NBEA	chr13:36129123 G>A	Splice acceptor variant	SNV	ENST00000310336	7.6	--
CGTE_05	TP53	chr17:7578203 C>T	missense variant	SNV	ENST00000269305	22.7	V216M
CGTE_05	ARHGAP29	chr1:94652142 G>A	stop gained	SNV	ENST00000260526	11.6	R565*
CGTE_05	ITGA9	chr3:37522988 G>A	missense variant	SNV	ENST00000264741	10.2	R145H
CGTE_05	FAT2	chr5:150932814 C>A	missense variant	SNV	ENST00000261800	6.6	E1360D
CGTE_07	BMP5	chr6:55638936 C>T	missense variant	SNV	ENST00000370830	7.4	R313Q
CGTE_08	CIC	chr19:42796882-42796883 ->C	frameshift variant	INS	ENST00000572681	27.5	A2022AX
CGTE_08	ARID1A	chr1:27106648 G>A	missense variant	SNV	ENST00000324856	15.5	G2087R
CGTE_08	MED23	chr6:131908937 C>A	missense variant	SNV	ENST00000368058	7.2	R1336L
CGTE_08	SVEP1	chr9:113171134 C>T	missense variant	SNV	ENST00000401783	14.6	R2252H
CGTE_08	ATRX	chrX:76812989 T>C	missense variant	SNV	ENST00000373344	18.6	E2211G

CGTE_09	TP53	chr17:7577539 G>A	missense variant	SNV	ENST00000269305	68.0	R248W
CGTE_09	ARID1A	chr1:27100207 C>T	stop gained	SNV	ENST00000324856	65.5	R1335*
CGTE_09	LEF1	chr4:108991877 C>T	missense variant	SNV	ENST00000265165	65.0	R353Q
CGTE_10	WRN	chr8:30924652 T>G	missense variant	SNV	ENST00000298139	5.8	F203C
CGTE_10	WNK2	chr9:96025969 C>T	missense variant	SNV	ENST00000297954	5.4	R1178C
CGTE_11	CDH1	chr16:68844181 G>T	missense variant	SNV	ENST00000611625	7.6	D257Y
CGTE_11	STAT3	chr17:40500530 G>A	missense variant	SNV	ENST00000677421	7.7	A2V
CGTE_11	RNF43	chr17:56440748 A>G	missense variant	SNV	ENST00000584437	5.6	L157P
CGTE_12	TP53	chr17:7578402-7578403 ->A	frameshift variant	INS	ENST00000269305	30.4	C176CX
CGTE_12	FAT1	chr4:187540977 G>A	missense variant	SNV	ENST00000614102	24.4	R2257C
CGTE_13	TP53	chr17:7578268 A>T	missense variant	SNV	ENST00000269305	30.7	L194H
CGTE_13	LRP1B	chr2:141356213 C>T	missense variant	SNV	ENST00000389484	30.2	G2394E
CGTE_13	LRP1B	chr2:141356217 C>G	missense variant	SNV	ENST00000389484	10.6	D2393H
CGTE_13	CTNNA2	chr2:80101257 A>T	missense variant	SNV	ENST00000402739	5.6	K214M
CGTE_14	MGMT	chr10:131557571 C>T	missense variant	SNV	ENST00000306010	21.5	A158V
CGTE_14	ZEB1	chr10:31815886-31815887 ->GA	frameshift variant	INS	ENST00000361642	21.0	-1024-1025X
CGTE_14	PRF1	chr10:72358081 C>A	missense variant	SNV	ENST00000441259	16.9	D466Y
CGTE_14	PRF1	chr10:72358581 C>T	missense variant	SNV	ENST00000441259	25.9	R299H
CGTE_14	GATA3	chr10:8100728-8100728 C>-	frameshift variant	DEL	ENST00000379328	18.1	F234X
CGTE_14	PTEN	chr10:89711924 T>C	missense variant	SNV	ENST00000371953	26.8	L181P
CGTE_14	EXT2	chr11:44255789 T>A	missense variant	SNV	ENST00000395673	30.6	I677N
CGTE_14	CTNND1	chr11:57561545 G>A	missense variant	SNV	ENST00000399050	17.3	G87R
CGTE_14	SDHAF2	chr11:61205534 C>T	missense variant	SNV	ENST00000301761	19.0	R107C
CGTE_14	CTTN	chr11:70275255 C>T	missense variant	SNV	ENST00000376561	25.8	R339W
CGTE_14	INPPL1	chr11:71946453 C>T	missense variant	SNV	ENST00000298229	15.3	R873W
CGTE_14	INPPL1	chr11:71948748-71948748 C>-	frameshift variant	DEL	ENST00000298229	9.1	P1154X
CGTE_14	FAT3	chr11:92600238 C>T	missense variant	SNV	ENST00000409404	15.8	P3997L
CGTE_14	MRE11	chr11:94224045 G>A	missense variant	SNV	ENST00000407439	33.3	T39M
CGTE_14	TBX3	chr12:115111984 C>T	missense variant	SNV	ENST00000257566	13.9	V586I

CGTE_14	NCOR2	chr12:124970996 C>T	missense variant	SNV	ENST00000405201	24.9	G75E
CGTE_14	KMT2D	chr12:49441816-49441816 C>-	frameshift variant	DEL	ENST00000301067	12.2	A1390X
CGTE_14	KMT2D	chr12:49443667-49443667 C>-	frameshift variant	DEL	ENST00000301067	10.1	G1235X
CGTE_14	CHD4	chr12:6682398 C>T	missense variant	SNV	ENST00000645095	22.6	R1828H
CGTE_14	CEP290	chr12:88487681-88487681 T>-	frameshift variant	DEL	ENST00000675476	17.4	I1346X
CGTE_14	ING1	chr13:111372127 G>A	missense variant	SNV	ENST00000375774	33.7	E373K
CGTE_14	WASF3	chr13:27255387-27255387 C>-	frameshift variant	DEL	ENST00000335327	15.1	P305X
CGTE_14	NBEA	chr13:35730248-35730248 A>-	frameshift variant	DEL	ENST00000310336	13.7	L852X
CGTE_14	NBEA	chr13:35733663-35733663 A>-	frameshift variant	DEL	ENST00000310336	12.6	K1119X
CGTE_14	AKT1	chr14:105239596 C>G	missense variant	SNV	ENST00000554581	30.1	A317P
CGTE_14	AKT1	chr14:105246551-105246551 C>-	frameshift variant	DEL	ENST00000554581	19.1	E17X
CGTE_14	MYH11	chr16:15811127 G>A	missense variant	SNV	ENST00000396324	12.1	R1799W
CGTE_14	CREBBP	chr16:3807363-3807364 ->G	frameshift variant	INS	ENST00000262367	25.8	P1208PX
CGTE_14	CTCF	chr16:67655348-67655348 A>-	frameshift variant	DEL	ENST00000646076	14.2	E404X
CGTE_14	ELAC2	chr17:12896245 G>A	missense variant	SNV	ENST00000338034	18.2	R791W
CGTE_14	NF1	chr17:29486057 T>A	missense variant	SNV	ENST00000358273	12	N78K
CGTE_14	ACACA	chr17:35445876 C>T	missense variant	SNV	ENST00000616317	24.4	R2342H
CGTE_14	ERBB2	chr17:37879672 C>T	missense variant	SNV	ENST00000269571	17.2	R683W
CGTE_14	CCR7	chr17:38711829 G>A	missense variant	SNV	ENST00000246657	20.6	A101V
CGTE_14	DDX5	chr17:62502209 C>T	missense variant	SNV	ENST00000585111	20.4	R10H
CGTE_14	BPTF	chr17:65900821 T>C	missense variant	SNV	ENST00000321892	22.7	W1019R
CGTE_14	GPS2	chr17:7216379-7216380 ->G	frameshift variant	INS	ENST00000389167	20.7	Q290PX
CGTE_14	TP53	chr17:7578226 T>G	missense variant	SNV	ENST00000269305	15.1	D208A
CGTE_14	CTC1	chr17:8141829-8141829 G>-	frameshift variant	DEL	ENST00000651323	9.1	L106X
CGTE_14	STK11	chr19:1207098 G>T	missense variant	SNV	ENST00000326873	21.5	K62N
CGTE_14	TNPO2	chr19:12830107 A>G	missense variant	SNV	ENST00000425528	20.7	L48P
CGTE_14	ARHGAP35	chr19:47425016-47425016 T>-	frameshift variant	DEL	ENST00000672722	8.4	N1028X
CGTE_14	MYH14	chr19:50812361 C>T	missense variant	SNV	ENST00000642316	21.9	R1963C
CGTE_14	POLD1	chr19:50917038 G>A	missense variant	SNV	ENST00000595904	32.0	G790S

CGTE_14	MUC16	chr19:9049102-9049103 ->A	frameshift variant	INS	ENST00000397910	18.1	F10843FX
CGTE_14	MUC16	chr19:9057394-9057394 T>-	frameshift variant	DEL	ENST00000397910	14.2	T10018X
CGTE_14	MTOR	chr1:11184645 T>G	missense variant	SNV	ENST00000361445	20.5	D2191A
CGTE_14	ATP1A1	chr1:116933455 C>T	missense variant	SNV	ENST00000537345	21.0	A425V
CGTE_14	NOTCH2	chr1:120468424 C>T	missense variant	SNV	ENST00000256646	23.4	G1339R
CGTE_14	PRDM2	chr1:14108728-14108728 A>-	frameshift variant	DEL	ENST00000235372	6.5	K1480X
CGTE_14	NTRK1	chr1:156841488 C>T	missense variant	SNV	ENST00000524377	22.4	T264M
CGTE_14	SPEN	chr1:16261685-16261685 C>-	frameshift variant	DEL	ENST00000375759	12.5	P2984X
CGTE_14	EPHA2	chr1:16458656 C>T	missense variant	SNV	ENST00000358432	15.0	R743H
CGTE_14	EPHA2	chr1:16464593-16464593 C>-	frameshift variant	DEL	ENST00000358432	9.7	G356X
CGTE_14	ASPM	chr1:197062204 C>T	missense variant	SNV	ENST00000367409	24.2	R3091H
CGTE_14	PTPRC	chr1:198701621 G>A	missense variant	SNV	ENST00000442510	17.1	R693H
CGTE_14	CR2	chr1:207642044-207642044 C>-	frameshift variant	DEL	ENST00000367057	16.1	V206X
CGTE_14	PRKCZ	chr1:2103812 C>T	missense variant	SNV	ENST00000378567	24.2	R424W
CGTE_14	ARID1A	chr1:27100207 C>T	stop gained	SNV	ENST00000324856	20.6	R1335*
CGTE_14	PTPRF	chr1:44035428 C>T	missense variant	SNV	ENST00000359947	20.3	R183C
CGTE_14	ARHGAP29	chr1:94649835 G>A	missense variant	SNV	ENST00000260526	23.0	R707C
CGTE_14	FOXA2	chr20:22562612 G>T	missense variant	SNV	ENST00000419308	13.2	P423H
CGTE_14	ASXL1	chr20:31022441-31022442 ->G	frameshift variant	INS	ENST00000375687	9.3	-642-643X
CGTE_14	PLCG1	chr20:39788272 C>T	missense variant	SNV	ENST00000244007	15.6	R82C
CGTE_14	CSNK2A1	chr20:468109 C>T	missense variant	SNV	ENST00000217244	23.3	R312Q
CGTE_14	BRWD1	chr21:40601331 C>T	missense variant	SNV	ENST00000333229	25.8	R1011Q
CGTE_14	MYH9	chr22:36691632 C>T	missense variant	SNV	ENST00000216181	30.2	R1135Q
CGTE_14	PLXNB2	chr22:50722576 C>T	missense variant	SNV	ENST00000359337	23.8	D750N
CGTE_14	ERCC3	chr2:128018887 A>G	missense variant	SNV	ENST00000647169	14.9	S686P
CGTE_14	LRP1B	chr2:141122262 C>T	missense variant	SNV	ENST00000389484	22.2	G3700E
CGTE_14	ITGAV	chr2:187529887 A>G	missense variant	SNV	ENST00000261023	22.8	E703G
CGTE_14	PMS1	chr2:190732628 A>G	missense variant	SNV	ENST00000441310	20.4	N816D
CGTE_14	CASP8	chr2:202136260-202136260 AGA>-	Inframe deletion	DEL	ENST00000358485	16.1	SE168-169S

CGTE_14	CASP8	chr2:202149789 A>C	missense variant	SNV	ENST00000358485	16.9	K410N
CGTE_14	BIRC6	chr2:32693816 T>C	missense variant	SNV	ENST00000421745	24.4	L2031P
CGTE_14	BIRC6	chr2:32702460 C>T	missense variant	SNV	ENST00000421745	23.9	R2293C
CGTE_14	LRPPRC	chr2:44145165-44145165 T>-	frameshift variant	DEL	ENST00000260665	6.1	K1049X
CGTE_14	MSH6	chr2:48027269-48027269 CAGT>-	frameshift variant	DEL	ENST00000234420	19.1	TV716-717X
CGTE_14	MSH6	chr2:48028193 G>A	missense variant	SNV	ENST00000234420	21.2	R1024Q
CGTE_14	MSH6	chr2:48030639-48030640 ->C	frameshift variant	INS	ENST00000234420	6.1	T1085TX
CGTE_14	MSH6	chr2:48033730-48033730 A>-	frameshift variant	DEL	ENST00000234420	7.2	Q1314X
CGTE_14	GATA2	chr3:128204593 C>T	missense variant	SNV	ENST00000341105	20.0	R283H
CGTE_14	PIK3CB	chr3:138409876 G>A	missense variant	SNV	ENST00000674063	17.9	R668W
CGTE_14	MAP3K13	chr3:185155367 C>T	missense variant	SNV	ENST00000265026	19.5	T203M
CGTE_14	TGFBR2	chr3:30732996 C>T	missense variant	SNV	ENST00000359013	20.1	R562C
CGTE_14	SETD2	chr3:47143042 T>A	missense variant	SNV	ENST00000409792	20.1	T1641S
CGTE_14	TET2	chr4:106197563 C>T	missense variant	SNV	ENST00000513237	20.5	R1987C
CGTE_14	FAT4	chr4:126238572 C>T	missense variant	SNV	ENST00000394329	17.4	R336C
CGTE_14	FBXW7	chr4:153244156-153244156 C>-	frameshift variant	DEL	ENST00000603548	14.8	G667X
CGTE_14	CLOCK	chr4:56325135 C>T	missense variant	SNV	ENST00000513440	32.4	R239H
CGTE_14	PTPN13	chr4:87679477-87679478 ->A	frameshift variant	INS	ENST00000436978	24.1	-1096-1097X
CGTE_14	CTNND2	chr5:10988304-10988304 T>-	frameshift variant	DEL	ENST00000304623	23.1	T1088X
CGTE_14	TRIO	chr5:14280476 G>A	missense variant	SNV	ENST00000344204	13.5	R93H
CGTE_14	TRIO	chr5:14508350 G>A	missense variant	SNV	ENST00000344204	12.5	R3038H
CGTE_14	CDH10	chr5:24537693-24537693 T>-	frameshift variant	DEL	ENST00000264463	14.5	T108X
CGTE_14	MAP3K1	chr5:56152480 G>A	missense variant	SNV	ENST00000399503	25.8	R179H
CGTE_14	MAP3K1	chr5:56155721-56155721 AA>-	frameshift variant	DEL	ENST00000399503	20.3	RK271-272RX
CGTE_14	TNPO1	chr5:72183988 C>T	missense variant	SNV	ENST00000337273	21.0	R463C
CGTE_14	MSH3	chr5:79970915-79970915 A>-	frameshift variant	DEL	ENST00000265081	12.3	K381X
CGTE_14	ARID1B	chr6:157100024-157100024 GGAGGA>-	Inframe deletion	DEL	ENST00000636930	15.5	GG404-405-
CGTE_14	ARID1B	chr6:157405954-157405954 G>-	frameshift variant	DEL	ENST00000636930	25.4	P802X
CGTE_14	HLA-A	chr6:29911050-29911050 CA>-	frameshift variant	DEL	ENST00000376806	18.2	H117X

CGTE_14	ZNF292	chr6:87928358-87928358 T>-	frameshift variant	DEL	ENST00000369577	19.1	H149X
CGTE_14	ZNF292	chr6:87964420 G>A	missense variant	SNV	ENST00000369577	16.8	R358H
CGTE_14	POT1	chr7:124503541 G>A	missense variant	SNV	ENST00000357628	27.5	R137C
CGTE_14	EPHA1	chr7:143092462 C>T	missense variant	SNV	ENST00000275815	31.4	G678D
CGTE_14	EZH2	chr7:148514997-148514997 CTC>-	Inframe deletion	DEL	ENST00000320356	27.1	E404-
CGTE_14	KMT2C	chr7:151845715 C>T	missense variant	SNV	ENST00000262189	18.4	A4433T
CGTE_14	KMT2C	chr7:151884920 C>A	missense variant	SNV	ENST00000262189	13.2	R1558L
CGTE_14	SFRP4	chr7:37947086 C>A	missense variant	SNV	ENST00000436072	17.3	V346L
CGTE_14	ADCY1	chr7:45753376 G>A	missense variant	SNV	ENST00000297323	14.8	E1048K
CGTE_14	PMS2	chr7:6031676 C>T	missense variant	SNV	ENST00000265849	23.4	V306M
CGTE_14	GRM3	chr7:86469103 C>T	missense variant	SNV	ENST00000361669	12.9	T758M
CGTE_14	TRRAP	chr7:98609892 G>A	missense variant	SNV	ENST00000456197	35.0	E3846K
CGTE_14	UBR5	chr8:103289349-103289349 T>-	frameshift variant	DEL	ENST00000520539	8.1	K2120X
CGTE_14	IKBKB	chr8:42175260 G>A	missense variant	SNV	ENST00000520810	18.6	R404Q
CGTE_14	ARFGEF1	chr8:68140284-68140284 T>-	frameshift variant	DEL	ENST00000262215	10.4	I1169X
CGTE_14	SVEP1	chr9:113169344 C>A	stop gained	SNV	ENST00000401783	17.1	G2849*
CGTE_14	RGS3	chr9:116269602 G>A	missense variant	SNV	ENST00000374140	14.8	R374H
CGTE_14	PTGS1	chr9:125143720-125143720 C>-	frameshift variant	DEL	ENST00000362012	12.8	D189X
CGTE_14	PTPRD	chr9:8521333 C>T	missense variant	SNV	ENST00000381196	17.5	C302Y
CGTE_14	FMR1	chrX:147013981 T>A	missense variant	SNV	ENST00000370475	14.8	F223Y
CGTE_14	BCOR	chrX:39913253-39913253 G>-	frameshift variant	DEL	ENST00000378444	5.9	P1621X
CGTE_14	BCOR	chrX:39932085-39932085 G>-	frameshift variant	DEL	ENST00000378444	7.6	P838X
CGTE_14	GATA1	chrX:48650777 C>T	missense variant	SNV	ENST00000376670	21.9	R216W
CGTE_14	MED12	chrX:70345325 G>A	missense variant	SNV	ENST00000374080	21.7	R784H
CGTE_14	ZMYM3	chrX:70462897 G>A	missense variant	SNV	ENST00000373988	20.5	T1159M
CGTE_14	ATRX	chrX:76849250 G>A	missense variant	SNV	ENST00000373344	18.5	A2009V
CGTE_15	KRAS	chr12:25398284 C>T	missense variant	SNV	ENST00000256078	14.3	G12D
CGTE_15	TP53	chr17:7578384-7578384 GCAGCGCTCATGGTGGGG>-	Inframe deletion	DEL	ENST00000269305	6.1	PHHERC177-182-
CGTE_15	DCAF12L2	chrX:125299112 G>A	missense variant	SNV	ENST00000360028	9.1	R266W

CGTE_16	ARID2	chr12:46245997 T>G	missense variant	SNV	ENST00000334344	6.6	F1364C
CGTE_16	BRCA2	chr13:32910549 T>G	missense variant	SNV	ENST00000380152	8.6	L686R
CGTE_16	BUB1B	chr15:40500899 T>G	missense variant	SNV	ENST00000412359	8.3	S705A
CGTE_16	SMAD4	chr18:48591837 C>T	stop gained	SNV	ENST00000342988	13.2	Q334*
CGTE_16	PIK3CA	chr3:178936091 G>A	missense variant	SNV	ENST00000263967	13.9	E545K
CGTE_16	CTNNB1	chr3:41266103 G>A	missense variant	SNV	ENST00000645276	20.0	G34R
CGTE_16	FAT2	chr5:150908890 T>C	missense variant	SNV	ENST00000261800	10.5	E3292G
CGTE_16	BRAF	chr7:140481492 T>G	missense variant	SNV	ENST00000496384	16.7	K439T
CGTE_16	NOTCH1	chr9:139395009 A>C	missense variant	SNV	ENST00000651671	12.3	F1977V
CGTE_17	TP53	chr17:7578406 C>T	missense variant	SNV	ENST00000269305	9.4	R175H
CGTE_17	POLQ	chr3:121192276 T>A	missense variant	SNV	ENST00000621776	5.4	K2290M
CGTE_17	GRM3	chr7:86469106 G>A	missense variant	SNV	ENST00000361669	6.9	R759Q
CGTE_18	CDH1	chr16:68844174 T>A	missense variant	SNV	ENST00000611625	55.0	D254E
CGTE_18	TAOK1	chr17:27825353-27825353 TGGCCGG>-	frameshift variant	DEL	ENST00000261716	21.0	VGR339-341X
CGTE_18	MNDA	chr1:158815463-158815463 A>-	frameshift variant	DEL	ENST00000368141	33.1	A219X
CGTE_18	ASPM	chr1:197073196 G>A	missense variant	SNV	ENST00000367409	53.7	R1729W
CGTE_18	PTPRU	chr1:29647276 A>C	missense variant	SNV	ENST00000345512	58.5	Y1266S
CGTE_18	CHEK2	chr22:29107982 A>G	missense variant	SNV	ENST00000382580	66.7	L279P
CGTE_18	CBLB	chr3:105377873 G>A	missense variant	SNV	ENST00000394030	39.5	R964W
CGTE_18	KDR	chr4:55964925 G>A	missense variant	SNV	ENST00000263923	60.0	T771M
CGTE_18	SDHA	chr5:254511 C>T	missense variant	SNV	ENST00000264932	64.7	R600W
CGTE_18	BCLAF1	chr6:136599391 C>T	missense variant	SNV	ENST00000531224	23.7	G210S
CGTE_18	ARID1B	chr6:157100023-157100024 ->GGAGGAGGA	Inframe insertion	INS	ENST00000636930	25.9	-403-404GGG
CGTE_18	SMO	chr7:128829066 A>G	missense variant	SNV	ENST00000249373	69.2	D25G
CGTE_18	PREX2	chr8:69039637 C>A	missense variant	SNV	ENST00000288368	22.8	A1250D
CGTE_18	SVEP1	chr9:113173401 G>A	missense variant	SNV	ENST00000401783	59.8	P2200L
CGTE_18	CDKN2A	chr9:21974684 G>A	missense variant	SNV	ENST00000498124	44.2	P48L
CGTE_19	CDH1	chr16:68844182 A>C	missense variant	SNV	ENST00000611625	5.0	D257A
CGTE_22	ARID2	chr12:46244296-46244296 CA>-	frameshift variant	DEL	ENST00000334344	7.1	P797X

CGTE_22	DICER1	chr14:95560482 G>A	missense variant	SNV	ENST00000343455	5.5	R1703C
CGTE_22	ERCC2	chr19:45855771 G>A	missense variant	SNV	ENST00000391945	5.6	A680V
CGTE_22	ITSN1	chr21:35254591 A>T	missense variant	SNV	ENST00000381318	5.7	K1462N
CGTE_22	CD28	chr2:204599610 G>A	missense variant	SNV	ENST00000458610	5.3	R227H
CGTE_22	SPTAN1	chr9:131383537 T>C	Splice donor variant	SNV	ENST00000630866	5.8	--
CGTE_23	GLI1	chr12:57861162 C>T	missense variant	SNV	ENST00000228682	18.1	T320M
CGTE_23	TP53	chr17:7578406 C>T	missense variant	SNV	ENST00000269305	36.5	R175H
CGTE_23	ZNF292	chr6:87970293-87970293 C>-	frameshift variant	DEL	ENST00000369577	18.1	R2316X
CGTE_23	CDKN2A	chr9:21971155-21971155 GCGCCGTGGAGCAGCAGCAGCTCCGCCACTCGGGCGCT>-	frameshift variant	DEL	ENST00000498124	32.0	SARVAELLLLHGA56-68X
CGTE_24	VIM	chr10:17278354 G>C	missense variant	SNV	ENST00000544301	27.6	K445N
CGTE_24	AXIN1	chr16:396838 C>T	missense variant	SNV	ENST00000262320	31.8	R63H
CGTE_24	ELF3	chr1:201983060 C>A	missense variant	SNV	ENST00000359651	28.9	F303L
CGTE_24	APC	chr5:112175639 C>T	stop gained	SNV	ENST00000257430	62.0	R1450*
CGTE_24	PIK3R1	chr5:67575562 G>A	Splice donor variant	SNV	ENST00000521381	23.7	--
CGTE_24	ROS1	chr6:117704490 G>C	missense variant	SNV	ENST00000368508	22.0	S829C
CGTE_25	PGR	chr11:100998703 C>T	missense variant	SNV	ENST00000325455	9.4	A367T
CGTE_25	ERBB3	chr12:56491645 G>T	missense variant	SNV	ENST00000267101	9.9	S846I
CGTE_25	CDH1	chr16:68842463 T>C	missense variant	SNV	ENST00000611625	23.5	L175P
CGTE_25	RNF43	chr17:56436128 G>A	stop gained	SNV	ENST00000584437	16.1	R337*
CGTE_25	SMAD2	chr18:45372160 G>A	missense variant	SNV	ENST00000262160	8.4	R337C
CGTE_25	LRP1B	chr2:141474326 C>A	missense variant	SNV	ENST00000389484	6.9	D1940Y
CGTE_25	ACVR2A	chr2:148680616-148680616 GATA>-	frameshift variant	DEL	ENST00000241416	16.1	RI384-385X
CGTE_25	PIK3CA	chr3:178936094 C>A	missense variant	SNV	ENST00000263967	10.4	Q546K
CGTE_26	BRCA2	chr13:32953550 G>A	missense variant	SNV	ENST00000380152	43.0	A2951T
CGTE_26	OCA2	chr15:28228553 C>T	missense variant	SNV	ENST00000354638	55.9	A481T
CGTE_26	ASH1L	chr1:155447791 G>A	missense variant	SNV	ENST00000368346	54.8	R1624W
CGTE_26	ASH1L	chr1:155448630 G>C	missense variant	SNV	ENST00000368346	43.0	P1344R
CGTE_26	PLXNB2	chr22:50714320 C>T	missense variant	SNV	ENST00000359337	38.3	E1804K
CGTE_26	INPP4A	chr2:99182547 C>T	missense variant	SNV	ENST00000074304	39.3	R784W

CGTE_26	FAT1	chr4:187541679 G>A	missense variant	SNV	ENST00000614102	47.9	R2023C
CGTE_26	POLR2B	chr4:57873010 C>T	missense variant	SNV	ENST00000381227	48.9	R416W
CGTE_28	ERCC6	chr10:50680430-50680430 G>-	frameshift variant	DEL	ENST00000355832	48.1	I972X
CGTE_28	NCOR1	chr17:15961336 C>T	missense variant	SNV	ENST00000268712	69.3	R2018Q
CGTE_28	CLTC	chr17:57763101 T>C	missense variant	SNV	ENST00000621829	18.6	W1591R
CGTE_28	TP53	chr17:7578212 G>A	stop gained	SNV	ENST00000269305	53.2	R213*
CGTE_28	CTC1	chr17:8131841 G>A	missense variant	SNV	ENST00000651323	68.9	P1165L
CGTE_28	EPHA4	chr2:222365859 G>A	missense variant	SNV	ENST00000281821	58.7	T286M
CGTE_28	INPP4A	chr2:99181226 G>A	missense variant	SNV	ENST00000074304	23.8	G723R
CGTE_28	FBXW7	chr4:153332605-153332605 CTC>-	Inframe deletion	DEL	ENST00000603548	30.1	E117-
CGTE_28	PDGFRA	chr4:55146608 T>G	missense variant	SNV	ENST00000257290	55.7	L761R
CGTE_28	PTGS1	chr9:125140732 C>T	missense variant	SNV	ENST00000362012	68.3	R78W
CGTE_28	NOTCH1	chr9:139399320 C>T	missense variant	SNV	ENST00000651671	21.8	R1608H
CGTE_29	SETBP1	chr18:42531215 C>T	missense variant	SNV	ENST00000677130	5.5	P637L
CGTE_30	ARID1A	chr1:27023479 C>A	stop gained	SNV	ENST00000324856	14.7	Y195*
CGTE_30	ARID1A	chr1:27092979-27092980 ->G	frameshift variant	INS	ENST00000324856	17.1	-970-971X
CGTE_30	PIK3CA	chr3:178952085 A>G	missense variant	SNV	ENST00000263967	9.4	H1047R
CGTE_30	RHOA	chr3:49413010 G>A	missense variant	SNV	ENST00000679208	32.3	R5W
CGTE_30	PIK3R1	chr5:67589138 G>A	missense variant	SNV	ENST00000521381	25.5	G376R
CGTE_30	ARID1B	chr6:157502256-157502257 ->CCCT	frameshift variant	INS	ENST00000636930	18.8	P1220PLX
CGTE_30	TNC	chr9:117849127 C>T	missense variant	SNV	ENST00000350763	16.3	V295M
CGTE_31	CDH1	chr16:68844191 C>G	missense variant	SNV	ENST00000611625	8.2	P260R
CGTE_31	TP53	chr17:7577548 C>T	missense variant	SNV	ENST00000269305	11.2	G245S
CGTE_31	CNOT4	chr7:135048801 C>T	missense variant	SNV	ENST00000541284	6.8	E549K
CGTE_34	H3-3A	chr1:226259113 C>G	missense variant	SNV	ENST00000366813	9.7	A115G
CGTE_35	TP53	chr17:7576901-7576902 ->GAGGAGCT	frameshift variant	INS	ENST00000269305	6.1	S315SAPX
CGTE_35	CUX1	chr7:101845383 G>A	missense variant	SNV	ENST00000360264	5.3	E947K
CGTE_36	CDH1	chr16:68844180 T>A	missense variant	SNV	ENST00000611625	5.9	N256K
CGTE_36	AXIN2	chr17:63530118-63530118 C>-	frameshift variant	DEL	ENST00000307078	6.3	E773X

CGTE_36	ITGAV	chr2:187532489 C>T	stop gained	SNV	ENST00000261023	9.0	Q807*
CGTE_37	TP53	chr17:7577538 C>T	missense variant	SNV	ENST00000269305	5.9	R248Q
CGTE_37	CDH10	chr5:24491924 C>A	missense variant	SNV	ENST00000264463	6.4	R546I
CGTE_38	CDK4	chr12:58145397 T>C	missense variant	SNV	ENST00000257904	31.4	K35R
CGTE_38	NTRK3	chr15:88669502 C>A	missense variant	SNV	ENST00000626019	21.9	G466C
CGTE_38	DCC	chr18:50278561 G>T	missense variant	SNV	ENST00000442544	20.0	D77Y
CGTE_38	STK11	chr19:1223061 G>A	missense variant	SNV	ENST00000326873	27.7	R333H
CGTE_38	AHCTF1	chr1:247059224 C>T	missense variant	SNV	ENST00000366508	26.2	E578K
CGTE_38	ARID1A	chr1:27105930-27105931 ->G	frameshift variant	INS	ENST00000324856	18.7	-1847-1848X
CGTE_38	PTPRU	chr1:29642528 C>A	missense variant	SNV	ENST00000345512	20.5	F1136L
CGTE_38	RHOA	chr3:49405962 T>C	missense variant	SNV	ENST00000679208	20.5	D59G
CGTE_38	POT1	chr7:124510966 T>A	missense variant	SNV	ENST00000357628	12.1	K85M
CGTE_38	BCOR	chrX:39932003-39932003 G>-	frameshift variant	DEL	ENST00000378444	22.1	H866X
CGTE_40	ERBB3	chr12:56481811 C>T	missense variant	SNV	ENST00000267101	54.8	R247W
CGTE_40	BIVM-ERCC5	chr13:103514694 G>A	missense variant	SNV	ENST00000639435	60.2	E853K
CGTE_40	BRCA2	chr13:32953550 G>A	missense variant	SNV	ENST00000380152	54.0	A2951T
CGTE_40	CDH1	chr16:68844191 C>G	missense variant	SNV	ENST00000611625	21.2	P260R
CGTE_40	CDH1	chr16:68849586 G>A	missense variant	SNV	ENST00000611625	17.2	E518K
CGTE_40	TP53	chr17:7577538 C>T	missense variant	SNV	ENST00000269305	31.5	R248Q
CGTE_40	PER1	chr17:8046675 C>T	missense variant	SNV	ENST00000317276	27.1	R994H
CGTE_40	PHOX2B	chr4:41747993-41747993 GCCGCCGCTGCCGCTGCCGCC>-	Inframe deletion	DEL	ENST00000226382	30.1	AAAAAAAA252-259A
CGTE_40	SMO	chr7:128829066 A>G	missense variant	SNV	ENST00000249373	42.0	D25G
CGTE_40	NOTCH1	chr9:139395221 G>A	missense variant	SNV	ENST00000651671	67.1	A1906V
CGTE_41	CDH1	chr16:68847305-68847305 GGAGGCTGTATACACCATATT>-	Inframe deletion	DEL	ENST00000611625	21.1	WEAVYTIL409-416W
CGTE_42	SH2B3	chr12:111856061 G>A	missense variant	SNV	ENST00000341259	5.8	A38T
CGTE_42	ERBB2	chr17:37879658 G>A	missense variant	SNV	ENST00000269571	5.3	R678Q
CGTE_42	LRP1B	chr2:141526869 C>A	stop gained	SNV	ENST00000389484	6.5	G1891*
CGTE_43	TJP1	chr15:30025404 G>A	missense variant	SNV	ENST00000356107	6.3	R637C
CGTE_43	PIK3CD	chr1:9783197 G>A	missense variant	SNV	ENST00000536656	5.9	G838D

CGTE_43	MECOM	chr3:168818709 C>A	missense variant	SNV	ENST00000651503	14.0	R938L
CGTE_44	PTEN	chr10:89692905 G>A	missense variant	SNV	ENST00000371953	34.6	R130Q
CGTE_44	ARID4A	chr14:58827682 C>T	stop gained	SNV	ENST00000355431	27.7	R668*
CGTE_44	SMAD4	chr18:48575186 G>C	missense variant	SNV	ENST00000342988	38.4	C127S
CGTE_44	ARID1A	chr1:27106648 G>A	missense variant	SNV	ENST00000324856	38.5	G2087R
CGTE_44	FN1	chr2:216257690 C>T	missense variant	SNV	ENST00000354785	24.6	G1345S
CGTE_44	PIK3CA	chr3:178936091 G>A	missense variant	SNV	ENST00000263967	47.7	E545K
CGTE_45	TP53	chr17:7578466 G>T	missense variant	SNV	ENST00000269305	8.1	T155N
CGTE_46	FAT3	chr11:92532030-92532030 TACATGCTGA>-	frameshift variant	DEL	ENST00000409404	18.3	YMLI1951-1954X
CGTE_46	AC006064.6	chr12:6702776 A>C	missense variant	SNV	ENST00000644480	18.5	S767A
CGTE_46	ZFHX3	chr16:72821799-72821799 GGAACAATGAAGG>-	frameshift variant	DEL	ENST00000641206	7.1	PFIVP3455-3459X
CGTE_46	STAT5B	chr17:40369218-40369219 ->G	frameshift variant	INS	ENST00000293328	12.2	Q447PX
CGTE_46	GNA13	chr17:63010797 C>A	missense variant	SNV	ENST00000439174	18.9	D238Y
CGTE_46	TP53	chr17:7579527-7579528 ->C	frameshift variant	INS	ENST00000269305	26.9	-53-54X
CGTE_46	ARID1A	chr1:27092780-27092780 T>-	frameshift variant	DEL	ENST00000324856	20.1	I934X
CGTE_46	MECOM	chr3:168849253 C>A	missense variant	SNV	ENST00000651503	5.7	D193Y
CGTE_46	FAT4	chr4:126367543-126367544 ->T	frameshift variant	INS	ENST00000394329	21.2	N2432NX
CGTE_46	PABPC1	chr8:101721758-101721758 CAGCT>-	frameshift variant	DEL	ENST00000523555	18.2	RAV390-392RX
CGTE_46	CSMD3	chr8:113812479 G>C	missense variant	SNV	ENST00000297405	5.8	D628E
CGTE_46	CDKN2A	chr9:21971110 T>C	missense variant	SNV	ENST00000498124	25.8	H83R
CGTE_48	TP53	chr17:7578289-7578290 ->T	frameshift variant	INS	ENST00000269305	12.1	G187EX
CGTE_48	RAD23B	chr9:110087284 G>A	missense variant	SNV	ENST00000358015	17.0	R372K
CGTE_49	BAP1	chr3:52441470 C>A	stop gained	SNV	ENST00000460680	9.9	G128*
CGTE_50	SETD2	chr3:47147585 C>A	stop gained	SNV	ENST00000409792	7.5	G1581*

Supplementary Table S2: Copy Number Variation (CNV) of patients with gastric adenocarcinoma treated at The Instituto Nacional de Cancerlogía between January 2019 and January 2020

#	Sample	Chr	Start	End	Cytoband	Gene Names	Copy Number	CNV Type
1	CGTE_01	1	861266	3650151	1p36.33-p36.32	PEX10,MRPL20,CFAP74,AGRN,CPTP,TMEM88B,MMP23B,CDK11B,DVL1,AURKAIP1,ARHGEF16,MIR6726,ISG15,SAMD11,HES5,UBE2J2,TP73,TAS1R3,MIB2,MIR6727,PLEKHN1,ATAD3C,ANKRD65,C1orf159,TMEM240,PLCH2,LOC102724312,RNF223,ATAD3B,RER1,FAM213B,ATAD3A,LOC148413,TNFRSF14,CDK11A,SSU72,LOC100996583,SCNN1D,C1orf233,SLC35E2B,ACAP3,MIR551A,FAAP20,PUSL1,SKI,GABRD,CCNL2,HES4,TTLL10,MEGF6,B3GALT6,PANK4,MIR6808,KLHL17,MIR200B,MIR4251,TTC34,LINC00982,TMEM52,NOC2L,ACTRT2,FAM132A,TNFRSF18,LINC01342,TPRG1L,PRDM16,MIR429,PRKCZ,MMEL1,NADK,MMP23A,LOC100129534,VWA1,GNB1,CALML6,TNFRSF4,SLC35E2,WRAP73,CPSF3L,MORN1,LOC115110,MIR200A,MXRA8,SDF4,PERM1	1	loss
2	CGTE_01	1	74574927	74621554	1p31.1	LRRIQ3	13	gain
3	CGTE_01	1	150681375	150686646	1q21.3	HORMAD1	8	gain
4	CGTE_01	1	152382018	153234472	1q21.3	LCE4A,SPRR2F,LOR,LCE1D,LCE2D,KPRP,LCE2B,IVL,LCE3D,SPRR4,CRCT1,SPRR3,LCE1C,LOC101928009,C1orf68,SPRR1B,SPRR2D,SPRR1A,LCE6A,SPRR2C,SPRR2B,LCE1B,CRNN,LCE1A,LCE2A,LELP1,LCE1E,SPRR2G,LCE3B,SPRR2A,SPRR2E,LCE3E,LCE3A,SMCP,LCE3C,LCE2C,LCE1F,PRR9,LCE5A	3	gain
5	CGTE_01	1	156925413	159683996	1q23.1-q23.2	OR6P1,CADM3,LOC646268,APCS,OR6N2,CYCSP52,IFI16,FCRL2,OR6N1,MNDA,OR10J1,CD1D,ETV3L,OR10Z1,ACKR1,CD1C,PYHIN1,OR10T2,AIM2,CD1B,OR10J5,FCRL4,CD1A,FCRL1,OR6K6,OR6K2,OR10K1,ARHGEF11,FCER1A,FCRL5,ETV3,KIRREL,CD5L,OR10J3,OR10X1,SPTA1,OR10R2,FCRL3,OR6K3,CRP,CADM3-AS1,OR10K2,CD1E,OR6Y1	3	gain
6	CGTE_01	1	214832129	218536906	1q41	GPATCH2,TGFB2-AS1,TGFB2,LINC00210,KCNK2,RRP15,ESRRG,SPATA17,LOC102723833,CENPF,KCTD3,USH2A,LOC101929631,SPATA17-AS1	3	gain
7	CGTE_01	2	137639709	149247964	2q22.2-q22.3	YY1P2,ORC4,MBD5,NXPH2,LRP1B,GTDC1,LOC101928386,HNMT,ZEB2-AS1,LINC01412,PABPC1P2,TEX41,ZEB2,ARHGAP15,LOC101928273,MIR7157,ACVR2A,THSD7B,KYNU,SPOPL	3	gain
8	CGTE_01	3	156638320	156645441	3q25.31	LEKR1	11	gain
9	CGTE_01	3	180679138	180689024	3q26.33	FXR1	4	gain
10	CGTE_01	4	524262	1809470	4p16.3	SPON2,MFSD7,TMEM129,LOC100130872,CRIPAK,CTBP1-AS2,SLC26A1,FGFR3,UVSSA,RNF212,IDUA,PCGF3,DGKQ,ATP5I,NKX1-1,TMED11P,LOC100129917,TMEM175,TACC3,GAK,SLBP,PDE6B,CPLX1,CTBP1,CTBP1-AS,FAM53A,MAEA,FGFRL1,MYL5,PIGG	1	loss
11	CGTE_01	4	57352532	57354249	4q12	SRP72	6	gain
12	CGTE_01	4	57899156	68340656	4q12-q13.1	IGFBP7,TECRL,MIR1269A,ADGRL3-AS1,LOC101927237,ADGRL3,MIR548AG1,EPHA5,CENPC,LOC401134,LOC101928851,EPHA5-AS1,IGFBP7-AS1	3	gain
13	CGTE_01	4	73988614	76439480	4q13.3-q21.1	PF4,CXCL8,ANKRD17,MTHFD2L,RCHY1,AFM,PF4V1,PPBP,CXCL3,CXCL6,RASSF6,CXCL2,AREG,LOC100507388,EPGN,PPBPP2,LOC441025,EREG,CXCL5,AFP,PARM1,ALB,BTC,CXCL1,LOC728040	3	gain
14	CGTE_01	5	126674797	130897788	5q31.1-q23.3	CHSY3,PRRC1,SLC27A6,MEGF10,SLC12A2,ADAMTS19-AS1,HINT1,CTXN3,MIR4633,RAPGEF6,CDC42SE2,KIAA1024L,ADAMTS19,ISOC1,LINC01184,MIR4460,FBN2,LYRM7	3	gain

15	CGTE_01	5	140187266	140238136	5q31.3	PCDHA4,PCDHA1,PCDHA5,PCDHA2,PCDHA7,PCDHA6,PCDHA8,PCDHA9,PCDHA10,PCDHA3	1	loss
16	CGTE_01	5	147010908	148586700	5q32	LOC102546294,SPINK13,SPINK7,LOC255187,FBXO38,ABLIM3,SH3TC2,SPINK14,SPINK1,SPINK6,ADRB2,C5orf46,JAKMIP2-AS1,SCGB3A2,SPINK9,SPINK5,JAKMIP2,HTR4	3	gain
17	CGTE_01	5	180376274	180429863	5q35.3	BTNL8,BTNL3	0	loss
18	CGTE_01	6	102337330	102372685	6q16.3	GRIK2	7	gain
19	CGTE_01	7	7274097	20199925	7p22.1-p21.2	SCIN,DGKB,TSPAN13,C1GALT1,AGMO,MEOX2,NXPH1,AHR,THSD7A,LRRC72,TWISTNB,ETV1,RPA3,LOC100505938,LOC101927630,SNX13,LOC101927668,LOC101927354,SOSTDC1,AGR3,FERD3L,KCCAT333,MIR3146,MEOX2-AS1,MACC1-AS1,BZW2,NDUFA4,PER4,ANKMY2,LOC101927391,GLCCI1,TMEM106B,UMAD1,PRPS1L1,AGR2,VWDE,PHF14,TMEM196,MACC1,LOC100505921,COL28A1,TWIST1,MIOS,ICA1,MIR1302-6,ISPD,ISPD-AS1,ARL4A,HDAC9	3	gain
20	CGTE_01	7	77580905	87516161	7q21.11-q21.12	DBF4,TMEM243,KIAA1324L,CACNA2D1,TP53TG1,RPL13AP17,LOC101927356,MAGI2,SEMA3A,CD36,CROT,SEMA3D,MAGI2-AS2,GRM3,GNAT3,SEMA3C,RUNDC3B,GNAI1,PHTF2,ABCB4,ABCB1,LOC100128317,MAGI2-AS3,LOC101927378,LOC101927269,DMTF1,PCLO,HGF,MIR548M,SEMA3E,SLC25A40	3	gain
21	CGTE_01	7	87525664	87526608	7q21.12	DBF4	7	gain
22	CGTE_01	7	113517940	117022253	7q31.1-q31.2	WNT2,LINC01392,ST7-OT3,TFEC,CAV2,MIR3666,LINC01393,TES,FOXP2,LINC01510,CAV1,MET,CAPZA2,ASZ1,LOC102724434,ST7-AS1,ST7-AS2,MDFIC,MIR6132,PPP1R3A,ST7,ST7-OT4	3	gain
23	CGTE_01	7	128470626	128533108	7q32.1	FLNC,KCP,ATP6V1F,LOC100130705	1	loss
24	CGTE_01	7	149430190	149529979	7q36.1	ZNF467,SSPO,KRBA1	1	loss
25	CGTE_01	7	150642408	150884874	7q36.1	ASB10,GBX1,NOS3,ATG9B,SLC4A2,ABCB8,FASTK,TMUB1,AGAP3,CDK5,KCNH2,ASIC3	1	loss
26	CGTE_01	8	2796097	4852016	8p23.2	CSMD1	3	gain
27	CGTE_01	8	73925882	73934635	8q21.11	TERF1	23	gain
28	CGTE_01	8	91094293	94777919	8q22.1-q21.3	RBM12B,FLJ46284,MIR8084,MIR4661,OTUD6B,TMEM67,SLC26A7,RUNX1T1,C8orf88,LINC01030,LINC00535,RBM12B-AS1,LOC102724710,LRRC69,OTUD6B-AS1,MIR7641-2,LINC00534,TRIQK,TMEM64,NECAB1,C8orf87,TMEM55A,CALB1,FAM92A1	3	gain
29	CGTE_01	8	104438165	104439534	8q22.3	DCAF13	15	gain
30	CGTE_01	8	104442755	121706198	8q23.2-q23.1	NOV,SAMD12-AS1,TRPS1,TAF2,EIF3H,EBAG9,EMC2,RIMS2,ZFPM2-AS1,TMEM74,EXT1,ZFPM2,ENY2,COL14A1,SYBU,DEPTOR,TRHR,AARD,ANGPT1,MIR3610,ABRA,MTBP,MAL2,EIF3E,DPYS,MED30,CSMD3,MIR2053,SNTB1,ENPP2,DSCC1,RSPO2,LINC01608,UTP23,SLC30A8,MRPL13,RAD21-AS1,COLEC10,DCSTAMP,PKHD1L1,LRP12,KCNV1,TNFRSF11B,NUDCD1,RAD21,LINC01609,SAMD12,OXR1,DCAF13,LINC00536	3	gain
31	CGTE_01	8	124789515	124798048	8q24.13	FAM91A1	4	gain
32	CGTE_01	9	7799507	14150308	9p23-p24.1	FLJ41200,MPDZ,SNORD137,LURAP1L,TYRP1,LURAP1L-AS1,PTPRD-AS1,TMEM261,PTPRD-AS2,LINC00583,PTPRD,NFIB	3	gain
33	CGTE_01	9	26984295	26990233	9p21.2	IFT74	164	gain
34	CGTE_01	9	117782851	122257926	9q33.1	LOC101928797,TLR4,BRINP1,PAPPA,PAPPA-AS1,SNORA70C,LOC101928775,LINC00474,ASTN2,TNC,DEC1,ASTN2-AS1,LOC101928748,TRIM32	3	gain
35	CGTE_01	9	139221204	139306777	9q34.3	SDCCAG3,GPSM1,DNLZ,PMPCA,DKFZP434A062,SNAPC4,CARD9	1	loss

36	CGTE_01	9	139390521	140435261	9q34.3	TPRN,DPP7,TOR4A,ENTPD2,C9orf173-AS1,NOTCH1,MIR4292,LOC100128593,FAM69B,LCNL1,FAM166A,TMEM210,RABL6,FBXW5,MIR126,SNORA17B,C9orf142,TUBB4B,MIR4479,EGFL7,SNHG7,UAP1L1,LCN6,C8G,MIR3621,MIR4673,CYSRT1,LRRC26,ABCA2,EDF1,EXD3,TRAF2,NRARP,CCDC183,MAN1B1-AS1,ENTPD8,LCN15,SNORA17A,LCN8,C9orf172,SSNA1,NOXA1,LCN10,AGPAT2,MAMDC4,LCN12,MAN1B1,RNF208,MIR6722,NPDC1,PHPT1,TMEM203,C9orf173,NELFB,FUT7,CLIC3,GRIN1,MIR7114,TMEM141,SLC34A3,PNPLA7,C9orf139,ANAPC2,MIR4674,PTGDS,CCDC183-AS1,NDOR1,RNF224,SAPCD2,NALT1,NSMF	1	loss
37	CGTE_01	11	279348	3685429	11p15.5-p15.4	MRPL23,LOC171391,TALDO1,PDDC1,SCT,SLC22A18,LOC143666,EPS8L2,CRACR2B,LRRC56,FAM99A,MUC6,CHID1,CD81,MRGPRG,SIGIRR,POLR2L,MIR210HG,OR7E12P,MIR4298,TSPAN32,PIDD1,IFITM3,KRTAP5-2,IGF2,KCNQ1DN,DEAF1,CTSD,MIR483,LINC01150,MIR4686,KCNQ1,IFITM1,MIR6744,SNORA52,ASCL2,MOB2,ANO9,TRPC2,IRF7,SYT8,MRGPRE,AP2A2,KRTAP5-6,TOLLIP-AS1,INS,NLRP6,HOTS,CD151,HRAS,PHLDA2,TRPM5,BRSK2,CDKN1C,IFITM2,MIR675,TMEM80,KRTAP5-5,SLC22A18AS,MUC5AC,RPLP2,IFITM10,LSP1,LINC01219,KRTAP5-1,MIR7847,CARS,ZNF195,LOC101927708,MUC5B,NAP1L4,H19,DUSP8,ATHL1,KRTAP5-AS1,DRD4,SNORD131,PANO1,PHRF1,CEND1,CDHR5,KCNQ1-AS1,OSBPL5,LMNTD2,PKP3,RASSF7,ART5,RNH1,TNNT3,TOLLIP,IFITM5,TSSC2,B4GALNT4,TSPAN4,MRGPRG-AS1,C11orf21,MRPL23-AS1,PTDSS2,FAM99B,KRTAP5-3,INS-IGF2,ART1,TSSC4,MUC2,TH,KCNQ1OT1,TNNI2,SLC25A22,SNORA54,KRTAP5-4,IGF2-AS,CD81-AS1,MIR210,PNPLA2	1	loss
38	CGTE_01	11	3687378	6221397	11p15.4	C11orf40,OR51L1,OR51M1,OR52B6,HBB,OR52D1,OR52I2,OR51G2,OR56A4,OR51V1,OR52W1,OR51B5,TRIM6-TRIM34,OR52N1,OR52N4,OR56A1,OR51F1,OR52E4,STIM1,OR51T1,HBD,RRM1,OLFM5P,BGLT3,OR52E8,OR52E6,OR56B1,HBBP1,HBG2,PGAP2,OR52E2,OR51A4,OR51Q1,OR52K1,OR52B4,OR52A5,HBE1,OR56A5,TRIM6,LOC100506082,HBG1,NUP98,TRIM5,OR52B2,TRIM22,TRIM34,OR52H1,OR51G1,OR52N2,TRIM68,OR51B2,OR56B4,OR51B4,UBQLNL,UBQLN3,OR52A1,MIR4687,OR52K2,OR51S1,OR51D1,OR51I2,OR52N5,TRIM21,OR51B6,OR51F2,OR52R1,RHOG,OR52I1,OR51I1,OR51A2,OR51E1,OR51E2,OR52J3,CHRNA10,MMP26,OR52L1,OR56A3,OR52M1,OR51A7	3	gain
39	CGTE_01	11	16010509	16766146	11p15.2-p15.1	SOX6,C11orf58	3	gain
40	CGTE_01	11	20949811	28119500	11p15.1-p14.2	NELL1,LINC01495,CCDC179,KIF18A,ANO3,FANCF,LIN7C,BDNF-AS,LOC105376671,LOC105376599,SLC5A12,MIR610,BBOX1-AS1,BBOX1,CCDC34,ANO5,LINC00678,MUC15,SLC17A6,LGR4,MIR8087,GAS2,LUZP2,MIR8054,SVIP,FIBIN,BDNF	3	gain
41	CGTE_01	11	28134787	28143794	11p14.1	METTL15	40	gain
42	CGTE_01	11	62781906	63276441	11q12.3	SLC22A8,LGALS12,SLC22A25,SLC22A9,SLC22A24,SLC22A10,HRASLS5	3	gain
43	CGTE_01	12	23915459	25801513	12p12.1	BCAT1,CASC1,MIR920,LRMP,C12orf77,SOX5,KRAS,LYRM5,LINC00477,LOC101928471,LMNTD1	4	gain
44	CGTE_01	12	78225164	88508999	12q21.32-q21.31	PAWR,MKRN9P,LRRIQ1,TSPAN19,SLC6A15,CCDC59,OTOGL,PPP1R12A,LOC101928449,PTPRQ,MIR548AL,LIN7A,MGAT4C,MYF6,MIR617,CEP290,NAV3,LINC01490,MIR4699,C12orf50,NTS,MYF5,TMTC2,C12orf29,RASSF9,MIR618,ACSS3,METTL25,MIR1252,MIR5692B,PPFIA2,ALX1,LOC102724663,SYT1	3	gain
45	CGTE_01	13	33232325	34395480	13q13.2-q13.1	LINC00423,STARD13-AS,STARD13,KL,RFC3,PDS5B	3	gain

46	CGTE_01	13	37593419	40253804	13q13.3-q14.11	LINC00571,PROSER1,CSNK1A1L,POSTN,LHFP,COG6,LINC00366,SUPT20H,FREM2,UFM1,LINC01048,STOML3,LINC00437,LINC00547,NHLRC3,TRPC4,MIR4305	3	gain
47	CGTE_01	13	48919205	48923282	13q14.2	RB1	4	gain
48	CGTE_01	13	61986973	72147771	13q21.32-q21.2	LINC00358,PCDH9,PCDH20,LINC00448,PCDH9-AS4,LINC01075,PCDH9-AS2,LINC01052,KLHL1,LINC00395,LINC00550,MIR4704,OR7E156P,DACH1,LINC00383,LINC00364,PCDH9-AS3,LINC00376,LOC102723968,ATXN8OS,MIR548X2,LINC00348	3	gain
49	CGTE_01	13	79233136	95232028	13q32.1-q31.2	LINC00353,MIR19B1,LOC105370306,LINC00333,LINC01080,SPRY2,LINC00379,MIR20A,LINC00375,LINC01049,LINC00559,LINC00440,LINC00397,GPC6,SNORA107,NDFIP2,LINC01047,SLITRK1,LINC00382,RBM26,GPC5,LINC00351,SLITRK5,GPC5-AS1,MIR18A,LINC01038,LINC00433,TGDS,MIR92A1,GPC6-AS2,NDFIP2-AS1,MIR622,LINC00363,MIR4500,GPC5-AS2,LINC00331,LINC00380,LINC00377,LINC01068,LINC01040,GPC6-AS1,MIR19A,LINC00410,MIR4500HG,DCT,MIR17HG,LINC00430,RBM26-AS1,LINC00564,RNF219,MIR17,SLITRK6	3	gain
50	CGTE_01	14	20201784	20770035	14q11.2	OR4K5,OR4K14,OR11H4,OR4K2,OR4K1,OR4N2,TTC5,OR4M1,OR11G2,OR4Q3,OR11H6,OR4L1,OR4K17,OR4K15,OR4N5,OR4K13	3	gain
51	CGTE_01	14	21994209	22918160	14q11.2	OR4E2,SALL2,OR10G3,OR4E1,OR10G2	3	gain
52	CGTE_01	14	24906371	31122881	14q12	G2E3,MIR548AI,SCFD1,GZMH,KHNYN,LOC101927045,LOC101927062,LINC01551,LOC102724890,SDR39U1,CTSG,NOVA1,LINC00645,MIR3171,CMA1,STXBP6,FOXG1-AS1,FOXG1,MIR4307HG,GZMB,PRKD1,MIR4307	3	gain
53	CGTE_01	15	63947930	64041752	15q22.31	HERC1	3	gain
54	CGTE_01	16	569699	1559937	16p13.3	WDR24,SSTR5,NHLRC4,JMJD8,C1QTNF8,MSLN,CACNA1H,HAGHL,TPSG1,PTX4,LINC00235,GNG13,UBE2I,CCDC78,BAIAP3,MIR662,CLCN7,TSR3,LMF1,CCDC154,PRR35,PIGQ,CAPN15,STUB1,FAM173A,NARFL,RAB40C,UNKL,SSTR5-AS1,CHTF18,TELO2,PRR25,RAB11FIP3,METRN,LMF1-AS1,TPSD1,MIR3176,FBXL16,MIR5587,WDR90,RPUSD1,GNPTG,WFIKKN1,RHBDL1,SOX8,TPSB2,TPSAB1,RHOT2,C16orf91,FAM195A,C16orf13	1	loss
55	CGTE_01	16	1812254	1884418	16p13.3	NME3,MRPS34,MAPK8IP3,MEIOB,NUBP2,EME2,FAHD1,IGFALS,SPSB3,HAGH	1	loss
56	CGTE_01	16	59772750	66413492	16q21	CDH8,LOC101927650,CDH11,LOC729159,APOOP5,CDH5,LOC101927580,MIR4426,LINC00922	3	gain
57	CGTE_01	16	88134292	89266065	16q24.2-q24.3	SLC22A31,GALNS,SNAI3,APRT,CDT1,LOC400553,CDH15,LOC339059,IL17C,CTU2,ZNF469,CBFA2T3,SNAI3-AS1,LOC101928880,MIR4722,LOC100289580,LOC100129697,CYBA,PIEZO1,LOC400558,RNF166,ZC3H18,TRAPPC2L,MVD,ACSF3,ZFPM1,LINC00304,MIR5189,PABPN1L	1	loss
58	CGTE_01	17	38816267	39623762	17q21.2	KRT222,KRTAP1-3,KRTAP9-9,KRTAP3-3,KRT38,KRTAP1-1,KRTAP2-1,KRTAP4-9,KRTAP2-4,KRTAP9-2,KRTAP9-1,KRTAP4-4,KRTAP9-3,KRT25,KRT37,KRTAP4-11,KRTAP17-1,KRT31,KRTAP4-8,KRTAP1-4,KRT40,KRT10,LOC100505782,KRT33A,KRT27,KRT39,KRTAP4-6,KRTAP9-7,KRTAP3-2,KRTAP3-1,KRT12,KRT34,KRTAP4-2,KRTAP4-3,KRTAP2-2,KRTAP1-5,TMEM99,KRTAP29-1,KRT33B,KRT32,KRTAP9-8,KRTAP16-1,KRTAP4-7,KRT24,KRTAP9-6,KRTAP2-3,KRTAP4-5,KRTAP9-4,KRTAP4-1,KRT20,KRT26,KRT23,KRTAP4-12,KRT28	3	gain
59	CGTE_01	18	9124945	9134187	18p11.22	NDUFV2,NDUFV2-AS1	0	loss
60	CGTE_01	18	30846877	32428424	18q12.1	ASXL3,CCDC178,NOL4,DTNA	3	gain
61	CGTE_01	18	39576542	39595579	18q12.3	PIK3C3	3	gain
62	CGTE_01	18	44398331	44408095	18q21.1	PIAS2	4	gain
63	CGTE_01	19	23557365	23557914	19p12	ZNF91	16	gain

64	CGTE_01	19	46543041	46734174	19q13.32	LOC400706,IGFL4,IGFL2,DKFZp434J0226,IGFL3,IGFL1	3	gain
65	CGTE_01	19	54802017	54804664	19q13.42	LILRA3	0	loss
66	CGTE_01	20	1569040	1592350	20p13	SIRPB1	5	gain
67	CGTE_01	20	58453197	58533860	20q13.33	SYCP2,PPP1R3D,CDH26,FAM217B	3	gain
68	CGTE_01	20	60882184	62203687	20q13.33	OGFR-AS1,SLCO4A1-AS1,ADRM1,NKAIN4,RBBP8NL,C20orf166-AS1,COL20A1,KCNQ2,MIR3196,DPH3P1,MIR4326,LINC00659,LINC00029,SRMS,MIR1-1,DIDO1,CABLES2,OGFR,RPS21,HELZ2,PTK6,MIR124-3,GID8,ARFGAP1,HAR1A,GATA5,NTSR1,LAMA5,YTHDF1,TCFL5,PPDPF,FLJ16779,LAMA5-AS1,LOC100130587,LINC01056,EEF1A2,C20orf195,MIR1-1HG,CHRNA4,HAR1B,SLCO4A1,SLC17A9,COL9A3,BHLHE23,MRGBP,BIRC7,LOC63930,MIR133A2,MIR4758	1	loss
69	CGTE_01	21	31311664	32253929	21q22.11-q21.3	KRTAP19-5,GRIK1,KRTAP27-1,KRTAP21-2,KRTAP20-4,KRTAP24-1,KRTAP6-3,KRTAP20-3,MIR4327,KRTAP13-3,KRTAP19-3,KRTAP20-2,KRTAP11-1,KRTAP19-1,KRTAP20-1,KRTAP22-1,KRTAP15-1,KRTAP21-3,KRTAP19-4,KRTAP6-1,KRTAP23-1,KRTAP26-1,CLDN17,KRTAP25-1,CLDN8,KRTAP6-2,KRTAP13-1,LINC00307,KRTAP19-7,KRTAP21-1,KRTAP13-2,KRTAP22-2,KRTAP19-6,KRTAP7-1,KRTAP13-4,KRTAP8-1,KRTAP19-2	3	gain
70	CGTE_01	21	38467719	38470260	21q22.13	TTC3	16	gain
71	CGTE_01	21	46902570	47581413	21q22.3	SLC19A1,COL6A2,LOC100129027,FTCD,COL18A1,COL6A1,SPATC1L,PCBP3,LOC101928796	1	loss
72	CGTE_01	22	50609165	51178618	22q13.33	SYCE3,SBF1,PANX2,DENND6B,PLXNB2,SCO2,ADM2,MIOX,ARSA,SHANK3,TUBGCP6,TRABD,SELO,NCAPH2,MAPK11,CHKB-AS1,LMF2,MAPK12,CPT1B,ODF3B,CHKB-CPT1B,TYMP,CHKB,HDAC10,MAPK8IP2,ACR,KLHDC7B,PPP6R2	1	loss
73	CGTE_02	1	2535209	2939383	1p36.32	MMEL1,ACTRT2,TTC34	1	loss
74	CGTE_02	1	3394940	3477362	1p36.32	ARHGEF16,MEGF6,MIR551A	1	loss
75	CGTE_02	1	29644302	29650308	1p35.3	PTPRU	0	loss
76	CGTE_02	1	228459619	228479809	1q42.13	OBSCN	1	loss
77	CGTE_02	1	228558737	228563939	1q42.13	OBSCN	0	loss
78	CGTE_02	2	55791964	55800876	2p16.1	PPP4R3B	0	loss
79	CGTE_02	2	128382945	128388817	2q14.3	MYO7B	1	loss
80	CGTE_02	2	197744704	197758057	2q33.1	PGAP1	1	loss
81	CGTE_02	2	197761845	197786969	2q33.1	PGAP1	3	gain
82	CGTE_02	2	201397638	201401012	2q33.1	SGOL2	0	loss
83	CGTE_02	2	220347788	220355707	2q35	SPEG	1	loss
84	CGTE_02	2	233404337	233405564	2q37.1	CHRNG	0	loss
85	CGTE_02	3	49688283	49701348	3p21.31	BSN	1	loss
86	CGTE_02	3	52521197	52558572	3p21.1	STAB1,NT5DC2,NISCH	1	loss
87	CGTE_02	4	1806029	1827578	4p16.3	LETM1,FGFR3	1	loss
88	CGTE_02	4	2259640	2273541	4p16.3	MXD4,ZFYVE28	1	loss
89	CGTE_02	4	26321380	26388081	4p15.2	RBPJ	1	loss
90	CGTE_02	4	70801612	70823574	4q13.3	CSN1S1,CSN2	1	loss
91	CGTE_02	5	37003351	37014908	5p13.2	NIPBL	1	loss
92	CGTE_02	5	137780948	137802679	5q31.2	REEP2,EGR1	0	loss
93	CGTE_02	5	176297414	176309118	5q35.2	HK3,UNC5A	0	loss
94	CGTE_02	6	30858539	30863284	6p21.33	DDR1,MIR4640	1	loss
95	CGTE_02	6	30994645	31002409	6p21.33	MUC22	3	gain
96	CGTE_02	6	70506626	70610226	6q13	LMBRD1,COL19A1	1	loss
97	CGTE_02	7	44146371	44149943	7p13	AEBP1	1	loss
98	CGTE_02	7	44279168	44281422	7p13	CAMK2B	3	gain
99	CGTE_02	7	73008617	73022176	7q11.23	MLXIPL	0	loss

100	CGTE_02	7	73245481	73279795	7q11.23	WBSCR27,CLDN4,WBSCR28	1	loss
101	CGTE_02	7	77026365	77227224	7q11.23	GSAP,PTPN12,LOC101927243	1	loss
102	CGTE_02	7	100283617	100285885	7q22.1	GIGYF1	0	loss
103	CGTE_02	7	101921207	101925292	7q22.1	CUX1	0	loss
104	CGTE_02	7	149473040	149523373	7q36.1	SSPO	1	loss
105	CGTE_02	7	150656628	150696631	7q36.1	KCNH2,NOS3	0	loss
106	CGTE_02	7	150773099	150813954	7q36.1	AGAP3,FASTK,SLC4A2,TMUB1	1	loss
107	CGTE_02	8	143745758	143857184	8q24.3	THEM6,LY6K,JRK,PSCA,LYNX1,SLURP1,LYPD2,LOC100288181	1	loss
108	CGTE_02	8	143857219	143867988	8q24.3	LY6D,LYNX1	4	gain
109	CGTE_02	8	144898752	145926361	8q24.3	MROH1,RECQL4,BOP1,GPAA1,MIR6893,WDR97,MIR6849,MAF1,TONSL,PARP10,C8orf82,OPLAH,CYHR1,TMEM249,PUF60,SHARPIN,KIFC2,CYC1,LRRC14,MIR6847,FOXH1,LRRC24,TONSL-AS1,MIR7112,FBXL6,SPATC1,GPT,EPPK1,MIR6845,HSF1,GRINA,MIR6846,EXOSC4,HGH1,SLC39A4,DGAT1,ARHGAP39,CPSF1,MIR939,SCX,PPP1R16A,MIR661,SCRT1,SLC52A2,ADCK5,NRBP2,MFSD3,MIR6848,VPS28,PLEC	1	loss
110	CGTE_02	9	136426137	136439102	9q34.2	ADAMTSL2	0	loss
111	CGTE_02	9	139410425	139414075	9q34.3	MIR4673,NOTCH1	0	loss
112	CGTE_02	9	140389432	140395360	9q34.3	PNPLA7	0	loss
113	CGTE_02	9	141012938	141016420	9q34.3	CACNA1B	0	loss
114	CGTE_02	10	33113414	33137641	10p11.22	CCDC7	0	loss
115	CGTE_02	10	45472905	45478088	10q11.21	RASSF4,C10orf10	0	loss
116	CGTE_02	10	118030298	118138134	10q25.3	CCDC172,GFRA1	1	loss
117	CGTE_02	12	40876490	40885045	12q12	MUC19	3	gain
118	CGTE_02	12	100536308	100551847	12q23.1	GOLGA2P5,UHRF1BP1L	0	loss
119	CGTE_02	12	122212749	122261630	12q24.31	RHOF,SETD1B,TMEM120B,LINC01089	1	loss
120	CGTE_02	13	31732845	31736268	13q12.3	HSPH1	1	loss
121	CGTE_02	13	46820594	46841128	13q14.13	LRRC63	0	loss
122	CGTE_02	13	47470790	48547614	13q14.2	LINC00562,HTR2A,SUCLA2	3	gain
123	CGTE_02	13	53237221	53241143	13q14.3	SUGT1	1	loss
124	CGTE_02	13	113873200	113891147	13q34	CUL4A	3	gain
125	CGTE_02	13	114503726	114524037	13q34	GAS6,GAS6-AS1,TMEM255B	0	loss
126	CGTE_02	13	114524942	114531738	13q34	GAS6,GAS6-AS1	3	gain
127	CGTE_02	13	114535323	114538630	13q34	GAS6,GAS6-AS1	1	loss
128	CGTE_02	14	73717613	73735366	14q24.2	PAPLN	1	loss
129	CGTE_02	14	104575535	105057945	14q32.33	KIF26A,ASPG,MIR203A,MIR203B,TMEM179,C14orf180	1	loss
130	CGTE_02	15	25415952	25585280	15q11.2	SNORD115-14,SNORD115-42,SNORD115-26,SNORD115-44,SNORD115-2,SNORD115-23,SNORD115-21,SNORD115-11,SNORD115-22,UBE3A,SNORD115-47,SNORD115-43,SNORD115-31,SNORD115-3,SNORD115-12,SNORD115-6,SNORD115-8,PWAR4,SNORD115-36,SNORD115-7,SNORD115-34,SNORD115-41,SNORD115-38,SNORD115-13,SNORD115-30,SNORD115-45,SNORD115-20,SNORD115-35,SNORD115-18,SNORD109B,SNORD115-10,SNORD115-33,SNORD115-40,SNORD115-4,SNORD115-32,SNORD115-5,SNORD109A,SNORD115-25,SNORD115-27,SNORD115-15,SNORD115-29,SNORD115-9,SNORD115-17,SNORD115-48,SNORD115-46,SNORD115-37,SNORD115-28,SNORD115-16,SNORD115-24,SNORD115-19,SNORD115-39	3	gain
131	CGTE_02	15	60745830	60747580	15q22.2	ICE2	1	loss
132	CGTE_02	16	616900	943180	16p13.3	NHLRC4,RHOT2,WDR24,HAGHL,WDR90,FAM173A,PIGQ,MIR662,CCDC78,METRN,C16orf13,FAM195A,WFIKKN1,RPUSD1,GNG13,MSLN,CHTF18,RAB40C,FBXL16,JMJD8,STUB1,PRR25,RHBDL1,NARFL,LMF1	1	loss
133	CGTE_02	16	28506429	28513583	16p11.2	APOBR,IL27	1	loss
134	CGTE_02	16	88104956	88535504	16q24.2	MIR5189,LOC101928880,ZNF469,ZFPM1,BANP,LOC400553	1	loss

135	CGTE_02	17	4269495	4495796	17p13.2	SPNS3,GGT6,MYBBP1A,SMTNL2,UBE2G1,SPNS2	1	loss
136	CGTE_02	17	73732364	73759508	17q25.1	ITGB4,GALK1	1	loss
137	CGTE_02	17	78081316	78090896	17q25.3	GAA	0	loss
138	CGTE_02	18	739755	751980	18p11.32	YES1	1	loss
139	CGTE_02	18	8824627	8825974	18p11.22	MTCL1	1	loss
140	CGTE_02	18	19119798	19140949	18q11.2	ESCO1	3	gain
141	CGTE_02	18	44756702	44775202	18q21.1	SKOR2	1	loss
142	CGTE_02	18	66509274	66513765	18q22.1	CCDC102B	0	loss
143	CGTE_02	18	77170379	77208984	18q23	NFATC1	1	loss
144	CGTE_02	19	55857597	56161033	19q13.42	ZNF580,IL11,TMEM190,ZNF784,COX6B2,NAT14,SBK3,RPL28,ZNF581,SSC5D,ZNF524,FAM71E2,TMEM238,SHISA7,FIZ1,CCDC106,SBK2,ISOC2,ZNF579,ZNF628,ZNF865,MIR6805,KMT5C,UBE2S	1	loss
145	CGTE_02	20	3208382	3214966	20p13	SLC4A11	1	loss
146	CGTE_02	20	60881681	61150950	20q13.33	CABLES2,MIR1-1HG,RPS21,ADRM1,LAMA5,RBBP8NL,C20orf166-AS1,LAMA5-AS1,MIR4758,GATA5	1	loss
147	CGTE_02	20	62664196	62738309	20q13.33	PRPF6,MIR6813,LINC00176,OPRL1,NPBWR2,TCEA2,SOX18,RGS19,LKAAEAR1	1	loss
148	CGTE_02	21	45743631	45755728	21q22.3	PFKL,C21orf2	1	loss
149	CGTE_02	21	45970688	46117586	21q22.3	KRTAP12-3,TSPEAR,KRTAP10-4,KRTAP10-6,KRTAP10-7,KRTAP10-12,KRTAP10-3,KRTAP12-4,KRTAP10-11,KRTAP10-8,KRTAP10-9,KRTAP10-5,KRTAP10-10,KRTAP12-2,KRTAP10-2,KRTAP12-1	1	loss
150	CGTE_02	21	47418985	47575502	21q22.3	COL6A1,FTCD,COL6A2	1	loss
151	CGTE_02	22	28389091	28397538	22q12.1	TTC28,TTC28-AS1	0	loss
152	CGTE_02	22	29885087	29907232	22q12.2	THOC5,NEFH	3	gain
153	CGTE_02	X	83372344	83442937	Xq21.1	RPS6KA6	1	loss
154	CGTE_02	X	114413987	114424946	Xq23	LRCH2,RBMXL3	0	loss
155	CGTE_02	X	152864479	152958189	Xq28	SLC6A8,PNCK,DUSP9,LOC105373383,FAM58A	1	loss
156	CGTE_02	X	152994685	153050948	Xq28	PLXNB3,ABCD1,SRPK3	1	loss
157	CGTE_02	X	153678592	153697856	Xq28	MIR6858,PLXNA3,FAM50A	1	loss
158	CGTE_03	1	185277924	198828280	1q31.2-q31.3	KCNT2,MIR548F1,CFHR1,RGS18,PRG4,HMCN1,MIR4426,LINC01350,LINC01031,PTPRC,LINC01351,TROVE2,CFH,CFHR3,MIR181B1,LOC102724919,UCHL5,LINC01032,ATP6V1G3,CRB1,MIR1278,CDC73,ZBTB41,C1orf27,DENND1B,F13B,CFHR4,BRINP3,RGS13,RGS2,TPR,ASPM,MIR181A1HG,CFHR5,IVNS1ABP,CFHR2,NEK7,PACERR,C1orf53,GLRX2,OCLM,RGS21,RGS1,PDC,GS1-279B7.1,MIR181A1,LINC01037,PTGS2,MIR4735,LINC01036,LHX9,LOC440704,PLA2G4A,B3GALT2	1	loss
159	CGTE_03	1	214830342	220091961	1q41	USH2A,TGFB2-AS1,KCNK2,LYPLAL1,MIR548F3,RNU5F-1,LYPLAL1-AS1,SPATA17-AS1,KCTD3,RRP15,TGFB2,SPATA17,LINC00210,TGFB2-OT1,LOC102723833,LOC101929631,ESRRG,SLC30A10,CENPF,GPATCH2	1	loss
160	CGTE_03	2	287503	672963	2p25.3	TMEM18,FAM150B	6	gain
161	CGTE_03	2	74884878	84518086	2p11.2-p13.1	LOC100507201,MRPL19,GCFC2,REG3A,REG3G,REG1B,MIR8080,LRRTM4,SNAR-H,CTNNA2,LOC1720,EVA1A,REG1A,HK2,LOC101927987,POLE4,SEMA4F,LOC101927948,LOC101927967,REG1CP,LOC101927884,LINC01291,LRRTM1,LOC101927907,FUNDC2P2,MIR5000,LOC101927926,TACR1,MIR4264	1	loss
162	CGTE_03	2	208601936	208615914	2q33.3	CCNYL1	3	gain
163	CGTE_03	3	77147119	97510704	3p12.3-p11.1	MIR5688,ROBO1,SNORA95,LINC00506,C3orf38,LOC101927374,LINC00971,MIR6730,CHMP2B,LINC00879,ARL13B,GBE1,ZNF654,POU1F1,MIR3923,NSUN3,STX19,CGGBP1,EPHA6,MIR4795,CADM2-AS2,PROS1,ARL6,HTR1F,CADM2,ROBO2,EPHA3,LOC728290,VGLL3,MIR8060,MTHFD2P1,DHFRL1	1	loss

164	CGTE_03	3	130279116	132431318	3q22.1	ATP2C1,LOC339874,PIK3R4,NUDT16,DNAJC13,NEK11,NPHP3,MIR5704,CPNE4,MRPL3,ACKR4,ACPP,UBA5,NPHP3-ACAD11,SNORA58,COL6A6,ACAD11,ASTE1,NUDT16P1	1	loss
165	CGTE_03	3	142671394	169540806	3q26.2-q25.31	IL12A,ERICH6-AS1,SIAH2,P2RY1,TM4SF1-AS1,MIR1263,PFN2,MFSD1,PAQR9-AS1,ANKUB1,SUCNR1,AADACP1,U2SURP,KCNAB1-AS1,LINC01213,WWTR1-AS1,TERC,C3orf79,RARRES1,PLSCR2,LXN,ZIC4,LINC01192,GOLIM4,PAQR9,LINC00880,LINC01324,SPTSSB,SMC4,IGSF10,HLTF,SLC9A9-AS1,SELT,CLRN1,ARHGEF26-AS1,KPNA4,CLRN1-AS1,AGTR1,PLCH1,PLSCR1,LOC101928105,SERPINI2,IQCJ-SCHIP1,BCHE,LOC100507537,LRRC34,TM4SF18,P2RY13,MIR3919,LRRIQ4,GPR171,TM4SF4,MED12L,B3GALNT1,SCHIP1,SLC33A1,SHOX2,LOC440982,ZBBX,MIR548H2,IQCJ-SCHIP1-AS1,RNF13,WDR49,AADACL2,ARHGEF26,TIPARP-AS1,MIR5186,WWTR1,GFM1,NMD3,SLITRK3,AADACL2-AS1,CCNL1,LINC01487,MIR16-2,LINC01214,SCARNA7,MBNL1,ACTRT3,KCNAB1-AS2,MYNN,LEKR1,CHST2,TM4SF1,TSC22D2,CPA3,SI,CP,KCNAB1,VEPH1,LOC646903,HLTF-AS1,MLF1,AADAC,MIR15B,PTX3,C3orf58,LOC100507661,LINC01327,GPR87,MME,P2RY12,PA2G4P4,PLOD2,PLSCR5,RAP2B,SSR3,MIR551B,ZIC1,SERPINI1,TMEM14EP,DHX36,LINC00886,LINC01100,MBNL1-AS1,EGFEM1P,PDCD10,CPB1,OTOL1,C3orf33,PPM1L,EIF2A,GYG1,LINC00881,PQLC2L,LOC101243545,TIPARP,LOC100996447,P2RY14,GPR149,GMPS,C3orf80,COMMD2,HPS3,IL12A-AS1,LINC01322,SLC9A9,MECOM,RSRC1,TRIM59,SERP1,IFT80,ARL14,ERICH6,LINC01330,PLSCR4,IQCJ,LOC100289361	1	loss
166	CGTE_03	3	170136872	182665071	3q26.2-q26.32	TNFSF10,RNU6-2,MIR6828,KCCAT211,LINC01208,SOX2,MFN1,SOX2-OT,LOC101928882,SLC2A2,MIR7977,TBL1XR1,NLGN1,TTC14,LINC00578,LOC101928739,PEX5L,TMEM212,LOC102724604,FNDC3B,MRPL47,ACTL6A,MIR569,EIF5A2,CLDN11,SPATA16,LINC01206,LINC00501,NAALADL2-AS2,KCNMB2,NAALADL2,SLC7A14,NDUFB5,ECT2,LOC100505609,NAALADL2-AS1,ZNF639,LINC01209,DCUN1D1,GHSR,TNIK,USP13,ZMAT3,ATP11B,FLJ46066,KCNMB3,PEX5L-AS2,TMEM212-AS1,PIK3CA,GNB4,NAALADL2-AS3,MIR548AY,NCEH1,LINC01014,FXR1,PLD1,MIR4789,CCDC39,KCNMB2-AS1,NLGN1-AS1,DNAJC19,RPL22L1	1	loss
167	CGTE_03	3	186571900	193386346	3q28-q29	PYDC2,LOC101929106,LPP,IL1RAP,ATP13A4-AS1,TPRG1-AS1,ATP13A5-AS1,CLDN1,RPL39L,OPA1,TPRG1-AS2,CLDN16,UTS2B,FGF12-AS1,SST,BCL6,FLJ42393,LOC100131635,MIR944,TP63,OSTN,P3H2,MASP1,LPP-AS1,SNAR-I,OPA1-AS1,MGC2889,HRASLS,MIR28,LINCR-0002,RTP4,P3H2-AS1,TPRG1,ST6GAL1,ADIPOQ-AS1,TMEM207,ATP13A5,MB21D2,ATP13A4,LPP-AS2,CCDC50,RTP1,FGF12,ADIPOQ,OSTN-AS1,GMNC,RTP2	1	loss
168	CGTE_03	4	493077	648705	4p16.3	PIGG,PDE6B	3	gain
169	CGTE_03	4	15972651	16002248	4p15.32	PROM1	3	gain

170	CGTE_03	4	57899156	71397202	4q13.3-q13.1	PRR27,TMPRSS11E,IGFBP7,ODAM,SMR3A,CSN1S1,UGT2B17,MUC7,TMPRSS11BNL,TMPRSS11GP,TMPRSS11F,UGT2A3,SMR3B,SYT14P1,CSN2,AMTN,GNRHR,ADGRL3-AS1,PROL1,TMPRSS11B,UGT2A1,FTLP10,TMPRSS11A,MIR548AG1,UGT2B15,UGT2B7,MIR1269A,STAP1,UGT2A2,UGT2B10,HTN3,STATH,UGT2B4,SULT1E1,ADGRL3,LOC550113,FDCSP,UGT2B11,CABS1,UGT2B28,TMPRSS11D,IGFBP7-AS1,EPHA5-AS1,YTHDC1,CSN1S2AP,CSN1S2BP,UBA6-AS1,LOC401134,HTN1,UBA6,TECRL,SULT1B1,LOC101928851,EPHA5,CSN3,LOC101927237,CENPC	1	loss
171	CGTE_03	5	140384	1510973	5p15.33	LINC01511,SLC6A19,CTD-3080P12.3,TPPP,LPCAT1,TERT,ZDHHC11,PDCD6,SLC6A3,SLC12A7,NKD2,MIR4456,LOC100506688,LRRC14B,PP7080,SLC9A3,MIR4635,SLC6A18,CLPTM1L,PLEKHG4B,EXOC3,BRD9,SDHA,AHRR,HRAT5,MIR4457,MIR6075,LOC100996325,CCDC127,TRIP13,LOC100288152,CEP72,EXOC3-AS1	3	gain
172	CGTE_03	5	1523757	1880334	5p15.33	LPCAT1,LOC101929034,MRPL36,IRX4,NDUFS6,SDHAP3,MIR4277,LOC728613	4	gain
173	CGTE_03	5	16935804	31323297	5p13.3-p14.1	LOC101929660,LOC101929681,SNORA105B,C5orf17,LOC340107,BASP1,PMCHL1,LINC01021,SNORA105A,CDH10,CDH12,LSP1P3,MYO10,CDH6,LOC285696,LOC101929645,CDH18,CDH9,PRDM9,LOC401177,GUSBP1	1	loss
174	CGTE_03	5	37328357	37480087	5p13.2	NUP155,WDR70	4	gain
175	CGTE_03	5	81574028	125824753	5q15-q23.1	LOC100289230,EPB41L4A-AS2,SLCO6A1,CCNH,SLC25A46,LOC101927023,MIR1244-4,TNFAIP8,LINC01170,LINC01023,TSSK1B,LINC01340,RIOK2,ERAP1,ATG12,RGMB-AS1,TMEM167A,ARL14EPL,MIR1244-1,ZNF608,SLF1,MCC,COX7C,LINC00492,MIR1244-3,LINC00461,RAB9BP1,NR2F1,KCNN2,LOC101927460,PCSK1,POLR3G,HAPLN1,CDO1,MIR5706,RHOBTB3,CCDC112,TMED7-TICAM2,LOC102467212,LYSMD3,MIR4280,PRDM6,SNCAIP,LINC01338,LOC55338,CAMK4,MGC32805,SNX24,LOC644285,PPIC,LOC100289673,C5orf30,ERAP2,SPATA9,MIR548F3,LOC101929710,PRR16,EDIL3,TICAM2,DMXL1,TRIM36,MIR548AO,LOC102467216,MCTP1,GIN1,KIAA0825,WDR36,GRAMD3,COMMD10,LOC101927078,APC,ZNF474,ATP6AP1L,LINC00491,HRAT56,PPIP5K2,ARRDC3-AS1,MAN2A1,DTWD2,LOC102467213,LINC01339,HNCAT21,PGGT1B,LOC101929380,POU5F2,AP3S1,NREP,LOC100505878,LOC101927379,MIR9-2,LOC101927190,FAM170A,TMED7,GPR150,ARSK,EFNA5,LOC102467226,LOC102467223,SLCO4C1,VCAN,LOC100133050,NR2F1-AS1,FER,MEF2C-AS1,LIX1,RGMB,TTC37,LOX,GLRX,SRFBP1,SEMA6A,NUDT12,MEF2C,FEM1C,PAM,FAM81B,LUCAT1,HSD17B4,FBXL17,CSNK1G3,LNPEP,CEP120,TMEM161B-AS1,MIR1244-2,MIR583,LOC102467224,YTHDC2,CETN3,SNORA13,TSLP,RASA1,ADGRV1,FTMT,LOC102467217,PJA2,MIR2277,EPB41L4A-AS1,CHD1,LOC102467225,CAST,LVRN,FAM174A,TMEM161B,STARD4,FAM172A,SNX2,SRP19,MIR3660,LOC102546226,REEP5,ARRDC3,LOC101927100,LOC100505841,LOC101927421,RFESD,CTD-2151A2.1,LINC01554,MIR3977,LOC731157,EPB41L4A,MIR3607,ELL2,LOC102546228,LOC102467214,SEMA6A-AS1,SCARNA18,LINC00992,NBPF22P,NREP-AS1,MIR548P,XRCC4,LOC101927488,ST8SIA4,RPS23,TMEM232,DCP2,STARD4-AS1,MBLAC2	1	loss
176	CGTE_03	5	126161669	129521485	5q23.3-q23.2	MIR4460,MARCH3,SLC12A2,ISOC1,CHSY3,CTXN3,SLC27A6,MEGF10,FBN2,LMNB1,LINC01184,ADAMTS19,KIAA1024L,ADAMTS19-AS1,PRRC1,C5orf63,MIR4633	1	loss

177	CGTE_03	6	24871020	29571433	6p22.1-p22.3	HIST1H2AI,ZSCAN26,HIST1H2AK,LINC01015,FAM65B,LRRC16A,VN1R10P,OR2B3,GUSBP2,SCGN,ZSCAN12P1,HIST1H4L,HIST1H4F,ZNF192P1,HIST1H3F,BTN3A3,HIST1H2BL,HIST1H1A,ZSCAN12,HIST1H2AJ,CMAHP,HIST1H1B,HIST1H2BK,OR12D2,HIST1H2AA,HIST1H2AH,BTN3A2,OR2J2,BTN1A1,OR2B6,HIST1H1D,BTN2A3P,HIST1H3D,HIST1H3E,HIST1H2BG,LINC00240,OR14J1,TRIM27,HIST1H2AB,PRSS16,HIST1H2AG,GPX5,SLC17A1,HIST1H4B,TRIM38,OR2H2,OR11A1,HIST1H2BD,GPX6,HMGN4,OR10C1,HIST1H2BO,NKAPL,HIST1H4H,HCG11,ABT1,ZNF311,TOB2P1,ZNF165,HIST1H2BE,SLC17A4,LOC100270746,HIST1H4G,HIST1H1C,LINC01556,ZSCAN16,SLC17A2,OR5V1,UBD,OR2W1,BTN2A2,ZKSCAN8,HIST1H2BJ,HIST1H4C,HIST1H2BA,HIST1H3H,OR2J3,ZBED9,ZNF204P,HIST1H4I,HIST1H1T,PGBD1,LINC01012,HIST1H2AL,SLC17A3,ZKSCAN4,LOC285819,HIST1H3B,HIST1H2AD,HIST1H2BC,HIST1H2BB,HIST1H2AM,LINC01623,MIR3143,ZNF322,SNORD32B,HIST1H1E,ZSCAN31,POM121L2,OR2B2,HIST1H2AE,HIST1H3C,OR12D3,HIST1H3J,ZKSCAN3,HIST1H4J,HIST1H2BH,BTN2A1,ZSCAN16-AS1,GABBR1,HIST1H2BM,HIST1H4D,LOC101928663,HIST1H4K,HCG14,HIST1H2APS1,HIST1H2BI,LOC100131289,HIST1H2BN,HFE,ZSCAN9,LOC100129636,BTN3A1,HIST1H2BF,MAS1L,OR2H1,ZSCAN23,HIST1H3A,ZNF184,HIST1H4E,HIST1H4A,HIST1H3I,HIST1H2AC,HIST1H3G,ZNF391	1	loss
178	CGTE_03	6	53948242	74064005	6p11.1-p12.1	HCRTR2,SLC25A51P1,MIR30C2,MIR30A,LGSN,EYS,RAB23,FAM135A,LINC00680-GUSBP4,LOC102723883,BMP5,KHDRBS2,GFRAL,B3GAT2,LOC101928307,DST,KCNQ5,PTP4A1,EVADR,COL21A1,HMGCLL1,DPPA5,SDHAF4,KHDC1L,FAM83B,LINC01626,BAG2,KCNQ5-AS1,MTRNR2L9,ADGRB3,COL19A1,TINAG,PRIM2,GUSBP4,LINC00472,LMBRD1,MIR548U,MIR4282,LOC101927211,LOC101930010,LOC441155,RIMS1,SMAP1,KCNQ5-IT1,LOC100506188,LOC101928280,ZNF451,PHF3,KIAA1586,LINC00680,OGFRL1,COL9A1,KHDC1,MLIP,BEND6	1	loss
179	CGTE_03	6	74072468	74354351	6q13	EEF1A1,MB21D1,MTO1,SNORD141B,KHDC3L,OOEP,DDX43,SNORD141A,SLC17A5	3	gain
180	CGTE_03	6	90659756	105192504	6q16.1-q16.3	KLHL32,SIM1,BACH2,MCHR2,MANEA-AS1,UFL1,MIR4464,EPHA7,USP45,MAP3K7,CASC6,MIR2113,FUT9,POU3F2,MCHR2-AS1,PNISR,PRDM13,MANEA,MMS22L,FBXL4,ASCC3,MIR548H3,CCNC,NDUFAF4,TSTD3,FAXC,LOC101927314,MIR4643,TSG1,GRIK2,COQ3,GPR63,FHL5,LOC101927365,HACE1	1	loss
181	CGTE_03	7	208660	2304128	7p22.3	NUDT1,PRKAR1B,C7orf50,DNAAF5,FAM20C,FTSJ2,GPER1,ELFN1-AS1,MIR339,SUN1,UNCX,PSMG3-AS1,LOC442497,TFAMP1,SNX8,LOC101927021,LOC101926963,COX19,WI2-2373I1.2,ZFAND2A,MAD1L1,MICALL2,GET4,HRAT92,INTS1,ADAP1,MIR4655,CYP2W1,LOC101927000,TMEM184A,ELFN1,MAFK,MIR6836,PDGFA,GPR146,PSMG3	3	gain

182	CGTE_03	7	7273811	20691196	7p21.2-p21.3	ANKMY2,PRPS1L1,ISPD,AGR3,MEOX2-AS1,MEOX2,AGMO,BZW2,RPA3,LOC101927769,SNX13,ISPD-AS1,MIR3146,GLCCI1,VWDE,PER4,LOC101927668,TWIST1,TSPAN13,LOC100505921,LOC101927630,LOC100505938,AGR2,TWISTNB,LOC101927391,ICA1,C1GALT1,ABCB5,AHR,UMAD1,PHF14,MACC1,TMEM106B,ITGB8,ETV1,NDUFA4,TMEM196,ARL4A,LOC101927811,SOSTDC1,LOC101927354,THSD7A,SCIN,LRRC72,HDAC9,DGKB,FERD3L,MIOS,MACC1-AS1,NXPH1,COL28A1,KCCAT333,MIR1302-6	1	loss
183	CGTE_03	7	77754993	86814437	7q21.11-q21.12	SEMA3D,HGF,RPL13AP17,LOC100128317,MAGI2-AS3,MIR548M,CD36,MAGI2,GNAT3,LOC101927378,GNAI1,LOC101927269,GRM3,CACNA2D1,DMTF1,SEMA3A,SEMA3E,MAGI2-AS2,SEMA3C,PCLO,LOC101927356,KIAA1324L	1	loss
184	CGTE_03	7	100953912	102063901	7q22.1	SH2B2,CUX1,COL26A1,IFT22,MIR548O,LOC100289561,MYL10,LOC101927746,LOC100630923,MIR4285,LINC01007,PRKRIP1,SPDYE6	3	gain
185	CGTE_03	7	135390659	137374849	7q33	MIR490,LOC349160,PTN,SLC13A4,LUZP6,DGKI,FAM180A,MTPN,CHRM2	1	loss
186	CGTE_03	7	143105849	148311424	7q35-q36.1	CTAGE8,CNTNAP2,MIR548T,OR2A5,EPHA1-AS1,TCAF2,OR2A12,OR6B1,LOC101928605,TCAF2P1,CTAGE15,ARHGEF34P,OR2A9P,TAS2R41,LOC154761,CTAGE6,CTAGE4,OR2A14,OR2F1,OR2A2,EPHA1,MIR548F4,OR2F2,NOBOX,OR2A1-AS1,ARHGEF5,OR2A42,TPK1,OR2A7,OR2A1,OR2A20P,ARHGEF35,LOC101928700,TCAF1,C7orf33,TAS2R60,OR2A25,MIR548I4	1	loss
187	CGTE_03	7	156752407	158935255	7q36.3	LOC100506585,DNAJB6,LOC101927914,NOM1,WDR60,MIR153-2,ESYT2,MIR5707,LINC01022,NCAPG2,MNX1-AS1,LINC00689,MIR595,VIPR2,UBE3C,MNX1,PTPRN2	3	gain
188	CGTE_03	8	2796097	6682822	8p23.1-p23.2	MCPH1-AS1,LOC100287015,CSMD1,MCPH1,MIR4659B,XKR5,MIR8055,AGPAT5,ANGPT2,MIR4659A	1	loss
189	CGTE_03	8	104145018	122916494	8q24.12-q24.13	SNTB1,DPYS,BAALC-AS1,TNFRSF11B,DEPTOR,HAS2,COLEC10,DCAF13,TRPS1,UTP23,LINC01609,DCSTAMP,SAMD12-AS1,ENY2,ENPP2,KCNV1,TMEM74,EIF3E,ZFPM2-AS1,SLC30A8,ANGPT1,EXT1,TAF2,BAALC-AS2,SLC25A32,LINC01608,NUDCD1,ABRA,EMC2,SAMD12,MRPL13,CSMD3,AARD,ZFPM2,OXR1,RAD21,COL14A1,MTBP,MAL2,NOV,MIR2053,LOC101927543,MIR3151,RAD21-AS1,LINC00536,FZD6,PKHD1L1,BAALC,EBAG9,EIF3H,MIR3610,DSCC1,RIMS2,LRP12,CTHRC1,HAS2-AS1,MED30,RSPO2,TRHR,SYBU	1	loss
190	CGTE_03	9	6601915	6814754	9p24.1	GLDC,KDM4C	3	gain
191	CGTE_03	9	6849500	19116696	9p23-p22.2	CCDC171,MPDZ,SAXO1,FLJ41200,MIR3152,RRAGA,FREM1,BNC2,LINC00583,PSIP1,ADAMTSL1,SCARNA8,LURAP1L,TYRP1,KDM4C,LURAP1L-AS1,PLIN2,LOC389705,ZDHHC21,PTPRD,SNORD137,CNTLN,PTPRD-AS2,C9orf92,PTPRD-AS1,CER1,TTC39B,TMEM261,SNAPC3,NFIB,SH3GL2,HAUS6	1	loss
192	CGTE_03	9	19118074	19123716	9p22.1	PLIN2	4	gain

193	CGTE_03	9	19125929	28889000	9p21.3-p21.1	IFNW1,IFNA13,IFNA1,IFNA2,MIR31HG,MIR4473,PLAA,LINC01239,IFNA10,KLHL9,MOB3B,CDKN2A-AS1,IFNA4,HACD4,C9orf72,SLC24A2,DENND4C,MTAP,MIR4474,LINC01241,IFNA22P,LOC100506422,MIR876,CDKN2A,RPS6,IFNA8,IFNA5,IFNB1,MIR31,TEK,PLIN2,IFNE,MIR491,LOC101929563,FOCAD,IFNA14,EQTN,DMRTA1,TUSC1,ELAVL2,IFNA17,IFNA6,CDKN2B-AS1,IFT74-AS1,IFNA21,CDKN2B,LRRC19,IFNA16,FOCAD-AS1,ACER2,LINC00032,CAAP1,IFNK,IFT74,MIR873,MLLT3,IFNA7,LINGO2,IZUMO3	1	loss
194	CGTE_03	9	117121996	122075634	9q33.1-q32	ASTN2,AKNA,ASTN2-AS1,BRINP1,SNORA70C,TNFSF8,TLR4,TNC,DEC1,ATP6V1G1,LOC100505478,PAPPA,LOC101928748,PAPPA-AS1,DFNB31,LOC101928775,TRIM32,LOC101928797,LINC00474,TNFSF15,C9orf91	1	loss
195	CGTE_03	9	138852816	138899253	9q34.3	UBAC1,NACC2	7	gain
196	CGTE_03	10	43607517	43617507	10q11.21	RET	4	gain
197	CGTE_03	10	51549305	69990938	10q21.1-q21.2	ANK3,EGR2,C10orf107,TMEM26,FAM21EP,A1CF,POU5F1P5,PCDH15,TIMM23,LINC00844,JMJD1C,ARID5B,MIR7151,FAM21A,HERC4,DNAJC12,IPMK,CDK1,ANXA2P3,BICC1,LINC01553,LINC00845,PRKG1-AS1,ASAH2B,RTKN2,LOC101928961,MYPN,CCDC6,CCEPR,MIR605,CTNNA3,MTRNR2L5,JMJD1C-AS1,LINC01515,PHYHIPL,LOC102724719,RHOBTB1,MIR3924,AGAP6,LOC283045,ASAH2,SGMS1-AS1,CISD1,REEP3,MIR548AV,LINC01468,SGMS1,PRKG1,LRRTM3,NCOA4,CSTF2T,ADO,ATOH7,MBL2,ZWINT,TMEM26-AS1,MRLN,FAM133CP,FAM13C,MIR1296,UBE2D1,NRBF2,SIRT1,TIMM23B,MSMB,DKK1,TFAM,SLC16A9,ZNF365	1	loss
198	CGTE_03	10	120820221	121259765	10q26.11	PRDX3,RGS10,EIF3A,SFXN4,GRK5,FAM45BP,FAM45A,MIR4681	3	gain
199	CGTE_03	10	131641582	135184358	10q26.3	DPYSL4,LINC01168,LINC01166,MIR202,C10orf91,MIR202HG,ADAM8,PWWP2B,UTF1,NKX6-2,PPP2R2D,TCERG1L-AS1,ZNF511,PRAP1,LINC01167,ADGRA1,ADGRA1-AS1,BNIP3,INPP5A,MIR378C,JAKMIP3,LINC00959,LINC01164,LRRC27,EBF3,CTAGE7P,GLRX3,TUBGCP2,VENTX,CALY,STK32C,KNDC1,CFAP46,ECHS1,FUOM,TCERG1L,MIR4297	3	gain
200	CGTE_03	11	1217087	1284336	11p15.5	MUC5B,MIR6744,MUC5AC	1	loss
201	CGTE_03	11	1284337	1501756	11p15.5	BRSK2,MOB2,TOLLIP-AS1,TOLLIP	4	gain
202	CGTE_03	11	4409368	6232349	11p15.4	HBD,OR52A5,OR52I2,OR51T1,OR51F2,OR56B1,OR56A4,OR51I1,TRIM21,OR51B2,UBQLN3,OR51A2,OR51L1,OR51I2,OR52K2,OR51F1,OR52K1,HBBP1,OR52H1,OR56B4,MMP26,TRIM34,OR52N2,OR52E4,OR52A1,TRIM6-TRIM34,UBQLNL,OR52E2,OR52N1,OR52J3,OR52R1,OR51M1,OR51D1,OR51S1,OR51A4,OR52N5,TRIM6,OR52M1,OR51E2,C11orf42,OR51B5,OR52B6,OR51B4,OLFM5P,HBE1,TRIM22,OR56A3,HBG1,OR56A1,TRIM68,OR52E8,HBB,OR52N4,OR52W1,OR51G2,OR51Q1,OR51B6,OR51V1,OR52L1,TRIM5,OR52D1,OR52I1,OR52E6,OR51A7,OR52B2,OR51G1,OR51E1,BGLT3,OR56A5,HBG2,C11orf40	1	loss
203	CGTE_03	11	10472505	10655649	11p15.4	AMPD3,RNF141,MTRNR2L8,MRVI1,MRVI1-AS1,LYVE1,MIR4485	3	gain
204	CGTE_03	11	20949668	26581308	11p14.2-p15.1	MIR8054,LUZP2,ANO5,LOC105376599,MUC15,GAS2,ANO3,LINC01495,FANCF,CCDC179,NELL1,SVIP,SLC17A6	1	loss
205	CGTE_03	11	27695577	31805147	11p14.1-p13	MIR8068,BDNF,ELP4,FSHB,BDNF-AS,MPPED2,ARL14EP,IMMP1L,KIF18A,DCDC5,DNAJC24,KCNA4,MIR610,METTL15,DCDC1,LINC01616	1	loss

206	CGTE_03	11	48238250	57004521	11p11.12-q12.1	TRIM64C,OR8K3,OR4X2,LOC440040,OR5T1,OR4C46,OR4A15,OR8U8,LOC101927120,OR4C15,OR5D18,OR4P4,OR5J2,OR5M1,OR4B1,OR4C13,OR7E5P,OR4C11,OR8K1,OR5T2,LOC646813,OR5W2,OR4A16,LRRC55,OR9G4,OR5D16,OR5AR1,OR8J1,APLNR,OR4C45,OR9G1,OR8H1,OR4A47,OR8U1,OR5L2,OR4A5,MIR6128,OR5D13,OR5I1,OR5D14,OR5M3,OR8H3,OR4C6,OR5M10,OR9G9,FOLH1,OR8J3,OR5AK2,OR5F1,OR4C16,TRIM51,OR5R1,OR4S1,TRIM48,OR5T3,OR8K5,TRIM51HP,OR8H2,OR5M9,OR5L1,OR5AK4P,OR5M11,TRIM49B,OR8I2,OR5AP2,OR4C12,OR10AG1,LOC441601,OR5M8,OR4X1,OR4C3,OR4S2,OR5AS1	1	loss
207	CGTE_03	11	57583233	58715466	11q12.1	OR5B2,TMX2-CTNND1,OR5B17,OR10Q1,GLYAT,OR6Q1,LOC283194,CTNND1,OR9Q2,ZFP91,OR9Q1,CNTF,LPXN,OR5B12,GLYATL1,OR9I1,ZFP91-CNTF,OR1S1,OR1S2,GLYATL2,OR5B3,OR5B21,OR10W1	1	loss
208	CGTE_03	11	69241999	69482869	11q13.3	CCND1,LINC01488,ORAOV1	4	gain
209	CGTE_03	11	102962438	107506563	11q22.3	LOC101928535,CWF19L2,CASP5,LOC643733,GRIA4,KBTBD3,DCUN1D5,MIR4693,AASDHPPT,CASP1P2,CASP4,ALKBH8,LOC102723895,DDI1,ELMOD1,LOC643923,MIR7641-1,PDGFD,CARD16,DYNC2H1,CASP12,MSANTD4,CARD17,GUCY1A2,CARD18,CASP1	1	loss
210	CGTE_03	11	123601215	124440854	11q24.2-q24.1	OR10G7,OR8G1,OR8D2,OR8G5,OR6X1,OR10G4,OR8B2,OR8D4,OR6T1,OR10G8,OR4D5,ZNF202,TMEM225,OR8B4,OR8G2,OR6M1,OR10S1,OR8D1,OR10G9,OR8B8,OR8A1,VWA5A,OR8B3,OR8B12	1	loss
211	CGTE_03	12	7303435	7869575	12p13.31	CLSTN3,ACSM4,PEX5,DPPA3,GDF3,APOBEC1,CD163L1,CD163	1	loss
212	CGTE_03	12	7882219	8091099	12p13.31	SLC2A14,NANOG,NANOGNB,CLEC4C,SLC2A3	3	gain
213	CGTE_03	12	55039829	56076242	12q13.2	OR6C65,NEUROD4,MUCL1,OR6C6,OR6C76,TESPA1,OR6C2,OR6C75,OR6C68,OR6C1,OR9K2,OR6C70,OR6C74,OR2AP1,OR6C4,OR6C3,OR10P1,OR10A7,DCD,METTL7B	1	loss
214	CGTE_03	12	70507857	93246132	12q21.2-q21.1	MIR618,LUM,RASSF9,MGAT4C,LOC100507377,PPP1R12A,LIN7A,C12orf50,TSPAN19,ACSS3,LINC00615,CCER1,CCDC59,TRHDE-AS1,LRRIQ1,ZDHHC17,POC1B-GALNT4,E2F7,TRHDE,DUSP6,LINC01481,TSPAN8,OTOGL,SYT1,ATXN7L3B,MYF6,POC1B,TMTC3,EPYC,NAV3,C12orf74,GLIPR1L1,MIR4699,PPFIA2,LOC101928449,OSBPL8,PLEKHG7,KRR1,CNOT2,GLIPR1,KCNC2,LINC00936,KCNMB4,BBS10,LOC101928137,ZFC3H1,THAP2,RAB21,PTPRB,LGR5,TBC1D15,GALNT4,MIR548AL,C12orf29,BTG1,CLLU1,MRS2P2,SLC6A15,GLIPR1L2,ATP2B1,KERA,LINC01490,MIR617,LINC01619,CAPS2,PAWR,DCN,TMEM19,MKRN9P,LOC728084,CSRP2,PHLDA1,CLLU1OS,PTPRR,CEP290,LOC102724663,ALX1,NAP1L1,TPH2,MYF5,MIR5692B,METTL25,TMTC2,MIR1252,PTPRQ,KITLG,EEA1,NTS	1	loss
215	CGTE_03	13	101179677	101257299	13q32.3	TMTC4,PCCA,GGACT	3	gain
216	CGTE_03	14	20201784	20774126	14q11.2	OR4M1,OR11H4,OR4K13,OR11H6,OR4N2,OR4L1,OR4Q3,OR4K2,OR11G2,OR4K5,OR4K1,TTC5,OR4K14,OR4N5,OR4K17,OR4K15	1	loss
217	CGTE_03	14	21968546	22918160	14q11.2	METTL3,OR10G2,OR4E2,OR4E1,OR10G3,SALL2	1	loss
218	CGTE_03	14	24883706	31113262	14q12	GZMB,STXBP6,GZMH,LINC00645,MIR4307HG,LOC101927045,FOXG1-AS1,MIR4307,SDR39U1,CMA1,CTSG,SCFD1,LOC102724890,G2E3,LOC101927062,KHNYN,MIR548AI,LINC01551,MIR3171,PRKD1,NYNRIN,CBLN3,FOXG1,NOVA1	1	loss
219	CGTE_03	14	45715960	50065810	14q21.2-q21.3	LINC00871,LINC00648,MDGA2,LRR1,MIR548Y,RPS29,MIS18BP1,RPL10L	1	loss
220	CGTE_03	14	70795831	72169107	14q24.2	SIPA1L1,LOC145474,SNORD56B,PCNX,COX16,ADAM20,MAP3K9,LINC01269,ADAM20P1,SYNJ2BP-COX16,MED6,LOC101928075,SYNJ2BP,TTC9,ADAM21	1	loss

221	CGTE_03	14	78221388	88951573	14q31.2-q31.3	KCNK10,LOC100506700,LINC01467,DIO2,DIO2-AS1,LOC101928767,GTF2A1,SEL1L,LINC00911,SPATA7,PTPN21,CEP128,ADCK1,FLRT2,STON2,SNW1,NRXN3,LOC283585,TSHR,GALC,LINC01146,GPR65,C14orf178,SNORA79,LOC101928791	1	loss
222	CGTE_03	16	97450	3255280	16p13.3	SNORA64,CRAMP1,ERVK13-1,MRPS34,MPG,FLYWCH1,NME3,LOC106660606,EME2,TRAF7,MSLN,CLDN6,CACNA1H,WFIKKN1,CCDC78,PRSS21,NTHL1,MIR3677,MMP25-AS1,LMF1,CASP16P,AXIN1,ZNF205,SYNGR3,POLR3K,FBXL16,PDPK1,NPRL3,MIR3178,SSTR5-AS1,MSRB1,PRSS30P,MLST8,PGP,DNASE1L2,ARHGDIG,MIR3176,TPSG1,PRR25,NPW,RPUSD1,ZNF205-AS1,IFT140,CLDN9,ECI1,NHLRC4,MIR6767,BRICD5,ABCA17P,BAIAP3,PAQR4,RNPS1,RGS11,LINC00235,PKMYT1,HAGH,RAB40C,TCEB2,PRSS22,AMDHD2,HBQ1,MIR5587,MIR6511B2,TMEM8A,MIR4717,LINC00254,GFER,WDR24,MRPL28,PTX4,HAGHL,MIR3180-5,FAM195A,GNPTG,OR1F1,PRSS33,LUC7L,TPSD1,SPSB3,ABCA3,RPS2,C1QTNF8,TBL3,HBZ,C16orf91,RNF151,HS3ST6,FAM173A,HBA1,TSC2,SNHG9,ZG16B,PRSS41,RAB26,PRSS27,LOC652276,STUB1,UNKL,CCDC154,CCDC64B,METRN,MIR6511B1,NOXO1,TPSB2,FAHD1,LINC00514,MIR1225,SSTR5,SNRNP25,THOC6,HN1L,GNG13,SNORA78,TPSAB1,MIR940,PRR35,MAPK8IP3,KCTD5,MIR4516,SRRM2-AS1,MIR662,CCNF,TSR3,ATP6V0C,MIR3177,ZSCAN10,SOX8,PIGQ,ZNF213,MMP25,SRRM2,NUBP2,CLCN7,ZNF598,JMJD8,NARFL,TMEM204,SNORA10,C16orf13,FLJ42627,RHBDF1,ZNF213-AS1,CASKIN1,KREMEN2,CEMP1,IGFALS,NME4,SNHG19,C16orf59,DECR2,E4F1,PDIA2,CHTF18,MEIOB,CAPN15,NDUFB10,FAM234A,LOC100128770,RHBDL1,NTN3,TELO2,TNFRSF12A,TBC1D24,LOC101929613,SLC9A3R2,RPL3L,IL32,LOC100134368,RAB11FIP3,HBM,MIR6768,LMF1-AS1,RHOT2,PKD1,HBA2,SNORD60,UBE2I,WDR90,FLYWCH2,HCFC1R1	3	gain
223	CGTE_03	16	59785799	65839602	16q21	CDH8,CDH11,LOC101927580,APOOP5,MIR4426,LOC101927650,LINC00922,LOC729159	1	loss
224	CGTE_03	17	3346433	5998605	17p13.2	DHX33,CTNS,LOC339166,C1QBP,SPNS3,VMO1,LOC103021295,P2RX1,ZFP3,RNF167,PFN1,SLC25A11,MYBBP1A,TRPV1,MIR6865,SMTNL2,SLC52A1,LOC728392,P2RX5-TAX1BP3,MINK1,MIS12,C17orf107,CXCL16,NCBP3,LOC100130950,CHRNE,ATP2A3,GLTPD2,TM4SF5,CAMTA2,SPNS2,ZNF594,TAX1BP3,ASPA,UBE2G1,PELP1,DERL2,INCA1,SPATA22,ANKFY1,LOC102724009,NUP88,EMC6,GGT6,ALOX15,PLD2,RABEP1,RPAIN,P2RX5,CAMKK1,ZNF232,CYB5D2,NLRP1,KIF1C,SCIMP,TRPV3,ARRB2,MED11,ZZEF1,WSCD1,SPAG7,ZMYND15,PSMB6,ITGAE,GSG2,LOC101559451,ENO3,USP6,SHPK,GP1BA,MIR6864	3	gain
225	CGTE_03	17	36965919	36969124	17q12	CWC25	6	gain
226	CGTE_03	17	38786898	39538647	17q21.2	KRTAP4-2,KRT12,KRT39,KRT23,KRTAP9-1,KRTAP9-9,KRTAP9-8,KRTAP4-7,KRT33B,KRTAP2-1,KRTAP4-5,KRTAP4-1,KRTAP9-6,KRTAP29-1,KRTAP9-2,KRTAP1-4,KRTAP3-2,KRTAP4-8,SMARCE1,KRTAP1-5,KRT25,KRTAP4-12,KRTAP2-4,KRTAP9-3,KRT222,KRTAP2-3,KRT27,KRT26,KRT34,KRTAP4-4,KRTAP4-9,KRTAP1-3,KRTAP17-1,KRTAP4-11,KRTAP9-7,KRT33A,KRT20,KRT40,KRTAP4-6,KRTAP4-3,KRTAP16-1,KRTAP1-1,KRT10,TMEM99,KRT28,KRTAP9-4,KRTAP2-2,KRT24,KRTAP3-3,KRTAP3-1	1	loss
227	CGTE_03	17	80574482	81006711	17q25.3	FN3KRP,WDR45B,MIR4525,FN3K,B3GNTL1,RAB40B,TBCD,ZNF750	3	gain

228	CGTE_03	18	22669287	32621646	18q12.1-q11.2	DSG2-AS1,CDH2,MEP1B,MIR8057,MIR302F,DSC1,CHST9,DSC2,TRAPPC8,PSMA8,LINC01543,ASXL3,SS18,AQP4,AQP4-AS1,NOL4,MAPRE2,RNF125,WBP11P1,B4GALT6,KLHL14,TTR,DSG1,DSCAS,DSC3,DTNA,KCTD1,TAF4B,GAREM1,SLC25A52,ZNF521,RNF138,DSG4,DSG1-AS1,DSG3,PCAT18,CCDC178,DSG2	1	loss
229	CGTE_03	18	48512969	55218104	18q21.2-q21.31	LINC01416,LOC100287225,RAB27B,LOC102724651,SMAD4,SNORA37,WDR7,LINC-ROR,DYNAP,ST8SIA3,TCF4,MIR4529,DCC,ONECUT2,C18orf54,POLI,LINC01539,BOD1L2,TXNL1,STARD6,MEX3C,LOC101927229,MIR4528,CCDC68,TCF4-AS1,MBD2,ELAC1,LOC101928167,FECH	1	loss
230	CGTE_03	18	61765070	71442177	18q22.2-q22.1	MIR5011,CBLN2,GTSCR1,LOC101060542,TMX3,LOC643542,LOC101927481,CDH19,CDH7,LOC284294,SOCS6,LINC01541,DOK6,LOC100505817,RTTN,NETO1,LINC00305,LINC01538,CCDC102B,DSEL,LOC400655,CD226,LOC102724913	1	loss
231	CGTE_03	19	18555986	18643252	19p13.11	ELL,FKBP8	3	gain
232	CGTE_03	20	2955810	3765655	20p13	GNRH2,UBOX5-AS1,C20orf194,FASTKD5,SIGLEC1,SPEF1,SLC4A11,GFRA4,AVP,ITPA,CENPB,DDRGK1,UBOX5,ATRN,LZTS3,HSPA12B,PTPRA,MRPS26,ADAM33,C20orf27,OXT	3	gain
233	CGTE_03	20	23383506	25436437	20p11.21	CST5,ABHD12,NINL,CST8,APMAP,LOC101926889,GGTLC1,GINS1,VSX1,CST9,CST7,FLJ33581,ACSS1,CST13P,NAPB,PYGB,CST11,ENTPD6,LOC284798,CSTL1,CST2,SYNDIG1,CST1,CST9L,CST3,CST4	3	gain
234	CGTE_03	20	25439071	25448178	20p11.21	NINL	6	gain
235	CGTE_03	20	58442759	58545236	20q13.33	CDH26,PPP1R3D,FAM217B,SYCP2	1	loss
236	CGTE_03	20	58547053	61489026	20q13.33	HRH3,LOC100506470,MRGBP,SS18L1,LAMA5-AS1,C20orf197,OGFR-AS1,TCFL5,MIR4533,TAF4,GATA5,RBBP8NL,MIR4758,C20orf166-AS1,MIR646HG,MIR1257,MIR646,LSM14B,MTG2,MIR133A2,PSMA7,MIR548AG2,SLCO4A1,LINC00659,DPH3P1,RPS21,OGFR,MIR3195,CDH26,LAMA5,COL9A3,OSBPL2,MIR1-1HG,NTSR1,SLCO4A1-AS1,CDH4,ADRM1,CABLES2,MIR1-1,LOC729296,LOC101928048	3	gain
237	CGTE_03	20	61489045	61491728	20q13.33	TCFL5	6	gain
238	CGTE_03	20	61512625	62905019	20q13.33	GMEB2,PPDPF,BIRC7,DIDO1,CHRNA4,ARFRP1,RGS19,EEF1A2,UCKL1,YTHDF1,ARFGAP1,TNFRSF6B,SAMD10,UCKL1-AS1,MIR941-5,ZNF512B,ZBTB46,MIR941-3,PTK6,HAR1B,NKAIN4,C20orf195,NPBWR2,MIR647,RTEL1-TNFRSF6B,MIR4326,MIR124-3,LINC00176,MIR941-1,KCNQ2,LKAAEAR1,COL20A1,ZBTB46-AS1,MIR6813,STMN3,MIR3196,SOX18,GID8,SRMS,MIR941-4,HELZ2,SLC17A9,FLJ16779,RTEL1,LINC00029,ZGPAT,TPD52L2,PCMTD2,MIR941-2,LIME1,LOC100130587,SLC2A4RG,LINC01056,PRPF6,TCEA2,MYT1,LOC63930,MIR1914,BHLHE23,ABHD16B,LOC100505771,DNAJC5,OPRL1,HAR1A	3	gain

239	CGTE_03	21	10793919	32410828	21q11.2-q21.3	POTED,KRTAP15-1,KRTAP24-1,MIR8069-1,LOC388813,KRTAP20-4,CYP4F29P,KRTAP19-5,BAGE4,BTG3,ADAMTS1,LTN1,KRTAP22-1,KRTAP25-1,MIR99AHG,LOC101927973,KRTAP6-3,LINC00161,MAP3K7CL,N6AMT1,GABPA,KRTAP19-8,NCAM2,LINC00320,MIR155,GRIK1-AS2,ADAMTS5,BAGE2,KRTAP13-4,BACH1-IT2,LIPI,ANKRD20A11P,KRTAP19-6,KRTAP19-3,LOC102724188,KRTAP21-2,MRPL39,KRTAP21-3,ANKRD30BP2,CLDN8,CHODL-AS1,LOC284825,KRTAP27-1,KRTAP13-3,USP16,MIR4327,KRTAP21-1,LINC00307,APP,LINC00113,KRTAP13-1,CCT8,LINC00308,BACH1,C21orf91,LINC00515,NRIP1,KRTAP13-2,MIR125B2,CHODL,D21S2088E,KRTAP20-1,MIR4759,KRTAP22-2,LINC01425,KRTAP19-4,KRTAP20-2,LOC101927843,KRTAP6-2,ATP5J,TMPRSS15,KRTAP19-2,MIR155HG,KRTAP6-1,KRTAP20-3,TPTE,SAMSN1,BAGE,MIR99A,LINC00158,MIR5009,MIR548XHG,SAMSN1-AS1,LOC101927869,GRIK1-AS1,LINC00189,BAGE3,CYYR1,ABCC13,JAM2,KRTAP26-1,GRIK1,MIR3118-1,KRTAP8-1,HSPA13,RBM11,LINC00317,KRTAP11-1,RWDD2B,KRTAP19-1,KRTAP7-1,BAGE5,CLDN17,LINC00314,KRTAP19-7,MIR8069-2,KRTAP23-1,CXADR,MIRLET7C,USP25,LOC339622,C21orf91-OT1,LINC01549,MIR3156-3	1	loss
240	CGTE_04	1	79387273	84670220	1p31.1	MIR548AP,LOC101927587,ADGRL2,LOC101927560,LINC01361,LOC101927434,PRKACB,ADGRL4,LOC101927412,TTLL7	3	gain
241	CGTE_04	1	96461622	98511030	1p21.3	PTBP2,MIR2682,DPYD-AS2,DPYD,MIR137HG,LOC102723661,DPYD-AS1,LOC101928241	3	gain
242	CGTE_04	1	101476750	104068999	1p21.1-p21.2	DPH5,RNPC3,LINC01307,LOC102606465,LOC101928370,DNAJA1P5,LOC101928436,OLFM3,S1PR1,COL11A1	3	gain
243	CGTE_04	1	152382018	153122472	1q21.3	SPRR3,C1orf68,SPRR2C,LCE3B,LCE2B,LCE1F,SPRR2A,LCE3A,LCE5A,SPRR2G,LCE2D,SPRR2B,CRCT1,LCE1C,LCE3E,LCE1B,SPRR2D,SPRR1A,SPRR2F,LCE3D,LCE4A,SPRR1B,SPRR2E,SPRR4,LCE3C,LCE2C,LCE1A,SMCP,KPRP,CRNN,LCE2A,LCE1D,IVL,LCE6A,LCE1E	3	gain
244	CGTE_04	1	185833615	186313715	1q31.1	MIR548F1,HMCN1,TPR,PRG4	3	gain
245	CGTE_04	1	214832129	216896792	1q41	CENPF,ESRRG,USH2A,LOC102723833,KCNK2,KCTD3	3	gain
246	CGTE_04	2	11359051	11376110	2p25.1	ROCK2	3	gain
247	CGTE_04	2	137814043	148672920	2q22.3-q22.1	TEX41,ACVR2A,LINC01412,ARHGAP15,HNMT,ZEB2,PABPC1P2,LRP1B,KYNU,GTDC1,ZEB2-AS1,MIR7157,LOC101928273,THSD7B,LOC101928386,SPOPL,YY1P2,NXPH2	3	gain
248	CGTE_04	3	77147119	89528654	3p12.2-p11.1	HTR1F,VGLL3,LOC101927374,CADM2,CGGBP1,ROBO1,LINC00506,SNORA95,ROBO2,MIR5688,MIR4795,ZNF654,C3orf38,POU1F1,GBE1,EPHA3,CHMP2B,LINC00971,CADM2-AS2,LOC728290,MIR3923	3	gain
249	CGTE_04	3	164709155	164906468	3q26.1	SI,SLITRK3	3	gain
250	CGTE_04	4	26741477	36166702	4p14-p15.1	LOC102723828,LOC101929199,MIR4275,ARAP2,STIM2,PCDH7,TBC1D19,LOC102723778,LOC101928622	3	gain
251	CGTE_04	4	62362908	67142606	4q13.1-q13.2	TECRL,MIR1269A,EPHA5,ADGRL3-AS1,ADGRL3,LOC401134,EPHA5-AS1	3	gain
252	CGTE_04	4	85654508	87703499	4q21.23-q21.3	MIR4452,WDFY3,WDFY3-AS2,LOC101929064,PTPN13,MIR4451,ARHGAP24,MAPK10	3	gain
253	CGTE_04	5	17275551	31323431	5p13.3-p14.3	CDH10,C5orf17,BASP1,LOC340107,CDH9,LINC01021,CDH6,LOC101929660,LOC101929681,CDH18,LSP1P3,SNORA105A,LOC401177,PRDM9,CDH12,LOC101929645,GUSBP1,SNORA105B,PMCHL1	3	gain
254	CGTE_04	5	64492809	64747488	5q12.3	ADAMTS6	3	gain
255	CGTE_04	6	22290359	22294768	6p22.3	PRL	3	gain
256	CGTE_04	6	46802354	46806792	6p12.3	MEP1A	5	gain

257	CGTE_04	6	53785258	56380579	6p12.1	LRRC1,GFRAL,HMGCLL1,COL21A1,BMP5,FAM83B,MLIP-IT1,DST,MLIP,HCRTR2,LOC101927189,TINAG	3	gain
258	CGTE_04	6	72943439	73952276	6q13	KCNQ5-IT1,RIMS1,MIR4282,KHDC1,KCNQ5,KHDC1L,KCNQ5-AS1	3	gain
259	CGTE_04	6	93953021	94124538	6q16.1	EPHA7	3	gain
260	CGTE_04	6	102069804	102483492	6q16.3	GRIK2	3	gain
261	CGTE_04	6	127834029	129687662	6q22.33	LOC101928140,PTPRK,C6orf58,LAMA2,THEMIS,SOGA3	3	gain
262	CGTE_04	6	146239211	147885677	6q24.3	GRM1,STXBP5-AS1,ADGB,LUADT1,LOC101928661,STXBP5,SHPRH,SAMD5,KATNBL1P6,RAB32	3	gain
263	CGTE_04	7	77762128	86493634	7q21.11-q21.12	MAGI2,SEMA3A,MAGI2-AS3,MIR548M,GNAI1,LOC101927378,CD36,PCLO,GNAT3,SEMA3D,LOC101927269,CACNA2D1,LOC101927356,GRM3,LOC100128317,SEMA3C,SEMA3E,HGF,RPL13AP17,MAGI2-AS2	3	gain
264	CGTE_04	7	113558263	115624648	7q31.2-q31.1	TFEC,MDFIC,FOXP2,LINC01393,LINC01392,PPP1R3A,MIR3666	3	gain
265	CGTE_04	8	107696465	117658993	8q23.1-q23.3	LINC01608,NUDCD1,OXR1,TRPS1,TMEM74,TRHR,LINC00536,EMC2,SYBU,MIR2053,EIF3H,KCNV1,PKHD1L1,ANGPT1,CSMD3,ENY2,RSPO2,ABRA,EBAG9,LINC01609,EIF3E	3	gain
266	CGTE_04	9	8460324	15175117	9p22.3-p24.1	NFIB,PTPRD-AS2,ZDHHC21,MPDZ,SNORD137,LINC00583,TYRP1,FREM1,PTPRD-AS1,LURAP1L,TTC39B,PTPRD,LURAP1L-AS1,LOC389705,FLJ41200,CER1	3	gain
267	CGTE_04	9	139408916	140361965	9q34.3	MIR6722,GRIN1,PTGDS,SNORA17A,LCNL1,NOXA1,NOTCH1,PHPT1,LRRC26,NRARP,TMEM203,CCDC183-AS1,EGFL7,NPDC1,MIR4292,CLIC3,DPP7,FAM166A,SSNA1,LCN10,NALT1,SLC34A3,MIR4674,TRAF2,C9orf173-AS1,NDOR1,MIR7114,RABL6,CYSRT1,C8G,TMEM210,LCN6,LCN8,FBXW5,UAP1L1,MAMDC4,EXD3,C9orf142,TPRN,FAM69B,FUT7,NSMF,RNF224,MIR126,TOR4A,ABCA2,MIR4673,MAN1B1,MAN1B1-AS1,C9orf173,C9orf139,TMEM141,LCN12,ENTPD8,CCDC183,EDF1,SAPCD2,SNORA17B,PNPLA7,NELFB,TUBB4B,C9orf172,ENTPD2,MIR3621,RNF208,MIR4479,ANAPC2,LCN15,AGPAT2,SNHG7,LOC100128593	1	loss
268	CGTE_04	10	52066900	69571375	10q21.3-q11.23	LRRTM3,RTKN2,EGR2,MIR3924,FAM13C,ADO,LINC01553,DKK1,REEP3,MIR1296,PRKG1-AS1,LOC283045,CCDC6,IPMK,PCDH15,MIR7151,SGMS1-AS1,LINC00845,SGMS1,ARID5B,TMEM26-AS1,C10orf107,LINC01515,TMEM26,JMJD1C,CSTF2T,CTNNA3,PHYHIPL,CDK1,LOC101928961,DNAJC12,LINC00844,PRKG1,TFAM,ANXA2P3,UBE2D1,CISD1,MRLN,BICC1,JMJD1C-AS1,ZNF365,RHOBTB1,SLC16A9,MTRNR2L5,FAM133CP,A1CF,MBL2,MIR548AV,ASAH2B,ANK3,NRBF2,CCEPR,LINC01468,ZWINT,LOC102724719,MIR605	3	gain
269	CGTE_04	10	89503172	90674426	10q23.31	LIPN,ATAD1,STAMBPL1,PAPSS2,LIPM,CFL1P1,LIPK,RNLS,PTEN,ANKRD22,LIPF,KLLN,LIPJ	3	gain
270	CGTE_04	11	4624250	6413356	11p15.4	OR52E6,OR52N2,OR52W1,OR52E8,OR52E2,OLFM5P,OR51D1,OR52L1,OR56A5,OR51F2,UBQLN3,HBG1,OR51I2,OR56A3,HBB,OR52A1,OR51M1,OR52N1,OR52J3,OR52E4,TRIM6-TRIM34,C11orf42,HBBP1,OR51E2,UBQLNL,CCKBR,SMPD1,OR56B4,OR51I1,TRIM5,OR52H1,OR52D1,HBD,MMP26,OR51A7,CNGA4,OR52N5,TRIM68,TRIM6,OR51L1,OR51A4,OR51B6,OR51E1,OR51B2,OR52A5,OR56B1,OR51G2,FAM160A2,OR56A4,OR51B5,OR51A2,OR56A1,OR51F1,OR51G1,OR52B6,TRIM22,PRKCDBP,OR52R1,TRIM34,OR51T1,OR51V1,OR51B4,OR51Q1,BGLT3,OR51S1,OR52B2,HBG2,HBE1,OR52N4	3	gain
271	CGTE_04	11	15096528	16760572	11p15.1-p15.2	INSC,MIR6073,CALCB,C11orf58,LOC102724957,SOX6	3	gain

272	CGTE_04	11	20949811	31812602	11p15.1-p13	KIF18A,BDNF-AS,CCDC179,DCDC5,BBOX1-AS1,MIR610,GAS2,LGR4,SLC5A12,DCDC1,MIR8054,CCDC34,MPPED2,FANCF,LOC105376671,METTL15,KCNA4,MIR8087,FSHB,FIBIN,ELP4,LINC01495,PAX6,BDNF,SVIP,DNAJC24,LINC00678,ARL14EP,MUC15,SLC17A6,ANO3,ANO5,LUZP2,LOC105376599,BBOX1,NELL1,LIN7C,IMMP1L,MIR8068,LINC01616	3	gain
273	CGTE_04	11	33080474	43852702	11p13-p12	CD44,MIR1343,NAT10,PAMR1,TRAF6,EHF,MIR129-2,PRR5L,ELF5,LOC100507205,LRRC4C,RAG2,LINC01493,LMO2,COMMD9,CAT,FBXO3,LINC00294,HIPK3,LOC103312105,PDHX,LOC100507144,C11orf74,CAPRIN1,MIR670HG,MIR670,FBXO3-AS1,SLC1A2,TTC17,CSTF3,APIP,CSTF3-AS1,LINC01499,TRIM44,KIAA1549L,CD59,TCP11L1,RAG1,FJX1,ABTB2,API5,LDLRAD3,MIR3973,C11orf91,HNRNPKP3,HSD17B12	3	gain
274	CGTE_04	11	48238466	57004521	11p11.12-p11.11	OR9G4,TRIM48,OR5D18,OR5D16,OR4C16,OR8U1,OR4A16,LOC441601,OR9G1,OR8H3,OR4B1,OR9G9,OR5T2,MIR6128,OR5L1,TRIM51HP,OR8K1,OR4C15,FOLH1,OR10AG1,OR5M9,OR5AS1,OR5AK2,OR5L2,OR4C11,OR4A15,OR4C45,OR4C3,OR4X2,OR4P4,OR5T3,OR5T1,OR8I2,OR8J1,OR4X1,OR5J2,OR5M10,OR5M3,OR5D13,TRIM64C,APLNR,OR8K5,OR4A5,OR5M8,TRIM49B,OR5M11,LOC646813,LOC440040,LOC101927120,OR4C12,OR5AK4P,OR4S2,OR8H1,OR4C6,OR4S1,OR5W2,OR8H2,OR5I1,OR5AP2,OR5F1,TRIM51,OR4C13,OR5M1,OR5R1,OR7E5P,LRRC55,OR5AR1,OR8J3,OR4A47,OR5D14,OR8K3,OR4C46,OR8U8	3	gain
275	CGTE_04	11	57558981	60271418	11q12.1-q12.2	FAM111B,OR1S1,TMX2-CTNND1,LPXN,LOC101927204,OR9Q2,MS4A5,OSBP,STX3,OR10V1,GLYAT,GIF,OR4D10,MS4A12,MS4A3,OR4D9,TCN1,OOSP2,OR5B21,DTX4,OR5B12,GLYATL2,OR10Q1,OR5AN1,MIR6503,OR5B2,MS4A2,FAM111A,CTNND1,ZFP91,MS4A6E,ZFP91-CNTF,OR10W1,OR5A2,MS4A1,MS4A6A,MS4A4A,OR5B3,MPEG1,CNTF,OR9Q1,OR4D6,PATL1,MRPL16,GLYATL1,OR10V2P,MIR3162,LOC283194,OR6Q1,OOSP1,OR5B17,OR4D11,MS4A7,OR5A1,OR1S2,MS4A14,OR9I1	3	gain
276	CGTE_04	11	62287062	62300063	11q12.3	AHNAK	1	loss
277	CGTE_04	11	85374804	85397164	11q14.1	CCDC89,CREBZF	5	gain
278	CGTE_04	11	85406167	107313782	11q14.2-q21	ME3,MMP20,IZUMO1R,MIR7641-1,JRKL,SNORD6,MTMR2,SNORA40,SNORA18,UBTFL1,KDM4E,RAB38,AMOTL1,PGR,MMP1,MMP3,YAP1,DISC1FP1,CNTN5,CASP1,AASDHPPT,LOC100128386,SNORA32,MMP8,GRM5-AS1,WTAPP1,MIR548L,CWC15,FAM76B,PICALM,SLC36A4,TRIM53AP,CASP12,KBTBD3,LOC101928535,SNORD5,MIR1261,CTSC,MTNR1B,CASP5,KDM4D,TRPC6,CASP4,TRIM49C,TAF1D,NOX4,LOC643733,LOC100506368,OR7E2P,GRIA4,MIR3166,ANKRD49,TRIM64B,CARD16,LOC100129203,CEP295,MIR4490,LOC101929295,EED,MRE11A,DDI1,MIR1304,C11orf97,C11orf70,MMP27,PANX1,BIRC2,BIRC3,GUCY1A2,ARHGAP42,MED17,SESN3,LOC101054525,ENDOD1,CASP1P2,JRKL-AS1,CCDC81,CCDC83,CCDC82,GPR83,TYR,TRIM64,FOLH1B,VSTM5,MSANTD4,SNORA1,TRIM49D2,MMP10,TMEM135,FUT4,TMEM133,CARD18,PRSS23,TMEM123,CEP126,TRIM77,MIR1260B,SCARNA9,CCDC67,SRSF8,CHORDC1,CWF19L2,MMP13,MMP12,MIR6755,HEPHL1,MMP7,C11orf73,DCUN1D5,CARD17,SNORA25,FAT3,NAALAD2,SNORA8,MAML2,C11orf54,MIR4693,TRIM49D1,PIWIL4,FZD4,CEP57,DYNC2H1,MIR3920,ANGPTL5,GRM5,LOC102723895,TRIM49,SMCO4,PDGFD,SYTL2	3	gain
279	CGTE_04	11	123599711	124487440	11q24.1-q24.2	OR8B2,OR8D1,OR8B3,OR10G7,OR8D4,OR10G8,OR8B12,OR8G2,OR10G4,OR6M1,TMEM225,OR6T1,OR8B8,OR8A1,OR8D2,OR4D5,VWA5A,OR8B4,OR8G1,OR6X1,PANX3,OR8G5,OR10G9,OR10S1,ZNF202	3	gain

280	CGTE_04	12	7361076	7654076	12p13.31	CD163,ACSM4,CD163L1,PEX5	3	gain
281	CGTE_04	12	22625119	24736702	12p12.1	LOC101928441,LINC00477,C2CD5,LOC101928471,SOX5,ETNK1,MIR920	3	gain
282	CGTE_04	12	33021885	40869108	12p11.1-p11.21	CPNE8,LRRK2,SYT10,KIF21A,ALG10B,C12orf40,PKP2,ALG10,ABCD2,SLC2A13,MUC19	3	gain
283	CGTE_04	12	40869306	40892168	12q12	MUC19	1	loss
284	CGTE_04	12	72665587	93226584	12q21.31-q21.32	GLIPR1L2,METTL25,LOC101928137,KCNC2,NTS,MYF6,C12orf74,CSRP2,SYT1,KERA,ACSS3,POC1B-GALNT4,CCDC59,MIR548AL,CCER1,PTPRQ,TRHDE-AS1,OSBPL8,CLLU1OS,EEA1,TMTC3,BBS10,LIN7A,PLEKHG7,DUSP6,LINC01619,NAP1L1,GALNT4,PHLDA1,MIR617,MYF5,DCN,MKRN9P,LINC00615,LOC101928449,OTOGL,PPP1R12A,TRHDE,LRRIQ1,PAWR,C12orf29,GLIPR1,RASSF9,ATP2B1,NAV3,TMTC2,LOC100507377,MIR1252,PPFIA2,SLC6A15,KITLG,BTG1,LOC102724663,C12orf50,MIR618,MIR5692B,ALX1,TSPAN19,POC1B,LINC00936,LOC728084,KRR1,GLIPR1L1,CLLU1,ZDHHC17,CEP290,LINC01490,MIR4699,CAPS2,E2F7,EPYC,MGAT4C,LUM,ATXN7L3B	3	gain
285	CGTE_04	13	66878771	73302626	13q21.32-q22.1	BORA,LINC00348,PCDH9,PCDH9-AS2,PCDH9-AS4,LINC00550,ATXN8OS,MZT1,LINC00383,LINC00364,DACH1,PCDH9-AS3,KLHL1	3	gain
286	CGTE_04	13	79233136	95050929	13q31.2-q31.3	MIR622,MIR17HG,NDFIP2,GPC5-AS2,LINC01080,LINC01047,MIR17,GPC5-AS1,LINC00375,LINC00380,NDFIP2-AS1,LINC01049,RBM26-AS1,LOC105370306,GPC5,MIR19B1,LINC00382,MIR92A1,MIR20A,SNORA107,LINC01038,LINC00351,LINC00440,GPC6-AS2,LINC00333,MIR4500HG,SLITRK5,LINC00379,LINC00377,RNF219,LINC00353,MIR4500,LINC01040,GPC6,LINC00410,MIR19A,LINC00559,LINC01068,LINC00331,RBM26,LINC00397,SLITRK6,LINC00363,MIR18A,SPRY2,LINC00433,SLITRK1,LINC00430,LINC00564,GPC6-AS1	3	gain
287	CGTE_04	14	20201784	20760312	14q11.2	OR4K15,OR11H4,OR4M1,OR4K2,OR4K1,OR4Q3,OR4K5,OR4N5,OR11H6,OR4K17,OR4K14,TTC5,OR11G2,OR4L1,OR4K13,OR4N2	3	gain
288	CGTE_04	14	47120321	47812399	14q21.3-q21.2	MDGA2,RPL10L	3	gain
289	CGTE_04	14	79081309	81670412	14q24.3-q31.1	DIO2,GTF2A1,TSHR,DIO2-AS1,CEP128,SNORA79,NRXN3	3	gain
290	CGTE_04	14	104552082	106388615	14q32.33	MIR4710,MIR8071-1,TEX22,ADSSL1,AHNAK2,ELK2AP,KIAA0125,SIVA1,ASPG,C14orf180,MIR4537,C14orf79,MTA1,MIR4538,KIF26A,CEP170B,BRF1,NUDT14,MIR4507,CRIP1,TMEM179,PACS2,AKT1,CRIP2,MIR6765,MIR203A,GPR132,PLD4,MIR4539,JAG2,INF2,TMEM121,C14orf80,LINC00638,MIR8071-2,LOC100507437,CDCA4,MIR203B,ZBTB42,BTBD6,LOC102723354	1	loss
291	CGTE_04	15	52513182	52606094	15q21.2	MYO5A,MIR1266,MYO5C	1	loss
292	CGTE_04	16	56484943	56485826	16q12.2	NUDT21,OGFOD1	5	gain
293	CGTE_04	16	59785799	65345479	16q21	CDH11,LOC729159,CDH8,APOOP5,LOC101927580,LINC00922,LOC101927650,MIR4426	3	gain
294	CGTE_04	17	3101036	3366070	17p13.3-p13.2	OR3A2,OR3A1,OR1A1,OR3A4P,OR1A2,OR3A3,SPATA22,OR1E2,OR1E1,OR1D4	3	gain
295	CGTE_04	17	20135400	20149352	17p11.2	SPECC1	4	gain
296	CGTE_04	17	39138402	39334187	17q21.2	KRTAP4-8,KRTAP2-3,KRTAP4-11,KRTAP4-12,KRTAP1-5,KRTAP4-2,KRTAP2-4,KRTAP4-7,KRTAP3-1,KRTAP1-1,KRTAP1-4,KRTAP4-6,KRTAP2-1,KRTAP4-3,KRT40,KRTAP3-2,KRTAP3-3,KRTAP4-4,KRTAP2-2,KRTAP4-9,KRTAP1-3,KRTAP4-5	4	gain
297	CGTE_04	17	49824824	52978503	17q21.33-q22	CA10,C17orf112,TOM1L1,KIF2B	3	gain
298	CGTE_04	17	61086885	61499208	17q23.3-q23.2	TANC2,MIR548W	3	gain
299	CGTE_04	17	65050023	65185827	17q24.2	CACNG1,HELZ	3	gain
300	CGTE_04	17	80085517	80103865	17q25.3	CCDC57	3	gain

301	CGTE_04	18	50278305	51053157	18q21.2	DCC,MIR4528	3	gain
302	CGTE_04	18	63430052	64239437	18q22.1	CDH19,CDH7	3	gain
303	CGTE_04	19	11891406	12267474	19p13.2	ZNF440,ZNF20,ZNF439,ZNF433,ZNF625,ZNF878,ZNF625-ZNF20,ZNF441,ZNF763,ZNF69,ZNF491,ZNF844,ZNF700,ZNF788	1	loss
304	CGTE_04	19	30447630	30506003	19q12	URI1	3	gain
305	CGTE_04	19	51649204	51769189	19q13.41	MIR8074,LOC101928517,SIGLECL1,SIGLEC17P,CD33,SIGLEC7	3	gain
306	CGTE_04	20	68259	390554	20p13	NRSN2,DEFB132,C20orf96,NRSN2-AS1,DEFB126,RBCK1,TRIB3,DEFB127,DEFB128,DEFB129,SOX12,DEFB125,ZCCHC3	3	gain
307	CGTE_04	21	31015079	32410703	21q22.11-q21.3	KRTAP11-1,KRTAP24-1,KRTAP25-1,KRTAP27-1,KRTAP22-2,KRTAP13-3,KRTAP19-2,KRTAP21-2,KRTAP20-1,GRIK1,KRTAP15-1,KRTAP20-2,KRTAP26-1,KRTAP19-5,KRTAP19-3,KRTAP13-4,KRTAP19-8,KRTAP19-4,LINC00307,KRTAP13-2,KRTAP20-4,KRTAP22-1,GRIK1-AS1,KRTAP20-3,CLDN17,KRTAP19-1,KRTAP13-1,KRTAP6-2,MIR4327,KRTAP8-1,KRTAP19-7,KRTAP23-1,KRTAP6-3,KRTAP21-1,KRTAP19-6,KRTAP6-1,KRTAP7-1,CLDN8,KRTAP21-3	3	gain
308	CGTE_04	22	32352559	32545606	22q12.3	YWHAH,AP1B1P1,C22orf42,SLC5A1	3	gain
309	CGTE_04	X	2986032	2994787	Xp22.33	ARSF	3	gain
310	CGTE_05	1	861266	1565994	1p36.33	MIR429,LOC102724312,VWA1,ANKRD65,ACAP3,CCNL2,TNFRSF4,TMEM88B,LINC01342,PLEKHN1,ATAD3B,MIR6808,C1orf233,HES4,TNFRSF18,MXRA8,MIR200B,SAMD11,CPSF3L,SCNN1D,NOC2L,MIR6727,B3GALT6,MIR200A,TAS1R3,RNF223,UBE2J2,PERM1,TMEM240,MIR6726,MIB2,KLHL17,PUSL1,SDF4,CPTP,SSU72,AURKAIP1,DVL1,MRPL20,ISG15,ATAD3A,ATAD3C,AGRN,LOC148413,FAM132A,C1orf159,TTLL10	0	loss

311	CGTE_05	1	55563631	145474408	1p31.3-p13.2	SRGAP2B,ACADM,PTPN22,FNDC7,LOC101926944,HIPK1-AS1,OVGP1,MIR3117,SLC44A5,CDC7,LOC100129620,FRRS1,BCAR3,AMPD1,AP4B1,GBP4,CELSR2,GNAT2,BTBD8,EFCAB7,NEGR1-IT1,SIKE1,FGGY,LOC101928241,SH3GLB1,PTGER3,LRRC7,GSTM4,SYCP1,HSD52,GBP3,ODF2L,SAMD13,SRGAP2-AS1,KCNC4-AS1,MIR3671,S1PR1,WDR77,MIR7156,DRAM2,HSD3B1,MIR548AP,TMEM56-RWDD3,CACHD1,LOC101929147,CCDC18,FAM69A,LOC101928995,TRMT13,TRIM33,LOC101928476,LOC101927560,GPR88,HIPK1,GNAI3,MIR4256,MYSM1,ASB17,ANKRD34A,SLC25A24,UOX,FOXD3,ZZZ3,PHGDH,SYT6,RPE65,DLSTP1,MGC34796,NEXN-AS1,FAM212B-AS1,SRSF11,F3,UBL4B,MIR101-1,MCOLN2,NBPF4,VCAM1,HMGCS2,FAM72B,MAB21L3,RPF1,MIR4423,DAB1-AS1,PROK1,MAN1A2,PKN2,VANGL1,SLC30A7,SRGAP2D,LOC100129046,LOC100507634,LOC101928370,MYBPHL,MIER1,DPYD-AS1,VAV3,AMY2A,LINC01057,LOC101927412,WDR78,WDR3,PIFO,SEP15,USP1,KCND3-IT1,RTCA,GCLM,FAM72C,MFSD14A,HSD3B2,AMY1A,CLCA2,HS2ST1,GADD45A,PDE4B,PKN2-AS1,DBT,C1orf168,TMIGD3,EMBP1,C1orf52,MIR6068,LINC00623,FCGR1B,RTCA-AS1,PPIAL4C,HFE2,FAM46C,PTGFR,LOC729987,WNT2B,EXTL2,GBP2,NRAS,CD2,ST6GALNAC3,LOC729930,ABCD3,IFI44L,CYMP,GSTM3,RBM15,ATP1A1,REG4,SPATA1,LINC01555,ABCA4,ZNF326,BRDT,LOC101928979,C8A,LMO4,ADAM30,DIRAS3,OLFML3,SERBP1,SNORD45A,NFIA-AS1,AP4B1-AS1,CASQ2,DLEU2L,LOC100132057,BARHL2,GBP1P1,ROR1,LOC100996251,LINC01356,LOC101927434,GIPC2,L1TD1,CHIA,FPGT-TNNI3K,CTTNBP2NL,DOCK7,LOC101928098,GEMIN8P4,LINC01307,CTH,GTF2B,MIR378G,TYW3,LRIF1,WDR63,DNASE2B,AKNAD1,LINC00466,CCDC18-AS1,CLCA1,CEPT1,DR1,GSTM2,PPIAL4A,PRPF38B,MGC27382,GSTM1,SEC22B,PTBP2,NBPF20,KIAA1324,LINC01364,FUBP1,PPIAL4G,LOC101927139,MIR7852,CD58,EPS8L3,CHIAP2,SETSIP,PDE4DIP,CLCA3P,PLPPR4,PFN1P2,SNORA66,LINC00869,SNORD45C,DEPDC1-AS1,PGCP1,MIR197,ANGPTL3,SLC16A4,SNORD21,MIR3116-2,KCND3,AMY1B,VAV3-AS1,SLC35A3,OMA1,USP33,CYB561D1,GPSM2,EPHX4,DNAJB4,ST7L,PLPPR5,TMEM167B,LINC01359,LEPR,ALX3,WARS2,RPL5,SPATA42,LINC01160,GNG12-AS1,ERICH3-AS1,LINC01360,MIR4711,LINC00622,HAO2-IT1,MCOLN3,GNG12,DNAJC6,C1orf194,LRRC39,FLJ27354,PSRC1,SNX7,UBE2U,DENND2C,CYR61,TBX15,NEXN,DAB1,LEPROT,PTGFRN,SCARNA2,RSBN1,SLC16A1,MIR760,TACSTD2,BCL10,LOC100996635,GBP6,GBP1,HOOK1,NTNG1,FAM212B,LINC01525,SPAG17,PALMD,CTBS,MIR553,ZRANB2-AS1,TGFBR3,CSF1,ADORA3,TSHB,LHX8,ZRANB2-AS2,STRIP1,FNBP1L,CLCA4,NBPF25P,GPR61,LOC101928118,LRRC8D,PRKACB,PPM1J,LOC101927844,OLFM3,LRRC40,TNNI3K,RHOC,SYPL2,ERICH3,BCL2L15,STXBP3,LPAR3,DPYD,MIR4794,LOC101929099,CHI3L2,ACTG1P4,FOXD3-AS1,KCNC4,CSDE1,AHCYL1,CD53,MIR2682,IL12RB2,KIAA1107,FPGT,AMY2B,HENMT1,KCND3-AS1,LOC101928270,LOC102606465,NBPF6,AK5,GFI1,NFIA,LINC01358,RNPC3,C1orf146,RPAP2,C8B,PIN1P1,GSTM5,ALG14,INADL,NBPF9,MOV10,NBPF8,SLC6A17,TM2D1,NGF,AMY1C,DPH5,PRKAA2,RBMXL1,MIR137HG,LOC101926964,KCNA3,ZRANB2,AK4,LOC440600,LOC	1	loss
312	CGTE_05	1	228523416	228527904	1q42.13	OBSCN	8	gain
313	CGTE_05	2	11357124	11358640	2p25.1	ROCK2	12	gain
314	CGTE_05	2	128391646	128394207	2q14.3	MYO7B	5	gain
315	CGTE_05	2	191155133	191184522	2q32.2	HIBCH	5	gain

316	CGTE_05	2	209212534	216976185	2q34-q35	RPE,MIR4776-2,LOC102725079,LINC01614,IKZF2,LOC102724849,ABCA12,LANCL1-AS1,BARD1,VWC2L-IT1,VWC2L,LOC101928103,TMEM169,LOC100130451,ATIC,PIKFYVE,UNC80,LANCL1,MAP2,MIR548F2,CPS1-IT1,MIR4776-1,XRCC5,ERBB4,CPS1,MREG,SPAG16,ACADL,FN1,LOC101928020,LINC00607,KANSL1L,MYL1,PTH2R,MIR4438,PECR	1	loss
317	CGTE_05	2	241508080	241662245	2q37.3	KIF1A,AQP12B,GPR35,CAPN10-AS1,RNPEPL1,AQP12A,CAPN10	0	loss
318	CGTE_05	2	241662762	241680895	2q37.3	KIF1A	5	gain
319	CGTE_05	2	241682247	241815455	2q37.3	AGXT,KIF1A	3	gain
320	CGTE_05	2	241816287	241870576	2q37.3	AGXT,C2orf54	0	loss
321	CGTE_05	3	47029424	47049379	3p21.31	NBEAL2	3	gain

322	CGTE_05	3	55052174	126193761	3q13.2-p14.2	EAF2,OR5H6,FRG2EP,CRYBG3,LINC00636,CCDC58,GABRR3,MUC13,MIR5688,ASB14,SENP7,LINC00870,ARHGAP31-AS1,FBXO40,DUBR,FAM19A4,GRAMD1C,FAM86JP,BTLA,SLC15A2,LOC101929579,SLC9C1,SPATA12,FLJ22763,MTHFD2P1,OR5K1,ROPN1,APPL1,GTF2E1,LINC00879,ITGB5,TFG,C3orf67-AS1,CMSS1,SNTN,FOXP1,ST3GAL6-AS1,FEZF2,STX19,MIR4447,ALCAM,LOC101929717,LINC01215,PHLDB2,MIR4795,PTPRG-AS1,LINC00635,EOGT,CD200R1L,C3orf52,TMEM39A,TUSC7,ATXN7,KCTD6,C3orf17,MIR5092,ABHD6,MIR8060,RABL3,FAM3D,C3orf49,FOXP1-AS1,LINC00960,CGGBP1,PPP4R2,ADAMTS9,MAGI1-AS1,PTPRG,SLC25A26,HACD2,SLMAP,GSK3B,LINC00901,TAGLN3,POGLUT1,ERC2,CASR,ZXDC,ABHD10,CFAP44,TBC1D23,ARF4-AS1,ALDH1L1,MITF,LNP1,MIR7110,LOC101929754,FRG2C,PVRL3-AS1,ADAMTS9-AS1,KLF15,SYNPR-AS1,LOC101927296,ROBO1,DNAH12,SLC12A8,PDCL3P4,COL8A1,ACOX2,SNORA95,PSMD6,IFT57,ARGFX,POU1F1,MIR4273,MYLK-AS1,PDZRN3-AS1,LINC00971,NFKBIZ,OR5H2,PVRL3,SHQ1,MIR5682,GPR15,CCDC80,FILIP1L,BBX,POLQ,MIR8076,FLNB,SYNPR,ADPRH,SLC35A5,ADGRG7,CD200R1,LOC101929694,B4GALT4,KBTBD8,GPR156,CCDC54,HESX1,GUCA1C,LINC01205,HSPBAP1,MINA,OR5H1,EPHA6,CCDC37-AS1,PCNP,SPICE1,ZBED2,PARP9,ATP6V1A,MIR567,UBA3,PROK2,WDR5B,TIGIT,C3orf38,ZBTB11,CADM2-AS2,CNTN3,ARHGAP31,TIMMDC1,CD200,FAM107A,MIR3923,ZNF80,MIR548A2,FAM86DP,GTPBP8,BOC,DHFRL1,MIR4272,IMPG2,SUCLG2-AS1,CACNA2D3,MIR568,ADCY5,TMEM30C,GPR27,CD80,HP09053,CD47,ZNF148,PLCXD2-AS1,PARP15,CCDC191,LOC728290,MIR6730,GAP43,PDHB,LOC101927374,LINC00882,FAM172BP,GXYLT2,LINC00506,FSTL1,LRRC58,IGSF11-AS1,KIAA1524,POPDC2,LINC00488,B4GALT4-AS1,MIR4444-2,LINC00877,CBLB,LINC01212,LINC01279,PARP14,DTX3L,ILDR1,NAA50,NIT2,MIR5002,PRICKLE2-AS1,LOC101929607,IQCB1,FAM19A1,NR1I2,ZPLD1,MIR3136,FRMD4B,OR5K4,C3orf67,MIR3921,PLA1A,FHIT,PRICKLE2-AS3,PLCXD2,ABI3BP,NSUN3,CHMP2B,EIF4E3,DENND6A,HHLA2,ZBTB20,WNT5A,OSBPL11,MYLK,PDZRN3,HEG1,ADAMTS9-AS2,PSMD6-AS2,HGD,MIR1324,ZNF654,LOC101927056,PDE12,MIR548BB,UMPS,PRICKLE2,OR5K2,QTRTD1,CADM2,ARL6,MIR548I1,CD86,ATG3,VGLL3,USF3,SIDT1,GBE1,RYBP,OR5K3,SLC41A3,MIR4444-1,CCDC14,MYH15,OR5H15,MIR548A3,ZBTB20-AS1,FAM162A,MIR1284,THOC7,CFAP100,RETNLB,LMOD3,OR5AC2,DCBLD2,OR5H14,ALG1L,SEMA5B,EBLN2,MAGI1,ARHGEF3,MIR544B,CD96,ARF4,NXPE3,DNASE1L3,ALDH1L1-AS2,SUCLG2,ARL6IP5,TMEM45A,ROPN1B,MIR198,FAM208A,CPOX,ZDHHC23,SEC22A,DRD3,CCDC66,RPL24,PROS1,TMPRSS7,DIRC2,ARL13B,LOC152225,TRAT1,IGSF11,ERC2-IT1,NDUFB4,COX17,GCSAM,IL17RD,DPPA4,PXK,STXBP5L,MYLK-AS2,HCLS1,HTR1F,ZBTB20-AS4,LSAMP,ALDH1L1-AS1,ZBTB11-AS1,LOC102723582,C3orf30,TOMM70A,TMF1,MIR3938,LINC00698,MAATS1,EPHA3,THOC7-	1	loss
323	CGTE_05	3	126193786	126207145	3q21.3	ZXDC,UROC1	5	gain

324	CGTE_05	3	129303249	182605539	3q22.1-q25.33	STAG1,TRIM59,TM4SF1-AS1,OTOL1,AADACL2,COL6A5,PXYLP1,PRKCI,ANKUB1,LINC01208,PLSCR2,MME,RBP1,LINC01213,TIPARP-AS1,SERP1,MRAS,A4GNT,SOX14,MIR548AY,MIR6827,IGSF10,AGTR1,SPSB4,ERICH6-AS1,NPHP3-ACAD11,PLSCR1,UBA5,LINC01192,WWTR1-AS1,GPR160,PLSCR5,MYNN,BCHE,ZNF639,U2SURP,SNORA58,PDCD10,LRRC34,ATP1B3,ZMAT3,ASTE1,KCNAB1-AS1,GPR171,RNF13,PPP2R3A,LOC100289361,TOPBP1,SOX2-OT,AADACL2-AS1,IL12A,CPA3,P2RY13,LINC00578,LEKR1,SHOX2,PAQR9-AS1,ATP2C1,SLC2A2,ACAD11,TM4SF4,NAALADL2-AS1,P2RY14,IQCJ-SCHIP1-AS1,LINC01330,GPR87,CCNL1,FLJ46066,KCNAB1-AS2,MECOM,RARRES1,PLCH1,ARHGEF26,PLXND1,KCCAT211,RYK,SLCO2A1,ZBBX,SUCNR1,TIPARP,GRK7,CLDN18,TMEM108,MIR4788,MIR16-2,WWTR1,MED12L,PFN2,GHSR,ACTRT3,IQCJ,PQLC2L,DBR1,SEC62,LINC00880,COL6A4P2,GK5,LRRC31,COL6A6,ESYT3,CDV3,C3orf33,TRIM42,CP,TMEM212-AS1,LOC100505609,GFM1,ALG1L2,CLRN1,LOC100507389,LOC102724604,PPM1L,AMOTL2,TTC14,SIAH2,CLDN11,CEP63,LINC00501,SCHIP1,NUDT16P1,MIR6828,MLF1,ACTL6A,LOC100507661,LOC100996447,TMEM212,B3GALNT1,NME9,CLSTN2,SLITRK3,GNB4,ARL14,SLC33A1,FAM86HP,CEP70,CCDC39,PHC3,LOC101928739,TERC,PLD1,SPATA16,MRPL3,ARHGEF26-AS1,SLC7A14,NAALADL2-AS3,MRPL47,GPR149,CHST2,NLGN1-AS1,SPTSSB,MRPS22,NAALADL2-AS2,PRR23B,NUDT16,RASA2,SMC4,SOX2,GOLIM4,RAP2B,LXN,LINC00886,BPESC1,PCCB,RNF7,LINC01210,PLSCR4,MBNL1-AS1,HLTF-AS1,EIF5A2,KCNMB2,C3orf79,FAIM,SCARNA7,XRN1,TM4SF18,RBP2,WDR49,FNDC3B,TNIK,MIR7977,NDUFB5,ZBTB38,SELT,MIR1263,SSR3,NEK11,EGFEM1P,PIK3CA,NPHP3,SLC9A9,ECT2,C3orf58,LINC01100,KCNAB1,FXR1,LINC01327,LOC339874,LINC01487,LINC01206,DNAJC19,DHX36,GYG1,COMMD2,ZIC1,LOC646903,EPHB1,PLS1,FOXL2NB,MIR3919,NMD3,AADAC,RAB6B,ARMC8,TRPC1,COPB2,IFT80,SLC9A9-AS1,KPNA4,IL20RB,SLC25A36,PISRT1,SKIL,LOC100128164,C3orf36,IQCJ-SCHIP1,LINC01391,NCK1-AS1,LOC100507537,PIK3CB,SERPINI1,TM4SF1,PTX3,KCNMB3,MIR15B,NAALADL2,RPL22L1,MIR5704,ANAPC13,CPB1,NCEH1,NPHP3-AS1,LINC01322,IL12A-AS1,LOC101928882,NLGN1,MFN1,PRR23A,SERPINI2,KCNMB2-AS1,SRPRB,PA2G4P4,PAQR9,NMNAT3,CLSTN2-AS1,ERICH6,TMCC1-AS1,ACPP,SLC35G2,ATR,AADACP1,ATP11B,LINC01324,NCK1,LOC100507291,CLRN1-AS1,P2RY1,LINC01214,GMPS,TBL1XR1,LINC01209,MIR5186,TFDP2,PLOD2,PRR23C,ZIC4,USP13,TMCC1,HPS3,TRH,RSRC1,PEX5L,P2RY12,RNU6-2,TNFSF10,PCOLCE2,LOC101243545,TMEM14EP,VEPH1,DNAJC13,CPNE4,LOC101928105,BFSP2,SAMD7,KY,PIK3R4,EIF2A,SI,HLTF,LINC00881,C3orf80,TSC22D2,TF,DZIP1L,MIR4789,MIR548H2,MIR551B,MSL2,MIR569,PEX5L-AS2,LINC01014,FOXL2,ACKR4,MBNL1,TMEM108-AS1,LOC440982,MFSD1,LRRIQ4	1	loss

325	CGTE_05	3	185538772	194219825	3q29-q27.2	DPPA2P3,CLDN1,LOC253573,ATP13A4-AS1,GMNC,LINC00887,PYDC2,EIF4A2,LOC101929337,MASP1,SST,LOC102724699,LPP-AS2,SNORA4,MIR944,IL1RAP,FGF12,IGF2BP2,LINC00884,HRG,AHSG,ADIPOQ,MB21D2,ATP13A3,CPN2,GP5,CLDN16,RPL39L,ST6GAL1,FLJ42393,RTP1,SNAR-I,UTS2B,DGKG,SNORA81,LRRC15,LPP,RTP4,LOC100131635,ETV5,CCDC50,P3H2,LPP-AS1,LOC100505920,LOC344887,ATP13A5,TRA2B,TPRG1,OPA1,LOC647323,HES1,BCL6,RFC4,OSTN,TP63,LINCR-0002,P3H2-AS1,MGC2889,TMEM207,ATP13A4,FGF12-AS1,OPA1-AS1,HRASLS,SNORD2,ADIPOQ-AS1,LOC101929106,MIR1248,TPRG1-AS1,RTP2,ATP13A5-AS1,TPRG1-AS2,SNORA63,FETUB,DNAJB11,CRYGS,MIR28,TBCCD1,OSTN-AS1,KNG1	1	loss
326	CGTE_05	4	884322	947156	4p16.3	TMEM175,GAK	4	gain
327	CGTE_05	4	948994	1306042	4p16.3	DGKQ,TMED11P,SLC26A1,RNF212,CTBP1,MAEA,LOC100130872,SPON2,CTBP1-AS2,TMEM175,IDUA,CTBP1-AS,FGFRL1	0	loss

328	CGTE_05	4	13371441	184596026	4q32.2-q21.23	APBB2,ANKRD17,PPEF2,INTU,FAM160A1,FAM47E-STBD1,CLGN,DCUN1D4,COL25A1,DKK2,SHROOM3,LINC00613,MIR4453,GUCY1A3,TACR3,SLC30A9,PP12613,LOC340017,H2AFZ,LOC101929194,TBC1D19,IGFBP7-AS1,SYNPO2,FGA,RASGEF1B,GYPE,LOC728040,SLC10A4,NAF1,ARAP2,RFC1,LOC102723828,AMTN,CENPE,LAP3,LIMCH1,STAP1,LINC01088,SH3RF1,TRAM1L1,LINC01365,LINC01207,CORIN,MIR3140,SLC39A8,PLRG1,PDGFC,PI4K2B,MAD2L1,GPAT3,LINC00989,STBD1,LOC101928851,UGT2A1,C1QTNF7,LOC101928978,RNF175,CEP135,DAPP1,C4orf51,CLOCK,LOC101927305,CNGA1,PRDM8,CXCL11,BMP2K,CAMK2D,LINC01612,MIR8082,SMR3A,PCDH7,DTHD1,ATP8A1,EREG,RHOH,PPID,IGFBP7,KDR,LOC101929577,CENPC,BMPR1B,SULT1E1,LINC00616,FAM13A-AS1,LIN54,ZNF330,SEPSECS-AS1,WDR17,NAAA,LOC101927282,PROM1,TMPRSS11A,RAB33B,EDNRA,WDR19,LINC01085,SLC9B1,FDCSP,NAA11,EMCN,LOC101929448,SMAD1,HSPA4L,TMEM33,MSMO1,LOC102723778,PPAT,ANKRD50,IL2,CYP2U1,MIR548AJ2,IL21-AS1,FBXL5,LOC441025,ADGRL3,ATP10D,STPG2-AS1,LOC105377348,CISD2,MND1,BBS7,GRXCR1,MIR4449,SEPT11,SPCS3,GPRIN3,RPL9,POU4F2,NPY2R,GSTCD,HADH,TNRC18P1,PITX2,JADE1,BTC,CC2D2A,KLHL2,ENOPH1,PF4,CWH43,POLR2B,SMAD1-AS1,GRID2,GC,CDKN2AIP,LINC00499,FAM200B,SMARCAD1,SPINK2,FGF5,AADAT,MIR4454,TBC1D1,TMA16,SEC31A,DCHS2,PLAC8,CXXC4,NR3C2,CSN1S2AP,ADH4,LRBA,LOC100506122,MFSD8,LARP7,PDE5A,RAPGEF2,GSX2,QDPR,SCRG1,SCOC,SNORA24,SCARB2,ANK2,LOC101928551,LOC101928131,SHISA3,FGB,ELF2,ZGRF1,LOC101929741,LOC100996694,DSPP,HTN1,MIR5091,MGAT4D,LINC00682,CXCL1,TMPRSS11GP,MGARP,ETNPPL,DANCR,RAB28,HELQ,MIR548AX,KLB,MIR5684,NSUN7,MIR4801,TMPRSS11D,MGST2,GNRHR,RASSF6,SEC24B-AS1,FAM13A,ZAR1,PDHA2,CCKAR,UGDH,BST1,MIR548AH,DCLK2,LAMTOR3,FRYL,NPFFR2,SCFD2,MIR1305,PRMT9,MIR1255A,EIF4E,FHDC1,IBSP,MAB21L2,JCHAIN,UGT2B10,INTS12,METAP1,ARL9,DKFZP434I0714,ANAPC4,LINC00290,SLAIN2,LGI2,COQ2,CLRN2,SH3D19,GPM6A,PRR27,ART3,FREM3,DMP1,DNAJB14,RPL34-AS1,CHRNA9,SLC7A11-AS1,LOC101927157,MIR3688-2,MIR3684,MIR3688-1,BMP3,CCNI,RWDD4,ANP32C,HTN3,AASDH,ENPEP,TLR1,MIR578,MIR7641-2,GUCY1B3,GUSBP5,UCHL1,CPEB2-AS1,DCAF16,SMIM20,ENAM,TRAPPC11,N4BP2,THAP9-AS1,CDKL2,USP46,BDH2,LOC100507388,EPGN,SRD5A3,ANXA5,PTPN13,STATH,PLA2G12A,PAICS,PF4V1,CHIC2,LOC101927636,TRPC3,SLC9B2,MRPS18C,NUDT6,SLC25A31,ANXA3,MIR574,NFXL1,TET2,TLR10,PRSS12,MEPE,TRIM60,MIR5096,GABRG1,PCNAP1,TKTL2,FAM47E,TMEM144,LINC01094,TDO2,EPHA5,KCTD8,LNX1-AS2,RRH,RXFP1,KLF3,CPEB2,TMPRSS11E,NPNT,CBR4,MIR3139,TMPRSS11B,BEND4,BBS12,COMMD8,MIR4450,NPY5R,HSD17B13,CDS1,CXCL6,PHOX2B,PCDH10,SAP30,TMEM154,OSTC,AFP,FAM114A1,MIR7849,SLC4A4,GLRA3,C4orf3,LOC101927087,LOC101928590,CNOT6L,TXK,LINC01618,ANXA2P1,NKX3-2,FSTL5,LINC01099,ARHGAP10,TMEM150C,RBM47,FTLP10,LRAT,CD38,ARSJ,SULT1B1,LOC550113,SNORD143,CCSER1,MIR7978,KCNIP4,DHX15,KLHL8,UGT2B4,ATOH1,BANK1,ADH5,LINC01179,UTP3,C4orf27,ERVMER34-	1	loss
329	CGTE_05	5	31486493	35089878	5p13.3-p13.2	MIR579,TTC23L,AGXT2,SUB1,BRIX1,DNAJC21,RXFP3,AMACR,PRLR,PDZD2,C5orf22,MIR7641-2,NPR3,MIR4279,LOC340113,MTMR12,RAD1,ADAMTS12,SLC45A2,RAI14,TARS,C1QTNF3,C1QTNF3-AMACR,GOLPH3,ZFR,DROSHA	3	gain
330	CGTE_05	5	37165544	38932702	5p13.1-p13.2	LIFR-AS1,MIR3650,GDNF-AS1,C5orf42,LIFR,EGFLAM-AS2,WDR70,NUP155,LINC01265,EGFLAM,OSMR,EGFLAM-AS4,GDNF,OSMR-AS1	3	gain
331	CGTE_05	5	42713458	42759330	5p12	GHR,CCDC152	7	gain

332	CGTE_05	5	140167086	140348609	5q31.3	PCDHA9,PCDHA1,PCDHA5,PCDHA4,PCDHA8,PCDHAC2,PCDHA7,PCDHAC1,PCDHA6,PCDHA13,PCDHA11,PCDHA10,PCDHA12,PCDHA3,PCDHA2	1	loss
333	CGTE_05	5	175993936	175996071	5q35.2	CDHR2	7	gain
334	CGTE_05	6	33131385	33143435	6p21.32	COL11A2	3	gain
335	CGTE_05	6	57472304	74104708	6q13-q11.2	SDHAF4,LMBRD1,COL19A1,LOC102723883,KHDC1,B3GAT2,DDX43,PRIM2,FAM135A,PHF3,LGSN,LINC00472,ADGRB3,KHDC1L,KHDC3L,LINC00680-GUSBP4,MIR4282,LOC101928307,SLC25A51P1,MIR30A,RIMS1,KHDRBS2,LINC00680,LOC441155,KCNQ5-AS1,SMAP1,LINC01626,EYS,EVADR,MTRNR2L9,KCNQ5,OOEP,COL9A1,GUSBP4,KCNQ5-IT1,DPPA5,OGFRL1,LOC101928280,MIR30C2,PTP4A1	1	loss
336	CGTE_05	7	75912736	91646498	7q21.13-q21.11	SEMA3E,HGF,DPY19L2P4,RPL13AP17,DMTF1,CFAP69,MAGI2,LOC101409256,GTPBP10,ABCB1,CACNA2D1,GNAT3,LOC101927269,AKAP9,LOC101927446,ZNF804B,POMZP3,TMEM243,KIAA1324L,FZD1,LOC100128317,UPK3B,CLDN12,ZP3,STEAP4,STEAP2,SRI,PHTF2,SEMA3C,STEAP1,TP53TG1,GNAI1,SEMA3A,MTERF1,YWHAG,ABCB4,CD36,MAGI2-AS3,LOC102723885,PMS2P9,APTR,SSC4D,DTX2P1-UPK3BP1-PMS2P11,SRRM3,LOC101927243,CDK14,CCDC146,SEMA3D,FGL2,DBF4,ADAM22,CROT,MIR548M,DTX2,PCLO,LOC100133091,TMEM60,PTPN12,GSAP,HSPB1,MAGI2-AS2,LOC101927356,RSBN1L,FDPSP2,C7orf62,RUNDC3B,LOC101927378,SLC25A40,GRM3,STEAP2-AS1	1	loss
337	CGTE_05	7	111936170	127011941	7q31.33-q31.31	POT1,C7orf60,TMEM168,RNF148,ASZ1,LOC101928012,LOC102724555,WNT2,LOC101928333,LOC101928283,FOXP2,LOC101928254,HYALP1,HYAL4,LSMEM1,POT1-AS1,MDFIC,NDUFA5,SLC13A1,CAV2,IQUB,GPR85,LMOD2,CTTNBP2,IFRD1,LINC01392,LSM8,LOC102724434,RNF133,LINC01393,CAV1,LOC101928211,HRAT17,MIR6132,TES,PTPRZ1,KCND2,WASL,LVCAT5,FEZF1-AS1,ST7-AS2,RNU6-2,ZNF277,C7orf77,AASS,PPP1R3A,CADPS2,FAM3C,CAPZA2,ASB15,ST7-AS1,TAS2R16,ST7,LINC00998,ST7-OT3,MIR3666,ANKRD7,CFTR,GPR37,ST7-OT4,WNT16,LINC01510,GRM8,ING3,MIR592,TMEM229A,TSPAN12,MET,LOC100996249,ZNF800,TFEC,FEZF1,SPAM1,CPED1	1	loss
338	CGTE_05	8	144643895	146279543	8q24.3	LRRC14,TIGD5,ZNF623,ZNF252P-AS1,C8orf33,FAM83H-AS1,ZNF252P,RPL8,PPP1R16A,PARP10,TMED10P1,GPAA1,MROH6,CPSF1,SLC39A4,TONSL-AS1,ZNF707,ZNF250,HGH1,MIR937,GRINA,LRRC24,MIR6850,FOXH1,MIR939,VPS28,MIR6849,ZNF16,GSDMD,ADCK5,CYHR1,PUF60,SCX,EXOSC4,MFSD3,SLC52A2,MIR6846,SCRIB,HSF1,MIR4664,NAPRT,ZNF517,CYC1,MIR7112,DGAT1,ZNF7,BOP1,RECQL4,SHARPIN,MIR661,FBXL6,WDR97,MAPK15,PYCRL,MIR6848,EPPK1,CCDC166,TMEM249,SCRT1,BREA2,NRBP2,ZNF34,MROH1,MIR6845,MAF1,SPATC1,EEF1D,C8orf82,KIFC2,COMMD5,FAM83H,OPLAH,TSTA3,GPT,MIR6893,PLEC,ZNF251,MIR6847,TONSL,ARHGAP39	0	loss
339	CGTE_05	9	139378808	140435261	9q34.3	LCN12,DPP7,TUBB4B,MIR6722,MAN1B1,FUT7,CLIC3,FBXW5,NRARP,MIR7114,GRIN1,LCN8,MIR4292,NPDC1,SAPCD2,CCDC183-AS1,EDF1,C9orf163,C9orf142,LOC100128593,LRRC26,TMEM210,RNF224,MAN1B1-AS1,TMEM203,NSMF,EXD3,RNF208,SNORA17B,C9orf173-AS1,SNORA17A,FAM69B,TOR4A,MIR4479,PNPLA7,ENTPD2,MIR3621,NOTCH1,NOXA1,NDOR1,LCNL1,ENTPD8,TRAF2,NALT1,PTGDS,LCN15,NELFB,LCN6,MAMDC4,SSNA1,SLC34A3,FAM166A,MIR126,UAP1L1,PHPT1,ANAPC2,C8G,SNHG7,C9orf139,CYSRT1,LCN10,TPRN,CCDC183,EGFL7,RABL6,C9orf172,ABCA2,MIR4674,TMEM141,C9orf173,MIR4673,AGPAT2	0	loss

340	CGTE_05	10	64135880	64979813	10q21.2-q21.3	NRBF2,ZNF365,JMJD1C,EGR2,ADO	4	gain
341	CGTE_05	10	122263013	123353405	10q26.12-q26.13	FGFR2,LINC01561,PLPP4,WDR11,MIR5694,WDR11-AS1	20	gain
342	CGTE_05	10	123503239	124610035	10q26.13	TACC2,DMBT1,NSMCE4A,ATE1,PLEKHA1,BTBD16,ATE1-AS1,CUZD1,C10orf120,FAM24B,MIR3941,DMBT1P1,ARMS2,FAM24B-CUZD1,HTRA1	3	gain
343	CGTE_05	10	126355878	129242426	10q26.2-q26.13	TEX36,C10orf90,MMP21,FAM53B,BCCIP,DOCK1,METTL10,FAM196A,CTBP2,MIR4484,ADAM12,LINC00601,ZRANB1,FLJ37035,FANK1-AS1,FAM53B-AS1,DHX32,MIR4296,EDRF1,EDRF1-AS1,FAM175B,UROS,TEX36-AS1,FANK1,LOC283038	4	gain
344	CGTE_05	11	193051	34074197	11p13-p15.5	CRACR2B,BTBD10,CD151,FIBIN,LMO1,LOC644656,OR52E2,OR52K1,LINC01495,IFITM2,TRIM6-TRIM34,IGSF22,FAM160A2,RPS13,MUC5B,KCNQ1DN,OR52J3,C11orf58,WT1-AS,OR56A3,SBF2,KRTAP5-2,OTOG,ARNTL,FAM99A,LYVE1,MUC5AC,LINC01219,PDE3B,C11orf40,OR51A2,BDNF,MRGPRE,OR52N5,SLC6A5,HBE1,OR51V1,CDKN1C,KIF18A,MMP26,OR52N2,OR5P3,LOC105376599,MRGPRX1,LOC100126784,SCGB1C2,OR52N4,TAF10,MIR5691,CAPRIN1,PNPLA2,MUC2,DCDC1,RNH1,PKP3,OR10A5,AKIP1,GVINP1,WT1,CSTF3-AS1,LOC101928053,OR2AG1,LMNTD2,OR52E6,KIAA1549L,OR51E2,IPO7,SMPD1,IFITM3,LOC494141,MIR210,METTL15,HTATIP2,PIDD1,LOC100506675,PRMT3,OR51F2,ELP4,KRTAP5-4,LOC102724784,SAA2-SAA4,SOX6,OR52N1,CTSD,LOC283299,SYT9,FBXO3,ZNF143,CCDC73,B4GALNT4,CSNK2A3,OR52E4,ARFIP2,RASSF10,ZNF215,OR51I2,LRRC56,LIN7C,TRIM3,TSG101,MOB2,ZNF214,INS,LDHC,TRIM34,LOC100506258,TRPC2,SAA1,UBQLNL,MIR610,DBX1,NAV2-AS4,C11orf16,QSER1,CD59,ZNF195,TRIM66,ASCL3,CYP2R1,LMO2,LGR4,HIPK3,OR6A2,SNORA3A,OR52A5,CD81-AS1,SCGB1C1,PTDSS2,CTR9,CNGA4,SNORD131,OR52B6,NLRP6,WEE1,OR52D1,MIR4687,ART1,UEVLD,OR7E12P,KCNJ11,KCNQ1-AS1,SNORA3B,SPON1,DNAJC24,ART5,ODF3,E2F8,MIR4486,SVIP,IGF2-AS,TRIM22,RCN1,TMEM9B-AS1,SBF2-AS1,TSPAN32,SNORD97,OR51S1,LOC101927708,TSPAN4,LSP1,HBBP1,MRPL23-AS1,SNORA52,GTF2H1,MICALCL,MIR6124,SLC22A18AS,OR56B4,MIR4686,CHRNA10,C11orf91,TPP1,OR52I1,OLFML1,KCNA4,BGLT3,LOC171391,PRRG4,OR10A4,LOC100506082,NAV2,DRD4,TOLLIP-AS1,OR51Q1,CEND1,POLR2L,PAUPAR,NLRP10,HOTS,MRVI1,CCDC34,IGF2,CD81,MIR6073,KRTAP5-1,MIR6744,MIR8068,PHLDA2,SNORA88,MRGPRX2,TSSC4,SAA2,OR51B5,SCUBE2,LINC01616,MYOD1,CYB5R2,OR2D3,TUB,NAV2-AS2,OR51B4,SERGEF,LOC101928008,OR56A4,CHID1,AMPD3,LINC00678,LOC102724957,SNORA23,MIR8054,OR51B2,UBQLN3,MIR8087,HRAS,TMEM41B,USP47,OSBPL5,MIR7847,ABCC8,INS-IGF2,PTPN5,FANCF,APBB1,OR51M1,RASSF7,DCDC5,OR52I2,MICAL2,SAAL1,RNF141,HPS5,OR10A2,MIR483,IFITM5,MRVI1-AS1,RBMXL2,INSC,MIR4694,TPH1,TIMM10B,TMEM86A,KCNQ1,SPTY2D1,PLEKHA7,KRTAP5-6,PPFIBP2,DEAF1,LDHA,FSHB,NAV2-AS5,SCT,LOC143666,SAA3P,TOLLIP,TRIM6,MUC15,KRT8P41,CCKBR,OR52A1,TCP11L1,DEPDC7,DCHS1,OR52L1,OR52R1,HBD,TEAD1,CASC23,RRM1,MIR8070,HBG2,MIR4298,PSMA1,IFITM1,ARL14EP,TRIM68,STK33,C11orf42,CALCB,LOC105376671,PHRF1,ILK,ST5,IFITM10,OR51E1,KRTAP5-5,COPB1,OR51L1,ZDHHC13,MRGPRX3,RPLP2,DKK3,H19,OR51G2,PARVA,SPTY2D1-AS1,SLC17A6,CALCA,ZBED5,OR52E8,TRIM21,ANO3,MRGPRX4,FBXO3-AS1,C11orf21,PANO1,FAR1,KCNC1,NUCB2,RRP8,TH,OR2D2,OLFM5P,RRAS2,OR51I1,MIR302E,TRIM5,TALDO1,MIR6743,SWAP70,ANO5,PDDC1,CCDC179,NLRP14,MIR675,PSMD13,PIK3C2A,SIGIRR,LOC105376575,TNNT3,OR5P2,OR51F1,OR10A3,SIRT3,MUC6,OR52W1,BRSK2,	1	loss

345	CGTE_05	11	34093198	35160966	11p13	PDHX,LOC100507144,APIP,NAT10,EHF,CD44,CAPRIN1,CAT,MIR1343,ELF5,ABTB2	5	gain
346	CGTE_05	11	35161026	36692999	11p12-p13	FJX1,PAMR1,RAG2,TRAF6,SLC1A2,RAG1,C11orf74,MIR3973,COMMD9,CD44,TRIM44,PRR5L,LDLRAD3	7	gain
347	CGTE_05	11	67172841	67818329	11q13.2	DOC2GP,GPR152,NDUFV1,GSTP1,MIR6753,PITPNM1,MIR6752,NDUFS8,CARNS1,CABP4,ACY3,CORO1B,ALDH3B1,TBX10,TBC1D10C,MIR7113,C11orf72,MIR4691,NUDT8,FAM86C2P,AIP,ALDH3B2,TCIRG1,RPS6KB2,TMEM134,CABP2,UNC93B1,PTPRCAP,CDK2AP2	0	loss
348	CGTE_05	12	10131985	48091533	12p11.23-q12	DENND5B,TMEM52B,PTHLH,ETV6,PPHLN1,YARS2,FAM234B,LOC100288798,LRMP,GUCY2C,LINC01489,KLRAP1,PDE3A,PLCZ1,RERGL,CASC1,PRR4,MIR4302,SMCO2,GOLT1B,KIF21A,RACGAP1P,TAS2R30,MIR1244-4,ARNTL2-AS1,MRPS35,TMTC1,PLEKHA5,LOC101928162,PCED1B,SLC2A13,KLHL42,TAS2R14,KIAA1551,ABCD2,MIR3974,LINC00938,SLCO1A2,PRH1,LINC01559,PRB1,AEBP2,MIR614,DUSP16,CLEC12A,APOLD1,LMNTD1,MIR4698,SMIM10L1,CLEC7A,LOC100506393,YAF2,TAS2R7,ITPR2,ERP27,KLRC1,PRH1-TAS2R14,TAS2R19,KLRC3,CLEC9A,CCDC91,STYK1,CNTN1,CAPZA3,CLEC12B,PLBD1-AS1,C12orf77,RPAP3,SSPN,DNM1L,BCL2L14,C2CD5,KLRC4,ATF7IP,MIR1244-3,DENND5B-AS1,MIR613,OLR1,ASUN,KLRK1,AMN1,ETNK1,PRH1-PRR4,MIR1244-1,DDX47,TAS2R31,LOC101927058,TAS2R8,SLC15A5,SLCO1B3,TAS2R43,MIR920,BICD1,LINC00477,CAPRIN2,IAPP,FGD4,GABARAPL1,PUS7L,LRRK2,CREBL2,TAS2R20,HEBP1,LRP6,CLEC1A,PTPRO,CPNE8,LMO3,YBX3,MUC19,C12orf71,BCAT1,SLC38A1,LINC01252,PIK3C2G,MIR1244-2,ARID2,SLCO1B1,MAGOHB,PPFIBP1,TAS2R13,ERGIC2,PDZRN4,MANSC4,MIR4494,SKP1P2,PLEKHA8P1,RERG,ART4,LOH12CR2,KLRC2,GPRC5A,IPO8,LINC00941,PYROXD1,KLRD1,ABCC9,PRB4,KRAS,GPRC5D,TAS2R42,AMIGO2,PRH2,PRB3,GRIN2B,PLBD1,MIR7641-2,LDHB,SLCO1C1,RPL13AP20,STRAP,RASSF8,ZCRB1,MGST1,CLEC1B,OVCH1-AS1,EPS8,TAS2R50,TAS2R46,GYS2,SOX5,ANO6,SLC38A2,FGFR1OP2,PCED1B-AS1,PRB2,SLCO1B7,HTR7P1,SCAF11,IRAK4,BHLHE41,SLC38A4,TMEM117,RECQL,GSG1,FLJ13224,KCNJ8,FAR2,ALG10,LOC100506314,RNY5,SMCO3,TM7SF3,DERA,MED21,ARNTL2,ARHGDIB,LOC102724020,TWF1,ST8SIA1,METTL20,KLRC4-KLRK1,RERG-AS1,DDX11-AS1,LOC100506606,MIR7851,C12orf60,DBX2,WBP11,LOC101928441,SYT10,TAS2R9,H2AFJ,LYRM5,EMP1,CDKN1B,MANSC1,CMAS,HIST4H4,LOC101928471,SPX,PDE6H,LOC101928100,TAS2R10,BORCS5,C12orf40,DDX11,PRICKLE1,GPR19,MGP,ALG10B,ADAMTS20,NELL2,RASSF8-AS1,STK38L,H3F3C,OVCH1,REP15,TSPAN11,PKP2,FAM60A,GXYLT1	1	loss
349	CGTE_05	12	48370605	48380214	12q13.11	COL2A1	3	gain
350	CGTE_05	12	57572621	57596318	12q13.3	MIR1228,LRP1	3	gain
351	CGTE_05	12	72289666	72300903	12q21.1	TBC1D15	8	gain

352	CGTE_05	12	72307522	93226584	12q21.2-q21.32	EPYC,TBC1D15,KERA,MIR618,LOC101928449,MGAT4C,E2F7,LINC00615,LINC01619,NAV3,DUSP6,C12orf50,MIR4699,PAWR,PPFIA2,TMTC2,SLC6A15,PPP1R12A,CCER1,MIR617,RASSF9,BTG1,ATP2B1,MIR548AL,KCNC2,LINC01490,CSRP2,KITLG,CCDC59,SYT1,PTPRQ,LRRIQ1,EEA1,ACSS3,OSBPL8,BBS10,OTOGL,METTL25,CLLU1OS,POC1B,C12orf74,PHLDA1,PLEKHG7,LUM,TMTC3,TPH2,CAPS2,TSPAN19,NTS,GLIPR1L2,LOC100507377,LIN7A,NAP1L1,KRR1,POC1B-GALNT4,TRHDE-AS1,LINC00936,DCN,C12orf29,CLLU1,MYF5,ALX1,MIR5692B,CEP290,MIR1252,MKRN9P,GLIPR1L1,GALNT4,LOC102724663,LOC101928137,ATXN7L3B,ZDHHC17,TRHDE,MYF6,GLIPR1,LOC728084	1	loss
353	CGTE_05	12	132502709	132505835	12q24.33	EP400	5	gain

354	CGTE_05	13	32371301	95095850	13q13.3-q14.2	RFC3,NEK3,PDS5B,UCHL3,KPNA3,DIAPH3,LINC00333,MIR4500HG,ERICH6B,TPT1-AS1,LINC00371,PCDH20,KLF12,GPC6-AS1,MIR19A,LINC00448,SCEL-AS1,LINC00392,RB1,LINC01040,LOC103191607,GPALPP1,LINC00564,DIAPH3-AS2,PCDH9-AS2,INTS6,LINC00284,MIR19B1,MTRF1,LACC1,LOC105370306,GPC6,FBXL3,MIR320D1,LINC01047,DLEU1-AS1,CTAGE10P,DIAPH3-AS1,NDFIP2-AS1,TPTE2P5,LINC01075,LRCH1,CYSLTR2,LOC101929657,VWA8-AS1,MIR4305,ALG5,CDADC1,LINC00382,KBTBD6,LINC00348,TRIM13,MIR3168,MIR4703,SLITRK6,SCEL,EDNRB-AS1,LINC00353,DLEU2,TUSC8,WDFY2,TSC22D1,BTF3P11,SLITRK5,AKAP11,STARD13,PRR20D,LINC01198,NHLRC3,INTS6-AS1,FAM216B,HNRNPA1L2,ATP7B,DNAJC15,ENOX1,RFXAP,PCDH9-AS3,LINC00445,LINC01078,FREM2,PROSER1,SNORA31,SPRY2,VPS36,LINC00440,LINC00282,MIR20A,MLNR,GPC6-AS2,MIR5007,SLITRK1,PRR20C,LINC00441,SUGT1,LOC102723968,PRR20B,MED4,MIR1297,KCTD4,SPG20,CCNA1,RNASEH2B-AS1,LINC01052,LINC01038,LINC00462,SOHLH2,STOML3,LINC00347,LINC01050,LINC00380,RBM26,GTF2F2,LINC00376,VWA8,MIR17,CKAP2,SLC25A30-AS1,PCDH8,PCDH9-AS4,TPTE2P3,RGCC,LINC00550,POSTN,RBM26-AS1,TNFSF11,FNDC3A,HTR2A,LINC00434,OLFM4,MIR3665,GPC5,EEF1DP3,TSC22D1-AS1,PIBF1,COG6,CCDC122,RNF219-AS1,LINC00331,LPAR6,RNF219,LOC101926897,LINC00410,DIS3,MRPS31,KCTD12,RXFP2,UFM1,LINC00363,LINC00558,KIAA0226L,SMIM2-IT1,DLEU1,LINC00330,MIR4704,OR7E156P,FOXO1,LINC01048,EBPL,DACH1,CLN5,SERPINE3,LMO7DN,SIAH3,MIR548X2,LINC00428,COG3,NAA16,DLEU7,MZT1,MIR621,MYCBP2,LINC00548,LINC00598,DLEU7-AS1,SUCLA2,LINC00457,LINC00374,MINOS1P1,LECT1,MIR18A,UTP14C,LINC00562,LINC00423,LINC00395,MIR4500,MIR5693,LINC00446,SMAD9,SERP2,LINC00437,LINC00397,LINC00364,TPT1,ITM2B,LINC00332,MIR3613,N4BP2L1,LINC00378,SERTM1,NEK5,HTR2A-AS1,SLC25A30,MIR759,COMMD6,LINC01080,TBC1D4,DCT,ATXN8OS,LINC01065,MIR17HG,LMO7,NDFIP2,LCP1,LMO7DN-IT1,EDNRB,CCDC70,RNASEH2B,DGKH,GPC5-AS2,LINC00390,KLHL1,LINC00379,LINC00433,TRPC4,STARD13-AS,LINC00547,FRY-AS1,NUFIP1,LINC01055,KCNRG,BORA,IRG1,SPRYD7,EPSTI1,LINC00458,N4BP2L2-IT2,EXOSC8,LRRC63,PHF11,LINC00351,LINC00358,MIR548F5,LINC00400,KLF5,SUPT20H,CCDC169,KL,SLAIN1,MIR15A,ARL11,MYCBP2-AS1,LINC00430,OR7E37P,SPG20-AS1,LINC00377,PCDH17,SMIM2-AS1,LINC01068,SPERT,LOC101929259,LOC101929140,TDRD3,LINC00366,LINC00563,PRR20A,MRPS31P5,SUGT1P3,LINC00381,N4BP2L2,SNORA107,FAM124A,PCDH9,SLC25A15,ZAR1L,LINC01069,WBP4,DCLK1,THSD1,KBTBD7,LHFP,CPB2,LINC00571,RCBTB2,ST13P4,MAB21L1,LINC01049,BRCA2,MIR16-1,MIR622,PRR20E,ESD,NBEA,LINC00383,MIR3169,NUDT15,MED4-AS1,ELF1,GUCY1B2,ALG11,LOC100129307,CCDC169-SOHLH2,ENOX1-AS2,GPC5-AS1,LINC00559,CTAGE11P,POU4F1,CAB39L,SETDB2,CPB2-AS1,CSNK1A1L,MIR5006,SMIM2,LMO7-	1	loss
355	CGTE_05	14	103576202	103596189	14q32.32	EXOC3L4,TNFAIP2	5	gain
356	CGTE_05	14	105412779	105418727	14q32.33	AHNAK2	5	gain
357	CGTE_05	16	570108	920066	16p13.3	RAB11FIP3,HAGHL,NHLRC4,MIR3176,CHTF18,CAPN15,MSLN,WDR90,GNG13,FAM195A,WDR24,FBXL16,MIR5587,C16orf13,RHOT2,JMJD8,RHBDL1,CCDC78,MIR662,FAM173A,LINC00235,STUB1,NARFL,LMF1,RPUSD1,RAB40C,PRR35,PRR25,PIGQ,METRN,WFIKKN1	0	loss
358	CGTE_05	16	920676	943180	16p13.3	LMF1	39	gain

359	CGTE_05	16	943201	2098345	16p13.3	SNORA78,TPSAB1,TELO2,MAPK8IP3,UNKL,EME2,HN1L,RNF151,TPSD1,TMEM204,GNPTG,NPW,BAIAP3,CRAMP1,NTHL1,UBE2I,SPSB3,TBL3,CACNA1H,C16orf91,CLCN7,C1QTNF8,SNORA64,MRPS34,RPL3L,CCDC154,IGFALS,GFER,TPSG1,LMF1-AS1,MEIOB,IFT140,NOXO1,SSTR5,MSRB1,SSTR5-AS1,SOX8,SNORA10,TSR3,LMF1,FAHD1,ZNF598,TPSB2,SYNGR3,NUBP2,MIR3177,TSC2,PTX4,SLC9A3R2,NDUFB10,LINC00254,NME3,HAGH,SNHG9,HS3ST6,RPS2	0	loss
360	CGTE_05	16	2098553	2107366	16p13.3	TSC2	4	gain
361	CGTE_05	16	2225006	2226645	16p13.3	TRAF7	8	gain
362	CGTE_05	16	2227921	2304119	16p13.3	MLST8,E4F1,RNPS1,PGP,TRAF7,DNASE1L2,BRICD5,ECI1,CASKIN1	0	loss
363	CGTE_05	19	281411	1257277	19p13.3	MIER2,ARID3A,PTBP1,CNN2,GRIN3B,FSTL3,SBNO2,MIDN,RNF126,GZMM,ELANE,PRTN3,R3HDM4,CDC34,RNU6-2,FGF22,GPX4,PLPP2,WDR18,PALM,HCN2,STK11,BSG,HMHA1,MIR3187,KISS1R,SHC2,C2CD4C,ATP5D,AZU1,TMEM259,TPGS1,CBARP,POLRMT,POLR2E,ABCA7,MADCAM1,PLPPR3,THEG,MED16,MIR4745,CFD,ODF3L2,PRSS57,MISP	0	loss
364	CGTE_05	19	1428756	2042872	19p13.3	REXO1,BTBD2,UQCR11,ADAMTSL5,REEP6,PCSK4,TCF3,KLF16,PLK5,MBD3,DAZAP1,MKNK2,MIR1909,ABHD17A,MEX3D,ONECUT3,APC2,ADAT3,RPS15,CSNK1G2,C19orf25,CSNK1G2-AS1,LOC100288123,ATP8B3,SCAMP4	0	loss
365	CGTE_05	19	2043072	2213958	19p13.3	MKNK2,IZUMO4,MOB3A,AP3D1,DOT1L	3	gain
366	CGTE_05	19	2214331	4212716	19p13.3	ZNF554,GADD45B,ZNF556,C19orf71,EEF2,MIR6789,MIR1227,PLEKHJ1,TMPRSS9,SNORD37,DOT1L,ATCAY,MIR7850,TJP3,JSRP1,AMH,HMG20B,MFSD12,TIMM13,MIR1268A,ZNF57,SMIM24,NCLN,FZR1,TLE2,MAP2K2,LMNB2,MATK,MIR4321,PIP5K1C,CACTIN,ZNF77,THOP1,SLC39A3,CACTIN-AS1,TLE6,ANKRD24,LINGO3,DIRAS1,LOC100996351,APBA3,RAX2,DAPK3,CREB3L3,S1PR4,MRPL54,GIPC3,C19orf35,SF3A2,NFIC,MIR637,SGTA,DOHH,ZNF555,MIR7108,GNA15,SPPL2B,TBXA2R,OAZ1,GNA11,SIRT6,ZBTB7A,AES,NMRK2,LSM7,CELF5,PIAS4,ZFR2,GNG7	1	loss
367	CGTE_05	19	4212727	4217438	19p13.3	ANKRD24	4	gain
368	CGTE_05	19	32959547	33687804	19q13.11	CEP89,TDRD12,FAAP24,PDCD5,GPATCH1,LRP3,SLC7A9,ANKRD27,RHPN2,RGS9BP,NUDT19,DPY19L3,WDR88	3	gain
369	CGTE_05	20	61937229	62051221	20q13.33	LOC100130587,CHRNA4,KCNQ2,COL20A1	0	loss
370	CGTE_05	20	62055397	62071117	20q13.33	KCNQ2	7	gain
371	CGTE_05	21	10793919	26961234	21q21.3-q21.2	C21orf91-OT1,MIR155HG,MIR3156-3,ANKRD20A11P,BTG3,LINC00317,BAGE2,CHODL-AS1,MIR8069-2,LOC339622,RBM11,LINC00158,LOC388813,CYP4F29P,TMPRSS15,MIRLET7C,D21S2088E,USP25,ANKRD30BP2,BAGE4,MIR8069-1,NCAM2,BAGE5,MIR3118-1,ABCC13,NRIP1,LIPI,BAGE3,C21orf91,MIR99A,MIR155,SAMSN1,CHODL,LINC00308,LINC00515,MIR125B2,HSPA13,MIR99AHG,MIR548XHG,BAGE,LINC00320,LOC102724188,LOC101927843,TPTE,MRPL39,LOC101927869,SAMSN1-AS1,CXADR,LINC01425,POTED,LINC01549	1	loss
372	CGTE_05	21	28326132	33347104	21q22.11-q21.3	KRTAP19-5,KRTAP22-1,LOC101927973,KRTAP13-3,MIR4759,BACH1-IT2,KRTAP8-1,KRTAP20-3,KRTAP20-1,LOC284825,KRTAP25-1,KRTAP19-3,KRTAP13-1,CCT8,KRTAP23-1,KRTAP15-1,KRTAP24-1,GRIK1,KRTAP22-2,KRTAP6-3,KRTAP13-2,N6AMT1,SOD1,KRTAP21-1,BACH1,KRTAP20-4,KRTAP13-4,GRIK1-AS2,LTN1,KRTAP7-1,RWDD2B,LINC00161,LINC00314,KRTAP19-1,KRTAP19-8,KRTAP6-2,KRTAP19-6,CLDN8,KRTAP27-1,KRTAP11-1,LINC00307,KRTAP19-2,KRTAP6-1,USP16,MAP3K7CL,KRTAP21-3,SCAF4,KRTAP26-1,ADAMTS5,CLDN17,MIR5009,LINC00113,TIAM1,GRIK1-AS1,KRTAP19-4,HUNK,KRTAP20-2,KRTAP21-2,KRTAP19-7,LINC00189,MIR4327	1	loss

373	CGTE_05	21	45705905	45713885	21q22.3	AIRE	0	loss
374	CGTE_05	21	45714266	45725833	21q22.3	AIRE,PFKL	4	gain
375	CGTE_05	21	47532625	47536771	21q22.3	COL6A2	6	gain
376	CGTE_05	21	47537235	47540582	21q22.3	COL6A2	0	loss
377	CGTE_05	21	47540864	47542862	21q22.3	COL6A2	5	gain
378	CGTE_05	22	42805414	50321250	22q13.33-q13.2	CYB5R3,LOC100506679,NUP50,TBC1D22A-AS1,TTLL12,UPK3A,LL22NC03-75H12.2,LOC101927526,RRP7BP,ATP5L2,POLDIP3,ZBED4,FBLN1,ARHGAP8,MIR1249,ARFGAP3,LINC00898,BIK,MPPED1,MIR3619,A4GALT,RRP7A,MCAT,LOC284930,PRR34,GTSE1-AS1,PPARA,PNPLA3,CELSR1,PKDREJ,SMC1B,FAM118A,MIRLET7B,TTLL1,FAM19A5,CDPF1,LDOC1L,PNPLA5,TTC38,PRR5,C22orf34,LINC00229,MIR3201,SERHL,RNU12,LOC284933,WNT7B,MIRLET7BHG,LINC01310,NFAM1,MIR4535,LOC730668,ATXN10,MIR3667,SAMM50,PHF21B,GTSE1,MIR4762,LINC00899,CRELD2,RIBC2,MIRLET7A3,SERHL2,TBC1D22A,PARVB,MIR4763,PRR5-ARHGAP8,CERK,SCUBE1,PACSIN2,TSPO,LINC01589,EFCAB6-AS1,KIAA0930,KIAA1644,BRD1,ALG12,PRR34-AS1,GRAMD4,EFCAB6,TRMU,PARVG,LINC00207,NUP50-AS1,SULT4A1	3	gain
379	CGTE_05	22	50354564	51178618	22q13.33	SBF1,SHANK3,NCAPH2,MOV10L1,TUBGCP6,PPP6R2,LMF2,PLXNB2,KLHDC7B,SELO,CHKB,ADM2,MAPK8IP2,DENND6B,IL17REL,PANX2,SYCE3,MIOX,TYMP,CPT1B,MIR6821,ODF3B,MLC1,CHKB-CPT1B,MAPK12,TRABD,ARSA,PIM3,SCO2,ACR,MAPK11,CHKB-AS1,HDAC10	1	loss
380	CGTE_05	X	152806771	152807402	Xq28	ATP2B3	59	gain
381	CGTE_05	X	152807749	153005886	Xq28	BCAP31,FAM58A,ABCD1,ATP2B3,DUSP9,SLC6A8,PNCK,LOC105373383	3	gain
382	CGTE_05	X	153009124	153050632	Xq28	ABCD1,SRPK3,PLXNB3	0	loss
383	CGTE_06	1	1560821	1585764	1p36.33	MMP23B,MIB2,MMP23A,CDK11B	1	loss
384	CGTE_06	1	3410927	3416707	1p36.32	MEGF6	1	loss
385	CGTE_06	1	76354942	76365421	1p31.1	MSH4	3	gain
386	CGTE_06	2	136505755	136511849	2q21.3	UBXN4	3	gain
387	CGTE_06	2	189852711	189859006	2q32.2	COL3A1	3	gain
388	CGTE_06	2	239006590	239051663	2q37.3	SCLY,KLHL30,UBE2F-SCLY,ESPNL	1	loss
389	CGTE_06	2	242755629	242757743	2q37.3	NEU4	0	loss
390	CGTE_06	3	5212190	5214335	3p26.1	ARL8B	4	gain
391	CGTE_06	4	76860988	76862042	4q21.1	NAAA	3	gain
392	CGTE_06	4	106320186	106345470	4q24	PPA2	3	gain
393	CGTE_06	5	13902159	13914063	5p15.2	DNAH5	3	gain
394	CGTE_06	6	76600922	76604644	6q14.1	MYO6	4	gain
395	CGTE_06	6	116901472	116911498	6q22.1	RWDD1	3	gain
396	CGTE_06	6	155095075	155109163	6q25.2	SCAF8	3	gain
397	CGTE_06	7	2686284	2689366	7p22.3	TTYH3	1	loss
398	CGTE_06	7	4874185	4876257	7p22.1	RADIL	1	loss
399	CGTE_06	7	73153008	73184107	7q11.23	ABHD11,CLDN3	1	loss
400	CGTE_06	8	145001406	145006398	8q24.3	PLEC	1	loss
401	CGTE_06	10	32751578	32760178	10p11.22	CCDC7	3	gain
402	CGTE_06	10	35929124	35929972	10p11.21	FZD8	0	loss
403	CGTE_06	11	11804661	11895517	11p15.3	USP47,MIR8070	1	loss
404	CGTE_06	11	64706867	64713743	11q13.1	C11orf85	3	gain
405	CGTE_06	11	82893443	82895930	11q14.1	PCF11	3	gain
406	CGTE_06	11	107427485	107431645	11q22.3	ALKBH8	3	gain
407	CGTE_06	11	110104147	110143259	11q22.3	RDX	1	loss
408	CGTE_06	12	80726778	80733058	12q21.31	OTOGL	3	gain
409	CGTE_06	12	85546791	85556647	12q21.31	LRRIQ1	3	gain

410	CGTE_06	14	21821691	21825567	14q11.2	SUPT16H	1	loss
411	CGTE_06	14	21825980	21826672	14q11.2	SUPT16H	3	gain
412	CGTE_06	14	39777764	39815150	14q21.1	CTAGE5	3	gain
413	CGTE_06	14	78197423	78202311	14q24.3	SNW1	3	gain
414	CGTE_06	14	103987257	103988824	14q32.32	CKB	1	loss
415	CGTE_06	16	596835	626274	16p13.3	CAPN15,PIGQ,NHLRC4,PRR35	1	loss
416	CGTE_06	18	732776	739994	18p11.32	YES1	3	gain
417	CGTE_06	19	1108294	1109781	19p13.3	SBNO2	0	loss
418	CGTE_06	19	23545423	23557675	19p12	ZNF91	4	gain
419	CGTE_06	19	58867388	58880780	19q13.43	ZNF497,ZNF837	1	loss
420	CGTE_06	20	25056799	25058636	20p11.21	VSX1	3	gain
421	CGTE_06	20	32691357	32699896	20q11.22	EIF2S2	1	loss
422	CGTE_06	20	60897209	60898961	20q13.33	LAMA5	1	loss
423	CGTE_06	20	60988989	60990496	20q13.33	RBBP8NL	1	loss
424	CGTE_06	20	62492901	62496853	20q13.33	TPD52L2,ABHD16B	1	loss
425	CGTE_06	21	17236573	17242549	21q21.1	USP25	3	gain
426	CGTE_06	21	34115884	34117803	21q22.11	PAXBP1	3	gain
427	CGTE_06	21	35276119	35279031	21q22.11	ATP5O	3	gain
428	CGTE_06	22	21983160	21984303	22q11.21	YDJC	1	loss
429	CGTE_07	1	865582	1153422	1p36.33	MIR200A,ISG15,MIR200B,AGRN,RNF223,LINC01342,PERM1,TNFRSF4,SAMD11,NOC2L,C1orf159,PLEKHN1,TNFRSF18,SDF4,KLHL17,MIR429,TTLL10,HES4	1	loss
430	CGTE_07	1	16272217	16382287	1p36.13	ZBTB17,CLCNKA,C1orf64,HSPB7,CLCNKB	1	loss
431	CGTE_07	1	21039926	21054085	1p36.12	KIF17,SH2D5	1	loss
432	CGTE_07	1	22168061	22176754	1p36.12	HSPG2	1	loss
433	CGTE_07	1	26508831	26517306	1p36.11	CNKSR1,CATSPER4	1	loss
434	CGTE_07	1	32264966	32280960	1p35.2	SPOCD1	1	loss
435	CGTE_07	2	220316348	220337841	2q35	SPEG	0	loss
436	CGTE_07	2	233200442	233405564	2q37.1	ALPI,ECEL1P2,ALPPL2,ECEL1,ALPP,CHRNG,PRSS56,CHRND,DIS3L2	1	loss
437	CGTE_07	2	241627709	242007386	2q37.3	AQP12A,SNED1,LOC200772,KIF1A,AGXT,C2orf54	1	loss
438	CGTE_07	3	46717731	46729876	3p21.31	ALS2CL	1	loss
439	CGTE_07	3	47040129	47049379	3p21.31	NBEAL2	1	loss
440	CGTE_07	3	48506203	48511291	3p21.31	ATRIP,SHISA5,TREX1	1	loss
441	CGTE_07	3	49688283	49700052	3p21.31	BSN	1	loss
442	CGTE_07	3	52525405	52562758	3p21.1	STAB1,NT5DC2,NISCH	1	loss
443	CGTE_07	3	126193786	126207145	3q21.3	ZXDC,UROC1	0	loss
444	CGTE_07	4	7728521	7736506	4p16.1	SORCS2	1	loss
445	CGTE_07	5	42762552	42799640	5p12	CCDC152	0	loss
446	CGTE_07	5	68670373	68692528	5q13.2	RAD17	1	loss
447	CGTE_07	5	172386735	172421778	5q35.1	RPL26L1,ATP6V0E1	0	loss
448	CGTE_07	6	499614	532622	6p25.3	EXOC2	4	gain
449	CGTE_07	6	31743635	31750847	6p21.33	VARS,VWA7	1	loss
450	CGTE_07	6	33131385	33136881	6p21.32	COL11A2	1	loss
451	CGTE_07	6	126298763	126320800	6q22.32	TRMT11,HINT3	0	loss
452	CGTE_07	6	128793785	128841575	6q22.33	PTPRK	1	loss
453	CGTE_07	6	134373515	134436545	6q23.2	HMGA1P7,SLC2A12	0	loss
454	CGTE_07	7	77026574	77227224	7q11.23	GSAP,PTPN12,LOC101927243	1	loss
455	CGTE_07	7	128470626	128586101	7q32.1	FLNC,ATP6V1F,IRF5,LOC100130705,KCP	1	loss
456	CGTE_07	7	142561726	142566926	7q34	EPHB6	1	loss
457	CGTE_07	8	21937870	21974073	8p21.3	FAM160B2,DMTN,HR,NUDT18	1	loss
458	CGTE_07	8	22052895	22067207	8p21.3	BMP1	1	loss
459	CGTE_07	8	142264799	142498765	8q24.3	PTP4A3,GPR20,LINC01300,MROH5	1	loss

460	CGTE_07	9	2029073	2635599	9p24.2-p24.3	VLDLR-AS1,VLDLR,SMARCA2	3	gain
461	CGTE_07	10	74033915	74035094	10q22.1	DDIT4	0	loss
462	CGTE_07	11	1217087	2604764	11p15.5	IGF2,MIR675,MOB2,MRPL23-AS1,ASCL2,LSP1,MIR4298,IFITM10,TOLLIP,KRTAP5-2,KRTAP5-5,MIR6744,MRPL23,MIR483,MUC5B,MIR4686,MIR7847,IGF2-AS,LINC01150,TRPM5,TOLLIP-AS1,KRTAP5-AS1,TSPAN32,LINC01219,FAM99A,TH,TSSC4,CTSD,BRSK2,HOTS,KRTAP5-4,KRTAP5-6,SYT8,TNNT3,CD81,TNNI2,CD81-AS1,INS,MUC5AC,C11orf21,H19,INS-IGF2,KRTAP5-3,KRTAP5-1,FAM99B,SNORD131,KCNQ1,DUSP8	1	loss
463	CGTE_07	11	64012729	64035133	11q13.1	PLCB3,PPP1R14B	1	loss
464	CGTE_07	11	68080262	68204494	11q13.2	LRP5	1	loss
465	CGTE_07	12	21349819	21370246	12p12.1	SLCO1B1	3	gain
466	CGTE_07	12	21375108	21377972	12p12.1	SLCO1B1	0	loss
467	CGTE_07	12	48370605	48378435	12q13.11	COL2A1	1	loss
468	CGTE_07	12	132394741	132404706	12q24.33	ULK1	0	loss
469	CGTE_07	12	132504556	132505835	12q24.33	EP400	0	loss
470	CGTE_07	12	132824366	133202381	12q24.33	GALNT9,LOC100130238,LRCOL1,LOC101928416,POLE,P2RX2,MIR6763,FBRSL1	1	loss
471	CGTE_07	13	53237221	53241143	13q14.3	SUGT1	0	loss
472	CGTE_07	14	24525427	24530031	14q11.2	LRRC16B	1	loss
473	CGTE_07	14	56122734	56128384	14q22.3	KTN1	0	loss
474	CGTE_07	14	73719374	73735366	14q24.2	PAPLN	1	loss
475	CGTE_07	14	103576202	103596189	14q32.32	TNFAIP2,EXOC3L4	1	loss
476	CGTE_07	14	105238565	105356150	14q32.33	ZBTB42,AKT1,CEP170B,LINC00638	1	loss
477	CGTE_07	14	105408121	105418727	14q32.33	AHNAK2	1	loss
478	CGTE_07	14	105613683	105685603	14q32.33	BRF1,JAG2,MIR6765,NUDT14	1	loss
479	CGTE_07	15	56681236	56704649	15q21.3	TEX9	1	loss
480	CGTE_07	15	70963151	70975218	15q23	UACA	0	loss
481	CGTE_07	15	90174779	90216675	15q26.1	KIF7,PLIN1	1	loss
482	CGTE_07	15	96875256	96877520	15q26.2	MIR1469,NR2F2	0	loss
483	CGTE_07	17	4269495	4446596	17p13.2	UBE2G1,SPNS2,SPNS3,MYBBP1A	1	loss
484	CGTE_07	17	4794182	4796852	17p13.2	MINK1	1	loss
485	CGTE_07	17	4936271	4937753	17p13.2	SLC52A1	0	loss
486	CGTE_07	17	7750242	7758746	17p13.1	TMEM88,KDM6B	1	loss
487	CGTE_07	17	27893041	27936461	17q11.2	GIT1,ANKRD13B,TP53I13,ABHD15	1	loss
488	CGTE_07	17	42162408	42166002	17q21.31	HDAC5	0	loss
489	CGTE_07	17	48613895	48655943	17q21.33	CACNA1G-AS1,SPATA20,EPN3,CACNA1G	1	loss
490	CGTE_07	17	71354022	71397492	17q25.1	SDK2	1	loss
491	CGTE_07	17	73720662	73843114	17q25.1	GALK1,H3F3B,MIR4738,WBP2,UNC13D,UNK,ITGB4	1	loss
492	CGTE_07	17	77709048	77768980	17q25.3	CBX2,ENPP7,CBX8	0	loss
493	CGTE_07	17	79982121	80017938	17q25.3	RAC3,GPS1,LRRC45,RFNG,DUS1L,DCXR	1	loss
494	CGTE_07	17	80399908	80402453	17q25.3	HEXDC,C17orf62	0	loss
495	CGTE_07	17	81009462	81051005	17q25.3	METRNL,B3GNTL1	0	loss
496	CGTE_07	18	43703293	43705945	18q21.1	HAUS1	1	loss
497	CGTE_07	20	33580830	33591426	20q11.22	TRPC4AP,MYH7B	0	loss
498	CGTE_07	20	61584142	61595750	20q13.33	SLC17A9	0	loss
499	CGTE_07	20	62076003	62192288	20q13.33	KCNQ2,EEF1A2,SRMS,PTK6,PPDPF,C20orf195,HELZ2	0	loss
500	CGTE_07	21	44527494	44590554	21q22.3	LOC102724652,CRYAA,FRGCA,U2AF1,LOC106780825	1	loss
501	CGTE_07	21	46032355	46117586	21q22.3	KRTAP10-11,KRTAP12-4,KRTAP10-12,TSPEAR,KRTAP12-3,KRTAP10-8,KRTAP10-9,KRTAP10-10,KRTAP12-1,KRTAP12-2	0	loss
502	CGTE_07	21	47414031	47570485	21q22.3	COL6A2,FTCD,COL6A1	1	loss

503	CGTE_07	22	50687759	50752992	22q13.33	HDAC10,PLXNB2,MAPK12,MAPK11,DENND6B	1	loss
504	CGTE_07	X	153677986	153698846	Xq28	PLXNA3,MIR6858,FAM50A	0	loss
505	CGTE_08	1	7869054	7879584	1p36.23	PER3	3	gain
506	CGTE_08	1	17914895	17930249	1p36.13	ARHGEF10L	3	gain
507	CGTE_08	1	55181422	55183407	1p32.3	MROH7-TTC4,TTC4	1	loss
508	CGTE_08	1	151786120	151803732	1q21.3	RORC	3	gain
509	CGTE_08	1	226258963	226349478	1q42.12	H3F3A,H3F3AP4,ACBD3	3	gain
510	CGTE_08	2	11347875	11359321	2p25.1	ROCK2	1	loss
511	CGTE_08	2	120704037	120712918	2q14.2	PTPN4	4	gain
512	CGTE_08	4	153247155	153252022	4q31.3	FBXW7	3	gain
513	CGTE_08	5	68606940	68660886	5q13.2	CCDC125,TAF9,AK6	1	loss
514	CGTE_08	5	177659476	177669204	5q35.3	PHYKPL,COL23A1	3	gain
515	CGTE_08	6	44150609	44187528	6p21.1	LOC101929726,SLC29A1,CAPN11	3	gain
516	CGTE_08	7	56169540	56879607	7p11.2	LOC101928401,LOC100240728,NUPR2,LOC650226,DKFZp434L192,LOC401357,CHCHD2	1	loss
517	CGTE_08	7	100675346	100685166	7q22.1	MUC17	1	loss
518	CGTE_08	7	154759386	154760815	7q36.2	PAXIP1	3	gain
519	CGTE_08	8	38814719	38827435	8p11.22	PLEKHA2	3	gain
520	CGTE_08	8	74659565	74692759	8q21.11	UBE2W,STAU2	4	gain
521	CGTE_08	8	89068321	89128887	8q21.3	MMP16	3	gain
522	CGTE_08	8	144640461	144669107	8q24.3	GSDMD,MROH6,EEF1D,NAPRT	3	gain
523	CGTE_08	10	7213850	7230771	10p14	SFMBT2,SNORD129	1	loss
524	CGTE_08	10	10989252	11207409	10p14	CELF2-AS2,LINC00710,CELF2	3	gain
525	CGTE_08	10	95148751	95155979	10q23.33	MYOF	3	gain
526	CGTE_08	10	115410207	115413966	10q25.3	NRAP	3	gain
527	CGTE_08	11	43349403	43419699	11p12	API5,TTC17	3	gain
528	CGTE_08	11	58379591	58384384	11q12.1	ZFP91,ZFP91-CNTF	3	gain
529	CGTE_08	12	40876490	40888021	12q12	MUC19	1	loss
530	CGTE_08	12	44165045	44176299	12q12	IRAK4	3	gain
531	CGTE_08	12	124821449	124832882	12q24.31	MIR6880,NCOR2	3	gain
532	CGTE_08	12	125591573	125619411	12q24.31	AACS	3	gain
533	CGTE_08	14	105408121	105419610	14q32.33	AHNAK2	1	loss
534	CGTE_08	16	70697469	70699130	16q22.1	MTSS1L	4	gain
535	CGTE_08	17	38977313	39018051	17q21.2	TMEM99,KRT10,KRT12	3	gain
536	CGTE_08	17	39032536	39034549	17q21.2	KRT20	4	gain
537	CGTE_08	17	79603864	79609613	17q25.3	NPLOC4,TSPAN10	4	gain
538	CGTE_08	19	3750797	3751556	19p13.3	APBA3,TJP3,MIR1268A	1	loss
539	CGTE_08	19	45496478	45506872	19q13.32	RELB,CLPTM1	4	gain
540	CGTE_08	22	39077900	39078343	22q13.1	TOMM22	3	gain
541	CGTE_09	1	861266	1278216	1p36.33	PLEKHN1,TAS1R3,MIR429,SAMD11,MIR6808,TTLL10,CPSF3L,RNF223,TNFRSF18,MIR6726,HES4,PERM1,NOC2L,ISG15,LINC01342,MIR200A,KLHL17,SCNN1D,B3GALT6,CPTP,PUSL1,FAM132A,MIR6727,UBE2J2,C1orf159,SDF4,AGRN,DVL1,TNFRSF4,MIR200B,ACAP3	1	loss
542	CGTE_09	1	2116304	3699358	1p36.32-p36.33	TNFRSF14,PLCH2,PANK4,TPRG1L,MIR551A,PRKCZ,PRDM16,MEGF6,FAM213B,FAAP20,HES5,LINC00982,TTC34,SMIM1,WRAP73,MIR4251,LOC115110,ARHGEF16,TP73-AS1,MORN1,LRRC47,MMEL1,SKI,LOC100129534,PEX10,CCDC27,TP73,RER1,LOC100996583,ACTRT2	1	loss
543	CGTE_09	1	17017581	17327039	1p36.13	ESPNP,CROCC,MIR3675,MST1L,FAM231A,FAM231C,ATP13A2,MFAP2	1	loss
544	CGTE_09	1	226895482	228247261	1q42.12-q42.13	PSEN2,LOC100130093,PRSS38,WNT3A,SNAP47,ZNF678,MIR5008,ADCK3,CDC42BPA,ITPKB,WNT9A,JMJD4,ZNF847P	1	loss

545	CGTE_09	1	245582765	248202584	1q44	OR2L5,OR13G1,OR2L13,MIR3916,TFB2M,OR2W3,OR2W5,ZNF670-ZNF695,OR1C1,OR2L8,OR2L1P,SMYD3,OR2G3,SCCPDH,OR2AK2,OR14A16,OR11L1,ZNF669,LINC01341,ZNF695,CNST,KIF26B,C1orf229,ZNF670,VN1R5,OR2G2,OR6F1,LOC255654,AHCTF1,OR2L2,OR2T8,ZNF124,GCSAML,NLRP3,GCSAML-AS1,ZNF496,TRIM58,OR2B11,OR2C3	1	loss
546	CGTE_09	2	136875496	219696166	2q31.1-q22.3	CPS1,GALNT13,ERBB4,MMADHC,MIR4775,LINC01473,LOC101929570,PLA2R1,SSB,PHOSPHO2-KLHL23,SLC11A1,MREG,HAGLR,RFTN2,ANKRD44-IT1,AOX1,ABI2,VWC2L-IT1,RAPH1,NFE2L2,PPP1R1C,MIR7157,CATIP-AS1,CREB1,COBLL1,LINC01124,MIR6888,SESTD1,SLC38A11,GLS,LOC200726,PLEKHM3,LOC101927156,HOXD8,LANCL1-AS1,C2orf88,RQCD1,PKP4,WIPF1,PECR,KIAA2012,GORASP2,LOC643072,PRKRA,KIF5C,NDUFB3,STK36,DNAJC10,ALS2CR12,LRP1B,GCSHP3,HSPD1,MOB4,DLX1,LOC105747689,OSGEPL1-AS1,NDUFS1,ORC4,TLK1,PABPC1P2,AAMP,GPD2,BOLL,RPE,TMEM237,DLX2-AS1,MIR7845,MARS2,OSGEPL1,LOC101929512,LOC100130452,TMEFF2,HAGLROS,KANSL1L,MIR561,FAM117B,MYL1,COQ10B,LOC101927619,PRKAG3,MDH1B,PKI55,CFLAR,CCDC173,TRAK2,FKBP7,LOC101928273,SPAG16,MIR6810,PDK1,GRB14,UBR3,C2orf80,CERS6-AS1,MTX2,DCAF17,MPP4,CCNYL1,EPC2,ANKAR,ATP5G3,ALS2,TFPI,SPC25,MIR2355,LINC01090,TTLL4,DPP4,INPP1,VWC2L,DIRC1,ARPC2,ALS2CR11,G6PC2,CCDC148,LOC100130451,NABP1,PSMD14,CYP20A1,LOC101928103,LOC101927431,MIR26B,CHRNA1,MYO3B,SATB2-AS1,LOC102724849,RBM45,CSRNP3,CYTIP,MIR548AE1,SLC4A10,OLA1,CALCRL,HOXD12,LYPD6,PIKFYVE,AHCTF1P1,UBE2E3,GPBAR1,LINC00607,RPRM,BBS5,NUP35,MIR4438,SUMO1,GCA,MIR1246,ABCA12,FZD7,LOC101928020,LY75-CD302,SCN9A,FSIP2,ZNF385B,HOXD10,USP37,LOC101927055,IKZF2,TBR1,LOC101929260,KLF7,GALNT3,BARD1,TNFAIP6,MIR3606,EVX2,LOC102725079,RND3,HAT1,CASP8,OSBPL6,KCNH7,SCN7A,KLHL23,PRPF40A,PPIL3,LOC105616981,FASTKD2,SP3,CASP10,HOXD4,SNORD11,LOC101929532,CFLAR-AS1,TTC30B,MIR9500,ZSWIM2,IGFBP5,RUFY4,STRADB,STAM2,TMEM169,DAPL1,LOC101929378,NXPH2,CIR1,DHRS9,CPS1-IT1,BAZ2B,SF3B1,THSD7B,GCG,TTC30A,UPP2,KIAA1715,LOC100507140,CARF,ZC3H15,TTC21B,MIR933,HOXD13,WDR12,LINC01305,TANK,SMARCAL1,LOC101927406,SCN2A,TTN-AS1,MYO1B,MARCH7,ZDBF2,ICA1L,BMPR2,LANCL1,COL3A1,FIGN,ACVR2A,LOC101929680,BZW1,CXCR1,LINC01116,SPATS2L,MIR6512,HOXD11,ZEB2,FTCDNL1,LOC101928386,CD302,IGFBP2,LYPD6B,HOXD1,LOC100130691,WDR75,MIR7704,HIBCH,LY75,TNS1,NRP2,HSPE1,DYTN,SCRN3,DLX2,SNORA105A,ZNF804A,MIR4776-1,SGOL2,LOC101927027,C2orf66,NMI,MIR6809,PARD3B,MIR10B,CCDC141,MIR4444-2,RAPGEF4-AS1,CRYGD,MIR1245B,CYBRD1,PLCD4,GALNT5,XIRP2,TEX41,SSFA2,CCDC148-AS1,MSTN,VIL1,SNORD11B,HNMT,MAP2,SLC40A1,HOXD9,GPR1,SLC39A10,ARL6IP6,EEF1B2,METTL5,ITGA4,SCN1A,CXCR2P1,ACVR1,RPL37A,ERMN,SPOPL,KLHL41,HECW2,HOXD3,RBM43,NAB1,RBMS1,ACVR1C,GPR155,B3GALT1,CYP27A1,MIR548N,DUSP19,MIR1302-4,NEMP2,METAP1D,INO80D,CWC22,ITGA6,PDE11A,ITGAV,LOC100506124,MBD5,SNORD70,GTF3C3,RNU6-2,LOC101926913,SNORA70F,NEUROD1,MFSD6,ZEB2-AS1,MIR6513,NEB,DIRC3-AS1,GAD1,KCNJ3,STK17B,TTN,PPIG,ACADL,TNP1,GTDC1,PCGEM1,RNF25,MIR4437,CRYGB,LOC100505984,UNC80,LOC101929633,LOC101927865,CHN1,ERICH2,CCDC150,CTDSP1,C2orf47,DYNC1I2,MIR4773-1,ORMDL1,MIR3130-1,ANKRD44,ITGB6,MIR4776-2,METTL8,IDH1-AS1,NCKAP1,LOC101927196,NOSTRIN,SDPR,KCTD18,PHOSPHO2,RAPGEF4,SP5,LOC100996579,NIF3L1,LINC01117,LINC01614,ICOS,NR4A2,STAT4,STK39,ZAK,LOC100144595,SATB2,MARCH4,HOXD-AS2,LOC101929231,TTC21B-AS1,LOC101929319,MIR3130-	3	gain

547	CGTE_09	3	161220793	175178037	3q26.2-q26.31	OTOL1,LRRIQ4,MIR551B,TNIK,SLC2A2,SLITRK3,MIR569,TNFSF10,SERPINI2,GOLIM4,LOC100128164,NAALADL2-AS2,SAMD7,MIR4789,EIF5A2,PRKCI,LINC01324,SERPINI1,NAALADL2-AS3,GHSR,TMEM212,ZBBX,LINC01322,NLGN1,FNDC3B,GPR160,CLDN11,SPATA16,RPL22L1,TMEM212-AS1,NAALADL2,PDCD10,LRRC34,ACTRT3,MIR1263,MIR6828,PLD1,WDR49,SKIL,MECOM,BCHE,TERC,ECT2,NLGN1-AS1,SEC62,MIR548AY,LINC01330,NCEH1,MYNN,SLC7A14,LOC100507661,LRRC31,LINC01192,SI,LINC01327,EGFEM1P,PHC3	3	gain
548	CGTE_09	3	184542572	193386346	3q27.3-q27.2	OPA1-AS1,ADIPOQ,VPS8,RPL39L,PYDC2,P3H2,EHHADH-AS1,TP63,SNORA81,RFC4,HRG,LOC102724699,SNAR-I,IGF2BP2,RTP4,TMEM41A,OSTN-AS1,SNORA4,OSTN,CCDC50,LIPH,DGKG,EIF4A2,TBCCD1,FLJ42393,TPRG1,C3orf70,RTP2,ETV5,OPA1,MIR28,RTP1,MIR944,SST,AHSG,EHHADH,TPRG1-AS1,CRYGS,LPP,TRA2B,MAP3K13,KNG1,BCL6,LINCR-0002,LPP-AS1,LOC253573,FGF12,ATP13A5-AS1,TPRG1-AS2,MGC2889,FGF12-AS1,IGF2BP2-AS1,MIR548AQ,CLDN1,TMEM207,UTS2B,MB21D2,MASP1,ATP13A4-AS1,LOC101929106,DNAJB11,IL1RAP,SNORA63,ATP13A5,MIR5588,LOC100131635,LOC344887,SENP2,ST6GAL1,LPP-AS2,SNORD2,ADIPOQ-AS1,HRASLS,MIR1248,FETUB,CLDN16,ATP13A4,GMNC,P3H2-AS1	3	gain
549	CGTE_09	4	366559	367119	4p16.3	ZNF141	3	gain
550	CGTE_09	4	367247	2498948	4p16.3	LOC100129917,DGKQ,IDUA,MAEA,ABCA11P,ZFYVE28,SLBP,SCARNA22,ATP5I,MIR4800,CTBP1-AS,NKX1-1,NAT8L,SLC26A1,MXD4,MIR943,TMEM175,TACC3,RNF4,CFAP99,FAM53A,WHSC1,HAUS3,TMED11P,CTBP1-AS2,UVSSA,ZNF141,PDE6B,TMEM129,ZNF721,CTBP1,CPLX1,C4orf48,NELFA,CRIPAK,LOC100130872,POLN,FGFR3,LETM1,MFSD7,FGFRL1,RNF212,PIGG,SPON2,GAK,PCGF3,MYL5	1	loss
551	CGTE_09	4	185340610	187631041	4q35.2-q35.1	SNX25,MIR3945HG,ACSL1,KLKB1,CFAP97,HELT,FAT1,SLED1,F11-AS1,PRIMPOL,F11,MIR4455,ANKRD37,UFSP2,CCDC110,CASP3,CENPU,MTNR1A,C4orf47,MIR3945,FLJ38576,PDLIM3,SORBS2,CYP4V2,FAM149A,TLR3,IRF2,LVCAT8,LRP2BP,LINC01093,SLC25A4	1	loss
552	CGTE_09	5	26886040	45645712	5p12-p14.1	C5orf34,SLC1A3,SEPP1,HMGCS1,LOC101926960,DROSHA,TTC23L,NUP155,RANBP3L,LMBRD2,FYB,MIR3650,LOC101929681,LOC100506639,GHR,AGXT2,LIFR,UGT3A1,NNT,C1QTNF3-AMACR,RXFP3,NPR3,NIPBL-AS1,MIR579,CDH9,TARS,SNORD72,CARD6,BRCAT107,RAI14,CAPSL,GDNF-AS1,DAB2,OXCT1,PRKAA1,C5orf51,SPEF2,UGT3A2,FGF10-AS1,C1QTNF3,C9,MRPS30,FLJ32255,OSMR,MIR4279,MROH2B,GDNF,PLCXD3,LINC00603,LOC648987,EGFLAM,LOC101929645,BRIX1,PRLR,PDZD2,LOC101929660,C5orf42,LOC101926940,SLC45A2,CDH6,LOC153684,ZFR,RAD1,FGF10,AMACR,MTMR12,ADAMTS12,IL7R,C6,LINC01021,MIR580,CCL28,C5orf22,WDR70,ANXA2R,NNT-AS1,PTGER4,LOC100506548,ZNF131,LOC340113,HCN1,PAIP1,C5orf28,LIFR-AS1,TTC33,BRCAT54,LOC100506406,NIM1K,GOLPH3,SKP2,RPL37,RICTOR,EGFLAM-AS4,SUB1,MIR7641-2,EGFLAM-AS2,OXCT1-AS1,NADK2,OSMR-AS1,LSP1P3,LINC01265,FBXO4,C7,NIPBL,DNAJC21,LOC100132356,CCDC152	3	gain

553	CGTE_09	6	24511919	27218565	6p22.2-p22.3	HIST1H2BC,GMNN,HIST1H3F,HIST1H2BK,MIR3143,HIST1H4C,LINC00240,HIST1H2AD,HIST1H2BI,HIST1H1A,GUSBP2,LOC101928663,HIST1H3E,HIST1H2BA,HIST1H2BF,KIAA0319,SLC17A2,HIST1H2AG,C6orf62,BTN2A2,SCGN,HIST1H1T,HIST1H1C,BTN3A2,C6orf229,HIST1H4B,HIST1H4A,HIST1H2AH,HIST1H4D,HIST1H2AC,ALDH5A1,HIST1H1E,SLC17A3,HIST1H3B,TRIM38,TDP2,HIST1H3A,HIST1H4I,ABT1,CMAHP,ZNF322,HIST1H3G,HIST1H4F,ACOT13,HIST1H2AB,HIST1H2BE,HIST1H2AE,HIST1H2AA,HIST1H4H,BTN3A1,PRSS16,HIST1H3D,HIST1H4E,HFE,BTN1A1,HIST1H2BB,HIST1H2BJ,LOC285819,FAM65B,SLC17A4,BTN2A1,HIST1H3C,HMGN4,HIST1H1D,BTN2A3P,HIST1H4G,HIST1H2APS1,BTN3A3,HIST1H2BH,LRRC16A,HIST1H2BD,HIST1H2BG,HCG11,LOC100270746,SLC17A1	3	gain
554	CGTE_09	6	43226761	43253116	6p21.1	TTBK1	1	loss
555	CGTE_09	6	43265966	84567149	6p12.2-q12	SMAP1,RSPH9,MIR586,MTRNR2L9,HTR1B,GUSBP4,ZNF318,RWDD2A,CRISP1,GLYATL3,KLHL31,PKHD1,HMGN3-AS1,PRSS35,MIR6780B,COL21A1,SUPT3H,NFKBIE,KHDC1L,PTCHD4,ME1,LINC01526,DST,LOC101928540,BMP5,LGSN,LINC01621,LRRC1,DEFB112,IBTK,TCTE1,GCLC,VEGFA,MB21D1,TMEM14A,RIPPLY2,LOC101926915,PTP4A1,TINAG,COL12A1,MIR4647,EEF1A1,LCAL1,KHDC3L,RCAN2,LOC101926898,MLIP,SENP6,ADGRF5,SNAP91,PRIM2,RIMS1,LOC101927082,DEFB114,MIR4463,CENPQ,IMPG1,BCKDHB,KIAA1586,FAM46A,LOC101930010,RUNX2,COL9A1,MTO1,MIR30A,LINC00472,CRISP2,DEFB133,GCM1,LOC730101,LOC101928516,LOC101929705,GSTA4,LOC102723883,KCNQ5-IT1,OOEP,RAB23,TFAP2D,LOC101927020,KHDRBS2,KHDC1,ADGRB3,FBXO9,HSP90AB1,CD109,AARS2,MAD2L1BP,B3GAT2,DDX43,SLC25A27,SLC25A51P1,XPO5,LINC00680-GUSBP4,MIR548U,IL17F,POLH,MRPL14,ENPP4,LRRC73,LOC101927048,TDRD6,RPS16P5,GFRAL,COL19A1,CYP39A1,LOC101927136,SLC22A7,RNY4,OGFRL1,EFHC1,PAQR8,DEFB110,TFAP2B,SNORD141A,SPATS1,SLC17A5,C6orf141,PHIP,SLC35B2,ICK,MEP1A,MLIP-IT1,ANKRD66,UBE3D,FILIP1,MIR4642,EVADR,ADGRF1,IL17A,YIPF3,IRAK1BP1,GSTA3,MIR4282,LINC00680,MRPS18A,KCNQ5-AS1,MIR30C2,PLA2G7,LOC100506804,LINC01512,ADGRF2,LOC101927189,CDC5L,TTK,LOC101928489,ENPP5,FAM135A,LOC100506188,HMGN3,BAG2,LOC101929726,CAPN11,OPN5,TMEM151B,SDHAF4,GSTA1,MYO6,EYS,TPBG,MUT,GSTA2,DEFB113,TMEM30A,DPPA5,LOC101928280,TRAM2,HMGCLL1,MEI4,KCNQ5,MIR5685,PGM3,GSTA7P,DOPEY1,TNFRSF21,PGK2,DLK2,TJAP1,SNORD141B,LOC101926962,ADGRF4,GTPBP2,MCM3,HCRTR2,LCA5,SH3BGRL2,CLIC5,LINC01564,MIR206,LOC101927211,GSTA5,CRISP3,C6orf223,ZNF451,POLR1C,BEND6,TMEM63B,MIR133B,CD2AP,ABCC10,ELOVL4,LOC441155,TRAM2-AS1,PHF3,LOC101928307,SLC29A1,RHAG,ELOVL5,LINCMD1,LMBRD1,LINC01626,FAM83B,CRIP3,COX7A2	3	gain
556	CGTE_09	6	111346572	112392713	6q21	WISP3,SLC16A10,RPF2,GSTM2P1,REV3L,TRAF3IP2-AS1,FYN,TRAF3IP2,KIAA1919,TUBE1	3	gain

557	CGTE_09	7	195557	55799980	7p14.1-p12.1	SUN1,CLK2P1,MIR6837,FERD3L,KBTBD2,HOXA6,ANKRD61,PPIA,TARP,MIR6838,CYTH3,TMED4,LRRC72,FAM126A,JAZF1,INHBA-AS1,MPLKIP,SEC61G,FBXL18,TAX1BP1,SEPT7P2,FKBP14,RAPGEF5,MRPS24,NOD1,LINC01450,SNX10,LOC100506178,LFNG,TBRG4,LOC101927811,SOSTDC1,KDELR2,LOC100506497,C7orf26,LOC401312,SFRP4,NXPH1,ABCA13,HNRNPA2B1,MIR550B2,PKD1L1,HOXA11-AS,MACC1,ANLN,INTS1,CYCS,CBX3,PHF14,RNF216,LOC401324,TMEM196,RBAKDN,MYL7,CCM2,KCCAT333,MYO1G,STARD3NL,YAE1D1,LOC100129603,LOC101926963,GLCCI1,FAM220A,TRG-AS1,ZNF815P,POU6F2,UMAD1,SUGCT,MIR4655,RAMP3,MEOX2-AS1,AP5Z1,TRA2A,ELMO1-AS1,URGCP-MRPS24,SCIN,NFE2L3,ZFAND2A,VWDE,GPR146,MRPL32,GPNMB,LINC01176,LOC100506725,FKBP9,EPDR1,RPL23P8,NUPL2,MIR3146,VPS41,NPVF,LOC401320,POM121L12,SNHG15,TBX20,EGFR,HDAC9,BZW2,MAFK,NUDT1,ELFN1,POLR2J4,IKZF1,VWC2,MTURN,PDGFA,KLHL7-AS1,POU6F2-AS1,NT5C3A,SDK1,HOXA7,CCDC126,LOC100506895,MIR550A1,DDX56,SNX8,AGR2,NPSR1-AS1,SNORA5B,PRKAR1B,ELMO1,HIBADH,LINC01445,AGMO,CHST12,SP4,MIR1200,NPC1L1,GPR141,KIAA0895,SCRN1,ZNRF2,C7orf25,SKAP2,SPDYE1,MIR196B,CDCA7L,HERPUD2,GARS,LOC101927181,FAM221A,BLVRA,GLI3,DPY19L1P1,NDUFA4,LINC01447,LINC01446,OCM,TFAMP1,PER4,DPY19L1P2,RASA4CP,GHRHR,PMS2,RALA,SP8,PSMA2,NPSR1,PDE1C,BBS9,DAGLB,WI2-2373I1.2,RP9P,ZNF890P,STEAP1B,C7orf72,NPY,LOC101927769,HUS1,MIR550B1,LOC100505921,EGFR-AS1,OGDH,MIR339,CAMK2B,RNF216-IT1,GRID2IP,PLEKHA8,HOXA11,DFNA5,NACAD,CCZ1,C7orf69,GGCT,ETV1,LOC101928168,FTSJ2,LOC441204,ZNF853,PRR15,DNAH11,INMT,FSCN1,COL28A1,CPVL,GNA12,TOMM7,UPP1,FAM183BP,ICA1,STK17A,LOC101927354,ARL4A,GCK,POLD2,HOXA10,MIR6874,MICALL2,INMT-FAM188B,TMEM184A,TWIST1,ZNRF2P1,RSPH10B2,CHN2,FKBP9P1,HRAT92,LOC101927630,CREB5,AOAH,MIR4648,PRPS1L1,LINC00525,AEBP1,C1GALT1,FOXK1,WIPF3,HOXA4,DBNL,LINC01449,MIR550A3,RAC1,ACTB,NME8,AOAH-IT1,HOXA9,AMZ1,POLM,UBE2D4,PURB,EVX1-AS,LOC101927391,CRHR2,HOXA10-HOXA9,LOC221946,PAPOLB,OSBPL3,HOXA2,RPA3,COX19,RADIL,SNORD93,LOC101928618,ELDR,HOXA-AS3,INHBA,LOC101927021,ZDHHC4,PGAM2,CYP2W1,ADAP1,IQCE,MIR3943,C7orf31,SNORA5C,HPVC1,ITGB8,ANKMY2,YKT6,ZMIZ2,MIR1183,HOXA3,GPER1,PSMG3,DGKB,FIGNL1,WIPI2,C7orf65,GET4,LOC646762,BMPER,ADCYAP1R1,GRIFIN,HOTTIP,EVX1,MIOS,HOXA10-AS,MIR148A,CDK13,DPY19L1,CCZ1B,NEUROD6,URGCP,LOC101927668,ZNRF2P2,MPP6,ZNF316,LOC101927000,DPY19L2P1,MIR4649,LOC102724484,TWISTNB,LOC100996654,LINC01448,HECW1,C7orf71,AHR,LOC100505938,IL6,COBL,HOTAIRM1,CDC14C,LOC100130673,AMPH,H2AFV,SNX13,EIF3B,MIR6836,AGR3,PMS2CL,DPY19L2P3,RBAK,MMD2,MALSU1,HOXA13,CARD11,VSTM2A-OT1,LSM5,RNF216P1,AQP1,RBAK-RBAKDN,TNS3,SNORA9,VSTM2A,TSPAN13,THSD7A,UNCX,LINC00265,USP42,GRB10,MIR1302-6,LOC541472,FAM188B,TTYH3,MIR4657,RPS2P32,ELFN1-AS1,MIR3683,C7orf50,LINC00957,KIAA0087,MIR4656,SEPT7,FAM20C,DDC-AS1,MACC1-	3	gain

558	CGTE_09	7	55874895	76144521	7p11.2-q11.21	TRIM50,LOC100287704,SBDS,ZNF680,INTS4P2,CCL26,STAG3L2,GS1-124K5.11,LINC00174,UPK3B,SNORA15,MIR4283-1,NSUN5P2,KCTD7,LINC01005,GTF2IRD2B,CCT6A,EIF4H,POM121C,RABGEF1,LOC100240728,MIR4284,ZNF479,BAZ1B,MIR4651,LINC01372,LOC641746,SUMF2,ELN,DTX2,MIR4650-1,ZNF713,GUSBP10,FZD9,GTF2I,PHKG1,BCL7B,NCF1C,LOC101928401,MIR590,ZNF716,STX1A,SEPT14,GBAS,YWHAEP1,LOC650226,ZNF727,PMS2P5,CRCP,ZNF92,HSPB1,GTF2IRD1,FDPSP2,PMS2P4,ZNF107,NCF1,WBSCR16,FKBP6,NCF1B,GATSL2,LOC100128885,LOC102723427,WBSCR22,PMS2P3,ZP3,TMEM120A,GTF2IP1,ZNF679,AUTS2,MIR3147,CCL24,ZNF735,CHCHD2,VPS37D,MIR4283-2,LOC100101148,MDH2,WBSCR28,PMS2P7,CCT6P3,LOC100507468,STYXL1,TRIM74,TPST1,PMS2P2,MIR6839,ABHD11,MIR3914-1,DNAJC30,ZNF273,ZNF138,POR,GTF2IRD1P1,TMEM248,CCT6P1,NSUN5,STAG3L4,CALN1,SPDYE7P,GUSB,WBSCR17,SNORA14A,CLDN4,CLDN3,TRIM73,GTF2IP4,MLXIPL,MIR3914-2,TBL2,LOC101926943,NSUN5P1,ZNF117,ABHD11-AS1,HIP1,GTF2IRD2,RFC2,LOC100287834,TYW1,SBDSP1,WBSCR27,SSC4D,LOC541473,STAG3L1,SPDYE5,SRRM3,LOC100996437,VKORC1L1,YWHAG,LOC100130849,DKFZp434L192,MRPS17,ZNF733P,NUPR2,ASL,LIMK1,SPDYE8P,ERV3-1,PSPH,LOC401357,LAT2,TYW1B,SNORA22,LOC441242,ZNF736,STAG3L3,POM121,MIR4650-2,RHBDD2,CLIP2	4	gain
559	CGTE_09	7	76825553	100014101	7q21.2-q21.11	DPY19L2P4,LOC102723885,COPS6,MIR5692A2,FAM200A,BRI3,LOC101409256,TFPI2,CYP51A1-AS1,PDK4,ASB4,CPSF4,FZD1,STEAP1,PVRIG,PON2,SAMD9,SEMA3D,KPNA7,LAMTOR4,MIR591,AKAP9,DMTF1,DYNC1I1,BAIAP2L1,TMEM243,AZGP1,BET1,ZCWPW1,MIR653,PILRA,GAL3ST4,CYP3A7-CYP3A51P,ARPC1A,CASD1,MBLAC1,TAC1,TMEM130,KRIT1,MYH16,MTERF1,SMURF1,ZNF394,STEAP2-AS1,PON3,FGL2,KIAA1324L,LOC101927356,STAG3L5P,SEMA3E,MIR3609,MCM7,CDK14,GJC3,CYP3A5,MGC72080,SLC25A13,MAGI2-AS2,LOC101927243,ATP5J2,PTCD1,GTPBP10,LOC100128317,STEAP4,OCM2,CCDC146,GNAT3,ADAM22,OR2AE1,LMTK2,TMEM60,ZNF655,CFAP69,MIR106B,MIR548M,MIR5692A1,LRRD1,STEAP2,SAMD9L,PEX1,PDAP1,PEG10,PILRB,PON1,CALCR,ZNF789,CD36,TP53TG1,LOC100506136,GRM3,SDHAF3,ASNS,BUD31,PMS2P1,HGF,MGC16142,MIR25,CNPY4,DLX5,MIR4658,MAGI2-AS3,STAG3L5P-PVRIG2P-PILRB,FAM133DP,C7orf62,FAM133B,ABCB4,VPS50,ZSCAN21,APTR,GATAD1,ABCB1,GNG11,AZGP1P1,SHFM1,PVRIG2P,ZKSCAN1,RBM48,MAGI2,GPC2,CROT,CYP3A7,MIR489,SLC25A40,RUNDC3B,COL1A2,NPTX2,C7orf76,ZNF3,SRI,MIR93,GS1-259H13.2,BHLHA15,SGCE,TAF6,DLX6-AS1,ATP5J2-PTCD1,GATS,PPP1R9A,CYP51A1,SCARNA28,GNGT1,C7orf43,DLX6,RPL13AP17,PTPN12,TRRAP,TECPR1,ZKSCAN5,CACNA2D1,PHTF2,PCLO,MIR6840,MIR4652,HEPACAM2,ZSCAN25,CYP3A43,ANKIB1,CLDN12,LOC101927446,LOC101927497,DBF4,TRIM4,GNAI1,SEMA3C,ARPC1B,CYP3A4,LOC101927269,ZNF804B,CDK6,GSAP,LOC101927378,STAG3,RSBN1L,SPDYE3,SEMA3A,AP4M1,LOC101927550	4	gain

560	CGTE_09	7	100014678	102553685	7q22.1	MIR4467,MUC3A,POP7,PCOLCE,AGFG2,SAP25,GIGYF1,FBXL13,TFR2,MIR4285,TSC22D4,PPP1R35,ZNHIT1,SLC12A9,RASA4B,TRIM56,RASA4,POLR2J3,CLDN15,EPO,LOC101927746,ALKBH4,PCOLCE-AS1,SPDYE2,ZCWPW1,MIR5090,POLR2J2,LOC102724094,UPK3BL,SERPINE1,MIR6875,MIR548O,LRRC17,SPDYE2B,GNB2,UFSP1,C7orf61,MIR4653,LOC100289561,ACHE,MUC17,MEPCE,POLR2J,IFT22,ORAI2,NAT16,LINC01007,MYL10,MOGAT3,AP1S1,ACTL6B,TRIP6,PRKRIP1,MOSPD3,SH2B2,FAM185A,COL26A1,ZAN,LRWD1,CUX1,SRRT,FBXO24,VGF,EPHB4,MUC12,PLOD3,FIS1,NYAP1,LRCH4,LOC100630923,ZASP,SPDYE6	3	gain
561	CGTE_09	7	102566666	110526812	7q22.1-q31.1	FBXL13,LINC01004,BCAP29,DLD,CDHR3,LHFPL3-AS2,THAP5,DNAJB9,C7orf66,CBLL1,NAPEPLD,ORC5,PNPLA8,RPL19P12,HBP1,LAMB4,EFCAB10,PRKAR2B,KMT2E,PIK3CG,LOC101927870,DUS4L,SLC26A3,LHFPL3,DNAJC2,SLC26A5,SLC26A4,COG5,LRRC17,PMPCB,NRCAM,LAMB1,PSMC2,PUS7,NAMPT,SLC26A4-AS1,RELN,SYPL1,KMT2E-AS1,IMMP2L,DPY19L2P2,LHFPL3-AS1,RINT1,GPR22,CCDC71L,SRPK2,ATXN7L1,EIF3IP1,ARMC10	4	gain
562	CGTE_09	7	110603027	124533842	7q31.2-q31.31	LRRN3,LSM8,CAV1,ANKRD7,IQUB,LINC01393,IFRD1,ASB15,SPAM1,LINC00998,RNU6-2,LSMEM1,LMOD2,CAPZA2,IMMP2L,NDUFA5,LOC101928012,CAV2,TMEM229A,TMEM168,AASS,DOCK4-AS1,HRAT17,FEZF1,WASL,MIR6132,RNF148,LOC102724434,MET,C7orf77,MDFIC,RNF133,FAM3C,LOC101928211,WNT2,TFEC,CTTNBP2,HYAL4,LOC100996249,FOXP2,ST7-OT3,ZNF277,KCND2,LOC102724555,LINC01510,LINC01392,ST7-OT4,ASZ1,LVCAT5,HYALP1,GPR37,WNT16,TES,ST7-AS2,TAS2R16,TSPAN12,GPR85,ST7,CPED1,CFTR,DOCK4,CADPS2,FEZF1-AS1,ING3,PTPRZ1,PPP1R3A,SLC13A1,C7orf60,MIR3666,POT1,ST7-AS1	3	gain

563	CGTE_09	7	124537116	149430183	7q32.3-q32.1	MEST,TSGA13,AHCYL2,TCAF2,AKR1D1,DGKI,MIR6892,TSPAN33,KLHDC10,GCC1,MKRN1,ZNF800,MIR129-1,LOC100506860,CREB3L2,NOBOX,LOC154761,MIR6133,SSMEM1,LRGUK,CLEC2L,RBM28,CLEC5A,TNPO3,SND1,CHRM2,EPHB6,OR2A1,ZNF746,LOC100130880,OR2A25,TMEM178B,MGAM,ARHGEF5,MIR29A,SMO,TMEM213,OR2A14,SLC37A3,CTAGE8,KIAA1549,OR9A2,TAS2R38,PODXL,EZH2,ARHGEF34P,BRAF,PRSS1,MIR4468,OR2F2,IMPDH1,C7orf34,MTRNR2L6,ZNF777,MRPS33,TMEM140,CUL1,TMEM209,KLF14,PRSS37,ARF5,PAX4,MIR6509,POT1-AS1,CPA4,NDUFB2,OR9A4,OR2A2,ZC3HC1,AGK,FAM131B,KEL,LINC-PINT,DENND2A,IRF5,SSBP1,UBN2,TAS2R3,LOC101928782,WDR91,FLJ40288,TRPV5,AGBL3,PRSS58,CTAGE15,LOC101928861,PDIA4,SND1-IT1,FAM180A,LOC101928333,ZNF282,OR2A1-AS1,SVOPL,AKR1B1,LOC100506682,CNOT4,ZNF398,LOC155060,TAS2R4,C7orf73,JHDM1D-AS1,AKR1B10,FLNC,CPA5,CCDC136,KCP,WEE2,OR2A5,PTN,MTPN,NDUFB2-AS1,TAS2R5,MOXD2P,C7orf55-LUC7L2,NRF1,ZYX,HIPK2,MIR592,GSTK1,PLXNA4,PARP12,MKLN1,LRRC4,MIR548F4,TRY2P,MIR548I4,GRM8,C7orf49,MKLN1-AS,LEP,CASP2,TPI1P2,OR2A9P,KDM7A,TCAF2P1,ZC3HAV1L,TMEM139,CNTNAP2,LOC100129148,HILPDA,CALD1,CALU,CTAGE4,TBXAS1,PRRT4,OR6V1,ZC3HAV1,EPHA1,TAS2R60,TRIM24,MIR183,CHCHD3,MIR29B1,SMKR1,TPK1,OR2A42,LOC101928700,MESTIT1,TTC26,KRBA1,COPG2,KIAA1147,CLCN1,CTAGE6,OR6W1P,LOC349160,EPHA1-AS1,LOC101928283,MIR335,STRIP2,LOC101928254,TRPV6,TAS2R40,KLRG2,OR2F1,ATP6V1F,FSCN3,C7orf33,MGAM2,LOC407835,CEP41,MIR96,ZNF786,SLC13A4,ZNF425,ADCK2,EXOC4,MIR490,WEE2-AS1,TCAF1,LUC7L2,ZNF212,BPGM,TAS2R41,PRSS3P2,MGC27345,C7orf55,STRA8,MIR3654,MIR593,GHET1,POT1,METTL2B,TAS2R39,ZNF767P,OR6B1,ATP6V0A4,ZNF783,FAM71F2,OR2A12,FAM71F1,CPA2,LUZP6,LOC101928605,RAB19,MIR182,OR2A20P,UBE2H,AKR1B15,PIP,LINC01000,LOC100130705,ARHGEF35,MIR548T,OPN1SW,SLC35B4,OR2A7,NUP205,CPA1	4	gain
564	CGTE_09	7	149430190	149541812	7q36.1	ZNF467,SSPO,ZNF862,KRBA1	3	gain
565	CGTE_09	7	149543136	150558365	7q36.1	RARRES2,ATP6V0E2,REPIN1,ATP6V0E2-AS1,AOC1,ZNF775,GIMAP1-GIMAP5,LINC00996,GIMAP4,GIMAP6,ACTR3C,ZBED6CL,GIMAP2,GIMAP1,GIMAP7,TMEM176A,LOC728743,TMEM176B,ZNF862,LRRC61,GIMAP5,GIMAP8	4	gain
566	CGTE_09	7	150642408	151093489	7q36.1	TMUB1,ATG9B,CHPF2,CDK5,NUB1,MIR671,AGAP3,NOS3,SLC4A2,WDR86,ASIC3,SMARCD3,KCNH2,FASTK,ABCF2,ASB10,ABCB8,GBX1,IQCA1L	3	gain
567	CGTE_09	7	151097207	158937439	7q36.1-q36.3	C7orf13,HTR5A,UBE3C,VIPR2,SHH,RNF32,MIR153-2,MIR595,PAXIP1-AS2,GALNTL5,HTR5A-AS1,MIR5707,PAXIP1-AS1,WDR86,LOC100506302,LINC00244,LOC101927914,PTPRN2,DPP6,BLACE,LOC100506585,WDR86-AS1,MIR3907,LINC01003,PRKAG2,EN2,MNX1-AS1,LOC389602,FABP5P3,LINC00689,WDR60,PRKAG2-AS1,DNAJB6,NCAPG2,INSIG1,LMBR1,LINC01006,CNPY1,GALNT11,XRCC2,ESYT2,CRYGN,LINC01287,LINC01022,NOM1,MNX1,KMT2C,ACTR3B,PAXIP1,RHEB,LOC285889,RBM33	4	gain

568	CGTE_09	8	190799	28321393	8p22-p23.2	DEFB107A,FBXO25,USP17L2,TUSC3,CSGALNACT1,DEFB109P1,DEFB103A,ZNF596,DEFB130,NKX2-6,LOC101929315,DLGAP2,FAM90A10P,ADAM7,LOC100128993,REEP4,MIR1322,USP17L4,LOC286083,DEFB104A,NAT1,STC1,MIR3926-1,RHOBTB2,NEFM,SLC35G5,DEFB105A,FDFT1,MIR4659B,CLU,LOC100287015,PNOC,STMN4,C8orf58,FAM167A-AS1,TNKS,DEFA1B,C8orf74,FAM86B3P,MICU3,PPP1R3B,DEFA6,PIWIL2,LINC00965,ESCO2,SPAG11A,PDGFRL,LOC254896,FAM160B2,DEFB104B,TRIM35,MIR597,ADAM28,FAM86B2,FAM66A,SCARA5,GATA4,LOC101929172,XKR6,MIR6843,SLC39A14,CLDN23,ZNF705G,SPAG11B,GS1-24F4.2,FBXO16,SGCZ,DEFT1P,BLK,ZNF705D,CNOT7,LINC00599,SLC25A37,SLC7A2,FAM86B1,MIR8055,LOC100506990,LOC101929294,SLC18A1,SORBS3,DEFB107B,EPHX2,SH2D4A,DLC1,CSMD1,MIR7160,PPP2R2A,DOCK5,LOC102467222,MTUS1,MCPH1,MTMR7,KCTD9,TDH,DEFB106B,FAM90A25P,MTMR9,DEFB106A,HR,LOC101927815,CCDC25,MIR4287,DEFB103B,PHYHIP,FAM66D,NKX3-1,PCM1,DEFA5,FAM167A,DEFB109P1B,ASAH1,NAT2,XKR5,TNFRSF10C,MIR3674,ADAMDEC1,MSRA,MIR383,PRSS55,GFRA2,LOC101929237,FGF17,MIR4660,ENTPD4,DLGAP2-AS1,DMTN,ADRA1A,KIAA1456,MIR3622B,CLN8,LOC649352,MIR124-1,FAM87A,GNRH1,SGK223,CDCA2,FGL1,EGR3,BIN3-IT1,FAM66B,MIR596,MYOM2,DEFA10P,EBF2,DEFA11P,TNFRSF10A,DEFB4A,PPP3CC,PEBP4,MIR5692A2,LOC101929128,MIR6841,C8orf49,MIR3622A,DEFB4B,SFTPC,MIR320A,NEIL2,CHRNA2,DEFA1,PTK2B,PSD3,ZNF395,CTSB,SNORA99,LOC729732,MIR4659A,PRR23D2,PBK,LOC286114,MCPH1-AS1,DOK2,CCAR2,CHMP7,USP17L7,USP17L3,DEFB134,POLR3D,KBTBD11-OT1,MIR548I3,ERICH1,LOC101929229,DPYSL2,MIR6842,LOXL2,USP17L1,LPL,ZNF705B,BIN3,DEFB136,MIR598,ARHGEF10,MIR3926-2,RP1L1,ERICH1-AS1,LOC157273,LOC100133267,FAM90A2P,LINCR-0001,INTS10,TNFRSF10D,SCARA3,VPS37A,PNMA2,NUGGC,NEFL,PRR23D1,ERI1,DEFA4,USP17L8,NUDT18,SOX7,DEFB135,DEFA8P,MFHAS1,ATP6V1B2,FGF20,LGI3,PINX1,LZTS1-AS1,LOC389641,LOC286059,KBTBD11,AGPAT5,ANGPT2,XPO7,LOC101929066,LINC00681,TDRP,BMP1,DEFT1P2,DEFA9P,LOC340357,ZDHHC2,MIR5692A1,LOC392196,BNIP3L,LOC100507156,NPM2,FAM66E,FAM90A7P,C8orf48,LONRF1,DEFA3,MIR4286,DEFB1,LINC00208,MIR6876,TNFRSF10B,MSR1,DEFB105B,ELP3,R3HCC1,MIR548V,PDLIM2,LZTS1	3	gain

569	CGTE_09	8	28322874	124786319	8q11.21-q24.12	CPA6,LINC00967,PLEKHA2,SAMD12-AS1,LRRCC1,LOC101929528,PRDM14,ESRP1,C8orf34,FSBP,KCNB2,CA1,MIR2053,NKX6-3,ZBTB10,KIAA1429,LINC00251,PI15,TGS1,PPP1R42,TBC1D31,DCSTAMP,SNORD87,HGSNAT,LINC01607,MIR486-1,EIF4EBP1,KCNV1,LOC101929709,RNF170,GPAT4,CRISPLD1,LOC101929415,SLC30A8,COX6C,ASPH,SDCBP,LOC401463,C8orf46,MIR875,SLC25A32,TERF1,UBE2W,LOC102724710,ANXA13,MBOAT4,ANKRD46,SNHG6,DKK4,FZD6,MATN2,ZHX1-C8orf76,PCMTD1,KLHL38,C8orf87,OTUD6B,SPAG1,TMEM55A,JPH1,FABP12,MIR4288,C8orf4,VPS13B,TMEM64,CHRNA6,CHCHD7,LOC392232,SNTB1,MIR2052HG,HOOK3,DEPTOR,ZFAND1,TRPS1,RGS20,DCTN6,DNAJC5B,SMIM19,LOC101926892,PREX2,LOC101926908,STAR,SDC2,MIR4663,EXTL3-AS1,MIR378D2,TMEM74,ATAD2,OXR1,LYPLA1,PSKH2,MIR4470,C8orf86,TAF2,LOC728024,MIR4661,LOC100288748,ANK1,PTDSS1,UBE2V2,FAM83A-AS1,LOC105375734,SBSPON,FABP9,LOC101927066,UNC5D,LINC01419,LINC01288,LRRC69,WWP1,STMN2,ZFHX4,SLC20A2,TACC1,PMP2,COL14A1,RRS1-AS1,HEY1,ZNF706,LETM2,MIR124-2,AARD,LINC01301,GRHL2,CASC9,SOX17,C8orf37,SFRP1,POLB,TRIM55,UG0898H09,LAPTM4B,CDH17,TRHR,VCPIP1,MRPL15,ZHX1,POMK,TNFRSF11B,ADAM5,MIR548O2,MRPL13,MIR4471,ZFPM2-AS1,CPNE3,WDYHV1,IMPAD1,CNBD1,MIR3149,CA8,POP1,PRKDC,KCNU1,RAD21-AS1,RBM12B,HAS2-AS1,ZFPM2,LINC01602,CPQ,LOC101927543,TEX15,C8orf59,LINC01151,MIR124-2HG,NBN,SDR16C5,MIR7641-2,SMIM18,TP53INP1,MTBP,TMEM70,CEBPD,LSM1,OTUD6B-AS1,MIR3610,COLEC10,PURG,ZNF703,LOC100130964,GOT1L1,EYA1,EFCAB1,LINC01603,POLR2K,NKAIN3,CSPP1,SYBU,MRPS28,SNX31,IDO1,C8orf44-SGK3,OPRK1,LINC01608,ADAM18,MAK16,CHMP4C,MIR7705,SNORA72,PAG1,DPYS,HNF4G,LINC01030,LOC104054148,SBF1P1,LOC101929550,MIR2052,LINC01299,LINC01606,MOS,DERL1,NSMAF,CSMD3,TRIQK,SNORD54,HTRA4,RAD54B,SNX16,INTS9,LINC00535,MIR1273A,TMEM68,PLAT,RB1CC1,MIR5680,IKBKB,TMEM67,SLC26A7,FGFR1,STAU2,UBR5-AS1,RPL30,THAP1,ERICH5,GGH,XKR9,ENY2,BRF2,LOC286177,PENK,BAALC-AS2,LOC105375650,BAALC,LINC01605,ODF1,MYBL1,AP3M2,TSPYL5,EIF3H,SULF1,ABRA,LINC01289,MIR486-2,CHRNB3,PROSC,RRM2B,BAG4,UBXN8,PEX2,GDAP1,RIMS2,DUSP26,GTF2E2,PLPP5,CA3,NPBWR1,LOC100287846,CRH,RDH10-AS1,RPL7,LOC101241902,C8orf34-AS1,IDO2,KLF10,BHLHE22,UQCRB,LOC102724623,LACTB2,LOC102723701,PPP2CB,MTFR1,ADRB3,STK3,PLEKHF2,CYP7A1,SARAF,LOC100500773,LOC101929897,FAM83A,NRG1-IT3,ZFHX4-AS1,C8orf76,DUSP4,RDH10,TTI2,MMP16,ZC2HC1A,LOC102723729,DCAF13,C8orf22,HRSP12,DPY19L4,CYP7B1,FUT10,YTHDF3,SNAI2,ATP6V1H,CTHRC1,LOC101929217,TTPA,LOC102724612,IL7,MIR3148,PKIA,MCM4,CLVS1,XKR4,NCOA2,E2F5,FAM183CP,LEPROTL1,RAB11FIP1,FABP5,SDR16C6P,EXT1,ARMC1,FABP4,FZD3,LINC00293,KAT6A,CCNE2,ENPP2,NDUFAF6,LOC101929470,EIF3E,SLCO5A1,FBXO32,EMC2,TOX,RP1,NRG1-IT1,ST18,GOLGA7,TCEB1,DECR1,LINC01298,LINC00968,ZNF704,RALYL,GSR,MIR5681A,MI	4	gain

570	CGTE_09	8	124787262	142459812	8q24.22-q24.21	MIR4662A,MROH5,LOC101927845,TMEM65,NDUFB9,EFR3A,LINC00964,FER1L6-AS2,LINC00977,MIR6844,AGO2,DENND3,TMEM71,PCAT2,LOC101927822,ST3GAL1,MYC,MIR1205,RNF139-AS1,CASC21,PVT1,FER1L6-AS1,RNF139,TRAPPC9,MIR1207,CCDC26,HHLA1,MIR5194,TATDN1,TG,SQLE,FAM84B,KIAA0196,FER1L6,ASAP1,MIR30D,TMEM75,LOC101927798,CCAT2,SLA,MIR7848,ASAP1-IT1,LINC01591,LINC01300,ASAP1-IT2,COL22A1,PCAT1,PRNCR1,LRRC6,LINC00824,PHF20L1,LOC101927657,LOC101927588,LINC00861,CHRAC1,PTP4A3,FAM49B,POU5F1B,NDRG1,PTK2,NSMCE2,LOC101927915,GPR20,MIR1208,OC90,NCRNA00250,ZFAT,FAM91A1,CCAT1,ZFAT-AS1,MIR4662B,KCNQ3,KHDRBS3,CASC19,TRIB1,CASC11,WISP1,GSDMC,CASC8,MIR30B,SLC45A4,MIR3686,ADCY8,FAM135B,MIR1204,HPYR1,MTSS1,TRMT12,MIR1206,ZNF572,KCNK9	3	gain
571	CGTE_09	8	142476442	142867788	8q24.3	MROH5,MIR1302-7	4	gain
572	CGTE_09	8	143257604	144425549	8q24.3	GLI4,MIR4472-1,TSNARE1,ADGRB1,LY6H,CYP11B2,LINC00051,SLURP1,CDC42P3,THEM6,PSCA,GPIHBP1,ARC,CYP11B1,JRK,ZNF696,TOP1MT,GML,LY6K,LOC100133669,LY6E,LYPD2,LOC100288181,ZFP41,LY6D,LYNX1,MINCR,C8orf31	3	gain
573	CGTE_09	8	144688662	144801648	8q24.3	ZNF707,MAPK15,BREA2,ZNF623,TSTA3,PYCRL,CCDC166	3	gain
574	CGTE_09	8	145133670	145759671	8q24.3	FOXH1,MIR6847,CYC1,CYHR1,SLC52A2,RECQL4,GPT,SCX,TONSL,EXOSC4,DGAT1,MIR939,MIR6893,PPP1R16A,SLC39A4,C8orf82,MAF1,ADCK5,VPS28,SHARPIN,TONSL-AS1,MFSD3,MROH1,LRRC24,KIFC2,FBXL6,LRRC14,SCRT1,MIR6849,HSF1,HGH1,MIR7112,MIR6848,GPAA1,WDR97,ARHGAP39,BOP1,CPSF1,TMEM249	3	gain
575	CGTE_09	8	145763066	146279543	8q24.3	ZNF250,C8orf33,ZNF517,ZNF16,COMMD5,ZNF252P-AS1,ZNF34,ZNF7,ARHGAP39,MIR6850,TMED10P1,RPL8,ZNF252P,ZNF251	4	gain
576	CGTE_09	9	124118311	124545861	9q33.2	DAB2IP,GGTA1P,STOM	1	loss
577	CGTE_09	9	139221204	139277985	9q34.3	DNLZ,DKFZP434A062,GPSM1,SNAPC4,CARD9	1	loss
578	CGTE_09	9	139378808	140435261	9q34.3	CCDC183-AS1,CYSRT1,LRRC26,RNF224,LCN15,PNPLA7,RNF208,NOTCH1,EXD3,SNORA17B,PHPT1,MIR4674,C9orf172,MAN1B1-AS1,SAPCD2,TRAF2,LCN10,C9orf139,DPP7,MIR7114,TMEM203,NALT1,MIR4479,PTGDS,NPDC1,SLC34A3,C8G,C9orf163,RABL6,ENTPD2,MIR4673,NSMF,GRIN1,TUBB4B,C9orf142,TMEM141,LCN12,EGFL7,LCN6,MAMDC4,SNORA17A,TPRN,CCDC183,MAN1B1,ANAPC2,EDF1,ENTPD8,MIR4292,SNHG7,FAM166A,MIR3621,NRARP,C9orf173,UAP1L1,LOC100128593,TOR4A,FUT7,AGPAT2,C9orf173-AS1,SSNA1,MIR6722,NELFB,LCN8,CLIC3,FAM69B,TMEM210,LCNL1,NDOR1,NOXA1,ABCA2,MIR126,FBXW5	1	loss

579	CGTE_09	10	93002	15060108	10p15.1-p15.3	LINC00708,BEND7,IL2RA,RBM17,SNORD129,MIR4481,MIR3155A,SEC61A2,TUBAL3,PROSER2-AS1,AKR1C6P,MIR4293,LOC101928298,ECHDC3,MIR4454,CALML3-AS1,DIP2C,SFTA1P,LOC101928272,CALML3,PRKCQ,SEPHS1,PFKFB3,AKR1E2,SUV39H2,FRMD4A,LINC00704,SNORD142,AKR1C8P,ADARB2-AS1,DHTKD1,HSPA14,USP6NL,ASB13,CCDC3,FAM107B,LINC00707,ZMYND11,CELF2,UCN3,ADARB2,FAM208B,OPTN,MIR3155B,PFKP,LINC00703,MCM10,CDC123,LOC101927964,ITIH2,PRR26,LINC00710,CAMK1D,LINC00702,MIR4480,PITRM1,NET1,IDI2-AS1,LINC00706,MIR7641-2,FBXO18,MIR5699,PRPF18,LINC00701,IDI1,RNU6-2,CDNF,DCLRE1C,AKR1C2,ITIH5,LOC105755953,PHYH,ATP5C1,MIR1265,PROSER2,GATA3,LOC101928150,MEIG1,LINC00200,AKR1C4,LOC101927762,LARP4B,UCMA,AKR1C3,TUBB8,CELF2-AS2,LOC101928322,LOC105376360,UPF2,KIN,IDI2,MIR548AK,AKR1C1,TAF3,SFMBT2,LINC00700,LINC00709,MIR6078,NUDT5,LOC399715,GATA3-AS1,WDR37,LINC00705,CELF2-AS1,PITRM1-AS1,ANKRD16,CALML5,MIR6072,GDI2,IL15RA,LOC101928453,GTPBP4,KLF6,PRKCQ-AS1	3	gain
580	CGTE_09	10	15063746	15089414	10p13	OLAH	5	gain
581	CGTE_09	10	15091618	27700966	10p12.2-p12.32	LOC100499489,MRC1,PDSS1,MIR511,ACBD5,FAM188A,THNSL1,SLC39A12,LRRC37A6P,PTF1A,MLLT10,GPR158,ANKRD26,HACD1,VIM-AS1,ARL5B,TMEM236,RPP38,ACBD7,SLC39A12-AS1,C10orf67,STAM-AS1,MASTL,DNAJC1,COMMD3,NMT2,ST8SIA6-AS1,MIR603,PIP4K2A,NSUN6,C10orf111,NEBL,TRDMT1,CUBN,LINC00264,LINC01516,ABI1,PTCHD3,KIAA1217,CACNB2,LOC101928834,OTUD1,LINC00202-1,MIR1915,PRTFDC1,VIM,YME1L1,MALRD1,C10orf113,LINC00836,MIR4675,EBLN1,LINC01552,C1QL3,RSU1,ITGA8,BMI1,ARMC3,MYO3A,NEBL-AS1,ARHGAP21,PTER,FAM171A1,APBB1IP,GAD2,ST8SIA6,ENKUR,MSRB2,PPIAP30,STAM,LOC101929073,CASC10,COMMD3-BMI1,LINC00202-2,LOC100130992,OLAH,PLXDC2,GPR158-AS1,SPAG6,MIR1254-2,SKIDA1	3	gain
582	CGTE_09	10	27702024	27702739	10p12.1	PTCHD3	5	gain
583	CGTE_09	10	27702744	27793808	10p12.1	PTCHD3,RAB18	1	loss
584	CGTE_09	10	27793879	32854619	10p11.22-p12.1	MIR8086,LINC01517,MKX,ZEB1-AS1,MIR5586,CCDC7,MIR604,MPP7,LINC00837,GOLGA2P6,KIAA1462,LOC101929279,ZNF438,ZEB1,RAB18,SVILP1,EPC1,WAC,MIR7162,LYZL1,WAC-AS1,LYZL2,SVIL-AS1,SVIL,MTPAP,MKX-AS1,BAMBI,MAP3K8,SNORD130,ARHGAP12,LOC102031319,KIF5B,MIR938,ARMC4,C10orf126,LOC101929431	3	gain

585	CGTE_09	10	33140691	62030022	10q11.23-p11.22	NCOA4,SNORA86,CHAT,PARG,MIR4294,FAM21C,ZNF22,MIR5100,PRKG1-AS1,ZNF32-AS1,NRP1,ANTXRLP1,ZNF485,SGMS1-AS1,FAM25C,MTRNR2L7,GDF10,PCDH15,CSGALNACT2,DRGX,MRLN,HNRNPA3P1,GDF2,VSTM4,RASGEF1A,HSD17B7P2,SLC16A9,ZWINT,ARHGAP22,C10orf128,GLUD1P7,FAM25BP,ANKRD30A,ANXA8L1,FAM35BP,IPMK,TFAM,ZNF32-AS3,CISD1,FAM21EP,MARCH8,ASAH2,PGBD3,AGAP6,LRRC18,BMS1P5,LINC00842,CH17-360D5.1,DKK1,MIR605,CXCL12,ZNF25,OGDHL,FAM133CP,HNRNPA1P33,FZD8,C10orf71,AGAP7P,LINC00999,CSTF2T,RET,ANK3,AGAP12P,LINC01553,LINC00841,BMS1,PHYHIPL,MSMB,CCDC6,C10orf25,LOC102724323,C10orf71-AS1,PARD3-AS1,FRMPD2B,ERCC6,ZNF488,LINC01468,LOC441666,FAM21A,ANKRD30BP3,PCAT5,MBL2,CCNYL2,LOC100129055,ZNF37BP,CCEPR,AGAP4,FAM35DP,BMS1P6,PRKG1,ALOX5,FAM170B,ZNF37A,MAPK8,C10orf10,LOC102724719,SEPT7P9,WDFY4,TIMM23,PARD3,ZNF33B,ZNF487,ZNF32,LINC01518,BICC1,MIR3611,HNRNPF,CUL2,TMEM72,CCDC7,RSU1P2,FAM13C,TMEM72-AS1,LINC00844,GPRIN2,LINC00839,C10orf142,GJD4,A1CF,ZNF239,ANXA8,ZFAND4,LINC01264,SLC18A3,FRMPD2,OR13A1,FAM25G,AGAP9,ZNF33A,LINC00993,ZNF248,ZNF32-AS2,UBE2D1,CCNY,ACTR3BP5,MIR4683,NPY4R,ITGB1,SYT15,FAM170B-AS1,LINC00838,ERCC6-PGBD3,MIR3156-1,LINC00840,RASSF4,FXYD4,CREM,SGMS1,MTRNR2L5,C10orf53,ZNF33BP1,CTSLP2,PARGP1,RBP3,TIMM23B,LINC00619,MIR3924,ASAH2B,PTPN20,ANTXRL	3	gain
586	CGTE_09	11	62652685	63403098	11q13.1-q12.3	RARRES3,LGALS12,SLC22A6,SLC22A8,SLC22A25,ATL3,SLC22A9,SLC22A24,SLC22A10,HRASLS5,CHRM1,PLA2G16,SLC3A2,HRASLS2	3	gain
587	CGTE_09	12	7351490	7528112	12p13.31	PEX5,ACSM4,CD163L1	3	gain
588	CGTE_09	12	11035506	18444026	12p13.2-p13.1	PRB1,RERGL,RERG-AS1,ETV6,DUSP16,PRH2,PLBD1-AS1,GSG1,LOH12CR2,MGP,EMP1,LOC100506314,GPR19,BORCS5,HIST4H4,GPRC5A,RERG,TAS2R14,PIK3C2G,PLBD1,PRB2,MIR614,DDX47,TAS2R19,TAS2R30,MANSC1,DERA,TAS2R43,TAS2R31,ERP27,LMO3,MGST1,PRH1-TAS2R14,MIR613,HTR7P1,ATF7IP,CREBL2,MIR1244-3,H2AFJ,SMCO3,GRIN2B,TAS2R13,SKP1P2,SLC15A5,TAS2R50,MIR7641-2,TAS2R46,RPL13AP20,TAS2R42,STRAP,PRH1-PRR4,APOLD1,TAS2R20,PTPRO,PRB3,LINC01559,HEBP1,MIR1244-2,CDKN1B,LINC01489,PDE6H,LINC01252,EPS8,ART4,PRB4,GPRC5D,MIR3974,ARHGDIB,MIR1244-4,C12orf60,MIR1244-1,FAM234B,BCL2L14,GUCY2C,SMIM10L1,LRP6,PRH1,WBP11	3	gain
589	CGTE_09	12	18446808	18474000	12p12.3	PIK3C2G	4	gain
590	CGTE_09	12	40874156	40885045	12q12	MUC19	1	loss
591	CGTE_09	12	70731482	76767237	12q21.1-q15	GLIPR1,RAB21,TBC1D15,ZFC3H1,ATXN7L3B,MRS2P2,TRHDE-AS1,CAPS2,TSPAN8,KCNMB4,LGR5,KRR1,TMEM19,TPH2,LOC100507377,KCNC2,NAP1L1,TRHDE,PHLDA1,CNOT2,OSBPL8,PTPRB,BBS10,LOC101928137,PTPRR,THAP2,GLIPR1L1,GLIPR1L2	3	gain

592	CGTE_09	13	79939685	95227094	13q31.2-q31.3	MIR17HG,SLITRK6,GPC6-AS2,MIR4500,LINC00440,SLITRK5,MIR19A,GPC5-AS2,MIR19B1,MIR92A1,DCT,LINC01068,LINC00564,RBM26,LINC01038,MIR4500HG,NDFIP2-AS1,LOC105370306,LINC00363,TGDS,GPC6,LINC00410,GPC6-AS1,LINC00353,LINC00377,NDFIP2,LINC00379,LINC00433,LINC00559,MIR20A,LINC00397,LINC01049,LINC00351,GPC5-AS1,LINC00333,LINC01080,LINC00375,LINC01040,LINC00382,RBM26-AS1,SNORA107,LINC00430,SPRY2,LINC00380,GPC5,MIR18A,LINC01047,SLITRK1,MIR622,MIR17	3	gain
593	CGTE_09	14	104569793	104573242	14q32.33	ASPG	0	loss
594	CGTE_09	14	104573497	105964268	14q32.33	LOC100507437,BRF1,BTBD6,AHNAK2,SIVA1,CRIP2,AKT1,ASPG,LINC00638,ZBTB42,PLD4,GPR132,PACS2,MIR203A,JAG2,CRIP1,TEX22,C14orf180,MTA1,LOC102723354,INF2,ADSSL1,MIR203B,CDCA4,NUDT14,CEP170B,MIR4710,MIR6765,C14orf80,KIF26A,C14orf79,TMEM179	1	loss
595	CGTE_09	16	570108	2324778	16p13.3	MIR1225,SSTR5-AS1,TPSAB1,SNORA78,RNPS1,CCDC78,TSC2,SOX8,TMEM204,MIR3177,ECI1,TBL3,NME3,CRAMP1,MIR5587,MLST8,TPSD1,RAB11FIP3,MIR6511B2,CAPN15,RPUSD1,CLCN7,DNASE1L2,NTHL1,PRR25,GNPTG,MIR4516,MIR6511B1,UBE2I,HAGH,IGFALS,UNKL,LMF1-AS1,MIR3176,MAPK8IP3,MSRB1,CCDC154,HAGHL,RPS2,RAB40C,SNHG9,E4F1,MIR4717,NOXO1,MIR3677,RHOT2,PIGQ,WDR24,JMJD8,TRAF7,MRPS34,LOC106660606,GNG13,STUB1,NARFL,LINC00235,NHLRC4,IFT140,FBXL16,MEIOB,C16orf91,NDUFB10,EME2,SPSB3,SNORD60,MIR662,BRICD5,MSLN,NPW,HS3ST6,LINC00254,RPL3L,TSR3,PGP,WDR90,SNORA10,MIR3180-5,TPSB2,NUBP2,RHBDL1,SYNGR3,CACNA1H,WFIKKN1,C1QTNF8,SNORA64,RAB26,HN1L,SSTR5,RNF151,TPSG1,PRR35,PKD1,CASKIN1,ZNF598,SLC9A3R2,FAHD1,METRN,FAM195A,MIR940,C16orf13,SNHG19,BAIAP3,FAM173A,CHTF18,GFER,LMF1,TELO2,PTX4	1	loss
596	CGTE_09	16	56480484	56485826	16q12.2	OGFOD1,NUDT21	4	gain
597	CGTE_09	16	67580394	67806001	16q22.1	RANBP10,CTCF,RLTPR,GFOD2,FAM65A,PARD6A,ENKD1,C16orf86,ACD	1	loss
598	CGTE_09	17	37840724	37883204	17q12	ERBB2,PGAP3,MIR4728	44	gain
599	CGTE_09	17	37883544	37898926	17q12	GRB7,MIEN1,ERBB2	4	gain
600	CGTE_09	17	37901465	37922730	17q12	GRB7,IKZF3	44	gain
601	CGTE_09	17	39633331	40044090	17q21.2	FKBP10,KRT14,KRT17,NT5C3B,KRT15,KRT9,ACLY,LINC00974,P3H4,JUP,KRT42P,KRT16,KLHL11,KRT36,MIR6510,KRT13,KLHL10,GAST,EIF1,KRT35,HAP1,KRT19	8	gain
602	CGTE_09	17	48733090	48742629	17q21.33	ABCC3	46	gain
603	CGTE_09	17	49247375	49302591	17q21.33	NME1-NME2,NME2,MBTD1	4	gain
604	CGTE_09	17	49337922	49357882	17q21.33	UTP18	44	gain
605	CGTE_09	17	49362689	49375199	17q21.33	UTP18	42	gain
606	CGTE_09	17	49567658	49825410	17q21.33	CA10	5	gain
607	CGTE_09	17	50008283	50212436	17q21.33-q22	CA10	40	gain
608	CGTE_09	17	79031491	80054361	17q25.3	MYADML2,MIR3186,NPLOC4,MAFG-AS1,PCYT2,ARL16,DCXR,SLC38A10,DUS1L,MIR3065,FSCN2,MIR338,AATK-AS1,MIR4740,PDE6G,TMEM105,LINC00482,FASN,CEP131,NPB,MIR657,SIRT7,STRA13,AATK,TSPAN10,MRPL12,ARHGDIA,SLC25A10,SNORD134,RAC3,PYCR1,FAAP100,ALYREF,ENTHD2,ACTG1,LRRC45,MAFG,BAHCC1,BAIAP2,C17orf89,ASPSCR1,HGS,PPP1R27,RFNG,CCDC137,NOTUM,MIR1250,ANAPC11,GPS1,MIR6786,FAM195B,GCGR,LOC100130370,OXLD1,P4HB	1	loss

609	CGTE_09	18	21293982	34289379	18q11.2-q12.1	KCTD1,CDH2,DSC3,ZSCAN30,DSG1-AS1,CABYR,DSC2,SLC25A52,LOC729950,AQP4,NOL4,FHOD3,MIR187,RNF125,SS18,DSC1,MIR8057,MEP1B,LOC105372071,DSG1,ELP2,MOCOS,RNF138,KLHL14,DTNA,ZNF396,HRH4,LAMA3,MIR3975,TTC39C-AS1,TRAPPC8,ZNF397,ASXL3,DSG3,LINC01543,TTC39C,GAREM1,DSCAS,PCAT18,MIR320C2,B4GALT6,INO80C,DSG4,MIR3929,DSG2,IMPACT,OSBPL1A,TAF4B,MIR302F,MAPRE2,DSG2-AS1,ZNF521,C18orf21,AQP4-AS1,SLC39A6,RPRD1A,GALNT1,ZNF271P,CHST9,TTR,CCDC178,WBP11P1,ZNF24,PSMA8	3	gain
610	CGTE_09	18	59739858	59751883	18q21.33	PIGN	4	gain
611	CGTE_09	19	281411	2382610	19p13.3	LINGO3,KISS1R,HCN2,CNN2,CSNK1G2,ABHD17A,C19orf25,PLPPR3,ADAT3,SBNO2,MADCAM1,APC2,PRTN3,MIR1909,C2CD4C,ADAMTSL5,C19orf35,RNU6-2,MIER2,SPPL2B,SF3A2,ARID3A,ABCA7,LOC100288123,HMHA1,POLRMT,CDC34,BSG,MIR1227,GAMT,BTBD2,ATP8B3,CSNK1G2-AS1,AZU1,AP3D1,CBARP,C19orf24,NDUFS7,CFD,MIR4745,MIR3187,PLK5,JSRP1,LSM7,GRIN3B,MUM1,MISP,FGF22,UQCR11,REXO1,PLEKHJ1,RNF126,ONECUT3,GPX4,MKNK2,PLPP2,STK11,MBD3,PALM,CIRBP-AS1,TMEM259,PRSS57,PCSK4,ATP5D,EFNA2,ELANE,IZUMO4,MOB3A,SCAMP4,MEX3D,MIR6789,ODF3L2,AMH,OAZ1,THEG,POLR2E,GZMM,MIDN,R3HDM4,TPGS1,KLF16,REEP6,WDR18,TCF3,SHC2,MED16,PTBP1,DOT1L,RPS15,CIRBP,FSTL3,DAZAP1,MIR4321	1	loss
612	CGTE_09	19	3094703	3831871	19p13.3	DOHH,ZFR2,MATK,S1PR4,MIR1268A,NCLN,MRPL54,TBXA2R,GNA15,GIPC3,C19orf71,HMG20B,CACTIN-AS1,MFSD12,SMIM24,GNA11,FZR1,CELF5,NFIC,PIP5K1C,CACTIN,TJP3,LOC100996351,RAX2,APBA3	1	loss
613	CGTE_09	19	19740953	19746684	19p13.11	GMIP	3	gain
614	CGTE_09	20	36407500	45216846	20q12-q11.23	ZSWIM3,DHX35,HNF4A-AS1,LOC101927159,YWHAB,LOC339568,SNORA71D,KIAA1755,ADA,TOMM34,ZNF335,CTNNBL1,NCOA5,CD40,PCIF1,TOX2,RALGAPB,PI3,GTSF1L,SNORA71C,ZNF334,L3MBTL1,MMP9,LPIN3,SNX21,WFDC5,KCNK15-AS1,ADIG,SNHG17,EPPIN,HNF4A,TP53TG5,MIR6812,STK4,SPINT3,FITM2,WFDC8,ELMO2,ZNF663P,EMILIN3,KCNK15,JPH2,SPINT4,SERINC3,VSTM2L,TOP1,PLCG1-AS1,SLC32A1,WISP2,ACOT8,TGM2,SLC12A5,SGK2,WFDC9,EPPIN-WFDC6,LINC01620,LOC149684,SLC35C2,SYS1,OSER1,LINC01430,ARHGAP40,NEURL2,TTPAL,PIGT,SNORA71B,SRSF6,SDC4,WFDC3,SEMG1,GDAP1L1,DNTTIP1,PLCG1,DBNDD2,OCSTAMP,WFDC10B,SLPI,TTI1,IFT52,RBPJL,ZSWIM1,MKRN7P,MIR3646,PLTP,ACTR5,CHD6,KCNS1,LINC01260,LINC01370,SEMG2,CTSA,FAM83D,WFDC2,WFDC13,R3HDML,SPATA25,OSER1-AS1,SNORA60,ZHX3,WFDC6,MYBL2,SYS1-DBNDD2,PABPC1L,MAFB,STK4-AS1,PPP1R16B,PKIG,BPI,SNORA71E,SNORA71A,MATN4,CDH22,RIMS4,LBP,WFDC12,RPRD1B,SNHG11,WFDC10A,TNNC2,PTPRT,MIR3617,WFDC11,SLC13A3,MIR6871,UBE2C	3	gain
615	CGTE_09	20	45217797	45362666	20q13.12	TP53RK,SLC13A3,SLC2A10	4	gain
616	CGTE_09	20	45607142	48301193	20q13.12-q13.13	LINC00494,CSE1L,DDX27,PREX1,CSE1L-AS1,LOC100131496,NCOA3,LINC01522,PTGIS,SNORD12C,ZFAS1,ZNFX1,ZMYND8,ARFGEF2,B4GALT5,EYA2,MIR3616,STAU1,LINC01523,SNORD12,SULF2,SNORD12B,KCNB1	3	gain
617	CGTE_09	20	57430497	57466900	20q13.32	GNAS,LOC101927932	4	gain
618	CGTE_09	20	62165533	62222147	20q13.33	GMEB2,C20orf195,HELZ2,PTK6,SRMS	1	loss
619	CGTE_09	21	39739442	40250959	21q22.2	ETS2,LOC400867,ERG,LINC00114,LOC101928398	16	gain

620	CGTE_09	21	46899797	47609908	21q22.3	LSS,COL18A1,SPATC1L,LOC101928796,PCBP3,SLC19A1,COL6A2,LOC100129027,FTCD,COL6A1	1	loss
621	CGTE_09	22	22385409	23236045	22q11.22	IGLL5,MIR650,ZNF280A,PRAME,BMS1P20,ZNF280B,VPREB1,POM121L1P,MIR5571,GGTLC2,LL22NC03-63E9.3	3	gain
622	CGTE_09	X	134124786	134155914	Xq26.3	SMIM10,FAM127C	3	gain
623	CGTE_09	Y	2655076	20826588	Yq11.221-Yp11.2	TTTY2B,NLGN4Y,CDY2B,TTTY19,DDX3Y,LINC00280,TTTY23,TTTY15,RBMY1A3P,FAM197Y5P,PCDH11Y,TTTY1,FAM224B,VCY,XKRY,USP9Y,TTTY2,TTTY23B,TTTY11,TSPY10,PRKY,XKRY2,TTTY8B,HSFY2,RBMY3AP,TTTY12,TMSB4Y,SRY,CDY2A,TTTY9B,UTY,TSPY8,GYG2P1,TTTY21B,FAM41AY1,TTTY9A,TBL1Y,TSPY1,TGIF2LY,TTTY7B,TTTY7,TTTY8,TTTY21,TTTY20,RPS4Y1,LINC00278,TTTY16,TSPY3,NLGN4Y-AS1,TSPY4,TTTY1B,TTTY22,VCY1B,AMELY,ZFY,TSPY2,FAM197Y2P,FAM41AY2,TTTY18,FAM224A,HSFY1	0	loss
624	CGTE_09	Y	21154393	24460980	Yq11.223-Yq11.222	KDM5D,PRY2,RBMY2FP,PRY,TTTY5,RBMY1B,RBMY2EP,RBMY1D,LOC101929148,TTTY14,TTTY10,RBMY1E,CD24,TXLNGY,EIF1AY,TTTY6B,RPS4Y2,RBMY1F,TTTY13,RBMY1J,TTTY6,PRORY,BCORP1,RBMY1A1	0	loss
625	CGTE_10	1	3394940	3477362	1p36.32	MIR551A,ARHGEF16,MEGF6	1	loss
626	CGTE_10	1	109810418	109816453	1p13.3	CELSR2	1	loss
627	CGTE_10	1	228459619	228479809	1q42.13	OBSCN	1	loss
628	CGTE_10	1	228479814	228493130	1q42.13	OBSCN	3	gain
629	CGTE_10	2	17877577	17884643	2p24.2	SMC6	0	loss
630	CGTE_10	2	128381736	128398619	2q14.3	LIMS2,MYO7B	1	loss
631	CGTE_10	2	170734973	170751876	2q31.1	UBR3	0	loss
632	CGTE_10	2	201397638	201401012	2q33.1	SGOL2	0	loss
633	CGTE_10	2	219259295	219294410	2q35	MIR26B,SLC11A1,CTDSP1,VIL1	1	loss
634	CGTE_10	2	220353153	220355707	2q35	SPEG	1	loss
635	CGTE_10	2	233404337	233405207	2q37.1	CHRNG	0	loss
636	CGTE_10	3	49688944	49701348	3p21.31	BSN	1	loss
637	CGTE_10	3	52521197	52558572	3p21.1	NISCH,NT5DC2,STAB1	1	loss
638	CGTE_10	3	148777122	148778694	3q24	HLTF	0	loss
639	CGTE_10	4	674262	677539	4p16.3	MFSD7,MYL5	0	loss
640	CGTE_10	4	2260571	2341325	4p16.3	MXD4,ZFYVE28	1	loss
641	CGTE_10	5	466806	488523	5p15.33	PP7080,LOC100288152,EXOC3,SLC9A3	1	loss
642	CGTE_10	5	42762552	42799640	5p12	CCDC152	1	loss
643	CGTE_10	5	61785088	61857112	5q12.1	IPO11	3	gain
644	CGTE_10	5	137780948	137802679	5q31.2	REEP2,EGR1	0	loss
645	CGTE_10	5	172386735	172421971	5q35.1	ATP6V0E1,RPL26L1	1	loss
646	CGTE_10	5	176297414	176309118	5q35.2	HK3,UNC5A	0	loss
647	CGTE_10	6	30860711	30863284	6p21.33	DDR1	0	loss
648	CGTE_10	6	33416529	33419732	6p21.32	SYNGAP1	0	loss
649	CGTE_10	6	33650996	33653610	6p21.31	ITPR3	1	loss
650	CGTE_10	6	126298763	126329578	6q22.32	HINT3,TRMT11	1	loss
651	CGTE_10	7	44146371	44186261	7p13	MIR4649,MYL7,GCK,AEBP1,POLD2	1	loss
652	CGTE_10	7	73008617	73022176	7q11.23	MLXIPL	0	loss
653	CGTE_10	7	75608724	75628597	7q11.23	POR,STYXL1,TMEM120A	1	loss
654	CGTE_10	7	100274997	100285885	7q22.1	GIGYF1,GNB2	0	loss
655	CGTE_10	7	149473040	149523373	7q36.1	SSPO	1	loss
656	CGTE_10	8	143745758	143857184	8q24.3	LOC100288181,PSCA,LYNX1,LYPD2,SLURP1,JRK,LY6K,THEM6	1	loss
657	CGTE_10	9	126128171	126146225	9q33.3	DENND1A,CRB2	1	loss
658	CGTE_10	9	136426137	136439102	9q34.2	ADAMTSL2	0	loss
659	CGTE_10	9	139397600	139414075	9q34.3	MIR4673,NOTCH1	1	loss

660	CGTE_10	9	139905026	139944967	9q34.3	C9orf139,ABCA2,ENTPD2,FUT7,NPDC1	1	loss
661	CGTE_10	9	140322327	140395360	9q34.3	NOXA1,NSMF,ENTPD8,PNPLA7,MIR7114	1	loss
662	CGTE_10	9	141012938	141016420	9q34.3	CACNA1B	0	loss
663	CGTE_10	10	24923943	24959368	10p12.1	ARHGAP21	1	loss
664	CGTE_10	10	45472905	45478088	10q11.21	RASSF4,C10orf10	0	loss
665	CGTE_10	10	59986854	60029367	10q21.1	CISD1,IPMK	0	loss
666	CGTE_10	10	75910873	75960656	10q22.2	ADK	0	loss
667	CGTE_10	10	94659293	94694342	10q23.33	EXOC6	3	gain
668	CGTE_10	10	105349201	105350213	10q24.33	NEURL1	0	loss
669	CGTE_10	10	112327503	112337260	10q25.2	SMC3	1	loss
670	CGTE_10	10	135020334	135088716	10q26.3	ADAM8,MIR202,UTF1,MIR202HG,KNDC1,VENTX	1	loss
671	CGTE_10	10	135137692	135165001	10q26.3	CALY,PRAP1	1	loss
672	CGTE_10	11	1009115	1018646	11p15.5	MUC6,AP2A2	3	gain
673	CGTE_10	11	46395748	46401039	11p11.2	DGKZ,MIR4688	1	loss
674	CGTE_10	12	52306706	52307955	12q13.13	ACVRL1	0	loss
675	CGTE_10	12	76741917	76767237	12q21.2	BBS10,OSBPL8	1	loss
676	CGTE_10	12	88519013	88533398	12q21.32	CEP290	1	loss
677	CGTE_10	12	100536308	100551847	12q23.1	GOLGA2P5,UHRF1BP1L	0	loss
678	CGTE_10	12	122212749	122261630	12q24.31	RHOF,TMEM120B,LINC01089,SETD1B	1	loss
679	CGTE_10	12	124819031	124839034	12q24.31	NCOR2,MIR6880	0	loss
680	CGTE_10	12	132271024	132404706	12q24.33	SFSWAP,ULK1,MMP17	1	loss
681	CGTE_10	13	25404580	25418092	13q12.12	RNF17	0	loss
682	CGTE_10	13	33315169	33333868	13q13.1	PDS5B	1	loss
683	CGTE_10	13	48938921	48953798	13q14.2	RB1	0	loss
684	CGTE_10	14	73717613	73732321	14q24.2	PAPLN	1	loss
685	CGTE_10	14	103523331	103570834	14q32.32	CDC42BPB,EXOC3L4	1	loss
686	CGTE_10	14	105954545	106174506	14q32.33	CRIP1,MIR8071-1,MIR8071-2,TMEM121,C14orf80,ELK2AP	1	loss
687	CGTE_10	15	74336375	74473360	15q24.1	ISLR2,LOC283731,PML,ISLR,GOLGA6A,STRA6	1	loss
688	CGTE_10	15	90610312	90611794	15q26.1	ZNF710	0	loss
689	CGTE_10	16	675947	943180	16p13.3	WFIKKN1,WDR90,GNG13,NARFL,FAM195A,RPUSD1,FBXL16,PRR25,LMF1,MSLN,METRN,CHTF18,JMJD8,WDR24,MIR662,C16orf13,RAB40C,STUB1,CCDC78,RHOT2,HAGHL,FAM173A,RHBDL1	1	loss
690	CGTE_10	16	4747006	4749159	16p13.3	ANKS3	0	loss
691	CGTE_10	16	28506429	28515495	16p11.2	APOBR,IL27	1	loss
692	CGTE_10	16	88763963	88876279	16q24.3	PIEZO1,MIR4722,RNF166,APRT,CDT1,LOC100289580,LOC339059,CTU2	1	loss
693	CGTE_10	17	2597383	2700009	17p13.3	CLUH,MIR1253,RAP1GAP2	1	loss
694	CGTE_10	17	4439181	4451607	17p13.2	MYBBP1A,SPNS2	1	loss
695	CGTE_10	17	37883544	37884350	17q12	ERBB2	0	loss
696	CGTE_10	17	40822923	40825994	17q21.2	PLEKHH3	0	loss
697	CGTE_10	17	60677889	60757647	17q23.2	MRC2,TLK2	1	loss
698	CGTE_10	17	78081316	78090896	17q25.3	GAA	0	loss
699	CGTE_10	18	18588008	18603698	18q11.1	ROCK1	1	loss
700	CGTE_10	20	3180580	3214966	20p13	ITPA,DDRGK1,SLC4A11	1	loss
701	CGTE_10	20	17607987	17610873	20p12.1	RRBP1	0	loss
702	CGTE_10	20	60881681	60994716	20q13.33	LAMA5-AS1,ADRM1,CABLES2,MIR4758,LAMA5,RBBP8NL,RPS21	1	loss
703	CGTE_10	21	38520916	38523254	21q22.13	TTC3	0	loss
704	CGTE_10	21	45743631	45755728	21q22.3	C21orf2,PFKL	1	loss
705	CGTE_10	21	45970688	46117586	21q22.3	KRTAP10-9,KRTAP10-4,KRTAP10-5,KRTAP10-6,KRTAP10-8,KRTAP10-3,TSPEAR,KRTAP12-1,KRTAP10-10,KRTAP10-2,KRTAP12-3,KRTAP10-11,KRTAP12-4,KRTAP10-12,KRTAP12-2,KRTAP10-7	1	loss
706	CGTE_10	21	47531374	47575502	21q22.3	FTCD,COL6A2	1	loss

707	CGTE_10	22	20126954	20231166	22q11.21	RTN4R,LINC00896,ZDHHC8,LOC284865,CCDC188	1	loss
708	CGTE_10	22	24562574	24580780	22q11.23	CABIN1,SUSD2	1	loss
709	CGTE_10	22	28389091	28397538	22q12.1	TTC28-AS1,TTC28	0	loss
710	CGTE_10	22	29115380	29120547	22q12.1	CHEK2	0	loss
711	CGTE_10	22	29706587	29710030	22q12.2	RASL10A,GAS2L1	0	loss
712	CGTE_10	22	31502455	31523136	22q12.2	INPP5J,SELM	1	loss
713	CGTE_10	22	36678647	36689915	22q12.3	MIR6819,MYH9	1	loss
714	CGTE_10	22	38019311	38039242	22q13.1	SH3BP1,LOC101927051,GGA1	1	loss
715	CGTE_10	22	40801000	40816493	22q13.1	MKL1,SGSM3	1	loss
716	CGTE_10	22	50696592	50755844	22q13.33	PLXNB2,MAPK11,DENND6B,MAPK12	1	loss
717	CGTE_10	X	53253865	53265824	Xp11.22	IQSEC2,KDM5C	0	loss
718	CGTE_10	X	96167296	96173642	Xq21.33	DIAPH2	0	loss
719	CGTE_10	X	152864479	152958189	Xq28	SLC6A8,LOC105373383,PNCK,FAM58A,DUSP9	0	loss
720	CGTE_10	X	153005560	153050948	Xq28	PLXNB3,SRPK3,ABCD1	0	loss
721	CGTE_10	X	153576841	153578464	Xq28	FLNA	0	loss
722	CGTE_11	1	1146913	1148902	1p36.33	TNFRSF4	3	gain
723	CGTE_11	1	38259876	38260423	1p34.3	MANEAL	9	gain
724	CGTE_11	2	16082129	16083029	2p24.3	MYCN	5	gain
725	CGTE_11	2	242755629	242757743	2q37.3	NEU4	4	gain
726	CGTE_11	5	122372167	122426343	5q23.2	PPIC,PRDM6	8	gain
727	CGTE_11	5	140202981	140221533	5q31.3	PCDHA5,PCDHA1,PCDHA3,PCDHA6,PCDHA2,PCDHA7,PCDHA4,PCDHA8	3	gain
728	CGTE_11	5	140222703	140230298	5q31.3	PCDHA7,PCDHA8,PCDHA4,PCDHA5,PCDHA9,PCDHA2,PCDHA6,PCDHA3,PCDHA1	3	gain
729	CGTE_11	5	170735324	170813936	5q35.1	MIR3912,TLX3	3	gain
730	CGTE_11	7	139477394	139478387	7q34	TBXAS1,HIPK2	52	gain
731	CGTE_11	8	145001406	145004577	8q24.3	PLEC	6	gain
732	CGTE_11	8	145772472	145773883	8q24.3	ARHGAP39	9	gain
733	CGTE_11	9	138378655	138379511	9q34.3	PPP1R26	0	loss
734	CGTE_11	9	139959192	139959712	9q34.3	SAPCD2	3	gain
735	CGTE_11	9	140167176	140174478	9q34.3	TOR4A,NELFB	7	gain
736	CGTE_11	10	27702744	27793808	10p12.1	RAB18,PTCHD3	4	gain
737	CGTE_11	10	35896546	35929050	10p11.21	GJD4,FZD8	3	gain
738	CGTE_11	10	35929124	35929972	10p11.21	FZD8	6	gain
739	CGTE_11	13	100637323	100638366	13q32.3	ZIC2	1	loss
740	CGTE_11	13	110434503	110438412	13q34	IRS2	0	loss
741	CGTE_11	14	50154724	50160086	14q21.3	POLE2,KLHDC1	12	gain
742	CGTE_11	14	103390059	103394894	14q32.32	AMN	5	gain
743	CGTE_11	15	68119103	68121600	15q23	SKOR1	3	gain
744	CGTE_11	16	1129250	1144174	16p13.3	C1QTNF8,SSTR5	1	loss
745	CGTE_11	16	1828394	1842341	16p13.3	SPSB3,NUBP2,IGFALS	3	gain
746	CGTE_11	16	2286845	2288033	16p13.3	DNASE1L2	9	gain
747	CGTE_11	16	23724343	23766536	16p12.2	CHP2,ERN2	5	gain
748	CGTE_11	16	31439330	31447600	16p11.2	COX6A2,ZNF843	5	gain
749	CGTE_11	17	7491714	7492935	17p13.1	SOX15	7	gain
750	CGTE_11	17	35299458	35306521	17q12	AATF,LHX1	4	gain
751	CGTE_11	19	1067397	1068618	19p13.3	HMHA1	6	gain
752	CGTE_11	19	1082793	1083518	19p13.3	HMHA1	8	gain
753	CGTE_11	19	1108294	1109781	19p13.3	SBNO2	6	gain
754	CGTE_11	19	1226370	1231290	19p13.3	CBARP,STK11	7	gain
755	CGTE_11	19	2247548	2252449	19p13.3	MIR4321,JSRP1,AMH,SF3A2	3	gain
756	CGTE_11	19	3751148	3754411	19p13.3	MIR1268A,APBA3	21	gain

757	CGTE_11	19	17558785	17597792	19p13.11	TMEM221,SLC27A1,NXNL1	6	gain
758	CGTE_11	19	18259953	18272244	19p13.11	MAST3,PIK3R2	9	gain
759	CGTE_11	19	18901558	18902255	19p13.11	COMP	4	gain
760	CGTE_11	19	19369459	19372355	19p13.11	HAPLN4	8	gain
761	CGTE_11	19	36430225	36435600	19q13.12	LRFN3	8	gain
762	CGTE_11	19	41040273	41060516	19q13.2	SPTBN4	6	gain
763	CGTE_11	19	46268871	46274431	19q13.32	DMPK,SIX5	5	gain
764	CGTE_11	19	48673389	48686003	19q13.33	C19orf68,LIG1	4	gain
765	CGTE_11	19	48994271	48997433	19q13.33	LMTK3	1	loss
766	CGTE_11	19	51051793	51126134	19q13.33	SNAR-F,SYT3,LRRC4B	8	gain
767	CGTE_11	19	56113984	56134055	19q13.42	ZNF784,ZNF524,ZNF865	0	loss
768	CGTE_11	22	19511121	19702380	22q11.21	LINC00895,CLDN5,SEPT5	6	gain
769	CGTE_11	22	21983160	21984303	22q11.21	YDJC	5	gain
770	CGTE_11	X	152686435	152710636	Xq28	ZFP92,TREX2	6	gain
771	CGTE_12	1	3412520	3418554	1p36.32	MEGF6	0	loss
772	CGTE_12	1	67895734	68152306	1p31.3	SERBP1,GADD45A	11	gain

773	CGTE_12	1	157551301	185276311	1q24.1-q25.2	ANKRD45,OR10K1,XCL2,FCGR3A,TIPRL,PRRC2C,MPC2,ZNF648,CD1D,MR1,HSPA7,FCRL3,RABGAP1L,PRDX6,OR6P1,EDEM3,CFAP126,SNORD81,GORAB,PFDN2,FAM20B,CCDC190,IFI16,LOC100147773,GPA33,OR6K2,SMG7,RFWD2,CENPL,TSTD1,LOC101928696,SLAMF7,METTL18,DUSP12,SFT2D2,XPR1,CFAP45,DDR2,FAM163A,ALDH9A1,MYOC,TEDDM1,OR6K6,MIR3121,PAPPA2,COPA,FMO6P,TNFSF4,LINC01363,FLJ23867,HSPA6,SH2D1B,USF1,TOP1P1,SPTA1,DEDD,C1orf226,SCYL3,UAP1,ABL2,SNORD47,ACKR1,NMNAT2,LHX4,DNM3OS,MIR557,KCNJ9,RNASEL,LINC00970,KLHL20,OVAAL,AXDND1,CD1A,FCRL4,MIR199A2,LOC729867,CD84,SMG7-AS1,SUCO,XCL1,LOC101928404,OR10R2,PPOX,PBX1,METTL13,LOC646268,MPZL1,MROH9,BRINP2,GPR52,FCRL6,MIR3119-2,APOBEC4,MIR3120,SNORD75,LOC100505918,KIAA0040,MIR1295A,LOC100422212,SWT1,NME7,CACNA1E,NR1I3,RGS8,HSD17B7,KIAA1614,NCSTN,PVRL4,TOR3A,FCRL1,LMX1A,SNORD78,CD247,NHLH1,KLHDC9,BLZF1,OLFML2B,OR10J5,SLAMF9,LRRC52,SNORA103,TSEN15,GLUL,SNORD79,SNORD77,FMO2,OR6K3,CD1C,PIGM,LAMC1,PEX19,MAEL,KIFAP3,LOC101928650,LOC100505795,IER5,LOC101928565,SELP,APOA2,FCGR3B,LINC00626,SNORD80,SLC19A2,SLAMF1,OR10X1,MIR4424,DNM3-IT1,DPT,VSIG8,LOC101928673,IVNS1ABP,B4GALT3,C1orf220,TMCO1,CD1B,LY9,SNORD44,CCDC181,CEP350,TEX35,MIR1255B2,NPHS2,VAMP4,F5,ATP1B1,SLC9C2,MPZ,SLAMF6,OR6N2,ASTN1,ITLN1,MIR3119-1,MIR556,USP21,PIGC,ARPC5,C1orf204,CADM3,ADCY10,LOC101928778,TNN,C1orf111,PRRX1,RXRG,CD5L,PCP4L1,POGK,CREG1,LOC284648,FCRL2,GPR161,METTL11B,UCK2,LOC100506023,RCSD1,FCRLB,TOR1AIP1,FMO1,NOS1AP,LAMC2,OR10T2,SELE,SNORD76,RASAL2,RGSL1,TDRD5,SOAT1,TNFSF18,FASLG,CD1E,MGST3,NUF2,TRMT1L,TNR,SUMO1P3,RGL1,NCF2,LINC00272,ATF6,LOC730159,RNF2,TADA1,OR10K2,OR6Y1,LINC01142,CD48,SDHC,IGSF9,RC3H1,LOC102724601,QSOX1,SCARNA3,FCGR2B,LINC01133,DCAF6,ADAMTS4,MIR4654,PEA15,SELL,DCAF8,F11R,DHX9,FAM78B,MRPS14,LHX4-AS1,NIT1,GAS5,VANGL2,C1orf21,OR10J3,LINC01344,LOC101928973,MIR921,LOC101928751,ARHGAP30,FCGR2A,LOC730102,NDUFS2,MIR3658,ACBD6,RGS5,FCGR2C,SNORD74,LOC101928372,KIRREL,GAS5-AS1,IGSF8,DUSP23,DUSP27,CADM3-AS1,MIR488,SERPINC1,TBX19,ZBTB37,MNDA,ILDR2,OR6N1,LOC440700,PYHIN1,C1orf105,ATP1A4,KCNJ10,RPL31P11,FCER1G,SHCBP1L,MIR5187,LOC102724661,TOR1AIP2,COLGALT2,RALGPS2,FMO9P,OR10Z1,STX6,UFC1,CASQ1,ATP1A2,NPL,FMO3,FCRLA,POU2F1,FCER1A,SLAMF8,DARS2,TOMM40L,CACYBP,CRP,CD244,OR10J1,APCS,ANKRD36BP1,SEC16B,RGS16,ANGPTL1,MIR1295B,FMO4,RGS4,RASAL2-AS1,DNM3,MIR4259,C1orf112,ITLN2,AIM2,TAGLN2,LOC400794,FAM129A,UHMK1,GM140,MIR214	3	gain
774	CGTE_12	3	62860588	63264394	3p14.2	LINC00698,CADPS,SYNPR	10	gain

775	CGTE_12	3	136646796	178546205	3q23-q26.31	DHX36,PISRT1,CCNL1,IL12A,MBNL1,SOX14,MED12L,IFT80,MIR569,TM4SF4,NAALADL2-AS3,SLC33A1,GHSR,GPR160,MIR4789,SIAH2,LINC01327,GRK7,MIR551B,P2RY1,LINC00886,RARRES1,PRKCI,SI,AADAC,MECOM,TRIM42,NMNAT3,AGTR1,SHOX2,LOC100128164,ECT2,SLC7A14,MBNL1-AS1,IL20RB,SKIL,KCNMB2,LOC100507537,MME,ERICH6-AS1,PAQR9,MIR548AY,LINC01322,PLD1,KCNAB1,B3GALNT1,FOXL2,EIF2A,ZBBX,LOC101243545,SAMD7,SLC2A2,PFN2,TIPARP,TERC,ZIC1,CP,LINC00881,NCEH1,SPSB4,SUCNR1,RAP2B,CPB1,LOC100507661,LINC00880,ARHGEF26,BCHE,NAALADL2,CHST2,RBP2,PLS1,TFDP2,LRRC31,TM4SF1-AS1,TRPC1,DBR1,RNF13,BPESC1,SSR3,C3orf80,PLSCR2,ARMC8,CLSTN2-AS1,AADACL2,A4GNT,LINC01192,EIF5A2,MIR548H2,LINC01100,GPR149,MIR1263,FNDC3B,SPTSSB,CPA3,ESYT3,LINC01014,PQLC2L,IQCJ-SCHIP1,RSRC1,NCK1,ANKUB1,CLRN1,PTX3,MIR6828,NLGN1-AS1,ERICH6,SCHIP1,ATP1B3,KPNA4,NLGN1,LINC01210,LRRC34,PXYLP1,IQCJ,SERPINI1,C3orf33,LOC101928105,LXN,NME9,DZIP1L,PAQR9-AS1,P2RY12,MIR3919,PPM1L,WWTR1,LINC01487,MIR15B,ZIC4,RASA2,TRIM59,LINC01208,TNFSF10,ZBTB38,XRN1,LOC440982,LOC100289361,WWTR1-AS1,TMEM14EP,MRPS22,GMPS,LINC01209,SEC62,MFSD1,SLITRK3,SCARNA7,GPR87,NAALADL2-AS2,RBP1,TBL1XR1,GYG1,U2SURP,LOC100507291,IL12A-AS1,CLDN18,COPB2,PDCD10,NAALADL2-AS1,ACTRT3,TIPARP-AS1,MRAS,TM4SF18,LINC01213,LOC100507389,LINC00501,SMC4,SPATA16,OTOL1,ARL14,SERPINI2,FAIM,PLSCR4,IGSF10,KCNAB1-AS2,AADACP1,MIR16-2,TMEM212-AS1,MYNN,TM4SF1,MLF1,GOLIM4,LOC646903,CLDN11,PRR23A,TSC22D2,PA2G4P4,SLC9A9,GPR171,PLSCR5,ARHGEF26-AS1,P2RY13,LOC100996447,HPS3,KCCAT211,MIR7977,LRRIQ4,HLTF,COMMD2,MIR5186,P2RY14,GFM1,PLOD2,RPL22L1,C3orf79,EGFEM1P,WDR49,SERP1,LINC01324,NMD3,CLSTN2,LINC01330,GK5,HLTF-AS1,PRR23C,ATR,IQCJ-SCHIP1-AS1,SELT,C3orf58,AADACL2-AS1,PHC3,RNF7,SLC25A36,PLCH1,CLRN1-AS1,VEPH1,KCNMB2-AS1,TMEM212,LEKR1,CEP70,SLC9A9-AS1,LINC01214,PIK3CB,KCNAB1-AS1,PLSCR1,PRR23B,TNIK,LINC00578,FOXL2NB,LINC01391,PCOLCE2	3	gain
776	CGTE_12	3	187003559	194140651	3q29-q28	HRASLS,BCL6,ATP13A4,FLJ42393,LOC647323,LOC100131635,SST,CPN2,MASP1,FGF12-AS1,ATP13A5,MIR944,LPP-AS1,GP5,HES1,OSTN,LINC00887,RTP4,TMEM207,UTS2B,IL1RAP,LOC100505920,TPRG1,CCDC50,TP63,P3H2,SNAR-I,LOC101929337,MB21D2,ATP13A5-AS1,OSTN-AS1,TPRG1-AS1,RTP2,LPP-AS2,P3H2-AS1,CLDN16,MGC2889,DPPA2P3,GMNC,ATP13A4-AS1,MIR28,PYDC2,LRRC15,TPRG1-AS2,OPA1-AS1,FGF12,ATP13A3,LPP,LINCR-0002,CLDN1,OPA1	3	gain
777	CGTE_12	4	1102015	1195036	4p16.3	RNF212,TMED11P,SPON2,LOC100130872	7	gain
778	CGTE_12	5	140215343	140216026	5q31.3	PCDHA3,PCDHA5,PCDHA4,PCDHA6,PCDHA2,PCDHA1,PCDHA7	7	gain
779	CGTE_12	5	179563362	179565001	5q35.3	RASGEF1C	7	gain
780	CGTE_12	6	30523318	30524037	6p21.33	GNL1	8	gain
781	CGTE_12	6	31865474	31868347	6p21.33	EHMT2,C2,ZBTB12	8	gain
782	CGTE_12	6	43227286	43253116	6p21.1	TTBK1	0	loss
783	CGTE_12	6	82461282	82462509	6q14.1	FAM46A	5	gain
784	CGTE_12	6	114281175	114292522	6q21	LOC101927768,HDAC2	8	gain

785	CGTE_12	7	7459384	20198035	7p21.2-p21.1	SOSTDC1,TMEM196,ISPD,UMAD1,NXPH1,LRRC72,MIOS,MEOX2-AS1,AGR3,LOC100505921,KCCAT333,LOC101927630,ANKMY2,LOC101927391,SNX13,TMEM106B,HDAC9,MACC1,SCIN,MACC1-AS1,ICA1,AGMO,NDUFA4,TWISTNB,MIR1302-6,TWIST1,PER4,GLCCI1,PRPS1L1,RPA3,VWDE,LOC101927668,BZW2,ARL4A,TSPAN13,ISPD-AS1,ETV1,MEOX2,LOC100505938,AGR2,THSD7A,COL28A1,PHF14,AHR,FERD3L,MIR3146,DGKB	3	gain
786	CGTE_12	8	49643795	121457931	8q13.1-q11.22	MIR3610,LOC102724804,GEM,MTBP,RRM2B,LOC101929528,CRH,TNFRSF11B,NACAP1,UTP23,ASPH,CSPP1,ERICH5,LOC101927066,UBR5-AS1,MSC,SNX16,MIR3151,MATN2,LOC102724710,MSC-AS1,SNHG6,MRPL15,RGS20,OPRK1,HRSP12,LINC01609,EBAG9,LRRCC1,SBF1P1,PLAG1,SDCBP,EYA1,LOC101929709,RAB2A,LINC00536,CA3-AS1,VPS13B,LOC286177,BAALC-AS2,RB1CC1,SDC2,GRHL2,POLR2K,MED30,TRIM55,MIR5681A,PREX2,AZIN1,MIR8084,C8orf87,TSPYL5,FLJ46284,MCMDC2,CYP7A1,SLC30A8,MIR124-2HG,RIPK2,EMC2,EIF3H,STK3,PKHD1L1,C8orf46,MIR875,SULF1,LINC00968,MIR3150B,C8orf59,TRPS1,ZBTB10,CSMD3,MIR5708,MIR1273A,LOC102724612,SNX31,NECAB1,DEPTOR,C8orf44-SGK3,LINC01608,RRS1-AS1,ADHFE1,TRPA1,LYPLA1,LINC00588,TCF24,LINC01592,MTFR1,LOC100130298,PRDM14,CNBD1,TRIQK,ZC2HC1A,MIR4471,RPS20,MIR599,DECR1,SOX17,RAD21,FABP12,MTDH,MIR4661,CHD7,C8orf34,C8orf88,NKAIN3,OTUD6B,BAALC-AS1,KLF10,IL7,LINC01607,TP53INP1,RNF19A,LINC00967,FSBP,UQCRB,PLEKHF2,PTDSS1,LINC01603,MRPS28,POP1,MMP16,PI15,LINC01299,SPAG1,COLEC10,STMN2,YWHAZ,RMDN1,ARFGEF1,IMPA1,ZNF704,LINC01301,MIR2053,PKIA,SNTG1,LOC101241902,CPA6,FAM92A1,FZD6,SNAI2,DPYS,TRAM1,OTUD6B-AS1,LOC100288748,PDP1,PDE7A,TOX,OXR1,RAD54B,SLC10A5,YTHDF3-AS1,SAMD12-AS1,LYN,MIR7641-2,DCAF13,NIPAL2,LOC104054148,LOC102724623,UBR5,MYBL1,ODF1,MIR3149,FABP4,PEX2,OSR2,MOS,CNGB3,SNORA72,CA13,C8orf44,TMEM68,SLCO5A1,CA1,KIAA1429,PCMTD1,NSMAF,UBXN2B,LINC00251,SDR16C6P,LOC105375650,MIR7705,MIR378D2,LOC101926892,TTPA,GDF6,MIR124-2,ENY2,TMEM70,C8orf37,ARMC1,C8orf89,LRRC69,FBXO43,MIR4470,SDR16C5,MIR5680,CLVS1,LINC00535,STAU2-AS1,PPP1R42,CHCHD7,BHLHE22,TAF2,SYBU,ENPP2,RP1,WWP1,LY96,RBM12B-AS1,KCNS2,NBN,EXT1,LINC01606,NPBWR1,TMEM74,PABPC1,IMPAD1,CYP7B1,CTHRC1,CASC9,MIR2052HG,SBSPON,PAG1,PXDNL,SGK3,TPD52,ATP6V1H,NCOA2,JPH1,LOC101929415,CHMP4C,MIR5681B,BAALC,INTS8,ZFAND1,XKR9,TCEA1,E2F5,TRHR,PMP2,DPY19L4,TERF1,LACTB2-AS1,ST18,ZFPM2-AS1,YTHDF3,RPL30,LRP12,MIR2052,TGS1,C8orf22,RRS1,SNORD87,CRISPLD1,LINC01298,RUNX1T1,HNF4G,CPNE3,CA2,RALYL,MTERF3,ANKRD46,LACTB2,LINC01289,ATP6V0D2,SLC25A32,RPL7,LINC01030,LOC392232,KCNB2,LOC102724874,CCNE2,LOC100500773,COX6C,ESRP1,DSCC1,FABP5,LINC00534,TMEM55A,GGH,VCPIP1,PKIA-AS1,RDH10-AS1,LINC01111,NDUFAF6,ZNF706,TMEM64,LOC101927040,LINC01419,ATP6V1C1,SLC26A7,SNORD54,EIF3E,REXO1L2P,KCNV1,EFCAB1,MAL2,ANGPT1,FABP9,RBM12B,RDH10,PENK,CALB1,CA8,CDH17,RGS22,RSPO2,LOC101926908,NOV,GDAP1,HEY1,UBE2W,ZFHX4,ZFPM2,LOC101929488,FLJ42969,RIMS2,CPQ,UG0898H09,SLC7A13,LOC401463,MRPL13,LAPTM4B,AARD,C8orf37-AS1,XKR4,LINC01602,C8orf34-AS1,AZIN1-AS1,DNAJC5B,SAMD12,PSKH2,NUDCD1,MIR3150A,ZFHX4-AS1,FAM110B,COL14A1,CA3,TMEM67,STAU2,COPS5,DCAF4L2,NCALD,RAD21-AS1,DCSTAMP,FAM150A,ABRA,TCEB1,OSGIN2,PTTG3P	3	gain

787	CGTE_12	8	143614698	143623738	8q24.3	ADGRB1	7	gain
788	CGTE_12	8	144891710	144893575	8q24.3	SCRIB	24	gain
789	CGTE_12	8	145018574	145020716	8q24.3	MIR661,PLEC	24	gain
790	CGTE_12	9	15307013	15423296	9p22.3	SNAPC3,TTC39B	13	gain
791	CGTE_12	9	98270334	98638481	9q22.32	LINC00476,ERCC6L2,PTCH1	9	gain
792	CGTE_12	9	127253279	127262533	9q33.3	NR5A1	5	gain
793	CGTE_12	9	138590665	138594223	9q34.3	SOHLH1,KCNT1	6	gain
794	CGTE_12	9	139234135	139244172	9q34.3	GPSM1	7	gain
795	CGTE_12	9	139378808	139391116	9q34.3	C9orf163,NOTCH1	6	gain
796	CGTE_12	9	139391120	139440752	9q34.3	MIR4674,MIR4673,NOTCH1	1	loss
797	CGTE_12	9	139562660	139564157	9q34.3	EGFL7	7	gain
798	CGTE_12	9	139731962	139733814	9q34.3	RABL6	7	gain
799	CGTE_12	9	139840110	139864132	9q34.3	LCN12,C8G	4	gain
800	CGTE_12	9	139959586	139961016	9q34.3	SAPCD2	6	gain
801	CGTE_12	9	140001694	140006652	9q34.3	DPP7,MAN1B1	4	gain
802	CGTE_12	9	140201373	140246688	9q34.3	EXD3	3	gain
803	CGTE_12	10	78637705	88024640	10q23.2-q22.3	NRG3,MBL1P,ANXA11,LINC00856,LOC102723703,LOC101929646,SH2D4B,DYDC1,C10orf99,DLG5,LOC101929574,TMEM254,ZMIZ1-AS1,NUTM2B,CDHR1,KCNMA1-AS2,KCNMA1-AS3,DYDC2,LINC01519,POLR3A,ZCCHC24,FAM213A,LRIT1,PLAC9,SFTPD,BMS1P21,LINC00858,GRID1-AS1,NRG3-AS1,ZMIZ1,LRIT2,DLG5-AS1,PPIF,MAT1A,LINC00857,KCNMA1,LINC01520,CCSER2,LINC00595,RPS24,RGR,KCNMA1-AS1,SFTPA2,MIR346,TMEM254-AS1,LOC642361,BEND3P3,SFTPA1,LOC101929662,NUTM2B-AS1,GHITM,EIF5AL1,TSPAN14,GRID1	3	gain
804	CGTE_12	11	289838	1587056	11p15.5	PANO1,SLC25A22,IFITM5,SIGIRR,LOC143666,DUSP8,LRRC56,CRACR2B,SCT,MOB2,B4GALNT4,MIR6744,IFITM3,POLR2L,PNPLA2,CHID1,MIR210HG,DEAF1,PKP3,MUC6,MUC5AC,CD151,CDHR5,HRAS,TOLLIP-AS1,ATHL1,MUC5B,MUC2,IFITM1,PHRF1,RNH1,TMEM80,MIR210,ANO9,DRD4,IFITM2,TALDO1,PDDC1,BRSK2,RPLP2,LMNTD2,PTDSS2,TSPAN4,LOC171391,EPS8L2,TOLLIP,PIDD1,AP2A2,IRF7,SNORA52,CEND1,RASSF7	1	loss
805	CGTE_12	11	1587059	1629695	11p15.5	KRTAP5-AS1,DUSP8,KRTAP5-3,KRTAP5-2,KRTAP5-1	6	gain
806	CGTE_12	11	1630291	3109561	11p15.5-p15.4	SYT8,MRPL23-AS1,TH,MRPL23,CD81-AS1,KRTAP5-6,TNNI2,C11orf21,OSBPL5,IGF2-AS,SNORD131,TRPM5,MIR4686,LSP1,KCNQ1OT1,FAM99A,KRTAP5-4,CTSD,HOTS,KCNQ1,CD81,LINC01219,TNNT3,MIR675,INS,KRTAP5-5,IFITM10,MIR7847,IGF2,KCNQ1-AS1,SNORA54,CDKN1C,LINC01150,TSPAN32,MIR4298,NAP1L4,KCNQ1DN,H19,MIR483,FAM99B,PHLDA2,CARS,TSSC4,SLC22A18AS,INS-IGF2,SLC22A18,ASCL2	1	loss
807	CGTE_12	11	3111007	3115132	11p15.4	OSBPL5	5	gain
808	CGTE_12	11	11454168	11990092	11p15.3	MIR8070,MIR4299,DKK3,USP47,GALNT18	4	gain
809	CGTE_12	11	12020088	14536085	11p15.2-p15.3	MIR6124,PARVA,ARNTL,BTBD10,COPB1,MICAL2,TEAD1,DKK3,FAR1,RRAS2,PSMA1,SPON1,MICALCL,PTH,LINC00958,RASSF10	3	gain
810	CGTE_12	11	14536106	15262189	11p15.2	INSC,CYP2R1,CALCA,PDE3B,PSMA1,CALCB	4	gain

811	CGTE_12	11	19138608	35757193	11p15.1-p13	LINC00294,CSTF3-AS1,EIF3M,MPPED2,TRIM44,FBXO3-AS1,PAX6,LINC00678,KIF18A,FBXO3,GAS2,EHF,LOC100507144,CCDC179,CCDC34,NAV2-AS5,APIP,FJX1,FANCF,LOC105376671,SLC6A5,BBOX1,MIR8087,E2F8,MIR4486,LUZP2,NAV2-AS4,SLC1A2,ANO5,MIR8068,PRMT3,SLC17A6,ELP4,ARL14EP,LOC100126784,MIR1343,ABTB2,FIBIN,HIPK3,CAT,ELF5,FSHB,METTL15,SLC5A12,DNAJC24,BBOX1-AS1,LGR4,DKFZp686K1684,WT1-AS,NAT10,PDHX,ZDHHC13,CSTF3,DEPDC7,CD44,CAPRIN1,MUC15,BDNF,CSRP3,DCDC1,DCDC5,KCNA4,ANO3,LINC01495,MIR8054,SVIP,BDNF-AS,LINC01616,QSER1,LOC105376599,LMO2,NAV2-AS2,TCP11L1,RCN1,CCDC73,SNORA88,LIN7C,PAUPAR,C11orf91,IMMP1L,LOC100506675,KIAA1549L,PAMR1,DBX1,HTATIP2,MIR4694,WT1,MIR610,CD59,PRRG4,NAV2,NELL1	3	gain
812	CGTE_12	12	6857915	6858310	12p13.31	MLF2	26	gain
813	CGTE_12	12	48577920	48723818	12q13.11	CCDC184,H1FNT,OR10AD1	11	gain
814	CGTE_12	13	88329233	94197714	13q31.2-q31.3	LOC105370306,MIR18A,LINC00380,LINC01049,LINC01047,GPC5-AS2,LINC00433,MIR17,MIR622,LINC00559,GPC5,LINC00440,LINC00353,LINC00397,LINC00363,LINC01040,GPC6,MIR20A,LINC00379,GPC5-AS1,SLITRK5,LINC00410,MIR17HG,MIR92A1,MIR19A,MIR19B1	3	gain
815	CGTE_12	13	111358388	111371904	13q34	CARS2,ING1	15	gain
816	CGTE_12	14	21558686	21561535	14q11.2	ZNF219	11	gain
817	CGTE_12	14	37051248	37135970	14q13.3	PAX9,NKX2-8	8	gain
818	CGTE_12	14	100615263	100705719	14q32.2	DEGS2,YY1	24	gain
819	CGTE_12	15	62352431	62360727	15q22.2	VPS13C,C2CD4A	13	gain
820	CGTE_12	15	90171648	90190438	15q26.1	KIF7	4	gain
821	CGTE_12	16	1116328	1131192	16p13.3	SSTR5-AS1,SSTR5	6	gain
822	CGTE_12	16	2027949	2030830	16p13.3	NOXO1,TBL3	14	gain
823	CGTE_12	16	2051485	2083637	16p13.3	SLC9A3R2,NPW,ZNF598	1	loss
824	CGTE_12	16	75147458	75150630	16q23.1	LDHD	4	gain
825	CGTE_12	17	21117437	21188384	17p11.2	TMEM11,MAP2K3,NATD1	5	gain
826	CGTE_12	17	38816267	39597163	17q21.2	KRTAP1-5,KRTAP4-6,KRTAP9-7,KRTAP1-4,KRT222,KRTAP9-8,KRTAP9-2,KRTAP4-4,KRTAP2-1,KRT40,KRTAP4-5,KRT28,KRTAP4-7,KRTAP9-4,KRT24,KRTAP4-8,KRTAP9-6,KRT12,KRT33B,KRT23,KRTAP16-1,KRTAP4-9,TMEM99,KRT39,KRTAP2-3,KRTAP1-3,KRT10,KRTAP9-1,KRT31,KRT33A,KRTAP1-1,KRT34,KRT25,LOC100505782,KRTAP9-3,KRT38,KRTAP17-1,KRTAP2-4,KRTAP3-3,KRTAP4-12,KRT37,KRT20,KRTAP9-9,KRTAP29-1,KRT26,KRTAP4-2,KRTAP2-2,KRTAP3-1,KRT27,KRTAP4-3,KRTAP4-1,KRTAP3-2,KRTAP4-11	3	gain
827	CGTE_12	17	78882374	79203329	17q25.3	MIR657,BAIAP2,RPTOR,AATK-AS1,CEP131,CHMP6,ENTHD2,AATK,BAIAP2-AS1,MIR338,MIR3065,MIR1250	1	loss
828	CGTE_12	17	79204352	79207918	17q25.3	ENTHD2	6	gain
829	CGTE_12	17	80391656	80398251	17q25.3	HEXDC	8	gain

830	CGTE_12	18	907564	11610055	18p11.32-p11.22	MIR4317,EPB41L3,METTL4,SLC35G4,MYOM1,LINC00470,RALBP1,LINC01387,GAPLINC,ADCYAP1,DLGAP1-AS3,VAPA,ANKRD12,LINC01254,PIEZO2,LAMA1,MIR3976,ZBTB14,MIR6718,NDC80,LPIN2,PPP4R1-AS1,LOC101927410,MYL12A,TXNDC2,L3MBTL4,APCDD1,MTCL1,NDUFV2,RAB31,LINC00668,L3MBTL4-AS1,TMEM200C,TWSG1,ARHGAP28,NAPG,PTPRM,MYL12B,LOC727896,DLGAP1-AS5,DLGAP1-AS4,GACAT2,PPP4R1,LOC100192426,DLGAP1-AS2,LINC00526,C18orf42,CBX3P2,MIR3976HG,SMCHD1,LOC101927188,TGIF1,NDUFV2-AS1,DLGAP1,DLGAP1-AS1,LOC104968399,LINC00667,MIR6788,RAB12,LINC01255,EMILIN2,LRRC30	3	gain
831	CGTE_12	18	30349416	30351094	18q12.1	KLHL14	5	gain
832	CGTE_12	18	51750376	51796246	18q21.2	MBD2,POLI	9	gain
833	CGTE_12	18	74962516	77474626	18q23	ATP9B,SALL3,LINC01029,GALR1,CTDP1,NFATC1	1	loss
834	CGTE_12	19	281411	1531891	19p13.3	PLPPR3,NDUFS7,PLK5,MIR3187,RNF126,C2CD4C,FSTL3,CIRBP,MIR4745,ADAMTSL5,PCSK4,PALM,HCN2,ELANE,POLRMT,WDR18,STK11,CIRBP-AS1,EFNA2,MED16,GZMM,BSG,MISP,HMHA1,GPX4,MADCAM1,C19orf25,RPS15,TPGS1,ARID3A,ABCA7,ATP5D,TMEM259,PTBP1,SBNO2,GAMT,CDC34,REEP6,PRSS57,PRTN3,GRIN3B,MIER2,POLR2E,PLPP2,APC2,AZU1,CBARP,MUM1,MIDN,FGF22,DAZAP1,SHC2,KISS1R,CFD,R3HDM4,CNN2,RNU6-2,C19orf24,THEG,ODF3L2	1	loss
835	CGTE_12	19	1533914	1578600	19p13.3	MBD3,PLK5,MEX3D	7	gain
836	CGTE_12	19	1581047	3633273	19p13.3	OAZ1,S1PR4,UQCR11,DIRAS1,SCAMP4,AP3D1,JSRP1,ZNF77,ONECUT3,LINGO3,ZNF554,LMNB2,KLF16,ADAT3,TBXA2R,C19orf35,MIR1227,CELF5,CSNK1G2-AS1,DOHH,TLE6,C19orf71,CACTIN-AS1,GIPC3,TIMM13,GNA11,ZNF556,FZR1,NFIC,ATP8B3,THOP1,DOT1L,ABHD17A,MIR7108,LOC100996351,MIR1909,REXO1,LSM7,ZNF555,MIR7850,AMH,AES,MBD3,ZNF57,PLEKHJ1,MIR6789,GNA15,LOC100288123,PIP5K1C,MOB3A,BTBD2,SGTA,SF3A2,TCF3,GNG7,MKNK2,IZUMO4,HMG20B,SLC39A3,SPPL2B,CACTIN,CSNK1G2,TMPRSS9,NCLN,TLE2,SMIM24,MIR4321,GADD45B,MFSD12	1	loss
837	CGTE_12	19	29567062	32102296	19q12	VSTM2B,TSHZ3,URI1,POP4,THEG5,C19orf12,LOC284395,PLEKHF1,CCNE1,ZNF536,UQCRFS1	7	gain
838	CGTE_12	19	50364545	50365979	19q13.33	PNKP	7	gain
839	CGTE_12	20	23420929	23860361	20p11.21	CST3,CST5,CST2,CST9L,CST9,CST13P,CSTL1,CST8,CST1,CST11,CST4	6	gain

840	CGTE_12	20	31669328	48130899	20q13.12-q13.11	LBP,NNAT,SNORA71E,MAFB,MIR499A,CBFA2T2,DNTTIP1,ROMO1,PREX1,SPATA25,WFDC8,ACSS2,LINC00489,WFDC9,TOP1,ZNFX1,YWHAB,LOC149684,PTGIS,NCOA5,SNORA71A,ARHGAP40,SLA2,SNTA1,SPAG4,SNHG17,TNNC2,BPI,TOX2,PHF20,STK4-AS1,ADA,LOC101927159,ACOT8,WFDC10A,TTI1,MMP24,PI3,SRSF6,AAR2,LINC01370,SEMG1,BPIFA4P,C20orf144,DDX27,SULF2,ZNF341-AS1,MIR644A,ZNF663P,MIR6812,CDK5RAP1,PLCG1-AS1,EYA2,LINC01620,CNBD2,MIR3646,MIR3617,ERGIC3,SLC2A10,RIMS4,JPH2,KCNS1,SDC4,EPPIN-WFDC6,MIR4755,LINC00494,C20orf24,SERINC3,CPNE1,OCSTAMP,ADIG,LOC339568,EDEM2,GTSF1L,EIF2S2,TLDC2,PIGU,SNHG11,PPP1R16B,TGIF2,MYH7B,RALGAPB,ZHX3,PROCR,SAMHD1,WFDC2,FITM2,EIF6,MIR3616,HMGB3P1,BPIFB1,BPIFB4,PKIG,RPN2,ITCH,TRPC4AP,ZNF341,SYS1-DBNDD2,SLC32A1,PIGT,AHCY,MATN4,DLGAP4-AS1,DSN1,GHRH,WFDC13,BPIFA3,DBNDD2,MROH8,E2F1,ZSWIM1,SNORD12B,SNORA71B,TP53TG5,LINC01260,KCNK15-AS1,DHX35,SYS1,TOMM34,LINC00657,SNORD12,OSER1,WFDC3,EMILIN3,FAM83C-AS1,WFDC11,LINC01430,SPINT3,ACTL10,LOC100287792,SNORA60,MYBL2,MMP24-AS1,ARFGEF2,RBM12,NDRG3,UBE2C,WISP2,KIAA1755,MYL9,L3MBTL1,MKRN7P,SGK2,ZFAS1,SNORA71C,SLPI,NEURL2,NCOA6,LINC01523,SLC13A3,GGT7,EPPIN,MMP9,PABPC1L,STAU1,SCAND1,ELMO2,NFS1,CEP250,ASIP,SOGA1,OSER1-AS1,SLC35C2,MANBAL,RBL1,KCNB1,ACTR5,ZNF334,SRC,FAM83C,WFDC6,NECAB3,IFT52,PXMP4,WFDC5,SLC12A5,MIR1289-1,BLCAP,C20orf173,STK4,LINC01522,GDAP1L1,R3HDML,EPB41L1,BPIFA2,GSS,ZMYND8,TP53INP2,LPIN3,PLCG1,PCIF1,SNORA71D,MIR499B,TP53RK,WFDC12,CHMP4B,PTPRT,LOC100131496,CD40,SPINT4,CSE1L,FER1L4,UQCC1,RBPJL,KCNK15,MAP1LC3A,CHD6,FAM83D,RALY-AS1,DYNLRB1,RALY,NCOA3,DLGAP4,TGIF2-C20orf24,GDF5,SNORD12C,CTNNBL1,TTPAL,CSE1L-AS1,BPIFA1,VSTM2L,TGM2,SNX21,SEMG2,PLTP,CTSA,WFDC10B,HNF4A-AS1,RBM39,ZSWIM3,RPRD1B,MIR6871,HNF4A,CDH22,ZNF335	3	gain
841	CGTE_12	20	48140633	48767984	20q13.13	TMEM189,UBE2V1,SLC9A8,TRERNA1,SNAI1,TMEM189-UBE2V1,PTGIS,SPATA2,RNF114,B4GALT5	6	gain
842	CGTE_12	20	49177770	49626079	20q13.13	ADNP,LOC100506175,BCAS4,KCNG1,PARD6B,DPM1,MIR1302-5,MIR645,MOCS3,PTPN1,ADNP-AS1,FAM65C	5	gain
843	CGTE_12	20	49626083	53267141	20q13.13-q13.2	MIR3194,ZFP64,DOK5,BCAS1,ZNF217,ATP9A,MIR4756,TSHZ2,CYP24A1,KCNG1,SUMO1P1,LINC01429,PFDN4,SALL4,LINC01524,NFATC2,LOC101927770	6	gain
844	CGTE_12	21	30445738	32253929	21q21.3-q22.11	KRTAP13-1,KRTAP21-1,KRTAP19-7,KRTAP6-2,KRTAP13-2,KRTAP13-3,KRTAP22-1,GRIK1-AS1,KRTAP19-4,KRTAP19-5,KRTAP21-2,KRTAP20-1,KRTAP25-1,KRTAP8-1,KRTAP13-4,GRIK1-AS2,CLDN8,KRTAP20-4,KRTAP7-1,BACH1-IT2,KRTAP19-6,KRTAP6-3,KRTAP19-1,MIR4327,KRTAP21-3,KRTAP19-3,KRTAP19-2,BACH1,KRTAP15-1,GRIK1,LINC00189,KRTAP6-1,KRTAP20-2,KRTAP11-1,KRTAP23-1,KRTAP24-1,KRTAP26-1,LINC00307,CCT8,KRTAP20-3,MAP3K7CL,CLDN17,KRTAP22-2,KRTAP27-1	3	gain
845	CGTE_12	21	46011225	46032244	21q22.3	KRTAP10-6,TSPEAR,KRTAP10-7,KRTAP10-8	4	gain
846	CGTE_12	21	46032355	46057457	21q22.3	KRTAP10-10,KRTAP10-9,KRTAP10-8,TSPEAR	0	loss
847	CGTE_12	21	46057516	46086730	21q22.3	TSPEAR,KRTAP10-11,KRTAP12-4,KRTAP12-3,KRTAP10-10,KRTAP12-2	3	gain
848	CGTE_12	21	46932092	46950855	21q22.3	SLC19A1,COL18A1	1	loss

849	CGTE_12	22	17589465	17590775	22q11.1	IL17RA	0	loss
850	CGTE_12	22	20137964	20229420	22q11.21	RTN4R,LINC00896,LOC284865	0	loss
851	CGTE_12	22	28192793	28196103	22q12.1	MN1	0	loss
852	CGTE_12	22	43608240	45281862	22q13.31-q13.2	MPPED1,SULT4A1,PRR5-ARHGAP8,LINC00229,ARHGAP8,SAMM50,EFCAB6,KIAA1644,LINC00207,PRR5,PARVB,LDOC1L,PNPLA3,SCUBE1,PARVG,LOC101927526,PNPLA5,EFCAB6-AS1,PHF21B	1	loss
853	CGTE_12	22	46509504	46780530	22q13.31	GTSE1-AS1,PPARA,MIRLET7B,MIR4763,GTSE1,TRMU,CELSR1,PKDREJ,MIRLET7BHG,TTC38,CDPF1	1	loss
854	CGTE_12	22	46931616	50169358	22q13.32-q13.31	TBC1D22A-AS1,LOC284930,LINC01310,C22orf34,GRAMD4,TBC1D22A,LINC00898,CERK,FAM19A5,CELSR1,LOC284933,LL22NC03-75H12.2,MIR4535,BRD1,MIR3201,MIR3667	1	loss
855	CGTE_12	22	50436448	50439063	22q13.33	IL17REL	1	loss
856	CGTE_12	22	50439137	50455037	22q13.33	IL17REL	5	gain
857	CGTE_12	22	50468850	50617727	22q13.33	MOV10L1,MLC1,PANX2	1	loss
858	CGTE_12	22	50636415	50647150	22q13.33	TRABD,SELO	1	loss
859	CGTE_12	22	50648627	50649334	22q13.33	SELO	3	gain
860	CGTE_12	22	50683861	50719903	22q13.33	MAPK12,HDAC10,MAPK11,PLXNB2	1	loss
861	CGTE_12	22	50870426	50876702	22q13.33	PPP6R2	1	loss
862	CGTE_12	22	50894703	50899100	22q13.33	SBF1	0	loss
863	CGTE_12	22	50899605	50904405	22q13.33	SBF1	4	gain
864	CGTE_12	22	50927456	50942903	22q13.33	MIOX,LMF2	8	gain
865	CGTE_12	22	51040227	51043541	22q13.33	MAPK8IP2	1	loss
866	CGTE_12	X	102840428	102841310	Xq22.2	TCEAL4	4	gain
867	CGTE_12	X	153037957	153040103	Xq28	PLXNB3	0	loss
868	CGTE_13	2	54531753	55527157	2p16.1-p16.2	ACYP2,C2orf73,RPS27A,CCDC88A,EML6,RPL23AP32,SPTBN1,RTN4,MTIF2,CLHC1,PRORSD1P,MIR4426	3	gain
869	CGTE_13	2	233194365	233405564	2q37.1	ECEL1,DIS3L2,CHRND,ALPPL2,CHRNG,ALPP,ECEL1P2,ALPI,PRSS56	1	loss
870	CGTE_13	3	48598268	48613430	3p21.31	PFKFB4,COL7A1,UCN2	1	loss
871	CGTE_13	3	49679647	49701348	3p21.31	BSN	1	loss
872	CGTE_13	3	52521197	52562758	3p21.1	NISCH,NT5DC2,STAB1	1	loss
873	CGTE_13	4	44626664	46086120	4p12	GABRG1,GUF1,YIPF7,GNPDA2	1	loss
874	CGTE_13	4	166416687	190876257	4q35.2-q34.3	HAND2-AS1,F11,LOC101928590,SNX25,F11-AS1,CDKN2AIP,TLR3,CLDN24,GLRA3,LINC01262,ENPP6,ASB5,CLDN22,LOC101928551,LINC01612,RWDD4,SPCS3,SCRG1,LOC101928131,SPATA4,FRG1,TENM3,ING2,ANKRD37,SPOCK3,LINC01098,DDX60L,GALNT7,MIR4455,NEK1,MIR3945,LOC100506085,HELT,MIR1305,LOC102723766,NEIL3,ADAM29,C4orf27,C4orf47,LINC01596,AADAT,GALNTL6,SLC25A4,LOC389247,FAM92A1P2,SORBS2,LRP2BP,GPM6A,HPGD,FLJ38576,MTNR1A,ANXA10,LOC339975,DCTD,LINC01099,DDX60,PRIMPOL,LVCAT8,HMGB2,LINC01179,TLL1,LOC100506122,STOX2,TRIML2,FAM149A,UFSP2,LOC101928314,LOC90768,CEP44,SLED1,ACSL1,ZFP42,HAND2,VEGFC,CYP4V2,CPE,AGA,WWC2-AS2,MIR3945HG,LINC01093,SAP30,TRIML1,TRAPPC11,CENPU,LOC100506272,LOC101928509,CASP3,LINC01060,CBR4,FBXO8,MIR6082,LINC00290,MFAP3L,CLCN3,LOC728175,MIR4276,SH3RF1,PALLD,PDLIM3,FAT1,WWC2-AS1,CCDC110,WWC2,KLKB1,WDR17,CFAP97,IRF2	1	loss
875	CGTE_13	5	140384	472156	5p15.33	HRAT5,PDCD6,EXOC3-AS1,SDHA,EXOC3,CCDC127,PP7080,AHRR,LRRC14B,PLEKHG4B	3	gain
876	CGTE_13	5	475667	488523	5p15.33	LOC100288152,SLC9A3	1	loss

877	CGTE_13	5	491875	1880334	5p15.33	CLPTM1L,LOC728613,MIR4277,SDHAP3,LOC101929034,LOC100996325,CEP72,SLC9A3,IRX4,LPCAT1,BRD9,TPPP,TRIP13,MIR4457,NDUFS6,CTD-3080P12.3,MRPL36,MIR4456,MIR6075,NKD2,TERT,SLC6A18,SLC12A7,SLC6A3,LOC100506688,ZDHHC11,MIR4635,SLC6A19,LINC01511	3	gain
878	CGTE_13	5	33937765	38953261	5p13.2-p13.1	PRLR,LMBRD2,C1QTNF3,UGT3A2,AMACR,MIR580,DNAJC21,NADK2,WDR70,LIFR-AS1,MIR7641-2,MIR3650,RAD1,RAI14,NUP155,CAPSL,NIPBL-AS1,BRIX1,RICTOR,SPEF2,SLC45A2,OSMR-AS1,OSMR,SLC1A3,EGFLAM-AS4,LINC01265,LIFR,C1QTNF3-AMACR,NIPBL,SKP2,C5orf42,TTC23L,UGT3A1,EGFLAM-AS2,AGXT2,GDNF-AS1,RANBP3L,RXFP3,IL7R,GDNF,LOC100506406,EGFLAM	3	gain
879	CGTE_13	5	39122309	41475827	5p13.1	DAB2,LOC101926940,RPL37,LOC100506548,SNORD72,LINC00603,C9,PRKAA1,PLCXD3,FYB,C6,CARD6,PTGER4,MROH2B,TTC33,C7	3	gain
880	CGTE_13	5	176304131	176309118	5q35.2	UNC5A,HK3	0	loss
881	CGTE_13	6	56032964	57058778	6p12.1-p11.2	BAG2,RAB23,KIAA1586,BEND6,DST,ZNF451,LOC101927211,LOC101930010,COL21A1	3	gain
882	CGTE_13	6	105280828	107077730	6q16.3-q21	ATG5,POPDC3,BVES,LINC00577,AIM1,PREP,HACE1,QRSL1,BVES-AS1,LIN28B,RTN4IP1,PRDM1	4	gain
883	CGTE_13	6	107087992	107097120	6q21	QRSL1	3	gain
884	CGTE_13	6	107099995	107372619	6q21	MIR587,LOC100422737,QRSL1,C6orf203	5	gain
885	CGTE_13	6	107389919	107391201	6q21	BEND3	3	gain
886	CGTE_13	6	107391275	108066376	6q21	SOBP,PDSS2,BEND3,SCML4	4	gain
887	CGTE_13	6	108067872	108068254	6q21	SCML4	1	loss
888	CGTE_13	6	108070774	108246228	6q21	SCML4,SEC63	5	gain
889	CGTE_13	6	108365887	108489400	6q21	OSTM1,NR2E1	6	gain
890	CGTE_13	6	108492486	110953382	6q21	MICAL1,ARMC2-AS1,LACE1,CEP57L1,NR2E1,CDK19,LOC100996634,GPR6,WASF1,FIG4,LINC00222,PPIL6,METTL24,DDO,CDC40,FOXO3,SMPD2,ZBTB24,MIR7641-2,AK9,SESN1,ARMC2,CD164,SLC22A16,CCDC162P,SNX3	3	gain
891	CGTE_13	6	120336436	123545406	6q22.31	TRDN,PKIB,SERINC1,HSF2,SMPDL3A,TBC1D32,FABP7,GJA1,CLVS2	3	gain
892	CGTE_13	6	123580716	126296127	6q22.32-q22.31	NCOA7-AS1,RNF217,NCOA7,HRAT13,TRDN,HDDC2,HINT3,HEY2,TPD52L1,NKAIN2,LOC643623,RNF217-AS1	3	gain
893	CGTE_13	6	126298763	126320800	6q22.32	TRMT11,HINT3	1	loss
894	CGTE_13	6	126329388	145075963	6q22.32-q24.2	PHACTR2,VNN2,NHEG1,STX7,MYB,ECHDC1,TMEM200A,LINC00326,EYA4,NHSL1,MIR3668,ABRACL,TXLNB,MIR548AJ1,TMEM244,HEBP2,THEMIS,HMGA1P7,TAAR6,ENPP1,C6orf58,TARID,LAMA2,LOC101928304,LOC102723649,IFNGR1,VTA1,MIR4465,SLC35D3,CCDC28A,CTAGE9,LOC101928231,OLIG3,TAAR3,FUCA2,CTGF,TBPL1,ARFGEF3,LINC01277,TCF21,SLC2A12,UTRN,LINC01013,MOXD1,IL20RA,LOC101928140,TAAR9,FLJ46906,KIAA0408,NMBR,ENPP3,STX11,IL22RA2,SMLR1,AHI1,L3MBTL3,LTV1,LINC01312,LOC100507406,PEX3,HBS1L,HYMAI,ARG1,LINC01625,TNFAIP3,MAP3K5,LOC101928461,EPB41L2,PEX7,PBOV1,ZC2HC1B,TAAR1,AIG1,LOC103352541,OR2A4,ADGRG6,SLC18B1,MIR5695,SGK1,LINC00271,PERP,PLAGL1,SF3B5,RPS12,PTPRK,PDE7B,LOC100130476,REPS1,SNORD100,LOC100132735,TAAR5,TAAR2,SNORA98,TAAR8,SNORA33,ADAT2,SOGA3,SAMD3,RSPO3,ALDH8A1,LINC01010,MIR3662,SNORD101,MED23,PHACTR2-AS1,RNF146,HIVEP2,GVQW2,VNN1,CENPW,MIR588,AKAP7,ECT2L,HECA,LOC153910,BCLAF1,MTFR2,VNN3,TRMT11,MIR3145,MAP7,LOC100507477,MIR548H5,CITED2,ARHGAP18	3	gain
895	CGTE_13	7	44146371	44152983	7p13	MIR4649,AEBP1	1	loss

896	CGTE_13	7	149473040	149523373	7q36.1	SSPO	1	loss
897	CGTE_13	8	17014249	33409031	8p21.1-p22	DMTN,FAM160B2,SLC25A37,CHMP7,NEFM,EXTL3,BIN3,NPM2,STC1,SLC7A2,ESCO2,PDGFRL,EBF2,MIR548V,TNFRSF10D,ZNF395,NRG1-IT3,HR,ADAMDEC1,MIR3148,RBPMS,REEP4,CNOT7,NUGGC,PURG,FBXO16,CCAR2,KIF13B,FAM183CP,MIR6841,DUSP4,LOC101929066,GFRA2,LINC00589,NKX2-6,NEFL,PDLIM2,SFTPC,CCDC25,DOCK5,NRG1,PNMA2,POLR3D,LOC101929172,SLC39A14,PTK2B,MBOAT4,ASAH1,PHYHIP,CLU,LOC101929315,LOC102467222,TRIM35,MTMR7,XPO7,LOC101929470,CHRNA2,INTS10,SCARA5,C8orf58,ZDHHC2,MIR6842,RHOBTB2,PBK,DCTN6,MIR3622B,MIR548O2,LOXL2,UBXN8,MIR6843,MIR7641-2,FZD3,ADAM7,SH2D4A,LOC286114,SMIM18,TEX15,WRN,DOK2,LOC101929450,SORBS3,MIR3622A,PPP3CC,NAT2,FGL1,TNFRSF10A,INTS9,CSGALNACT1,LZTS1-AS1,BMP1,NAT1,FGF17,STMN4,ENTPD4,ELP3,NKX3-1,NRG1-IT1,MIR320A,BNIP3L,GSR,EGR3,PSD3,SLC18A1,R3HCC1,KCTD9,PEBP4,MIR6876,FUT10,TNFRSF10C,GNRH1,PIWIL2,BIN3-IT1,ADRA1A,DPYSL2,RNF122,VPS37A,ATP6V1B2,NUDT18,LOC101929294,ADAM28,LOC254896,SARAF,GTF2E2,LOC100128993,EPHX2,LEPROTL1,CDCA2,TTI2,LZTS1,LOC286059,RBPMS-AS1,EXTL3-AS1,PNOC,MIR4287,MTUS1,HMBOX1,LPL,PPP2R2A,MAK16,PCM1,SCARA3,LGI3,PPP2CB,LOC389641,MIR4288,TNFRSF10B,LOC101929237,LOC100507156	3	gain

898	CGTE_13	8	41557823	142264794		MIR5681B,SGK3,RGS20,LOC101929528,TGS1,C8orf59,KLHL38,ST18,LINC01592,LINC00251,COX6C,RIPK2,LACTB2,RB1CC1,RNF19A,TMEM75,TCF24,MIR599,CHMP4C,SNTB1,PPP1R42,NCRNA00250,CASC21,MIR7705,VDAC3,FABP5,LOC392232,YWHAZ,LOC102724710,ZHX1-C8orf76,LINC01030,DPYS,UQCRB,HHLA1,MTDH,FLJ42969,CHRNB3,RP1,POTEA,ANKRD46,RAD21,NPBWR1,PRKDC,MIR3149,LOC101927822,CA2,VPS13B,SOX17,LOC401463,ERICH5,C8orf34-AS1,TMEM74,STMN2,NSMAF,LRRC69,RAB2A,CHRNA6,SBSPON,TCEB1,MRPL15,RPL30,HPYR1,SLC20A2,ZNF572,TRAM1,OPRK1,MIR6844,SDCBP,LOC102724623,MIR7848,PHF20L1,MRPL13,IMPAD1,CYP7A1,MIR2052,MAL2,LINC01289,MIR1208,TRIM55,OC90,LY96,MIR7641-2,FABP12,LINC01591,DCAF4L2,PCAT1,PI15,SLC25A32,AP3M2,MCMDC2,LINC01607,ZBTB10,LOC101929217,LINC00824,STAU2,POLB,TRMT12,TRIB1,CHD7,LOC105375734,LINC00977,ANXA13,PLAT,ARFGEF1,LINC01419,LINC01606,ZC2HC1A,C8orf37,SULF1,TRHR,SNORD54,LOC101929488,YTHDF3,C8orf87,LOC100500773,AZIN1-AS1,MIR3151,TNFRSF11B,PXDNL,MIR3686,IL7,ASAP1,ZHX2,LOC286177,CYP7B1,FAM84B,RBM12B,TMEM65,YTHDF3-AS1,RALYL,MTERF3,FABP9,NSMCE2,ZFHX4,PRDM14,C8orf89,RPL7,SLC26A7,ZFAT,FER1L6,PCMTD1,LINC01299,XKR4,MOS,LOC101927040,RAD21-AS1,LINC01603,MIR8084,CPA6,EBAG9,FER1L6-AS2,LINC00861,BAALC,SLC10A5,CA3,MIR1204,ZFAT-AS1,MIR30B,LOC101929709,POMK,LINC01609,MIR2053,MIR5194,TCEA1,TAF2,LOC101241902,TP53INP1,PLEKHF2,LRRC6,RRM2B,LOC102724612,DEPTOR,UG0898H09,RNF170,EFCAB1,HRSP12,SDC2,SNTG1,NECAB1,MIR875,LINC00535,NDUFAF6,SYBU,LOC101929897,LINC00293,KCNK9,SLC30A8,C8orf34,SPAG1,DKK4,RRS1,PDP1,PLAG1,BAALC-AS2,REXO1L2P,INTS8,LOC101926908,DSCC1,PAG1,CSPP1,CCDC26,MIR3610,ARMC1,ZFPM2-AS1,STAU2-AS1,NDRG1,KHDRBS3,ANK1,PMP2,OSGIN2,MIR124-2HG,ENPP2,CLVS1,EIF3H,MSC,LINC00968,CPQ,SMIM19,ST3GAL1,KLF10,COL22A1,DCAF13,MIR4471,MYBL1,SPIDR,CASC9,MIR3150A,CCNE2,RSPO2,HGSNAT,IKBKB,RAD54B,WWP1,MIR4663,LRRCC1,COPS5,UBR5-AS1,CA1,LOC105375650,LINC00534,PDE7A,ZNF704,CDH17,LINC01111,SAMD12-AS1,PKIA-AS1,SBF1P1,SNAI2,GDF6,PTDSS1,MED30,SLC7A13,FBXO32,NDUFB9,DCSTAMP,AZIN1,TSPYL5,FAM83A-AS1,LINC00967,TRIQK,OTUD6B,MIR1206,MIR4662B,CEBPD,LINC01608,THAP1,NBN,ANGPT1,NCOA2,LOC101927543,ZHX1,TRPA1,STK3,SAMD12,MIR3150B,PTK2,OTUD6B-AS1,PKIA,FLJ46284,LOC101927845,LOC101927066,MIR30D,MIR378D2,C8orf88,LINC01602,IMPA1,MCM4,GDAP1,MIR4662A,SNX31,BAALC-AS1,ZFHX4-AS1,ATP6V0D2,CPNE3,RUNX1T1,TRAPPC9,TMEM68,NIPAL2,TRPS1,HAS2,DPY19L4,LYN,COLEC10,KCNB2,MIR4469,MYC,JPH1,LINC00964,C8orf44,LINC01298,CA8,ODF1,CNBD1,SNORD87,MIR1207,FER1L6-AS1,TOX,MTBP,FABP4,RDH10,C8orf44-SGK3,PTTG3P,CASC19,CSMD3,MIR2052HG,TMEM64,RRS1-AS1,EIF3E,WDYHV1,SDR16C6P,C8orf22,CNGB3,SLC45A4,UBE2V2,FAM83A,ENY2,FZD6,LOC101929415,LINC01301,OSR2,FAM110B,LYPLA1,SNORA72,LRP12,KCNS2,MIR1273A,LOC101929268,ABRA,ATP6V1H,UTP23,MSC-AS1,GGH,POU5F1B,PABPC1,PREX2,LACTB2-AS1,EMC2,ENTA,HNF4G,KIAA1429,LOC100130298,NCALD,COL14A1,E2F5,ASPH,KAT6A,	NA	
899	CGTE_13	8	145537478	145758721	8q24.3	MIR6849,CPSF1,C8orf82,CYHR1,MIR939,HSF1,FBXL6,MIR6893,GPT,SLC39A4,ADCK5,FOXH1,ARHGAP39,MIR6848,LRRC14,DGAT1,TMEM249,SCRT1,KIFC2,SLC52A2,RECQL4,LRRC24,TONSL,TONSL-AS1,MFSD3,PPP1R16A,VPS28	1	loss
900	CGTE_13	8	145759506	146279543	8q24.3	ZNF517,C8orf33,ZNF252P,COMMD5,ARHGAP39,MIR6850,ZNF250,ZNF251,ZNF16,ZNF34,TMED10P1,ZNF7,RPL8,ZNF252P-AS1	3	gain
901	CGTE_13	9	141012938	141016420	9q34.3	CACNA1B	0	loss
902	CGTE_13	10	61802379	64893349	10q21.2-q21.3	CDK1,C10orf107,NRBF2,RTKN2,LOC283045,ZNF365,LINC00845,ANK3,EGR2,TMEM26-AS1,TMEM26,ARID5B,RHOBTB1,MIR548AV,ADO	3	gain
903	CGTE_13	10	64906042	65383499	10q21.3	JMJD1C,JMJD1C-AS1,MIR1296,NRBF2,REEP3	5	gain

904	CGTE_13	10	65383534	72026267	10q22.1-q21.3	SNORD98,TACR2,LRRTM3,POU5F1P5,TET1,COL13A1,PPA1,DNAJC12,DDX50,LOC101928994,HKDC1,DNA2,CCAR1,REEP3,CTNNA3,LINC01515,H2AFY2,C10orf35,HK1,MIR7151,STOX1,AIFM2,HERC4,NPFFR1,MYPN,SRGN,SIRT1,TSPAN15,RUFY2,TYSND1,VPS26A,DDX21,SAR1A,MIR1254-1,ATOH7,NEUROG3,KIAA1279,PBLD,ANXA2P3,LOC101928961,HNRNPH3,SLC25A16,SUPV3L1	3	gain
905	CGTE_13	10	72520259	79795586	10q22.3-q22.2	C10orf105,MCU,MRPS16,FAM149B1,SPOCK2,ZSWIM8-AS1,LOC102723439,ASCC1,LOC101929234,P4HA1,USP54,SEC24C,ZSWIM8,PPP3CB-AS1,ADK,RPS24,DDIT4,SGPL1,LOC102723377,ZNF503-AS1,CHCHD1,MIR7152,ZNF503,CDH23,OIT3,C10orf55,MIR606,ANAPC16,DUSP13,NUDT13,DNAJB12,DNAJC9-AS1,AGAP5,PPP3CB,MICU1,CHST3,PCBD1,SLC29A3,C10orf11,POLR3A,KAT6B,AP3M1,ANXA7,VCL,VDAC2,C10orf54,DUPD1,BMS1P4,UNC5B,FUT11,GLUD1P3,KCNMA1-AS3,PLA2G12B,SYNPO2L,DNAJC9,PSAP,SAMD8,ZNF503-AS2,TBATA,NDST2,COMTD1,ECD,KCNMA1-AS2,CFAP70,KCNMA1,ADAMTS14,DLG5,DLG5-AS1,UNC5B-AS1,KCNMA1-AS1,MIR4676,CAMK2G,MSS51,PLAU,MYOZ1,LOC105378367	3	gain
906	CGTE_13	11	51412006	59225350	11p11.12-q12.1	OR5AK2,OR4A15,SSRP1,BTBD18,OR5R1,OR8I2,CNTF,OR9G1,OR4P4,ZDHHC5,CTNND1,OR4A5,OR8J1,OR4S2,OR5T3,GLYAT,TIMM10,OR4C6,OR8K3,LOC283194,OR5B12,DTX4,LPXN,MIR6128,OR8U8,OR9Q2,OR7E5P,OR4C46,GLYATL2,TRIM51HP,OR10W1,OR5I1,OR8U1,OR10Q1,OR1S2,OR8H2,SLC43A3,APLNR,SLC43A1,OR5M9,OR9G9,OR5T1,UBE2L6,OR4C15,ZFP91-CNTF,OR5T2,OR1S1,PRG3,ZFP91,OR6Q1,OR4C16,OR10AG1,OR5A2,FAM111A,OR5D16,OR5AN1,C11orf31,TRIM48,OR5M1,GLYATL1,OR8H3,OR5AS1,OR9Q1,LRRC55,OR5F1,OR5M3,OR9I1,TNKS1BP1,OR5AP2,OR5J2,OR5L1,OR5B17,LOC101927120,TMX2-CTNND1,PRG2,OR5M11,TRIM51,OR8K1,SMTNL1,OR5B2,OR5D18,OR4C11,OR5D14,P2RX3,FAM111B,OR5W2,OR4D6,OR5A1,SERPING1,OR5AK4P,OR8J3,OR9G4,TMX2,OR8K5,MIR130A,OR5B21,OR5M8,RTN4RL2,CLP1,OR8H1,LOC101927204,YPEL4,OR5D13,OR5B3,OR5AR1,MED19,OR5L2,OR4A16,MPEG1,OR5M10	1	loss
907	CGTE_13	11	67433645	69949380	11q13.2-q13.3	FAM86C2P,MRGPRF-AS1,CPT1A,KMT5B,LOC101928443,ANO1-AS2,GAL,MYEOV,UNC93B1,IGHMBP2,MRGPRF,MIR4691,ANO1,ALDH3B1,CHKA,LOC338694,LINC01488,MTL5,TPCN2,FGF19,LRP5,MIR3164,CCND1,MIR6753,NDUFS8,PPP6R3,FGF3,MIR7113,MRGPRD,TCIRG1,MRPL21,C11orf24,FGF4,ORAOV1,ALDH3B2	3	gain
908	CGTE_13	11	69950051	73364704	11q13.3-q13.4	ARHGEF17,PPFIA1,MIR3664,STARD10,NUMA1,MIR6754,ATG16L2,MIR3165,FAM86C1,FOLR3,ANO1,P2RY2,ZNF705E,KRTAP5-8,PLEKHB1,SHANK2,SHANK2-AS1,FAM168A,IL18BP,ARAP1,NADSYN1,DEFB108B,PHOX2A,P2RY6,LOC100133315,FOLR1,MIR139,KRTAP5-7,FLJ42102,KRTAP5-10,CLPB,MIR4692,LAMTOR1,LOC100128494,FADD,ALG1L9P,FCHSD2,SHANK2-AS3,RELT,CTTN,FOLR2,ANAPC15,KRTAP5-11,PDE2A,KRTAP5-9,LRTOMT,INPPL1,RNF121,MIR548K,LINC01537,LOC100129216,DHCR7,MIR4459	4	gain

909	CGTE_13	11	73366759	77595808	11q13.4-q14.1	TPBGL,LOC283214,KCNE3,ARRB1,LOC101928580,PAAF1,PAK1,RNF169,OR2AT4,DGAT2,ACER3,KLHL35,LRRC32,WNT11,MAP6,PRKRIR,PLEKHB1,CLNS1A,MIR4696,COA4,AAMDC,GUCY2EP,SERPINH1,RSF1,GDPD5,MYO7A,CAPN5,EMSY,MOGAT2,SPCS2,OMP,TSKU,PGM2L1,MRPL48,C2CD3,B3GNT6,NEU3,MIR326,PPME1,LOC101928837,XRRA1,CHRDL2,UVRAG,LIPT2,LOC646029,AQP11,UCP3,POLD3,SNORD15A,RPS3,RAB6A,GDPD4,P4HA3,DNAJB13,SNORD15B,LOC100506127,INTS4,UCP2,SLCO2B1	3	gain
910	CGTE_13	11	77614647	78489773	11q14.1	NARS2,KCTD21-AS1,NDUFC2-KCTD14,KCTD21,TENM4,KCTD14,USP35,NDUFC2,THRSP,LOC101928896,GAB2,INTS4,LOC101928865,ALG8	4	gain
911	CGTE_13	11	78497874	87032472	11q14.2-q14.1	CCDC81,DLG2,MIR4300,MIR5579,LOC100506368,CCDC90B,ME3,MIR4300HG,EED,MIR6755,CREBZF,SYTL2,DDIAS,TMEM126A,TMEM126B,MIR708,TMEM135,RAB30-AS1,CCDC83,LOC101928944,CCDC89,PCF11,PICALM,OR7E2P,PRSS23,TENM4,FAM181B,PRCP,SNORA70E,C11orf73,RAB30,ANKRD42,FZD4	3	gain
912	CGTE_13	11	91536326	102094697	11q21-q22.1	SRSF8,TMEM133,TAF1D,LOC101929295,HEPHL1,CNTN5,ARHGAP42,AMOTL1,LOC100129203,CEP57,CCDC67,SNORA1,ANGPTL5,PGR,C11orf97,MIR548L,SNORD5,FUT4,LOC101054525,ANKRD49,SCARNA9,LOC100128386,CEP126,PIWIL4,MTMR2,CCDC82,JRKL-AS1,MIR1260B,C11orf70,SNORA40,KDM4E,PANX1,TRPC6,MED17,ENDOD1,MIR1304,YAP1,KDM4D,IZUMO1R,SNORA32,SNORA8,SNORA25,SNORA18,SESN3,FAT3,MIR3920,SMCO4,MRE11A,SLC36A4,VSTM5,MAML2,FAM76B,GPR83,CEP295,MTNR1B,SNORD6,CWC15,C11orf54,JRKL	3	gain
913	CGTE_13	11	115080340	118392809	11q23.3	MPZL3,BACE1-AS,CD3E,JAML,FXYD6-FXYD2,BACE1,CADM1,TAGLN,DSCAML1,APOA4,CEP164,TMPRSS4-AS1,APOA1-AS,APOC3,LOC101929089,UBE4A,LINC00900,TMPRSS13,CD3G,PAFAH1B2,SCN2B,ZPR1,RNF214,SIDT2,SCN4B,CD3D,BUD13,FXYD6,SIK3,IL10RA,ATP5L,APOA1,PCSK7,MPZL2,FXYD2,APOA5,KMT2A,LOC100131626,TMPRSS4,LOC100652768	3	gain
914	CGTE_13	12	46325273	46347667	12q12	SCAF11	1	loss
915	CGTE_13	12	57556550	57605616	12q13.3	MIR1228,LRP1	1	loss
916	CGTE_13	13	25404580	25418092	13q12.12	RNF17	0	loss
917	CGTE_13	14	20201784	20404833	14q11.2	OR4K2,OR4K1,OR4Q3,OR4M1,OR4N2,OR4K5	3	gain
918	CGTE_13	14	20443704	20781836	14q11.2	CCNB1IP1,OR11H6,OR4K17,OR4K15,OR4L1,OR4K14,OR4N5,OR11H4,TTC5,OR11G2,OR4K13	4	gain
919	CGTE_13	14	20781907	20783844	14q11.2	CCNB1IP1	1	loss
920	CGTE_13	14	20784001	20876569	14q11.2	TEP1,PARP2,RPPH1,SNORD126,CCNB1IP1	4	gain
921	CGTE_13	14	20896952	21504014	14q11.2	OSGEP,RNASE11,ANG,RNASE6,LOC254028,RNASE10,TPPP2,NDRG2,RNASE12,RNASE3,RNASE2,PNP,METTL17,RNASE1,APEX1,MIR6717,RNASE9,KLHL33,EDDM3A,RNASE4,ECRP,SLC39A2,OR6S1,EDDM3B,TMEM55B,LOC101929718,RNASE13	3	gain
922	CGTE_13	14	23314861	23792440	14q11.2	AJUBA,PABPN1,CEBPE,LRP10,RBM23,LOC101926933,PSMB5,PPP1R3E,MIR4707,REM2,CDH24,BCL2L2-PABPN1,HOMEZ,PRMT5,BCL2L2,C14orf93,HAUS4,SLC7A8,PSMB11,MMP14,RNF212B,PRMT5-AS1,C14orf119,ACIN1	3	gain
923	CGTE_13	14	103478622	103600330	14q32.32	CDC42BPB,TNFAIP2,EXOC3L4	1	loss
924	CGTE_13	14	105408891	105418727	14q32.33	AHNAK2	1	loss
925	CGTE_13	15	56665563	56721424	15q21.3	MNS1,TEX9	1	loss
926	CGTE_13	15	90172648	90196148	15q26.1	KIF7	1	loss

927	CGTE_13	15	91421368	91428753	15q26.1	FURIN,FES	1	loss
928	CGTE_13	16	28506429	28515495	16p11.2	APOBR,IL27	1	loss
929	CGTE_13	17	60741821	60769686	17q23.2	MRC2	1	loss
930	CGTE_13	17	73727230	73831648	17q25.1	H3F3B,UNC13D,GALK1,UNK,ITGB4,MIR4738	1	loss
931	CGTE_13	17	79972924	80065616	17q25.3	DCXR,ASPSCR1,FASN,GPS1,RAC3,DUS1L,LRRC45,RFNG,SNORD134,CCDC57,STRA13	1	loss
932	CGTE_13	20	3214107	3214966	20p13	SLC4A11	0	loss
933	CGTE_13	20	3215205	60881550		JAG1,SLC2A10,GTSF1L,PCNA,ERGIC3,LINC01428,CASS4,OSER1-AS1,SPINT3,ZFP64,NCOA5,TMEM189-UBE2V1,GFRA4,HM13-AS1,LOC100506175,MIR7641-2,MIR1289-1,MIR3646,MANBAL,CRNKL1,MCTS2P,SNORA60,STX16-NPEPL1,FAM209B,FRG1DP,CDC25B,FAM209A,EPPIN-WFDC6,WFDC5,BFSP1,CDK5RAP1,SRSF6,PLTP,LOC100506384,STX16,CST5,MCM8-AS1,ZNF831,PLCG1-AS1,DOK5,LOC100505515,SNX21,KIF3B,AP5S1,SEMG1,C20orf196,RBM39,LOC101929207,STK4-AS1,PRNP,MIR3195,SNORD12C,MYLK2,LOC101926889,WFDC10B,MIR548AG2,LOC100270804,RNF24,LOC101929395,KCNK15-AS1,LPIN3,PPP1R16B,PMEPA1,JPH2,TFAP2C,DTD1,LOC339568,VSTM2L,HNF4A-AS1,LINC00489,TNNC2,CENPB,SNAP25-AS1,PET117,C20orf194,PRND,RIMS4,RTFDC1,NCOR1P1,CYP24A1,ZNF337-AS1,MIR646,LINC01427,SOGA1,SUMO1P1,WISP2,XKR7,MIR663A,C20orf85,NKILA,CST13P,TTPAL,VAPB,LOC101926955,SLX4IP,C20orf27,CST9,MIR6870,MIR1302-5,SNORA71C,ADAM33,ESF1,MKRN7P,LINC00028,MIR663AHG,MKKS,SLPI,LOC101929371,C20orf78,SEMG2,MC3R,TP53TG5,MIR4533,CD93,FER1L4,TUBB1,LINC01431,C20orf197,R3HDML,YWHAB,BPIFA1,LOC101927932,CST7,SLC32A1,TMEM189,CBLN4,ADNP,CDH26,RPN2,KIAA1755,NFS1,LOC100131496,NXT1,NKX2-4,LOC101927159,TGIF2,ELMO2,MATN4,RALY,STK4,UQCC1,PI3,PANK2,PLCG1,HNF4A,SNHG11,BLCAP,DNMT3B,LOC101928048,LOC79160,DEFB119,PIGU,FOXS1,WFDC8,LINC01260,SNORD17,LINC01370,MAVS,LINC01270,KCNG1,PCIF1,SNAP25,ADIG,RBPJL,TPX2,MAP1LC3A,ABHD12,PARD6B,CDH4,RALY-AS1,COMMD7,SDC4,WFDC6,KIZ-AS1,LOC284788,NPEPL1,ANKEF1,ZMYND8,TSPY26P,PREX1,LBP,CEBPB,EPPIN,SNX5,WFDC13,LOC339593,PHACTR3,RPRD1B,PLAGL2,AURKA,FRG1BP,NAPB,CST1,MAPRE1,MLLT10P1,ISM1-AS1,CST3,LOC101929312,GDF5,MIR296,PLCB4,LINC01430,SNORA71B,MIR4756,LINC01273,STAU1,BCAS1,PIGT,CBFA2T2,SPINT4,SNORD12,DUSP15,PHF20,ACTL10,DEFB115,MIR103A2,SPEF1,SNORA71A,MIR644A,SLC24A3,SLC9A8,SPAG4,FAM182A,IFT52,LINC01598,TLDC2,LOC101929125,SCAND1,TRPC4AP,LINC01440,ZNFX1,TMEM230,LINC00657,RIN2,SNORA71E,CFAP61,SLMO2-ATP5E,FAM217B,ZNF133,CST11,SEC23B,PAX1,ANKRD60,NEURL2,ZNF341,HM13,MYL9,PROCR,BPIFA2,NNAT,GGTLC1,TGM2,TGIF2-C20orf24,OSER1,NCOA6,CTCFL,LINC01429,ZNF217,E2F1,PTPN1,MTRNR2L3,ASIP,FAM83C-AS1,GNAS,MIR4532,CCM2L,CDH22,ACSS2,SAMHD1,CHD6,KCNB1,COX4I2,SUN5,UBE2C,LOC643406,SLC23A2,ADNP-AS1,WFDC2,RNF114,NDRG3,BPIFB6,SCP2D1,RALGAPA2,MIR499A,CPNE1,INSM1,THBD,PTGIS,ATRN,HMGB3P1,CTSA,LOC729296,PPP4R1L,SLA2,NFATC2,SSTR4,TRERNA1,CST4,LINC00652,SYS1,DEFB118,C20orf173,UBE2V1,NOL4L,ADRM1,GCNT7,HAO1,MIR3194,BMP7,ACOT8,LINC00493,TOMM34,LOC149684,ROMO1,LOC102606466,LOC100134868,NANP,TASP1,GINS1,PFDN4,WFDC3,CASC20,LINC00656,OSBPL2,RAE1,MACROD2-IT1,ATP9A,LOC101929608,CSTL1,FOXA2,PTPRT,LOC101929526,SPO11,APCDD1L-AS1,MIR4325,DEFB124,SULF2,SGK2,LINC01524,CTNNBL1,CEP250,SLC13A3,L3MBTL1,ZNF341-	NA	
934	CGTE_13	20	60881681	60912710	20q13.33	ADRM1,LAMA5,MIR4758	1	loss
935	CGTE_13	20	62614441	62697906	20q13.33	TCEA2,SOX18,PRPF6,LINC00176	3	gain

936	CGTE_13	20	62744500	62905019	20q13.33	PCMTD2,MYT1	3	gain
937	CGTE_13	21	45970688	46117586	21q22.3	KRTAP10-8,KRTAP12-4,KRTAP10-4,KRTAP10-5,KRTAP10-11,KRTAP10-10,TSPEAR,KRTAP10-2,KRTAP10-7,KRTAP10-3,KRTAP12-3,KRTAP12-1,KRTAP10-6,KRTAP12-2,KRTAP10-9,KRTAP10-12	1	loss
938	CGTE_13	21	47531374	47575502	21q22.3	FTCD,COL6A2	1	loss
939	CGTE_13	22	21343045	22020513	22q11.21	TMEM191C,THAP7-AS1,SDF2L1,BCRP2,LRRC74B,SLC7A4,MIR649,PI4KAP2,MIR130B,RIMBP3B,FAM230B,RIMBP3C,POM121L8P,CCDC116,UBE2L3,LZTR1,P2RX6P,MIR301B,HIC2,YDJC,TUBA3FP,P2RX6,PPIL2,THAP7	1	loss
940	CGTE_13	22	50659334	50755844	22q13.33	MAPK12,TUBGCP6,PLXNB2,DENND6B,MAPK11,HDAC10	1	loss
941	CGTE_13	X	96167296	96194162	Xq21.33	DIAPH2	0	loss
942	CGTE_13	X	153005560	153050948	Xq28	PLXNB3,SRPK3,ABCD1	0	loss
943	CGTE_13	X	153216772	153222187	Xq28	HCFC1	0	loss
944	CGTE_14	1	876418	878465	1p36.33	SAMD11	17	gain
945	CGTE_14	1	909258	914766	1p36.33	PERM1,PLEKHN1	6	gain
946	CGTE_14	1	984547	987203	1p36.33	AGRN	9	gain
947	CGTE_14	1	1132786	1139644	1p36.33	TNFRSF18,TTLL10	7	gain
948	CGTE_14	1	1354456	1355917	1p36.33	ANKRD65	8	gain
949	CGTE_14	1	6530507	6531354	1p36.31	PLEKHG5	21	gain
950	CGTE_14	1	17263187	17298275	1p36.13	CROCC	3	gain
951	CGTE_14	2	43451377	43452948	2p21	ZFP36L2	5	gain
952	CGTE_14	2	45169266	45171964	2p21	SIX3	4	gain
953	CGTE_14	2	73513497	73613325	2p13.1	ALMS1,EGR4	4	gain
954	CGTE_14	2	74734693	74742176	2p13.1	PCGF1,TLX2	0	loss
955	CGTE_14	2	95541403	95691686	2q11.1	MAL,TEKT4,LOC442028	7	gain
956	CGTE_14	2	101767605	101870015	2q11.2	CNOT11,TBC1D8	6	gain
957	CGTE_14	2	177036459	177134590	2q31.1	HAGLROS,MTX2,HOXD1,HAGLR,HOXD3,MIR7704	5	gain
958	CGTE_14	2	208631540	208633633	2q33.3	FZD5	4	gain
959	CGTE_14	2	219757660	219825892	2q35	CDK5R2,WNT10A,LINC01494	0	loss
960	CGTE_14	2	219857691	219867844	2q35	CRYBA2,CFAP65,LOC100129175,MIR375	22	gain
961	CGTE_14	2	233386477	233387878	2q37.1	PRSS56	0	loss
962	CGTE_14	2	239009003	239013579	2q37.3	ESPNL	9	gain
963	CGTE_14	2	241989264	241991846	2q37.3	SNED1	3	gain
964	CGTE_14	2	242755629	242757743	2q37.3	NEU4	0	loss
965	CGTE_14	2	242757746	242794264	2q37.3	NEU4,PDCD1	3	gain
966	CGTE_14	3	48936127	48956549	3p21.31	ARIH2OS,SLC25A20,ARIH2	6	gain
967	CGTE_14	3	128780416	128781263	3q21.3	GP9	4	gain
968	CGTE_14	3	138724504	138739537	3q23	PRR23B,PRR23A	0	loss
969	CGTE_14	4	960201	961182	4p16.3	DGKQ	5	gain
970	CGTE_14	4	982642	985752	4p16.3	IDUA,SLC26A1	5	gain
971	CGTE_14	4	1806505	1808710	4p16.3	FGFR3	4	gain

972	CGTE_14	5	140384	45695927	5p13.1-p15.1	CCDC127,PRLR,LINC01017,LIFR-AS1,FLJ33360,C9,C7,LOC101926940,MARCH11,EGFLAM,LOC101929681,TARS,LOC100506639,FGF10-AS1,LOC442132,C5orf42,IRX2,LOC153684,TAS2R1,LOC101929034,BRIX1,ROPN1L,LOC401177,MIR4277,MIR4637,LINC01511,EXOC3-AS1,NIPBL-AS1,PAIP1,C5orf22,SDHAP3,LPCAT1,LOC648987,CDH18,RANBP3L,LOC101929645,LOC100505625,ZNF622,NPR3,RXFP3,ANKH,CTD-2350J17.1,PDCD6,LOC101929412,GOLPH3,NUP155,SKP2,LINC01019,LOC100996325,IRX4,CDH9,AHRR,PRKAA1,SRD5A1,PP7080,UBE2QL1,C5orf28,CCT5,OXCT1,FBXO4,DNAH5,CMBL,LOC101929505,MIR4458HG,C1QTNF3,BASP1,SEPP1,NNT,ANKRD33B,GHR,SLC6A19,PLCXD3,NKD2,GDNF-AS1,MTMR12,PRDM9,SNORD72,C1QTNF3-AMACR,LOC285692,WDR70,PAPD7,CLPTM1L,DAP,LINC01265,SUB1,ROPN1L-AS1,MROH2B,LOC100506548,ADAMTS12,LINC01194,ZDHHC11,OSMR-AS1,NNT-AS1,LINC01020,SLC6A18,LOC101929454,CDH12,RAI14,LOC100506406,SNORD141A,TTC23L,LRRC14B,TRIP13,CAPSL,GDNF,DNAJC21,HCN1,LOC340107,MIR4458,RICTOR,SEMA5A,ADCY2,LINC01018,MARCH6,CTD-2201E9.1,PMCHL1,MYO10,LOC100506858,SNHG18,NDUFS6,MIR887,ZFR,LOC100130744,TERT,MIR4279,LSP1P3,TRIO,ZNF131,EXOC3,ADAMTS16,IL7R,TPPP,UGT3A1,SNORA105A,PTGER4,FLJ32255,MIR4456,LIFR,C5orf49,CCDC152,FAM134B,HRAT5,RPL37,LOC101926960,SLC12A7,SLC9A3,MIR4454,OTULIN,DAB2,LMBRD2,DROSHA,C5orf34,MIR3650,CDH10,PDZD2,NADK2,LOC285696,CTD-2194D22.4,ANXA2R,MIR580,OXCT1-AS1,CARD6,MIR4636,CTNND2,MIR4278,FGF10,SLC1A3,C6,LOC728613,MIR4635,ICE1,FAM105A,MED10,GUSBP1,FAM173B,C5orf38,SNORD141B,CCL28,LOC101929284,LINC00603,CTD-2297D10.2,NIM1K,LINC01021,SNORD123,TTC33,MIR6075,EGFLAM-AS4,AGXT2,LINC01377,CTD-3080P12.3,RAD1,CDH6,LOC101929153,MRPS30,SDHA,MIR6131,BRCAT54,LOC100132356,LOC100506688,BRD9,MTRR,LOC100288152,AMACR,EGFLAM-AS2,FBXL7,BRCAT107,C5orf17,LOC101929524,MRPL36,FYB,LOC101929660,MIR579,SNORA105B,UGT3A2,NSUN2,FASTKD3,NIPBL,SLC6A3,HMGCS1,OSMR,SLC45A2,SPEF2,LOC340113,PLEKHG4B,CEP72,IRX1,MIR4457,C5orf51,MIR7641-2,LOC729506	3	gain
973	CGTE_14	5	92920681	92924208	5q15	MIR548AO,NR2F1	8	gain
974	CGTE_14	5	140188239	140188988	5q31.3	PCDHA2,PCDHA3,PCDHA4,PCDHA1	6	gain
975	CGTE_14	5	140188993	140250118	5q31.3	PCDHA7,PCDHA4,PCDHA3,PCDHA1,PCDHA10,PCDHA5,PCDHA11,PCDHA6,PCDHA8,PCDHA2,PCDHA9	3	gain
976	CGTE_14	6	21231307	21595242	6p22.3	SOX4,CDKAL1,LINC00581	5	gain
977	CGTE_14	6	43227286	43251172	6p21.1	TTBK1	6	gain
978	CGTE_14	6	43251177	43253116	6p21.1	TTBK1	4	gain
979	CGTE_14	6	154831135	155055139	6q25.2	SCAF8,CNKSR3	6	gain
980	CGTE_14	7	1023809	1026519	7p22.3	CYP2W1	5	gain
981	CGTE_14	7	1543466	1586849	7p22.3	INTS1,MAFK,TMEM184A	0	loss
982	CGTE_14	7	2434279	2584703	7p22.3	GRIFIN,CHST12,LOC101927181,BRAT1,MIR4648,LFNG	3	gain
983	CGTE_14	7	2697926	2752620	7p22.3	TTYH3,AMZ1	4	gain
984	CGTE_14	7	5402323	5417648	7p22.1	TNRC18	5	gain
985	CGTE_14	7	5427289	5569119	7p22.1	FBXL18,LOC221946,MIR589,TNRC18,ACTB	3	gain

986	CGTE_14	7	5633317	27142120	7p15.2-p21.2	NFE2L3,KIAA0087,SOSTDC1,HOXA2,SCIN,LOC100505921,LOC541472,RNF216-IT1,LOC101927630,AGR3,EIF2AK1,MALSU1,SP4,DFNA5,MIR1302-6,RSPH10B,FSCN1,ISPD-AS1,MIR6874,TMEM106B,CYCS,AIMP2,MIOS,MACC1-AS1,LOC441204,MIR1183,DNAH11,GPNMB,THSD7A,AGR2,LOC100505938,STEAP1B,MPP6,CBX3,HDAC9,MEOX2,NPY,UMAD1,MIR3146,SP8,RSPH10B2,ARL4A,KLHL7,LOC100131257,GLCCI1,LOC101927769,MIR148A,RAPGEF5,C7orf71,DGKB,PHF14,LINC01162,ISPD,PMS2,STK31,TRA2A,ITGB8,ETV1,OCM,C7orf31,HNRNPA2B1,IGF2BP3,MACC1,LOC100506178,RPS2P32,SNORD93,SNX13,TMEM196,CDCA7L,MEOX2-AS1,COL28A1,ANKMY2,FAM221A,SNX10,KCCAT333,ZDHHC4,RAC1,HOTAIRM1,LOC401312,AHR,NXPH1,USP42,DAGLB,SKAP2,CCDC126,GRID2IP,FAM220A,CCZ1B,AGMO,FAM126A,RNF216,CLK2P1,ZNF12,FERD3L,LOC101927811,TSPAN13,PRPS1L1,TOMM7,VWDE,LOC101927391,RPA3,ZNF316,PER4,IL6,KDELR2,HOXA1,KLHL7-AS1,ICA1,OSBPL3,LOC101927668,C7orf26,NUPL2,NPVF,CCZ1,CYTH3,PMS2CL,ABCB5,C1GALT1,ZNF853,TWIST1,LRRC72,TWISTNB,RPL23P8,ANKRD61,ZNF815P,LOC101927354,NDUFA4,MIR3683,BZW2	3	gain
987	CGTE_14	7	27147499	27150286	7p15.2	HOXA3	6	gain
988	CGTE_14	7	27238756	27283080	7p15.2	HOTTIP,EVX1-AS,EVX1,HOXA13	9	gain
989	CGTE_14	7	44924502	45002733	7p13	MYO1G,PURB	0	loss
990	CGTE_14	7	73038481	73083978	7q11.23	MLXIPL,VPS37D	0	loss
991	CGTE_14	7	76022756	76030482	7q11.23	ZP3,SSC4D	3	gain
992	CGTE_14	7	99817517	99818496	7q22.1	PVRIG,GATS	182	gain
993	CGTE_14	7	150715229	150716625	7q36.1	ATG9B	5	gain
994	CGTE_14	8	53626595	53853490	8q11.23	NPBWR1,RB1CC1	5	gain
995	CGTE_14	8	144462802	144464107	8q24.3	RHPN1	6	gain
996	CGTE_14	8	144642315	144662755	8q24.3	MROH6,NAPRT,GSDMD,EEF1D	3	gain
997	CGTE_14	8	145106141	145107489	8q24.3	OPLAH	10	gain
998	CGTE_14	8	145772472	145806697	8q24.3	ARHGAP39	0	loss
999	CGTE_14	9	96210751	96215054	9q22.31	FAM120A,FAM120AOS	4	gain
1000	CGTE_14	9	124414069	124461707	9q33.2	DAB2IP	8	gain
1001	CGTE_14	9	124989598	124991118	9q33.2	LHX6	10	gain
1002	CGTE_14	9	139271962	139273930	9q34.3	SNAPC4	12	gain
1003	CGTE_14	9	139276405	139277985	9q34.3	SNAPC4	8	gain
1004	CGTE_14	9	139378808	139400374	9q34.3	NOTCH1,C9orf163	3	gain
1005	CGTE_14	9	139418188	139562839	9q34.3	MIR4674,NALT1,EGFL7,NOTCH1	4	gain
1006	CGTE_14	9	139747560	139752289	9q34.3	MAMDC4	6	gain
1007	CGTE_14	9	139835417	139836980	9q34.3	FBXW5	20	gain
1008	CGTE_14	9	140120446	140127610	9q34.3	RNF224,SLC34A3,CYSRT1	1	loss
1009	CGTE_14	9	140127612	140130900	9q34.3	SLC34A3	0	loss
1010	CGTE_14	9	140351824	140353591	9q34.3	NSMF	7	gain
1011	CGTE_14	10	76994622	77160199	10q22.2	ZNF503-AS1,ZNF503,COMTD1	0	loss
1012	CGTE_14	10	102586739	102672858	10q24.31	SLF2,PAX2	3	gain
1013	CGTE_14	10	114205194	114207293	10q25.2	VTI1A,ZDHHC6	71	gain
1014	CGTE_14	10	129534510	129537196	10q26.2	FOXI2	6	gain
1015	CGTE_14	11	788141	792998	11p15.5	SLC25A22,CEND1	12	gain
1016	CGTE_14	11	824170	825018	11p15.5	PNPLA2	21	gain
1017	CGTE_14	11	1215315	1255724	11p15.5	MUC5AC,MUC5B	3	gain
1018	CGTE_14	11	17741323	17757750	11p15.1	KCNC1,MYOD1	1	loss
1019	CGTE_14	11	64135895	64138222	11q13.1	MIR1237,RPS6KA4	5	gain
1020	CGTE_14	11	65487524	65548032	11q13.1	RNASEH2C,AP5B1	7	gain

1021	CGTE_14	11	67203262	67206151	11q13.2	CORO1B,PTPRCAP	6	gain
1022	CGTE_14	12	49363872	49374483	12q13.12	WNT10B,WNT1	3	gain
1023	CGTE_14	12	63195729	63328560	12q14.2	PPM1H	6	gain
1024	CGTE_14	12	124819031	124821908	12q24.31	NCOR2,MIR6880	4	gain
1025	CGTE_14	12	132401917	132404156	12q24.33	ULK1	4	gain
1026	CGTE_14	12	133158221	133160392	12q24.33	MIR6763,FBRSL1	86	gain
1027	CGTE_14	13	52004448	52026733	13q14.3	INTS6	6	gain
1028	CGTE_14	13	60737611	61013821	13q21.2	TDRD3,DIAPH3,LINC00434	5	gain
1029	CGTE_14	13	78235542	78272705	13q22.3	LOC100129307,SLAIN1,MIR3665	0	loss
1030	CGTE_14	13	78273082	95095850	13q31.2-q22.3	MIR622,MIR17HG,MIR20A,LINC00380,LINC01047,LINC00353,LINC00446,GPC6,LINC00397,LINC00351,LINC00363,SLITRK6,MIR18A,LINC00559,LINC01038,MIR4500HG,LINC00333,RNF219,SPRY2,MIR92A1,LINC01040,LINC00377,DCT,SLAIN1,GPC6-AS2,MIR4500,MIR19B1,GPC5-AS1,LINC00331,LINC01069,LINC00564,RBM26-AS1,LOC105370306,SNORA107,MIR19A,LINC00382,GPC5-AS2,SLITRK1,LINC00433,EDNRB,RNF219-AS1,LINC00375,LINC00379,EDNRB-AS1,POU4F1,LINC01068,LINC01080,GPC6-AS1,RBM26,LINC00440,MIR17,NDFIP2-AS1,SLITRK5,LINC00410,NDFIP2,LINC01049,LINC00430,GPC5	3	gain
1031	CGTE_14	13	96293503	99108987	13q32.2-q32.1	FARP1,STK24,IPO5,DNAJC3-AS1,MIR3170,MIR4501,HS6ST3,DZIP1,LINC00359,DNAJC3,RNF113B,MBNL2,OXGR1,LINC00456,UGGT2,RAP2A	3	gain
1032	CGTE_14	13	107219911	110408720	13q33.3-q34	LINC00551,LINC00443,FAM155A-IT1,TNFSF13B,IRS2,MYO16-AS1,MYO16,LIG4,LINC00399,MIR1267,LINC00676,ABHD13,FAM155A,ARGLU1	3	gain
1033	CGTE_14	13	113817263	114524037	13q34	CUL4A,MIR8075,PROZ,TMEM255B,DCUN1D2,GAS6-AS1,TFDP1,TMCO3,LINC00552,ATP4B,GRTP1,GRTP1-AS1,LAMP1,LOC101928841,GAS6,PCID2,GRK1,ADPRHL1	3	gain
1034	CGTE_14	14	59113300	75759134	14q23.3-q24.3	GPHN,RPS6KL1,SPTB,HIF1A-AS2,FAM161B,PAPLN,LRRC9,DLST,RAD51B,PTGR2,YLPM1,LINC01500,SMOC1,LIN52,PPM1A,DCAF4,ZBTB1,SALRNA1,ZFYVE1,DCAF5,CHURC1-FNTB,AKAP5,SLC39A9,HIF1A-AS1,PCNXL4,MLH3,ACOT1,FCF1,MNAT1,LINC01269,PROX2,CCDC175,ZFP36L1,ADAM20,LINC00643,ATP6V1D,TMEM30B,FAM71D,RTN1,SNAPC1,ELMSAN1,LOC100128233,LTBP2,PPP2R5E,CHURC1,SRSF5,MIR4709,SUSD6,ADAM21,ACOT6,PPP1R36,DAAM1,SNORD56B,GALNT16,RDH11,RAB15,LOC102723809,SYNE2,RDH12,ISCA2,EXD2,EIF2B2,SYNJ2BP,SIX4,ACTN1-AS1,HIF1A,ESR2,MIR625,MIR4706,LINC00644,CCDC177,ABCD4,ACTN1,LOC101928075,ACOT2,TRMT5,ENTPD5,NUMB,TEX21P,TTC9,PCNX,NPC2,TMED10,GPR135,PLEKHD1,MIR7843,SLC10A1,PSEN1,ALDH6A1,MPP5,BBOF1,EIF2S1,LOC100506321,SIX1,JKAMP,COQ6,SYT16,C14orf39,HEATR4,VTI1B,SLC8A3,SYNJ2BP-COX16,COX16,ADAM20P1,ERH,FUT8-AS1,KCNH5,ARG2,ADAM21P1,ACYP1,HSPA2,PLEKHG3,GPHB5,L3HYPDH,NEK9,VRTN,LINC00238,SYNDIG1L,FNTB,ZNF410,PLEK2,FOS,DPF3,SIPA1L1,WDR89,DHRS7,SLC38A6,MAP3K9,LOC101927780,DACT1,RHOJ,MIR548H1,C14orf169,SIX6,FLJ22447,PRKCH,MTHFD1,GPX2,RBM25,PGF,LOC145474,SGPP1,MED6,RGS6,MIR4708,DNAL1,AREL1,MIR548AZ,PLEKHH1,VSX2,MAX,TMEM229B,ZBTB25,MIR4505,PIGH,FUT8,ZC2HC1C,PNMA1,LOC100506476,MIR7855,LOC100289511,ACOT4,ZFYVE26	3	gain
1035	CGTE_14	14	104577768	104641459	14q32.33	ASPG,KIF26A,MIR203B,MIR203A	3	gain
1036	CGTE_14	14	104641463	105052865	14q32.33	C14orf180,KIF26A	1	loss

1037	CGTE_14	15	62352431	62360851	15q22.2	VPS13C,C2CD4A	6	gain
1038	CGTE_14	15	74284411	74315825	15q24.1	STOML1,PML	3	gain
1039	CGTE_14	16	1832841	1841830	16p13.3	IGFALS,NUBP2	8	gain
1040	CGTE_14	16	1841834	1843741	16p13.3	IGFALS	0	loss
1041	CGTE_14	16	30537579	30544381	16p11.2	ZNF768,ZNF747	10	gain
1042	CGTE_14	16	67211927	67212627	16q22.1	KIAA0895L	29	gain
1043	CGTE_14	16	88502594	88504034	16q24.2	ZNF469	11	gain
1044	CGTE_14	16	89763212	89764739	16q24.3	SPATA2L	7	gain
1045	CGTE_14	17	6003	707099	17p13.3	RPH3AL,FAM57A,GEMIN4,FAM101B,VPS53,LOC100506371,DOC2B,RNMTL1,DBIL5P,NXN,LOC100506388,GLOD4,C17orf97	3	gain
1046	CGTE_14	17	1303229	2274678	17p13.3	SRR,INPP5K,PITPNA-AS1,SLC43A2,SMG6,OVCA2,SERPINF1,YWHAE,TLCD2,LOC101927839,SCARF1,TSR1,RTN4RL1,MIR132,HIC1,SNORD91A,MIR212,CRK,SMYD4,WDR81,PITPNA,SNORD91B,DPH1,SGSM2,SERPINF2,MIR22HG,RPA1,RILP,PRPF8,MIR22,MYO1C	3	gain
1047	CGTE_14	17	39165202	39296795	17q21.2	KRTAP4-6,KRTAP4-12,KRTAP4-9,KRTAP4-8,KRTAP3-1,KRTAP2-2,KRTAP2-3,KRTAP1-3,KRTAP1-1,KRTAP4-11,KRTAP1-4,KRTAP2-1,KRTAP1-5,KRTAP4-7,KRTAP2-4	3	gain
1048	CGTE_14	17	39305513	39316719	17q21.2	KRTAP4-5,KRTAP4-4	6	gain
1049	CGTE_14	17	40705158	40706686	17q21.2	HSD17B1	5	gain
1050	CGTE_14	17	47554300	47583971	17q21.33	LOC100288866,NGFR	6	gain
1051	CGTE_14	17	63532430	63533301	17q24.1	AXIN2	4	gain
1052	CGTE_14	17	70117549	70119154	17q24.3	SOX9	5	gain
1053	CGTE_14	17	74071017	74073560	17q25.1	GALR2	4	gain
1054	CGTE_14	17	76113303	76114031	17q25.3	TMC6	8	gain
1055	CGTE_14	17	76115042	76120255	17q25.3	TMC6	1	loss
1056	CGTE_14	17	76120518	76121988	17q25.3	TMC6	4	gain
1057	CGTE_14	17	79077357	79092323	17q25.3	AATK,BAIAP2	1	loss
1058	CGTE_14	17	79100234	79106597	17q25.3	AATK	8	gain
1059	CGTE_14	17	79173468	79411164	17q25.3	C17orf89,ENTHD2,LOC100130370,SLC38A10,LINC00482,MIR4740,TMEM105,BAHCC1,CEP131	1	loss
1060	CGTE_14	18	24127679	24128654	18q11.2	KCTD1	7	gain
1061	CGTE_14	18	51750376	51796246	18q21.2	POLI,MBD2	5	gain
1062	CGTE_14	18	76740231	76754997	18q23	SALL3	7	gain
1063	CGTE_14	19	1064787	1079914	19p13.3	HMHA1,ABCA7	1	loss
1064	CGTE_14	19	3595703	3600671	19p13.3	TBXA2R	7	gain
1065	CGTE_14	19	7676369	7677671	19p13.2	CAMSAP3	23	gain
1066	CGTE_14	19	7795141	7796111	19p13.2	CLEC4G	11	gain
1067	CGTE_14	19	14590152	14593844	19p13.12	GIPC1	7	gain
1068	CGTE_14	19	39663444	39666158	19q13.2	PAK4	10	gain
1069	CGTE_14	19	49131996	49133049	19q13.33	SPHK2	5	gain
1070	CGTE_14	19	50370224	50376503	19q13.33	PNKP,AKT1S1	7	gain
1071	CGTE_14	20	3765656	3767104	20p13	CENPB	17	gain
1072	CGTE_14	20	60893431	60894979	20q13.33	LAMA5	6	gain
1073	CGTE_14	20	62166220	62172360	20q13.33	SRMS,PTK6	6	gain
1074	CGTE_14	21	47565323	47581413	21q22.3	FTCD,SPATC1L	3	gain
1075	CGTE_14	22	28192793	28196103	22q12.1	MN1	4	gain
1076	CGTE_14	22	39773554	39795936	22q13.1	SYNGR1,TAB1	10	gain
1077	CGTE_14	22	50895501	50898014	22q13.33	SBF1	5	gain
1078	CGTE_14	22	50898019	50898760	22q13.33	SBF1	0	loss
1079	CGTE_14	X	47003825	47030498	Xp11.23	NDUFB11,RBM10	5	gain

1080	CGTE_14	X	150396052	150565998	Xq28	VMA21	20	gain
1081	CGTE_15	1	861266	1154087	1p36.33	MIR429,TTLL10,TNFRSF4,MIR200B,HES4,C1orf159,PLEKHN1,SDF4,KLHL17,PERM1,AGRN,TNFRSF18,NOC2L,ISG15,RNF223,MIR200A,SAMD11,LINC01342	1	loss
1082	CGTE_15	1	3383677	3477362	1p36.32	ARHGEF16,MEGF6,MIR551A	1	loss
1083	CGTE_15	1	6445517	6537717	1p36.31	ACOT7,PLEKHG5,ESPN,TNFRSF25,MIR4252,HES2	1	loss
1084	CGTE_15	1	16261499	16475571	1p36.13	CLCNKA,SPEN,ZBTB17,HSPB7,FAM131C,CLCNKB,EPHA2,C1orf64	1	loss
1085	CGTE_15	1	109810418	109816453	1p13.3	CELSR2	1	loss
1086	CGTE_15	1	150524563	150533880	1q21.3	ADAMTSL4,ADAMTSL4-AS1	1	loss
1087	CGTE_15	1	156877685	156880176	1q23.1	PEAR1	0	loss
1088	CGTE_15	2	128381736	128412192	2q14.3	LIMS2,MYO7B,GPR17	1	loss
1089	CGTE_15	2	196791124	196852946	2q32.3	DNAH7	3	gain
1090	CGTE_15	2	233194365	233414508	2q37.1	ALPI,ALPPL2,ECEL1P2,CHRND,ALPP,CHRNG,DIS3L2,TIGD1,PRSS56,ECEL1	1	loss
1091	CGTE_15	2	241627709	241980267	2q37.3	LOC200772,AGXT,C2orf54,AQP12A,KIF1A,SNED1	1	loss
1092	CGTE_15	2	242738454	242842580	2q37.3	RTP5,NEU4,GAL3ST2,PDCD1,LINC01237	1	loss
1093	CGTE_15	3	52469491	52562758	3p21.1	SEMA3G,NISCH,NT5DC2,TNNC1,STAB1	1	loss
1094	CGTE_15	4	431184	435956	4p16.3	ZNF721,ABCA11P	4	gain
1095	CGTE_15	4	2749465	2826493	4p16.3	SH3BP2,TNIP2	1	loss
1096	CGTE_15	4	10079395	10080667	4p16.1	MIR3138,WDR1	0	loss
1097	CGTE_15	4	69816725	69870628	4q13.2	UGT2B10,UGT2A3	3	gain
1098	CGTE_15	4	103644985	103681849	4q24	MANBA	0	loss
1099	CGTE_15	6	30859818	30863284	6p21.33	DDR1	0	loss
1100	CGTE_15	6	32013871	32049418	6p21.33	TNXB	1	loss
1101	CGTE_15	6	33416529	33419732	6p21.32	SYNGAP1	0	loss
1102	CGTE_15	7	44146371	44193101	7p13	POLD2,AEBP1,GCK,MIR4649,MYL7	1	loss
1103	CGTE_15	7	73008617	73012279	7q11.23	MLXIPL	0	loss
1104	CGTE_15	7	149476529	149523373	7q36.1	SSPO	1	loss
1105	CGTE_15	7	150553918	150839653	7q36.1	ATG9B,AGAP3,FASTK,ASIC3,AOC1,NOS3,CDK5,ABCB8,KCNH2,SLC4A2,TMUB1	1	loss
1106	CGTE_15	8	143808726	143832748	8q24.3	THEM6,LYPD2,SLURP1	1	loss
1107	CGTE_15	8	144775803	145690505	8q24.3	MROH1,HGH1,BOP1,MIR6846,MIR6893,CYC1,TONSL,FAM83H-AS1,SPATC1,MIR661,SCX,NRBP2,MIR7112,SCRIB,MIR6849,EPPK1,DGAT1,SLC39A4,ADCK5,MAF1,SHARPIN,FBXL6,MIR4664,MIR6848,ZNF707,BREA2,SCRT1,MIR6845,CYHR1,PARP10,FAM83H,PLEC,MAPK15,CPSF1,SLC52A2,TONSL-AS1,MIR937,MIR6847,GRINA,CCDC166,WDR97,HSF1,VPS28,PUF60,OPLAH,EXOSC4,GPAA1,MIR939,TMEM249	1	loss
1108	CGTE_15	8	145694713	145745558	8q24.3	PPP1R16A,LRRC14,KIFC2,FOXH1,MFSD3,GPT,RECQL4	0	loss
1109	CGTE_15	9	126128171	126146225	9q33.3	DENND1A,CRB2	1	loss
1110	CGTE_15	9	139820118	140392723	9q34.3	LRRC26,C9orf142,MIR7114,MAN1B1,NSMF,MIR3621,NOXA1,GRIN1,FAM166A,C9orf173-AS1,TUBB4B,SSNA1,TPRN,LCN12,FUT7,C9orf139,NPDC1,TMEM203,NDOR1,CLIC3,ABCA2,ANAPC2,TOR4A,PNPLA7,TRAF2,DPP7,C9orf173,ENTPD2,LCNL1,FBXW5,PTGDS,UAP1L1,RNF224,RNF208,NELFB,SAPCD2,MAN1B1-AS1,TMEM210,EXD3,NRARP,SLC34A3,CYSRT1,C8G,ENTPD8	1	loss
1111	CGTE_15	10	18795375	20466077	10p12.31	ARL5B,LOC101928834,NSUN6,MALRD1,CACNB2,PLXDC2	3	gain
1112	CGTE_15	10	43597796	43604660	10q11.21	RET	0	loss
1113	CGTE_15	11	63990528	64387974	11q13.1	ESRRA,FKBP2,MIR1237,DNAJC4,KCNK4,PPP1R14B,NRXN2,PRDX5,RPS6KA4,SLC22A12,LOC100996455,KCNK4-TEX40,SLC22A11,TRMT112,TEX40,BAD,GPR137,CCDC88B,VEGFB,PLCB3,MIR7155,TRPT1,NUDT22,FERMT3	1	loss
1114	CGTE_15	12	48369099	48377642	12q13.11	COL2A1	1	loss
1115	CGTE_15	12	124832366	124839034	12q24.31	NCOR2	0	loss

1116	CGTE_15	14	73712217	73735366	14q24.2	PAPLN	1	loss
1117	CGTE_15	14	103478622	103596189	14q32.32	TNFAIP2,EXOC3L4,CDC42BPB	1	loss
1118	CGTE_15	14	105238565	105418727	14q32.33	LINC00638,PLD4,CEP170B,AKT1,AHNAK2,ZBTB42	1	loss
1119	CGTE_15	15	25422061	25584361	15q11.2	SNORD115-42,SNORD115-14,SNORD115-30,SNORD115-12,SNORD115-11,SNORD115-29,SNORD115-5,SNORD115-23,SNORD115-28,SNORD115-44,SNORD115-19,SNORD115-8,SNORD115-32,SNORD115-27,SNORD115-46,SNORD115-37,SNORD115-15,SNORD115-24,SNORD115-16,SNORD115-9,SNORD115-17,SNORD115-45,SNORD115-31,SNORD115-26,SNORD115-34,SNORD115-35,SNORD115-41,SNORD115-6,PWAR4,SNORD115-21,SNORD115-38,SNORD115-39,SNORD115-7,SNORD115-40,UBE3A,SNORD109A,SNORD115-47,SNORD115-18,SNORD115-13,SNORD109B,SNORD115-36,SNORD115-48,SNORD115-22,SNORD115-20,SNORD115-25,SNORD115-43,SNORD115-10,SNORD115-33	1	loss
1120	CGTE_15	15	74336375	74472662	15q24.1	ISLR2,GOLGA6A,PML,ISLR,STRA6,LOC283731	0	loss
1121	CGTE_15	15	75977640	75983187	15q24.2	CSPG4	1	loss
1122	CGTE_15	15	90174779	90210971	15q26.1	PLIN1,KIF7	1	loss
1123	CGTE_15	15	91421368	91422790	15q26.1	FURIN	1	loss
1124	CGTE_15	16	287186	1578698	16p13.3	FAM234A,MIR662,ARHGDIG,CCDC78,PDIA2,C16orf91,LOC100134368,METRN,MIR3176,BAIAP3,UNKL,RAB11FIP3,LMF1-AS1,FBXL16,UBE2I,GNG13,JMJD8,FAM173A,GNPTG,WFIKKN1,NARFL,C16orf13,PRR35,SSTR5-AS1,SOX8,MRPL28,DECR2,IFT140,TPSB2,LMF1,RPUSD1,RGS11,MSLN,NME4,NHLRC4,LINC00235,WDR90,CACNA1H,RAB40C,SSTR5,TSR3,TPSAB1,RHBDL1,CLCN7,C1QTNF8,TMEM8A,RHOT2,AXIN1,CHTF18,TPSD1,TELO2,HAGHL,FAM195A,PIGQ,CCDC154,CAPN15,PTX4,TPSG1,STUB1,MIR5587,WDR24,PRR25	1	loss
1125	CGTE_15	17	4389332	4463789	17p13.2	SPNS3,MYBBP1A,GGT6,SPNS2	1	loss
1126	CGTE_15	17	48213181	48272996	17q21.33	COL1A1,PPP1R9B,HILS1,SGCA	1	loss
1127	CGTE_15	19	44871176	46888262	19q13.31-q13.32	OPA3,CEACAM20,CD3EAP,MIR642A,KLC3,MIR642B,FOXA3,PVR,VASP,SIX5,CKM,PPM1N,PPP1R13L,TOMM40,PPP1R37,DMPK,MIR769,EXOC3L2,EML2-AS1,NOVA2,ZNF229,MYPOP,PPP5C,QPCTL,RELB,NANOS2,LOC400706,BCL3,SNRPD2,ZNF285,EML2,TRAPPC6A,BLOC1S3,BHMG1,CBLC,APOC1P1,CEACAM16,CLASRP,BCAM,MARK4,CCDC61,MIR4531,APOC4,MIR330,IGFL2,ZNF180,HIF3A,FBXO46,NKPD1,CLPTM1,PGLYRP1,DKFZp434J0226,GPR4,CEACAM22P,IRF2BP1,MIR6088,MIR8085,ERCC1,APOC4-APOC2,IGFL1,GEMIN7,RTN2,PVRL2,SYMPK,ZNF296,IGSF23,CEACAM19,APOE,IGFL3,ERCC2,IGFL4,APOC2,FOSB,GIPR,DMWD,RSPH6A,APOC1	3	gain
1128	CGTE_15	19	55855038	56172560	19q13.42	SHISA7,ZNF581,TMEM238,ZNF865,SSC5D,FIZ1,SBK2,U2AF2,KMT5C,COX6B2,ZNF580,CCDC106,ZNF579,SBK3,MIR6805,UBE2S,ZNF784,ZNF524,FAM71E2,TMEM190,NAT14,IL11,ZNF628,ISOC2,RPL28	1	loss
1129	CGTE_15	20	3180580	3214697	20p13	SLC4A11,DDRGK1,ITPA	1	loss
1130	CGTE_15	21	45714266	45755728	21q22.3	C21orf2,PFKL,AIRE	1	loss
1131	CGTE_15	21	45970688	46117586	21q22.3	TSPEAR,KRTAP10-4,KRTAP10-8,KRTAP12-4,KRTAP12-2,KRTAP12-3,KRTAP10-5,KRTAP10-10,KRTAP10-9,KRTAP10-7,KRTAP10-11,KRTAP12-1,KRTAP10-3,KRTAP10-6,KRTAP10-12,KRTAP10-2	1	loss
1132	CGTE_15	21	47361519	47575502	21q22.3	COL6A2,PCBP3,COL6A1,LOC101928796,FTCD	1	loss
1133	CGTE_15	22	19119588	19164797	22q11.21	TSSK2,SLC25A1,DGCR14,LINC01311,GSC2	1	loss
1134	CGTE_15	22	20126954	20231166	22q11.21	RTN4R,LOC284865,ZDHHC8,CCDC188,LINC00896	0	loss
1135	CGTE_15	22	38017499	38038684	22q13.1	LOC101927051,GGA1,SH3BP1	1	loss
1136	CGTE_15	X	153678592	153698971	Xq28	PLXNA3,FAM50A,MIR6858	0	loss
1137	CGTE_16	1	1560821	1564479	1p36.33	MIB2	1	loss

1138	CGTE_16	1	33646628	33747382	1p35.1	ZNF362,TRIM62	1	loss
1139	CGTE_16	1	35851015	35851814	1p34.3	ZMYM4	4	gain
1140	CGTE_16	1	115428140	115430420	1p13.2	SYCP1	3	gain
1141	CGTE_16	2	11427774	11590337	2p25.1	E2F6,LINC00570,ROCK2	3	gain
1142	CGTE_16	2	73052921	73114960	2p13.2	EXOC6B,SPR	0	loss
1143	CGTE_16	2	74734693	74743597	2p13.1	TLX2,PCGF1	0	loss
1144	CGTE_16	2	171570087	171573960	2q31.1	LOC101926913,LINC01124,SP5	1	loss
1145	CGTE_16	2	219757393	219847166	2q35	LINC01494,WNT10A,LINC00608,CDK5R2,FEV	0	loss
1146	CGTE_16	2	239009003	239051663	2q37.3	ESPNL,KLHL30	1	loss
1147	CGTE_16	3	48698495	48700037	3p21.31	CELSR3	1	loss
1148	CGTE_16	3	138478010	138763157	3q23-q22.3	LINC01391,PRR23A,FOXL2,FOXL2NB,PIK3CB,PRR23B,PRR23C	1	loss
1149	CGTE_16	4	954336	1020758	4p16.3	IDUA,SLC26A1,DGKQ,FGFRL1	1	loss
1150	CGTE_16	4	1795112	1809470	4p16.3	FGFR3	1	loss
1151	CGTE_16	4	3768419	3769730	4p16.3	ADRA2C	1	loss
1152	CGTE_16	4	13480954	13544271	4p15.33	RAB28,NKX3-2,LINC01097	1	loss
1153	CGTE_16	5	140167086	140263947	5q31.3	PCDHA7,PCDHA6,PCDHA8,PCDHA4,PCDHA3,PCDHA13,PCDHA10,PCDHA9,PCDHA12,<br>PCDHA2,PCDHA1,PCDHA5,PCDHA11	1	loss
1154	CGTE_16	5	176023753	176025158	5q35.2	GPRIN1	1	loss
1155	CGTE_16	6	29795542	29912461	6p22.1	HLA-G,HCG4B,HLA-A,HLA-H	1	loss
1156	CGTE_16	6	30993283	30997476	6p21.33	MUC22	1	loss
1157	CGTE_16	6	31324110	31371612	6p21.33	MICA,HLA-B	1	loss
1158	CGTE_16	6	43227286	43253116	6p21.1	TTBK1	1	loss
1159	CGTE_16	6	127794344	127837673	6q22.33	SOGA3	1	loss
1160	CGTE_16	9	140120072	140137625	9q34.3	RNF224,SLC34A3,TUBB4B,CYSRT1	1	loss
1161	CGTE_16	10	27702744	27793808	10p12.1	PTCHD3,RAB18	1	loss
1162	CGTE_16	10	35896546	36813201	10p11.21	GJD4,MIR4683,PCAT5,FZD8	1	loss
1163	CGTE_16	10	76994109	77161387	10q22.2	COMTD1,ZNF503,ZNF503-AS1,ZNF503-AS2	1	loss
1164	CGTE_16	10	102893995	102988367	10q24.31	TLX1,LBX1,LINC01514,TLX1NB	1	loss
1165	CGTE_16	11	823474	825018	11p15.5	PNPLA2	1	loss
1166	CGTE_16	11	13030596	13032710	11p15.2	RASSF10	1	loss
1167	CGTE_16	11	17351714	17353070	11p15.1	NUCB2	3	gain
1168	CGTE_16	11	63682829	63684158	11q13.1	RCOR2	0	loss
1169	CGTE_16	11	64126712	64139065	11q13.1	RPS6KA4,MIR1237	1	loss
1170	CGTE_16	12	40876490	40885045	12q12	MUC19	1	loss
1171	CGTE_16	13	38934712	39262135	13q13.3	LINC00437,UFM1,LINC00366,FREM2	1	loss
1172	CGTE_16	13	95899908	95953573	13q32.1	ABCC4	3	gain
1173	CGTE_16	14	54907906	54946497	14q22.2	GMFB,CNIH1	1	loss
1174	CGTE_16	14	71275477	71374746	14q24.2	MAP3K9,PCNX	1	loss
1175	CGTE_16	14	103987257	103988824	14q32.32	CKB	0	loss
1176	CGTE_16	14	105398113	105419610	14q32.33	PLD4,AHNAK2	1	loss
1177	CGTE_16	14	105609769	105617483	14q32.33	MIR6765,JAG2	1	loss
1178	CGTE_16	16	704987	706503	16p13.3	WDR90	1	loss
1179	CGTE_16	16	23724343	23766536	16p12.2	ERN2,CHP2	0	loss
1180	CGTE_16	16	30537579	30581364	16p11.2	ZNF768,ZNF764,ZNF747,ZNF688	1	loss
1181	CGTE_16	16	30679689	30681040	16p11.2	FBRS	1	loss
1182	CGTE_16	16	67211927	67212627	16q22.1	KIAA0895L	0	loss
1183	CGTE_16	16	88493912	88505649	16q24.2	ZNF469	1	loss

1184	CGTE_16	17	26088086	28499711	17q11.2	MYO18A,CORO6,SNORD4A,EFCAB5,ERAL1,RPL23A,NLK,PIPOX,MIR4523,FAM222B,TP53I13,ANKRD13B,PROCA1,TLCD1,LOC101927018,ABHD15,TRAF4,SNORD4B,MIR4732,MIR144,TIAF1,GIT1,IFT20,POLDIP2,SGK494,PYY2,MIR423,UNC119,FOXN1,SLC46A1,SUPT6H,FLOT2,NEK8,SPAG5,SDF2,SNORD42A,KIAA0100,TMEM97,MIR451B,SSH2,SEBOX,NUFIP2,SPAG5-AS1,LYRM9,KRT18P55,SLC13A2,ALDOC,PHF12,TAOK1,PPY2P,TMEM199,VTN,NSRP1,TNFAIP1,PIGS,MIR3184,MIR4723,SEZ6,DHRS13,SNORD42B,RAB34,CRYBA1,SARM1,NARR,NOS2,MIR451A	3	gain
1185	CGTE_16	17	35307559	36214044	17q12	AATF,C17orf78,MIR2909,SYNRG,DDX52,MIR378J,HNF1B,ACACA,TADA2A,SNORA90,YWHAEP7,DUSP14	3	gain
1186	CGTE_16	17	36474565	36709175	17q12	ARHGAP23,MRPL45,GPR179,SOCS7,SRCIN1	4	gain
1187	CGTE_16	17	36714398	36716076	17q12	SRCIN1	1	loss
1188	CGTE_16	17	36716150	39165197	17q21.1-q21.2	SNORD124,KRT26,KRT20,TCAP,WIPF2,SMARCE1,MIR4728,MSL1,CASC3,KRT39,MED24,IGFBP4,NR1D1,PCGF2,LRRC37A11P,PGAP3,FBXL20,MIEN1,GJD3,CWC25,CSF3,C17orf96,CACNB1,MLLT6,KRT23,KRT12,CDC6,MIR4727,LASP1,FBXO47,CISD3,RPL19,MIR6884,STAC2,C17orf98,ARL5C,CDK12,ZPBP2,KRT28,SRCIN1,PSMB3,PPP1R1B,RPL23,KRTAP3-2,ORMDL3,GSDMA,LINC00672,KRT25,MIR6779,PLXDC1,LOC100131347,IKZF3,RARA-AS1,GSDMB,TMEM99,MIR4734,MIR6866,PSMD3,PNMT,KRT40,KRT27,PIP4K2B,ERBB2,MIR6867,RAPGEFL1,KRTAP3-3,KRT10,LRRC3C,GRB7,MIR4726,RARA,MED1,KRTAP3-1,CCR7,NEUROD2,TOP2A,STARD3,THRA,KRT222,SNORA21,TNS4,KRT24	4	gain
1189	CGTE_16	18	18540055	18595573	18q11.1	ROCK1	3	gain
1190	CGTE_16	19	993923	1012048	19p13.3	GRIN3B,TMEM259,WDR18	1	loss
1191	CGTE_16	19	48182423	48198463	19q13.33	GLTSCR1	0	loss
1192	CGTE_16	20	4202161	4229364	20p13	ADRA1D	1	loss
1193	CGTE_16	20	44518858	44520452	20q13.12	CTSA,NEURL2	1	loss
1194	CGTE_16	20	60908052	60909856	20q13.33	LAMA5	1	loss
1195	CGTE_16	22	17589465	17590650	22q11.1	IL17RA	1	loss
1196	CGTE_16	22	28192793	28196535	22q12.1	MN1	1	loss
1197	CGTE_16	22	30899667	30901629	22q12.2	SEC14L4	1	loss
1198	CGTE_16	22	39077900	39078343	22q13.1	TOMM22	1	loss
1199	CGTE_16	22	42525115	42539180	22q13.2	CYP2D6,CYP2D7,LOC101929829	1	loss
1200	CGTE_16	22	50609165	50631721	22q13.33	PANX2,TRABD	1	loss
1201	CGTE_16	22	50705363	50706398	22q13.33	MAPK11	0	loss
1202	CGTE_16	Y	14847312	14847705	Yq11.21	USP9Y	0	loss
1203	CGTE_17	1	38259876	38260423	1p34.3	MANEAL	11	gain
1204	CGTE_17	1	50460939	50514009	1p33	AGBL4,ELAVL4	5	gain
1205	CGTE_17	1	152009228	153122682	1q21.3	LCE3C,TCHHL1,LCE2C,LOC100131107,SPRR1B,FLG-AS1,SPRR3,SPRR1A,LCE5A,SPRR4,IVL,LCE6A,LCE1C,SPRR2B,LCE3A,FLG,SPRR2E,HRNR,LCE1A,C1orf68,KPRP,LCE2B,SPRR2A,LCE3D,LCE2A,CRCT1,CRNN,LCE3B,S100A11,LCE1F,SPRR2C,RPTN,LCE1B,SPRR2F,TCHH,LCE4A,SPRR2G,LCE2D,SMCP,LCE1D,LCE3E,SPRR2D,LCE1E,FLG2	3	gain
1206	CGTE_17	1	157489448	159683996	1q23.2-q23.1	CD1A,OR10X1,CD1D,OR10J5,OR10K1,OR6N2,CD1C,APCS,AIM2,OR6K3,OR6P1,FCRL1,OR6K2,FCER1A,SPTA1,FCRL4,OR10R2,CD5L,OR6K6,KIRREL,CADM3-AS1,CRP,OR10J3,OR10K2,OR6N1,FCRL2,CADM3,PYHIN1,OR6Y1,CD1B,FCRL5,ACKR1,FCRL3,IFI16,OR10T2,OR10Z1,LOC646268,OR10J1,MNDA,CD1E	3	gain

1207	CGTE_17	1	185278462	200378764	1q31.1-q31.3	CFH,GLRX2,C1orf27,LINC01351,CDC73,ATP6V1G3,RGS21,PLA2G4A,MIR181A1,HMCN1,TPR,CFHR2,CFHR3,MIR548F1,PTGS2,MIR1278,RGS13,BRINP3,PDC,GS1-279B7.1,PACERR,MIR181A1HG,LINC01036,LHX9,MIR4426,IVNS1ABP,NEK7,LINC01221,LINC00862,MIR181B1,ZBTB41,DENND1B,CFHR4,MIR4735,RGS1,LINC01350,LOC102724919,RGS18,C1orf53,F13B,PRG4,LINC01222,CFHR1,UCHL5,OCLM,NR5A2,KCNT2,PTPRC,LINC01037,LOC440704,LINC01031,LINC01032,ZNF281,B3GALT2,CRB1,CFHR5,RGS2,TROVE2,ASPM	3	gain
1208	CGTE_17	1	215754190	220156742	1q41	SPATA17,SPATA17-AS1,LOC102723833,SLC30A10,LOC101929631,EPRS,TGFB2,LYPLAL1-AS1,ESRRG,RRP15,LYPLAL1,TGFB2-OT1,USH2A,MIR548F3,TGFB2-AS1,GPATCH2,RNU5F-1,LINC00210,KCTD3	3	gain
1209	CGTE_17	2	16082129	16083029	2p24.3	MYCN	8	gain
1210	CGTE_17	2	50149070	51253601	2p16.3	MIR8485,NRXN1	3	gain
1211	CGTE_17	2	51254441	51255586	2p16.3	NRXN1	5	gain
1212	CGTE_17	2	74734693	74743597	2p13.1	TLX2,PCGF1	4	gain
1213	CGTE_17	2	74884878	75874029	2p13.1-p12	LINC01291,TACR1,MIR5000,EVA1A,SEMA4F,MRPL19,POLE4,HK2,LOC101927884	3	gain
1214	CGTE_17	2	75937763	84518086	2p12-p11.2	REG3G,LOC101927926,LOC101927948,REG1B,LOC100507201,GCFC2,LRRTM4,LOC1720,MIR8080,REG1A,MIR4264,LOC101927907,LRRTM1,SNAR-H,LOC101927987,CTNNA2,REG1CP,LOC101927967,FUNDC2P2,REG3A	3	gain
1215	CGTE_17	2	187703737	190044422	2q32.2-q32.1	GULP1,MIR1245B,MIR3606,COL5A2,LINC01090,ZSWIM2,COL3A1,MIR561,TFPI,CALCRL,MIR3129,DIRC1,MIR1245A	3	gain
1216	CGTE_17	2	210590395	213921855	2q34	CPS1-IT1,IKZF2,KANSL1L,MIR4776-2,LANCL1,CPS1,LOC102725079,LANCL1-AS1,LOC101928020,MAP2,RPE,UNC80,MIR548F2,MYL1,ERBB4,MIR4776-1,ACADL	3	gain
1217	CGTE_17	3	16450916	19575699	3p24.3	TBC1D5,KCNH8,MIR3714,SATB1-AS1,SATB1,MIR4791,LOC339862,LINC00690,RFTN1,PLCL2,DAZL	3	gain
1218	CGTE_17	3	75790414	97541211	3q11.1-p11.2	DHFRL1,MTHFD2P1,HTR1F,VGLL3,CHMP2B,ZNF654,GBE1,LINC00506,ROBO2,LINC00879,EPHA3,EPHA6,ARL13B,MIR3923,LOC728290,CADM2,MIR4795,NSUN3,ROBO1,CADM2-AS2,ZNF717,POU1F1,MIR6730,ARL6,MIR5688,SNORA95,LINC00971,LOC101927374,C3orf38,CRYBG3,MIR8060,CGGBP1,PROS1,STX19	3	gain
1219	CGTE_17	3	114014556	118866432	3q13.31-q13.32	LINC00901,LSAMP,ZBTB20-AS4,MIR4796,TIGIT,C3orf30,TUSC7,MIR4447,LSAMP-AS1,ZBTB20,LOC101929754,ZBTB20-AS1,GAP43,MIR568,ZBTB20-AS3,IGSF11,IGSF11-AS1	3	gain
1220	CGTE_17	3	138724259	138763157	3q23	PRR23A,PRR23B,PRR23C	5	gain
1221	CGTE_17	3	180707157	181431090	3q26.33	RNU6-2,SOX2,SOX2-OT,DNAJC19,LOC102724604	4	gain
1222	CGTE_17	5	122372167	122435671	5q23.2	PRDM6,PPIC	10	gain
1223	CGTE_17	5	126676202	129520635	5q23.3-q23.2	KIAA1024L,PRRC1,MIR4633,MEGF10,LINC01184,CTXN3,SLC27A6,ADAMTS19,ADAMTS19-AS1,CHSY3,MIR4460,FBN2,SLC12A2,ISOC1	3	gain
1224	CGTE_17	5	140167086	140263292	5q31.3	PCDHA2,PCDHA6,PCDHA3,PCDHA7,PCDHA4,PCDHA13,PCDHA5,PCDHA9,PCDHA12,PCDHA11,PCDHA1,PCDHA8,PCDHA10	4	gain

1225	CGTE_17	7	7470955	19813386	7p21.3-p21.1	ARL4A,LRRC72,UMAD1,MIR1302-6,TWISTNB,AGR3,FERD3L,GLCCI1,AGR2,AGMO,ICA1,DGKB,TSPAN13,BZW2,SCIN,MEOX2,TMEM196,THSD7A,SNX13,RPA3,SOSTDC1,PHF14,LOC100505938,NXPH1,ISPD,LOC101927391,LOC101927630,ANKMY2,PRPS1L1,VWDE,COL28A1,TMEM106B,ISPD-AS1,MEOX2-AS1,PER4,TWIST1,KCCAT333,LOC100505921,MIOS,ETV1,HDAC9,MIR3146,AHR,NDUFA4	3	gain
1226	CGTE_17	7	44923990	44924956	7p13	PURB	7	gain
1227	CGTE_17	7	77648494	97846838	7q21.11-q21.13	SDHAF3,CDK6,ABCB1,MIR653,PON2,SAMD9,CLDN12,PEX1,LOC101927356,FZD1,PON3,LRRD1,SGCE,MIR5692A1,CYP51A1,KIAA1324L,HGF,PDK4,TFPI2,GNG11,CYP51A1-AS1,SAMD9L,KRIT1,LMTK2,MIR489,RBM48,C7orf62,PCLO,DLX5,COL1A2,CDK14,SRI,SEMA3A,AKAP9,BHLHA15,MIR548M,LOC100506136,GNAI1,SHFM1,LOC101927497,MIR591,C7orf76,DLX6-AS1,OCM2,MAGI2-AS2,LOC101927269,LOC102723885,DBF4,SLC25A40,STEAP1,ASB4,RPL13AP17,FAM133DP,CASD1,MTERF1,CROT,GATAD1,PPP1R9A,FAM133B,SEMA3D,GTPBP10,GRM3,ASNS,SEMA3C,STEAP2,DPY19L2P4,MGC16142,MAGI2,CD36,LOC101927378,PON1,ADAM22,ANKIB1,TMEM243,TP53TG1,RUNDC3B,CFAP69,LOC101409256,STEAP2-AS1,STEAP4,LOC100128317,HEPACAM2,TAC1,CACNA2D1,MIR4652,MIR5692A2,DMTF1,VPS50,GNAT3,CALCR,SLC25A13,BET1,DLX6,MAGI2-AS3,ZNF804B,TECPR1,PEG10,ABCB4,SEMA3E,DYNC1I1,MGC72080,GNGT1,LOC101927446	3	gain
1228	CGTE_17	7	101272386	101461087	7q22.1	MYL10,CUX1	34	gain
1229	CGTE_17	7	139477394	139478387	7q34	TBXAS1,HIPK2	38	gain
1230	CGTE_17	8	88624218	94767401	8q21.3-q22.1	LOC102724710,CALB1,TRIQK,FAM92A1,LINC00534,OTUD6B-AS1,OTUD6B,TMEM64,MIR4661,NECAB1,MIR8084,RBM12B,LINC00535,RUNX1T1,LOC101929709,C8orf88,SLC26A7,MMP16,LRRC69,LINC01030,RBM12B-AS1,TMEM67,DCAF4L2,RIPK2,NBN,FLJ46284,OSGIN2,TMEM55A,MIR7641-2,C8orf87,DECR1	3	gain
1231	CGTE_17	8	104341897	124085722	8q22.3-q23.3	TAF2,TRHR,DCAF13,RIMS2,ANGPT1,KCNV1,RAD21,TNFRSF11B,CSMD3,MED30,EIF3H,COL14A1,EIF3E,LOC101927543,ZHX2,EBAG9,RAD21-AS1,LINC00536,ABRA,SAMD12,UTP23,MIR3610,LRP12,CTHRC1,EXT1,RSPO2,MRPL13,TMEM74,OXR1,TBC1D31,PKHD1L1,SAMD12-AS1,SYBU,DPYS,LINC01609,MIR2053,NUDCD1,HAS2,NOV,MTBP,HAS2-AS1,DCSTAMP,COLEC10,ZFPM2-AS1,SLC25A32,SLC30A8,LINC01608,DERL1,MAL2,SNTB1,ENPP2,DSCC1,ENY2,FZD6,EMC2,TRPS1,LOC105375734,DEPTOR,ZFPM2,AARD,LINC01151	3	gain
1232	CGTE_17	8	145772472	145773883	8q24.3	ARHGAP39	7	gain
1233	CGTE_17	10	8097430	9450267	10p14	LINC00708,LOC101928272,LINC00709,GATA3,LOC105755953	4	gain
1234	CGTE_17	10	27702148	27793808	10p12.1	PTCHD3,RAB18	8	gain
1235	CGTE_17	10	35896546	35929717	10p11.21	GJD4,FZD8	7	gain
1236	CGTE_17	10	52005141	69571375	10q21.1-q21.2	LINC00844,LINC01553,REEP3,ANK3,PRKG1-AS1,MBL2,RHOBTB1,RTKN2,LINC01515,MRLN,LOC101928961,FAM133CP,MIR605,CCDC6,LINC00845,TMEM26-AS1,JMJD1C,PHYHIPL,BICC1,ARID5B,CDK1,ASAH2B,DNAJC12,SGMS1-AS1,ZWINT,MIR3924,LOC102724719,MIR7151,ANXA2P3,A1CF,NRBF2,MTRNR2L5,MIR548AV,SGMS1,ADO,LRRTM3,TMEM26,IPMK,LINC01468,CTNNA3,PCDH15,CSTF2T,CCEPR,TFAM,CISD1,FAM13C,ASAH2,SLC16A9,C10orf107,JMJD1C-AS1,LOC283045,EGR2,ZNF365,MIR1296,DKK1,UBE2D1,PRKG1	3	gain

1237	CGTE_17	10	105991630	106015089	10q25.1	CFAP43,GSTO1	5	gain
1238	CGTE_17	11	4409604	6412189	11p15.4	OR56A3,OLFM5P,OR52E2,UBQLNL,OR56B1,C11orf42,TRIM21,HBG2,OR52A1,OR51A2,OR52D1,OR52E8,PRKCDBP,HBG1,OR51G1,OR51Q1,OR51B5,OR51S1,CNGA4,OR51L1,OR51G2,OR51A7,OR52B2,OR52E6,OR51F2,TRIM6-TRIM34,TRIM5,OR52E4,OR52L1,OR52R1,OR52N5,MMP26,OR52N1,OR52N2,TRIM68,C11orf40,HBB,OR52K2,OR51E1,OR52W1,CCKBR,OR52H1,OR51B4,OR52M1,OR52K1,OR56B4,HBBP1,OR51T1,OR56A1,FAM160A2,TRIM34,UBQLN3,OR51D1,OR51M1,SMPD1,TRIM6,OR52A5,HBD,BGLT3,TRIM22,OR51B6,OR52J3,OR51I2,OR56A5,OR51I1,HBE1,OR52B6,OR51A4,OR51V1,OR51B2,OR51F1,OR52I2,OR52N4,OR52I1,OR51E2,OR56A4	3	gain
1239	CGTE_17	11	14541729	16766434	11p15.2-p15.1	LOC102724957,CALCA,PDE3B,SOX6,CYP2R1,CALCB,INSC,C11orf58,PSMA1,MIR6073	3	gain
1240	CGTE_17	11	44606746	45919930	11p11.2	CHST1,DKFZp779M0652,LOC101928812,TSPAN18,MIR7154,LOC399886,SLC35C1,MAPK8IP1,PRDM11,CD82,LOC100507384,SYT13,CRY2,TP53I11,LOC221122	7	gain
1241	CGTE_17	11	45921597	46071079	11p11.2	C11orf94,PHF21A,MAPK8IP1,GYLTL1B,PEX16	5	gain
1242	CGTE_17	11	46098220	47380237	11p11.2	MIR4688,CREB3L1,ZNF408,DGKZ,F2,MDK,C11orf49,ACP2,MADD,PHF21A,MYBPC3,ARHGAP1,HARBI1,MIR3160-2,CHRM4,NR1H3,SPI1,SNORD67,DDB2,MIR3160-1,AMBRA1,PACSIN3,LOC101928943,LOC101928894,CKAP5,LRP4-AS1,MIR6745,ATG13,LRP4,MIR5582,ARFGAP2	4	gain
1243	CGTE_17	11	47380280	47521192	11p11.2	CELF1,SLC39A13,MIR4487,PSMC3,SPI1,RAPSN	6	gain
1244	CGTE_17	11	68747546	70034065	11q13.3	FGF3,LOC338694,ANO1,ORAOV1,MRGPRF,LINC01488,MRGPRF-AS1,CCND1,FGF19,FGF4,MYEOV,ANO1-AS2,MRGPRD,LOC101928443,MIR3164,TPCN2	3	gain
1245	CGTE_17	11	70049550	70349214	11q13.3	PPFIA1,SHANK2,MIR548K,FADD,CTTN	4	gain
1246	CGTE_17	11	123601215	124481679	11q24.1-q24.2	OR4D5,OR10G8,PANX3,OR8A1,OR8D1,VWA5A,OR8B4,OR8B8,OR8B2,OR8D2,OR6T1,OR6X1,OR8B12,OR10G7,OR8G5,OR10S1,OR8G1,OR8B3,OR8D4,TMEM225,OR10G4,OR6M1,ZNF202,OR8G2,OR10G9	3	gain
1247	CGTE_17	12	55233149	56079296	12q13.2	OR6C74,OR9K2,TESPA1,OR6C3,OR2AP1,OR6C2,OR6C76,OR6C4,OR10P1,OR6C6,OR6C75,OR10A7,METTL7B,OR6C65,OR6C68,OR6C1,OR6C70,MUCL1,NEUROD4,ITGA7	3	gain
1248	CGTE_17	12	63195555	64173875	12q14.2	DPY19L2,AVPR1A,PPM1H,TMEM5	4	gain
1249	CGTE_17	14	20201784	20774126	14q11.2	OR4K1,OR4K14,OR4N5,OR11G2,OR4K15,OR4K13,OR4K17,OR4N2,OR4M1,TTC5,OR4L1,OR4K2,OR4Q3,OR11H4,OR4K5,OR11H6	3	gain
1250	CGTE_17	14	21994087	22783359	14q11.2	OR4E1,OR4E2,SALL2,OR10G3,OR10G2	3	gain
1251	CGTE_17	14	24911249	29237816	14q12	FOXG1-AS1,MIR4307HG,GZMB,FOXG1,CTSG,LOC102724890,LINC00645,CMA1,NOVA1,MIR4307,MIR3171,LOC101927045,GZMH,STXBP6,LOC101927062,SDR39U1	3	gain
1252	CGTE_17	14	34268878	34931479	14q13.1	SPTSSA,NPAS3,EGLN3	3	gain
1253	CGTE_17	14	99640502	99642207	14q32.2	BCL11B	13	gain
1254	CGTE_17	15	33010173	33023439	15q13.3	LOC100131315,GREM1	4	gain
1255	CGTE_17	15	62333460	62360851	15q22.2	VPS13C,C2CD4A	6	gain
1256	CGTE_17	16	1660699	1664793	16p13.3	IFT140,CRAMP1	7	gain
1257	CGTE_17	16	2286845	2288033	16p13.3	DNASE1L2	8	gain
1258	CGTE_17	16	30537579	30545692	16p11.2	ZNF747,ZNF768	4	gain
1259	CGTE_17	16	30545859	30581364	16p11.2	ZNF764,ZNF747,ZNF688	3	gain
1260	CGTE_17	16	31439038	31447600	16p11.2	COX6A2,ZNF843	6	gain
1261	CGTE_17	16	52473073	52580647	16q12.1	TOX3	4	gain
1262	CGTE_17	16	59785799	65345479	16q21	MIR4426,APOOP5,LOC101927650,LOC101927580,LINC00922,CDH8,LOC729159,CDH11	3	gain

1263	CGTE_17	16	68609738	68679767	16q22.1	ZFP90,CDH3	12	gain
1264	CGTE_17	16	79633237	80575065	16q23.2	DYNLRB2,LINC01229,LOC102724084,MAF,MAFTRR	5	gain
1265	CGTE_17	17	7491714	7492935	17p13.1	SOX15	8	gain
1266	CGTE_17	17	39149959	39334187	17q21.2	KRTAP4-3,KRTAP4-7,KRTAP4-5,KRTAP3-1,KRTAP2-4,KRTAP1-1,KRTAP4-2,KRTAP2-1,KRTAP4-6,KRTAP4-11,KRTAP4-12,KRTAP1-5,KRTAP2-2,KRTAP4-9,KRTAP1-4,KRTAP4-4,KRTAP3-3,KRTAP2-3,KRTAP4-8,KRTAP3-2,KRTAP1-3	4	gain
1267	CGTE_17	17	39334358	39622071	17q21.2	KRTAP9-1,KRTAP9-4,KRTAP9-6,KRTAP9-2,KRT33A,KRTAP9-7,KRTAP16-1,KRTAP4-2,KRT37,KRTAP9-9,LOC100505782,KRT31,KRT34,KRTAP4-1,KRTAP17-1,KRTAP9-3,KRTAP29-1,KRT32,KRT33B,KRT38,KRTAP9-8	3	gain
1268	CGTE_17	17	56833320	56833928	17q22	PPM1E	19	gain
1269	CGTE_17	18	3451873	5892041	18p11.31	MIR3976HG,EPB41L3,LINC00667,GAPLINC,C18orf42,TGIF1,MIR3976,ZBTB14,DLGAP1-AS3,DLGAP1-AS1,LINC00526,DLGAP1-AS4,DLGAP1-AS5,MIR6718,DLGAP1-AS2,DLGAP1,TMEM200C	3	gain
1270	CGTE_17	19	3751148	3754411	19p13.3	APBA3,MIR1268A	15	gain
1271	CGTE_17	19	14073337	14074878	19p13.12	RFX1	2306	gain
1272	CGTE_17	19	48673389	48690022	19q13.33	C19orf68,LIG1	6	gain
1273	CGTE_17	19	51601784	51607961	19q13.41	CTU1	114	gain
1274	CGTE_17	20	37554943	44258455	20q11.23-q13.12	PABPC1L,MIR6812,SEMG2,MAFB,SPINT3,SGK2,HNF4A-AS1,WFDC8,OSER1,KCNK15-AS1,PIGT,SLPI,WFDC5,LOC101927159,TOX2,MIR6871,FITM2,EPPIN-WFDC6,PKIG,MYBL2,EPPIN,PI3,HNF4A,SDC4,TP53TG5,YWHAB,WFDC10A,FAM83D,TOMM34,ZHX3,PLCG1-AS1,PTPRT,WISP2,LINC01620,R3HDML,SYS1,GTSF1L,WFDC2,L3MBTL1,WFDC9,SYS1-DBNDD2,SEMG1,TOP1,DBNDD2,LINC01370,MATN4,RIMS4,MIR3646,GDAP1L1,STK4-AS1,WFDC12,LINC01260,IFT52,EMILIN3,OSER1-AS1,WFDC6,SRSF6,PLCG1,LPIN3,LINC01430,ADA,DHX35,STK4,KCNK15,CHD6,LOC339568,JPH2,KCNS1,TTPAL,RBPJL,SERINC3	3	gain
1275	CGTE_17	20	57617751	58545236	20q13.33-q13.32	PRELID3B,ZNF831,PHACTR3,SLMO2-ATP5E,PPP1R3D,FAM217B,LOC100506384,EDN3,SYCP2,CDH26	3	gain
1276	CGTE_17	20	62369118	62489147	20q13.33	ZBTB46,LIME1,SLC2A4RG,ZBTB46-AS1	3	gain
1277	CGTE_17	21	10862592	26978980	21q21.1-p11.2	MRPL39,NRIP1,MIR155,BTG3,MIR125B2,BAGE4,LINC00515,LINC00308,POTED,LOC101927843,MIR99A,LINC00317,LINC00158,LOC339622,MIR3118-1,SAMSN1,CHODL,ABCC13,LIPI,LINC00320,D21S2088E,NCAM2,BAGE2,LOC102724188,LINC01549,SAMSN1-AS1,C21orf91,MIRLET7C,LOC388813,MIR548XHG,C21orf91-OT1,CXADR,MIR8069-2,TPTE,BAGE5,CYP4F29P,LOC101927869,MIR99AHG,HSPA13,USP25,BAGE,LINC01425,CHODL-AS1,MIR3156-3,MIR8069-1,ANKRD30BP2,ANKRD20A11P,MIR155HG,TMPRSS15,RBM11,BAGE3	3	gain
1278	CGTE_17	21	27542717	28216305	21q21.3	ADAMTS1,APP,CYYR1	3	gain
1279	CGTE_17	21	31233886	32410703	21q22.11-q21.3	LINC00307,KRTAP6-1,KRTAP15-1,KRTAP21-1,KRTAP25-1,KRTAP19-6,KRTAP8-1,KRTAP11-1,KRTAP21-3,KRTAP24-1,KRTAP19-2,KRTAP13-2,KRTAP19-7,KRTAP19-1,KRTAP13-3,KRTAP26-1,KRTAP19-4,KRTAP22-2,KRTAP19-3,KRTAP13-1,KRTAP19-5,KRTAP6-3,KRTAP6-2,KRTAP20-1,KRTAP27-1,KRTAP20-4,KRTAP21-2,CLDN8,KRTAP23-1,KRTAP13-4,CLDN17,KRTAP22-1,KRTAP20-2,KRTAP7-1,GRIK1,MIR4327,KRTAP19-8,KRTAP20-3	3	gain
1280	CGTE_17	21	33764906	33785380	21q22.11	URB1-AS1,EVA1C,URB1	6	gain
1281	CGTE_17	21	34399847	34443066	21q22.11	OLIG1,LINC00945,OLIG2	19	gain
1282	CGTE_17	21	41684017	42647485	21q22.2-q22.3	DSCAM,PLAC4,BACE2,DSCAM-IT1,MIR3197,DSCAM-AS1,LINC00323	3	gain

1283	CGTE_17	21	42647488	42689035	21q22.3	FAM3B,BACE2	6	gain
1284	CGTE_17	21	45953454	46101952	21q22.3	KRTAP10-6,KRTAP10-9,KRTAP10-1,KRTAP12-4,KRTAP12-3,KRTAP10-7,KRTAP12-2,KRTAP10-8,TSPEAR,KRTAP10-10,KRTAP10-4,KRTAP12-1,KRTAP10-5,KRTAP10-2,KRTAP10-3,KRTAP10-11	3	gain
1285	CGTE_17	22	20130396	20136478	22q11.21	CCDC188,ZDHHC8	0	loss
1286	CGTE_17	22	21975728	21984303	22q11.21	YDJC,UBE2L3	6	gain
1287	CGTE_17	X	11316735	11776515	Xp22.2	AMELX,MSL3,ARHGAP6	4	gain
1288	CGTE_17	X	152686435	152710636	Xq28	ZFP92,TREX2	6	gain
1289	CGTE_18	1	6741013	101487301	1p33-p32.3	FOXD2,MYCBP,TXLNA,ROR1-AS1,LOC729970,MYCL,DAB1,ERICH3,MIR6729,LACTBL1,CASZ1,C1orf158,LOC101927876,ZBTB40,PIGK,WNT4,TMED5,EIF2B3,MST1L,SNORD103A,FOXE3,SGIP1,DPH5,C1QC,MIR7156,SLC35D1,MIR378F,SNX7,SNORD103B,SYDE2,CCDC18,EMC1,PITHD1,ZFP69B,GJA9-MYCBP,ZBTB8B,SMPDL3B,MIIP,SYTL1,LOC653160,CTRC,ASB17,DDI2,PUM1,LOC646471,LINC01364,EDN2,A3GALT2,FBXO2,SESN2,LOC101928241,CYR61,CYP4A11,MIR4695,HSPB7,LINC01144,LINC00466,LOC339539,HS2ST1,TEX38,WASF2,RRAGC,TTC4,B4GALT2,ZRANB2-AS1,ZYG11A,AUNIP,AGTRAP,HEYL,TNFRSF1B,DIRAS3,FOXD2-AS1,HFM1,UQCRH,ELOVL1,PRAMEF12,TNFRSF8,P3H1,APITD1,CYP4Z2P,BCL10,FAM183A,SLC35A3,MIR2682,CLCA4,STX12,ECE1,TFAP2E,NPPB,JUN,LOC105378683,LEPR,SLC44A3,HIVEP3,ACADM,TMEM61,MKNK1-AS1,CLDN19,ADGRL4,PTGER3,GJB4,NFIA-AS2,EIF3I,PPT1,PRKAA2,MIR6084,LOC102724571,BEND5,SNORD46,KAZN,PRAMEF20,NCMAP,TGFBR3,EPHB2,DNAJC11,LRRC42,MIR761,EXOSC10,ERI3,TRNAU1AP,UTP11L,SERBP1,OSBPL9,HOOK1,RBP7,TMEM201,GLMN,ZMYND12,SETSIP,C8A,PADI3,LRRC8C,ITGB3BP,SNORD21,PSMB2,MARCKSL1,DHDDS,ERI3-IT1,LOC101927139,ORC1,PADI6,SNIP1,SH2D5,MGC34796,EPS15,SNORD128,SSBP3-AS1,MIR101-1,MCOLN2,DEPDC1-AS1,RBMXL1,MATN1,RNU6-2,RNF186,LHX8,GPR88,FLJ27354,ASAP3,PLA2G2D,MIR6735,FHL3,GBP2,SPEN,FOXJ3,WDR63,PRAMEF18,RHD,GBP6,RPE65,SH3D21,TMEM125,CYP2J2,UOX,UBXN10,CFAP57,NUDC,AKR7A2,SNORD45B,PARK7,UQCRHL,PPIH,SLC25A33,MIR4422,DIO1,MIR6733,MIR6079,RPS8,MECR,SMAP2,RAB42,SNORA59A,LOC100506801,XKR8,KLHDC7A,PTBP2,LINC01359,SNORA61,ZCCHC17,ELAVL4,ROR1,SNORD55,NFIA-AS1,YTHDF2,PGM1,LOC100506022,LOC101928118,MIR4684,PLEKHM2,SLC2A5,LOC101926964,ZNF593,ST3GAL3,CLSPN,PRAMEF2,PLPPR4,SF3A3,MIR3917,MCOLN3,MED8,SLC30A7,GBP1,C1orf195,GALE,LOC100129046,BMP8B,PTPRU,ARHGEF19,MTHFR,TMCO4,C1orf109,CTH,CDCP2,SRRM1,PALMD,PRAMEF17,PRAMEF26,LOC101928163,ARTN,GNG12,MTF1,EPHA8,PAQR7,PODN,TIE1,GPN2,LINC01361,LOC101927244,MAD2L2,MIR6068,OXCT2,LOC101927560,HNRNPR,SRSF10,MIR4419A,SYNC,JAK1,AIM1L,SNORA59B,ADPRHL2,CELA2A,LINC01389,RAB3B,GBP3,HSPB11,MATN1-AS1,ARHGAP29,CA6,LRRC40,LINC01355,GEMIN8P4,RSRP1,DBT,SZT2,SLC5A9,FAM151A,GNL2,BRDT,SSBP3,AADACL3,MIR4418,EPHX4,PINK1-AS,ZNF436-AS1,ZNF684,TMEM51-AS1,PRDM2,MIR5581,MFAP2,PEX14,GJA4,RNF19B,IL12RB2,NBL1,RAVER2,DCDC2B,NEGR1-IT1,HPCAL4,MDS2,PLA2G2E,NEXN-AS1,PRAMEF4,PRAMEF15,MMACHC,ZNF683,MIR6730,DPH2,PRAMEF22,RLF,TARDBP,ZNF326,MIR30C1,RSPO1,GLIS1,FAM131C,ARHGEF10L,FBLIM1,ACTG1P20,TSPAN1,HTR6,BTBD19,LOC101929721,PARS2,C1orf64,ZNF436,PRAMEF5,TRMT13,LRRC38,ZDHHC18,PGD,AADACL4,MGC27382,MAP7D1,HHLA3,CTNNBIP1,CTBS,ECHDC2,DDOST,DPYD-AS1,CORT,LOC101927412,DNTTIP2,KTI12,CCDC30,MIR5095,PLK3,HPDL,C1orf123,IPP,SNHG12,MFSD2A,CCDC24,HPCA,LINC00853,ERICH3-AS1,DOCK7,FOXO6,SVBP,GNG12-AS1,PDIK1L,HNRNPCL1,IPO13,CMPK1,PTCH2,TAL1,KIAA2013,TTC39A,PDE4B,FCN3,FOXD3-AS1,GPATCH3,NCDN,PKN2-AS1,C1orf146,MIR548D1,LOC646626,RTCA,NPPA-AS1,CROCC,DLEU2L,HNRNPCL3,C1QA,GUCA2A,GJB5,MIR1976,GCLM,MYSM1,MAGOH,HMGN2,LINC00339,BARHL2,TYW3,FAAH,TMEM56,MTF2,TMEM200B,UTS2,TCEB3,LINC0	3	gain

1290	CGTE_18	1	101490748	108023667	1p21.1-p13.3	ACTG1P4,LOC101928370,LOC100129138,AMY1B,LOC101928436,AMY2B,OLFM3,PRMT6,NTNG1,AMY1C,AMY2A,LOC101928476,DPH5,LOC102606465,S1PR1,COL11A1,AMY1A,LINC01307,DNAJA1P5,RNPC3	4	gain
1291	CGTE_18	1	108115797	152006332	1q21.1-p11.1	NHLH2,KCND3-IT1,TSHB,FAM72C,TXNIP,PLEKHO1,C1orf162,GSTM5,AHCYL1,KIAA1324,PDZK1P1,NUDT17,HORMAD1,VPS72,MIR5087,GSTM4,ADAMTSL4-AS1,GDAP2,HIPK1-AS1,KCNC4,PSMB4,HAO2-IT1,THEM5,WARS2,KCNA2,NBPF8,LOC440602,RNVU1-8,LRIG2,C1orf194,HIST2H4B,CGN,GPR61,MIR6878,LAMTOR5-AS1,HIST2H2BF,PI4KB,CYB561D1,SPAG17,SPATA42,THEM4,NGF,OVGP1,HIST2H3A,RPRD2,NBPF11,FAM212B,RNVU1-19,TTF2,KCND3,FNDC7,FALEC,RSBN1,PROK1,TSPAN2,BOLA1,OAZ3,PTPN22,DCLRE1B,C1orf137,VAV3,KCNC4-AS1,CLCC1,MIR4256,GOLPH3L,CIART,CELF3,PDE4DIP,PEX11B,NBPF6,SYCP1,RBM8A,GNAI3,NBPF10,TDRKH,HIPK1,FAM72D,ARNT,TMEM167B,CELSR2,ST7L,TRIM45,SF3B4,TUFT1,NBPF25P,EPS8L3,ECM1,LINC01160,CTSK,LOC440600,GPSM2,SEMA6C,MOV10,MIR320B1,HIST2H2AC,LINC00624,NBPF14,WNT2B,HIST2H3C,CTTNBP2NL,HENMT1,SLC16A1-AS1,PRPF3,CYMP,LOC101928977,LOC102723769,POLR3C,CSDE1,HIST2H2BC,LINGO4,STRIP1,STXBP3,RORC,CHIAP2,CD2,WDR3,CDC42SE1,DRD5P2,APH1A,VTCN1,LOC100132111,KCNA3,NBPF4,GSTM2,HSD3BP4,PPM1J,MAB21L3,MIR6077,SLC16A4,FAM63A,ATP5F1,ANP32E,MIR942,ATP1A1-AS1,FAM46C,WARS2-IT1,TAF13,GNRHR2,C1orf56,ATP1A1,HAO2,HIST2H2AA4,MCL1,PRKAB2,WDR47,TMIGD3,MIR4257,RIIAD1,SIKE1,LOC643355,NOTCH2NL,SCNM1,BCL2L15,GSTM1,LINC01356,LINC01138,LOC100996251,SELENBP1,CERS2,LINC00623,SCARNA2,MTMR11,PIP5K1A,SLC16A1,BCL9,AKR7A2P1,LOC728989,GABPB2,CHIA,NBPF20,AP4B1,NOTCH2,FCGR1A,IGSF3,POGZ,S100A11,BNIPL,DENND2C,NBPF9,NBPF15,LIX1L,UBL4B,FAM102B,TRIM33,OLFML3,CEPT1,PRPF38B,SLC6A17,HIST2H2AA3,CD160,HIST2H2BA,LOC101927468,DRAM2,ANXA9,PSRC1,NBPF7,C1orf54,ACP6,MLLT11,HFE2,TBX15,MAGI3,RNVU1-20,ANKRD35,ZNF687,NBPF12,SETDB1,PGCP1,PDZK1,CASQ2,MRPS21,AKNAD1,KCND3-AS1,GPR89B,C2CD4D,DDX20,VPS45,MAN1A2,HSD3B2,REG4,SYPL2,FCGR1C,HMGCS2,RNF115,ATXN7L2,NRAS,KCNA10,AMIGO1,NBPF13P,ADAMTSL4,TARS2,POLR3GL,FAM72B,MIR197,SYT6,VANGL1,PPIAL4A,LOC101060524,ALX3,SLC25A24,PFN1P2,LOC101929099,LOC101928979,SLC22A15,PIFO,SRGAP2D,CHI3L2,MYBPHL,PRUNE,FAM212B-AS1,GJA8,LINC01525,ENSA,HIST2H2BE,PHTF1,LOC643441,ADORA3,RAP1A,FAM19A3,SORT1,AP4B1-AS1,LOC101928995,WDR77,PHGDH,FCGR1B,GPR89A,S100A10,SRGAP2B,ITGA10,MIR554,HSD3B1,TNFAIP8L2-SCNM1,SEC22B,SRGAP2-AS1,FMO5,TNFAIP8L2,LOC101928718,LYSMD1,LINC00869,EMBP1,OTUD7B,CSF1,PSMA5,LOC645166,SARS,CAPZA1,LOC103091866,ADAM30,CTSS,MRPL9,CD58,LOC101929023,AMPD1,HIST2H4A,LINC01397,PPIAL4F,HIST2H2AB,FAM231D,PSMD4,MIR6736,LAMTOR5,PIAS3,PPIAL4D,PDIA3P1,LOC101929147,NBPF18P,LOC388692,BCAS2,ANKRD20A12P,AMPD2,GBAT2,LOC100132057,PPIAL4C,CHD1L,HIST2H3D,RFX5,CA14,RHOC,PPIAL4E,ANKRD34A,PPIAL4G,GNAT2,MIR7852,GSTM3,CD101,PTGFRN,DENND2D,SV2A,GJA5,VAV3-AS1,ZNF697,SNX27,CD53,LOC100996263,RBM15,TMOD4,LRIF1,LINC00622	3	gain
1292	CGTE_18	1	152009228	152382006	1q21.3	RPTN,S100A11,LOC100131107,FLG,HRNR,FLG-AS1,CRNN,FLG2,TCHHL1,TCHH	4	gain
1293	CGTE_18	1	152382018	153234472	1q21.3	LCE6A,LOR,C1orf68,LCE3A,LELP1,SPRR2G,SPRR1A,LCE3C,LCE1D,LCE4A,SPRR4,LCE3E,LCE5A,SPRR2F,SPRR2A,KPRP,IVL,CRNN,LCE1A,SMCP,LCE1E,CRCT1,LCE3D,LCE2D,LCE3B,LCE1B,SPRR3,LCE1F,LOC101928009,SPRR2C,LCE2B,SPRR2B,PRR9,SPRR2D,SPRR2E,SPRR1B,LCE2C,LCE2A,LCE1C	6	gain

1294	CGTE_18	1	157490037	158986620	1q23.1	OR6N1,CD1E,OR6P1,CD1C,CD5L,FCRL1,OR10K1,FCRL5,LOC646268,OR6K3,OR10R2,IFI16,CD1B,PYHIN1,CD1A,OR6Y1,OR10K2,FCRL3,OR6K2,MNDA,OR6K6,OR10X1,OR10Z1,SPTA1,KIRREL,FCRL2,FCRL4,CD1D,OR6N2,OR10T2	4	gain
1295	CGTE_18	1	158987988	200183405	1q24.1-q31.1	TEX35,PVRL4,KCNT2,NR1I3,PRRX1,DNM3,HMCN1,LRRC52,F13B,SNORD79,VAMP4,DUSP23,CD247,MROH9,RGS13,BLZF1,GLUL,SUMO1P3,PIGM,TOR1AIP2,RFWD2,CD48,LOC440700,LOC101928565,LOC101928404,PTGS2,SOAT1,LOC730102,TADA1,IVNS1ABP,CFHR4,MR1,LOC100147773,APOA2,SH2D1B,SLAMF1,ATP6V1G3,RPL31P11,RALGPS2,VSIG8,SLC19A2,SNORD76,MIR4654,NHLH1,LOC102724601,RGS18,RNASEL,MRPS14,DPT,SNORD81,XCL1,SNORD75,CDC73,PTPRC,IGSF8,QSOX1,DNM3-IT1,SMG7-AS1,ADCY10,FAM20B,OR10J3,LOC400794,SHCBP1L,PACERR,OCLM,C1orf53,LHX4,ILDR2,TSTD1,UFC1,USF1,SEC16B,PBX1,TNN,RGS21,DUSP12,LINC01221,DDR2,SMG7,ATF6,CADM3,EDEM3,ZNF648,TPR,CADM3-AS1,DENND1B,CD84,GLRX2,MIR3658,NME7,C1orf105,LOC102724919,LINC01031,LOC101928973,LINC01344,CACNA1E,AIM2,GM140,MAEL,F5,DARS2,UHMK1,LOC101928372,FAM163A,ATP1A2,LINC00970,ABL2,MIR181B1,SNORA103,OVAAL,TNFSF4,LINC01363,NEK7,RXRG,ATP1A4,TOMM40L,MIR214,DUSP27,CACYBP,MIR488,NUF2,MIR4424,LOC729867,LHX4-AS1,LINC01037,MIR548F1,LOC101928673,NPHS2,ACBD6,RCSD1,LINC00272,LINC01350,LOC100505918,C1orf111,MIR556,OR10J1,XCL2,RNF2,RGS4,C1orf204,IFI16,FMO3,MIR4735,KCNJ9,C1orf220,MIR199A2,FMO6P,C1orf226,MIR1295A,RGL1,SNORD78,LY9,FAM129A,KCNJ10,CCDC181,CFHR1,PCP4L1,POGK,HSPA7,PFDN2,SLC9C2,UCK2,SLAMF9,CFHR3,PLA2G4A,METTL18,NCF2,FLJ23867,LINC00626,GS1-279B7.1,CFHR5,LAMC2,SLAMF7,FCGR2A,CFAP45,MIR1278,LOC101928696,LOC730159,FCGR2C,C1orf21,MPZ,CD244,TAGLN2,GAS5-AS1,SFT2D2,GPA33,C1orf27,MIR4426,TDRD5,FCRLB,FCGR2B,TOP1P1,LINC01351,MPZL1,NMNAT2,ANKRD45,GPR52,TRMT1L,B4GALT3,CFHR2,USP21,CREG1,RGS5,POU2F1,LOC101928751,CEP350,MIR3121,RASAL2,DNM3OS,FCGR3A,CRB1,SNORD80,SELL,SUCO,RGSL1,LINC01032,F11R,C1orf112,ATP1B1,TMCO1,OLFML2B,AXDND1,LINC01142,TSEN15,LOC100506023,PAPPA2,ITLN2,TBX19,RGS1,MIR181A1,KIAA1614,MIR181A1HG,RABGAP1L,PEX19,LOC100505795,STX6,GAS5,LAMC1,HSD17B7,KIAA0040,PRG4,FAM78B,ZBTB37,VANGL2,ARHGAP30,FMO1,GORAB,LHX9,SDHC,SNORD47,KIFAP3,FCRL6,FMO9P,PDC,CRP,DCAF6,TEDDM1,ALDH9A1,LINC01036,B3GALT2,METTL11B,SCYL3,COLGALT2,APCS,ITLN1,PPOX,MIR1255B2,NR5A2,RASAL2-AS1,RC3H1,TOR1AIP1,BRINP3,TNFSF18,LOC101928778,MIR5187,LOC284648,NPL,HSPA6,SNORD44,ACKR1,TNR,SERPINC1,PRDX6,COPA,APOBEC4,CCDC190,MIR4259,XPR1,MIR557,CASQ1,ARPC5,ASTN1,SCARNA3,SLAMF8,BRINP2,ASPM,RGS16,NDUFS2,DHX9,CFH,METTL13,LOC100422212,FASLG,SELP,NIT1,MIR921,FCRLA,ANKRD36BP1,TOR3A,CFAP126,OR10J5,SWT1,DCAF8,TIPRL,MIR3120,LOC102724661,FCER1G,SLAMF6,RGS2,SELE,CENPL,DEDD,NOS1AP,FCGR3B,MPC2,MIR3119-1,FCER1A,PEA15,MGST3,LOC440704,NCSTN,UAP1,ANGPTL1,MYOC,SNORD74,RGS8,MIR3119-2,ADAMTS4,TROVE2,KLHL20,ZBTB41,IER5,LMX1A,GPR161,KLHDC9,MIR1295B,UCHL5,LOC101928650,PRRC2C,SNORD77,IGSF9,PIGC,LINC01133,FMO2,LINC01222,FMO4	3	gain

1296	CGTE_18	1	205042603	214826873	1q32.1-q41	MIR205HG,TATDN3,TMEM206,LEMD1-AS1,SNORA16B,IL10,KCNH1,SMYD2,KLHDC8A,VASH2,HHAT,NUCKS1,CR1L,C4BPB,CENPF,NUAK2,MIR29C,ATF3,MIR6769B,LOC284581,MFSD4,RASSF5,PPP2R5A,MIR205,CR1,ELK4,PM20D1,FAM71A,RBBP5,FCAMR,IKBKE,NEK2,PTPN14,PROX1-AS1,SYT14,SPATA45,PFKFB2,YOD1,INTS7,LOC101929565,FAM72A,PLXNA2,LAMB3,BATF3,LOC148696,SRGAP2D,BLACAT1,ANGEL2,SERTAD4-AS1,TMEM81,MIR3122,FLVCR1-AS1,CTSE,TMCC2,SLC30A1,SERTAD4,SLC45A3,G0S2,CD55,CNTN2,RAB29,RD3,LEMD1,CR2,DSTYK,DTL,IL20,SRGAP2,LINC00538,CD46,TRAF5,LPGAT1,MIR29B2,FCMR,C4BPA,FLVCR1,MIR4260,C1orf186,AVPR1B,MAPKAPK2,HSD11B1,RCOR3,PROX1,IL19,SLC26A9,RPS6KC1,MIR135B,DYRK3,CAMK1G,NENF,LOC101929541,IRF6,LOC105748977,CDK18,FAM72C,C1orf74,LOC284578,SLC41A1,CD34,NSL1,C1orf116,TRAF3IP3,PIGR,IL24,SRGAP2C,DIEXF,LINC00467,EIF2D	3	gain
1297	CGTE_18	1	214828613	218520449	1q41	LOC101929631,RRP15,SPATA17,SPATA17-AS1,ESRRG,KCTD3,TGFB2-AS1,USH2A,LOC102723833,LINC00210,KCNK2,CENPF,GPATCH2,TGFB2	4	gain
1298	CGTE_18	1	218520470	228430933	1q42.13-q42.11	CDC42BPA,WDR26,MARC1,MARC2,AIDA,TP53BP2,RAB3GAP2,CNIH4,ZNF678,AURKAPS1,PARP1,FAM177B,ITPKB-IT1,LIN9,GTF2IP20,CNIH3,IBA57,HLX,IARS2,MIR320B2,MIR664A,MIR194-1,MIR215,RNU5F-1,SNORA36B,C1orf145,TLR5,JMJD4,BROX,SLC30A10,C1orf140,GJC2,LOC101927143,GUK1,LEFTY1,PSEN2,BPNT1,C1orf35,PYCR2,WNT3A,FBXO28,C1orf95,TAF1A-AS1,DEGS1,ITPKB,MIR6741,CAPN2,LYPLAL1-AS1,LOC101929771,MIR4742,C1orf115,HLX-AS1,SNAP47,SUSD4,SRP9,CAPN8,DNAH14,ENAH,LEFTY2,LOC101927164,LYPLAL1,ACBD3,ADCK3,WNT9A,MRPL55,SDE2,H3F3AP4,MIA3,LOC100130093,MIXL1,HHIPL2,LBR,TMEM63A,TGFB2,CCDC185,IBA57-AS1,ZNF847P,TGFB2-OT1,EPHX1,EPRS,MIR3620,ARF1,TAF1A,H3F3A,DUSP10,DISP1,MIR548F3,PRSS38,MARK1,NVL,LINC01352,OBSCN,MIR5008	3	gain
1299	CGTE_18	1	228431000	228452127	1q42.13	OBSCN	5	gain

1300	CGTE_18	1	228601501	249212637	1q42.3-q43	GNG4,OPN3,FAM89A,TSNAX-DISC1,MIR4454,CNST,SLC35F3,GNPAT,OR14C36,LOC255654,LYST,OR14A16,MIR1537,NLRP3,ZNF695,OR2G3,LOC101927683,ARV1,SMYD3,OR2M3,DISC2,SCCPDH,ACTN2,PCNXL2,TBCE,TAF5L,MIR5096,OR2T4,TARBP1,C1orf131,KMO,CEP170,OR2M1P,OR2T1,OR2C3,ACTA1,NID1,MIR4753,MIR4677,EXOC8,GCSAML,MIR1182,SPRTN,OR2M2,COA6,MIR4666A,ABCB10,WDR64,RPS7P5,DISC1-IT1,CHRM3-AS1,RNF187,OR2M7,NUP133,TOMM20,ZNF669,OR2L8,LINC01139,MIR3916,CCSAP,HEATR1,OR13G1,ARID4B,AKT3,LOC101927787,LOC101927765,LINC01132,MIR3124,GREM2,ZNF124,HNRNPU,OR2W5,LOC149373,OR2T33,LOC101927851,MAP10,COG2,BTNL10,OR2T34,TTC13,OR1C1,LGALS8,URB2,AHCTF1,SPHAR,DUSP5P1,RBM34,SNORA100,GALNT2,ZNF670,VN1R5,SH3BP5L,KIF26B,DISC1,C1orf100,LOC100130331,MIR4428,ZNF670-ZNF695,TSNAX,OR2T8,OR2L5,OR14I1,MAP1LC3C,LYPD8,TFB2M,PGBD2,LINC01341,OR2L1P,ZNF496,IRF2BP2,ZBTB18,OR2M5,PLD5,OR2L13,LINC01354,FH,LOC101927604,B3GALNT2,ZP4,OR2W3,MIR1273E,OR2L2,LINC01347,RYR2,RGS7,COX20,PGBD5,OR2T35,OR2G2,OR2L3,EXO1,KIAA1804,SIPA1L2,CHRM3-AS2,C1orf101,OR2T10,LOC101928226,OR2T6,OR2T5,OR2T11,OR2B11,TRIM58,OR6F1,GGPS1,EGLN1,MIR4427,MIR7641-2,CHML,MIR3123,HIST3H2BB,MTR,LOC339529,SNORA14B,ERO1B,EFCAB2,AGT,OR11L1,RHOU,OR2T29,MT1HL1,OR2G6,NTPCR,OR2T27,CHRM3,SNRPD2P2,GPR137B,OR2T12,FMN2,LOC101928068,TRIM67,DESI2,LGALS8-AS1,EDARADD,BECN2,MIR4671,LINC00184,LINC00582,C1orf229,HIST3H2A,ZNF692,GCSAML-AS1,C1orf198,OR2AK2,TRIM17,ADSS,SDCCAG8,OR2T3,CAPN9,HIST3H3,RAB4A,LOC101927478,KCNK1,ZNF672,OR2M4,OR2T2	3	gain
1301	CGTE_18	2	28863705	28975061	2p23.2	PPP1CB,PLB1	7	gain
1302	CGTE_18	2	49295268	52929801	2p16.3-p16.2	MIR8485,NRXN1,MIR4431,FSHR	4	gain
1303	CGTE_18	2	74906586	84752864	2p12-p11.2	GCFC2,LOC101927967,LOC1720,LOC101927884,DNAH6,REG1CP,LOC100507201,HK2,LRRTM1,SEMA4F,POLE4,REG1B,MIR8080,SNAR-H,LINC01291,TACR1,LOC101927926,MRPL19,MIR4264,LOC101927987,REG3A,LOC101927907,FUNDC2P2,SUCLG1,EVA1A,LOC101927948,LRRTM4,REG3G,CTNNA2,MIR5000,REG1A	3	gain
1304	CGTE_18	2	137639709	149402771	2q22.3-q23.1	ZEB2,LRP1B,LOC101928273,SPOPL,KYNU,MBD5,PABPC1P2,ORC4,HNMT,GTDC1,ZEB2-AS1,EPC2,LOC101928386,ARHGAP15,TEX41,YY1P2,ACVR2A,LINC01412,NXPH2,THSD7B,MIR7157	3	gain
1305	CGTE_18	2	163144666	164468397	2q24.2-q24.3	GCA,FIGN,IFIH1,LOC101929570,KCNH7	4	gain
1306	CGTE_18	2	164591380	170336166	2q31.1-q24.3	SNORA70F,GALNT3,SPC25,COBLL1,GRB14,G6PC2,STK39,LRP2,XIRP2-AS1,CERS6-AS1,TTC21B-AS1,NOSTRIN,LOC101929680,LOC100506124,DHRS9,LOC105616981,SCN9A,CSRNP3,SCN2A,SCN3A,SCN7A,LOC101929633,BBS5,XIRP2,TTC21B,MIR4774,B3GALT1,CERS6,ABCB11,SCN1A,LOC102724058,FIGN,SLC38A11	3	gain
1307	CGTE_18	2	179465497	190306394	2q31.3-q31.2	MIR1258,FSIP2,SCHLAP1,MIR548AE1,MIR3606,ZC3H15,WDR75,SSFA2,MIR548N,UBE2E3,ZSWIM2,CERKL,TTN,MIR1245B,MIR4437,PDE1A,LINC01473,PPP1R1C,LOC101927055,NUP35,ZNF385B,ZNF804A,MIR561,NCKAP1,FRZB,COL5A2,NEUROD1,MIR3129,LOC101927196,CCDC141,DIRC1,ITGAV,ITGA4,LOC101927156,FAM171B,TTN-AS1,MIR1245A,TFPI,CWC22,DUSP19,SESTD1,CALCRL,DNAJC10,COL3A1,LINC01090,GULP1	3	gain

1308	CGTE_18	2	209165620	216990802	2q34-q35	XRCC5,TMEM169,LOC102725079,MIR4776-2,LANCL1,ACADL,MIR548F2,VWC2L-IT1,LINC01614,PECR,UNC80,LOC100130451,ABCA12,LOC101928020,ERBB4,PIKFYVE,LINC00607,FN1,LANCL1-AS1,MIR4438,KANSL1L,MAP2,LOC101928103,PTH2R,MIR4776-1,CPS1,MYL1,BARD1,VWC2L,LOC102724849,ATIC,SPAG16,MREG,RPE,CPS1-IT1,IKZF2	3	gain
1309	CGTE_18	3	77147119	89528654	3p11.2-p12.2	LINC00971,EPHA3,MIR4795,ROBO2,CADM2,SNORA95,CADM2-AS2,LINC00506,CGGBP1,C3orf38,LOC101927374,LOC728290,HTR1F,ROBO1,CHMP2B,MIR5688,MIR3923,VGLL3,GBE1,ZNF654,POU1F1	3	gain
1310	CGTE_18	3	129305373	133292983	3q22.1	NUDT16,ATP2C1,FAM86HP,MRPL3,COL6A5,ALG1L2,DNAJC13,ASTE1,TMEM108-AS1,NUDT16P1,ACPP,SNORA58,NPHP3-ACAD11,CDV3,TRH,CPNE4,PLXND1,NEK11,UBA5,TMEM108,NPHP3,ACKR4,BFSP2,NPHP3-AS1,LOC339874,TMCC1,TMCC1-AS1,PIK3R4,MIR5704,COL6A6,ACAD11,COL6A4P2	3	gain
1311	CGTE_18	3	138724259	139063000	3q23	PISRT1,BPESC1,PRR23C,PRR23B,PRR23A,MRPS22	7	gain
1312	CGTE_18	3	139063430	180705962	3q26.31-q26.2	MIR7977,ARHGEF26-AS1,P2RY1,CPA3,MIR15B,ZBTB38,LOC100507537,MIR16-2,LINC00886,GPR149,TFDP2,CP,NAALADL2-AS2,TM4SF1-AS1,P2RY14,LOC100128164,MIR5186,LOC101928882,HLTF,PAQR9-AS1,MIR551B,TRPC1,RNF13,AGTR1,LOC100507291,AADACL2-AS1,RSRC1,RAP2B,KCNAB1-AS1,MED12L,SCARNA7,TNIK,LOC440982,WWTR1-AS1,TTC14,LINC01213,KCNMB2,RARRES1,NCEH1,TNFSF10,TM4SF1,LOC101243545,EIF5A2,LOC100507661,XRN1,PXYLP1,SLC7A14,LOC100505609,PCOLCE2,CLDN11,LOC101928739,NAALADL2,IGSF10,GOLIM4,AADACP1,NLGN1,SLC9A9-AS1,GHSR,AADAC,TMEM212-AS1,SMC4,MLF1,LINC00578,RBP1,RPL22L1,GRK7,PLSCR4,PRKCI,TRIM59,SLC2A2,EGFEM1P,SAMD7,KCNMB3,NAALADL2-AS1,SPSB4,COPB2,IQCJ,GK5,PLCH1,PAQR9,GMPS,COMMD2,ATP1B3,AADACL2,CLSTN2-AS1,SEC62,PLSCR5,PLSCR1,MME,MIR1263,CPB1,MIR569,SHOX2,C3orf79,LINC00881,ERICH6,FNDC3B,PLOD2,SLC33A1,OTOL1,ANKUB1,LINC01209,GPR87,SLITRK3,ARHGEF26,DHX36,MIR548AY,GNB4,MBNL1,TBL1XR1,LINC00501,ACTRT3,LEKR1,PTX3,LRRC34,IFT80,CLRN1,LINC01330,MRPL47,RASA2,MBNL1-AS1,PQLC2L,SIAH2,GPR160,MFSD1,IL12A,LINC00880,PHC3,SPTSSB,GYG1,MIR6828,HLTF-AS1,IQCJ-SCHIP1-AS1,TIPARP-AS1,TMEM212,SERPINI2,SSR3,LINC01208,ARL14,LOC100289361,LOC646903,KCNAB1-AS2,C3orf80,ECT2,LINC01327,SERP1,LINC01214,MIR4789,CCDC39,SLC9A9,LINC01192,PDCD10,WWTR1,SUCNR1,SCHIP1,EIF2A,NLGN1-AS1,PLS1,LOC100507389,PA2G4P4,CHST2,LXN,HPS3,SPATA16,TM4SF4,TM4SF18,ZMAT3,GPR171,SELT,LINC01322,IQCJ-SCHIP1,VEPH1,ACTL6A,SI,WDR49,ZNF639,MIR3919,TSC22D2,LOC100996447,TIPARP,PLSCR2,KPNA4,LINC01324,LOC101928105,USP13,LINC01014,P2RY13,MECOM,SLC25A36,SKIL,ERICH6-AS1,LINC01487,CCNL1,PEX5L-AS2,ZIC4,ZBBX,CLSTN2,MYNN,LRRC31,KCNAB1,DNAJC19,NDUFB5,U2SURP,TMEM14EP,TRIM42,FXR1,RNF7,LINC01100,C3orf33,PFN2,PIK3CA,KCNMB2-AS1,MRPS22,IL12A-AS1,CLRN1-AS1,MIR548H2,RBP2,P2RY12,LRRIQ4,NMNAT3,PEX5L,BCHE,NMD3,PPM1L,SERPINI1,B3GALNT1,KCCAT211,C3orf58,MFN1,PLD1,NAALADL2-AS3,ZIC1,GFM1,TERC,ATR	3	gain
1313	CGTE_18	3	180707157	182166175	3q26.33	SOX2,SOX2-OT,LOC102724604,FLJ46066,RNU6-2,DNAJC19,LINC01206	6	gain

1314	CGTE_18	3	186978491	192980997	3q28-q27.3	SNAR-I,TP63,TPRG1-AS1,MB21D2,LOC100131635,PYDC2,TPRG1-AS2,MIR28,P3H2,LPP,BCL6,CCDC50,HRASLS,MGC2889,LPP-AS2,SST,CLDN16,FLJ42393,LPP-AS1,GMNC,RTP4,P3H2-AS1,UTS2B,IL1RAP,LINCR-0002,CLDN1,RTP2,OSTN,TPRG1,FGF12,OSTN-AS1,MASP1,MIR944,FGF12-AS1,TMEM207	3	gain
1315	CGTE_18	4	53238	10600464	4p16.3-p16.2	PPP2R2C,UVSSA,MRFAP1L1,MSANTD1,TACC3,FAM86EP,HAUS3,ABCA11P,IDUA,MIR378D1,LOC93622,USP17L21,ACOX3,MYL5,FLJ36777,ADD1,LOC100129931,NELFA,LOC100129917,MIR4274,AFAP1,FGFRL1,EVC,CPZ,PCGF3,NOP14-AS1,HGFAC,USP17L25,SCARNA22,WHSC1,USP17L20,MSX1,PDE6B,S100P,CYTL1,TADA2B,MAN2B2,MAEA,USP17L9P,USP17L15,TRMT44,SH3BP2,LOC650293,USP17L24,ZFYVE28,MIR4798,STK32B,LYAR,SORCS2,USP17L10,RNF4,ZNF595,MIR4800,NSG1,USP17L11,NOP14,USP17L22,GRK4,CTBP1-AS2,DRD5,ADRA2C,MFSD10,GAK,SLBP,CRMP1,MRFAP1,SLC26A1,TMED11P,AFAP1-AS1,LOC389199,LOC100133461,LOC100130872,ZNF721,USP17L18,ZNF876P,PSAPL1,STX18,ZNF718,USP17L26,LRPAP1,C4orf48,STX18-IT1,LOC101928279,LINC00955,FAM53A,BLOC1S4,USP17L27,TMEM129,SH3TC1,CPLX1,CTBP1-AS,USP17L30,DOK7,WDR1,DEFB131,GRPEL1,FGFR3,LETM1,USP17L28,LOC285484,CLNK,HTRA3,USP17L17,ZNF518B,ZNF732,USP17L13,USP17L12,USP17L6P,CFAP99,SLC2A9,HMX1,DGKQ,USP17L5,MIR95,KIAA0232,OTOP1,GPR78,CRIPAK,JAKMIP1,ABLIM2,HTT,MIR548I2,FAM193A,NKX1-1,TBC1D14,USP17L19,LOC101928306,STX18-AS1,RGS12,CTBP1,TMEM175,NAT8L,PIGG,SPON2,ZNF141,USP17L29,EVC2,RNF212,MXD4,MIR943,ATP5I,CCDC96,ZBTB49,MIR3138,POLN,MIR571,WFS1,TNIP2,MFSD7,LINC01396,TMEM128,LINC01587,HTT-AS	1	loss
1316	CGTE_18	4	62383011	76439480	4q21.1-q13.1	CXCL5,MTHFD2L,EPHA5-AS1,LOC728040,ODAM,UGT2A3,MUC7,AFP,PF4V1,AMTN,PROL1,DCK,CSN2,UGT2A1,UBA6,AREG,FDCSP,LOC100507388,COX18,TMPRSS11F,CABS1,UGT2A2,MOB1B,EPHA5,ADGRL3,CXCL3,CSN1S1,TMPRSS11BNL,ENAM,RUFY3,PPBPP2,AMBN,CXCL6,CSN1S2BP,ANKRD17,RCHY1,UGT2B17,UBA6-AS1,JCHAIN,TECRL,UTP3,TMPRSS11B,STAP1,LOC401134,NPFFR2,CSN3,CXCL1,SULT1B1,SYT14P1,CXCL2,LOC550113,TMPRSS11E,ADAMTS3,CSN1S2AP,TMPRSS11D,UGT2B15,SLC4A4,ADGRL3-AS1,STATH,UGT2B10,GC,LOC441025,UGT2B4,EREG,ALB,CXCL8,GNRHR,PPBP,PRR27,UGT2B28,TMPRSS11A,LOC101927237,YTHDC1,GRSF1,PF4,UGT2B7,SMR3A,PARM1,SULT1E1,FTLP10,MIR1269A,HTN1,HTN3,EPGN,AFM,RASSF6,BTC,TMPRSS11GP,CENPC,SMR3B,UGT2B11	3	gain

1317	CGTE_18	4	77357165	184366266	4q35.1-q21.21	UGT8,RPS3A,ZGRF1,WWC2,BBS7,LOC101929577,NUDT6,ETFDH,GATB,USP53,LOC100506746,ADH1C,CXXC4,PCDH10,LOC101929210,TRAM1L1,ADH1B,ADH6,DKFZP434I0714,SPOCK3,TMA16,MIR1255A,LINC01179,GAR1,LOC101927636,GLRA3,RNF150,C4orf36,THAP9-AS1,LOC340017,LOC101929529,SLC10A7,C4orf45,TET2,CEP44,PPP3CA,ANAPC10,HHIP,HHIP-AS1,SMARCA5,IL2,MEPE,WDR17,SNCA,WDFY3,NR3C2,MIR302D,UNC5C,FAM160A1,TIGD2,MFSD8,WWC2-AS2,PYURF,CDKN2AIP,LINC01216,LARP1B,HERC5,FGA,DNAJB14,GPAT3,LOC101928590,MIR4452,GALNT7,MIR2054,AGA,TET2-AS1,LINC01061,LOC101927282,MIR575,LOC100506122,LOC101928314,GUCY1A3,LIN54,LOC101929595,FGG,DCHS2,LOC101929468,RAB33B,IL15,SNCA-AS1,TBC1D9,NKX6-1,DAPP1,ARHGEF38-IT1,KLHL8,CCSER1,MIR4450,PRDM5,MAB21L2,MANBA,TMEM154,PIGY,CEP170P1,ARHGAP24,MIR8082,LINC01098,MRPL1,OTUD4,POU4F2,CCNG2,ELOVL6,NDNF,HSD17B13,ADH7,HNRNPD,LINC01095,LOC101927359,MGAT4D,RRH,SMARCA5-AS1,SPARCL1,NAA15,TBCK,NOCT,NPNT,QRFPR,NFKB1,BMPR1B-AS1,IL21,MSMO1,DDX60,SH3D19,LINC01378,ABCE1,NEIL3,INTS12,DCLK2,JADE1,RXFP1,PABPC4L,MFAP3L,MIR4799,RPL34,PLRG1,C4orf46,CLDN24,MIR578,SFRP2,GYPA,PRSS48,TDO2,C4orf3,LOC101929194,MIR4451,FABP2,EIF4E,PAQR3,SHROOM3,HELQ,LOC100507487,ELMOD2,HAND2,LOC101928942,ADAD1,H2AFZ,BMP3,MYOZ2,ENOPH1,APELA,FHDC1,RPL34-AS1,LOC101928131,CPE,BMPR1B,AP1AR,ADAM29,KLHL2,SLC9B2,MAP9,ANK2,GUCY1B3,TRIM2,FRAS1,RAPGEF2,MMAA,ASB5,PDGFC,BANK1,TMEM144,CCNA2,INTU,SNORD73A,MIR3688-2,CLDN22,PDHA2,LOC101927849,CASP6,MIR5684,SEC24D,C4orf17,SLC7A11,FBXW7,RNF175,PRSS12,FAM13A-AS1,TRIM61,PLAC8,LRBA,EMCN,MAML3,COQ2,LOC101928978,MIR302C,TRIM60,LINC00989,PPID,DSPP,LOC729218,CENPE,FAM175A,CBR4,MIR7849,FAT4,GLRB,LINC01365,LOC101927087,MIR3139,NPY5R,LINC01091,NDUFC1,SCOC-AS1,LOC100507639,MIR1973,LEF1-AS1,GALNTL6,SH3RF1,HNRNPDL,MND1,LOC90768,PGRMC2,NEK1,LOC101929762,CCNI,MTTP,DEAR,MARCH1,SLC25A31,STPG2,MIR5705,IBSP,SNORD143,SMAD1-AS2,SEPT11,HSPA4L,NDST3,ZNF330,RBM46,LOC100507053,OSTC,FAM198B,LINC00499,SMAD1-AS1,CLCN3,LINC00613,WDFY3-AS2,UCP1,LOC101928509,HPGDS,TACR3,SLC9B1,LINC01088,MIR302A,ADH5,MAPK10,GK3P,CLGN,MGST2,FGB,HSD17B11,TMEM184C,TMEM155,LOC101927305,PTPN13,METAP1,PRMT9,CISD2,MIR4454,TIGD4,C4orf51,C4orf22,PDE5A,CXCL13,PALLD,KIAA1109,BBS12,MIR577,SCRG1,TLL1,SCD5,SPRY1,ETNPPL,VEGFC,DDIT4L,TNRC18P1,SEC24B,PPM1K,SOWAHB,SPATA5,LINC00575,GSTCD,MIR3140,HMGB2,COPS4,TNIP3,MIR4453,HPSE,ARSJ,LINC00616,LOC101929064,ARFIP1,TTC29,LINC01207,MRPS18C,LINC01612,LOC645513,MGARP,DCTD,PDLIM5,EDNRA,CCDC109B,LINC01099,SNORD144,FREM3,LSM6,ANXA5,ADH1A,TENM3,LOC100996286,DDX60L,MMRN1,CYP2U1,GPM6A,LOC101928551,FAM92A1P2,DMP1,FLJ20021,ANTXR2,MIR302B,INPP4B,TMEM150C,ASIC5,CDS1,PLA2G12A,FGF5,MIR3688-1,PLK4,MIR5096,ELF2,HERC3,UBE2D3,MIR6082,SNHG8,LOC101928052,LOC100506085,PCN	3	gain

1318	CGTE_18	5	81574028	125759596	5q15-q14.2	MIR548P,XRCC4,SEMA6A,DMXL1,NREP-AS1,LUCAT1,LINC01023,TMED7,LOC100289673,NREP,SRFBP1,TSSK1B,LINC01554,FAM174A,STARD4,POLR3G,MIR5706,TMEM161B,PGGT1B,MIR3977,VCAN,MEF2C,LOC731157,LNPEP,ARRDC3-AS1,ST8SIA4,SLC25A46,PAM,TTC37,MBLAC2,MIR548AO,TRIM36,SPATA9,SRP19,CEP120,FAM81B,LOC102546228,ARRDC3,EPB41L4A-AS1,PRDM6,EPB41L4A-AS2,PRR16,LOC102467212,LOC101927421,LINC01339,MGC32805,KIAA0825,MEF2C-AS1,REEP5,TMEM167A,EDIL3,SNORA13,TMED7-TICAM2,WDR36,MCTP1,MIR548F3,LOC102467216,CCNH,ZNF608,LOC102546226,LOC55338,MIR1244-2,MAN2A1,FER,NR2F1-AS1,SEMA6A-AS1,MIR9-2,DTWD2,PPIC,CAMK4,CETN3,LOC101927023,LOC102467214,LOC101927460,LINC01340,C5orf30,LINC01170,LOC100133050,AP3S1,CHD1,LOC102467226,SLCO4C1,MIR583,GPR150,SLCO6A1,LOC101927078,TMEM161B-AS1,RHOBTB3,LINC00492,ARSK,TSLP,HNCAT21,LOC100505841,CDO1,MIR3660,COMMD10,STARD4-AS1,CCDC112,FTMT,LIX1,RGMB-AS1,SNX2,SLF1,PJA2,MIR1244-1,HRAT56,LVRN,HAPLN1,EFNA5,LOC100505878,ELL2,TNFAIP8,LOC101927190,MIR1244-3,MIR1244-4,ARL14EPL,CAST,LOC101927100,GIN1,LOC102467224,PPIP5K2,LOC101929380,RFESD,LINC00491,GLRX,SNCAIP,FAM172A,LINC01338,LOC100289230,LOC102467213,LOC101929710,EPB41L4A,LYSMD3,NBPF22P,LINC00992,POU5F2,GRAMD3,COX7C,PCSK1,LOC102467217,LOC101927488,CTD-2151A2.1,DCP2,FBXL17,MIR3607,LOC101927379,MIR4280,LOX,ATP6AP1L,HSD17B4,ERAP2,RAB9BP1,TMEM232,SNX24,MIR2277,LOC102467223,TICAM2,LOC102467225,RPS23,FEM1C,NR2F1,ADGRV1,KCNN2,APC,CSNK1G3,RIOK2,ZNF474,NUDT12,FAM170A,YTHDC2,MCC,RGMB,LINC00461,RASA1,ERAP1,ATG12,SCARNA18,LOC644285	3	gain
1319	CGTE_18	5	126674797	130506801	5q23.2-q23.3	FBN2,ADAMTS19-AS1,MIR4633,ISOC1,MEGF10,LYRM7,PRRC1,HINT1,ADAMTS19,SLC12A2,SLC27A6,MIR4460,CTXN3,KIAA1024L,LINC01184,CHSY3	3	gain
1320	CGTE_18	5	140165831	140221533	5q31.3	PCDHA8,PCDHA2,PCDHA4,PCDHA6,PCDHA5,PCDHA1,PCDHA3,PCDHA7	6	gain
1321	CGTE_18	5	140221661	140238136	5q31.3	PCDHA8,PCDHA4,PCDHA9,PCDHA2,PCDHA6,PCDHA5,PCDHA7,PCDHA10,PCDHA1,PCDHA3	0	loss
1322	CGTE_18	5	140242428	140307679	5q31.3	PCDHA13,PCDHAC1,PCDHA6,PCDHA5,PCDHA11,PCDHA10,PCDHA7,PCDHA1,PCDHA3,PCDHA12,PCDHA8,PCDHA4,PCDHA9,PCDHA2	5	gain
1323	CGTE_18	5	140307683	140998466	5q31.3	PCDHA9,LOC101926905,PCDHB4,PCDHGB1,PCDHGC3,PCDHB18P,PCDHB8,PCDHA2,PCDHGA11,PCDHGB5,PCDHA4,TAF7,PCDHGA8,PCDHB2,LOC100505658,PCDHGA3,PCDHGC4,PCDHA12,PCDHA8,PCDHGB8P,PCDHGA9,PCDHB9,PCDHB11,PCDHGB3,PCDHB12,PCDHB14,PCDHB13,PCDHGA4,PCDHGA5,PCDHB19P,PCDHB15,PCDHB16,PCDHB7,PCDHB10,PCDHB3,PCDHA3,PCDHA1,PCDHGB7,PCDHGC5,PCDHGA10,PCDHGB6,PCDHB5,PCDHA7,PCDHGA2,SLC25A2,PCDHA11,PCDHB6,PCDHB1,PCDHA10,PCDHA5,PCDHA6,PCDHB17P,PCDHGB4,DIAPH1,PCDHAC1,PCDHGB2,PCDHAC2,PCDHGA7,PCDHGA6,PCDHA13,PCDHGA12,PCDHGA1	3	gain
1324	CGTE_18	5	142416744	148586700	5q32-q31.3	LOC102546294,PPP2R2B,JAKMIP2-AS1,LARS,SPINK6,ARHGAP26,PPP2R2B-IT1,KCTD16,HMHB1,SPINK9,SPINK5,FBXO38,ARHGAP26-IT1,GRXCR2,C5orf46,JAKMIP2,ABLIM3,TCERG1,RBM27,SPINK7,ADRB2,HTR4,DPYSL3,LOC255187,SPINK14,GPR151,POU4F3,SCGB3A2,SH3RF2,PLAC8L1,STK32A,PRELID2,SPINK1,YIPF5,SPINK13,MIR5197,SH3TC2,NR3C1	3	gain

1325	CGTE_18	6	203388	26017700	6p25.3-p24.3	LOC101928191,ALDH5A1,DTNBP1,ELOVL2-AS1,ELOVL2,MIR4639,HIST1H2APS1,RNF144B,LOC101927691,KDM1B,RBM24,FAM50B,LOC100507194,LINC00518,CD83,SLC22A23,NUP153,RNF182,MIR5689,BLOC1S5-TXNDC5,MIR5689HG,EEF1E1,LOC102724096,DEK,ADTRP,NEDD9,TDP2,SLC35B3,TUBB2A,TMEM170B,ATXN1,HDGFL1,NHLRC1,SERPINB1,LRRC16A,LOC285766,KIAA0319,MIR548A1,SYCP2L,SCARNA27,SLC17A2,NRSN1,WRNIP1,KIF13A,MRS2,ID4,PPP1R3G,SERPINB9P1,ACOT13,EDN1,PRL,TBC1D7,BLOC1S5,DUSP22,ERVFRD-1,GCM2,HIVEP1,CAP2,LOC101928491,CASC15,LYRM4,SSR1,JARID2,SLC17A3,LOC100130357,FOXCUT,LINC01600,NBAT1,IRF4,HULC,TMEM14C,HUS1B,FOXC1,RANBP9,LOC101928519,FAM65B,FOXQ1,MAK,TXNDC5,LINC01108,SCGN,NRN1,LOC100507506,RREB1,LOC100506207,LOC101928253,PAK1IP1,HIST1H2AA,NOL7,MIR6720,PHACTR1,LYRM4-AS1,TRIM38,CDKAL1,LOC101927972,SIRT5,FAM8A1,TFAP2A-AS1,EEF1E1-BLOC1S5,SLC17A4,STMND1,FAM217A,LY86-AS1,MIR4645,HTATSF1P2,C6orf229,LOC101927950,TPMT,ECI2,CAGE1,BPHL,LINC01622,HIST1H1A,MIR7641-2,PXDC1,RIOK1,LY86,C6orf201,SMIM13,DSP,LOC101928663,PRPF4B,MIR5683,MYLIP,MCUR1,RPP40,FARS2,SLC17A1,SERPINB9,F13A1,TFAP2A,CDYL,GMDS,KAAG1,EXOC2,GMDS-AS1,E2F3,FOXF2,GFOD1,RIPK1,LINC00581,KU-MEL-3,DCDC2,MBOAT1,NQO2,SERPINB6,HIST1H2BA,JARID2-AS1,GMNN,PSMG4,C6orf62,C6orf52,CMAHP,MIR3691,TUBB2B,GCNT2,MYLK4,GMPR,TMEM14B,SOX4,GPLD1,LOC101927730,BMP6,SNRNP48,PIP5K1P1,LOC101927759,LINC01011	3	gain
1326	CGTE_18	6	26017701	26285778	6p22.2	HIST1H3D,HIST1H1D,HIST1H4D,HIST1H2BH,HIST1H2BB,HIST1H3F,HIST1H4H,HIST1H2BF,HIST1H4F,HIST1H3A,HIST1H2AC,HIST1H2BC,HIST1H1T,HIST1H2AD,HIST1H2AE,HIST1H4E,HIST1H4C,HIST1H2BE,HIST1H3B,HIST1H3G,HIST1H4G,HIST1H3E,HIST1H2AB,HIST1H1A,HIST1H4B,HIST1H4A,HIST1H1E,HFE,HIST1H2BD,HIST1H2BI,HIST1H1C,HIST1H3C,HIST1H2BG	5	gain

1327	CGTE_18	6	26365492	33084975	6p21.33-p22.1	MICB,TUBB,CYP21A1P,VN1R10P,PSMB8-AS1,HCG14,MDC1,HCG23,BTN2A1,C2,DHX16,OR2H1,HIST1H2AI,HLA-DQB2,APOM,ABCF1,CSNK2B,MOG,ZBED9,FKBPL,OR12D2,C6orf15,SLC44A4,LY6G5B,TRIM39-RPP21,HCP5,TRIM31,NKAPL,MAS1L,HLA-L,LINC01623,IFITM4P,EHMT2,HIST1H4J,CCHCR1,LSM2,OR2B2,ZSCAN12P1,HLA-F,HIST1H4I,MCCD1,AGER,NOTCH4,RPP21,GNL1,HSPA1A,PSMB9,ZNF391,TRIM15,PPP1R18,NCR3,HLA-DQB1,ZKSCAN8,ATP6V1G2-DDX39B,LST1,MIR4646,TAP2,TNXB,DDX39B-AS1,SNORD48,ZNF184,HLA-DMA,HCG18,PPP1R11,HSPA1L,ZKSCAN4,TAP1,GTF2H4,HLA-H,OR2B6,HIST1H2BN,TCF19,GPSM3,MIR3143,HLA-E,MIR3135B,LOC100129636,HIST1H2AJ,LINC00243,ZSCAN26,HIST1H4K,BTNL2,HIST1H2BM,LTA,GPANK1,MIR6891,VARS,HLA-DOB,CYP21A2,CLIC1,OR5V1,AGPAT1,NRM,PSORS1C2,BTN3A3,ZNF204P,DDR1,HCG22,SKIV2L,ABHD16A,POM121L2,MIR6832,ZSCAN9,SAPCD1,OR2W1,SNORD117,HLA-DQA1,PRRC2A,HLA-DRB5,C4A,OR2J3,SFTA2,MSH5,HIST1H2AG,LOC100131289,HCG4B,HLA-J,ZNF311,HLA-C,LOC100294145,CFB,OR14J1,FLOT1,ZSCAN23,LY6G6F,PRR3,OR10C1,C6orf47,HCG4,HIST1H1B,TRIM39,HCG27,AIF1,HCG26,SAPCD1-AS1,NEU1,DDAH2,HLA-DMB,HCG11,ATAT1,MRPS18B,HLA-DRA,MUC21,HLA-DRB6,LTB,HIST1H3I,ZSCAN16-AS1,RNF5,OR11A1,MIR877,MIR6721,MIR6833,BRD2,C6orf48,NELFE,HSPA1B,TRIM10,HIST1H4L,HLA-DPB2,HLA-DRB1,PPP1R10,MSH5-SAPCD1,BTN3A2,ZNF165,HIST1H3H,UBD,TRIM31-AS1,HMGN4,OR2B3,TNXA,PBX2,OR2J2,OR2H2,ZFP57,LOC100507547,BTN2A3P,TRIM27,DDX39B,PPT2-EGFL8,HIST1H2AM,LY6G6D,C6orf10,HLA-DQA2,HCG17,OR12D3,NFKBIL1,GPX6,LINC00240,GPX5,BTN3A1,BTN1A1,HIST1H2BJ,ZBTB12,HIST1H2BK,GUSBP2,PRSS16,LINC01015,LINC01556,ZNRD1,POU5F1,PSORS1C3,BAG6,TNF,MIR4640,HIST1H2BL,ATF6B,LY6G6C,LOC285819,ABT1,HLA-DOA,TRIM26,C4B_2,ATP6V1G2,CDSN,PGBD1,HIST1H3J,HIST1H2AL,TOB2P1,DXO,HCG9,HIST1H2AK,MUC22,HLA-B,ZKSCAN3,TRIM40,MIR1236,PRRT1,DPCR1,VARS2,GABBR1,BTN2A2,LY6G5C,PSMB8,C4B,LY6G6E,HIST1H2AH,RNF5P1,C2-AS1,C6orf25,ZSCAN12,ZNRD1-AS1,HLA-DPA1,HLA-G,PPT2,SNORD52,ZSCAN16,VWA7,LINC01012,LOC554223,HLA-A,RNF39,LOC100270746,STK19,HIST1H2BO,C6orf136,HCG8,SNORD32B,SNORA38,ZNF322,HLA-DPB1,ZSCAN31,MDC1-AS1,IER3,EGFL8,PSORS1C1,SNORD84,ZNF192P1,HLA-F-AS1,MICA	3	gain

1328	CGTE_18	6	44280544	74104893	6p21.1-q13	GLYATL3,AARS2,BMP5,TFAP2D,EYS,TMEM14A,IL17F,MLIP,CENPQ,LOC102723883,LINC00680,EVADR,LINC00680-GUSBP4,KHDRBS2,RAB23,GCLC,DEFB133,TRAM2,KHDC3L,LRRC1,GSTA7P,TNFRSF21,GSTA1,PLA2G7,FAM83B,MIR30A,MLIP-IT1,LGSN,GSTA2,LOC101927048,RCAN2,CRISP2,LOC441155,RHAG,ADGRF1,ANKRD66,MIR5685,CD2AP,PTCHD4,MIR30C2,PGK2,BEND6,LINC01564,MIR4282,GCM1,LINC00472,LOC101926962,LINCMD1,CYP39A1,OPN5,OGFRL1,ADGRF4,ADGRF2,B3GAT2,ADGRB3,BAG2,LOC101930010,MCM3,CDC5L,KCNQ5-AS1,MUT,MIR4642,GSTA4,KLHL31,HCRTR2,RPS16P5,LMBRD1,RUNX2,COL19A1,PHF3,LOC730101,LOC101927136,KHDC1L,COL21A1,FBXO9,C6orf141,EFHC1,SLC25A51P1,LOC101927189,SMAP1,MIR133B,CRISP1,SPATS1,GUSBP4,ICK,PTP4A1,SDHAF4,KIAA1586,GFRAL,FAM135A,KHDC1,TINAG,DDX43,PAQR8,DEFB113,LOC101926898,GSTA3,RIMS1,TFAP2B,DPPA5,MIR548U,LOC101928280,MTRNR2L9,CRISP3,KCNQ5,OOEP,DEFB110,ELOVL5,LOC101927082,ENPP4,ENPP5,PKHD1,MIR586,TDRD6,PRIM2,LOC101926915,COL9A1,KCNQ5-IT1,CLIC5,SLC25A27,MEP1A,SUPT3H,ADGRF5,LOC101927211,DEFB112,DST,DEFB114,TRAM2-AS1,LOC100506188,IL17A,HMGCLL1,ZNF451,LOC101928307,MIR206,LOC101927020,GSTA5,LINC01626	3	gain
1329	CGTE_18	6	76600922	86160243	6q14.3-q14.1	LINC01611,RNY4,ME1,LINC01526,SNAP91,DOPEY1,MRAP2,PGM3,HTR1B,IMPG1,HMGN3,BCKDHB,LINC01621,IBTK,SH3BGRL2,TTK,CYB5R4,TBX18,MEI4,TBX18-AS1,MYO6,UBE3D,PRSS35,CEP162,ELOVL4,LCAL1,TPBG,HMGN3-AS1,PHIP,RWDD2A,LCA5,FAM46A,IRAK1BP1,RIPPLY2,NT5E	3	gain
1330	CGTE_18	7	6746040	17962384	7p22.1-p21.1	DGKB,MIR3683,LOC100505938,LOC100131257,AGMO,COL28A1,PER4,AGR2,ICA1,RSPH10B2,AGR3,MEOX2-AS1,LOC101927391,KCCAT333,CCZ1B,PHF14,ISPD-AS1,MIOS,LRRC72,RPA3,GLCCI1,TMEM106B,THSD7A,LOC100505921,UMAD1,PMS2CL,ETV1,AHR,C1GALT1,TSPAN13,LOC101927630,ZNF12,ANKMY2,MEOX2,BZW2,SOSTDC1,NXPH1,ARL4A,NDUFA4,SNX13,RSPH10B,LOC101927354,SCIN,VWDE,ISPD	4	gain
1331	CGTE_18	7	17979868	19185164	7p21.1	SNX13,TWIST1,MIR1302-6,FERD3L,HDAC9,PRPS1L1	5	gain

1332	CGTE_18	7	19737967	43918909	7p13-p15.3	NPVF,DKFZP586I1420,NPSR1-AS1,EVX1-AS,HOXA11-AS,SCRN1,LINC01176,SUGCT,POU6F2,MPLKIP,MRPL32,ABCB5,RALA,COA1,KIAA0087,SNX10,TRA2A,NT5C3A,LOC541472,EVX1,RP9,FAM183BP,CBX3,MIR550A1,DPY19L2P1,ADCYAP1R1,DNAH11,MIR550A3,ANLN,HOXA10-HOXA9,MIR1200,SEPT7,BBS9,LINC00997,LOC441204,GLI3,RPL23P8,KBTBD2,TOMM7,LINC01450,NUPL2,CCDC126,LINC01448,STARD3NL,MIR3146,PDE1C,BMPER,HOXA2,MTURN,AQP1,CHN2,LOC401320,DPY19L1P2,ZNRF2P1,HOXA11,HOXA5,DPY19L1,CDCA7L,KLHL7-AS1,CREB5,NOD1,CRHR2,FKBP14,TRIL,LOC401324,DFNA5,HOXA6,LINC01162,TBX20,URGCP-MRPS24,SNORD93,EPDR1,ZNRF2,MIR196B,MALSU1,IL6,SP8,HOXA10-AS,AMPH,YAE1D1,HOXA3,NEUROD6,FAM126A,MIR148A,HOXA9,JAZF1,GPNMB,TRG-AS1,TMEM196,INHBA-AS1,GHRHR,JAZF1-AS1,CYCS,HOTTIP,CLK2P1,AOAH,LOC100130673,FKBP9,LOC100506497,MIR3943,LINC00265,ITGB8,ELMO1-AS1,MIR550B2,LOC100506725,PLEKHA8,CPVL,C7orf25,MIR550B1,KLHL7,VPS41,POU6F2-AS1,SFRP4,WIPF3,GGCT,GARS,HECW1,LOC646762,HERPUD2,INMT-FAM188B,HOXA13,NPY,KIAA0895,GPR141,LOC101927811,HIBADH,AVL9,RAPGEF5,PPP1R17,HOXA7,LINC01449,LOC102724484,MACC1-AS1,NME8,MACC1,SKAP2,AOAH-IT1,RP9P,URGCP,LOC101928618,MPP6,OSBPL3,DPY19L2P3,TAX1BP1,RPS2P32,LOC100506895,STK17A,STEAP1B,LOC100506178,CDK13,TWISTNB,INHBA,STK31,FAM188B,PRR15,NFE2L3,HOXA10,HOTAIRM1,BLVRA,ELMO1,FAM221A,TSL,CCDC129,ZNRF2P2,LOC101927769,HOXA-AS2,IGF2BP3,DPY19L1P1,LOC101928168,HOXA-AS3,SEPT7-AS1,MRPS24,MIR550A2,SP4,HNRNPA2B1,LOC401312,LSM5,LOC101927668,HOXA4,INMT,TARP,EEPD1,HOXA1,NPSR1,MIR1183,PSMA2,C7orf31,C7orf71	3	gain
1333	CGTE_18	7	48068353	55087073	7p12.2-p12.3	C7orf72,UPP1,SEC61G,HPVC1,LINC01446,VSTM2A,VWC2,CDC14C,COBL,DDC-AS1,GRB10,POM121L12,FIGNL1,IKZF1,C7orf57,SUN3,ABCA13,VSTM2A-OT1,EGFR,DDC,ZPBP,LOC100996654,LINC01445	3	gain

1334	CGTE_18	7	56174120	77755167	7p11.1-q21.11	APTR,PHTF2,LAT2,CCDC146,LINC01372,LOC101926943,MIR4650-1,LOC100996437,NUPR2,LOC641746,MIR3147,GS1-124K5.11,ZNF117,VPS37D,NCF1,CLIP2,PMS2P3,HIP1,ZNF680,FKBP6,GATSL2,LOC401357,UPK3B,SPDYE5,CHCHD2,ZNF138,RABGEF1,ZNF479,ZNF107,ABHD11-AS1,FZD9,NCF1B,AUTS2,MIR590,ZNF727,LOC100128885,POMZP3,CCT6P1,RFC2,ZNF735,CRCP,SNORA22,LOC101927243,PMS2P4,GTF2IRD2,TRIM74,SBDS,ASL,CALN1,MLXIPL,CLDN3,YWHAEP1,WBSCR22,STAG3L4,MDH2,NCF1C,GTF2IP4,VKORC1L1,TMEM60,NSUN5,CCL26,STAG3L3,BCL7B,MIR3914-2,DTX2P1-UPK3BP1-PMS2P11,DKFZp434L192,PMS2P2,BAZ1B,STX1A,TBL2,CCL24,LOC100133091,ELN,TRIM73,EIF4H,TYW1,ZNF273,STYXL1,LOC101928401,CCT6P3,ZNF716,HSPB1,LOC100287834,LOC100287704,RSBN1L,SPDYE8P,TRIM50,FGL2,POM121,MIR3914-1,TPST1,DTX2,LOC100240728,KCTD7,INTS4P2,GTF2IRD1,DNAJC30,CLDN4,GTF2IRD2B,LINC00174,TMEM248,PTPN12,SBDSP1,POM121C,GUSBP10,MIR4283-2,YWHAG,LOC441242,MIR4284,SRRM3,PMS2P7,SPDYE7P,MIR4651,TYW1B,LOC100130849,ZNF92,PMS2P5,ZNF679,MIR4283-1,SNORA14A,PMS2P9,GSAP,ZNF736,LOC650226,ABHD11,LOC102723427,GTF2I,GTF2IP1,NSUN5P1,SSC4D,LOC100101148,NSUN5P2,POR,LOC541473,RHBDD2,FDPSP2,STAG3L1,MIR4650-2,GTF2IRD1P1,LOC100507468,LINC01005,WBSCR16,MAGI2,STAG3L2,ZP3,ERV3-1,WBSCR28,TMEM120A,WBSCR17,ZNF733P,MIR6839,LIMK1,WBSCR27,GUSB	4	gain
1335	CGTE_18	7	77756570	86817676	7q21.11-q21.12	GNAI1,MAGI2-AS2,LOC101927378,GNAT3,CD36,CACNA2D1,RPL13AP17,SEMA3A,LOC101927356,LOC101927269,MAGI2-AS3,LOC100128317,GRM3,SEMA3D,HGF,DMTF1,PCLO,SEMA3C,MIR548M,SEMA3E,MAGI2,KIAA1324L	5	gain
1336	CGTE_18	7	86820235	92735394	7q21.12-q21.13	MGC16142,LOC102723885,FAM133B,CLDN12,TP53TG1,AKAP9,ABCB1,MTERF1,DPY19L2P4,ANKIB1,LOC101927497,PEX1,CDK14,FZD1,C7orf62,CDK6,ZNF804B,RBM48,STEAP4,KRIT1,ABCB4,SLC25A40,CYP51A1,CFAP69,STEAP1,CROT,DBF4,CYP51A1-AS1,LOC101927446,STEAP2-AS1,TMEM243,GTPBP10,RUNDC3B,ADAM22,GATAD1,LOC101409256,SRI,FAM133DP,LRRD1,SAMD9,STEAP2,DMTF1	4	gain
1337	CGTE_18	7	92760508	95499368	7q21.3-q21.2	ASB4,PDK4,MIR4652,PON1,PON3,COL1A2,SAMD9L,PON2,MIR489,CASD1,PEG10,GNG11,TFPI2,CALCR,HEPACAM2,MIR653,SGCE,VPS50,GNGT1,BET1,DYNC1I1,PPP1R9A	3	gain
1338	CGTE_18	7	95606672	99690797	7q21.3-q22.1	DLX6-AS1,DYNC1I1,COPS6,MIR3609,SDHAF3,ZNF789,CYP3A7-CYP3A51P,CYP3A5,ATP5J2,ZSCAN21,DLX5,MIR591,MIR5692A2,SMURF1,ZKSCAN1,ATP5J2-PTCD1,FAM200A,SLC25A13,DLX6,MGC72080,ZNF394,GS1-259H13.2,ARPC1B,SCARNA28,TECPR1,LMTK2,CYP3A4,C7orf76,TAC1,ASNS,CYP3A7,TRRAP,PTCD1,GJC3,NPTX2,PDAP1,AZGP1,BRI3,BAIAP2L1,CPSF4,LOC101927550,OR2AE1,KPNA7,TMEM130,ZNF655,MYH16,ZSCAN25,ARPC1A,SHFM1,BHLHA15,ZKSCAN5,BUD31,MIR5692A1,MCM7,LOC100506136,AZGP1P1,OCM2,CYP3A43,ZNF3,TRIM4	6	gain

1339	CGTE_18	7	106508558	123254744	7q31.1-q31.32	LAMB4,NRCAM,NDUFA5,IFRD1,MDFIC,TFEC,LSM8,HBP1,FEZF1,COG5,SLC26A4,ST7-AS1,SLC26A3,WNT16,C7orf66,LOC100996249,FOXP2,ST7-OT3,KCND2,CBLL1,BCAP29,SLC13A1,CAPZA2,LOC101928012,DUS4L,LINC01392,TMEM168,CFTR,FAM3C,SLC26A4-AS1,CADPS2,GPR22,LINC01393,EIF3IP1,PRKAR2B,FEZF1-AS1,PTPRZ1,ASB15,IQUB,PPP1R3A,MIR6132,MET,CAV2,THAP5,DLD,TSPAN12,TES,LSMEM1,DOCK4,DOCK4-AS1,CTTNBP2,WNT2,AASS,LAMB1,LINC01510,ASZ1,IMMP2L,CPED1,ZNF277,RNF148,HRAT17,LRRN3,ST7-OT4,RNF133,C7orf60,LOC102724555,LVCAT5,CAV1,MIR3666,LOC102724434,DNAJB9,PIK3CG,LINC00998,ANKRD7,ST7-AS2,GPR85,TAS2R16,ST7,ING3,PNPLA8	3	gain
1340	CGTE_18	7	128851536	133261628	7q32.2-q33	MIR335,TMEM209,TSGA13,MEST,ZC3HC1,AHCYL2,MKLN1-AS,CPA5,PLXNA4,SSMEM1,EXOC4,MIR3654,SMKR1,KLF14,LOC100506860,MIR29A,CEP41,NRF1,SMO,MIR96,MIR182,COPG2,CPA1,CPA4,CHCHD3,MIR6133,CPA2,MIR183,MKLN1,LOC101928782,LINC-PINT,UBE2H,MESTIT1,PODXL,MIR29B1,FLJ40288,STRIP2,KLHDC10	3	gain
1341	CGTE_18	7	133314740	134262612	7q33	SLC35B4,AKR1B15,LOC101928861,AKR1B10,AKR1B1,LRGUK,EXOC4	6	gain
1342	CGTE_18	8	190799	2796467	8p23.2-p23.3	MIR7160,DLGAP2,ERICH1-AS1,KBTBD11-OT1,ZNF596,MIR596,ERICH1,MIR3674,MYOM2,LOC286083,DLGAP2-AS1,LOC101927815,FAM87A,CSMD1,FBXO25,TDRP,KBTBD11,ARHGEF10,CLN8	1	loss
1343	CGTE_18	8	49647558	67345001	8q12.1-q12.2	LYPLA1,ARMC1,MIR4470,RRS1-AS1,LOC286177,TGS1,PCMTD1,FAM150A,CYP7A1,RPS20,MIR124-2HG,LOC100130298,NSMAF,CA8,ADHFE1,FAM110B,GGH,OPRK1,PXDNL,PDE7A,CHCHD7,ASPH,UG0898H09,CLVS1,IMPAD1,MRPL15,XKR4,CRH,TRIM55,NPBWR1,LOC101929415,RRS1,LINC01299,ATP6V1H,RP1,LOC102724612,ST18,RB1CC1,LINC01289,LOC101929528,TOX,MIR124-2,SNORD54,CYP7B1,C8orf22,RGS20,LOC401463,LYN,MTFR1,EFCAB1,SBF1P1,DNAJC5B,SDR16C5,LINC00588,YTHDF3-AS1,SNAI2,CHD7,LOC101929488,BHLHE22,SNTG1,SOX17,YTHDF3,UBXN2B,NKAIN3,RAB2A,TMEM68,LINC01606,LINC00251,MOS,LINC00968,PLAG1,TCEA1,SDCBP,SDR16C6P,LINC00967,PENK,LOC102724623,LINC01301,LINC01602,TTPA	3	gain
1344	CGTE_18	8	68658114	91090740	8q13.2-q21.3	RPL7,LOC392232,ZBTB10,CALB1,ZFHX4-AS1,TRAM1,MSC,MIR5708,STAU2,ZFAND1,ZNF704,UBE2W,LOC101927040,RDH10-AS1,C8orf59,LOC101241902,WWP1,CA2,JPH1,LOC101926892,PREX2,MSC-AS1,MIR2052HG,MIR5681A,SBSPON,RALYL,ATP6V0D2,TMEM70,C8orf34,GDAP1,PMP2,CNGB3,MIR2052,IL7,LACTB2,MMP16,TCEB1,LOC102724874,DECR1,TRPA1,CA1,STAU2-AS1,MIR3149,STMN2,XKR9,OSGIN2,FABP4,FABP9,FABP12,MRPS28,ZC2HC1A,LRRCC1,ZFHX4,RIPK2,CASC9,LOC101926908,C8orf34-AS1,NCOA2,PAG1,TPD52,SLC10A5,CNBD1,RDH10,RMDN1,LOC101929709,PSKH2,EYA1,PRDM14,CRISPLD1,HNF4G,PKIA,LACTB2-AS1,LINC01607,NBN,CPNE3,E2F5,CPA6,LINC01592,SLC7A13,C8orf89,SLCO5A1,HEY1,CA3-AS1,LINC01419,CHMP4C,DCAF4L2,LINC01111,PKIA-AS1,SULF1,SNX16,CA13,MIR5681B,FABP5,KCNB2,LY96,CA3,PEX2,LINC01603,REXO1L2P,TERF1,PI15,IMPA1	3	gain

1345	CGTE_18	8	91094171	94179120	8q22.1-q21.3	OTUD6B-AS1,LRRC69,C8orf88,LINC01030,TMEM55A,CALB1,NECAB1,TRIQK,TMEM64,MIR8084,SLC26A7,LOC102724710,FLJ46284,LINC00534,OTUD6B,RUNX1T1,MIR7641-2,C8orf87,MIR4661	4	gain
1346	CGTE_18	8	94358957	104420025	8q22.1-q22.3	C8orf37,MIR1273A,KLF10,FZD6,FAM92A1,STK3,GEM,PABPC1,YWHAZ,TSPYL5,TP53INP1,NCALD,MIR3151,GRHL2,MIR875,FSBP,ERICH5,ZNF706,MIR7705,LOC100288748,LAPTM4B,RNF19A,NACAP1,CPQ,GDF6,PDP1,LOC101927066,RBM12B,CDH17,LOC100500773,FLJ42969,KCNS2,HRSP12,INTS8,MIR5680,ESRP1,LINC01298,LINC00535,MIR3150B,RRM2B,UBR5,PLEKHF2,LOC104054148,SNORA72,SLC25A32,MIR378D2,UQCRB,CTHRC1,RBM12B-AS1,FBXO43,POP1,ANKRD46,COX6C,LOC105375650,SPAG1,BAALC-AS1,MIR4471,ATP6V1C1,MIR599,ODF1,PTDSS1,LOC102724804,AZIN1,AZIN1-AS1,VPS13B,RGS22,NIPAL2,MTERF3,NDUFAF6,SNX31,POLR2K,BAALC-AS2,DPY19L4,C8orf37-AS1,CCNE2,UBR5-AS1,TMEM67,MATN2,MTDH,RAD54B,KIAA1429,OSR2,BAALC,MIR3150A,RPL30,SDC2	3	gain
1347	CGTE_18	8	104427000	120759194	8q23.1-q22.3	TAF2,ZFPM2,RIMS2,SLC30A8,SAMD12-AS1,EXT1,NOV,PKHD1L1,RAD21,DCAF13,MIR3610,LINC01609,MIR2053,SAMD12,ENY2,ENPP2,KCNV1,ANGPT1,UTP23,ABRA,EIF3E,LRP12,MED30,EMC2,CSMD3,RAD21-AS1,ZFPM2-AS1,LINC01608,TRPS1,TRHR,COLEC10,OXR1,TNFRSF11B,SYBU,SLC25A32,DPYS,TMEM74,AARD,EIF3H,LINC00536,MAL2,NUDCD1,EBAG9,DCSTAMP,RSPO2	4	gain
1348	CGTE_18	8	120768230	120865474	8q24.12	DSCC1,TAF2	3	gain
1349	CGTE_18	8	120867712	124027896	8q24.12-q24.13	DSCC1,ZHX2,HAS2-AS1,SNTB1,MRPL13,COL14A1,DERL1,MTBP,LINC01151,DEPTOR,LOC101927543,LOC105375734,HAS2	4	gain
1350	CGTE_18	8	124031191	133807104	8q24.22-q24.13	FAM84B,LINC00861,EFR3A,NSMCE2,MIR1205,CASC8,RNF139,MTSS1,ANXA13,ASAP1-IT1,MIR1208,OC90,MIR5194,LINC00977,ZHX1,TMEM71,MIR4662A,TRIB1,GSDMC,MIR3686,FAM49B,KLHL38,TRMT12,NDUFB9,KIAA0196,FBXO32,CCAT1,ADCY8,TBC1D31,MIR4662B,PCAT1,TATDN1,FER1L6-AS2,MIR6844,ASAP1,MIR1204,SQLE,FAM83A,LRRC6,FER1L6-AS1,MIR4663,FAM91A1,LOC101927588,DERL1,PCAT2,CASC19,FER1L6,C8orf76,FAM83A-AS1,LINC00824,CASC21,ATAD2,PVT1,CCDC26,MYC,WDYHV1,ZNF572,HHLA1,MIR1206,PHF20L1,TMEM75,ZHX1-C8orf76,MIR1207,ASAP1-IT2,TMEM65,CCAT2,RNF139-AS1,POU5F1B,KCNQ3,PRNCR1,LINC00964,HPYR1,LOC101927657,CASC11	3	gain
1351	CGTE_18	8	133810996	133882076	8q24.22	PHF20L1,TG	5	gain
1352	CGTE_18	8	133883543	142146838	8q24.22-q24.23	ST3GAL1,KCNK9,COL22A1,TG,LOC101927915,MIR7848,LOC101927822,SLA,PTK2,NCRNA00250,MIR30D,AGO2,ZFAT-AS1,LOC101927798,ZFAT,MIR30B,WISP1,NDRG1,CHRAC1,LINC01591,LOC101927845,DENND3,FAM135B,KHDRBS3,TRAPPC9	3	gain
1353	CGTE_18	8	145806187	146279543	8q24.3	RPL8,ZNF16,ZNF251,MIR6850,ZNF252P-AS1,ARHGAP39,C8orf33,ZNF252P,ZNF250,TMED10P1,ZNF517,COMMD5,ZNF34,ZNF7	3	gain

1354	CGTE_18	9	71079983	85905692	9q21.31-q21.12	LOC101927358,TLE1,BANCR,PTAR1,TMC1,LINC01506,GNAQ,MAMDC2,TMEM2,OSTF1,C9orf135,TJP2,MIR204,MAMDC2-AS1,CEP78,CARNMT1,GCNT1,PRKACG,PRUNE2,KLF9,PIP5K1B,RORB,SPATA31D4,LINC01507,TMEM252,TRPM3,SPATA31D5P,APBA1,LOC101927502,LINC01504,RORB-AS1,SPATA31D1,TLE4,FRMD3,GNA14-AS1,C9orf135-AS1,C9orf40,PGM5,PSAT1,VPS13A,ZFAND5,ANXA1,RFK,GDA,VPS13A-AS1,GNA14,NMRK1,LOC101927069,FAM122A,RPSAP9,ABHD17B,MIR6130,MIR548H3,C9orf57,FAM189A2,FOXB2,LOC101927450,PCSK5,SMC5-AS1,SPATA31D3,RASEF,SMC5,PCA3,TRPM6,ALDH1A1,LINC01474,C9orf85,C9orf41-AS1,FXN	3	gain
1355	CGTE_18	10	27702148	27793808	10p12.1	PTCHD3,RAB18	7	gain
1356	CGTE_18	10	32344735	33623497	10p11.22	LOC102031319,NRP1,SNORA86,EPC1,ITGB1,CCDC7,LOC101929431,KIF5B	64	gain
1357	CGTE_18	10	35814757	35858202	10p11.21	CCNY	13	gain
1358	CGTE_18	10	52002917	62671364	10q21.1-q21.2	FAM133CP,LINC00844,LINC01468,CCDC6,ASAH2B,TFAM,MIR605,BICC1,SLC16A9,ANK3,PRKG1-AS1,IPMK,MBL2,PHYHIPL,ZWINT,DKK1,CSTF2T,LOC102724719,FAM13C,CDK1,MRLN,PCDH15,ASAH2,PRKG1,A1CF,SGMS1,SGMS1-AS1,LINC01553,MTRNR2L5,MIR3924,RHOBTB1,UBE2D1,CISD1,CCEPR	3	gain
1359	CGTE_18	10	63170121	64136835	10q21.2	MIR548AV,ZNF365,TMEM26-AS1,C10orf107,RTKN2,TMEM26,LOC283045,ARID5B	16	gain
1360	CGTE_18	10	65132713	65383499	10q21.3	JMJD1C-AS1,MIR1296,REEP3,JMJD1C	11	gain
1361	CGTE_18	10	65383534	69366814	10q21.3	LRRTM3,CTNNA3,REEP3,LOC101928961,LINC01515,MIR7151,ANXA2P3	3	gain
1362	CGTE_18	10	78704574	80763620	10q22.3	DLG5,KCNMA1-AS1,KCNMA1,LINC00856,POLR3A,KCNMA1-AS3,ZMIZ1-AS1,LINC00595,RPS24,KCNMA1-AS2,DLG5-AS1	11	gain
1363	CGTE_18	10	121717942	128594159	10q26.13-q26.12	PLEKHA1,ATE1,DOCK1,ACADSB,FLJ37035,FANK1-AS1,PSTK,FGFR2,DMBT1,GPR26,BTBD16,EDRF1-AS1,TEX36,NSMCE4A,CUZD1,DHX32,HMX3,C10orf120,LINC01561,FAM24B,FAM24A,BUB3,MIR5694,LOC283038,FAM24B-CUZD1,FAM53B,ADAM12,WDR11,HMX2,CPXM2,TACC2,MIR4682,UROS,FAM175B,MIR3941,MIR4484,ZRANB1,FANK1,HTRA1,MIR4296,CHST15,TEX36-AS1,C10orf90,LOC399815,BCCIP,NKX1-2,CTBP2,PLPP4,OAT,IKZF5,ARMS2,LHPP,WDR11-AS1,C10orf88,ATE1-AS1,METTL10,FAM53B-AS1,MMP21,DMBT1P1,EDRF1,LINC00601	12	gain

1364	CGTE_18	11	193051	4185675	11p15.4-p15.5	LINC01150,LOC143666,SNORA54,FAM99A,MRGPRE,HOTS,SNORA52,KRTAP5-1,KRTAP5-4,LRRC56,ZNF195,MIR675,SNORD131,CDHR5,RNH1,POLR2L,PHRF1,KRTAP5-6,ATHL1,DRD4,PKP3,TALDO1,KCNQ1,DUSP8,IFITM3,PDDC1,PGAP2,KRTAP5-AS1,MIR4686,KCNQ1DN,MRPL23-AS1,TOLLIP-AS1,MIR210,INS-IGF2,PSMD13,KRTAP5-2,SIRT3,ASCL2,IRF7,MIR210HG,LOC101927708,KCNQ1OT1,RASSF7,TSPAN32,HRAS,CD81-AS1,PIDD1,KRTAP5-3,EPS8L2,C11orf21,IGF2-AS,MIR6744,MUC6,BRSK2,CRACR2B,ODF3,AP2A2,TOLLIP,IFITM1,RRM1,MUC5AC,LINC01219,TMEM80,BET1L,CTSD,TRPM5,TH,LOC171391,TSSC2,SYT8,NUP98,SCT,IFITM5,LSP1,H19,MRGPRG-AS1,MIR483,SLC22A18,PNPLA2,NAP1L4,CHID1,TSPAN4,STIM1,B4GALNT4,PANO1,OSBPL5,SCGB1C1,IFITM10,MIR4687,ANO9,DEAF1,MUC2,TNNT3,TNNI2,KCNQ1-AS1,CARS,SLC22A18AS,TSSC4,MRPL23,CEND1,MIR4298,SLC25A22,KRTAP5-5,CD81,NLRP6,PHLDA2,CHRNA10,SCGB1C2,MIR6743,MOB2,PTDSS2,OR7E12P,RIC8A,CDKN1C,SIGIRR,CD151,IFITM2,ART1,IGF2,RHOG,MUC5B,INS,RPLP2,TRPC2,FAM99B,MIR7847,LMNTD2,MRGPRG,ART5	1	loss
1365	CGTE_18	11	4388593	6412189	11p15.4	OR51A4,OR52W1,OR56B1,OR52B6,OR56A1,OR51E1,OR52E8,OR51A7,UBQLNL,UBQLN3,OR51Q1,OR52K1,OR52E4,OR52A5,OR52N5,TRIM34,CCKBR,OR52E6,OR52M1,HBE1,OR56A5,OR51I2,OR56A3,OR52N1,OR52N2,OR52I1,C11orf40,OR56B4,OR52J3,OR51F2,HBD,OR52I2,OR51B5,OR52R1,OR52L1,TRIM68,OLFM5P,OR51B6,OR51G1,OR51B4,OR51L1,HBBP1,TRIM5,OR51M1,FAM160A2,TRIM21,SMPD1,OR51E2,OR51D1,OR52D1,OR52H1,MMP26,TRIM6-TRIM34,OR52A1,OR52N4,OR51A2,BGLT3,OR51S1,CNGA4,TRIM6,OR51I1,OR52K2,HBG1,C11orf42,TRIM22,PRKCDBP,OR51B2,OR51T1,HBB,OR51V1,HBG2,OR51F1,OR52E2,OR52B2,OR56A4,OR51G2,OR52B4	3	gain
1366	CGTE_18	11	14913496	16766146	11p15.2-p15.1	INSC,CYP2R1,C11orf58,CALCB,SOX6,MIR6073,LOC102724957,CALCA	3	gain
1367	CGTE_18	11	18978527	19138931	11p15.1	ZDHHC13,MRGPRX2	4	gain
1368	CGTE_18	11	48238466	57004521	11p11.12-q11	OR5D16,OR5D18,TRIM51,OR8U8,OR5M3,MIR6128,LOC441601,OR5M9,TRIM64C,OR5I1,OR5AP2,LRRC55,APLNR,TRIM51HP,OR5W2,OR8K3,OR9G4,OR8H1,OR4C11,LOC646813,OR4B1,OR4A5,OR7E5P,OR4C45,OR4C46,TRIM48,OR5M10,OR4C12,OR5AS1,OR8J3,OR5D14,OR5T1,OR8U1,OR5AR1,OR5M8,OR4C13,OR4A47,OR4A16,OR4X1,OR8J1,OR5AK2,OR4C6,OR10AG1,OR5M1,LOC101927120,OR8I2,OR4C3,OR8K5,OR8H3,OR5J2,OR4C16,OR5T3,TRIM49B,OR8K1,OR4A15,OR5F1,OR4P4,LOC440040,OR5L2,OR5R1,OR4S2,OR4S1,OR4X2,OR5AK4P,OR5T2,OR8H2,OR4C15,OR9G1,OR5L1,FOLH1,OR5D13,OR5M11,OR9G9	3	gain
1369	CGTE_18	11	57798775	58723173	11q12.1	OR5B2,LPXN,OR5B12,GLYATL1,OR6Q1,OR9I1,OR5B3,CNTF,OR10W1,LOC283194,OR5B17,ZFP91,OR9Q2,OR10Q1,OR5B21,OR9Q1,OR1S1,GLYAT,OR1S2,ZFP91-CNTF,GLYATL2	3	gain
1370	CGTE_18	11	62848412	63258479	11q12.3	SLC22A10,SLC22A9,SLC22A24,HRASLS5,SLC22A25	3	gain
1371	CGTE_18	11	73716730	74800863	11q13.4	LIPT2,PGM2L1,PPME1,KCNE3,NEU3,SPCS2,POLD3,P4HA3,MIR4696,OR2AT4,XRRA1,CHRDL2,LOC101928580,C2CD3,RNF169,UCP3	3	gain

1372	CGTE_18	11	78285310	107799576	11q14.2-q22.2	RAB38,TRIM77,MIR1260B,GPR83,NOX4,UBTFL1,CWF19L2,MIR548L,PCF11,MTNR1B,DISC1FP1,CEP57,PRSS23,LOC101928944,ANKRD49,TAF1D,TRIM49,GUCY1A2,MIR3166,C11orf54,CARD18,TRIM64,TMEM135,GRM5,CREBZF,CARD16,DDI1,IZUMO1R,ME3,SNORA70E,SNORD5,ELMOD1,MMP12,PANX1,TRIM53AP,TRIM49C,LOC643733,TMEM126B,CCDC67,MIR1261,MIR4490,TENM4,CASP1P2,KDM4D,CCDC83,LOC643923,SLC36A4,VSTM5,MIR4693,TYR,CASP4,SNORA8,CWC15,CEP126,CASP1,TRIM64B,PIWIL4,JRKL-AS1,MIR708,SESN3,CNTN5,JRKL,PRCP,CCDC81,MED17,MTMR2,LOC100506368,MIR4300HG,FZD4,RAB30-AS1,WTAPP1,DLG2,MIR3920,MMP8,SLN,OR7E2P,CCDC90B,SCARNA9,ARHGAP42,MIR1304,RAB30,CCDC82,YAP1,MIR5579,SNORA25,FUT4,ENDOD1,FAM76B,NARS2,MIR7641-1,GRIA4,CTSC,TMEM126A,AASDHPPT,C11orf97,DDIAS,HEPHL1,SNORA18,KDM4E,LOC101928535,MRE11A,CASP12,TRIM49D2,ANKRD42,MIR6755,SNORA32,SNORD6,KBTBD3,SLC35F2,LOC100129203,FOLH1B,DYNC2H1,MMP13,MMP27,TRIM49D1,LOC102723895,MMP1,SYTL2,C11orf73,BIRC3,MMP20,CARD17,CHORDC1,BIRC2,PICALM,LOC100128386,MSANTD4,SMCO4,MMP10,RAB39A,CEP295,FAT3,MMP3,TMEM123,SRSF8,PGR,PDGFD,MMP7,NAALAD2,LOC101054525,DCUN1D5,CASP5,ALKBH8,TRPC6,TMEM133,C11orf70,LOC101929295,SNORA40,AMOTL1,GRM5-AS1,FAM181B,CCDC89,ANGPTL5,MAML2,MIR4300,SNORA1,EED	3	gain
1373	CGTE_18	11	123600383	124440854	11q24.2-q24.1	OR10S1,OR6M1,OR8D2,OR8B12,OR6X1,OR8D4,OR10G8,OR8G2,TMEM225,OR10G9,OR10G4,OR8B3,OR8D1,OR10G7,OR8B4,OR8B8,OR6T1,OR4D5,ZNF202,OR8B2,OR8G1,VWA5A,OR8G5,OR8A1	4	gain
1374	CGTE_18	12	208094	6562548	12p13.31-p13.33	LINC00942,FKBP4,GALNT8,CACNA1C-IT3,CCDC77,C12orf4,LINC00940,KCNA6,LOC101929549,NTF3,CCND2-AS1,SLC6A13,CD9,FBXL14,LOC100049716,RAD51AP1,CRACR2A,LOC101929584,THCAT155,DCP1B,CD27,CCND2,TAPBPL,TULP3,CACNA1C-AS4,B4GALNT3,WNK1,CACNA1C-IT2,LOC101929384,FGF6,KDM5A,LRTM2,ERC1,NINJ2,RHNO1,ADIPOR2,PARP11,IQSEC3,NRIP2,CACNA1C-AS1,CACNA2D4,SCNN1A,LOC100507424,TSPAN9,TIGAR,MIR3649,FGF23,NDUFA9,TEAD4,ANO2,PRMT8,LOC102723544,LTBR,CD27-AS1,DYRK4,PLEKHG6,KCNA1,TNFRSF1A,CACNA1C-AS2,AKAP3,LOC574538,SLC6A12,VWF,ITFG2,LOC283440,RAD52,WNT5B,KCNA5,CACNA1C,FOXM1	3	gain
1375	CGTE_18	12	7342500	7655405	12p13.31	PEX5,CD163L1,ACSM4,CD163	4	gain

1376	CGTE_18	12	9067052	22688280	12p13.2-p12.3	CLECL1,TAS2R30,HIST4H4,GABARAPL1,SLCO1B1,SKP1P2,PRH1-TAS2R14,CLEC12B,RERGL,LOC101928162,CDKN1B,ERP27,LOC102724020,TAS2R50,MGP,LOC374443,ARHGDIB,CLEC12A,SLCO1C1,TAS2R43,PRR4,TAS2R19,CLEC9A,HTR7P1,APOLD1,GPRC5A,CD69,SLC15A5,TAS2R13,MIR7641-2,LOC101928030,RPL13AP20,KLRG1,BCL2L14,PRH1-PRR4,FAM234B,A2M,MIR1244-3,KLRC4-KLRK1,RERG,GUCY2C,ABCC9,SMCO3,PZP,MIR1244-4,KLRF1,SLCO1A2,KLRF2,PIK3C2G,KLRAP1,SLCO1B3,MIR1244-1,TAS2R9,GPR19,LOC101928100,PLBD1-AS1,LDHB,LINC00987,LOC100506159,KLRC4,TAS2R42,DDX47,PLCZ1,M6PR,DUSP16,PDE3A,KLRC2,EPS8,ETV6,LINC00612,LMO3,OLR1,H2AFJ,CLEC1B,MIR3974,ST8SIA1,PTPRO,PRB1,CLEC2D,LOC100506393,DERA,YBX3,TAS2R10,TAS2R31,CMAS,MIR1244-2,PRH2,EMP1,PLEKHA5,PRH1,LOC642846,PLBD1,ATF7IP,WBP11,SMIM10L1,TAS2R7,A2MP1,KLRC3,A2M-AS1,TAS2R20,STYK1,IAPP,STRAP,PRB4,RERG-AS1,PHC1,SPX,TAS2R46,LINC01489,TAS2R14,TAS2R8,CREBL2,MAGOHB,GSG1,LINC01559,PYROXD1,GPRC5D,BORCS5,CLEC7A,KCNJ8,KLRB1,LINC01252,MIR614,C2CD5,CLEC2A,CAPZA3,GYS2,LRP6,LOC100506314,PRB3,MIR613,CLEC1A,ART4,KLRC1,KLRK1,MGST1,CLEC2B,C12orf60,GRIN2B,SLCO1B7,HEBP1,LOC101930452,MANSC1,TMEM52B,PRB2,LOH12CR2,GOLT1B,AEBP2,RECQL,PDE6H,DDX12P,KLRD1	3	gain
1377	CGTE_18	12	22696903	24736959	12p12.1	SOX5,LINC00477,C2CD5,MIR920,ETNK1,LOC101928471,LOC101928441	5	gain
1378	CGTE_18	12	24970877	47758052	12p11.1-p11.23	ARID2,C12orf71,ERGIC2,TSPAN11,BICD1,FLJ13224,MUC19,ITPR2,PCED1B,FGFR1OP2,TMEM117,RASSF8,AMN1,PPHLN1,SLC2A13,SLC38A2,MRPS35,ASUN,LOC100288798,FAM60A,CNTN1,RACGAP1P,PLEKHA8P1,LOC101927058,PUS7L,ANO6,RNY5,MANSC4,C12orf40,TM7SF3,NELL2,LOC100506606,H3F3C,OVCH1,DDX11,IRAK4,ARNTL2,DENND5B,MIR7851,SCAF11,ABCD2,LINC00941,CASC1,CAPRIN2,DENND5B-AS1,PCED1B-AS1,FGD4,DDX11-AS1,LINC00938,SYT10,GXYLT1,TMTC1,LRMP,CPNE8,PDZRN4,ALG10,LMNTD1,SMCO2,YAF2,PRICKLE1,REP15,MED21,LRRK2,MIR4302,IPO8,CCDC91,SLC38A4,ZCRB1,DNM1L,PKP2,KIF21A,MIR4698,TWF1,MIR4494,BCAT1,KRAS,RASSF8-AS1,LYRM5,PPFIBP1,METTL20,STK38L,ALG10B,SLC38A1,FAR2,KIAA1551,PTHLH,ARNTL2-AS1,SSPN,DBX2,BHLHE41,YARS2,AMIGO2,C12orf77,ADAMTS20,KLHL42,OVCH1-AS1	3	gain

1379	CGTE_18	12	50560888	55042237	12q13.2-q13.13	MFSD5,ATF1,LOC100652999,FLJ12825,ESPL1,LACRT,KRT7,KRT3,C12orf80,HOTAIR,PRR13,GLYCAM1,METTL7A,SCN8A,HNRNPA1P10,TARBP2,MIR3198-2,LOC102724050,HOXC8,ATF7,KRT6A,MIR196A2,MIR148B,LOC400043,HOXC13,KRT6B,KRT85,KRT73-AS1,SPRYD3,SP1,SP7,TMPRSS12,PCBP2,KRT8,AMHR2,TNS2,KRT74,RARG,SMUG1,SOAT2,HOXC10,KRT82,KRT5,KRT77,ATP5G2,LOC100240734,KRT75,DIP2B,AAAS,BIN2,KRT86,NR4A1,LARP4,ATG101,KRT18,LOC283335,KRT79,ACVRL1,KRT73,EIF4B,C12orf10,ZNF385A,MIR1293,ITGA5,KRT80,SMAGP,FAM186A,TFCP2,CBX5,DAZAP2,IGFBP6,CSRNP2,KRT1,PDE1B,HOXC9,KRT84,KRT81,NPFF,ACVR1B,KRT72,LINC00592,ANKRD33,LIMA1,OR7E47P,PCBP2-OT1,CSAD,PPP1R1A,KRT6C,SNORD133,HIGD1C,LOC100240735,ZNF740,PFDN5,MIR615,KRT78,HOXC13-AS,KRT71,KRT2,HOXC11,COPZ1,SLC11A2,KRT4,HOXC-AS1,LETMD1,DCD,GPR84,HNRNPA1,GALNT6,GRASP,SLC4A8,MIR6757,NFE2,POU6F1,HOXC12,KRT83,HOXC5,CELA1,CISTR,MAP3K12,HOXC6,HOXC4,CALCOCO1,GTSF1,HOXC-AS3,KRT76,NCKAP1L,CERS5,ITGB7,FIGNL2,HOXC-AS2	3	gain
1380	CGTE_18	12	55233010	56076242	12q13.2	OR6C76,OR6C1,OR9K2,TESPA1,NEUROD4,OR10A7,OR2AP1,METTL7B,OR6C3,OR6C4,OR6C74,OR6C65,OR10P1,OR6C2,MUCL1,OR6C6,OR6C70,OR6C75,OR6C68	4	gain

1381	CGTE_18	12	59199716	122186424	12q15-q21.31	MIR548AL,TDG,HSP90B1,ATXN2,FAM222A,MIR7106,LOC101928731,TMEM5-AS1,PGAM1P5,LOC101929058,LOC101928449,STAB2,RASAL1,BRAP,CFAP54,MGAT4C,DPY19L2,TPH2,SNORA70G,LINC00936,SELPLG,MYO1H,MIR618,LEMD3,HRK,ANKS1B,ATP2B1,UNC119B,ALX1,NR2C1,DEPDC4,CHPT1,SLC41A2,PMCH,NUP37,LINC00615,MIR1827,P2RX7,SRGAP1,SIRT4,MIR3913-1,LOC100507195,MIR548Z,MIR6762,DUSP6,MIR617,CCDC64,PRDM4,GCN1,HELB,GATC,SRSF9,CRY1,RPLP0,LINC01490,VPS29,IFT81,PLA2G1B,NFYB,VSIG10,TMEM233,GLIPR1L1,LINC01479,TMTC3,TMTC2,LINC01619,ARL1,LIN7A,NTS,RPL6,MIR6760,ANO4,XLOC_009911,LINC01234,DYRK2,LINC01486,ACADS,LUM,C12orf73,NR1H4,FBXO21,MIR1251,GLIPR1L2,SYCP3,RBM19,NT5DC3,MIR620,CSRP2,KRT19P2,HNF1A-AS1,TBK1,RMST,RPH3A,TSPAN8,PAWR,TBX5,MIR4699,RAB35,USP30-AS1,FOXN4,MYBPC1,PXN,NAP1L1,PTPN11,C12orf50,TRHDE-AS1,BBS10,RFX4,TXNRD1,RASSF3,RNF10,TRIAP1,THAP2,SUDS3,MIR7107,PTPRR,SLC9A7P1,C12orf56,TCTN1,OAS2,SLC5A8,PLXNC1,HNF1A,MKRN9P,LRRIQ1,WSB2,RNFT2,MIR4497,HCFC2,CEP83-AS1,MIR548C,MIR1279,SNRPF,MIR6861,USP15,E2F7,NUDT4P2,TBX3,LOC100507065,MAP1LC3B2,ACAD10,CCDC53,OSBPL8,GOLGA2P5,CLLU1OS,SLC25A3,PPTC7,MIR7641-2,MYF6,PXN-AS1,CCDC60,LOC102724663,LOC102724421,NUAK1,LOC100507377,NOS1,MIR4498,HECTD4,NUP107,SART3,C12orf45,LOC101929084,CAMKK2,AMDHD1,TPCN1,NUDT4,C12orf76,ANKRD13A,RFC5,EEA1,PPM1H,ALKBH2,CPM,TRHDE,FBXW8,PEBP1,MIR4700,TMCC3,IFNG,MDM1,CPSF6,SSH1,CCER1,MMAB,OTOGL,MSI1,NTN4,C12orf29,RPSAP52,CEP290,TRPV4,GNPTAB,SLC6A15,LHX5,LINC01481,MIR4303,VEZT,MSRB3,METTL25,MTERF2,POP5,MIR7844,WIF1,MLEC,PTPRB,SH2B3,SDSL,ANAPC7,MIR3922,FAM109A,NDUFA12,RASSF9,KRR1,LRRC10,CAPS2,RAB21,SLC16A7,TTC41P,TMEM263,FICD,EID3,LOC101929162,ZFC3H1,DYNLL1,GLT8D2,MIR1178,MIR1252,LOC100129940,POC1B-GALNT4,OAS3,GALNT4,LLPH-AS1,DTX1,UHRF1BP1L,CCDC63,IQCD,UNG,MRS2P2,C12orf49,LINC01465,SYT1,KCNMB4,LYZ,UTP20,TRAFD1,RAD9B,LOC414300,XPOT,ANAPC5,FLJ41278,FRS2,HMGA2,ERP29,MIR4495,WSCD2,HAL,PPP1CC,ELK3,GLIPR1,SNORA53,CHST11,LOC102724933,LOC728739,LOC100507175,MVK,RNF34,LOC643711,LINC00934,PRKAB1,SDS,TMBIM4,LINC01405,IRAK3,NAA25,MIR619,SPPL3,BTBD11,NAV3,CDK17,MIR6125,MYF5,CLLU1,COX6A1,TMEM119,CAND1,C12orf75,RIC8B,RAB3IP,CRADD,KCTD10,MIR4472-2,CUX2,MIR6502,DDX54,MIR1302-1,YEATS4,FAM216A,POC1B,ADAM1A,MIR135A2,LOC100130075,LOC643339,CASC18,ASCL4,PHLDA1,TCHP,MIR3913-2,LOC728084,TESC-AS1,MYL2,SOCS2-AS1,KERA,TMPO,CNOT2,GNS,UBE2N,LOC101928002,LOC100506869,RAP1B,LTA4H,OAS1,ACACB,LOC100505978,USP30,RITA1,PTPRQ,MAPKAPK5,C12orf66,MYRFL,SVOP,CMKLR1,MIR4496,TMEM19,USP44,HSPB8,TMEM120B,IGF1,FAM19A2,MIR3652,ACTR6,NUDT4P1,CIT,IL22,P2RX4,PWP1,PLEKHG7,CCT2,ACSS3,CEP83,LINC00173,KCCAT198,PPP1R12A,LOC643770,NRAV,MIR3657,TCP11L2,GPN3,CCDC59,CCDC38,TMPO-AS1,KIAA1033,DCN,LOC101928937,EPYC,KSR2,FAM71C,GIT2,MIRLET7I,DRAM1,SLC17A8,ORAI1,C12orf43,LGR5,LRIG3,GAS2L3,BTG1,TESC,C12orf74,LOC100507250,NEDD1,SPIC,TBC1D15,C12orf42,GLTP,TAOK3,MIR5692B,LLPH,MON2,LINC01498,KDM2B,TMEM116,LOC101927901,SRRM4,PAH,LOC100287944,OASL,SCYL2,FGD6,POLR3B,TSPAN19,PARPBP,PPFIA2,	3	gain
1382	CGTE_18	13	19600474	20539498	13q12.11	MPHOSPH8,ANKRD26P3,ZMYM5,TPTE2,TUBA3C,PSPC1,LINC00350,LOC101928697,LINC00421,ZMYM2	4	gain

1383	CGTE_18	13	53419777	73302626	13q21.2-q21.31	MIR1297,LINC01075,PRR20B,MZT1,LINC00358,LINC00376,PRR20A,PRR20C,LINC00374,LINC00550,LINC00348,PCDH9-AS2,PCDH20,PCDH9,LOC102723968,ATXN8OS,PRR20D,LINC00364,DACH1,LINC01065,OLFM4,LINC00378,MIR3169,MIR5007,PCDH8,LINC00434,DIAPH3-AS1,PCDH9-AS3,LINC00395,LINC00458,KLHL1,LINC00558,PCDH17,DIAPH3,TDRD3,OR7E156P,LOC101926897,LINC00448,LINC01052,MIR548X2,PRR20E,DIAPH3-AS2,PCDH9-AS4,MIR4704,LINC00383,BORA	3	gain
1384	CGTE_18	13	113337502	115091802	13q34	MIR8075,RASA3,PROZ,GRTP1-AS1,GAS6-AS2,ATP11AUN,MCF2L-AS1,GAS6,CUL4A,ATP4B,ADPRHL1,LAMP1,TFDP1,ATP11A,F10,LINC00565,MCF2L,PCID2,F7,MIR548AR,CDC16,GAS6-AS1,LOC101928841,UPF3A,MIR4502,ATP11A-AS1,GRTP1,DCUN1D2,TMEM255B,F10-AS1,LINC00452,GRK1,TMCO3,CHAMP1,LINC00552	1	loss
1385	CGTE_18	14	20201784	20665925	14q11.2	OR11G2,OR4K15,OR4K2,OR4N5,OR4Q3,OR4K17,OR4K5,OR4M1,OR4K13,OR4L1,OR4K14,OR4K1,OR4N2	7	gain
1386	CGTE_18	14	21979045	22749637	14q11.2	OR4E2,OR10G3,METTL3,OR10G2,OR4E1,SALL2	4	gain
1387	CGTE_18	14	24906371	29237816	14q12	GZMB,FOXG1,GZMH,LOC101927062,CTSG,CMA1,STXBP6,LINC00645,MIR4307,FOXG1-AS1,KHNYN,MIR4307HG,SDR39U1,MIR3171,NOVA1,LOC102724890,LOC101927045	4	gain
1388	CGTE_18	14	29237820	45716484	14q21.2-q13.2	SCFD1,TRAPPC6B,LOC101927178,SPTSSA,PNN,FAM177A1,LOC100288846,FOXA1,RNU6-2,AKAP6,KIAA0391,PRPF39,MIA2,PRKD1,BRMS1L,SNX6,TTC6,EAPP,SRP54,FKBP3,EGLN3,NKX2-1,LRFN5,LOC101927418,PTCSC3,NKX2-1-AS1,SFTA3,MIR548AI,GPR33,PSMA6,LOC101927124,MIR4503,MIR624,SSTR1,AP4S1,PPP2R3C,CTAGE5,G2E3,LINC00639,IGBP1P1,MIPOL1,C14orf28,NFKBIA,SNORA89,KLHL28,PAX9,INSM2,CFL2,MBIP,CLEC14A,MIS18BP1,DTD2,BAZ1A,NUBPL,LINC01551,LINC00609,FBXO33,FOXG1,NKX2-8,ARHGAP5-AS1,SEC23A,LOC644919,STRN3,SLC25A21,SLC25A21-AS1,NPAS3,FSCB,SNORD127,FANCM,LOC100506071,COCH,HEATR5A,HECTD1,FAM179B,RALGAPA1,ARHGAP5,GEMIN2,RALGAPA1P	3	gain
1389	CGTE_18	14	47120321	50065932	14q21.2-q21.3	MDGA2,RPL10L,RPS29,MIR548Y,LRR1,LINC00648	5	gain

1390	CGTE_18	14	50319306	99880405	14q21.3-q23.1	ACOT6,FRMD6-AS2,PGF,LINC01146,SIX4,LINC01588,NIN,ARF6,VRTN,NEK9,CALM1,ARG2,LOC730202,MIR548AZ,LINC01220,C14orf177,FLJ22447,MIR4506,CNIH1,ABHD12B,GCH1,SIX1,MIR5580,FOS,NPC2,FRMD6-AS1,NOXRED1,PLEKHH1,LIN52,CCDC177,FOXN3,PSMA3,FNTB,LINC00642,ATP5S,SERPINA4,TCL1B,C14orf37,CCDC88C,ZBTB25,TDP1,SNAPC1,MIR3173,RGS6,PAPLN,LOC102724190,VTI1B,ABCD4,PPP4R3A,C14orf159,ADCK1,AHSA1,SNORA79,TEX21P,KTN1-AS1,LRRC74A,DACT1,DCAF4,GSC,BDKRB1,ZC3H14,LOC101928791,SRSF5,SIX6,LOC101929241,IFI27L1,DNAL1,CIPC,PRKCH,ACYP1,DICER1,TTC8,ALDH6A1,AP5M1,IFI27L2,TUNAR,ISCA2,ZC2HC1C,NGB,GSKIP,LGMN,LOC100129345,RPS6KA5,SYNE3,JDP2,VRK1,ISM2,C14orf178,FUT8,KCNH5,C14orf169,GNPNAT1,NRXN3,EIF2S1,LOC101929080,TTC7B,GPR137C,UNC79,LOC101927780,PRIMA1,LOC102723604,BATF,TMED8,DCAF5,PSMC6,HEATR4,GALNT16,ESR2,SYNJ2BP,PIGH,SMOC1,CGRRF1,FAM181A-AS1,IRF2BPL,C14orf1,SLC39A9,MIR548H1,TXNDC16,TRIP11,PCNX,LINC00520,ZFP36L1,SPATA7,ITPK1-AS1,PELI2,SLC8A3,C14orf39,PTGER2,TMEM30B,GTF2A1,AKAP5,L2HGDH,ENTPD5,FAM181A,HIF1A,PYGL,PPP4R4,MIR4504,PROX2,KIAA0586,RHOJ,IFT43,LOC101927620,SLC38A6,SNHG10,SOCS4,MIR1260A,AK7,FRMD6,EFCAB11,NRDE2,ATXN3,SYNE2,EIF2B2,SAMD15,FOXN3-AS1,DLST,TSHR,FCF1,CHURC1,OTUB2,SETD3,LINC01599,LOC101928075,VIPAS39,ZDHHC22,SERPINA13P,LOC101928767,PLEKHD1,CDKL1,BBOF1,LOC100506700,OTX2-AS1,CATSPERB,SERPINA1,FLVCR2,SYNDIG1L,VASH1,COQ6,PNMA1,TC2N,ZBTB1,GPX2,SLC35F4,TTLL5,COX16,TMX1,MLH3,RPL13AP3,ITPK1,MAX,NID2,PPM1A,MAP3K9,LINC01269,ATL1,MIR4709,LOC283575,LOC100506321,MED6,ANGEL1,JKAMP,LOC283585,TCL6,GOLGA5,EXOC5,KTN1,RIN3,SYNJ2BP-COX16,FERMT2,GLRX5,HIF1A-AS2,LOC100128233,ACOT1,FAM161B,DAAM1,DICER1-AS1,SERPINA12,ACOT4,SERPINA3,GSTZ1,LINC00911,FBLN5,EML5,FBXO34,GPHN,LGALS3,SERPINA11,ERH,SGPP1,ADAM21P1,LINC00618,SERPINA2,ZFYVE1,MTHFD1,MNAT1,RBM25,PTPN21,SCARNA13,PCNXL4,RPS6KL1,ARID4A,TMEM229B,LOC145474,SNW1,LINC00640,WDR89,SNORA11B,GPR135,STON2,WDHD1,FUT8-AS1,VCPKMT,ACTR10,LOC100506603,LOC100289511,CHGA,LINC01550,SAV1,SERPINA10,PAPOLA,GNG2,C14orf132,C14orf142,LOC101928909,ESRRB,RTN1,RAD51B,GPATCH2L,MOAP1,STYX,TRIM9,HIF1A-AS1,MIR6076,SUSD6,UBR7,LINC00643,PTGDR,NAA30,ZNF410,GALC,BMP4,TCL1A,C14orf166,LTBP2,TMEM63C,FOXN3-AS2,TMEM251,LOC102723809,ACTN1-AS1,COX8C,ATG14,DHRS7,FAM71D,ELMSAN1,PPP2R5E,SAMD4A,BDKRB2,ACTN1,L3HYPDH,GPR65,FLRT2,PSEN1,NDUFB1,CHURC1-FNTB,ATP6V1D,ADAM20P1,CLMN,DIO2,GMFB,IFI27,NUMB,C14orf105,ADAM21,CEP128,POMT2,SPTB,DPF3,DDX24,RAB15,SLIRP,MIR7641-2,PSMC1,KCNK10,ZFYVE26,DIO2-AS1,LINC01467,AREL1,LOC102724153,DLGAP5,MAPK1IP1L,TOMM20L,NEMF,LINC00521,SYT16,PLEK2,SLC10A1,TGFB3,CCDC175,YLPM1,LINC00644,ASB2,PSMA3-AS1,KCNK13,SERPINA6,MPP5,SERPINA5,RDH11,BTBD7,TIMM9,VSX2,TRMT5,ACOT2,BCL11B,ALKBH1,TTC9,TMED10,SNORD56B,CPSF2,HSPA2,CDKN3,MIR7843,PTGR2,MIR4308,G	3	gain
1391	CGTE_18	15	20170004	22833683	15q11.2-q11.1	REREP3,MIR4509-3,MIR3118-3,MIR4509-2,CHEK2P2,GOLGA6L1,GOLGA6L22,POTEB2,OR4N4,POTEB3,LOC646214,TUBGCP5,MIR3118-4,GOLGA8CP,NF1P2,POTEB,MIR4509-1,MIR3118-2,CXADRP2,MIR5701-1,HERC2P3,LINC01193,OR4M2,MIR5701-2,LOC727924,LOC101927079,GOLGA8DP,OR4N3P,GOLGA6L6,NBEAP1,MIR5701-3,MIR1268A	3	gain

1392	CGTE_18	15	23086229	25277865	15q11.2	PWRN1,MIR4508,PWRN4,SNRPN,WHAMMP3,PWARSN,PWRN3,MAGEL2,GOLGA8EP,SNURF,SNORD108,GOLGA8IP,HERC2P7,GOLGA6L22,SNORD107,NDN,MKRN3,PWAR5,SNORD64,NPAP1,HERC2P2,LOC283683,GOLGA6L2,PWRN2,GOLGA8S,NIPA1	3	gain
1393	CGTE_18	15	26107784	40293533	15q13.2-q12	DKFZP434L187,NSMCE3,FAM98B,MIR4509-2,HERC2,SLC12A6,PGBD4,LOC100128714,RYR3,C15orf41,MIR4509-3,GOLGA8B,GOLGA8F,C15orf54,MIR1233-2,LPCAT4,ATP10A,MIR4510,ULK4P3,CHRM5,AQR,PDCD6IPP2,FAM189A1,LOC100288637,MIR1233-1,MIR3942,NOP10,NUTM1,SCG5,RASGRP1,AVEN,LOC101928174,LOC101928227,ULK4P2,GOLGA8T,GJD2,GOLGA8M,KATNBL1,OTUD7A,LOC145845,LOC283710,MIR4509-1,CHRFAM7A,WHAMMP1,MEIS2,TMCO5A,ANP32AP1,GOLGA8O,KLF13,FAN1,ULK4P1,WHAMMP2,GOLGA8R,MIR211,ACTC1,GOLGA8H,C15orf53,GOLGA8K,GOLGA8G,GOLGA8J,LOC101928134,LINC00929,CSNK1A1P1,MTMR10,ZNF770,GPR176,HERC2P9,OCA2,EIF2AK4,GOLGA8N,GOLGA6L7P,HERC2P10,SPRED1,ARHGAP11B,LOC100996255,APBA2,LOC100131315,TRPM1,TJP1,ARHGAP11A,MIR8063,GABRG3-AS1,DPH6-AS1,EMC7,EMC4,GABRG3,GABRA5,GABRB3,THBS1,GOLGA8A,FSIP1,TMCO5B,DPH6,FMN1,CHRNA7,GREM1,LOC100289656	3	gain
1394	CGTE_18	15	48051987	50475068	15q21.1-q21.2	FBN1,MYEF2,FGF7,ATP8B4,CTXN2,SHC4,SECISBP2L,LINC01491,CEP152,SEMA6D,SLC12A1,SLC27A2,DUT,DTWD1,FAM227B,MIR4716,EID1,COPS2,GALK2,NDUFAF4P1,SLC24A5	3	gain
1395	CGTE_18	15	52611297	60720911	15q22.1-q21.3	LIPC,GCOM1,RSL24D1,HSP90AB4P,ICE2,LDHAL6B,RAB27A,UNC13C,TCF12,PYGO1,ADAM10,MNS1,LINC01413,ALDH1A2,CGNL1,GCNT3,FOXB1,DYX1C1,WDR72,C15orf65,LOC145783,FAM81A,ARPP19,CCNB2,POLR2M,MYO1E,MIR628,DYX1C1-CCPG1,MYZAP,NEDD4,ZNF280D,SLTM,TEX9,LOC101928694,PRTG,PIGBOS1,MIR2116,PIGB,CCPG1,FAM214A,AQP9,BNIP2,LINC00926,ONECUT1,GTF2A2,RFX7,ANXA2,RNF111,MYO5A,FAM63B	3	gain
1396	CGTE_18	15	60740134	72252424	15q22.33-q22.2	TRIP4,VPS13C,PARP16,SKOR1,THSD4,RPL4,NR2E3,MIR422A,FBXL22,VWA9,HACD3,TPM1,ZNF609,THAP10,SALRNA3,MIR190A,MEGF11,KIAA0101,RAB8B,SNAPC5,ANP32A-IT1,LCTL,DENND4A,CA12,FEM1B,SLC24A1,MIR629,PIF1,MGC15885,SALRNA2,EWSAT1,SPESP1,RORA,MTFMT,CALML4,TLN2,MIR8067,SCARNA14,CLN6,IQCH-AS1,TIPIN,LOC145694,LRRC49,TLE3,MYO9A,SNORD18B,LOC101928988,UBAP1L,PAQR5,ZWILCH,LINC00593,NOX5,MIR6085,KIF23,HERC1,PLEKHO2,LINC01169,SNORD16,DAPK2,UACA,THSD4-AS2,RPLP1,PPIB,IQCH,OAZ2,MIR4312,SMAD6,RBPMS2,DPP8,AAGAB,KBTBD13,MIR1272,C15orf61,RORA-AS2,RPS27L,PIAS1,SNX22,LOC102723344,USP3,CSNK1G1,MIR4512,ICE2,RORA-AS1,FAM96A,PDCD7,C2CD4A,ANKDD1A,MIR548H4,RASL12,GLCE,RNU6-2,IGDCC3,PCAT29,MAP2K5,THSD4-AS1,SNX1,C2CD4B,MIR4511,RAB11A,SLC51B,IGDCC4,ANP32A,CLPX,CORO2B,SPG21,LOC101929076,SNORD18C,SMAD3,APH1B,ITGA11,LARP6,LACTB,DIS3L,MAP2K1,CT62,DRAIC,MIR4311,SNORD18A,USP3-AS1,CILP	3	gain

1397	CGTE_18	15	91560930	102389808	15q26.2-q26.1	LUNAR1,LINC01581,MIR4714,PCSK6,CERS3-AS1,SV2B,ST8SIA2,LRRK1,VPS33B,ASB7,LRRC28,SLCO3A1,FAM169B,LINC01197,LINS1,OR4F6,VIMP,SNRPA1,C15orf32,LOC440311,MEF2A,OR4F15,ASB9P1,CHSY1,LINC00930,ALDH1A3,MIR1469,CRAT37,CERS3,PGPEP1L,SPATA8-AS1,HSP90B2P,LOC101927310,ADAMTS17,LOC100507472,NR2F2-AS1,SPATA8,ARRDC4,TTC23,OR4F13P,TM2D3,LINC00923,IGF1R,LOC101927153,NR2F2,DNM1P46,LINC01582,MIR3175,RGMA,CHD2,LOC101927286,TARSL2,PCSK6-AS1,LINC01580,LOC104613533,SYNM,LINC00924,PRKXP1,LOC101926911,LYSMD4,FAM174B,MCTP2,SPATA41,LINC01578,IRAIN	3	gain

1398	CGTE_18	16	2809750	34257343	16p13.12-p12.3	SMG1P2,TBC1D10B,SEPT12,OR1F1,GGA2,YBX3P1,ZNF843,ROGDI,CDIPT,PRM3,ZP2,NPIPB4,PKD1P1,MIR6769A,MMP25-AS1,MIR3179-3,C16orf82,NOMO2,RBFOX1,TP53TG3C,CCDC189,ZNF771,PRSS36,MIR548D2,DCTN5,MIR3680-2,TMEM219,EEF2KMT,LOC554206,AQP8,CLUHP3,MYH11,TBX6,DEXI,CDIPT-AS1,TGFB1I1,MIR365A,MIR4517,SLX1A-SULT1A3,SRCAP,ZNF597,ATF7IP2,MAPK3,PRKCB,ZNF646,NUDT16L1,VASN,ZNF768,RSL1D1,ZNF785,SEC14L5,MIR6511A1,POLR3E,ABCC6P1,CDR2,ACSM2A,HERC2P4,GDE1,GSPT1,LOC101927348,COQ7,THUMPD1,SNX29P1,XYLT1,MIR762,PYCARD,DNAH3,PAM16,SLC5A11,MMP25,GSG1L,SLX4,FUS,ZNF764,ATP2A1-AS1,ZNF668,UBFD1,LCMT1-AS2,CORO1A,FOPNL,TMEM159,PKMYT1,ARL6IP1,MGRN1,LOC388242,FBRS,LOC606724,LINC00514,ITPRIPL2,MTRNR2L4,SCNN1G,XPO6,CLUAP1,SULT1A2,SULT1A1,HS3ST2,CLEC19A,ORAI3,DNAJA3,ZNF500,VWA3A,LCMT1-AS1,NOMO3,HCFC1R1,CLN3,COG7,BOLA2,LOC105447648,HS3ST4,SCNN1B,NPIPA1,MIR6862-2,GPRC5B,HMOX2,MIR6770-1,TNFRSF17,ITGAL,PMM2,RNF40,LINC01195,ACSM1,APOBR,SGF29,LOC102724927,ARHGAP17,MIR548X,CPPED1,MIR3680-1,ZSCAN32,ALG1,LOC613038,SNORA30,KIAA0556,BFAR,RABEP2,PAQR4,ZNF688,HSD3B7,LOC613037,BOLA2B,LOC81691,LOC100288162,MIR3180-2,LITAF,C16orf58,NPIPB6,SHISA9,TUFM,SLC5A2,LAT,MVP,MIR6862-1,NDE1,ACSM5,ADCY9,C16orf62,MIR6511A2,PHKG2,KREMEN2,C16orf45,PDXDC1,C16orf92,MIR4518,SETD1A,PLA2G10,C16orf71,ZKSCAN2,GPR139,PRSS33,MIR193BHG,ACSM3,ZNF213-AS1,ZC3H7A,MPV17L,CCP110,AHSP,RMI2,NSMCE1,TIGD7,FBXL19,DCTPP1,MIR3179-2,PRSS8,CHP2,FAM57B,LOC653786,MIR3670-2,EMP2,ANKS3,ZNF75A,STX1B,IL21R-AS1,TMC7,PRSS22,RRN3P3,KAT8,TP53TG3B,TFAP4,CACNG3,SMG1P1,ITGAM,TRIM72,CLEC16A,YPEL3,NPIPA5,LOC101927814,LYRM1,SOCS1,TMEM265,PAGR1,SPNS1,ABCC1,ERI2,IQCK,SMG1,SLX1A,THOC6,ITGAD,GLIS2,LINC00921,PRSS41,MYLPF,TEKT5,NDUFAB1,ZG16B,ARMC5,ACSM2B,C16orf96,TMC5,MIR4519,LOC100190986,NFATC2IP,PRSS21,MIR3670-4,SRRM2,SMG1P3,ABCC6P2,FLYWCH1,OR2C1,TMEM186,RRN3P1,GTF3C1,MIR762HG,MIR6511B1,GRIN2A,ALDOA,UQCRC2,EEF2K,RRN3,SYT17,NOMO1,ZNF267,NTAN1,SLX1B-SULT1A4,NPIPA8,MIR6770-2,IL32,USP7,PRRT2,OR1F2P,NPIPA7,TRAP1,CD2BP2,MIR4718,LINC01567,C16orf89,IGSF6,TNP2,CLDN6,ZNF747,CRYM-AS1,LOC101929613,ASPHD1,FBXL19-AS1,CASP16P,SNX29,MIR3179-4,KIAA0430,ZNF629,NPIPB8,PRM1,DCUN1D3,TP53TG3,MIR3179-1,LOC100128770,BCAR4,NUBP1,CIITA,EARS2,SRL,PDILT,CORO7,CREBBP,OTOA,MIR3180-3,UMOD,QPRT,TNRC6A,SNN,PYDC1,SEPHS2,CTF1,MIR193B,RBBP6,PRM2,PALB2,PDZD9,IL4R,TNFRSF12A,KNOP1,LOC101927311,NAGPA,TP53TG3D,LCMT1,TXNDC11,SEPT1,ZNF48,ANKS4B,STX4,LINC01569,ZG16,METTL9,C16orf54,NMRAL1,C16orf72,MIR3180-4,MIR6506,CD19,NUPR1,ZNF689,UBN1,CLDN9,DNASE1,ZNF205-AS1,EIF3C,C16orf52,NPIPB11,PLK1,SEZ6L2,MEFV,METTL22,NPIPA3,LINC00273,PRR14,RPS15A,DOC2A,LINC01570,CRYM,LOC101927131,GDPD3,NPIPB3,EIF3CL,PPL,NLRC3,ATXN2L,HIRIP3,KDM8,GLIS2-AS1,ZNF263,MIR484,SH2B1,NAGPA-AS1,GP2,MIR6126,CCDC64B,ABCC6,SBK1,ZNF213,LOC390705,MAZ,LOC102723385,MIR6511	3	gain
1399	CGTE_18	16	58767932	65345479	16q21	LOC729159,CDH8,APOOP5,GOT2,CDH11,LOC101927650,LINC00922,LOC101927580,MIR4426	4	gain
1400	CGTE_18	16	75446337	80673702	16q23.2-q23.1	GABARAPL2,LINC01229,CFDP1,SYCE1L,ADAT1,MAFTRR,MIR4719,MAF,WWOX,TMEM170A,MON1B,CHST5,LOC101928203,LINC01227,CHST6,CDYL2,DYNLRB2,CLEC3A,TMEM231,ADAMTS18,NUDT7,CNTNAP4,KARS,TERF2IP,VAT1L,LOC102724084	3	gain

1401	CGTE_18	17	16137230	19871817	17p11.2	SLC47A2,CCDC144A,FLJ35934,SNORD65,LOC388436,LRRC75A-AS1,SREBF1,FLII,PRPSAP2,MIR6777,USP32P2,TBC1D28,TOP3A,ALDH3A1,PEMT,ZNF287,MIR1288,KRT16P2,FBXW10,EPN2-IT1,FLCN,KRT16P1,SNORA59A,EVPLL,TRPV2,ULK2,TVP23B,MPRIP,SLC5A10,SNORA59B,LOC79999,UBB,LLGL1,GRAP,SMCR2,USP32P1,CCDC144B,MFAP4,COPS3,GRAPL,AKAP10,ZNF624,LGALS9C,SNORD49A,GID4,NT5M,MIR6778,TRIM16L,FAM106A,LRRC75A,DRC3,ATPAF2,FAM106CP,SMCR5,RAI1-AS1,MIR33B,MED9,MIEF2,PLD6,FAM83G,SLC47A1,SMCR8,DRG2,RNF112,EPN2-AS1,SNORD49B,CENPV,EPN2,TNFRSF13B,RAI1,SHMT1,RASD1,FOXO3B,ZNF286B,TOM1L2,KRT17P5,MIR1180,ALKBH5,MYO15A,ALDH3A2,PIGL,MAPK7,B9D1	1	loss
1402	CGTE_18	17	19880893	20149352	17p11.2	AKAP10,SPECC1	3	gain
1403	CGTE_18	17	38801891	39139427	17q21.2	KRT26,KRT25,KRT40,KRT39,KRT12,KRT20,KRT23,KRT222,KRT10,KRT27,TMEM99,KRT28,KRT24,SMARCE1	3	gain
1404	CGTE_18	17	39140011	39502449	17q21.2	KRTAP3-1,KRTAP1-4,KRT33A,KRTAP2-4,KRTAP4-2,KRTAP4-12,KRTAP3-3,KRTAP9-2,KRTAP2-1,KRTAP4-1,KRTAP4-3,KRTAP9-6,KRTAP2-3,KRTAP1-3,KRTAP29-1,KRTAP4-4,KRTAP4-11,KRTAP9-8,KRTAP4-7,KRTAP3-2,KRTAP9-3,KRTAP4-9,KRTAP4-8,KRTAP2-2,KRTAP4-5,KRTAP16-1,KRTAP9-4,KRTAP9-7,KRTAP4-6,KRTAP17-1,KRTAP1-1,KRTAP9-1,KRTAP1-5,KRT40,KRTAP9-9	6	gain
1405	CGTE_18	17	39502451	39622249	17q21.2	KRT38,KRT33B,KRT33A,KRT34,KRT37,KRT31,LOC100505782,KRT32	3	gain
1406	CGTE_18	17	49824824	54921501	17q21.33-q22	KIF2B,TOM1L1,COX11,NOG,STXBP4,PCTP,DGKE,ANKFN1,MMD,C17orf112,HLF,TMEM100,C17orf67,CA10	3	gain
1407	CGTE_18	17	66453395	71189502	17q24.2-q24.3	MAP2K6,KCNJ2-AS1,MIR4524A,LINC01482,SSTR2,LOC102723505,LINC01497,KCNJ16,ABCA10,PRKAR1A,FAM20A,ABCA6,SOX9-AS1,SLC39A11,LOC102723517,LOC101928205,LINC01028,LINC00511,ABCA9,LINC01483,WIPI1,ABCA8,MIR4524B,ABCA5,LINC00673,SOX9,PRO1804,LINC01152,COG1,ABCA9-AS1,KCNJ2,CASC17	3	gain
1408	CGTE_18	18	163296	1359559	18p11.32	THOC1,USP14,COLEC12,CLUL1,TYMSOS,TYMS,LINC00470,CETN1,ENOSF1,YES1,ADCYAP1	3	gain
1409	CGTE_18	18	10855298	14106021	18p11.22-p11.21	TUBB6,AFG3L2,RNMT,CHMP1B,PTPN2,CIDEA,MIR4526,CEP76,PIEZO2,C18orf61,MPPE1,LOC100996324,ZNF519,LINC01255,MC2R,LDLRAD4,MIR5190,PSMG2,MC5R,IMPA2,SLC35G4,LDLRAD4-AS1,FAM210A,ANKRD62,SEH1L,GNAL,SPIRE1,PRELID3A,CEP192,MIR7153	4	gain
1410	CGTE_18	18	18963484	20936654	18q11.2-q11.1	MIB1,MIR4741,CTAGE1,LOC101927571,GATA6,MIR133A1HG,GREB1L,ESCO1,MIR1-2,ABHD3,TMEM241,CABLES1,GATA6-AS1,RBBP8,MIR320C1,MIR133A1,SNRPD1	7	gain
1411	CGTE_18	18	20945391	21113473	18q11.2	RIOK3,C18orf8,NPC1,TMEM241	4	gain
1412	CGTE_18	18	21114423	21595061	18q11.2	TTC39C-AS1,TTC39C,ANKRD29,LAMA3,NPC1	5	gain
1413	CGTE_18	19	11849492	11878133	19p13.2	ZNF823,ZNF441	6	gain
1414	CGTE_18	19	13947517	14857833	19p13.12-p13.13	NDUFB7,C19orf57,SNORA104,MIR639,ZNF333,LOC113230,PODNL1,PTGER1,CC2D1A,PRKACA,IL27RA,ASF1B,DNAJB1,ADGRE5,PALM3,ADGRE2,ADGRE3,NANOS3,TECR,C19orf67,MIR181D,DDX39A,CLEC17A,MIR1199,PKN1,LOC100507373,DCAF15,SAMD1,RLN3,RFX1,ADGRL1,GIPC1,MIR181C	5	gain
1415	CGTE_18	19	14857842	15218412	19p13.12	OR7A5,OR7C2,SLC1A6,OR7A17,OR1I1,CCDC105,OR7A10,CASP14,SYDE1,OR7C1,ADGRE2	7	gain
1416	CGTE_18	19	15219723	15571234	19p13.12	BRD4,ILVBL,RASAL3,MIR6795,SYDE1,NOTCH3,MIR1470,AKAP8,AKAP8L,EPHX3,WIZ	4	gain

1417	CGTE_18	19	15571803	16060573	19p13.12	UCA1,CYP4F22,PGLYRP2,CYP4F12,CYP4F24P,OR10H5,CYP4F11,OR10H4,OR10H3,OR10H2,OR10H1,CYP4F3,CYP4F2,LOC102724279,CYP4F8,RASAL3	7	gain
1418	CGTE_18	19	16060707	17717215	19p13.11-p13.12	RAB8A,MRPL34,CALR3,C19orf44,COLGALT1,ABHD8,HSH2D,SLC27A1,PGLS,USHBP1,DDA1,GTPBP3,UNC13A,CPAMD8,BISPR,LINC00905,NR2F6,OCEL1,BABAM1,AP1M1,EPS15L1,CIB3,NXNL1,FAM129C,SIN3B,ANO8,PLVAP,CHERP,MED26,F2RL3,BST2,KLF2,ANKLE1,SMIM7,OR10H4,TMEM221,USE1,TPM4,NWD1,HAUS8,LINC00661,MVB12A,SLC35E1,FAM32A,MYO9B,TMEM38A	5	gain
1419	CGTE_18	19	17720739	18085140	19p13.11	SNORA68,KCNN1,MAP1S,CCDC124,JAK3,B3GNT3,SLC5A5,UNC13A,RPL18A,FCHO1,INSL3	3	gain
1420	CGTE_18	19	20150097	22951248	19p12	ZNF429,ZNF85,GOLGA2P9,LOC100996349,ZNF257,ZNF738,ZNF90,LINC00664,ZNF737,ZNF99,LOC641367,ZNF676,ZNF208,ZNF714,LINC01233,ZNF100,ZNF626,MIR1270,ZNF493,ZNF486,ZNF708,ZNF98,ZNF43,ZNF431,ZNF729,ZNF682,LOC101929124,ZNF430,ZNF492,ZNF826P	3	gain
1421	CGTE_18	19	22952020	23300098	19p12	ZNF730,ZNF728,ZNF99,LOC101929164,LOC101929144	4	gain
1422	CGTE_18	19	30433464	30936813	19q12	URI1,ZNF536	4	gain
1423	CGTE_18	19	38572128	41403382	19q13.13-q13.2	SIRT2,LGALS4,ZNF780A,RYR1,SPTBN4,C19orf47,ZNF780B,CAPN12,SPINT2,PAK4,MIR4530,PLEKHG2,PSMD8,RAB4B,LGALS7,MIR641,RASGRP4,RINL,ZFP36,C19orf54,CLC,LEUTX,LGALS16,LGALS7B,EGLN2,YIF1B,ECH1,SERTAD3,RAB4B-EGLN2,LGALS13,HIPK4,MAP3K10,CYP2G1P,DLL3,SPRED3,NUMBL,IFNL4,SERTAD1,AKT2,DPF1,ZNF546,FBXO27,PAF1,EID2,CNTD2,IFNL3,SARS2,CATSPERG,MRPS12,FBXO17,ADCK4,IFNL1,LRFN1,ITPKC,C19orf33,ACP7,SIPA1L3,SYCN,MIR6796,HNRNPL,FAM98C,SUPT5H,NCCRP1,LGALS14,EIF3K,TIMM50,MIA,MIA-RAB4B,SAMD4B,CYP2A6,MIR6719,SHKBP1,SNRPA,GMFG,PPP1R14A,RPS16,LOC100129935,BLVRB,SELV,DYRK1B,PSMC4,FBL,MAP4K1,EID2B,NFKBIB,IFNL2,LTBP4,PLD3,ACTN4,CCER2,TTC9B,PRX,GGN,CYP2A7,MED29,FCGBP,KCNK6,LGALS17A	3	gain
1424	CGTE_18	19	41403474	41601917	19q13.2	CYP2B6,CYP2A13,CYP2G1P,CYP2B7P	5	gain
1425	CGTE_18	19	41622090	43233452	19q13.2	CEACAM5,BCKDHA,CEACAM6,PSG3,LOC101930071,LINC01480,CXCL17,DEDD2,TGFB1,PRR19,MIR4323,EXOSC5,RABAC1,CNFN,POU2F2,MIR6797,CEACAM1,GRIK5,PAFAH1B3,ERICH4,TMEM91,PCAT19,GSK3A,ERF,ZNF526,CEACAM7,CD79A,MIR8077,LIPE-AS1,AXL,CYP2F1,HNRNPUL1,LOC100505622,TMEM145,B9D2,CEACAM21,ARHGEF1,LYPD4,CEACAM3,CEACAM4,CCDC97,LIPE,RPS19,CIC,ATP1A3,DMRTC2,CYP2S1,ATP5SL,B3GNT8,MEGF8,CEACAM8,ZNF574	3	gain
1426	CGTE_18	19	43244488	43570793	19q13.31-q13.2	PSG1,LOC100289650,PSG10P,PSG11,PSG8,PSG2,PSG6,PSG3,PSG7	5	gain

1427	CGTE_18	19	43575700	52772958	19q13.33-q13.32	KLK4,INAFM1,C19orf48,LOC101928517,LINC01530,NUCB1,BSPH1,PRRG2,ZNF221,CD33,PVRL2,ZNF235,BBC3,DKKL1,SIGLEC14,IRGQ,SPACA6P-AS,AP2S1,ARHGAP35,MIR8085,CEACAM20,SLC1A5,TBC1D17,ZNF114,PGLYRP1,KLK13,SMG9,POLD1,TMEM143,CGB8,ZNF155,ZNF836,NOVA2,HAS1,ZNF766,PPP2R1A,ZNF180,ZNF350,PIH1D1,MARK4,SIGLEC10,MIR4531,SHANK1,SNAR-A7,PPM1N,ZNF285,C19orf81,CLDND2,SNAR-B2,CLEC11A,AP2A1,ZNF576,NPAS1,NAPSA,GYS1,MIR642A,PPP1R37,CD37,PVR,CCDC114,BCL3,MED25,SNAR-C2,PSG2,NUP62,MIR3190,LOC100129083,IZUMO1,ELSPBP1,MIR4749,BCL2L12,IRF2BP1,SYNGR4,IGSF23,LIN7B,ZNF222,MIR4751,ZNF841,FLT3LG,KLC3,KLK2,ZNF577,SNAR-C4,CCDC61,PPP5C,STRN4,APOC4,FUT1,ZNF223,SNAR-A14,ZNF233,ZNF649-AS1,CGB5,CARD8,ERCC1,SNAR-B1,SPIB,PSG5,BAX,SNAR-D,NAPSB,APOE,CEACAM16,MEIS3,SNORD32A,CLPTM1,CEACAM19,FUZ,PLEKHA4,CGB1,SPACA6,KLK9,ZNF225,MIR6798,C19orf73,LOC284344,KLK7,ZNF283,CKM,LMTK3,DKFZp434J0226,SNAR-A5,VRK3,SNORD88A,IL4I1,MYPOP,JOSD2,MIR320E,C5AR2,PNMAL2,LIM2,MIR125A,ALDH16A1,EMP3,ZNF649,CABP5,MIR6799,SNAR-A1,SNAR-A12,SPACA4,LOC100379224,TULP2,KLK5,SCAF1,DMWD,SAE1,NAPA,SNAR-G1,FGF21,NAPA-AS1,SNAR-C1,SNAR-C3,NKPD1,TSKS,SRRM5,CCDC155,LYPD3,CTU1,GNG8,IRGC,PNMAL1,MYH14,KDELR1,GRWD1,ZNF45,ZNF575,NR1H2,SNAR-A13,SIGLEC7,PNKP,KLK15,CGB7,C19orf68,CA11,BLOC1S3,APOC2,PRR12,IGFL2,NUCB1-AS1,CGB,ZNF614,MGC45922,VSIG10L,SNAR-G2,SIGLEC8,DHX34,SNAR-A6,EML2-AS1,SNORD23,FPR1,SIGLEC12,SYT3,CADM4,SIGLEC16,LOC100505715,ERCC2,TEAD2,ADM5,LOC101928063,LRRC4B,SEC1P,PTOV1-AS1,DHDH,FAM83E,SNAR-A10,GLTSCR2,HSD17B14,ASPDH,CALM3,C5AR1,PRMT1,TRPM4,SNORD35B,GLTSCR1,CGB2,ZNF428,KCNC3,ZNF404,FPR3,SIX5,GEMIN7,MAMSTR,ETFB,ZNF473,LOC400706,CEACAM18,SNORD33,SEPW1,MIR4324,QPCTL,SLC8A2,TMEM160,BCAM,FLJ26850,APOC1P1,LHB,FKRP,C19orf84,SLC17A7,SNAR-A4,TEX101,CCDC9,PHLDB3,MIR3191,SIGLECL1,RRAS,CD3EAP,PINLYP,RTN2,FBXO46,CD177,SIGLEC6,MIR8074,EXOC3L2,KCNJ14,BHMG1,KLK10,PSG9,SNAR-A2,SNORD34,SNORD88B,SLC6A16,PPP5D1,AKT1S1,LOC101059948,MYBPC2,PRG1,KLK14,ZNF541,MIR6800,IGLON5,PTH2,SNAR-A8,MIRLET7E,XRCC1,ZNF234,LOC101928295,ATF5,NTN5,SPHK2,NKG7,ZNF613,KLK6,DACT3-AS1,DMPK,CEACAM22P,FOSB,ZNF224,NANOS2,PTGIR,SIGLEC9,FPR2,CYTH2,TPRX1,SIGLEC17P,RASIP1,CARD8-AS1,PRKD2,GFY,NOSIP,TOMM40,SIGLEC5,HIF3A,MIR769,CBLC,SNRNP70,MIR150,SNORD35A,SYMPK,SNORD88C,RPL13A,ZNF227,KLK3,ACPT,FOXA3,PLAUR,FTL,IRF3,RCN3,IGFL1,BCAT2,KLK8,RUVBL2,RELB,EHD2,ETHE1,KLK12,VASP,APOC1,CCDC8,SNAR-F,SIGLEC11,MIR4750,PPFIA3,MIR6088,KCNA7,SNAR-E,ZNF175,ZNF226,GPR4,LOC105372441,KLK11,PTOV1-AS2,HRC,MIR642B,ZNF296,OPA3,ZNF112,IZUMO2,MIR6801,GLTSCR2-	3	gain

1428	CGTE_18	19	54301418	59093745	19q13.43-q13.42	LENG8,LILRA1,LILRA2,LILRB4,A1BG,MIR4754,LILRP2,SSC5D,MZF1,LILRB2,ZNF587B,ZNF417,GP6,NLRP7,ZSCAN18,ZFP28,ZNF304,GALP,CCDC106,PPP1R12C,ZNF135,ZNF524,ZIK1,ZNF549,NLRP5,ZNF582,HSPBP1,SBK3,ZSCAN22,ZNF606,LILRA4,MIR7975,ZNF530,KIR3DL2,ZNF17,ZSCAN5B,DNAAF3,LILRB5,ZNF324B,MIR6806,UBE2S,MBOAT7,FIZ1,ZNF584,LOC101928886,ZNF324,RFPL4A,TRAPPC2B,ZNF865,RDH13,TMEM86B,ZBTB45,FAM71E2,KIR3DL3,ZNF471,ZNF776,ZNF419,ZNF416,LENG1,MIR6804,MIMT1,ZSCAN4,MIR6805,SYT5,ZNF671,ZNF154,ZNF470,ZNF71,TMC4,SMIM17,ZNF460,NDUFA3,ZNF787,LENG9,ZNF542P,NLRP9,UBE2M,KIR2DL1,CENPBD1P1,TMEM190,MYADM,ZNF749,PEG3,TNNI3,ZSCAN5A,LAIR1,TRIM28,ZNF550,ZNF274,EPS8L1,NCR1,ZNF805,CACNG8,SLC27A5,RNF225,MIR6803,KIR2DS4,ZNF264,ZNF837,ZNF551,IL11,ZNF211,ZNF329,ZNF583,CDC42EP5,NLRP12,RFPL4AL1,PRPF31,ZNF543,RPL28,MIR935,TTYH1,ZNF134,PEG3-AS1,KIR3DX1,ZNF548,ZIM2,COX6B2,ZNF446,VSTM1,A1BG-AS1,LILRA3,CACNG6,USP29,ZNF772,ZNF667,NAT14,LOC101928804,FKBP1AP1,ZNF835,ZNF579,LILRB1,CACNG7,EPN1,ZNF552,U2AF2,FCAR,TSEN34,C19orf18,ZNF444,DUXA,MIR4752,MIR8061,ZNF497,SBK2,TFPT,ZNF581,ZNF8,RPS5,ZSCAN1,KIR3DL1,LILRA6,TARM1,RPS9,ZIM2-AS1,LILRA5,KIR2DL4,LAIR2,ZNF587,MIR6802,BRSK1,MZF1-AS1,KIR2DL3,CNOT3,NLRP11,CHMP2A,AURKC,OSCAR,LOC100128398,LILRB3,KMT5C,ZNF586,ZNF132,ZNF580,TMEM150B,ZNF773,NLRP8,ZNF547,ZNF582-AS1,MIR6807,ZNF256,TMEM238,ZNF628,ZNF814,TNNT1,LENG8-AS1,ZNF418,ZNF667-AS1,SHISA7,ISOC2,ZNF784,ZNF544,NLRP13,PTPRH,PRKCG,NLRP2,VN1R1,NLRP4,ZIM3,PPP6R1	3	gain
1429	CGTE_18	20	68259	2380624	20p13	TMEM74B,SDCBP2-AS1,NRSN2-AS1,SIRPG,DEFB127,SRXN1,MIR6869,TBC1D20,STK35,FKBP1A-SDCBP2,PSMF1,LOC100289473,RBCK1,SCRT2,SLC52A3,DEFB132,SIRPB1,SNPH,ANGPT4,DEFB128,DEFB125,SIRPA,TGM3,PDYN,LOC388780,DEFB126,SIRPD,TRIB3,RAD21L1,NSFL1C,CSNK2A1,SDCBP2,C20orf96,DEFB129,SIRPG-AS1,C20orf202,TGM6,FKBP1A,ZCCHC3,SIRPB2,NRSN2,TCF15,SOX12,FAM110A,RSPO4	4	gain
1430	CGTE_18	20	2380926	2552911	20p13	SNORD119,SNRPB,TGM6,ZNF343,TMC2	5	gain
1431	CGTE_18	20	2559703	10394194	20p13-p12.2	UBOX5,MRPS26,TMEM230,LAMP5-AS1,PRNT,PRNP,LINC00654,LOC101929125,SNORD57,HSPA12B,SNAP25,UBOX5-AS1,HAO1,C20orf196,CDS2,C20orf27,MKKS,CPXM1,FERMT1,EBF4,FASTKD5,PAK7,MCM8,C20orf141,CASC20,SLC4A11,PCED1A,MIR103A2,BMP2,LOC101929207,IDH3B,DDRGK1,PCNA,LINC00658,CHGB,AVP,PANK2,SNORD86,PLCB1,SMOX,TMC2,LOC101929312,OXT,TMEM239,C20orf194,SNORA51,LINC01428,VPS16,ATRN,MCM8-AS1,AP5S1,NOP56,MAVS,ITPA,GNRH2,LZTS3,PCNA-AS1,CDC25B,RASSF2,ADAM33,PTPRA,GPCPD1,LOC643406,PROKR2,SIGLEC1,MIR1292,GFRA4,CENPB,CRLS1,ANKEF1,SNORD56,LRRN4,ADRA1D,SLC23A2,SNORD110,TRMT6,SPEF1,RNF24,MIR8062,LINC01433,MIR103B2,PLCB4,TMX4,PRND,SNAP25-AS1,LOC101929371,LAMP5	3	gain
1432	CGTE_18	20	25597106	26189020	20p11.1-p11.21	FAM182A,FAM182B,NANP,NCOR1P1,LOC100134868,MIR663AHG,MIR663A,ZNF337,LOC101926955,LOC101926935,ZNF337-AS1	3	gain
1433	CGTE_18	20	43588844	44331069	20q13.12	WFDC6,MIR6812,WFDC12,SLPI,WFDC9,WFDC2,WFDC10A,SPINT3,SEMG2,MATN4,SEMG1,TP53TG5,EPPIN,PI3,STK4,EPPIN-WFDC6,PIGT,DBNDD2,SYS1-DBNDD2,KCNS1,TOMM34,SYS1,WFDC13,WFDC5,WFDC10B,WFDC8,SDC4,STK4-AS1,WFDC11,RBPJL	3	gain

1434	CGTE_18	20	49574895	54824841	20q13.13-q13.2	MIR3194,LINC01429,MOCS3,BCAS1,ZNF217,DOK5,SUMO1P1,TSHZ2,CBLN4,ATP9A,KCNG1,LINC01441,SALL4,MIR4756,LOC101927770,CYP24A1,ZFP64,LINC01524,NFATC2,PFDN4,LINC01440,DPM1,MC3R	3	gain
1435	CGTE_18	20	57598503	58349555	20q13.32	ATP5E,EDN3,SLMO2-ATP5E,ZNF831,TUBB1,PRELID3B,LOC100506384,PHACTR3	3	gain
1436	CGTE_18	20	58381088	58564286	20q13.33-q13.32	SYCP2,PHACTR3,FAM217B,PPP1R3D,CDH26	4	gain
1437	CGTE_18	20	58567391	62905019	20q13.33	NPBWR2,RPS21,MIR646,PCMTD2,MIR4758,MIR124-3,ZNF512B,C20orf195,HELZ2,EEF1A2,PPDPF,MIR4533,MIR1-1HG,COL20A1,MIR647,MIR646HG,PSMA7,MIR1-1,LOC101928048,SLCO4A1-AS1,RBBP8NL,HAR1B,MYT1,LINC01056,NTSR1,PRPF6,SLCO4A1,MIR941-2,ADRM1,ARFGAP1,SRMS,MIR133A2,MIR3196,LOC100130587,LINC00176,STMN3,SOX18,MIR941-4,TCEA2,ZBTB46,ZGPAT,LSM14B,HAR1A,RTEL1,MIR3195,CDH26,PTK6,SLC17A9,LOC63930,LINC00659,LOC100505771,LOC100506470,GID8,OGFR,RGS19,DIDO1,MIR548AG2,OSBPL2,LAMA5-AS1,GATA5,BHLHE23,LAMA5,TNFRSF6B,COL9A3,YTHDF1,ARFRP1,OGFR-AS1,C20orf197,FLJ16779,TAF4,ABHD16B,MIR941-1,DNAJC5,MIR1914,HRH3,MIR4326,NKAIN4,OPRL1,CHRNA4,MIR6813,MIR941-5,LOC729296,MIR1257,GMEB2,ZBTB46-AS1,CDH4,LIME1,DPH3P1,SS18L1,LKAAEAR1,TPD52L2,BIRC7,C20orf166-AS1,SLC2A4RG,SAMD10,UCKL1-AS1,MRGBP,KCNQ2,LINC00029,MTG2,CABLES2,MIR941-3,TCFL5,UCKL1,RTEL1-TNFRSF6B	3	gain
1438	CGTE_18	21	10793919	31234036	21q21.2-q21.3	NRIP1,C21orf91,LINC01425,TPTE,MIR155HG,BAGE3,CHODL-AS1,POTED,LOC101927973,MIR125B2,MIR4759,C21orf91-OT1,NCAM2,ADAMTS1,LOC388813,USP16,LOC101927869,LINC00317,ADAMTS5,LINC00158,ANKRD30BP2,GRIK1-AS2,GRIK1,JAM2,LOC102724188,MRPL39,BAGE,HSPA13,MIR99A,CCT8,MIR8069-2,BAGE2,BACH1,MIR548XHG,LIPI,LINC00161,N6AMT1,LINC00113,TMPRSS15,RBM11,LOC101927843,MIRLET7C,MIR3156-3,CYP4F29P,APP,GABPA,D21S2088E,LOC339622,LINC00308,CXADR,ATP5J,LINC01549,MIR5009,LINC00320,BACH1-IT2,LINC00515,ANKRD20A11P,LINC00314,MIR3118-1,CHODL,BAGE4,USP25,MIR155,LOC284825,CYYR1,MAP3K7CL,LINC00189,MIR99AHG,SAMSN1-AS1,RWDD2B,LTN1,BAGE5,GRIK1-AS1,ABCC13,BTG3,MIR8069-1,SAMSN1	3	gain
1439	CGTE_18	21	31311664	32410828	21q22.11-q21.3	KRTAP19-8,KRTAP19-7,KRTAP15-1,KRTAP7-1,KRTAP21-2,GRIK1,KRTAP8-1,KRTAP20-3,CLDN8,KRTAP19-4,KRTAP13-3,KRTAP19-5,KRTAP19-1,KRTAP22-1,KRTAP11-1,KRTAP13-4,KRTAP6-3,KRTAP23-1,KRTAP26-1,CLDN17,KRTAP19-6,KRTAP21-3,KRTAP22-2,MIR4327,KRTAP13-2,KRTAP20-1,LINC00307,KRTAP6-2,KRTAP19-2,KRTAP24-1,KRTAP20-2,KRTAP25-1,KRTAP19-3,KRTAP21-1,KRTAP13-1,KRTAP27-1,KRTAP20-4,KRTAP6-1	5	gain
1440	CGTE_18	21	42647170	42689035	21q22.3	FAM3B,BACE2	5	gain
1441	CGTE_18	21	45953454	46101952	21q22.3	KRTAP10-4,KRTAP12-4,KRTAP10-8,KRTAP10-3,TSPEAR,KRTAP10-5,KRTAP12-1,KRTAP10-11,KRTAP10-6,KRTAP10-9,KRTAP10-7,KRTAP10-2,KRTAP10-1,KRTAP12-3,KRTAP12-2,KRTAP10-10	3	gain
1442	CGTE_18	22	17063382	17395399	22q11.1	CCT8L2,TPTEP1,XKR3,HSFY1P1,ANKRD62P1-PARP4P3	4	gain
1443	CGTE_18	22	21212839	21213514	22q11.21	PI4KA,SNAP29	6	gain

1444	CGTE_18	22	22385409	23237666	22q11.22	VPREB1,IGLL5,LL22NC03-63E9.3,MIR5571,PRAME,POM121L1P,BMS1P20,ZNF280A,ZNF280B,MIR650,GGTLC2	3	gain
1445	CGTE_18	22	24373191	24384346	22q11.23	LOC391322,GSTT1,GSTT1-AS1	5	gain
1446	CGTE_18	22	25155788	26706762	22q12.1-q11.23	CRYBB3,TOP1P2,KIAA1671,SGSM1,TMEM211,MIR6817,MYO18B,SEZ6L,CRYBB2,ADRBK2,LOC100128531,LRP5L,PIWIL3,CRYBB2P1,IGLL3P	3	gain
1447	CGTE_18	22	39077900	39078343	22q13.1	TOMM22	5	gain
1448	CGTE_18	22	39360578	39382343	22q13.1	APOBEC3A_B,APOBEC3B	7	gain
1449	CGTE_18	X	30870781	38019602	Xp21.1-Xp11.4	PRRG1,CYBB,MAGEB16,CFAP47,FAM47B,FTHL17,LANCL3,FAM47A,XK,TMEM47,HYPM,SYTL5,MIR3915,FTH1P18,DYNLT3,FAM47C,RP11-87M18.2,TAB3,SRPX,DMD	3	gain
1450	CGTE_18	X	153924181	154774973	Xq28	RAB39B,FUNDC2,SNORA56,SMIM9,F8,F8A1,TMLHE-AS1,MIR664B,BRCC3,H2AFB3,GAB3,CLIC2,LOC101927830,MIR1184-3,F8A3,H2AFB2,H2AFB1,CMC4,MTCP1,VBP1,MIR1184-2,MPP1,DKC1,SNORA36A,TMLHE,MIR1184-1,F8A2	5	gain
1451	CGTE_18	Y	2655076	20826588	Yp11.1-Yq11.222	ZFY,TTTY8B,TTTY2B,TTTY23B,TTTY22,VCY1B,FAM41AY2,NLGN4Y-AS1,USP9Y,RBMY3AP,LINC00278,TSPY3,AMELY,TTTY9A,TTTY8,LINC00280,RBMY1A3P,TTTY23,TSPY4,TTTY7,SRY,TTTY7B,TTTY1B,CDY2B,FAM197Y2P,UTY,TTTY21B,TTTY19,DDX3Y,XKRY,TGIF2LY,TTTY21,TBL1Y,FAM224A,TSPY8,HSFY1,TTTY11,TSPY2,CDY2A,TTTY12,TMSB4Y,FAM224B,TTTY15,GYG2P1,PRKY,XKRY2,FAM197Y5P,TTTY1,TTTY20,FAM41AY1,TSPY10,PCDH11Y,VCY,TTTY2,TSPY1,TTTY16,NLGN4Y,TTTY9B,HSFY2,TTTY18,RPS4Y1	0	loss
1452	CGTE_18	Y	21154393	24460980	Yq11.222-Yq11.223	PRORY,CD24,TTTY5,TTTY6,TTTY14,BCORP1,RBMY1F,RBMY1B,RPS4Y2,RBMY2EP,TTTY13,RBMY2FP,RBMY1D,TTTY6B,EIF1AY,RBMY1J,RBMY1A1,PRY,RBMY1E,TTTY10,KDM5D,TXLNGY,PRY2,LOC101929148	0	loss
1453	CGTE_19	1	861266	1154087	1p36.33	RNF223,AGRN,C1orf159,SDF4,MIR200B,KLHL17,MIR200A,PERM1,TNFRSF18,LINC01342,PLEKHN1,MIR429,NOC2L,ISG15,TNFRSF4,HES4,SAMD11,TTLL10	1	loss
1454	CGTE_19	1	3385306	3512096	1p36.32	MIR551A,MEGF6,ARHGEF16	1	loss
1455	CGTE_19	2	128381736	128398619	2q14.3	LIMS2,MYO7B	1	loss
1456	CGTE_19	2	233164682	233409253	2q37.1	ALPI,CHRNG,DIS3L2,ECEL1P2,PRSS56,ECEL1,CHRND,ALPPL2,ALPP	1	loss
1457	CGTE_19	3	49688944	49701348	3p21.31	BSN	1	loss
1458	CGTE_19	3	52521197	52523688	3p21.1	NISCH	0	loss
1459	CGTE_19	3	52523945	52562758	3p21.1	NISCH,STAB1,NT5DC2	1	loss
1460	CGTE_19	3	126707504	126741135	3q21.3	PLXNA1	1	loss
1461	CGTE_19	3	130139954	130141680	3q22.1	COL6A5	4	gain
1462	CGTE_19	3	167191457	167240349	3q26.1	WDR49,SERPINI2	1	loss
1463	CGTE_19	4	2160770	2238194	4p16.3	HAUS3,POLN	3	gain
1464	CGTE_19	5	70785260	70849240	5q13.2	BDP1	3	gain
1465	CGTE_19	5	168690722	169028770	5q35.1	SLIT3,SPDL1	3	gain
1466	CGTE_19	6	30859818	30863284	6p21.33	DDR1	0	loss
1467	CGTE_19	6	33416529	33419732	6p21.32	SYNGAP1	0	loss
1468	CGTE_19	7	128470626	128587596	7q32.1	LOC100130705,ATP6V1F,FLNC,IRF5,KCP	1	loss
1469	CGTE_19	7	149473040	149523373	7q36.1	SSPO	1	loss
1470	CGTE_19	8	144802384	145781157	8q24.3	NRBP2,LRRC24,MIR7112,MIR6846,SCX,ARHGAP39,FAM83H-AS1,MIR6845,C8orf82,TONSL,ADCK5,MAF1,RECQL4,GPT,FBXL6,EPPK1,SLC52A2,PUF60,KIFC2,CYC1,MFSD3,HGH1,MIR937,SLC39A4,PPP1R16A,MIR6847,MIR4664,MAPK15,PLEC,GRINA,PARP10,DGAT1,MROH1,EXOSC4,TONSL-AS1,SPATC1,WDR97,BOP1,CPSF1,FAM83H,VPS28,CYHR1,SHARPIN,SCRIB,MIR939,HSF1,MIR661,OPLAH,MIR6848,GPAA1,SCRT1,MIR6893,TMEM249,MIR6849,LRRC14,FOXH1	1	loss
1471	CGTE_19	9	126132739	126146225	9q33.3	CRB2,DENND1A	1	loss

1472	CGTE_19	9	133223951	133230568	9q34.11	HMCN2	1	loss
1473	CGTE_19	9	139221204	139266530	9q34.3	DNLZ,GPSM1,DKFZP434A062,CARD9	1	loss
1474	CGTE_19	9	140389432	140395360	9q34.3	PNPLA7	0	loss
1475	CGTE_19	9	141012938	141016420	9q34.3	CACNA1B	0	loss
1476	CGTE_19	10	11543067	11567510	10p14	USP6NL	4	gain
1477	CGTE_19	10	105214830	105363195	10q24.33	NEURL1,CALHM1,NEURL1-AS1,SH3PXD2A,CALHM3	1	loss
1478	CGTE_19	10	135137692	135165001	10q26.3	PRAP1,CALY	1	loss
1479	CGTE_19	11	1010855	1018646	11p15.5	MUC6,AP2A2	3	gain
1480	CGTE_19	11	1018649	2608966	11p15.5	TNNI2,CD81-AS1,MUC5B,MIR7847,MRPL23,LINC01150,KRTAP5-5,TSPAN32,MOB2,FAM99B,KCNQ1,MIR6744,KRTAP5-1,LSP1,HOTS,MIR4686,MUC5AC,TH,SNORD131,IGF2-AS,TRPM5,ASCL2,DUSP8,LINC01219,MUC2,KRTAP5-2,H19,KRTAP5-AS1,MIR483,KRTAP5-3,TOLLIP-AS1,CD81,SYT8,IFITM10,KRTAP5-4,MUC6,C11orf21,KRTAP5-6,TSSC4,CTSD,FAM99A,INS,IGF2,TNNT3,INS-IGF2,TOLLIP,BRSK2,MIR675,MIR4298,MRPL23-AS1	1	loss
1481	CGTE_19	11	46394166	46401039	11p11.2	MIR4688,DGKZ	1	loss
1482	CGTE_19	12	124819031	124839034	12q24.31	MIR6880,NCOR2	0	loss
1483	CGTE_19	13	32885444	33306355	13q13.1	N4BP2L2,PDS5B,N4BP2L1,N4BP2L2-IT2,ZAR1L,BRCA2,MINOS1P1	3	gain
1484	CGTE_19	13	46820594	46841128	13q14.13	LRRC63	0	loss
1485	CGTE_19	13	53237221	53241143	13q14.3	SUGT1	1	loss
1486	CGTE_19	14	92140505	92278897	14q32.12	CATSPERB,TC2N	3	gain
1487	CGTE_19	14	96781439	96800894	14q32.2	ATG2B	3	gain
1488	CGTE_19	17	4269495	4463789	17p13.2	GGT6,UBE2G1,MYBBP1A,SPNS2,SPNS3	1	loss
1489	CGTE_19	17	60741821	60769686	17q23.2	MRC2	1	loss
1490	CGTE_19	18	2566831	2769870	18p11.32	METTL4,SMCHD1,CBX3P2,NDC80	3	gain
1491	CGTE_19	18	51691000	52928858	18q21.2	RAB27B,TCF4,LOC101927229,STARD6,C18orf54,CCDC68,SNORA37,MBD2,DYNAP,POLI	3	gain
1492	CGTE_19	20	3208382	3214966	20p13	SLC4A11	0	loss
1493	CGTE_19	21	34931629	34939659	21q22.11	SON	0	loss
1494	CGTE_19	21	45970688	46117586	21q22.3	KRTAP10-3,KRTAP10-12,KRTAP12-4,KRTAP12-2,KRTAP10-6,KRTAP10-4,KRTAP10-11,KRTAP12-1,KRTAP10-2,KRTAP10-8,KRTAP10-5,TSPEAR,KRTAP10-10,KRTAP12-3,KRTAP10-9,KRTAP10-7	1	loss
1495	CGTE_19	21	47418985	47575502	21q22.3	COL6A2,COL6A1,FTCD	1	loss
1496	CGTE_19	22	19131925	19164797	22q11.21	SLC25A1,DGCR14,GSC2,LINC01311	1	loss
1497	CGTE_19	22	21365694	21372431	22q11.21	TUBA3FP,P2RX6	1	loss
1498	CGTE_19	22	28389091	28397538	22q12.1	TTC28-AS1,TTC28	0	loss
1499	CGTE_19	22	31502455	31523136	22q12.2	INPP5J,SELM	0	loss
1500	CGTE_19	22	38019311	38038684	22q13.1	GGA1,LOC101927051,SH3BP1	1	loss
1501	CGTE_19	22	50688239	50751051	22q13.33	MAPK12,PLXNB2,DENND6B,HDAC10,MAPK11	1	loss
1502	CGTE_19	X	114413987	114424946	Xq23	LRCH2,RBMXL3	0	loss
1503	CGTE_19	X	153677986	153698971	Xq28	PLXNA3,MIR6858,FAM50A	1	loss

1504	CGTE_20	1	865582	6676969	1p36.33-p36.32	WRAP73,ESPN,MIR6808,UBE2J2,ATAD3A,PANK4,MIR551A,MMEL1,C1orf159,FAAP20,PERM1,MORN1,ISG15,HES2,MIR200B,VWA1,TPRG1L,ACTRT2,MIR6726,PHF13,LINC00982,LINC01134,PLEKHN1,HES3,CHD5,KLHL21,MIR4689,HES5,FAM213B,CPSF3L,LINC00337,PLEKHG5,PRDM16,ACOT7,CFAP74,AGRN,SAMD11,TTC34,LINC01342,SDF4,CPTP,MIR4417,LOC284661,PUSL1,GPR153,MIR4252,LINC01346,MIR429,TP73-AS1,C1orf233,NPHP4,TNFRSF18,SKI,CDK11B,SLC35E2B,TAS1R3,MIR4251,TNFRSF14,CCDC27,NOC2L,KLHL17,NOL9,SMIM1,MEGF6,AURKAIP1,GNB1,MIR6727,CALML6,ATAD3B,MMP23B,B3GALT6,TAS1R1,LOC100996583,FAM132A,ANKRD65,TNFRSF4,RER1,TMEM52,TMEM240,ICMT,DVL1,PRKCZ,ARHGEF16,MXRA8,LOC100129534,ACAP3,MRPL20,ATAD3C,DFFB,SSU72,C1orf174,TNFRSF25,LOC102724312,RNF207,GABRD,AJAP1,MMP23A,MIB2,SCNN1D,TTLL10,KCNAB2,LOC148413,RPL22,NADK,TMEM88B,PLCH2,CEP104,CDK11A,SLC35E2,LOC102724450,LRRC47,RNF223,LOC115110,MIR200A,HES4,CCNL2,PEX10,TP73,ZBTB48	1	loss
1505	CGTE_20	1	22151052	22592853	1p36.12	CELA3B,LOC101928043,LINC00339,MIR4418,LDLRAD2,HSPG2,CDC42,WNT4,CELA3A	1	loss
1506	CGTE_20	1	152079783	153113190	1q21.3	FLG,SPRR2F,IVL,FLG-AS1,LCE3B,LCE1F,SPRR2B,RPTN,LCE1D,LCE3D,LCE6A,LCE2D,C1orf68,LCE5A,LCE1C,LCE2A,SPRR2D,TCHH,LCE3E,LCE2C,SPRR2A,LCE1A,SMCP,SPRR2C,SPRR4,LCE3C,LCE2B,FLG2,LCE1E,LCE3A,SPRR1B,KPRP,SPRR2E,LCE4A,CRCT1,SPRR1A,HRNR,CRNN,LCE1B,SPRR3	3	gain
1507	CGTE_20	1	228108968	228613248	1q42.13	IBA57,IBA57-AS1,MRPL55,GUK1,MIR5008,TRIM17,WNT3A,ARF1,HIST3H3,C1orf35,MIR6742,MIR3620,TRIM11,C1orf145,WNT9A,GJC2,OBSCN	1	loss
1508	CGTE_20	2	128381736	128412192	2q14.3	MYO7B,LIMS2,GPR17	1	loss
1509	CGTE_20	2	136633824	141812875	2q21.3-q22.1	THSD7B,SPOPL,LRP1B,DARS-AS1,CXCR4,MIR7157,DARS,YY1P2,MCM6,HNMT,LOC101928273,NXPH2	3	gain
1510	CGTE_20	2	220307852	220502592	2q35	SPEG,OBSL1,CHPF,INHA,GMPPA,STK11IP,SLC4A3,LOC100996693,ASIC4,TMEM198,MIR3132	1	loss
1511	CGTE_20	2	233194365	233405564	2q37.1	ALPPL2,CHRNG,CHRND,ALPP,PRSS56,ECEL1,DIS3L2,ALPI,ECEL1P2	1	loss
1512	CGTE_20	2	241627709	242007386	2q37.3	AQP12A,AGXT,C2orf54,SNED1,KIF1A,LOC200772	1	loss
1513	CGTE_20	2	242663106	242842580	2q37.3	RTP5,D2HGDH,GAL3ST2,PDCD1,ING5,NEU4,LINC01237	1	loss
1514	CGTE_20	3	35723189	36873669	3p22.3-p22.2	MIR128-2,DCLK3,STAC,ARPP21,TRANK1	3	gain
1515	CGTE_20	3	52519734	52562758	3p21.1	NISCH,STAB1,NT5DC2	1	loss
1516	CGTE_20	3	129278459	129305563	3q22.1	PLXND1	1	loss
1517	CGTE_20	3	164730745	164906468	3q26.1	SLITRK3,SI	3	gain
1518	CGTE_20	4	493077	1834569	4p16.3	CTBP1,CTBP1-AS,PDE6B,MAEA,TMED11P,TMEM175,SLC26A1,SPON2,CPLX1,FGFR3,TACC3,DGKQ,SLBP,FGFRL1,IDUA,GAK,MFSD7,LETM1,CTBP1-AS2,NKX1-1,FAM53A,LOC100130872,UVSSA,LOC100129917,TMEM129,RNF212,CRIPAK,PCGF3,MYL5,PIGG,ATP5I	1	loss
1519	CGTE_20	4	165111242	165118448	4q32.3	MARCH1,ANP32C	6	gain
1520	CGTE_20	5	17275551	31323297	5p14.3-p14.1	LOC401177,BASP1,CDH18,SNORA105B,LOC101929681,CDH12,LINC01021,LOC340107,CDH9,LOC101929645,CDH10,C5orf17,PRDM9,LOC101929660,LSP1P3,PMCHL1,SNORA105A,GUSBP1,CDH6	3	gain
1521	CGTE_20	5	175933900	176316690	5q35.2	GPRIN1,SNCB,CDHR2,HK3,RNF44,MIR4281,UNC5A,TSPAN17,LINC01574,EIF4E1B,FAF2	1	loss

1522	CGTE_20	6	26020837	26285778	6p22.2	HIST1H3C,HIST1H4F,HIST1H3B,HIST1H1E,HIST1H2BG,HIST1H1D,HIST1H4A,HIST1H4G,HFE,HIST1H4D,HIST1H2BF,HIST1H2BD,HIST1H2AE,HIST1H4E,HIST1H4C,HIST1H3F,HIST1H3A,HIST1H1C,HIST1H2BE,HIST1H2BH,HIST1H2BI,HIST1H2AB,HIST1H1T,HIST1H4H,HIST1H2BB,HIST1H3E,HIST1H2AC,HIST1H2AD,HIST1H2BC,HIST1H3G,HIST1H4B,HIST1H3D	3	gain
1523	CGTE_20	6	30993283	30997903	6p21.33	MUC22	4	gain
1524	CGTE_20	6	32489819	32731352	6p21.32	HLA-DQB2,MIR3135B,HLA-DQB1,HLA-DRB1,HLA-DRB5,HLA-DQA2,HLA-DRB6,HLA-DQA1	3	gain
1525	CGTE_20	7	195557	2290609	7p22.3	ELFN1-AS1,MAD1L1,PDGFA,CYP2W1,PSMG3,LOC101927000,C7orf50,ADAP1,DNAAF5,MAFK,MIR339,MIR4655,INTS1,GET4,LOC442497,FAM20C,ZFAND2A,UNCX,FTSJ2,GPER1,NUDT1,LOC101927021,SUN1,GPR146,TFAMP1,WI2-2373I1.2,MICALL2,ELFN1,PRKAR1B,PSMG3-AS1,HRAT92,LOC101926963,COX19,TMEM184A	1	loss
1526	CGTE_20	7	44097563	44298577	7p13	DBNL,MYL7,POLM,MIR4649,GCK,AEBP1,MIR6838,YKT6,POLD2,CAMK2B,PGAM2	1	loss
1527	CGTE_20	7	128470626	128547673	7q32.1	ATP6V1F,FLNC,LOC100130705,KCP	1	loss
1528	CGTE_20	7	142021128	142482285	7q34	PRSS1,PRSS3P2,MTRNR2L6	3	gain
1529	CGTE_20	7	143573042	144095663	7q35	OR2A42,OR2A9P,OR6B1,ARHGEF35,LOC101928605,OR2A7,ARHGEF5,OR2F1,OR2A1-AS1,OR2F2,OR2A5,NOBOX,TCAF1,OR2A20P,OR2A2,OR2A1,OR2A14,OR2A25,CTAGE4,CTAGE8,OR2A12,ARHGEF34P	3	gain
1530	CGTE_20	7	149473040	149522466	7q36.1	SSPO	1	loss
1531	CGTE_20	7	150556781	150831617	7q36.1	AOC1,ABCB8,NOS3,KCNH2,TMUB1,ATG9B,CDK5,AGAP3,ASIC3,SLC4A2,FASTK	1	loss
1532	CGTE_20	8	41814533	41834637	8p11.21	KAT6A	5	gain
1533	CGTE_20	8	142366747	145830743	8q24.3	PLEC,KIFC2,TSNARE1,MIR6893,MIR939,RECQL4,MROH6,DGAT1,SLC52A2,PSCA,TMEM249,TONSL,MROH1,TONSL-AS1,CYHR1,PTP4A3,LOC100133669,ZNF623,LYNX1,LRRC24,C8orf82,RHPN1,FAM83H,NRBP2,MIR937,HSF1,MAFA,SHARPIN,LOC100288181,GPT,GPR20,MIR4664,GPIHBP1,TSTA3,CPSF1,SCRIB,MIR6848,EPPK1,BOP1,EEF1D,ZNF707,MIR6847,PARP10,SLC39A4,MAFA-AS1,VPS28,TIGD5,SPATC1,LYPD2,MFSD3,MROH5,LY6K,CCDC166,OPLAH,ARC,JRK,FAM83H-AS1,MIR4539,FOXH1,ZFP41,PPP1R16A,RHPN1-AS1,MAF1,C8orf31,GRINA,ADGRB1,BREA2,THEM6,CYC1,LRRC14,CYP11B2,ADCK5,WDR97,MAPK15,TOP1MT,MIR6845,SCX,CYP11B1,GLI4,GML,FBXL6,HGH1,SCRT1,LY6H,GPAA1,LY6D,EXOSC4,MIR6846,ZNF696,NAPRT,SLURP1,MIR661,PYCRL,LY6E,MIR6849,CDC42P3,ARHGAP39,PUF60,MINCR,MIR1302-7,GSDMD,LINC00051,ZC3H3,MIR7112,MIR4472-1	1	loss

1534	CGTE_20	9	135941913	140510590	9q34.3-q34.2	ENTPD8,TUBB4B,RNF208,VAV2,MIR3689E,LINC00094,TRAF2,MIR3689F,GLT6D1,MIR3689D1,MIR3689C,MIR4479,C8G,C9orf173,STKLD1,CARD9,MIR7114,OBP2B,SURF1,GRIN1,RALGDS,FBXW5,FAM163B,EDF1,LRRC26,UAP1L1,MIR3621,PNPLA7,BRD3,LINC01502,RNU6ATAC,LCN9,CCDC183,MIR3689D2,SNORD36A,SLC2A6,SURF2,DBH,DPH7,ABO,LCNL1,SNORD24,FCN2,SARDH,COL5A1,ANAPC2,FAM166A,MRPL41,ARRDC1-AS1,C9orf139,SAPCD2,ENTPD2,LOC401557,TMEM210,SEC16A,SNAPC4,PTGDS,PPP1R26,CAMSAP1,CCDC183-AS1,DPP7,MIR6722,OBP2A,ADAMTS13,ARRDC1,MIR3689B,NDOR1,SDCCAG3,INPP5E,PAEP,MED22,CACFD1,FCN1,ABCA2,REXO4,CYSRT1,QSOX2,PHPT1,NRARP,MIR4292,NSMF,TMEM8C,SURF4,LOC101928525,C9orf163,MIR3689A,MIR4673,C9orf173-AS1,MAN1B1,KCNT1,CEL,SLC34A3,SNHG7,SNORA17A,SNORA17B,ADAMTSL2,DNLZ,PMPCA,DBH-AS1,ZMYND19,DKFZP434A062,LOC101448202,SNORD36B,MRPS2,RABL6,GPSM1,PPP1R26-AS1,LCN1,GBGT1,NELFB,NPDC1,UBAC1,TMEM203,CELP,NALT1,EGFL7,MIR126,RPL7A,CLIC3,MIR4674,AGPAT2,LCN15,SSNA1,WDR5,FUT7,RXRA,SOHLH1,TPRN,MIR4669,RNF224,LCN6,C9orf62,MAN1B1-AS1,SURF6,C9orf69,OLFM1,LCN10,TMEM141,C9orf142,NOXA1,C9orf116,EXD3,MAMDC4,LHX3,SNORD36C,FAM69B,TOR4A,C9orf172,LCN12,LCN8,NACC2,LOC100128593,NOTCH1	1	loss
1535	CGTE_20	10	52086816	59975969	10q11.23-q21.1	A1CF,PRKG1,IPMK,LINC01468,CSTF2T,LOC102724719,DKK1,MIR3924,PRKG1-AS1,MTRNR2L5,MBL2,SGMS1,ASAH2B,PCDH15,ZWINT,MIR605,SGMS1-AS1	3	gain
1536	CGTE_20	10	133946791	135183618	10q26.3	DPYSL4,VENTX,ADAM8,KNDC1,PWWP2B,MIR202,MIR202HG,ADGRA1,C10orf91,LINC01166,LINC01167,LINC01168,UTF1,PRAP1,STK32C,ECHS1,CALY,CFAP46,TUBGCP2,FUOM,LRRC27,ADGRA1-AS1,NKX6-2,JAKMIP3,ZNF511,INPP5A	1	loss
1537	CGTE_20	11	319932	2608966	11p15.5	SNORD131,IGF2-AS,TALDO1,FAM99B,PIDD1,MIR483,DUSP8,LINC01150,CRACR2B,MIR7847,SNORA52,CTSD,CD81,INS-IGF2,MIR4686,PKP3,MUC2,TRPM5,POLR2L,LOC171391,HRAS,H19,LINC01219,CHID1,ASCL2,KRTAP5-AS1,MIR675,LRRC56,TSSC4,KRTAP5-5,C11orf21,IFITM3,MIR6744,AP2A2,MRPL23-AS1,TMEM80,IFITM10,LMNTD2,BRSK2,DEAF1,TSPAN4,LSP1,KRTAP5-4,SCT,TOLLIP,SYT8,CEND1,KCNQ1,ANO9,KRTAP5-6,TSPAN32,KRTAP5-3,RNH1,TOLLIP-AS1,TH,CD151,LOC143666,PNPLA2,MIR210HG,CD81-AS1,SLC25A22,CDHR5,DRD4,MUC5AC,TNNI2,FAM99A,IGF2,SIGIRR,PANO1,PTDSS2,RPLP2,KRTAP5-1,MIR210,EPS8L2,MIR4298,HOTS,KRTAP5-2,TNNT3,INS,PHRF1,IRF7,MUC6,MOB2,B4GALNT4,MRPL23,MUC5B,RASSF7,PDDC1	1	loss
1538	CGTE_20	11	63517312	64695304	11q13.1	MIR194-2HG,LOC100996455,PPP2R5B,PYGM,MIR6749,EHD1,PPP1R14B,OTUB1,MIR194-2,SLC22A12,FLRT1,KCNK4,DNAJC4,MIR6750,STIP1,MEN1,FKBP2,KCNK4-TEX40,FERMT3,PRDX5,MIR192,RCOR2,GPR137,C11orf84,RTN3,C11orf95,ESRRA,PLCB3,CCDC88B,SLC22A11,ATG2A,NRXN2,MARK2,RPS6KA4,RASGRP2,MIR1237,TRPT1,MACROD1,MAP4K2,TRMT112,VEGFB,CDC42BPG,MIR7155,NUDT22,NAA40,COX8A,BAD,SF1,TEX40	1	loss
1539	CGTE_20	11	123813658	123909798	11q24.2-q24.1	OR6T1,OR10G8,OR10S1,OR10G4,OR10G9,OR10G7	4	gain
1540	CGTE_20	12	7360083	7655405	12p13.31	CD163L1,ACSM4,PEX5,CD163	3	gain
1541	CGTE_20	12	40876490	40884792	12q12	MUC19	4	gain

1542	CGTE_20	12	57570810	57625671	12q13.3	SHMT2,MIR1228,LRP1,NXPH4	1	loss
1543	CGTE_20	12	132598985	133202381	12q24.33	DDX51,GALNT9,LOC101928416,POLE,P2RX2,LOC100130238,LRCOL1,NOC4L,EP400NL,MIR6763,FBRSL1	1	loss
1544	CGTE_20	13	33309287	41508190	13q13.3-q14.11	PROSER1,MIR4305,FREM2,STOML3,KL,EXOSC8,RFXAP,LINC00423,COG6,MIR621,CCNA1,TRPC4,LINC00437,LINC00547,PDS5B,SUGT1P3,SERTM1,MAB21L1,FOXO1,SUPT20H,CCDC169,SLC25A15,MRPS31,RFC3,UFM1,MIR320D1,TPTE2P5,LINC00571,SPG20,LINC00457,NHLRC3,SOHLH2,ELF1,SMAD9,LINC00598,LINC01048,NBEA,SPG20-AS1,ALG5,LINC00548,LHFP,STARD13-AS,DCLK1,POSTN,CCDC169-SOHLH2,MIR548F5,CSNK1A1L,LINC00332,LINC00366,STARD13,LINC00445	3	gain
1545	CGTE_20	13	61986973	72440412	13q21.2-q21.31	MIR4704,OR7E156P,LINC00358,KLHL1,LINC00395,PCDH9-AS3,PCDH9-AS4,LINC00383,LINC01052,PCDH9-AS2,LINC01075,LOC102723968,DACH1,LINC00364,ATXN8OS,PCDH20,LINC00348,MIR548X2,LINC00550,LINC00448,PCDH9,LINC00376	3	gain
1546	CGTE_20	14	24906371	27064759	14q12	LOC101927045,GZMB,KHNYN,NOVA1,CMA1,CTSG,SDR39U1,GZMH,STXBP6	3	gain
1547	CGTE_20	14	104561864	106437088	14q32.33	MTA1,KIAA0125,TMEM179,INF2,CRIP2,C14orf180,BTBD6,LOC102723354,C14orf80,JAG2,MIR4539,PACS2,CRIP1,MIR8071-2,AHNAK2,LOC100507437,ADSSL1,KIF26A,MIR203A,CEP170B,MIR4537,MIR4538,C14orf79,LINC00638,ZBTB42,NUDT14,GPR132,MIR4710,ASPG,AKT1,BRF1,SIVA1,TEX22,ADAM6,MIR203B,PLD4,MIR4507,MIR6765,MIR8071-1,TMEM121,ELK2AP,CDCA4	1	loss
1548	CGTE_20	15	20170004	22489980	15q11.2-q11.1	MIR5701-2,LOC646214,CHEK2P2,MIR5701-3,OR4M2,MIR3118-4,HERC2P3,OR4N3P,POTEB,POTEB3,POTEB2,NBEAP1,MIR3118-3,OR4N4,GOLGA8CP,LOC101927079,GOLGA6L6,MIR3118-2,LOC727924,MIR5701-1,CXADRP2,NF1P2,LINC01193	3	gain
1549	CGTE_20	15	42113991	42211608	15q15.1	EHD4,JMJD7-PLA2G4B,SPTBN5,MAPKBP1,PLA2G4B,JMJD7,MIR4310	1	loss
1550	CGTE_20	16	309429	3198092	16p13.3	TSR3,EME2,UNKL,PGP,ZG16B,GFER,MIR3176,MAPK8IP3,NTN3,NME3,CCDC64B,BRICD5,CACNA1H,RNF151,WFIKKN1,ZSCAN10,MSRB1,PRSS22,CLDN9,RNPS1,RPUSD1,JMJD8,PRR35,ARHGDIG,MIR3677,SNORA10,MIR1225,TPSG1,SLC9A3R2,GNPTG,SNORA78,MIR6511B2,METRN,C16orf91,RHBDL1,CHTF18,MIR5587,PRSS33,E4F1,PTX4,SNHG9,RGS11,PDPK1,LOC101929613,PRSS30P,NTHL1,DNASE1L2,NARFL,PDIA2,CAPN15,MMP25,FAM195A,LINC00254,C16orf59,SNORD60,IGFALS,MEIOB,ZNF205,HAGHL,MIR4516,CCDC78,IL32,SRRM2-AS1,CLDN6,NDUFB10,LOC106660606,LOC100128770,LINC00514,LOC652276,HCFC1R1,LOC100134368,ZNF213,CEMP1,MLST8,TCEB2,FAHD1,GNG13,FAM234A,UBE2I,MMP25-AS1,TELO2,CLCN7,STUB1,CCDC154,HAGH,RHOT2,SNHG19,SNORA64,WDR90,RAB40C,TMEM8A,TRAF7,FBXL16,SSTR5,MSLN,ERVK13-1,CCNF,SPSB3,TSC2,MIR6768,FLJ42627,RAB26,PKMYT1,DECR2,SSTR5-AS1,HN1L,LMF1,TPSB2,HS3ST6,MIR662,PIGQ,AXIN1,ABCA17P,NUBP2,NPW,THOC6,SYNGR3,ZNF213-AS1,ZNF598,MIR4717,TNFRSF12A,CASP16P,RPS2,PRSS27,AMDHD2,TBC1D24,MIR3178,FLYWCH2,SOX8,ZNF205-AS1,PRSS41,MIR6511B1,TBL3,BAIAP3,LMF1-AS1,PRR25,TMEM204,PRSS21,CRAMP1,NHLRC4,PKD1,NOXO1,CASKIN1,NME4,MRPL28,FLYWCH1,IFT140,TPSD1,TPSAB1,MIR3180-5,ABCA3,MIR6767,WDR24,RAB11FIP3,MRPS34,ATP6V0C,LINC00235,SRRM2,C1QTNF8,FAM173A,PAQR4,KREMEN2,MIR940,KCTD5,ECI1,RPL3L,C16orf13,MIR3177	1	loss

1551	CGTE_20	16	88763963	89261436	16q24.3	CTU2,CDH15,PIEZO1,MIR4722,LOC339059,RNF166,CBFA2T3,GALNS,LOC100129697,ACSF3,LOC400558,TRAPPC2L,PABPN1L,APRT,LINC00304,CDT1,LOC100289580	1	loss
1552	CGTE_20	18	9886821	9887946	18p11.22	TXNDC2	3	gain
1553	CGTE_20	18	67992223	67993430	18q22.2	SOCS6	4	gain
1554	CGTE_20	19	281411	2269667	19p13.3	TCF3,C19orf25,AZU1,BSG,LOC100288123,AP3D1,MISP,MIER2,POLRMT,UQCR11,HMHA1,MADCAM1,MIR1227,PLPPR3,SBNO2,CSNK1G2-AS1,GAMT,PRSS57,MEX3D,GZMM,ADAT3,PTBP1,APC2,CBARP,OAZ1,ADAMTSL5,CFD,MED16,MUM1,R3HDM4,POLR2E,GPX4,IZUMO4,CSNK1G2,PLPP2,ATP5D,REEP6,ABHD17A,MIR6789,GRIN3B,RNU6-2,ELANE,MIDN,STK11,AMH,MIR4745,MBD3,DOT1L,SHC2,SCAMP4,RPS15,PLEKHJ1,HCN2,NDUFS7,FSTL3,CIRBP-AS1,MIR1909,PLK5,REXO1,CNN2,SF3A2,PALM,BTBD2,DAZAP1,KLF16,MIR3187,FGF22,PCSK4,MOB3A,ONECUT3,TPGS1,EFNA2,THEG,C2CD4C,ATP8B3,CDC34,ARID3A,MIR4321,WDR18,KISS1R,TMEM259,ODF3L2,CIRBP,JSRP1,MKNK2,RNF126,C19orf24,PRTN3,ABCA7	1	loss
1555	CGTE_20	19	15784395	16060573	19p13.12	OR10H3,CYP4F24P,OR10H5,OR10H2,CYP4F11,OR10H4,CYP4F12,LOC102724279,OR10H1,CYP4F2,UCA1	3	gain
1556	CGTE_20	19	41403474	41600365	19q13.2	CYP2B7P,CYP2G1P,CYP2A13,CYP2B6	3	gain
1557	CGTE_20	19	42222142	42311251	19q13.2	CEACAM6,CEACAM5,CEACAM3	3	gain
1558	CGTE_20	19	55054628	55420907	19q13.42	LILRP2,KIR2DL4,LILRA2,LOC101928804,KIR3DL3,KIR3DL1,LILRB1,KIR3DX1,KIR3DL2,MIR8061,NCR1,KIR2DL3,LILRB4,LILRA1,KIR2DS4,KIR2DL1,FCAR	3	gain
1559	CGTE_20	19	55855038	56172560	19q13.42	ZNF865,ZNF580,SSC5D,ZNF524,SBK3,U2AF2,MIR6805,RPL28,IL11,ZNF579,TMEM190,NAT14,CCDC106,ZNF581,ZNF628,ZNF784,FIZ1,UBE2S,ISOC2,COX6B2,FAM71E2,TMEM238,SHISA7,SBK2,KMT5C	1	loss
1560	CGTE_20	20	60294263	62738309	20q13.33	LKAAEAR1,COL20A1,MIR941-4,OGFR-AS1,ABHD16B,RGS19,ARFGAP1,MIR133A2,TCEA2,SLCO4A1,MIR941-1,MTG2,NKAIN4,ZBTB46-AS1,SAMD10,OPRL1,ZGPAT,UCKL1-AS1,HRH3,MIR124-3,LOC100505771,SRMS,NPBWR2,GMEB2,MIR4758,RPS21,MIR941-5,LOC100130587,LINC00029,MIR1914,FLJ16779,MIR3195,HAR1B,RBBP8NL,SOX18,NTSR1,C20orf195,LINC00176,KCNQ2,ARFRP1,TCFL5,CDH4,C20orf166-AS1,SLC2A4RG,BHLHE23,LIME1,RTEL1,LSM14B,PTK6,PPDPF,LINC01056,OGFR,MIR1257,MIR1-1HG,TAF4,EEF1A2,DPH3P1,BIRC7,SS18L1,MIR941-2,LAMA5-AS1,PRPF6,LOC63930,MIR6813,ZNF512B,SLCO4A1-AS1,MIR3196,YTHDF1,MIR647,MRGBP,TNFRSF6B,LAMA5,GID8,HAR1A,UCKL1,TPD52L2,ZBTB46,MIR4326,MIR1-1,RTEL1-TNFRSF6B,CABLES2,PSMA7,DIDO1,COL9A3,CHRNA4,HELZ2,GATA5,ADRM1,OSBPL2,STMN3,MIR941-3,DNAJC5,SLC17A9,LINC00659	1	loss
1561	CGTE_20	21	31720609	32253929	21q22.11	KRTAP21-1,KRTAP19-6,KRTAP22-2,KRTAP13-4,KRTAP13-3,KRTAP21-2,KRTAP6-2,KRTAP6-1,KRTAP19-2,KRTAP6-3,KRTAP20-2,KRTAP15-1,KRTAP8-1,KRTAP19-1,KRTAP19-4,KRTAP11-1,KRTAP13-1,KRTAP20-4,KRTAP7-1,KRTAP23-1,KRTAP20-1,KRTAP19-3,MIR4327,KRTAP22-1,KRTAP19-5,KRTAP21-3,KRTAP20-3,KRTAP19-7,KRTAP13-2	3	gain

1562	CGTE_20	21	45649420	46572711	21q22.3	PTTG1IP,TSPEAR-AS2,KRTAP10-8,LINC00163,KRTAP10-2,LINC01424,KRTAP12-1,ITGB2-AS1,DNMT3L,FAM207A,UBE2G2,ITGB2,KRTAP10-6,KRTAP10-11,KRTAP10-1,ICOSLG,KRTAP10-4,KRTAP10-5,LINC00162,C21orf2,KRTAP12-2,KRTAP10-12,KRTAP12-4,KRTAP10-7,TSPEAR-AS1,KRTAP10-10,PFKL,KRTAP10-3,ADARB1,LRRC3-AS1,SUMO3,LRRC3,LINC01547,TRPM2,KRTAP12-3,KRTAP10-9,AIRE,SSR4P1,TSPEAR,TRPM2-AS	1	loss
1563	CGTE_20	21	46687210	47575502	21q22.3	COL18A1,COL18A1-AS1,COL18A1-AS2,LOC642852,POFUT2,LOC100129027,SLC19A1,COL6A2,FTCD,LINC00316,LOC101928796,MIR6815,PCBP3,COL6A1	1	loss
1564	CGTE_20	22	17063382	17395399	22q11.1	CCT8L2,XKR3,TPTEP1,HSFY1P1,ANKRD62P1-PARP4P3	3	gain
1565	CGTE_21	1	861266	1022680	1p36.33	AGRN,KLHL17,HES4,PERM1,ISG15,NOC2L,PLEKHN1,RNF223,C1orf159,SAMD11	1	loss
1566	CGTE_21	1	3391186	3477362	1p36.32	MEGF6,ARHGEF16,MIR551A	1	loss
1567	CGTE_21	1	53972246	53995691	1p32.3	GLIS1	1	loss
1568	CGTE_21	1	109810418	109816453	1p13.3	CELSR2	1	loss
1569	CGTE_21	1	156823569	156880176	1q23.1	PEAR1,NTRK1,INSRR	1	loss
1570	CGTE_21	1	228459619	228479809	1q42.13	OBSCN	1	loss
1571	CGTE_21	1	246797741	246805141	1q44	CNST	4	gain
1572	CGTE_21	2	71913554	72371345	2p13.2	DYSF,CYP26B1	1	loss
1573	CGTE_21	2	233164682	233405564	2q37.1	ALPPL2,ALPP,ALPI,ECEL1,PRSS56,ECEL1P2,DIS3L2,CHRND,CHRNG	1	loss
1574	CGTE_21	3	52521197	52523688	3p21.1	NISCH	0	loss
1575	CGTE_21	3	57545213	57561314	3p14.3	ARF4,PDE12	3	gain
1576	CGTE_21	3	72861711	72864603	3p13	SHQ1	4	gain
1577	CGTE_21	3	126707504	126741135	3q21.3	PLXNA1	1	loss
1578	CGTE_21	3	196669188	196675668	3q29	NCBP2-AS2,NCBP2,PIGZ	1	loss
1579	CGTE_21	6	32017678	32030067	6p21.33	TNXB	0	loss
1580	CGTE_21	6	84872803	84881443	6q14.2	CEP162	5	gain
1581	CGTE_21	7	100274997	100285885	7q22.1	GNB2,GIGYF1	0	loss
1582	CGTE_21	7	149473040	149523373	7q36.1	SSPO	1	loss
1583	CGTE_21	8	99761477	99787043	8q22.2	STK3	3	gain
1584	CGTE_21	8	144459324	145998710	8q24.3	BOP1,EPPK1,RECQL4,CYHR1,FBXL6,ZNF34,MROH6,TONSL,RHPN1,MIR6847,MIR6849,PLEC,ZNF707,HGH1,MROH1,SLC39A4,FAM83H,LRRC14,CYC1,FAM83H-AS1,TMEM249,MAFA,MIR937,ARHGAP39,OPLAH,CPSF1,ADCK5,MAF1,MIR6848,PARP10,MIR939,SCRIB,GPAA1,TIGD5,CCDC166,VPS28,SLC52A2,GSDMD,MIR6893,MIR6846,ZNF251,TSTA3,ZNF623,MAFA-AS1,DGAT1,KIFC2,NRBP2,NAPRT,MFSD3,WDR97,PUF60,MIR4664,FOXH1,PPP1R16A,PYCRL,GRINA,EXOSC4,MIR661,SHARPIN,LRRC24,GPT,MIR7112,MAPK15,C8orf82,HSF1,SCX,BREA2,SPATC1,TONSL-AS1,MIR6845,ZC3H3,SCRT1,EEF1D	1	loss
1585	CGTE_21	9	136426137	136439102	9q34.2	ADAMTSL2	0	loss
1586	CGTE_21	9	139820118	139982048	9q34.3	UAP1L1,C9orf139,C9orf142,MAN1B1-AS1,CLIC3,FUT7,LCN12,ABCA2,SAPCD2,FBXW5,LCNL1,C8G,PTGDS,NPDC1,MAN1B1,ENTPD2,TRAF2	1	loss
1587	CGTE_21	10	105254143	105350213	10q24.33	NEURL1,NEURL1-AS1	0	loss
1588	CGTE_21	10	134008268	134040427	10q26.3	STK32C,DPYSL4	1	loss
1589	CGTE_21	11	46394166	46401039	11p11.2	MIR4688,DGKZ	1	loss
1590	CGTE_21	11	64593981	64607796	11q13.1	CDC42BPG	1	loss
1591	CGTE_21	11	102953944	103039664	11q22.3	DCUN1D5,DYNC2H1	3	gain
1592	CGTE_21	12	105282760	105289199	12q23.3	SLC41A2	4	gain

1593	CGTE_21	12	122212749	122261630	12q24.31	RHOF,TMEM120B,LINC01089,SETD1B	1	loss
1594	CGTE_21	12	124819031	124840094	12q24.31	NCOR2,MIR6880	1	loss
1595	CGTE_21	13	72440672	73346085	13q21.33-q22.1	DACH1,MZT1,BORA,DIS3	3	gain
1596	CGTE_21	13	73410289	73482851	13q22.1	PIBF1	4	gain
1597	CGTE_21	13	96684081	96743893	13q32.1	UGGT2,HS6ST3	0	loss
1598	CGTE_21	14	31071283	31075059	14q12	G2E3	0	loss
1599	CGTE_21	14	105936119	106174506	14q32.33	CRIP2,TMEM121,MTA1,MIR8071-1,MIR8071-2,ELK2AP,CRIP1,C14orf80	1	loss
1600	CGTE_21	15	62172805	62174081	15q22.2	VPS13C	1	loss
1601	CGTE_21	15	79063423	79069094	15q25.1	ADAMTS7	0	loss
1602	CGTE_21	16	601972	943180	16p13.3	FBXL16,RHOT2,JMJD8,LMF1,WDR90,WFIKKN1,FAM173A,NARFL,RPUSD1,CAPN15,C16orf13,MSLN,CHTF18,METRN,FAM195A,GNG13,PIGQ,CCDC78,MIR662,PRR25,STUB1,HAGHL,WDR24,NHLRC4,RAB40C,RHBDL1,PRR35	1	loss
1603	CGTE_21	16	4733758	4749159	16p13.3	MGRN1,ANKS3,NUDT16L1	0	loss
1604	CGTE_21	17	4349375	4462503	17p13.2	GGT6,MYBBP1A,SPNS2,SPNS3	1	loss
1605	CGTE_21	18	48458636	48473474	18q21.2	ME2	3	gain
1606	CGTE_21	18	77156185	77193872	18q23	NFATC1	0	loss
1607	CGTE_21	19	55817586	56172064	19q13.42	TMEM150B,FIZ1,UBE2S,ZNF580,COX6B2,KMT5C,CCDC106,TMEM190,MIR6805,IL11,SSC5D,ZNF784,ZNF524,NAT14,SBK3,BRSK1,ZNF628,SBK2,ZNF865,ISOC2,FAM71E2,SHISA7,U2AF2,ZNF579,RPL28,ZNF581,TMEM238	1	loss
1608	CGTE_21	20	44580830	44642541	20q13.12	MMP9,ZNF335	1	loss
1609	CGTE_21	20	60882313	61150950	20q13.33	RPS21,C20orf166-AS1,LAMA5-AS1,RBBP8NL,ADRM1,MIR1-1HG,LAMA5,MIR4758,GATA5,CABLES2	1	loss
1610	CGTE_21	21	27081685	27087105	21q21.3	JAM2	1	loss
1611	CGTE_21	21	45678397	45758346	21q22.3	PFKL,AIRE,DNMT3L,C21orf2	1	loss
1612	CGTE_21	21	45959977	46117586	21q22.3	KRTAP10-3,KRTAP10-7,KRTAP10-1,KRTAP12-2,KRTAP12-1,KRTAP10-5,KRTAP10-10,KRTAP12-4,KRTAP10-9,KRTAP10-4,KRTAP12-3,KRTAP10-2,KRTAP10-6,KRTAP10-12,KRTAP10-11,TSPEAR,KRTAP10-8	1	loss
1613	CGTE_21	21	47411950	47575502	21q22.3	FTCD,COL6A1,COL6A2	1	loss
1614	CGTE_21	Y	15505657	15523163	Yq11.221	UTY	0	loss
1615	CGTE_22	1	861266	1251008	1p36.33	KLHL17,UBE2J2,TTLL10,SCNN1D,ACAP3,AGRN,SDF4,MIR429,SAMD11,PLEKHN1,HES4,TNFRSF4,NOC2L,MIR6727,ISG15,MIR6726,C1orf159,PERM1,B3GALT6,RNF223,MIR200A,CPSF3L,MIR200B,FAM132A,TNFRSF18,LINC01342,PUSL1	0	loss
1616	CGTE_22	1	1254154	1258750	1p36.33	CPSF3L	7	gain
1617	CGTE_22	1	3383677	3390282	1p36.32	ARHGEF16	6	gain
1618	CGTE_22	1	3391186	3542170	1p36.32	ARHGEF16,MEGF6,TPRG1L,MIR551A	0	loss
1619	CGTE_22	1	17248472	17294933	1p36.13	CROCC	0	loss
1620	CGTE_22	1	22084070	22454142	1p36.12	CDC42,CELA3A,LOC101928043,HSPG2,LINC00339,CELA3B,USP48,WNT4,LDLRAD2	0	loss
1621	CGTE_22	1	22456137	22592853	1p36.12	WNT4,MIR4418	8	gain
1622	CGTE_22	1	26508831	26517306	1p36.11	CNKSR1,CATSPER4	4	gain
1623	CGTE_22	1	228335094	228595830	1q42.13	TRIM17,IBA57,MIR6742,TRIM11,IBA57-AS1,OBSCN,GUK1,C1orf145,GJC2	0	loss
1624	CGTE_22	1	228595833	228612732	1q42.13	TRIM17,HIST3H3	7	gain
1625	CGTE_22	2	234728295	234732381	2q37.1	MROH2A	14	gain
1626	CGTE_22	2	239006590	239071484	2q37.3	SCLY,KLHL30,UBE2F-SCLY,FAM132B,ESPNL	0	loss
1627	CGTE_22	2	242694666	242815455	2q37.3	PDCD1,D2HGDH,RTP5,NEU4,GAL3ST2	0	loss
1628	CGTE_22	3	52543869	52546972	3p21.1	STAB1	1	loss
1629	CGTE_22	3	52547016	52548604	3p21.1	STAB1	27	gain
1630	CGTE_22	3	138664424	138763157	3q22.3-q23	PRR23A,PRR23B,FOXL2NB,PRR23C,FOXL2	0	loss
1631	CGTE_22	3	183952141	183954780	3q27.1	VWA5B2	6	gain
1632	CGTE_22	4	949946	1211207	4p16.3	TMEM175,TMED11P,LOC100130872,SPON2,FGFRL1,DGKQ,SLC26A1,CTBP1-AS,IDUA,CTBP1,RNF212	0	loss

1633	CGTE_22	4	1739332	1816262	4p16.3	FGFR3,LETM1,TACC3	0	loss
1634	CGTE_22	4	3432335	3450074	4p16.3	RGS12,HGFAC	0	loss
1635	CGTE_22	5	1708779	3600446	5p15.33	IRX2,LINC01019,IRX1,LINC01377,LINC01017,NDUFS6,IRX4,MRPL36,MIR4277,C5orf38,LOC100506858,LOC101929034,CTD-2194D22.4	0	loss
1636	CGTE_22	5	140167086	140264088	5q31.3	PCDHA7,PCDHA8,PCDHA3,PCDHA9,PCDHA2,PCDHA1,PCDHA11,PCDHA12,PCDHA10,PCDHA4,PCDHA13,PCDHA6,PCDHA5	0	loss
1637	CGTE_22	5	176859739	176860619	5q35.3	GRK6	6	gain
1638	CGTE_22	5	176860624	176919698	5q35.3	PRR7-AS1,DBN1,PDLIM7,GRK6,PRR7	0	loss
1639	CGTE_22	6	26156865	26385593	6p22.2	HIST1H2AD,HIST1H2BG,BTN3A2,HIST1H1D,HIST1H4F,HIST1H2AE,HIST1H2BD,HIST1H4G,HIST1H1E,HIST1H2BH,HIST1H3D,HIST1H2BE,HIST1H4E,HIST1H2BF,HIST1H3E,HIST1H2BI,BTN2A2,HIST1H3G,HIST1H4D,HIST1H4H,HIST1H3F	3	gain
1640	CGTE_22	6	33131385	33143435	6p21.32	COL11A2	3	gain
1641	CGTE_22	7	5401178	5467863	7p22.1	TNRC18	0	loss
1642	CGTE_22	8	21974388	22006206	8p21.3	REEP4,LGI3,HR	0	loss
1643	CGTE_22	8	142264799	142367014	8q24.3	GPR20,LINC01300	7	gain
1644	CGTE_22	8	144643895	144922047	8q24.3	BREA2,FAM83H,PYCRL,SCRIB,TSTA3,NRBP2,PUF60,GSDMD,EEF1D,MIR937,FAM83H-AS1,MAPK15,MIR6845,NAPRT,CCDC166,ZNF623,MROH6,MIR4664,ZNF707,TIGD5	0	loss
1645	CGTE_22	8	144922051	144941406	8q24.3	NRBP2,EPPK1	27	gain
1646	CGTE_22	8	144941407	145666471	8q24.3	MIR7112,MIR6846,CYC1,SLC39A4,FBXL6,SPATC1,PARP10,MIR661,MIR6849,MROH1,SCX,HSF1,CPSF1,DGAT1,ADCK5,SLC52A2,EXOSC4,MIR939,TONSL,GPAA1,MIR6848,PLEC,TMEM249,TONSL-AS1,WDR97,MAF1,SCRT1,GRINA,MIR6893,VPS28,OPLAH,SHARPIN,HGH1,EPPK1,MIR6847,BOP1	0	loss
1647	CGTE_22	8	145667477	145668656	8q24.3	TONSL	7	gain
1648	CGTE_22	8	145669132	145758721	8q24.3	RECQL4,TONSL,CYHR1,LRRC24,GPT,C8orf82,MFSD3,ARHGAP39,PPP1R16A,FOXH1,LRRC14,KIFC2	0	loss
1649	CGTE_22	9	139221204	139256633	9q34.3	GPSM1,DKFZP434A062,DNLZ	0	loss
1650	CGTE_22	9	139257347	139841290	9q34.3	CARD9,NOTCH1,INPP5E,MIR6722,EDF1,LCN8,MIR4674,NALT1,SNHG7,CCDC183-AS1,EGFL7,SNAPC4,LCN10,C9orf163,MAMDC4,RABL6,MIR4673,SNORA17A,C8G,SEC16A,SNORA17B,PMPCA,AGPAT2,FBXW5,C9orf172,PHPT1,CCDC183,LCN6,MIR4292,SDCCAG3,DNLZ,TRAF2,TMEM141,LOC100128593,FAM69B,MIR4479,LCN15,MIR126	1	loss
1651	CGTE_22	9	139846694	139917668	9q34.3	ABCA2,PTGDS,C9orf142,LCN12,CLIC3,LCNL1	0	loss
1652	CGTE_22	9	139917709	139918760	9q34.3	ABCA2	14	gain
1653	CGTE_22	9	139922497	140353055	9q34.3	NDOR1,SSNA1,EXD3,C9orf173-AS1,TMEM203,NSMF,ENTPD8,FAM166A,RNF208,LRRC26,ABCA2,NRARP,ANAPC2,TOR4A,UAP1L1,NPDC1,FUT7,TPRN,SLC34A3,SAPCD2,MIR3621,C9orf173,GRIN1,NELFB,NOXA1,ENTPD2,TUBB4B,MIR7114,C9orf139,MAN1B1,MAN1B1-AS1,TMEM210,RNF224,DPP7,CYSRT1	0	loss
1654	CGTE_22	9	140353421	140356100	9q34.3	NSMF,PNPLA7	13	gain
1655	CGTE_22	9	140356372	140395360	9q34.3	PNPLA7	0	loss
1656	CGTE_22	10	73565497	73569884	10q22.1	CDH23	6	gain
1657	CGTE_22	10	134915762	135009288	10q26.3	ADGRA1,KNDC1	0	loss

1658	CGTE_22	11	193051	2466762	11p15.5	CD81-AS1,TSSC4,IFITM5,CD151,LINC01150,LSP1,LMNTD2,AP2A2,B4GALNT4,SCGB1C1,IGF2,SIGIRR,CTSD,TMEM80,INS,TSPAN32,MIR210HG,IFITM10,MRPL23-AS1,SCT,IFITM1,RIC8A,TNNT3,POLR2L,TOLLIP,TNNI2,MOB2,EPS8L2,DRD4,RASSF7,IGF2-AS,MUC5AC,SCGB1C2,MIR483,SNORA52,KRTAP5-6,KRTAP5-2,PIDD1,SLC25A22,RNH1,KRTAP5-1,MIR6743,RPLP2,ATHL1,ASCL2,FAM99A,PSMD13,LRRC56,MIR675,TOLLIP-AS1,CEND1,TALDO1,KRTAP5-AS1,TSPAN4,MIR4686,BET1L,CHID1,PHRF1,H19,INS-IGF2,KCNQ1,IRF7,HRAS,SNORD131,LOC171391,MUC5B,MUC6,LOC143666,NLRP6,PNPLA2,HOTS,CDHR5,ODF3,IFITM3,MIR7847,BRSK2,PDDC1,PKP3,KRTAP5-3,C11orf21,MRPL23,PTDSS2,FAM99B,MUC2,DEAF1,MIR6744,MIR4298,SIRT3,IFITM2,TRPM5,ANO9,CRACR2B,KRTAP5-5,CD81,MIR210,SYT8,PANO1,TH,DUSP8,LINC01219,KRTAP5-4	0	loss
1659	CGTE_22	11	64022670	64026250	11q13.1	PLCB3	8	gain
1660	CGTE_22	11	64026325	64375632	11q13.1	LOC100996455,PRDX5,KCNK4,BAD,TRMT112,MIR1237,MIR7155,ESRRA,SLC22A11,CCDC88B,KCNK4-TEX40,PLCB3,TEX40,NRXN2,GPR137,RPS6KA4,SLC22A12	0	loss
1661	CGTE_22	11	67172841	67818329	11q13.2	C11orf72,CABP4,CDK2AP2,PTPRCAP,CARNS1,TBX10,DOC2GP,NDUFS8,TBC1D10C,MIR6753,CABP2,GPR152,ACY3,MIR4691,MIR7113,CORO1B,MIR6752,RPS6KB2,ALDH3B2,TCIRG1,AIP,ALDH3B1,NUDT8,NDUFV1,GSTP1,TMEM134,FAM86C2P,UNC93B1,PITPNM1	0	loss
1662	CGTE_22	12	124779984	124857156	12q24.31	FAM101A,MIR6880,NCOR2,ZNF664-FAM101A	0	loss
1663	CGTE_22	12	132271024	132396615	12q24.33	ULK1,MMP17,SFSWAP	0	loss
1664	CGTE_22	12	132397725	132399028	12q24.33	ULK1	12	gain
1665	CGTE_22	12	132399441	132405923	12q24.33	ULK1	0	loss
1666	CGTE_22	12	132504556	132505835	12q24.33	EP400	103	gain
1667	CGTE_22	13	45556210	45563877	13q14.12	NUFIP1,GPALPP1	5	gain
1668	CGTE_22	13	46357511	46358239	13q14.13	SIAH3	5	gain
1669	CGTE_22	13	46820594	46841128	13q14.13	LRRC63	6	gain
1670	CGTE_22	13	49796388	49830102	13q14.2	MLNR,CDADC1	5	gain
1671	CGTE_22	13	111102728	111111243	13q34	COL4A2	5	gain
1672	CGTE_22	13	113795187	113803926	13q34	F10	8	gain
1673	CGTE_22	13	114778596	114782883	13q34	RASA3	7	gain
1674	CGTE_22	14	104569793	105181171	14q32.33	KIF26A,TMEM179,MIR4710,MIR203B,MIR203A,ASPG,C14orf180,INF2	0	loss
1675	CGTE_22	14	105412779	105415182	14q32.33	AHNAK2	7	gain
1676	CGTE_22	14	105415266	106318638	14q32.33	TEX22,GPR132,JAG2,MIR8071-2,MIR8071-1,BRF1,NUDT14,PACS2,BTBD6,CRIP2,CDCA4,LOC100507437,TMEM121,CRIP1,AHNAK2,MTA1,LOC102723354,MIR6765,ELK2AP,C14orf79,C14orf80	1	loss
1677	CGTE_22	15	42133953	42134837	15q15.1	JMJD7-PLA2G4B,PLA2G4B	5	gain
1678	CGTE_22	15	42135798	42170978	15q15.1	SPTBN5,PLA2G4B,MIR4310,JMJD7-PLA2G4B	0	loss
1679	CGTE_22	16	677226	1435475	16p13.3	WDR24,MIR662,GNG13,SSTR5-AS1,JMJD8,FAM173A,LMF1,GNPTG,RPUSD1,BAIAP3,CHTF18,WFIKKN1,HAGHL,UNKL,LMF1-AS1,CACNA1H,C16orf13,SSTR5,RHOT2,FAM195A,TPSB2,STUB1,CCDC78,METRN,WDR90,RHBDL1,MSLN,SOX8,RAB40C,TPSG1,C1QTNF8,PRR25,UBE2I,TSR3,TPSD1,TPSAB1,FBXL16,NARFL	0	loss

1680	CGTE_22	16	1440612	3199803	16p13.3	RPL3L,KREMEN2,MIR6767,RAB26,SRRM2,MIR4717,IFT140,SRRM2-AS1,CLCN7,PGP,C16orf59,FAHD1,NTN3,MIR6511B1,SPSB3,PKMYT1,PRSS41,NDUFB10,MMP25-AS1,LOC106660606,ERVK13-1,CCDC154,PRSS27,SNHG9,PDPK1,ECI1,TBL3,TMEM204,MIR6511B2,PAQR4,LOC100128770,TSC2,LOC101929613,HCFC1R1,TELO2,EME2,E4F1,RNF151,MRPS34,KCTD5,SNORA78,SYNGR3,CRAMP1,PRSS21,TBC1D24,PRSS22,NUBP2,PTX4,ZNF213,ZNF205-AS1,HN1L,RNPS1,CCDC64B,C16orf91,FLYWCH1,SNORA10,MIR3178,THOC6,CLDN9,FLJ42627,SLC9A3R2,ZNF205,ABCA3,NOXO1,SNORA64,NME3,CEMP1,HAGH,BRICD5,LINC00254,ZNF213-AS1,IL32,PRSS33,IGFALS,MIR6768,MAPK8IP3,MIR4516,MLST8,SNHG19,MIR3177,LOC652276,TNFRSF12A,ATP6V0C,CASP16P,MIR3677,CCNF,NTHL1,TCEB2,CLDN6,LINC00514,PRSS30P,MEIOB,MIR3180-5,MIR940,GFER,MIR1225,FLYWCH2,SNORD60,ABCA17P,ZSCAN10,DNASE1L2,MMP25,ZNF598,MSRB1,RPS2,AMDHD2,ZG16B,TRAF7,CASKIN1,NPW,UNKL,PKD1,HS3ST6	1	loss
1681	CGTE_22	16	88052065	89266412	16q24.2-q24.3	IL17C,LOC339059,PIEZO1,LOC100289580,LOC400558,BANP,SNAI3-AS1,ZNF469,MVD,ZFPM1,TRAPPC2L,PABPN1L,ZC3H18,MIR5189,APRT,ACSF3,SLC22A31,CDT1,CBFA2T3,MIR4722,LINC00304,LOC100129697,LOC400553,RNF166,CDH15,LOC101928880,GALNS,CTU2,SNAI3,CYBA	0	loss
1682	CGTE_22	17	79092145	80051264	17q25.3	FAAP100,FAM195B,AATK-AS1,CEP131,MIR6786,CCDC137,MRPL12,RAC3,PCYT2,DCXR,GCGR,FASN,ASPSCR1,HGS,LINC00482,MAFG-AS1,GPS1,PYCR1,ARL16,MIR657,C17orf89,PPP1R27,ACTG1,NPLOC4,AATK,MIR338,STRA13,RFNG,OXLD1,P4HB,ARHGDIA,MIR1250,SIRT7,FSCN2,TSPAN10,MIR3065,LRRC45,MIR4740,ALYREF,SLC25A10,SLC38A10,ANAPC11,DUS1L,NOTUM,MYADML2,NPB,BAHCC1,LOC100130370,MAFG,MIR3186,TMEM105,SNORD134,ENTHD2,PDE6G	0	loss
1683	CGTE_22	18	46145781	46163065	18q21.1	CTIF	5	gain
1684	CGTE_22	18	72998553	73000165	18q22.3	TSHZ1	6	gain
1685	CGTE_22	18	77156185	77171501	18q23	NFATC1	0	loss
1686	CGTE_22	19	281411	3963752	19p13.3	JSRP1,GZMM,ABCA7,CSNK1G2-AS1,FZR1,GPX4,CFD,MFSD12,APBA3,THEG,MIR4745,ATCAY,MUM1,REEP6,MKNK2,SF3A2,GNG7,MRPL54,TLE6,ZNF77,ONECUT3,MOB3A,UQCR11,TLE2,MIER2,PLPPR3,MIDN,THOP1,SHC2,MIR1227,TMEM259,NCLN,CELF5,NMRK2,LOC100996351,KISS1R,ELANE,APC2,CNN2,WDR18,MIR7108,ZNF555,GADD45B,CSNK1G2,SPPL2B,SBNO2,BSG,ATP5D,RAX2,RNF126,CBARP,ADAMTSL5,TMPRSS9,GAMT,ZNF554,GIPC3,LMNB2,POLRMT,CDC34,TCF3,ZNF556,PALM,HMG20B,RNU6-2,PIP5K1C,SGTA,DIRAS1,TPGS1,MIR4321,EFNA2,PLEKHJ1,C19orf25,ZFR2,OAZ1,PLK5,STK11,SCAMP4,PLPP2,C2CD4C,FSTL3,DOT1L,AES,MATK,MIR1909,AZU1,HCN2,CACTIN,C19orf35,AP3D1,S1PR4,REXO1,ABHD17A,PCSK4,DOHH,GNA15,MEX3D,C19orf71,IZUMO4,LOC100288123,BTBD2,ARID3A,NFIC,POLR2E,SLC39A3,MIR637,TIMM13,MIR7850,PRSS57,DAPK3,PTBP1,GRIN3B,GNA11,TBXA2R,ODF3L2,MISP,SMIM24,MIR6789,ADAT3,AMH,RPS15,ZNF57,CIRBP,TJP3,HMHA1,FGF22,CIRBP-AS1,MIR1268A,KLF16,R3HDM4,MADCAM1,ATP8B3,MBD3,LSM7,PRTN3,NDUFS7,LINGO3,C19orf24,MIR3187,MED16,DAZAP1,CACTIN-AS1	0	loss
1687	CGTE_22	20	58456426	58467733	20q13.33	SYCP2	11	gain

1688	CGTE_22	20	60882999	61292077	20q13.33	CABLES2,MIR4758,GATA5,RPS21,MIR1-1,MIR133A2,RBBP8NL,SLCO4A1,LAMA5-AS1,MIR1-1HG,C20orf166-AS1,ADRM1,LAMA5	0	loss
1689	CGTE_22	20	61591678	61594717	20q13.33	SLC17A9	15	gain
1690	CGTE_22	20	61594900	62293905	20q13.33	LOC63930,MIR3196,PPDPF,FLJ16779,HAR1A,LINC00029,MIR124-3,KCNQ2,GMEB2,BHLHE23,SLC17A9,CHRNA4,HELZ2,LOC100505771,BIRC7,RTEL1-TNFRSF6B,MIR4326,ARFGAP1,C20orf195,STMN3,SRMS,LINC01056,RTEL1,LOC100130587,HAR1B,YTHDF1,COL20A1,EEF1A2,NKAIN4,PTK6	0	loss
1691	CGTE_22	20	62293910	62312246	20q13.33	RTEL1-TNFRSF6B,RTEL1	3	gain
1692	CGTE_22	20	62316696	62322371	20q13.33	RTEL1-TNFRSF6B,RTEL1	0	loss
1693	CGTE_22	21	46057516	46067022	21q22.3	KRTAP10-10,KRTAP10-11,TSPEAR	21	gain
1694	CGTE_22	21	46067186	47602866	21q22.3	ADARB1,COL18A1-AS1,LOC101928796,LINC01424,SUMO3,COL6A2,KRTAP12-1,MIR6815,SPATC1L,KRTAP10-12,LINC01547,KRTAP12-4,LINC00162,ITGB2,LINC00316,SSR4P1,COL18A1,COL18A1-AS2,ITGB2-AS1,KRTAP12-3,TSPEAR,LINC00163,FAM207A,FTCD,POFUT2,KRTAP12-2,UBE2G2,KRTAP10-11,PCBP3,COL6A1,LOC100129027,PTTG1IP,SLC19A1,LOC642852	1	loss
1695	CGTE_22	22	45601420	45609285	22q13.31	KIAA0930	10	gain
1696	CGTE_22	22	50609165	50955778	22q13.33	ADM2,TUBGCP6,NCAPH2,MAPK11,PANX2,SELO,DENND6B,PPP6R2,PLXNB2,MIOX,MAPK12,SBF1,TRABD,LMF2,HDAC10	0	loss
1697	CGTE_22	22	50955801	50957173	22q13.33	NCAPH2	16	gain
1698	CGTE_22	X	152914543	153051781	Xq28	IDH3G,PNCK,BCAP31,PLXNB3,SRPK3,DUSP9,SLC6A8,ABCD1	0	loss
1699	CGTE_22	X	153146107	153222187	Xq28	LCA10,ARHGAP4,NAA10,L1CAM,RENBP,HCFC1,AVPR2	0	loss
1700	CGTE_22	X	153688885	153690623	Xq28	PLXNA3	9	gain
1701	CGTE_22	X	153691606	153697115	Xq28	PLXNA3	0	loss
1702	CGTE_22	X	153697280	153698846	Xq28	PLXNA3	4	gain

1703	CGTE_23	1	29487092	60521262	1p32.3-p32.2	RPS8,MATN1-AS1,MKNK1,GJB5,LINC01135,CITED4,TMEM125,SSBP3,DNALI1,ATP6V0B,C1orf87,TFAP2E,KIF2C,SH3D21,RAD54L,DCDC2B,KIAA1522,TMEM59,MIR761,HSD52,DMBX1,TTC39A-AS1,TMEM61,NKAIN1,C1orf109,FAM183A,MIR6079,LOC105378732,OSCP1,LOC101929406,LURAP1,MIR6734,TMEM39B,LOC101929626,RIMKLA,MIR6735,FOXE3,AKR1A1,CCDC163P,SNORD103A,MIR3605,FOXO6,AK2,AGO1,LINC01137,IPP,GJB3,IQCC,ZCCHC11,C1orf216,GPX7,POMGNT1,CDCA8,TAL1,RNF11,TEX38,HDAC1,LOC101929464,MIR4420,AGO3,PCSK9,DAB1-AS1,SNRNP40,GJB4,SMIM12,MTF1,POU3F1,MIR4781,ZMYM1,MIR5585,HCRTR1,ZBTB8OS,SMAP2,RPS15AP10,MAGOH,CYP4B1,DMRTB1,EIF2B3,ZYG11A,LAPTM5,NFYC,KCNQ4,CDC20,MFSD2A,LCK,BEND5,ZBTB8A,CCDC30,MEAF6,CYP4X1,SNORD38A,NSUN4,OMA1,LOC101929516,CCDC24,PHC2,PUM1,SRSF4,CTPS1,MIR4422,TIE1,MIR1273F,EFCAB14-AS1,TTC22,KDM4A-AS1,MROH7-TTC4,LRRC41,GUCA2B,BEST4,PPIEL,RLF,NFYC-AS1,EPS15,C1orf185,YRDC,PRDX1,MTMR9LP,FNDC5,SYNC,PPIE,SLC2A1,TEKT2,TMEM69,SNORD103C,SNORA110,CDCP2,PPIH,CPT2,SVBP,FAM229A,A3GALT2,LOC653160,TTC39A,SPOCD1,NASP,CYP2J2,TTC4,ZYG11B,CFAP57,ST3GAL3,C1orf168,TCTEX1D4,CCDC28B,MIR6500,P3H1,EVA1B,KPNA6,CSMD2-AS1,LOC101928460,C1orf50,FAM159A,SKINTL,RHBDL2,DIO1,TXLNA,SERINC2,CYP4Z1,LOC101929592,GJA4,MMACHC,CCDC17,CSMD2,NRDC,ZSCAN20,HPCA,EIF3I,MARCKSL1,ZMYND12,MACF1,MAP7D1,HECTD3,TSSK3,GUCA2A,LOC101926944,COA7,LOC100507634,AGBL4,MKNK1-AS1,HPCAL4,SLC2A1-AS1,MOB3C,SLFNL1,INPP5B,CSF3R,TSPAN1,MYCBP,PPCS,RRAGC,AZIN2,UROD,CYP4Z2P,ADGRB2,LINC01343,SFPQ,MIR5581,NCDN,MIR5584,MIR4255,FOXD2-AS1,NDUFS5,ZMYM6NB,KTI12,C1orf228,ZFP69,DHCR24,CYP4A11,GJA9,ELOVL1,FAAH,JUN,EXO5,MIR1273G,FABP3,AKIRIN1,FAM151A,SLC5A9,SNORD46,ZFP69B,FOXD2,ERMAP,EPHA10,MIR4421,MRPL37,C1orf123,LOC101929721,LSM10,COL9A2,SCMH1,BSDC1,MIR30E,PODN,STK40,ECHDC2,GLIS1,TMEM54,SLC6A9,BMP8A,PRKAA2,NT5C1A,PARS2,SNORA55,YARS,PLPP3,KIAA0319L,SDC3,MIR3659,ADPRHL2,EBNA1BP2,TXNDC12-AS1,RSPO1,ERI3,FAM167B,FGGY,SLFNL1-AS1,C8A,KLF17,GJA9-MYCBP,TRIM62,GPBP1L1,COL16A1,PEF1,TRAPPC3,RNF19B,SLC1A7,TINAGL1,SSBP3-AS1,SNORD55,DPH2,IPO13,ZC3H12A,MIR4254,PPT1,MPL,BTF3L4,MROH7,MIR6732,CYB5RL,ELAVL4,HSPB11,LOC100507564,PIK3R3,OXCT2,TXNDC12,SF3A3,KIAA0754,OSBPL9,UTP11L,BSND,TRIT1,MANEAL,RBBP4,PABPC4,TACSTD2,ZMPSTE24,AGBL4-IT1,PTPRU,DLGAP3,LRP8,LOC105378683,USP24,DMAP1,HMGB4,PTPRF,MATN1,SCP2,TRABD2B,FAAHP1,MIR5095,COL8A2,FAF1,MIR552,SNIP1,MED8,PTCH2,YBX1,LRRC42,ORC1,FOXJ3,MUTYH,CLSPN,ACOT11,PRPF38A,SLC25A3P1,ZMYM4,LINC00853,LDLRAD1,HOOK1,S100PBP,LOC339539,TESK2,BTBD19,GNL2,ZBTB8B,KNCN,LINC01225,LOC100129924,ZNF684,TMEM53,TCEANC2,LINC01144,RNF220,DAB1,B4GALT2,KHDRBS1,EFCAB14,EDN2,C1orf122,LINC01398,C1orf94,CMPK1,ZNF691,RIMS3,MECR,ERI3-IT1,PSMB2,CYP4A22,KDM4A,NDC1,CLDN19,ZNF362,ZSWIM5,YIPF1,FHL3,AGO4,LEXM,MIR4711,MIR6733,LINC01226,BMP8B,LINC01389,LINC01358,THRAP3,ZFYVE9,ATPAF1,TOE1,TMCO2,SZT2,CAP1,MYSM1,HIVEP3,MAST2,PTP4A2,GRIK3,SNORD38B,C1orf210,MIR30C1,RAB3B,MYCL,C8B,HEYL,MRPS15,HYI,STIL,CC2D1B,ARTN,CDKN2C,PLK3,DMRTA2,ZMY	3	gain
1704	CGTE_23	1	60538210	63879837	1p31.3-p32.1	LINC00466,FOXD3-AS1,TM2D1,KANK4,NFIA-AS1,INADL,ALG6,NFIA,ANGPTL3,NFIA-AS2,C1orf87,LOC101926964,MGC34796,DOCK7,MIR3116-2,USP1,MIR6068,L1TD1,MIR3116-1,ATG4C,FOXD3	4	gain
1705	CGTE_23	1	63881498	66458912	1p31.3	ROR1-AS1,CACHD1,ALG6,LEPR,DNAJC6,MIR4794,RAVER2,ITGB3BP,MIR3671,EFCAB7,JAK1,LEPROT,PDE4B,PGM1,LINC01359,AK4,MIR101-1,UBE2U,DLEU2L,ROR1	6	gain

1706	CGTE_23	1	66999959	68513106	1p31.3	GNG12,C1orf141,IL12RB2,MIER1,WDR78,SLC35D1,DIRAS3,GNG12-AS1,MIR3117,SERBP1,TCTEX1D1,IL23R,GADD45A,SGIP1,INSL5	4	gain
1707	CGTE_23	1	84417825	84649859	1p31.1	PRKACB,TTLL7	3	gain
1708	CGTE_23	1	150478079	150941700	1q21.3	CTSS,ADAMTSL4,SETDB1,MIR4257,ECM1,FALEC,CERS2,TARS2,CTSK,ADAMTSL4-AS1,HORMAD1,ENSA,MCL1,GOLPH3L,ARNT	4	gain
1709	CGTE_23	1	154574309	155265591	1q22-q21.3	EFNA3,ZBTB7B,DCST2,PKLR,GBA,HCN3,SHC1,ADAR,SCAMP3,MTX1,SLC50A1,TRIM46,CKS1B,MIR4258,FLAD1,GBAP1,PYGO2,THBS3,KRTCAP2,ADAM15,DCST1,EFNA1,LENEP,CLK2,MUC1,EFNA4,DPM3,PMVK,KCNN3,FAM189B,LOC100505666,PBXIP1,MIR92B	3	gain
1710	CGTE_23	2	11484064	11587754	2p25.1	ROCK2,LINC00570,E2F6	7	gain
1711	CGTE_23	2	47612184	47613708	2p21	EPCAM	13	gain
1712	CGTE_23	2	65333470	65357196	2p14	RAB1A	3	gain
1713	CGTE_23	2	70082038	70092046	2p13.3	GMCL1	7	gain
1714	CGTE_23	2	73052921	73114960	2p13.2	SPR,EXOC6B	6	gain
1715	CGTE_23	2	73115479	73144168	2p13.2	SPR	3	gain
1716	CGTE_23	2	73145021	73151630	2p13.2	EMX1	9	gain
1717	CGTE_23	2	74741195	74743597	2p13.1	TLX2	5	gain
1718	CGTE_23	2	114684854	114688902	2q14.1	ACTR3	12	gain
1719	CGTE_23	2	120639292	120684189	2q14.2	PTPN4	3	gain
1720	CGTE_23	2	150432241	150433145	2q23.2	MMADHC	3	gain
1721	CGTE_23	2	178481375	178482345	2q31.2	TTC30A	3	gain
1722	CGTE_23	2	219738134	219757656	2q35	WNT6,WNT10A	3	gain
1723	CGTE_23	2	219757660	219825892	2q35	WNT10A,LINC01494,CDK5R2	6	gain
1724	CGTE_23	2	239037238	239040249	2q37.3	ESPNL	8	gain
1725	CGTE_23	3	5212065	5214335	3p26.1	ARL8B	4	gain
1726	CGTE_23	3	73096187	73110159	3p13	PPP4R2	4	gain
1727	CGTE_23	3	97516817	97562115	3q11.2	ARL6,CRYBG3	5	gain
1728	CGTE_23	3	97823363	97888546	3q11.2	OR5H14,OR5H15,OR5H1	4	gain
1729	CGTE_23	3	97926315	101298931	3q12.2-q11.2	DCBLD2,OR5K2,ST3GAL6-AS1,FILIP1L,TMEM30C,FAM172BP,ADGRG7,CLDND1,NIT2,TFG,OR5K1,GPR15,OR5H2,ST3GAL6,PCNP,OR5K3,OR5H6,HP09053,COL8A1,LNP1,SENP7,MIR548G,IMPG2,TMEM45A,ABI3BP,CMSS1,TBC1D23,OR5K4,MIR3921,CPOX,TOMM70A,TRMT10C	3	gain
1730	CGTE_23	3	101304175	101309063	3q12.3	PCNP	10	gain
1731	CGTE_23	3	101309068	101504565	3q12.3	RPL24,CEP97,ZBTB11,ZBTB11-AS1,PDCL3P4,NXPE3,PCNP	3	gain
1732	CGTE_23	3	101519919	101578320	3q12.3	NXPE3,NFKBIZ	5	gain
1733	CGTE_23	3	132393844	132394651	3q22.1	UBA5,NPHP3-ACAD11	5	gain
1734	CGTE_23	3	138724504	138739537	3q23	PRR23B,PRR23A	5	gain
1735	CGTE_23	3	160952812	160955897	3q26.1	NMD3	12	gain
1736	CGTE_23	4	1803014	1825588	4p16.3	FGFR3,LETM1	3	gain
1737	CGTE_23	4	16227830	16229109	4p15.32	TAPT1-AS1,TAPT1	5	gain
1738	CGTE_23	4	74270463	74270940	4q13.3	ALB	4	gain
1739	CGTE_23	4	84391230	84397791	4q21.23	FAM175A	4	gain
1740	CGTE_23	4	84397796	84403309	4q21.23	FAM175A	1	loss
1741	CGTE_23	4	146083834	146086303	4q31.21	OTUD4	6	gain
1742	CGTE_23	5	34918526	34922457	5p13.2	BRIX1	5	gain
1743	CGTE_23	5	115202421	115230941	5q23.1	AP3S1	5	gain
1744	CGTE_23	5	140167086	140181934	5q31.3	PCDHA2,PCDHA3,PCDHA1	3	gain
1745	CGTE_23	5	140188016	140229220	5q31.3	PCDHA6,PCDHA2,PCDHA9,PCDHA5,PCDHA7,PCDHA3,PCDHA8,PCDHA1,PCDHA4	4	gain

1746	CGTE_23	5	140229291	140230298	5q31.3	PCDHA8,PCDHA3,PCDHA4,PCDHA1,PCDHA2,PCDHA9,PCDHA7,PCDHA5,PCDHA6	5	gain
1747	CGTE_23	5	140230367	140263947	5q31.3	PCDHA10,PCDHA3,PCDHA8,PCDHA13,PCDHA1,PCDHA4,PCDHA12,PCDHA11,PCDHA2,PCDHA9,PCDHA5,PCDHA7,PCDHA6	3	gain
1748	CGTE_23	6	21594561	31237012	6p22.3-p22.1	PRR3,HLA-A,IFITM4P,ZNRD1,LOC100131289,FAM65B,ALDH5A1,HCG4B,BTN3A1,HIST1H2BE,HIST1H2AH,TRIM10,BTN3A3,LINC00240,TCF19,HLA-E,GPX6,TRIM39,DCDC2,HIST1H3B,HIST1H2AB,HIST1H4I,PPP1R18,ZNF311,TDP2,HIST1H2BD,VARS2,MUC22,ZSCAN31,HIST1H1C,C6orf62,ZKSCAN8,LINC01623,ZNF184,TRIM31-AS1,HIST1H4F,KAAG1,OR2W1,LOC100129636,HIST1H2BL,HIST1H2AG,HIST1H2AC,ZSCAN9,RNF39,LINC00243,HLA-H,NKAPL,HIST1H2AA,TRIM27,HIST1H1B,ZKSCAN3,PSORS1C2,BTN1A1,SLC17A4,MIR3143,HLA-C,MAS1L,ABCF1,HIST1H2AL,HIST1H2BG,SFTA2,LOC100270746,HLA-F-AS1,HIST1H1A,ZSCAN12P1,ZNF322,GPLD1,DPCR1,MDC1-AS1,HIST1H4A,OR2B2,GMNN,NRM,OR10C1,RPP21,LINC01556,PSORS1C3,ZSCAN16,HIST1H4L,PSORS1C1,OR2B6,ZNF391,BTN2A3P,LOC285819,NRSN1,BTN3A2,IER3,CDSN,GUSBP2,ZSCAN26,C6orf229,FLOT1,HIST1H2AE,CASC15,ZBED9,HIST1H2AD,TRIM39-RPP21,ZSCAN16-AS1,HIST1H2BC,MOG,HCG4,NBAT1,DHX16,HLA-F,HCG27,MRPS18B,PRSS16,HMGN4,ZNF165,MIR4640,HIST1H4K,HIST1H4H,HIST1H4E,SLC17A2,TUBB,LOC554223,GABBR1,HIST1H3F,HCG22,HIST1H3E,SCGN,HIST1H2AM,POU5F1,TRIM38,OR2B3,HIST1H1D,HIST1H3D,HCG8,OR2J3,HIST1H2BK,HIST1H2BJ,ABT1,UBD,HLA-L,HIST1H4J,MRS2,HIST1H2BN,TRIM26,HIST1H2AJ,OR12D3,HCG14,HIST1H4C,LRRC16A,TRIM40,HIST1H2AI,DDR1,ZSCAN23,HIST1H4B,TRIM31,HIST1H3J,HLA-G,ACOT13,OR11A1,HIST1H2BF,HIST1H2BI,GNL1,HCG18,CMAHP,HDGFL1,ATAT1,HIST1H1E,ZNRD1-AS1,ZNF204P,HIST1H3G,HIST1H1T,HIST1H2BM,HIST1H2APS1,HLA-J,LINC01015,ZFP57,TRIM15,ZKSCAN4,HCG17,PPP1R11,OR2H1,HIST1H2BA,KIAA0319,HCG11,OR12D2,C6orf15,GTF2H4,HIST1H3H,PGBD1,C6orf136,GPX5,LINC01012,TOB2P1,ZSCAN12,HIST1H2AK,SOX4,LOC101928663,HIST1H3I,HIST1H2BO,CCHCR1,OR5V1,OR14J1,MIR877,HIST1H2BB,OR2J2,HIST1H3A,HFE,PRL,HIST1H4D,BTN2A1,HIST1H4G,BTN2A2,MIR6891,HIST1H2BH,PPP1R10,SNORD32B,ZNF192P1,OR2H2,HCG9,POM121L2,SLC17A1,VN1R10P,HIST1H3C,MDC1,SLC17A3,MUC21	3	gain
1749	CGTE_23	6	31237140	31379176	6p21.33	HLA-C,HLA-B,MICA,MIR6891	4	gain
1750	CGTE_23	6	31379650	32427941	6p21.32-p21.33	NEU1,C4A,NFKBIL1,SNORD52,EHMT2,LY6G6F,LOC100507547,HCG26,TNXA,MIR6833,STK19,CFB,C4B_2,PBX2,HLA-DRA,C4B,SNORD48,C6orf48,MIR4646,LY6G5C,CYP21A2,BAG6,CYP21A1P,SLC44A4,C6orf25,MICA,SNORD117,ATF6B,RNF5,MSH5-SAPCD1,LSM2,AIF1,FKBPL,BTNL2,DDAH2,PPT2,SNORA38,CLIC1,LY6G5B,NCR3,AGER,C2,HCG23,PRRC2A,GPSM3,SAPCD1,VARS,RNF5P1,SAPCD1-AS1,APOM,LY6G6D,AGPAT1,LST1,LTA,DXO,HSPA1A,MIR6721,C6orf47,DDX39B,C2-AS1,MSH5,PRRT1,MIR6832,NELFE,LY6G6E,ATP6V1G2,SKIV2L,HSPA1B,ATP6V1G2-DDX39B,HCP5,SNORD84,TNXB,NOTCH4,MICB,PPT2-EGFL8,C6orf10,HSPA1L,GPANK1,LY6G6C,LTB,MCCD1,VWA7,MIR1236,ZBTB12,CSNK2B,DDX39B-AS1,ABHD16A,EGFL8,TNF	3	gain

1751	CGTE_23	6	32485399	32729654	6p21.32	HLA-DQA2,HLA-DRB5,HLA-DQA1,HLA-DQB1,HLA-DRB6,HLA-DRB1,MIR3135B,HLA-DQB2	4	gain
1752	CGTE_23	6	32731082	43747577	6p21.32-p21.31	HLA-DMA,BRD2,TREML2,TSPO2,GNMT,HLA-DMB,GUCA1A,ADCY10P1,LOC101929555,SLC22A7,FOXP4,FRS3,MIR6873,DAXX,PHF1,DNAH8,SLC26A8,SPDEF,KLHDC3,CUL7,B3GALT4,TEAD3,VPS52,RPS18,CLPS,GLO1,APOBEC2,WDR46,UBR2,C6orf226,MDFI,ABCC10,KLC4,CUTA,NFYA,POLR1C,MRPS10,PXT1,TAP1,MIR219A1,SRPK1,ZBTB9,DLK2,LOC100294145,HSD17B8,MIR4641,TAPBP,RAB44,PTK7,HLA-DOA,PIM1,MTCH1,XPO5,KCNK16,C6orf132,MAPK14,GLP1R,SRSF3,LINC00951,MIR3934,SYNGAP1,NCR2,HLA-DQB2,RRP36,TREML1,RSPH9,PNPLA1,NUDT3,MLN,ZNF318,TFEB,YIPF3,GRM4,PSMB8-AS1,TREML3P,PRPH2,PSMB9,LINC00336,CLPSL2,LINC01016,MAPK13,TRERF1,GGNBP1,DEF6,ETV7,POLH,SNRPC,TDRG1,PGC,LHFPL5,BTBD9,MIR1275,RING1,FOXP4-AS1,HLA-DPA1,STK38,MIR5004,ZFAND3,RPL10A,SCUBE3,CNPY3,SRF,HLA-DPB2,C6orf222,VEGFA,CLPSL1,C6orf89,LOC100505530,CPNE5,TAF11,MOCS1,CCND3,ITPR3,TTBK1,CRIP3,COL11A2,HLA-DOB,KCNK17,KIF6,CDKN1A,MIR6835,MRPS18A,FANCE,TOMM6,MIR7111,MIR6834,RXRB,BAK1,MED20,FGD2,PACSIN1,MIR5690,HCG25,CUL9,MRPL2,BRPF3,PRICKLE4,MIR3925,LRFN2,HLA-DPB1,TULP1,TAF8,BYSL,LOC100131047,LOC285847,RPS10-NUDT3,CCDC167,DAAM2,ATP6V0CP3,MIR7159,RGL2,LOC100505635,TMEM217,MAD2L1BP,TCP11,PSMB8,CMTR1,KIFC1,LRRC73,MDGA1,GLTSCR1L,UNC5CL,PI16,TREML4,ZBTB22,SLC39A7,DNPH1,TJAP1,GUCA1B,PFDN6,LEMD2,IP6K3,OARD1,TBCC,HMGA1,USP49,TAP2,GTPBP2,PANDAR,ANKS1A,PEX6,TREM1,MEA1,KCNK5,SAYSD1,PPIL1,FKBP5,TREM2,C6orf1,LINC01276,PTCRA,TBC1D22B,MIR4462,KCTD20,ZNF76,TREML5P,MIR6780B,C6orf106,RNF8,PPARD,UQCC2,RPL7L1,ARMC12,MIR1234,PPP2R5D,UHRF1BP1,RPS10	3	gain

1753	CGTE_23	6	121604874	170893019	6q22.31-q22.32	TAB2,LUADT1,SNORA20,LINC01010,SYTL3,PACRG-AS1,HBS1L,LOC103352541,SLC35D3,LOC441178,MTFR2,TTLL2,AGPAT4-IT1,CITED2,LOC101929297,TFB1M,RNF146,TAAR6,TIAM2,LOC101928461,RAET1K,MLLT4-AS1,LOC100129518,CCR6,MIR4465,TAAR9,MIR3163,HDDC2,UTRN,TARID,THBS2,CCDC28A,AGPAT4,LTV1,LOC100507477,OLIG3,MAP3K5,CTGF,ABRACL,ENPP3,VIP,AIG1,MIR3692,RMND1,ARID1B,GINM1,GVQW2,TRMT11,REPS1,HINT3,HMGA1P7,MOXD1,RSPO3,CNKSR3,LOC100289495,LINC01277,ZC2HC1B,ENPP1,ADGRG6,MIR3939,SNORA29,MIR588,GTF2H5,ULBP3,SERINC1,SNORA98,DYNLT1,LINC00271,PPP1R14C,C6orf99,FBXO30,C6orf58,SNORA33,SNX9,SGK1,PHACTR2,RNF217-AS1,OSTCP1,CAHM,KIF25-AS1,TBPL1,RAET1G,NCOA7,SLC18B1,LINC01312,NMBR,LINC00242,TAAR3,ACAT2,LINC01558,MIR3918,EPB41L2,RAET1L,ZDHHC14,HEY2,ARMT1,FNDC1,NOX3,TXLNB,ARFGEF3,LATS1,MAS1,SYNJ2-IT1,ULBP1,GJA1,LPA,IL20RA,RSPH3,LINC01615,MTRF1L,STXBP5,FGFR1OP,LRP11,AIRN,MIR1202,CLDN20,MEAT6,RAB32,TAAR1,VTA1,CCDC170,SMLR1,RPS6KA2-AS1,SLC22A1,TMEM244,RPS6KA2-IT1,PERP,ADAT2,MIR548AJ1,TMEM181,WDR27,FLJ46906,HYMAI,LOC154449,TULP4,SHPRH,MYCT1,ARHGAP18,LOC401286,MRPL18,PSMB1,AKAP7,SYNE1,KIAA0408,CLVS2,LOC643623,PEX3,IFNGR1,SCAF8,MIR5695,TAAR8,WTAP,LOC100132735,RNASET2,SYNJ2,TNFAIP3,SAMD5,ESR1,EZR-AS1,ARG1,PACRG-AS3,PKIB,ECT2L,RGS17,PLAGL1,SNORD100,SUMO4,L3MBTL3,TCP10,MTHFD1L,NUP43,MPC1,FRMD1,SFT2D1,MIR4644,KIF25,FBXO5,SOD2,TAAR2,MIR4466,LINC00602,LOC100507557,LOC102723831,MAP3K4,LOC102724511,PDE7B,RNF217,TCTE3,LINC01624,UST,PNLDC1,MAP7,ULBP2,MIR7641-2,ALDH8A1,LAMA2,BCLAF1,THEMIS,MIR7161,TRDN,LINC01013,TAGAP,ZBTB2,HIVEP2,MIR1913,DACT2,LOC101928140,PDCD2,LOC729603,PDE10A,MIR3662,PACRG-AS2,IPCEF1,VNN1,OR2A4,ZC3H12D,SLC2A12,QKI,IGF2R,LOC153910,LOC100130476,LINC00574,STX11,MED23,VNN3,AKAP12,C6orf118,SLC22A3,KATNA1,ECHDC1,MIR3668,RPS18P9,DLL1,SNORD101,LINC01625,C6orf120,EPM2A,SF3B5,KATNBL1P6,UST-AS1,LINC00326,HECA,LOC100507406,LOC101928661,TPD52L1,FUCA2,CTAGE9,LINC00473,SMOC2,NCOA7-AS1,NKAIN2,PLG,TMEM200A,RAET1E,HSF2,PHACTR2-AS1,PCMT1,SASH1,DKFZp451B082,SOGA3,FABP7,PARK2,MYB,SMPDL3A,SAMD3,TBP,STX7,PACRG,VNN2,LOC101929122,CENPW,ERMARD,LOC101929420,LOC285804,RAET1E-AS1,HGC6.3,PBOV1,AHI1,TCP10L2,GPR31,MIR3145,TAAR5,MLLT4,PEX7,SLC22A2,PTPRK,TBC1D32,NHSL1,LPAL2,LOC101928304,LOC102724053,LOC102723649,TMEM242,IL22RA2,RPS12,SYNE1-AS1,STXBP5-AS1,NHEG1,MIR1273C,UNC93A,SERAC1,LOC101929504,PRR18,HRAT13,OPRM1,PHF10,LOC101928231,TCF21,FAM120B,EZR,EYA4,TCP1,GRM1,T,ADGB,IYD,PPIL4,MIR548H5,RPS6KA2,HEBP2,PLEKHG1	3	gain
1754	CGTE_23	7	2686284	2687323	7p22.3	TTYH3	8	gain
1755	CGTE_23	7	51240048	55499079	7p11.2-p12.1	EGFR,VSTM2A,HPVC1,COBL,LINC01445,LOC100996654,VSTM2A-OT1,ELDR,EGFR-AS1,LANCL2,POM121L12,LINC01446,SEC61G	4	gain
1756	CGTE_23	7	55540645	56174248	7p11.2	SNORA15,CCT6A,PHKG1,SEPT14,ZNF713,PSPH,FKBP9P1,MRPS17,VOPP1,GBAS,SUMF2,CHCHD2	5	gain

1757	CGTE_23	7	84628699	93073084	7q21.3-q21.11	MTERF1,ABCB1,STEAP1,STEAP4,DMTF1,KIAA1324L,LOC102723885,ANKIB1,ZNF804B,GTPBP10,GRM3,AKAP9,LOC101927446,SRI,FZD1,C7orf62,LOC101927497,KRIT1,CFAP69,CDK6,STEAP2-AS1,CYP51A1-AS1,CDK14,DPY19L2P4,TMEM243,TP53TG1,LRRD1,SAMD9L,FAM133DP,CLDN12,RUNDC3B,PEX1,SAMD9,CROT,GATAD1,CYP51A1,VPS50,HEPACAM2,LOC101409256,ADAM22,FAM133B,STEAP2,CALCR,SEMA3D,ABCB4,DBF4,RBM48,MGC16142,SLC25A40	3	gain
1758	CGTE_23	7	101671386	102938414	7q22.1	POLR2J3,NAPEPLD,ORAI2,FBXL13,SPDYE6,POLR2J,ARMC10,DPY19L2P2,LOC100630923,SPDYE2B,SPDYE2,RASA4B,POLR2J2,PMPCB,MIR4467,MIR5090,UPK3BL,RASA4,SH2B2,LOC100289561,LRRC17,RPL19P12,LRWD1,MIR4285,ALKBH4,PRKRIP1,FAM185A,MIR548O,CUX1	3	gain
1759	CGTE_23	8	8175520	10583646	8p23.1	SOX7,MFHAS1,PPP1R3B,MIR124-1,C8orf74,SGK223,MIR4660,MIR597,MIR4286,ERI1,PRSS55,LINC00599,LINCR-0001,MSRA,TNKS,LOC157273,LOC101929128,CLDN23,RP1L1	7	gain
1760	CGTE_23	8	10583649	10588092	8p23.1	SOX7	0	loss
1761	CGTE_23	8	10622893	11420658	8p23.1	MIR598,FAM167A,TDH,FAM167A-AS1,XKR6,MIR1322,PINX1,SLC35G5,LOC101929229,MTMR9,BLK	5	gain
1762	CGTE_23	8	11421394	13425439	8p22-p23.1	NEIL2,MIR5692A1,LOC649352,LOC392196,KIAA1456,FAM66A,FAM90A2P,DEFB136,LOC340357,MIR3926-1,LINC00681,DEFB134,LOC100506990,LONRF1,DEFB109P1,SNORA99,DLC1,MIR3926-2,FDFT1,C8orf49,LOC729732,USP17L7,FAM90A25P,FAM66D,DEFB135,ZNF705D,LINC00208,DEFB130,FAM86B2,FAM86B1,CTSB,C8orf48,BLK,LOC100133267,USP17L2,MIR5692A2,GATA4	8	gain
1763	CGTE_23	8	39463055	39466723	8p11.22	ADAM18	7	gain
1764	CGTE_23	8	117668031	124787860	8q23.3-q24.13	DERL1,SAMD12,SAMD12-AS1,NOV,TBC1D31,MTBP,FAM83A-AS1,UTP23,EXT1,MAL2,C8orf76,ZHX1-C8orf76,FAM91A1,SNTB1,HAS2-AS1,LINC01151,HAS2,RAD21-AS1,TAF2,ZHX1,ZHX2,KLHL38,MRPL13,RAD21,AARD,COL14A1,DSCC1,TNFRSF11B,FBXO32,SLC30A8,ENPP2,LOC105375734,FAM83A,LOC101927543,COLEC10,MED30,MIR3610,ATAD2,MIR4663,ANXA13,EIF3H,WDYHV1,DEPTOR	3	gain
1765	CGTE_23	8	124789390	124811758	8q24.13	FAM91A1	4	gain

1766	CGTE_23	8	124811864	146279543	8q24.21-q24.22	TIGD5,MAF1,ZNF7,MIR6850,SLC45A4,TMEM249,FOXH1,PCAT2,LYNX1,SCRT1,MIR4662B,CYC1,MAFA-AS1,PYCRL,C8orf31,TONSL,ZC3H3,KCNK9,LOC101927845,MIR939,SQLE,AGO2,MIR30D,MIR6893,CCDC26,CCDC166,ZNF623,TMEM65,EEF1D,LOC100288181,LOC101927588,MIR1206,GPAA1,LRRC6,LRRC24,CPSF1,ADCK5,MIR1208,SCRIB,SPATC1,TRIB1,CASC21,TOP1MT,MIR6846,RHPN1-AS1,COL22A1,LY6E,RECQL4,NRBP2,TG,MIR5194,MIR6848,MROH5,LINC01591,NDRG1,MIR1204,LINC00861,HPYR1,ARC,DGAT1,CYHR1,SLURP1,TRAPPC9,KHDRBS3,DENND3,ZFP41,MIR6845,LRRC14,LINC00824,MIR661,MIR1207,CASC11,FER1L6,WDR97,RHPN1,TONSL-AS1,ADGRB1,NDUFB9,MYC,FAM135B,TMED10P1,PHF20L1,ZNF252P-AS1,FAM83H,LINC00051,LOC100133669,ASAP1-IT1,OC90,FAM83H-AS1,MIR4539,LY6K,JRK,FBXL6,CCAT1,FAM49B,CYP11B1,ZNF250,LY6D,ST3GAL1,MIR937,NCRNA00250,LINC00964,BREA2,ZNF517,MIR1205,RNF139-AS1,SLC52A2,PCAT1,CDC42P3,TATDN1,FAM91A1,HHLA1,MTSS1,CASC19,SHARPIN,COMMD5,LOC101927798,KIFC2,FAM84B,PUF60,TSTA3,GPR20,RNF139,MIR4664,SCX,TMEM71,MINCR,EXOSC4,MIR6847,GLI4,MIR4472-1,MIR7112,WISP1,ASAP1-IT2,CYP11B2,ZFAT,MROH6,ADCY8,MIR30B,MFSD3,GSDMD,CCAT2,MAPK15,MIR3686,KCNQ3,PTK2,OPLAH,ZNF34,GPIHBP1,EFR3A,MIR6849,TSNARE1,ZNF572,MIR6844,LINC01300,PLEC,PVT1,POU5F1B,GML,FER1L6-AS1,FER1L6-AS2,RPL8,CHRAC1,ARHGAP39,ZNF696,MIR1302-7,SLC39A4,LYPD2,GRINA,ZNF251,GPT,EPPK1,ASAP1,SLA,MAFA,TMEM75,TRMT12,VPS28,HGH1,ZNF16,PSCA,ZNF707,LOC101927915,MIR4662A,PRNCR1,PTP4A3,CASC8,MIR7848,ZNF252P,LY6H,ZFAT-AS1,GSDMC,LOC101927822,PPP1R16A,LINC00977,NAPRT,C8orf82,C8orf33,HSF1,PARP10,KIAA0196,NSMCE2,BOP1,LOC101927657,MROH1,THEM6	3	gain
1767	CGTE_23	9	34379549	38621271	9p13.2-p13.1	ALDH1B1,FRMPD1,EXOSC3,STOML2,FANCG,RUSC2,ZCCHC7,CA9,FAM95C,PAX5,POLR1E,DCAF10,CNTFR-AS1,SHB,FAM219A,CCDC107,RNF38,FAM221B,TLN1,GNE,CNTFR,C9orf131,SPAG8,CCL27,SLC25A51,UNC13B,DNAJB5,ARHGEF39,OR13J1,SIGMAR1,FAM166B,RPP25L,DNAJB5-AS1,HINT2,CREB3,ENHO,ATP8B5P,MIR4476,TRMT10B,NPR2,MIR4540,FAM205A,MIR4475,TMEM8B,VCP,MSMP,LINC00950,IL11RA,CD72,DNAI1,IGFBPL1,RGP1,MIR6852,ANKRD18A,TESK1,CCL19,LINC00961,ARID3C,GALT,TOMM5,HRCT1,FAM201A,SIT1,MIR4667,FAM205C,CCIN,FAM214B,MIR6853,FAM205BP,CLTA,ZBTB5,C9orf24,FBXO10,OR2S2,PHF24,CCL21,EBLN3,GRHPR,TPM2,GBA2,PIGO,MELK,RMRP,GLIPR2,DCTN3,RECK	3	gain

1768	CGTE_23	9	117138194	141016420	9q34.13-q34.3	MIR4672,MIR3689F,C9orf91,C9orf173-AS1,OR1N1,MAN1B1-AS1,PPP6C,OR1J2,MIR3689A,SURF2,TRAF1,CRB2,NALT1,URM1,ST6GALNAC6,LOC101448202,PSMB7,COL5A1,LRSAM1,C9orf50,POMT1,MIR4292,C9orf142,MIR6877,USP20,LOC101928775,SNORA70C,TNC,DOLK,MIR6856,CIZ1,FAM166A,MIR147A,NRON,SLC2A8,COQ4,FCN2,SNORD24,PRRC2B,GBGT1,TUBB4B,MED27,STRBP,TLR4,PRRX2-AS1,GOLGA2,PSMD5-AS1,ABL1,NTNG2,SPTAN1,LOC100128593,AK1,GLT6D1,RALGPS1,CACFD1,PIP5KL1,ARPC5L,SNORA17B,RPL35,C9orf9,PPP1R26,MIR3689B,NOXA1,SAPCD2,MIR3621,CCDC183,LINC01502,PTRH1,NOTCH1,BRD3,TTC16,GPR21,UBAC1,MVB12B,CACNA1B,SLC2A6,LINC00963,NPDC1,FAM163B,SNORD141B,LCN2,ABCA2,NUP188,ABO,CAMSAP1,TRUB2,MIR6855,NCS1,OR1L4,GAPVD1,SNORD36A,C9orf173,C9orf62,ZNF79,PTGES2-AS1,MIR126,PHYHD1,LOC100272217,MIR3960,GPR107,LHX3,EHMT1-IT1,MED22,LOC100133077,LHX6,PBX3,MIR7114,SETX,DBH-AS1,FAM69B,SCAI,LCN15,RABEPK,LINC01613,SARDH,QSOX2,PAPPA,TMEM203,VAV2,MIR181B2,DENND1A,CDK5RAP2,C9orf163,RABL6,PTGDS,NSMF,OR1L8,DOLPP1,FUT7,LOC100288842,PDCL,RAPGEF1,CCDC183-AS1,SNORD62A,MIR199B,C9orf116,SET,ZBTB34,LOC105376331,FAM78A,LOC101929116,DPM2,ASTN2-AS1,SDCCAG3,OR1L3,SNORA65,SLC34A3,KCNT1,ENTPD2,SWI5,TRIM32,RNU6ATAC,PPP2R4,LCN1,GFI1B,PAPPA-AS1,SURF6,MAN1B1,MIR3911,LCNL1,PKN3,MORN5,MRPL41,PSMD5,MAPKAP1,ODF2,DPH7,NTMT1,AIF1L,C5,FIBCD1,GGTA1P,LCN8,ARRDC1-AS1,ZMYND19,CERCAM,MIR3689D2,SLC27A4,MIR181A2HG,ZBTB26,OBP2B,MIR1268A,MEGF9,MIR600HG,ASS1,GPSM1,MIR219A2,RNF224,SLC25A25,MIR219B,C9orf106,TSC1,PRDM12,ASTN2,C9orf69,LHX2,PTGES,AK8,WDR38,MIR181A2,ENTPD8,RALGDS,FAM129B,AKNA,TBC1D13,IER5L,FPGS,EHMT1,LOC101928748,TNFSF15,FBXW2,INPP5E,PHPT1,LOC101929331,UCK1,LOC101928786,MIR2861,ANAPC2,NDOR1,TOR4A,LMX1B,RC3H2,SH3GLB2,SNORD62B,GSN,WDR34,GRIN1,LINC00474,SURF4,LOC51145,FNBP1,OR1L1,OR1Q1,DKFZP434A062,ZBTB6,RPL12,LRRC8A,LAMC3,EXD3,LINC01503,LCN10,GLE1,MIR601,ASB6,ENDOG,TMEM8C,STKLD1,NAIF1,PHF19,GARNL3,MIR3689C,PMPCA,ADAMTS13,CFAP77,ARRDC1,PAEP,ENG,MIR6722,CDK9,CFAP157,PTGS1,MIR3689E,DBH,CELP,MIR4674,CCBL1,LCN6,SOHLH1,NR5A1,NUP214,GOLGA1,TTLL11,DNM1,TMEM210,CARD9,REXO4,FBXW5,PRRX2,FAM73B,STXBP1,EDF1,ADAMTSL2,NR6A1,TMEM141,LOC401557,DFNB31,MIR600,MIR3689D1,TRAF2,BARHL1,OR1J4,QRFP,EGFL7,EXOSC2,PLPP7,LOC101928797,ZER1,OR5C1,TOR1A,NDUFA8,CYSRT1,CRAT,MIR3154,SNORA17A,ST6GALNAC4,MIR4479,MIR7150,SEC16A,TTF1,MAMDC4,PTGES2,LOC100129034,BRINP1,OR1L6,RBM18,RPL7A,FCN1,PPP1R26-AS1,MIR4478,NELFB,SNAPC4,GTF3C5,DEC1,RAB14,TPRN,NEK6,C9orf139,HMCN2,MIR4673,OLFM1,NACC2,TOR2A,C9orf78,MIR602,DAB2IP,ATP6V1G1,DDX31,MIR548AW,SSNA1,OR1K1,OR1B1,MRPS2,WDR5,OR1J1,SNORD90,RABGAP1,C9orf114,LOC101928525,SH2D3C,SNORD141A,PNPLA7,LRRC26,GTF3C4,CLIC3,LOC100505478,CNTRL,C8G,NRARP,CEL,LCN9,SURF1,ZBTB43,MIR4669,LOC100506100,DPP7,SLC25A25-AS1,STOM,ZDHHC12,LINC00094,GSN-AS1,OLFML2A,DNLZ,OBP2A,C9orf16,FAM102A,TNFSF8,SNHG7,FUBP3,LCN12,HSPA5,SNORD36C,ANGPTL2,SNORD36B,RNF208,C9orf172,OR1N2,UAP1L1,AGPAT2,MRRF,TOR1B,RXRA	3	gain
1769	CGTE_23	10	22292106	23003281	10p12.31-p12.2	DNAJC1,COMMD3,SPAG6,PIP4K2A,BMI1,LOC100130992,EBLN1,LOC100499489,COMMD3-BMI1	4	gain

1770	CGTE_23	10	23220846	53822660	10q11.1-q21.1	C10orf142,C10orf71,MSMB,ARHGAP22,LINC00619,LOC100129055,HSD17B7P2,ASAH2,MIR604,PARD3,MIR605,FAM35BP,FAM25C,ANKRD30A,KIAA1217,FZD8,FAM170B,ZNF37A,MSRB2,FAM170B-AS1,ZNF485,LOC101929431,RASSF4,KIF5B,MAPK8,LINC01552,ACBD5,MTRNR2L7,SNORA86,RSU1P2,ANXA8L1,SEPT7P9,LINC00838,MKX,ABI1,ZFAND4,LINC00202-2,ARHGAP21,C10orf128,PTPN20,LINC01264,BAMBI,ZNF32-AS2,C10orf25,FAM35DP,C10orf71-AS1,ANKRD30BP3,ZEB1-AS1,ZNF32-AS3,MIR3156-1,CSGALNACT2,NPY4R,PARGP1,AGAP9,LINC00840,LINC00839,LINC00836,ZNF37BP,VSTM4,OR13A1,MAP3K8,ALOX5,FAM21A,RAB18,WDFY4,LINC00837,CTSLP2,MIR603,HNRNPA3P1,LYZL1,ZEB1,FRMPD2B,ZNF488,CXCL12,TMEM72-AS1,LINC01518,PARD3-AS1,MYO3A,RBP3,FAM25G,LINC00841,MIR5586,CSTF2T,ZNF239,CCNYL2,SYT15,ZNF438,ARMC4,ACTR3BP5,BMS1P6,LINC00999,ZNF33A,LINC00202-1,GJD4,C10orf126,ANTXRLP1,A1CF,BMS1P5,GPR158-AS1,FRMPD2,MIR4294,CH17-360D5.1,ZNF32-AS1,ANTXRL,PGBD3,AGAP6,FAM21EP,EPC1,GOLGA2P6,ZNF487,BMS1,ERCC6-PGBD3,AGAP4,ASAH2B,SLC18A3,AGAP12P,HNRNPF,ZNF33B,TIMM23B,MARCH8,FAM21C,MIR1254-2,ARHGAP12,ERCC6,GPRIN2,GDF10,MIR4683,MKX-AS1,MIR5100,NRP1,LRRC37A6P,ANXA8,TIMM23,FAM25BP,OGDHL,AGAP7P,ZNF25,SVIL,MIR938,C10orf67,HNRNPA1P33,LRRC18,ARMC3,ANKRD26,SNORD130,LOC101929073,GDF2,SGMS1-AS1,MTPAP,APBB1IP,LOC101929279,CHAT,YME1L1,MIR7162,SGMS1,GLUD1P7,MIR3611,ZNF33BP1,OTUD1,LYZL2,LOC102031319,MASTL,WAC-AS1,FXYD4,TMEM72,C10orf53,MIR8086,CCNY,NCOA4,PARG,ITGB1,RET,ZNF22,GPR158,MPP7,LOC441666,LOC102724323,LOC102724719,SVIL-AS1,DRGX,SVILP1,GAD2,KIAA1462,ZNF32,PDSS1,LINC01516,LINC00993,PTF1A,LINC01517,PRKG1,C10orf10,CUL2,LINC00842,THNSL1,WAC,PRTFDC1,ENKUR,RASGEF1A,CCDC7,PTCHD3,LINC00264,ZNF248,PCAT5,CREM	3	gain
1771	CGTE_23	10	74033915	76360290	10q22.2-q22.1	SEC24C,FAM149B1,PLAU,DNAJB12,MIR4676,PLA2G12B,GLUD1P3,PPP3CB-AS1,ZSWIM8-AS1,ANXA7,USP54,SYNPO2L,NDST2,AP3M1,CFAP70,DNAJC9-AS1,DDIT4,OIT3,ADK,MCU,DNAJC9,CHCHD1,P4HA1,CAMK2G,MICU1,FUT11,ECD,PPP3CB,VCL,C10orf55,MYOZ1,ZSWIM8,MSS51,BMS1P4,LOC102723439,AGAP5,NUDT13,MRPS16	7	gain
1772	CGTE_23	10	76429765	82394197	10q22.3-q23.1	RPS24,DYDC2,NUTM2B,FAM213A,LINC00595,ZNF503-AS1,LINC00857,LINC00856,DLG5,ZNF503,ADK,EIF5AL1,LOC642361,DYDC1,SFTPA1,ANXA11,KCNMA1,TMEM254-AS1,MBL1P,VDAC2,NUTM2B-AS1,SH2D4B,LOC101929574,BEND3P3,BMS1P21,DUSP13,PLAC9,LOC105378367,LOC101929234,KAT6B,LOC102723703,TMEM254,TSPAN14,DUPD1,C10orf11,KCNMA1-AS2,ZCCHC24,ZNF503-AS2,KCNMA1-AS1,SFTPA2,SFTPD,MAT1A,DLG5-AS1,ZMIZ1-AS1,POLR3A,KCNMA1-AS3,SAMD8,PPIF,COMTD1,MIR606,ZMIZ1	3	gain
1773	CGTE_23	10	127408366	134039398	10q26.3-q26.13	FOXI2,STK32C,FANK1-AS1,ADAM12,GLRX3,TCERG1L-AS1,UROS,LINC01164,LINC00959,FAM196A,DPYSL4,C10orf90,PTPRE,EDRF1,MGMT,BCCIP,MIR4484,JAKMIP3,FANK1,MMP21,MIR4297,LINC00601,TCERG1L,BNIP3,EDRF1-AS1,EBF3,CTAGE7P,MIR378C,MKI67,CLRN3,PPP2R2D,LINC01163,DHX32,DOCK1,NPS	3	gain
1774	CGTE_23	10	134040262	134261569	10q26.3	STK32C,LRRC27,C10orf91,PWWP2B	4	gain

1775	CGTE_23	11	32949525	33309089	11p13	TCP11L1,CSTF3-AS1,QSER1,HIPK3,DEPDC7,CSTF3,LINC00294	8	gain
1776	CGTE_23	11	33350033	34905099	11p13	HIPK3,CAT,C11orf91,ABTB2,LMO2,NAT10,FBXO3-AS1,APIP,KIAA1549L,FBXO3,ELF5,CD59,CAPRIN1,EHF	3	gain
1777	CGTE_23	11	34909672	34918583	11p13	APIP	5	gain
1778	CGTE_23	11	34937810	35496326	11p13	LOC100507144,APIP,PDHX,MIR1343,PAMR1,SLC1A2,CD44	6	gain
1779	CGTE_23	11	45944334	47159285	11p11.2	CREB3L1,LRP4,CKAP5,MDK,SNORD67,MIR4688,HARBI1,ZNF408,ARHGAP1,MIR5582,DGKZ,GYLTL1B,MIR3160-1,C11orf49,F2,CHRM4,LRP4-AS1,ATG13,MIR3160-2,PHF21A,AMBRA1,LOC101928894	3	gain
1780	CGTE_23	11	65088168	65429688	11q13.1	CDC42EP2,NEAT1,SCYL1,MIR612,EHBP1L1,MIR4690,LTBP3,KCNK7,FRMD8,SLC25A45,SIPA1,FAM89B,SSSCA1,SSSCA1-AS1,TIGD3,PCNXL3,DPF2,MAP3K11,MIR4489,MALAT1,RELA	4	gain
1781	CGTE_23	11	65430185	72019847	11q13.1-q13.2	UNC93B1,INPPL1,IL18BP,SF3B2,AIP,LOC100133315,TMEM151A,KLC2,EFEMP2,SHANK2-AS1,CARNS1,CCS,MIR3164,MIR3664,C11orf80,FLJ42102,SLC29A2,EIF1AD,MIR4691,ALDH3B1,CLPB,OVOL1-AS1,MIR6752,FOLR2,LOC101928443,CTSW,PPP6R3,CLCF1,PPP1CA,BANF1,TPCN2,ALDH3B2,DPP3,DEFB108B,LRTOMT,FAM86C1,LOC100130987,MYEOV,TMEM134,ZDHHC24,PHOX2A,SYT12,NUDT8,KAT5,TSGA10IP,MIR3163,CCDC85B,PITPNM1,YIF1A,NDUFS8,CTSF,DHCR7,TBC1D10C,ANAPC15,GSTP1,PC,BBS1,MIR7113,RIN1,NDUFV1,TCIRG1,FGF3,ORAOV1,OVOL1,FIBP,CST6,KRTAP5-10,SPTBN2,SHANK2,RBM14-RBM4,RBM4B,SSH3,FAM86C2P,DOC2GP,MTL5,LINC01488,ALG1L9P,C11orf68,ACTN3,MIR6754,MIR3165,MRPL11,GAL3ST3,ANO1-AS2,IGHMBP2,ZNF705E,NPAS4,C11orf24,MRPL21,RCE1,LOC338694,KRTAP5-7,B4GAT1,RHOD,TBX10,LOC100129216,MIR6860,SHANK2-AS3,NADSYN1,RNF121,LOC100128494,MRGPRD,ADRBK1,SNX32,RBM14,CORO1B,RPS6KB2,CD248,CCND1,FGF4,CABP2,C11orf86,LOC101928069,MIR1234,FOLR3,ACY3,PTPRCAP,CPT1A,NUMA1,MRGPRF-AS1,FGF19,CABP4,PELI3,FOSL1,PPFIA1,LAMTOR1,MUS81,CDK2AP2,CFL1,ANKRD13D,KMT5B,RELA,FADD,MRGPRF,DRAP1,BRMS1,CNIH2,CATSPER1,KRTAP5-9,GPR152,KRTAP5-11,GAL,LRFN4,ANO1,FOLR1,RBM4,POLD4,MIR6753,CTTN,C11orf72,KDM2A,CCDC87,RAD9A,PACS1,AP5B1,KRTAP5-8,LRP5,RNASEH2C,SART1,RAB1B,MIR548K,CHKA	3	gain
1782	CGTE_23	11	72028044	77622131	11q13.4-q13.5	LOC283214,ACER3,NEU3,EMSY,B3GNT6,GUCY2EP,P4HA3,WNT11,STARD10,AAMDC,MRPL48,TPBGL,LOC101928580,MIR4459,LOC646029,KCNE3,DGAT2,LRRC32,CLNS1A,LOC100506127,RNF169,CHRDL2,UCP3,DNAJB13,RPS3,LIPT2,MIR4696,FCHSD2,LOC101928837,OR2AT4,PDE2A,SPCS2,MIR326,TSKU,MYO7A,RSF1,C2CD3,CLPB,XRRA1,RELT,UVRAG,PLEKHB1,SNORD15A,P2RY6,GDPD4,MIR139,P2RY2,SLCO2B1,CAPN5,INTS4,RAB6A,POLD3,ARRB1,ARAP1,MOGAT2,MIR4692,ARHGEF17,SNORD15B,GDPD5,AQP11,OMP,PAK1,MAP6,ATG16L2,KLHL35,FAM168A,PPME1,SERPINH1,PAAF1,PRKRIR,LINC01537,PGM2L1,COA4,UCP2	4	gain
1783	CGTE_23	11	77671228	78204281	11q14.1	ALG8,LOC101928865,KCTD21-AS1,GAB2,NARS2,KCTD14,INTS4,NDUFC2-KCTD14,KCTD21,USP35,NDUFC2,THRSP	4	gain
1784	CGTE_23	11	78523090	83344554	11q14.1	FAM181B,ANKRD42,PCF11,RAB30-AS1,LOC101928944,SNORA70E,PRCP,DDIAS,MIR4300HG,DLG2,CCDC90B,MIR708,MIR5579,TENM4,MIR4300,RAB30	3	gain

1785	CGTE_23	11	131530086	134856681	11q25	NTM-IT,VPS26B,MIR4697,THYN1,NCAPD3,B3GAT1,OPCML,SPATA19,GLB1L2,IGSF9B,NTM,LOC283177,ACAD8,JAM3,LOC100128239,GLB1L3,MIR4697HG,NTM-AS1,LOC646522	1	loss
1786	CGTE_23	12	24970877	27944819	12p11.23-p12.1	C12orf71,RASSF8,ASUN,LMNTD1,C12orf77,MRPS35,PPFIBP1,REP15,KLHL42,LYRM5,CASC1,KRAS,MED21,ITPR2,LRMP,MIR4302,STK38L,FGFR1OP2,MANSC4,ARNTL2-AS1,SSPN,TM7SF3,RASSF8-AS1,ARNTL2,BCAT1,BHLHE41,SMCO2	8	gain
1787	CGTE_23	12	49688945	49689674	12q13.12	PRPH,LOC101927267	7	gain
1788	CGTE_23	13	38934079	39262135	13q13.3	UFM1,LINC00366,LINC00437,FREM2	4	gain
1789	CGTE_23	13	61013701	61018931	13q21.2	TDRD3	8	gain
1790	CGTE_23	13	73547695	73650015	13q22.1	PIBF1,KLF5	4	gain
1791	CGTE_23	13	74269658	78218459	13q22.1-q22.3	COMMD6,KLF12,SCEL,LMO7,LINC00347,MYCBP2-AS1,FBXL3,LMO7-AS1,LMO7DN-IT1,MYCBP2,BTF3P11,KCTD12,LINC01078,SCEL-AS1,CLN5,LMO7DN,TBC1D4,CTAGE11P,UCHL3,IRG1,LINC00381	3	gain
1792	CGTE_23	13	78235542	78272705	13q22.3	SLAIN1,MIR3665,LOC100129307	0	loss
1793	CGTE_23	13	78273082	103326982	13q31.3-q32.2	OXGR1,CLYBL,SLC15A1,TMTC4,PCCA,HS6ST3,LOC105370333,STK24,LINC01038,RNF219,UBAC2,EDNRB-AS1,FGF14-AS1,RNF113B,LOC101927284,PCCA-AS1,GPC6,LINC00433,UBAC2-AS1,DZIP1,GGACT,NDFIP2,TGDS,ITGBL1,DOCK9-AS2,DNAJC3-AS1,RBM26-AS1,LINC00456,IPO5,NDFIP2-AS1,MIR3170,GPC6-AS1,TPP2,FGF14-IT1,SOX21-AS1,CLYBL-AS2,MIR92A1,SOX21,POU4F1,LINC00363,MIR4306,LINC00379,LINC00449,LINC00375,LINC00557,LINC00331,SLITRK1,MIR20A,MIR19B1,MIR18A,NALCN,DOCK9,LOC101927248,RBM26,CLDN10-AS1,MIR4501,LINC01047,UGGT2,FKSG29,MIR4705,LINC00430,ZIC5,LINC00380,LINC00333,RNF219-AS1,SPRY2,SLAIN1,LINC00554,LINC00411,LINC01039,LINC01049,CLYBL-AS1,MIR17HG,LINC00397,DOCK9-AS1,GPC5-AS1,LOC105370306,DNAJC3,MIR2681,MIR548AN,DCT,FARP1,LINC00440,MIR622,GPC5,NALCN-AS1,LINC00353,SNORA107,LINC00359,MIR17,GPC5-AS2,LINC00377,MIR4500HG,LINC00382,MBNL2,LINC00564,LINC01068,FGF14,ZIC2,LINC01069,GPR180,RAP2A,LINC00559,MIR4500,GPR18,LINC00446,LINC01232,SLITRK6,SLITRK5,GPR183,EDNRB,LOC101927437,TM9SF2,LINC01080,LINC01040,FGF14-AS2,LINC00410,CLDN10,ABCC4,MIR623,GPC6-AS2,LINC00351,MIR19A	3	gain
1794	CGTE_23	13	103445606	109614084	13q33.1-q33.2	LINC00343,SLC10A2,ERCC5,FAM155A,LINC00443,DAOA-AS1,KDELC1,LIG4,TNFSF13B,DAOA,BIVM,ABHD13,BIVM-ERCC5,FAM155A-IT1,LINC00460,MIR1267,LINC00551,LINC01309,EFNB2,ARGLU1,METTL21EP,MYO16,MIR548AS	3	gain
1795	CGTE_23	14	50121421	50154960	14q21.3	POLE2	1	loss
1796	CGTE_23	14	54907906	54946497	14q22.2	CNIH1,GMFB	5	gain
1797	CGTE_23	15	56927308	56959034	15q21.3	ZNF280D	3	gain
1798	CGTE_23	16	704987	706624	16p13.3	WDR90	7	gain

1799	CGTE_23	16	12618487	15932334	16p13.11-p13.12	MIR3179-3,MIR6511A2,ERCC4,MIR6511A1,MIR3670-2,MIR3180-4,PLA2G10,MIR6511A3,MIR3180-3,C16orf45,PKD1P6,PDXDC1,BFAR,MIR193B,MIR3180-2,MIR6506,MIR484,CPPED1,MIR365A,NTAN1,NPIPA5,RRN3,MIR3670-4,NPIPA3,LOC100288162,NDE1,MIR6511B2,MIR3179-2,NPIPA1,KIAA0430,MIR4718,NPIPA2,MIR6511B1,MIR193BHG,LOC105447648,MIR3670-1,NOMO1,LOC101927348,SHISA9,LOC101927311,SNX29,MIR6770-1,LOC100505915,ABCC6P2,MPV17L,MIR6770-2,MIR3179-4,MYH11,PARN,MIR6770-3,MIR6511A4,MKL2,MIR3180-1,MIR3670-3,MIR3179-1	4	gain
1800	CGTE_23	16	15961272	16292001	16p13.11	ABCC6,ABCC1,FOPNL	3	gain
1801	CGTE_23	16	30543958	30567352	16p11.2	ZNF764,ZNF747	6	gain
1802	CGTE_23	17	39182910	39622249	17q21.2	KRT38,KRTAP1-3,KRTAP2-1,KRTAP4-12,KRTAP4-9,KRTAP29-1,KRTAP4-2,KRTAP9-2,KRTAP9-7,KRTAP4-1,KRTAP4-7,KRT33A,KRT31,KRTAP2-3,KRTAP9-1,KRTAP9-4,KRTAP4-8,KRTAP9-6,KRTAP4-11,KRTAP4-6,KRTAP4-3,KRTAP2-2,KRT34,KRTAP17-1,KRTAP1-4,KRT37,KRTAP1-1,KRTAP4-4,LOC100505782,KRTAP16-1,KRTAP1-5,KRT32,KRTAP9-3,KRTAP9-9,KRTAP4-5,KRT33B,KRTAP9-8,KRTAP2-4	3	gain
1803	CGTE_23	18	2784713	5238441	18p11.32-p11.31	SMCHD1,MYL12A,DLGAP1,C18orf42,DLGAP1-AS2,LOC104968399,LINC00526,LINC00667,EMILIN2,MIR6718,DLGAP1-AS1,DLGAP1-AS5,MYL12B,DLGAP1-AS4,GAPLINC,TGIF1,MYOM1,DLGAP1-AS3,LPIN2,LOC727896	3	gain
1804	CGTE_23	19	5790076	8140501	19p13.3-p13.2	SLC25A41,LOC101928844,CCL25,CAMSAP3,CLEC4GP1,SH2D3A,TGFBR3L,MIR6885,CAPS,KHSRP,DENND1C,PRR36,MIR6791,MCOLN1,TUBB4A,TNFSF9,MIR6790,NDUFA11,PNPLA6,CD209,LYPLA2P2,MIR3940,VAV1,PCP2,MBD3L3,RETN,ACER1,ZNF557,ACSBG2,LOC100128573,INSR,FLJ25758,TIMM44,C3,GPR108,PET100,MIR6792,ALKBH7,MBD3L5,CTXN1,STXBP2,PSPN,TRAPPC5,CD70,CLEC4G,VMAC,CLEC4M,SLC25A23,ADGRE4P,CRB3,C19orf45,ELAVL1,XAB2,SNAPC2,FCER2,TNFSF14,FUT6,MBD3L2,ADGRE1,CLPP,MBD3L4,ARHGEF18,MLLT1,RANBP3,LOC100128568,LRRC8E,FBN3,DUS3L,MAP2K7,MCEMP1,ZNF358,RFX2,PEX11G,EVI5L,GTF2F1,FUT3,TRIP10,NRTN,FUT5	1	loss
1805	CGTE_23	19	16130521	18174873	19p13.11-p13.12	FAM129C,HSH2D,CPAMD8,B3GNT3,SNORA68,SLC27A1,BST2,AP1M1,RAB8A,ANKLE1,FAM32A,TMEM38A,LINC00661,LINC00905,OCEL1,MED26,MYO9B,COLGALT1,PLVAP,KLF2,IL12RB1,F2RL3,ANO8,CIB3,MRPL34,MVB12A,MAP1S,USHBP1,TPM4,KCNN1,SIN3B,BABAM1,USE1,NXNL1,PGLS,TMEM221,BISPR,ARRDC2,RPL18A,C19orf44,FCHO1,SMIM7,JAK3,SLC5A5,NR2F6,SLC35E1,ABHD8,EPS15L1,INSL3,UNC13A,CALR3,HAUS8,GTPBP3,CCDC124,NWD1,CHERP,DDA1	4	gain
1806	CGTE_23	19	18177353	19674483	19p13.11	CRLF1,TMEM59L,CERS1,MEF2BNB-MEF2B,MEF2B,FKBP8,SUGP2,UBA52,TM6SF2,ISYNA1,LSM4,MIR640,SLC25A42,GDF15,LOC729966,KXD1,TSSK6,C19orf60,MIR3189,MIR3188,HAPLN4,NCAN,DDX49,RFXANK,UPF1,PIK3R2,BORCS8,KLHL26,IFI30,COPE,IL12RB1,ARMC6,LRRC25,MPV17L2,HOMER3,COMP,PGPEP1,RAB3A,SSBP4,YJEFN3,NDUFA13,SUGP1,KIAA1683,TMEM161A,CILP2,CRTC1,PDE4C,GDF1,PBX4,GATAD2A,ELL,MAST3,JUND,NR2C2AP,MAU2	3	gain
1807	CGTE_23	20	32691357	32699896	20q11.22	EIF2S2	13	gain
1808	CGTE_23	20	32848208	33244993	20q11.22	DYNLRB1,PIGU,ASIP,AHCY,MAP1LC3A,MIR644A,ITCH	3	gain
1809	CGTE_23	20	33244998	33297349	20q11.22	PIGU,TP53INP2	8	gain

1810	CGTE_23	20	33297750	34295933	20q11.22	PROCR,ERGIC3,GGT7,C20orf173,MIR499B,SPAG4,EDEM2,CEP250,MYH7B,UQCC1,NFS1,NCOA6,MMP24,GSS,FER1L4,GDF5,MMP24-AS1,MIR499A,MIR1289-1,TP53INP2,FAM83C-AS1,ROMO1,TRPC4AP,HMGB3P1,ACSS2,FAM83C,RBM12,EIF6,RBM39,CPNE1	3	gain
1811	CGTE_23	20	34297028	34301081	20q11.22	RBM39	6	gain
1812	CGTE_23	20	42310237	44517637	20q13.12	RBPJL,EPPIN-WFDC6,MIR6812,SLPI,KCNS1,DNTTIP1,DBNDD2,WISP2,FITM2,TOX2,HNF4A,RIMS4,MYBL2,TTPAL,OSER1-AS1,GTSF1L,MIR3617,STK4,SNX21,STK4-AS1,SDC4,KCNK15-AS1,PKIG,SPINT3,R3HDML,LINC01430,TNNC2,WFDC2,WFDC3,LINC01260,YWHAB,SEMG1,SERINC3,NEURL2,WFDC9,WFDC10A,ZSWIM1,HNF4A-AS1,PABPC1L,SEMG2,EPPIN,TP53TG5,SYS1-DBNDD2,ZSWIM3,WFDC13,WFDC11,WFDC12,GDAP1L1,JPH2,MIR3646,TOMM34,SPATA25,MATN4,KCNK15,PI3,PIGT,OSER1,ACOT8,WFDC5,LINC01620,ADA,SPINT4,UBE2C,SYS1,WFDC6,WFDC10B,WFDC8	3	gain
1813	CGTE_23	20	44518858	44520063	20q13.12	NEURL2,CTSA	5	gain
1814	CGTE_23	20	44520257	56140920	20q13.12-q13.13	CTSA,MIR3194,MMP9,TSHZ2,MIR1302-5,LINC01522,LINC01273,PTGIS,CTCFL,LINC01441,ZFAS1,RBM38,BCAS4,OCSTAMP,PREX1,NFATC2,NCOA3,LOC101927770,RNF114,MIR4325,SLC35C2,PARD6B,LINC01272,PTPN1,KCNG1,BMP7,ZNF217,FAM210B,CSE1L,SPO11,LINC01440,ATP9A,NCOA5,SLC2A10,TFAP2C,SLC9A8,SPATA2,ZNFX1,LINC01429,MC3R,CD40,ZNF334,SULF2,ZNF335,PFDN4,TMEM189-UBE2V1,TP53RK,FAM209B,LINC01271,CEBPB-AS1,SUMO1P1,MIR3616,MOCS3,DOK5,PLTP,LOC100131496,LINC01524,TMEM189,SNORD12,ARFGEF2,PCIF1,CASS4,BCAS1,ADNP,MIR4756,BMP7-AS1,LINC01270,DDX27,FAM65C,RTFDC1,SNORD12C,CSE1L-AS1,LINC00494,SALL4,STAU1,LOC100506175,ELMO2,UBE2V1,CDH22,SNAI1,ADNP-AS1,EYA2,CBLN4,TRERNA1,SLC13A3,CSTF1,B4GALT5,CYP24A1,LINC01523,CEBPB,GCNT7,FAM209A,PCK1,MKRN7P,ZMYND8,ZFP64,AURKA,DPM1,ZNF663P,KCNB1,SNORD12B,RAE1,MTRNR2L3,SLC12A5,MIR645	3	gain
1815	CGTE_23	20	58452978	58461095	20q13.33	SYCP2	4	gain
1816	CGTE_23	20	58461773	58490389	20q13.33	SYCP2	3	gain
1817	CGTE_23	20	58490468	58494275	20q13.33	SYCP2	7	gain
1818	CGTE_23	20	60897209	60898961	20q13.33	LAMA5	7	gain
1819	CGTE_23	20	60899130	60988988	20q13.33	RBBP8NL,LAMA5,CABLES2,RPS21,LAMA5-AS1,MIR4758	3	gain
1820	CGTE_23	20	60988989	60991114	20q13.33	RBBP8NL	7	gain
1821	CGTE_23	20	61492429	61512624	20q13.33	DIDO1,TCFL5	7	gain
1822	CGTE_23	20	62492901	62496853	20q13.33	TPD52L2,ABHD16B	67	gain
1823	CGTE_23	21	32253187	34258369	21q22.11	PAXBP1,TIAM1,C21orf62-AS1,PAXBP1-AS1,MIS18A,C21orf59,LINC00159,URB1,C21orf62,SYNJ1,SOD1,EVA1C,HUNK,SNORA80A,KRTAP11-1,MRAP,SCAF4,KRTAP19-8,TCP10L,URB1-AS1	1	loss
1824	CGTE_23	21	43187006	44074041	21q22.3	SNORA91,C2CD2,SLC37A1,LOC101928233,PRDM15,RIPK4,UMODL1-AS1,ZNF295-AS1,ZBTB21,UMODL1,PDE9A,ABCG1,TMPRSS3,RSPH1,TFF1,UBASH3A,TFF3,TFF2	1	loss
1825	CGTE_23	22	19511121	19702380	22q11.21	LINC00895,SEPT5,CLDN5	3	gain
1826	CGTE_23	22	21983160	21984303	22q11.21	YDJC	9	gain
1827	CGTE_23	X	125299442	125686552	Xq25	DCAF12L2,DCAF12L1	3	gain
1828	CGTE_23	Y	15467796	15481254	Yq11.221	UTY	0	loss

1829	CGTE_23	Y	22741292	23545869	Yq11.223	RPS4Y2,EIF1AY,PRORY	0	loss
1830	CGTE_24	1	861266	866507	1p36.33	SAMD11	4	gain
1831	CGTE_24	1	871064	987468	1p36.33	KLHL17,NOC2L,PERM1,ISG15,SAMD11,PLEKHN1,HES4,AGRN	0	loss
1832	CGTE_24	1	988720	990505	1p36.33	AGRN	9	gain
1833	CGTE_24	1	1007107	1564067	1p36.33	LOC102724312,SSU72,VWA1,ATAD3C,TMEM88B,MIR6727,TNFRSF4,FAM132A,PUSL1,CCNL2,DVL1,TTLL10,MIB2,SDF4,UBE2J2,TMEM240,ACAP3,MIR6808,ANKRD65,CPTP,TNFRSF18,RNF223,MIR429,ATAD3A,SCNN1D,MIR200B,B3GALT6,AURKAIP1,MXRA8,TAS1R3,LINC01342,LOC148413,C1orf159,ATAD3B,MIR200A,MIR6726,CPSF3L,MRPL20,C1orf233	0	loss
1834	CGTE_24	1	16955102	32051476	1p36.11-p35.2	CROCC,MST1L,HTR1D,MED18,EXTL1,HMGN2,FAM231C,MIR6731,CNR2,SNORA16A,HSPG2,NBPF3,IFNLR1,SESN2,CLIC4,C1QC,MIR4425,GRHL3,PAFAH2,MIR4253,FABP3,ATP13A2,LOC101928303,NKAIN1,PLA2G2D,PTAFR,CDC42,PHACTR4,ACTL8,MDS2,EMC1,AKR7A2,PADI2,ZNF593,DDOST,SFN,LACTBL1,PAX7,PADI6,VWA5B1,LYPLA2,TRIM63,FUCA1,PITHD1,FAM46B,LIN28A,RCAN3,KDF1,C1QB,GPATCH3,CROCCP2,UBR4,MRTO4,MECR,RCC2,RHCE,LDLRAP1,PADI4,PTPRU,EPHA8,RPL11,CAMK2N1,DHDDS,USP48,CELA3B,E2F2,SCARNA1,RNU11,LDLRAD2,TRNP1,MIR6127,MST1P2,SNHG3,NR0B2,IFI6,LOC101928324,PLA2G2F,CEP85,AHDC1,ZBTB40,MIR3917,IL22RA1,OTUD3,TAS1R2,ID3,SH3BGRL3,MIR1290,TMEM57,WNT4,GPR3,ACTG1P20,C1orf234,SNHG12,SNORD103B,PIGV,TCEB3,LINC01355,FAM110D,LOC100506801,ALPL,NIPAL3,LOC646471,LOC100506730,PINK1-AS,SDHB,DNAJC8,MYOM3,WASF2,PINK1,RCAN3AS,SDC3,RPS14P3,AKR7A3,SMPDL3B,FAM76A,ARID1A,IGSF21,LOC101929406,SH2D5,TMEM50A,PUM1,LOC284632,MIR3115,PAQR7,ASAP3,KIF17,UBXN10-AS1,CD52,SNORA73B,STMN1,RAB42,STPG1,THEMIS2,CD164L2,MIR3675,ARHGEF10L,LUZP1,ZDHHC18,RPS6KA1,HTR6,RSRP1,MIR378F,HP1BP3,LOC101928163,MATN1,EYA3,PLA2G2A,ALDH4A1,CAPZB,PLA2G5,MIR4695,AKR7L,SERINC2,CDA,EPHB2,SLC30A2,SYTL1,MIR1976,PLA2G2E,ZNF683,PPP1R8,MATN1-AS1,MINOS1-NBL1,TINAGL1,PNRC2,ECE1,EPB41,MIR4684,KDM1A,LOC101928043,TCEB3-AS1,LINC00339,SLC9A1,NCMAP,LOC101928460,MINOS1,KLHDC7A,MTFR1L,ZNF436,RCC1,SYF2,SRSF4,RPA2,SRSF10,SNORD103A,TAF12,WDTC1,MAP3K6,FAM43B,MIR3972,MAN1C1,GPN2,MFAP2,CNKSR1,LAPTM5,CELA3A,GMEB1,RNF186,STX12,SNRNP40,SEPN1,TRNAU1AP,ATPIF1,CATSPER4,PADI1,MIR6084,PDIK1L,ZNF436-AS1,LOC101927876,SNORA44,MUL1,HNRNPR,TCEA3,RAP1GAP,TMEM200B,SRRM1,YTHDF2,PQLC2,LINC01225,C1QA,ZCCHC17,MIR1256,NUDC,HMGCL,FGR,FAM231A,TMEM222,XKR8,AIM1L,NBL1,RUNX3,OPRD1,SNORD99,PADI3,SNORD103C,PLA2G2C,TMCO4,RHD,LINC01226,LOC100506985,IFFO2,SNORA61,AUNIP,MIR4420,EIF4G3,UBXN10,LINC01141,ESPNP,MIR4419A,UBXN11,FCN3,GALE,MIR4418	1	loss

1835	CGTE_24	1	32052133	60538408	1p34.2-p35.2	GJA4,ZMPSTE24,SCMH1,AGO4,TESK2,FOXO6,SLC2A1-AS1,RPS15AP10,NCDN,MYSM1,CCDC30,STK40,ELAVL4,DMRTA2,C1orf185,CITED4,NFYC,TINAGL1,BSDC1,BMP8B,LINC01389,AZIN2,ZMYM4,MYCBP,ZNF684,MIR3659,TEKT2,ZBTB8B,EBNA1BP2,HEYL,MIR3605,TRIM62,YRDC,KPNA6,SPATA6,SPOCD1,FOXJ3,MFSD2A,NT5C1A,UQCRH,RPS8,KTI12,CDCA8,MIR1273F,SKINTL,MMACHC,SNORA110,MIR4711,ERI3,C1orf94,BEST4,ZBTB8A,PHC2,EIF3I,LRP8,FHL3,CYP4A22,CCDC24,KIF2C,LSM10,CCDC28B,TEX38,TSPAN1,RNF220,SNORD55,PPIE,MIR6735,EIF2B3,OSBPL9,C1orf123,DAB1-AS1,TMEM125,FAM183A,HSPB11,BTBD19,HPDL,TMEM59,SLC6A9,MIR4255,ZFP69,SLC1A7,MIR6732,PRKAA2,KLF17,BEND5,HOOK1,MKNK1-AS1,MAGOH,MIR30E,TMCO2,SLC5A9,HCRTR1,EFCAB14,FAM151A,GJB3,PEF1,ATPAF1,LOC100129924,LOC101929464,MIR5585,TXNDC12,HPCAL4,MIR4422,LEXM,LURAP1,HSD52,C8A,ZFYVE9,ZCCHC11,SLFNL1,C1orf216,ORC1,LRRC42,CSMD2-AS1,PARS2,DHCR24,SVBP,TMEM69,KNCN,MPL,RLF,MIR30C1,NSUN4,MROH7,CSMD2,ERI3-IT1,PCSK9,AGBL4-IT1,C1orf210,LINC01144,C1orf109,MANEAL,ZMYM1,SCP2,ZMYM6NB,MIR5095,ZSWIM5,TCEANC2,MIR6734,SNORA55,MRPL37,NASP,CDKN2C,AGO1,OSCP1,FOXD2-AS1,LOC101929516,SMAP2,C1orf228,TAL1,USP24,LOC100507634,GPBP1L1,ADGRB2,ZBTB8OS,NDUFS5,MIR4421,HIVEP3,ZC3H12A,BMP8A,P3H1,OMA1,TRAPPC3,SSBP3-AS1,CYP4A11,TRIT1,SNORD38A,RAB3B,ST3GAL3,LINC01135,CYP2J2,TTC22,FGGY,PRPF38A,LINC01398,MIR4781,LOC101929592,TMEM53,TFAP2E,ZYG11B,AGBL4,MOB3C,LINC01343,MYCL,KHDRBS1,MUTYH,KDM4A-AS1,PIK3R3,COL8A2,HPCA,GUCA2A,MIR6500,DMAP1,TMEM39B,FAAHP1,SFPQ,GJA9,RIMKLA,A3GALT2,FAAH,LOC105378732,DPH2,TTC39A,KIAA0754,PTP4A2,ZMYM6,LCK,ZNF691,SLC2A1,GNL2,CCDC17,ERMAP,LOC100507564,CLDN19,CYP4X1,EPHA10,YARS,ECHDC2,BTF3L4,MEAF6,LINC00853,PPIH,PTPRF,SNIP1,MIR5584,DLGAP3,ZFP69B,POU3F1,KCNQ4,SYNC,TSSK3,B4GALT2,DMRTB1,JUN,LINC01137,POMGNT1,CCDC163P,SNORD38B,MAST2,PPT1,PTCH2,CYP4Z1,ZNF362,TRABD2B,RIMS3,PLK3,EPS15,HYI,LOC105378683,TACSTD2,SLFNL1-AS1,NRDC,GPX7,TTC4,SZT2,EDN2,NFYC-AS1,COA7,EXO5,FAF1,HMGB4,DAB1,GJA9-MYCBP,GJB4,ZMYND12,CLSPN,C8B,MTMR9LP,PRDX1,HECTD3,RSPO1,UTP11L,CFAP57,MED8,ZYG11A,PSMB2,IQCC,BSND,ELOVL1,THRAP3,CMPK1,CSF3R,KIAA1522,COL16A1,SH3D21,CYP4Z2P,MROH7-TTC4,CDC20,LOC339539,KIAA0319L,DIO1,TIE1,S100PBP,AGO3,MAP7D1,PDZK1IP1,LRRC41,AKIRIN1,RAD54L,CAP1,RBBP4,MIR552,C1orf168,RNF11,RRAGC,CYB5RL,FOXE3,PODN,MIR761,MIR6733,COL9A2,NDC1,OXCT2P1,YBX1,CDCP2,PABPC4,EVA1B,TOE1,C1orf122,MRPS15,TTC39A-AS1,TMEM234,LOC101929626,CYP4B1,GRIK3,LDLRAD1,FAM159A,PPCS,C1orf50,PLPP3,ACOT11,CTPS1,TXNDC12-AS1,OXCT2,CC2D1B,YIPF1,GUCA2B,SLC25A3P1,DNALI1,LINC01358,CPT2,AKR1A1,INPP5B,MIR6079,ATP6V0B,EFCAB14-AS1,MARCKSL1,MIR5581,IPP,HDAC1,AK2,ZSCAN20,MTF1,TMEM54,DCDC2B,FNDC5,TXLNA,STIL,TCTEX1D4,MIR1273G,SSBP3,SF3A3,TMEM61,FAM229A,C1orf87,PPIEL,ARTN,GJB5,ADPRHL2,RHBDL2,UROD,LOC101929721,MKNK1,FAM167B,LOC653160,DMBX1,MACF1,	3	gain
1836	CGTE_24	1	109810627	109815044	1p13.3	CELSR2	6	gain
1837	CGTE_24	1	228464505	228479809	1q42.13	OBSCN	5	gain
1838	CGTE_24	2	27260982	27262097	2p23.3	TMEM214	20	gain
1839	CGTE_24	2	72359386	72371345	2p13.2	CYP26B1	7	gain
1840	CGTE_24	2	73479806	73491115	2p13.2	FBXO41,CCT7	3	gain

1841	CGTE_24	2	95942963	101126073	2q11.1-q11.2	CNNM4,FAHD2CP,NEURL3,ANKRD39,VWA3B,FAHD2B,ADRA2B,ZAP70,PROM2,CNNM3,EIF5B,LYG1,TRIM43,LYG2,LMAN2L,TMEM127,COA5,ANKRD23,LINC01104,ANKRD36,ITPRIPL1,FER1L5,CIAO1,LOC101927053,TXNDC9,LOC101927070,TSGA10,ANKRD36B,UNC50,COX5B,MIR3127,ACTR1B,SNRNP200,FAM178B,KIAA1211L,KCNIP3,LOC100506123,MRPL30,MITD1,LINC01125,C2orf15,ARID5A,CHST10,ANKRD36C,AFF3,LINC00342,STARD7-AS1,KANSL3,NMS,FAHD2A,CNGA3,LIPT1,SEMA4C,STARD7,NCAPH,GPAT2,LONRF2,REV1,TRIM43B,DUSP2,TMEM131,ASTL,LOC100506076,INPP4A,MGAT4A	3	gain
1842	CGTE_24	2	128381736	128388817	2q14.3	MYO7B	6	gain
1843	CGTE_24	2	186620916	186626792	2q32.1	FSIP2	42	gain
1844	CGTE_24	2	220349256	220355707	2q35	SPEG	6	gain
1845	CGTE_24	2	233271583	233322505	2q37.1	ALPI,ALPPL2	5	gain
1846	CGTE_24	2	233404337	233405564	2q37.1	CHRNG	9	gain
1847	CGTE_24	2	239006590	239056757	2q37.3	KLHL30,UBE2F-SCLY,SCLY,ESPNL	0	loss
1848	CGTE_24	2	239057642	239077233	2q37.3	FAM132B,KLHL30	4	gain
1849	CGTE_24	2	240011689	240036937	2q37.3	HDAC4	0	loss
1850	CGTE_24	2	240036941	240061531	2q37.3	HDAC4	8	gain
1851	CGTE_24	2	240062556	242842580	2q37.3	OTOS,AQP12A,C2orf54,CAPN10-AS1,PRR21,D2HGDH,LOC150935,MGC16025,MIR2467,GAL3ST2,MTERF4,PP14571,OR6B3,MYEOV2,BOK,PASK,BOK-AS1,NEU4,AGXT,THAP4,KIF1A,STK25,SEPT2,ING5,FARP2,ATG4B,LINC01237,HDLBP,DUSP28,LOC200772,GPR35,ANO7,DTYMK,NDUFA10,MIR3133,MIR149,PDCD1,RNPEPL1,MIR4786,CAPN10,ANKMY1,PPP1R7,RTP5,SNED1,HDAC4,AQP12B,MIR4269,GPC1,OR6B2	1	loss
1852	CGTE_24	3	48602227	48613430	3p21.31	COL7A1	3	gain
1853	CGTE_24	3	52521197	52523688	3p21.1	NISCH	7	gain

1854	CGTE_24	3	160395252	187387143	3q25.33-q26.32	MAP6D1,NLGN1,LINC01192,DGKG,LOC344887,LOC100507661,EGFEM1P,MIR5588,MIR6828,TRA2B,PEX5L,SLC2A2,CCDC39,NDUFB5,ATP11B,ECE2,SOX2,MASP1,PARL,PIK3CA,HTR3E-AS1,MCCC1,ACTRT3,SNORA4,MIR4789,MAGEF1,SNORA63,LINC01324,NAALADL2-AS1,EIF4A2,ZNF639,AHSG,FAM131A,SERPINI2,DNAJB11,DCUN1D1,EPHB3,B3GALNT1,IGF2BP2,ADIPOQ,HTR3C,ST6GAL1,LOC101243545,MIR548AY,MIR551B,FETUB,LINC01322,LIPH,FNDC3B,DNAJC19,NLGN1-AS1,LINC01209,GOLIM4,MRPL47,KLHL6,RNU6-2,PHC3,PPM1L,LINC01208,THPO,LINC01014,MECOM,SNORD66,SKIL,TNIK,POLR2H,SOX2-OT,C3orf70,LOC102724699,DVL3,SLC7A14,KCNMB2-AS1,GNB4,GHSR,ABCF3,TBCCD1,FLJ46066,FXR1,NAALADL2-AS2,MIR548AQ,KCCAT211,SI,PEX5L-AS2,ARL14,MCF2L2,ACTL6A,RPL22L1,AP2M1,LOC253573,LOC100128164,ABCC5-AS1,LINC01327,TBL1XR1,EIF4G1,MAP3K13,ETV5,VPS8,TMEM212,SAMD7,ZMAT3,LOC101928882,EIF2B5-AS1,LINC00578,ECT2,CAMK2N2,TERC,LINC01330,LOC101928739,SNORA81,ALG3,SPTSSB,CLDN11,KLHL6-AS1,LAMP3,PDCD10,IGF2BP2-AS1,LOC100505609,PRKCI,TTC14,NAALADL2-AS3,RFC4,MYNN,CLCN2,SLITRK3,RTP1,LINC01206,MIR1224,ABCC5,KCNMB2,MFN1,LINC00888,MIR1263,SEC62,ADIPOQ-AS1,CHRD,WDR49,GPR160,LRRC31,EHHADH-AS1,SPATA16,OTOL1,RPL39L,ZBBX,RTP4,KCNMB3,EHHADH,TNFSF10,LOC101928992,LOC101929106,NAALADL2,LINC00501,HTR3D,MIR7977,VWA5B2,YEATS2,TMEM41A,KLHL24,LRRC34,NMD3,EIF5A2,KNG1,LRRIQ4,HTR3E,B3GNT5,CRYGS,HRG,EIF2B5,SST,LOC102724604,MIR1248,NCEH1,USP13,PSMD2,PLD1,SERPINI1,TMEM212-AS1,MIR569,SENP2,SNORD2,BCHE	3	gain
1855	CGTE_24	3	187387861	187419851	3q27.3	RTP2,SST	8	gain
1856	CGTE_24	3	187419856	196668897	3q29-q27.3	CEP19,TNK2,CLDN1,MGC2889,TP63,TMEM207,LINC00885,TM4SF19-TCTEX1D2,MUC20,FGF12-AS1,ATP13A3,ATP13A4,MIR6829,OSTN-AS1,UBXN7,XXYLT1-AS2,FLJ42393,LINCR-0002,SNAR-I,OPA1-AS1,SLC51A,LINC00969,HRASLS,ATP13A5,LOC100505920,MB21D2,OSTN,DPPA2P3,PCYT1A,LOC100507391,P3H2-AS1,CPN2,SDHAP2,SDHAP1,ATP13A5-AS1,TCTEX1D2,XXYLT1,LINC00884,FGF12,TM4SF19,MIR570,LPP-AS2,TPRG1-AS2,FAM43A,MUC4,LOC100131635,LINC00887,MIR28,MIR3137,TPRG1-AS1,WDR53,IL1RAP,LSG1,LRRC15,LOC647323,CCDC50,LINC01063,LOC101929337,FBXO45,SENP5,PAK2,RTP2,TMEM44,MIR944,MIR5692C1,HES1,LPP-AS1,PPP1R2,TFRC,TPRG1,NCBP2,PYDC2,PIGX,SMCO1,ACAP2,NRROS,TMEM44-AS1,TM4SF19-AS1,UTS2B,LPP,ZDHHC19,GMNC,P3H2,XXYLT1-AS1,BCL6,GP5,APOD,CLDN16,ATP13A4-AS1,RNF168,OPA1	3	gain
1857	CGTE_24	3	196669188	196742468	3q29	MFI2-AS1,NCBP2,PIGZ,MFI2,NCBP2-AS2	5	gain
1858	CGTE_24	3	196743070	197765684	3q29	LMLN,MIR922,IQCG,BDH1,MIR4797,LOC220729,FYTTD1,LMLN-AS1,MFI2,DLG1-AS1,LRCH3,RUBCN,DLG1,RPL35A	3	gain
1859	CGTE_24	4	954336	985752	4p16.3	SLC26A1,DGKQ,IDUA	0	loss
1860	CGTE_24	4	985843	994786	4p16.3	IDUA,SLC26A1	4	gain
1861	CGTE_24	4	3432335	3447980	4p16.3	HGFAC,RGS12	0	loss
1862	CGTE_24	5	140167749	140174656	5q31.3	PCDHA1,PCDHA2	9	gain
1863	CGTE_24	5	180038243	180040333	5q35.3	FLT4	13	gain
1864	CGTE_24	6	1312879	1624844	6p25.3	FOXC1,MIR6720,FOXCUT,FOXQ1,FOXF2,GMDS	0	loss
1865	CGTE_24	6	30859818	30864695	6p21.33	DDR1	8	gain

1866	CGTE_24	6	30864720	32017402	6p21.33	MICB,SNORD52,ATP6V1G2-DDX39B,MSH5,PSORS1C1,LTB,MUC22,LY6G6E,SNORD48,AIF1,TNXA,ABHD16A,C6orf47,GPANK1,LY6G6D,DDAH2,SNORD84,SAPCD1-AS1,LY6G6C,PSORS1C3,CFB,VARS,HLA-B,C6orf15,APOM,CDSN,LST1,MIR4646,VWA7,C2,LY6G6F,GTF2H4,DPCR1,HCG22,MIR1236,MCCD1,C4B,MUC21,LY6G5B,MIR6832,CSNK2B,LTA,SNORA38,PRRC2A,BAG6,TNF,HLA-C,SLC44A4,LY6G5C,TCF19,LSM2,NFKBIL1,NCR3,TNXB,HCG26,SNORD117,SKIV2L,HSPA1B,DXO,PSORS1C2,DDX39B-AS1,C4B_2,ATP6V1G2,MSH5-SAPCD1,VARS2,CYP21A2,POU5F1,HCP5,MICA,SFTA2,CLIC1,ZBTB12,EHMT2,NEU1,HSPA1A,DDR1,C6orf25,C2-AS1,HSPA1L,SAPCD1,DDX39B,C4A,NELFE,CYP21A1P,CCHCR1,MIR6891,HCG27,STK19,C6orf48	3	gain
1867	CGTE_24	6	32017678	32035471	6p21.33	TNXB	8	gain
1868	CGTE_24	6	32035646	33641476	6p21.32-p21.33	B3GALT4,FKBPL,NOTCH4,RXRB,MIR6721,PRRT1,AGER,PSMB9,RNF5,HLA-DRB6,HLA-DMB,HLA-DPB1,HLA-DPB2,KIFC1,ZBTB9,HLA-DRB1,HCG25,HLA-DQB2,HLA-DRA,LINC00336,SYNGAP1,ZBTB22,MIR6833,PPT2-EGFL8,HSD17B8,LOC100507547,HLA-DQA2,HLA-DOB,GPSM3,RPS18,GGNBP1,MIR5004,HLA-DQB1,TAP2,TNXB,MIR1234,RING1,COL11A2,BRD2,PSMB8,ITPR3,VPS52,ATF6B,TAPBP,DAXX,HCG23,HLA-DQA1,PFDN6,MIR219A1,HLA-DMA,MIR3135B,TAP1,RGL2,CUTA,PHF1,SLC39A7,C6orf10,BAK1,MIR6834,HLA-DPA1,PBX2,BTNL2,PSMB8-AS1,EGFL8,WDR46,RNF5P1,MIR6873,LOC100294145,HLA-DOA,PPT2,HLA-DRB5,AGPAT1	3	gain
1869	CGTE_24	6	33641890	33656195	6p21.31	ITPR3	4	gain
1870	CGTE_24	6	33656421	36689121	6p21.31-p21.2	MLN,MIR7111,TULP1,MIR5690,DEF6,RPS10-NUDT3,MIR3925,C6orf106,MIR7159,C6orf1,ITPR3,CLPSL2,C6orf222,ZNF76,ARMC12,UQCC2,HMGA1,MIR1275,MIR6835,CLPS,TCP11,SLC26A8,FANCE,RPL10A,SCUBE3,LEMD2,PACSIN1,MIR3934,NUDT3,GRM4,PPARD,MAPK13,ANKS1A,CLPSL1,MAPK14,STK38,SRSF3,RPS10,UHRF1BP1,TAF11,SRPK1,PANDAR,SPDEF,LOC285847,TEAD3,PXT1,CDKN1A,PNPLA1,LHFPL5,RAB44,BRPF3,KCTD20,SNRPC,FKBP5,ETV7,IP6K3,LINC01016	3	gain
1871	CGTE_24	6	36689126	36690514	6p21.2	RAB44	6	gain

1872	CGTE_24	6	36693561	52996903	6p12.3-p21.1	C6orf89,MED20,C6orf141,GSTA3,TMEM217,SUPT3H,CNPY3,GSTA7P,TRAM2,CDC5L,LINC00951,CMTR1,LOC101927020,DAAM2,LOC101929555,CRISP1,XPO5,TREML5P,POLR1C,LOC101927082,PGK2,CD2AP,IL17A,RNF8,MIR4462,GCM1,MRPL2,TREML1,USP49,TTBK1,TBCC,PIM1,MDGA1,KLHDC3,DEFB133,MTCH1,LOC101929705,KLC4,GLYATL3,TOMM6,OARD1,GSTA2,C6orf132,TFAP2B,RPL7L1,MRPS10,APOBEC2,MIR133B,NFKBIE,MIR4642,ICK,GSTA4,CAPN11,LRRC73,VEGFA,MUT,MEP1A,UNC5CL,TDRD6,LOC101926915,PGC,TRERF1,LINC01276,TREML4,LOC100131047,LOC101926898,LRFN2,PRICKLE4,SLC29A1,C6orf223,PTCRA,MOCS1,BYSL,MIR6780B,TREML2,NCR2,EFHC1,LOC101926962,DNPH1,CENPQ,LOC101929726,GTPBP2,MCM3,SAYSD1,RRP36,MIR4647,CYP39A1,GNMT,ENPP4,ANKRD66,TMEM14A,MRPL14,MRPS18A,POLH,ADGRF2,SPATS1,PTCHD4,GLTSCR1L,CRISP2,RUNX2,TMEM151B,TFAP2D,KCNK5,KCNK16,DLK2,TFEB,CUL9,GUCA1B,DNAH8,FOXP4-AS1,MIR206,CCND3,FGD2,TDRG1,RCAN2,MEA1,ADCY10P1,PRPH2,TSPO2,ZFAND3,ATP6V0CP3,PPIL1,OPN5,CRISP3,TBC1D22B,RAB44,PI16,GLP1R,LOC100505530,GLO1,LOC730101,ADGRF5,CPNE5,TRAM2-AS1,PKHD1,LOC100505635,CLIC5,SLC35B2,CUL7,SLC25A27,SRF,KIF6,MDFI,PPP2R5D,FBXO9,ADGRF1,YIPF3,PEX6,TCTE1,GSTA1,PAQR8,ABCC10,SLC22A7,GSTA5,FOXP4,ADGRF4,TREML3P,CCDC167,DEFB114,PTK7,ENPP5,DEFB113,TMEM63B,BTBD9,IL17F,LINCMD1,RHAG,LINC01512,KCNK17,TJAP1,TAF8,FRS3,DEFB112,PLA2G7,TREM2,NFYA,RSPH9,AARS2,DEFB110,MIR586,CRIP3,MAD2L1BP,GUCA1A,UBR2,MIR4641,ZNF318,C6orf226,LOC101927048,TNFRSF21,HSP90AB1,TREM1	3	gain
1873	CGTE_24	7	195557	4829883	7p22.2-p22.1	CYP2W1,GRIFIN,ZFAND2A,COX19,FOXK1,FAM20C,ADAP1,WI2-2373I1.2,LOC101926963,CHST12,MAFK,MIR6836,DNAAF5,PSMG3-AS1,LOC101927021,SUN1,ELFN1-AS1,TFAMP1,HRAT92,PRKAR1B,UNCX,AP5Z1,GPER1,LOC100129603,MIR4648,FTSJ2,TTYH3,MIR4656,MIR339,TMEM184A,NUDT1,SDK1,C7orf50,LOC442497,GNA12,PDGFA,AMZ1,BRAT1,IQCE,SNX8,LOC101927000,INTS1,MAD1L1,GPR146,ELFN1,MICALL2,EIF3B,LFNG,PSMG3,CARD11,GET4,MIR4655,LOC101927181	3	gain
1874	CGTE_24	7	4829888	4856142	7p22.1	AP5Z1,RADIL	0	loss
1875	CGTE_24	7	4856159	5334721	7p22.1	RBAK,RBAKDN,WIPI2,RBAK-RBAKDN,ZNF890P,RADIL,PAPOLB,MMD2,RNF216P1,SLC29A4	3	gain
1876	CGTE_24	7	5336597	5643151	7p22.1	ACTB,FBXL18,FSCN1,LOC221946,TNRC18,MIR589,SLC29A4	0	loss

1877	CGTE_24	7	5643155	77016785	7p12.1-p15.2	KLHL7,MIR4650-2,MDH2,GARS,POM121L12,AUTS2,PRPS1L1,UPK3B,TYW1,DPY19L1,HERPUD2,FAM221A,LOC101927811,LINC00957,SNORA22,HOXA7,CCZ1B,GTF2IRD2,PKD1L1,SRRM3,MIR3683,DDX56,ZNF815P,KIAA0087,LOC102723427,ZNF117,CAMK2B,GTF2I,LOC101927630,LOC646762,GSAP,WBSCR17,EGFR,ANKMY2,LOC541473,PPIA,SEPT7P2,POM121,FIGNL1,TBX20,MIR196B,LOC102724484,LAT2,GS1-124K5.11,COBL,MIR550B1,HOXA1,GUSB,SPDYE5,FERD3L,SPDYE7P,AGR2,HOXA10-HOXA9,RASA4CP,ANLN,TAX1BP1,SNX13,ELDR,HSPB1,STK17A,MRPS24,STAG3L1,NME8,KIAA0895,VPS41,MIR6837,LOC100507468,HOTTIP,FAM126A,NCF1B,LOC100506178,GTF2IRD2B,TRIM50,GRB10,TMEM248,NOD1,INMT-FAM188B,INTS4P2,HOXA4,ZNF92,C7orf31,TPST1,LOC101928168,TSL,PSMA2,UMAD1,TOMM7,WBSCR22,STEAP1B,UBE2D4,CRCP,IGFBP3,LSM5,DPY19L1P1,POM121C,BMPER,YWHAEP1,MACC1-AS1,OGDH,MIR148A,GTF2IRD1,KBTBD2,LINC00525,RNF216,DKFZP586I1420,PHF14,MYL7,RALA,EGFR-AS1,CCL26,SNORA5C,STAG3L4,ANKRD61,RABGEF1,MIR1183,RPS2P32,MIR4283-1,COL28A1,LOC101927668,FGL2,ERV3-1,RSPH10B,SFRP4,SEPT7,POMZP3,SNORA14A,MIR3914-2,MRPS17,MACC1,PMS2P3,LINC01449,LOC541472,LOC641746,TNS3,TWIST1,DDC,LOC650226,MIR550A1,ELN,C1GALT1,ETV1,LRRC72,TBL2,NACAD,MIR3943,POR,HOXA13,H2AFV,MIR4650-1,ZNF138,GTF2IP1,FKBP9,WBSCR28,CCDC146,BCL7B,HECW1,SNX10,JAZF1,TRG-AS1,POU6F2,LINC01448,IL6,SP8,PMS2P2,INHBA,FKBP9P1,YAE1D1,NDUFA4,MIR550A3,NUPL2,FKBP14,NT5C3A,STX1A,MIR6874,NPSR1-AS1,CCDC129,MIR4649,PMS2,NSUN5,HOTAIRM1,COA1,UPP1,GLCCI1,SKAP2,GPNMB,CDCA7L,NPSR1,GTF2IP4,NPVF,ZNF713,EEPD1,HOXA-AS3,ITGB8,FAM188B,MIR550B2,AEBP1,TRA2A,INMT,ABCB5,GBAS,NCF1,LOC101928618,URGCP,VWC2,STARD3NL,TBRG4,LOC100133091,HOXA5,YWHAG,C7orf72,STAG3L3,SUMF2,DNAH11,LOC100505921,C7orf26,DDC-AS1,LOC101927769,AGR3,C7orf25,MIR590,LOC100505938,CYCS,C7orf65,DTX2P1-UPK3BP1-PMS2P11,ARL4A,AGMO,LOC100240728,IGF2BP3,ZNF736,PPP1R17,FKBP6,LOC100131257,LOC100101148,HOXA6,MIR550A2,DFNA5,TMEM106B,C7orf69,LOC441204,POLM,IGFBP1,TMEM196,GUSBP10,KCTD7,LOC100506497,ABHD11,ADCYAP1R1,HOXA2,TWISTNB,PMS2CL,WBSCR27,FSCN1,LOC100996437,BZW2,SNORA5A,SNHG15,HOXA9,NFE2L3,STK31,TRIM73,ZPBP,ZNF735,AOAH,ISPD,SNORA9,CLK2P1,AMPH,JAZF1-AS1,LINC01176,SCIN,INHBA-AS1,MEOX2-AS1,EPDR1,MIR3914-1,TMED4,NSUN5P2,GHRHR,LINC01372,GCK,RAPGEF5,CCM2,HOXA10,STYXL1,LOC101927391,CLDN4,IKZF1,CYTH3,EVX1-AS,ZNF107,FAM220A,LINC00997,MIR3146,SNORA5B,MYO1G,LOC401312,MIR3147,AVL9,MIOS,RAC1,SEPT14,SNORA15,SUGCT,BBS9,GRID2IP,ZNF316,PURB,KDELR2,HDAC9,NSUN5P1,VWDE,MEOX2,PRR15,HOXA-AS2,LINC01450,C7orf57,SSC4D,CDK13,LOC401324,TRIL,ZDHHC4,MALSU1,CPVL,ELMO1-AS1,LOC100130849,DPY19L2P1,POU6F2-AS1,NPC1L1,EIF2AK1,ISPD-AS1,LOC401320,PGAM2,ZNF12,ZNRF2,LOC100287704,MIR4283-	3	gain
1878	CGTE_24	7	77026242	77221627	7q11.23	GSAP,PTPN12,LOC101927243	7	gain

1879	CGTE_24	8	190799	17188875	8p23.1-p22	USP17L8,FBXO25,LINC00599,DEFB4A,DLC1,C8orf74,DEFB135,DEFB103A,FAM86B1,LOC392196,LOC340357,DEFA5,MIR596,LOC101929229,DEFB1,LINC00965,MIR3674,PRR23D2,MTMR9,SLC35G5,BLK,ZNF705G,DEFT1P,KBTBD11,C8orf48,DEFB136,SNORA99,CTSB,MIR124-1,SGK223,LINC00208,GS1-24F4.2,FAM66A,FAM90A7P,DEFA1B,DEFA3,MIR4659A,NEIL2,ARHGEF10,DEFA9P,MIR1322,DEFB103B,LINC00681,LOC729732,MIR3926-2,FAM90A10P,ZNF705D,FAM66D,LOC157273,ANGPT2,PRR23D1,USP17L3,SGCZ,MIR383,DEFB104B,MCPH1-AS1,CNOT7,USP17L1,LOC649352,GATA4,MICU3,MIR5692A2,DEFA1,MIR3926-1,DEFA8P,MCPH1,DEFA6,ERICH1-AS1,KBTBD11-OT1,ERI1,USP17L4,DEFB109P1B,FAM66E,DEFB106B,MIR598,FAM86B3P,DEFB130,MFHAS1,LOC101927815,ZDHHC2,DEFA4,DEFB105B,DLGAP2,FAM86B2,MSRA,FAM90A2P,RP1L1,MTMR7,ZNF596,TDH,MIR4659B,XKR6,DEFB106A,DEFA10P,FAM87A,MIR8055,CLDN23,SPAG11A,FAM167A-AS1,LINCR-0001,PPP1R3B,LOC101929128,PINX1,DEFB134,KIAA1456,SOX7,LONRF1,AGPAT5,MIR7160,LOC100287015,DEFB104A,XKR5,DEFB109P1,DEFA11P,ERICH1,DLGAP2-AS1,LOC100133267,LOC100506990,FGF20,MIR4286,MIR597,USP17L7,TUSC3,VPS37A,CSMD1,FAM90A25P,DEFT1P2,MSR1,DEFB105A,MIR5692A1,FAM167A,TDRP,TNKS,USP17L2,PRSS55,DEFB107B,MIR548I3,DEFB107A,DEFB4B,MYOM2,C8orf49,FAM66B,ZNF705B,SPAG11B,LOC286083,FDFT1,MIR4660,CLN8	1	loss
1880	CGTE_24	8	17198797	28998121	8p21.3-p21.1	BMP1,LZTS1-AS1,PPP2R2A,STMN4,NEFL,LOC389641,R3HCC1,ESCO2,FAM160B2,MIR320A,MIR6876,SLC39A14,TNFRSF10B,RHOBTB2,LOC100128993,GFRA2,CHMP7,LPL,ASAH1,CCAR2,TNFRSF10A,LOC101929294,CHRNA2,HMBOX1,ADAMDEC1,PHYHIP,CSGALNACT1,FGF17,EGR3,NEFM,LOC101929172,ADRA1A,CDCA2,ENTPD4,ADAM28,NUGGC,DPYSL2,CLU,PDLIM2,EBF2,MIR6842,SORBS3,TRIM35,BIN3-IT1,NUDT18,PIWIL2,POLR3D,LOC102467222,MIR4288,HR,INTS9,NAT1,FBXO16,LOC254896,LZTS1,NKX3-1,PCM1,MIR6841,XPO7,SLC7A2,DMTN,LGI3,DOK2,PNMA2,PSD3,CCDC25,GNRH1,PEBP4,MTUS1,EXTL3,LOC101929066,PBK,FZD3,ATP6V1B2,MIR6843,NKX2-6,BIN3,ELP3,MIR7641-2,EPHX2,MTMR7,MIR3622A,LOXL2,ADAM7,FGL1,SLC18A1,PNOC,STC1,INTS10,KIF13B,PPP3CC,EXTL3-AS1,TNFRSF10D,ZNF395,PDGFRL,C8orf58,LOC286114,LOC101929237,KCTD9,MIR548V,LOC101929315,PTK2B,NAT2,TNFRSF10C,MIR3622B,REEP4,LOC286059,SLC25A37,LOC100507156,NPM2,DOCK5,SCARA5,SH2D4A,MIR4287,BNIP3L,SFTPC,SCARA3	3	gain
1881	CGTE_24	8	48196565	54163625	8q11.22-q11.23	SNTG1,PRKDC,NPBWR1,CEBPD,RB1CC1,LOC101929268,MCM4,FAM150A,EFCAB1,LOC101929217,PCMTD1,SPIDR,UBE2V2,OPRK1,ST18,C8orf22,SNAI2,PXDNL	1	loss

1882	CGTE_24	8	72987419	103327113	8q22.1-q21.3	SLC10A5,MIR3150B,C8orf87,LINC01111,STMN2,COX6C,OTUD6B,SDC2,LOC100500773,OTUD6B-AS1,LOC101241902,LOC101927066,RDH10,TP53INP1,ZBTB10,RBM12B,FABP5,MIR875,RBM12B-AS1,LINC00535,FBXO43,FAM92A1,RNF19A,VPS13B,PI15,C8orf37-AS1,TRPA1,MIR378D2,FSBP,KCNS2,PMP2,MIR7705,KIAA1429,TPD52,TERF1,UBR5,SNORA72,IMPA1,MRPS28,UBR5-AS1,IL7,LINC01030,DPY19L4,CA2,NDUFAF6,TSPYL5,INTS8,MIR8084,STAU2,FABP4,LOC102724804,MIR5681B,LINC01607,LINC01298,POP1,MIR5681A,LOC104054148,LOC102724710,C8orf37,HRSP12,RPL7,MIR599,PLEKHF2,PEX2,RALYL,PAG1,ZFHX4,SBSPON,JPH1,CA13,NECAB1,MIR5708,RPL30,SLC7A13,PKIA,LINC00534,MIR7641-2,TMEM55A,KCNB2,MIR2052,RIPK2,LOC392232,ZFHX4-AS1,ATP6V0D2,ZFAND1,TMEM67,RMDN1,C8orf59,TMEM64,E2F5,MTDH,RAD54B,NBN,LOC101927040,CA1,FABP12,MIR4471,TMEM70,ZC2HC1A,OSGIN2,MIR5680,MIR3149,CRISPLD1,FLJ42969,CALB1,RRM2B,YWHAZ,CA3-AS1,CASC9,STAU2-AS1,CA3,MATN2,FLJ46284,RGS22,PTDSS1,RDH10-AS1,HNF4G,C8orf88,FABP9,ZNF706,LOC102724874,GDAP1,LOC105375650,ERICH5,SLC26A7,PABPC1,SPAG1,POLR2K,MIR2052HG,UBE2W,PKIA-AS1,LAPTM4B,ESRP1,LOC100288748,REXO1L2P,CNBD1,LOC101929709,MIR4661,NACAP1,PDP1,GRHL2,DECR1,SNX16,C8orf89,MIR3150A,HEY1,LRRCC1,MTERF3,ZNF704,TRIQK,CCNE2,TCEB1,LOC101926908,CPNE3,MIR1273A,PSKH2,RUNX1T1,WWP1,CDH17,UQCRB,STK3,CHMP4C,ANKRD46,CNGB3,DCAF4L2,OSR2,NIPAL2,LY96,MMP16,CPQ,LINC01419,GEM,GDF6,NCALD,LRRC69,SNX31	3	gain
1883	CGTE_24	8	117669324	143604143	8q24.13-q24.23	EIF3H,AARD,CCAT2,RAD21-AS1,GSDMC,PTP4A3,TRIB1,ASAP1-IT2,TMEM75,CCDC26,DEPTOR,MRPL13,DENND3,ATAD2,ADGRB1,ENPP2,CASC19,EFR3A,LINC00861,MIR30D,TNFRSF11B,MIR1204,LOC101927915,SLC30A8,C8orf76,RAD21,CHRAC1,MIR6844,HAS2,NDUFB9,MIR1208,MIR4662A,MIR1302-7,LINC01300,DERL1,ASAP1,PTK2,LRRC6,NCRNA00250,TMEM65,LINC00977,CCAT1,ASAP1-IT1,TBC1D31,WDYHV1,MTBP,LOC101927657,SQLE,SNTB1,FER1L6-AS1,HAS2-AS1,LINC01591,KLHL38,MED30,TRMT12,ZFAT-AS1,HPYR1,TMEM71,NDRG1,KIAA0196,FER1L6,ANXA13,TRAPPC9,MIR1205,TAF2,HHLA1,CASC11,LOC101927543,WISP1,LINC01151,ADCY8,FBXO32,MTSS1,MIR5194,FAM49B,MIR4539,OC90,LINC00964,ZHX1,LOC101927798,PVT1,CASC8,FAM91A1,NSMCE2,SAMD12,EXT1,MIR1206,CASC21,LOC101927588,POU5F1B,TSNARE1,FER1L6-AS2,UTP23,ZHX1-C8orf76,MIR1207,PCAT1,FAM135B,MIR3686,AGO2,SLA,MIR7848,COL22A1,MIR3610,ST3GAL1,RNF139-AS1,PCAT2,MROH5,ZNF572,LINC00824,ZHX2,NOV,ZFAT,MIR4472-1,LOC101927822,LOC101927845,TATDN1,SLC45A4,FAM84B,LINC00051,KHDRBS3,MIR4662B,COLEC10,RNF139,KCNQ3,MAL2,KCNK9,COL14A1,MIR30B,LOC105375734,PHF20L1,GPR20,DSCC1,MIR4663,FAM83A-AS1,FAM83A,TG,MYC,PRNCR1,SAMD12-AS1	3	gain
1884	CGTE_24	8	143605476	143695649	8q24.3	ARC,ADGRB1	0	loss
1885	CGTE_24	8	143696644	144130746	8q24.3	LY6K,LYPD2,LOC100133669,LOC100288181,GML,JRK,LY6D,C8orf31,CYP11B1,LYNX1,CDC42P3,SLURP1,CYP11B2,THEM6,LY6E,PSCA	3	gain

1886	CGTE_24	8	144162370	145138426	8q24.3	TSTA3,OPLAH,MIR6847,MINCR,ZNF707,GSDMD,SCRIB,FAM83H-AS1,RHPN1-AS1,GPAA1,MAPK15,MIR937,NRBP2,GLI4,EPPK1,MIR6846,MIR661,BREA2,ZC3H3,MROH6,MAFA,MIR6845,FAM83H,CCDC166,RHPN1,GRINA,PYCRL,PLEC,EXOSC4,MAFA-AS1,TIGD5,SPATC1,EEF1D,NAPRT,ZNF623,ZNF696,PARP10,TOP1MT,MIR4664,GPIHBP1,PUF60,ZFP41,LY6H	0	loss
1887	CGTE_24	8	145138598	145583658	8q24.3	MROH1,CYC1,MIR7112,MIR6848,FBXL6,SCRT1,DGAT1,GPAA1,WDR97,BOP1,TMEM249,SCX,HSF1,MAF1,SLC52A2,SHARPIN,HGH1	3	gain
1888	CGTE_24	8	145583659	145739940	8q24.3	MIR6849,VPS28,MFSD3,SLC52A2,SLC39A4,CYHR1,PPP1R16A,RECQL4,KIFC2,CPSF1,FOXH1,TONSL-AS1,GPT,MIR6893,MIR939,ADCK5,TONSL	0	loss
1889	CGTE_24	8	145740177	146279543	8q24.3	ZNF34,RECQL4,COMMD5,LRRC14,ZNF251,ARHGAP39,ZNF517,LRRC24,TMED10P1,C8orf82,C8orf33,MIR6850,ZNF252P,ZNF7,ZNF250,ZNF252P-AS1,RPL8,ZNF16	3	gain
1890	CGTE_24	9	214455	977298	9p24.3	KANK1,DOCK8,DMRT3,C9orf66,DMRT1	1	loss

1891	CGTE_24	9	115931543	138837937	9q33.3-q33.2	LOC51145,NCS1,DNM1,FBXW2,LOC100272217,LOC101929116,CAMSAP1,ASTN2-AS1,SURF1,ENG,CDK9,MEGF9,SNORA65,GOLGA2,PRRX2,ENDOG,PIP5KL1,DFNB31,RAPGEF1,TMEM8C,C9orf62,SLC31A1,NEK6,PTGES2,DENND1A,FNBP1,MIR219A2,SURF6,LCN9,RABGAP1,OR1N2,ORM1,TSC1,LOC101448202,PHF19,OR1N1,CERCAM,UBAC1,MIR1268A,C9orf16,LOC101928748,NRON,MIR7150,SLC2A8,AIF1L,MIR455,COL27A1,ORM2,CACFD1,SLC2A6,SPTAN1,PPP2R4,REXO4,FAM78A,SLC27A4,GAPVD1,MIR2861,CFAP157,OR1J1,OR1L6,ZNF618,LCN2,CRAT,COQ4,MIR4672,KCNT1,ASS1,MED22,PRRC2B,ZBTB6,OR1J4,LOC100506100,PAPPA,SNORD62B,MIR181A2,PRRX2-AS1,PTRH1,PSMD5-AS1,OR1B1,OR1K1,SET,MIR181B2,GPR21,TRAF1,SNORD141B,NUP188,GBGT1,RXRA,OLFM1,SCAI,ZBTB34,SETX,SLC25A25-AS1,SURF2,MIR3960,ST6GALNAC6,LINC00963,OR1Q1,ZBTB26,MIR181A2HG,FUBP3,FIBCD1,VAV2,FPGS,LINC00094,MIR600,LHX2,OBP2A,BRINP1,PBX3,COL5A1,DBH-AS1,ATP6V1G1,STXBP1,SH2D3C,ALAD,SNORD36A,NR5A1,LOC100505478,DOLPP1,FCN1,LOC101929331,MIR601,C9orf43,WDR5,SNORD90,FAM102A,LAMC3,AMBP,BARHL1,CDK5RAP2,MIR3689F,HDHD3,C9orf106,LRRC8A,CELP,ARPC5L,NUP214,MIR6855,SNORD24,GOLGA1,ASTN2,DEC1,STRBP,SNORD141A,DAB2IP,SH3GLB2,PPP1R26,CEL,WDR31,UCK1,TTC16,C9orf9,EXOSC2,HMCN2,MIR4478,SNORD36C,C9orf78,LMX1B,LOC100129034,ADAMTSL2,C5,RPL35,MORN5,STOM,CRB2,PPP1R26-AS1,MED27,RGS3,MIR4669,TOR2A,MIR3689C,AK1,CDC26,IER5L,FCN2,DOLK,GFI1B,STKLD1,SWI5,FKBP15,LCN1,PTGES,LINC01503,PTGS1,POLE3,GSN,SARDH,BSPRY,MIR3154,MIR600HG,GLT6D1,DBH,C9orf116,TRUB2,OR1L4,WDR38,NTNG2,LHX6,NTMT1,PRDM12,LINC00474,GLE1,C9orf50,ODF2,GGTA1P,SOHLH1,SNORD62A,TTF1,OR1L1,RBM18,NR6A1,PSMB7,MIR3911,POMT1,CIZ1,MIR219B,LOC100288842,MIR6856,OR1L3,WDR34,ANGPTL2,RABEPK,TOR1B,FAM129B,TLR4,CCBL1,GTF3C5,CFAP77,TTLL11,MIR3689D2,RNF183,PAEP,ST6GALNAC4,RPL7A,PLPP7,TNC,LOC101928775,LOC101928525,ADAMTS13,SNORD36B,MIR199B,BRD3,TNFSF15,RALGPS1,NAIF1,HSPA5,LRSAM1,URM1,MIR6877,FAM163B,PRPF4,QRFP,LOC101928797,CNTRL,PTGES2-AS1,MRPS2,RC3H2,ASB6,GPR107,OBP2B,MIR548AW,ZNF79,RPL12,RALGDS,LOC401557,MIR3689A,NDUFA8,AKNA,AK8,SURF4,PKN3,TOR1A,USP20,SNORA70C,TNFSF8,DDX31,C9orf114,ZBTB43,OLFML2A,LINC01613,FAM73B,RAB14,TBC1D13,OR1J2,MAPKAP1,ZER1,GARNL3,MIR147A,ZDHHC12,SLC25A25,MIR3689D1,MVB12B,PHYHD1,TRIM32,GTF3C4,ABL1,MIR3689B,PDCL,RNU6ATAC,KIF12,MRRF,PAPPA-AS1,MIR3689E,LINC01502,OR5C1,ABO,C9orf91,PPP6C,PSMD5,OR1L8,GSN-AS1,DPM2	3	gain
1892	CGTE_24	9	140391507	140395360	9q34.3	PNPLA7	9	gain
1893	CGTE_24	9	140396086	141016420	9q34.3	MIR602,LOC105376331,EHMT1,EHMT1-IT1,PNPLA7,LOC101928786,CACNA1B,ZMYND19,DPH7,ARRDC1-AS1,ARRDC1,MRPL41,LOC100133077	3	gain
1894	CGTE_24	10	72300840	72520615	10q22.1	PRF1,PALD1,ADAMTS14	3	gain
1895	CGTE_24	10	94820772	94834155	10q23.33	CYP26A1,CYP26C1	0	loss
1896	CGTE_24	10	134648057	134649786	10q26.3	CFAP46	10	gain
1897	CGTE_24	10	134650296	134663839	10q26.3	CFAP46	0	loss
1898	CGTE_24	11	64072992	64085072	11q13.1	TRMT112,ESRRA	5	gain
1899	CGTE_24	11	64085711	64139065	11q13.1	RPS6KA4,PRDX5,CCDC88B,MIR1237,MIR7155	0	loss
1900	CGTE_24	12	208094	966442	12p13.33	IQSEC3,KDM5A,CCDC77,B4GALNT3,WNK1,SLC6A12,LOC101929384,LOC574538,LOC102723544,NINJ2,LOC100049716,SLC6A13	1	loss
1901	CGTE_24	12	968365	1291224	12p13.33	ERC1,RAD52,WNK1	5	gain

1902	CGTE_24	12	1292423	4870374	12p13.32-p13.33	NDUFA9,PRMT8,FOXM1,CRACR2A,TIGAR,ADIPOR2,CCND2-AS1,TULP3,CACNA1C-AS4,ITFG2,ERC1,FGF6,LINC00940,CACNA1C-IT2,GALNT8,LOC100507424,CACNA1C-AS1,LOC283440,FBXL14,LRTM2,C12orf4,CACNA1C,LINC00942,RHNO1,AKAP3,TEAD4,LOC101929549,PARP11,CCND2,RAD51AP1,FKBP4,MIR3649,FGF23,CACNA1C-IT3,NRIP2,DYRK4,DCP1B,CACNA1C-AS2,TSPAN9,CACNA2D4,WNT5B,THCAT155	4	gain
1903	CGTE_24	12	4872346	17142515	12p12.3-p13.32	LOC101928100,PRH2,RBP5,ACRBP,LINC01489,TNFRSF1A,P3H3,A2M,EMP1,KLRB1,SMIM10L1,SMCO3,EPS8,LPCAT3,GSG1,LMO3,MFAP5,MIR141,KCNA1,PHC1,H2AFJ,YBX3,GPR162,RPL13AP20,TAS2R9,PRB1,TAS2R50,TAS2R46,KLRAP1,LOC101930452,C3AR1,KLRF1,CD69,LOC100506159,MAGOHB,PTPN6,APOBEC1,NTF3,TAS2R14,TAS2R13,LOC101928162,PZP,CLEC6A,ANO2,CLEC2B,NANOG,C1S,ART4,DUSP16,LINC00612,M6PR,LOC101929584,RIMKLB,NOP2,BCL2L14,TAPBPL,DDX47,SLC15A5,CLEC2A,CD163,HTR7P1,SCNN1A,FAM90A1,C12orf57,KLRK1,KCNA5,TAS2R43,TAS2R20,ARHGDIB,WBP11,DDX12P,MGP,CD9,STYK1,TAS2R7,CDCA3,TAS2R42,CD4,PIANP,RPL13P5,TAS2R8,LINC01252,SPSB2,KLRF2,MRPL51,FAM66C,MIR614,KLRC2,CLEC1B,PTPRO,NCAPD2,FAM234B,DPPA3,C1R,C12orf60,KLRC1,OLR1,LOC374443,STRAP,CLSTN3,MIR1244-3,A2MP1,PRH1,FOXJ2,CLEC12A,TAS2R31,MIR1244-2,KLRG1,CLEC7A,GDF3,PRH1-TAS2R14,TMEM52B,VAMP1,A2M-AS1,CDKN1B,VWF,FAM86FP,MGST1,C1RL-AS1,POU5F1P3,ZNF384,ACSM4,COPS7A,ATF7IP,GNB3,RERG-AS1,GAPDH,CLEC4E,MIR7641-2,BORCS5,CHD4,LOH12CR2,NECAP1,LOC101928030,GPRC5A,TAS2R30,CLEC4D,DERA,SLC2A14,C1RL,KCNA6,CD27,GPRC5D,GALNT8,MIR200C,SCARNA12,PLBD1,PTMS,DSTNP2,ATN1,APOLD1,GRIN2B,LOC642846,CLEC12B,PRB3,CLECL1,TPI1,KLRC4,LOC102724020,LRP6,HIST4H4,ENO2,PLEKHG6,AICDA,PRB2,PLBD1-AS1,PRB4,CD27-AS1,SCARNA11,MLF2,CREBL2,GUCY2C,A2ML1,NANOGNB,MIR1244-1,CLEC9A,CLEC4A,SLC2A3,GABARAPL1,TAS2R19,CD163L1,SCARNA10,KLRC3,LAG3,PHB2,CLEC2D,LINC00937,LOC101927905,RERG,LTBR,HEBP1,CLEC4C,IFFO1,PDE6H,TAS2R10,LINC01559,MANSC1,LPAR5,USP5,ZNF705A,KLRD1,CLEC1A,PRR4,MIR1244-4,ETV6,SKP1P2,ING4,LINC00987,LOC100506314,PEX5,GPR19,LRRC23,ERP27,MIR613,EMG1,KLRC4-KLRK1,PRH1-PRR4	3	gain
1904	CGTE_24	12	131623493	131687373	12q24.33	LINC01257,ADGRD1	11	gain
1905	CGTE_24	12	132195840	133811188	12q24.33	LRCOL1,LOC101928530,ZNF605,EP400,NOC4L,GOLGA3,CHFR,LOC101928597,ULK1,DDX51,EP400NL,ZNF84,ANHX,PGAM5,LOC100130238,LOC101928416,MMP17,PXMP2,ZNF140,P2RX2,ZNF268,ZNF891,POLE,SNORA49,ZNF26,GALNT9,MIR6763,PUS1,ZNF10,FBRSL1,SFSWAP,ANKLE2	1	loss

1906	CGTE_24	13	19600474	38933561	13q13.3-q13.1	ZMYM5,GTF3A,SKA3,MTIF3,CENPJ,PDX1-AS1,MIPEP,ATP5EP2,MIR4499,GJB6,MPHOSPH8,CCDC169,RNF17,GJA3,SPG20-AS1,LINC00426,URAD,LATS2,BASP1P1,LINC00547,GPR12,HSPH1,LINC00457,FLT1,LINC01058,GJB2,SPATA13-AS1,USP12,GSX1,LINC00571,RFC3,SACS-AS1,CDX2,POSTN,MIPEPP3,EEF1DP3,EXOSC8,NUP58,STARD13,ANKRD20A19P,TRPC4,SUPT20H,B3GLCT,LINC00412,N4BP2L1,LINC01048,MTMR6,SMAD9,SOHLH2,MEDAG,FRY,MRPL57,POMP,SGCG,LINC00327,AMER2,FLT3,SPATA13,LINC00566,LINC00572,SNORD102,ZAR1L,N6AMT2,SAP18,MIR548F5,TEX26-AS1,LNX2,SHISA2,SNORA27,LINC01046,UBL3,SERTM1,ATP12A,NBEA,N4BP2L2,N4BP2L2-IT2,TPTE2P6,LINC00350,LINC00424,LINC00423,LINC00539,PSPC1,ATP8A2,LOC101928697,ZDHHC20,SACS,LINC01053,BRCA2,CDK8,RXFP2,PAN3,MAB21L1,CRYL1,MINOS1P1,UFM1,LINC00540,ALG5,DCLK1,PDS5B,MTUS2,USP12-AS2,LINC00297,SPG20,LINC00365,C1QTNF9B,C1QTNF9B-AS1,LINC00445,ALOX5AP,IL17D,TNFRSF19,RPL21P28,MICU2,STARD13-AS,PABPC3,SLC7A1,PARP4,MTUS2-AS1,HMGB1,RPL21,LINC00463,TPTE2P1,CCNA1,PDX1,XPO4,ZMYM2,TPTE2,LINC00367,LINC00544,CSNK1A1L,RNF6,KL,SLC46A3,FRY-AS1,LINC01072,LINC00545,MIR2276,IFT88,WASF3,FGF9,CCDC169-SOHLH2,PAN3-AS1,KATNAL1,LINC00421,POLR1D,ANKRD26P3,RFXAP,USP12-AS1,C1QTNF9,RASL11A,TEX26,TUBA3C,LINC00398,USPL1	3	gain
1907	CGTE_24	13	38934079	39262135	13q13.3	LINC00366,UFM1,FREM2,LINC00437	0	loss

1908	CGTE_24	13	39262137	96675996	13q14.12-q13.3	MIR3168,LINC00400,LINC00563,AKAP11,LINC00434,MED4-AS1,MIR548X2,DZIP1,TRIM13,NAA16,FBXL3,GPC6,DLEU7-AS1,GPC6-AS2,LINC01065,LOC105370306,LINC00379,LACC1,SMIM2,RBM26,COG6,DIS3,RGCC,MIR8079,LINC00333,PCDH9-AS2,LMO7,LINC00375,DIAPH3,TBC1D4,KIAA0226L,KCTD4,LPAR6,LINC01198,LINC00380,PIBF1,CLDN10-AS1,INTS6,SUGT1,SOX21,MIR15A,SLAIN1,OLFM4,LHFP,GPC5-AS2,SMIM2-AS1,GPC5-AS1,LECT1,UGGT2,ZC3H13,MIR20A,PCDH8,STOML3,UTP14C,LINC00381,INTS6-AS1,MIR759,TPT1,MIR4305,RNASEH2B,LINC00332,LRRC63,LINC00440,TNFSF11,NEK5,LINC00448,ITM2B,LINC00559,LINC00557,SMIM2-IT1,NUDT15,LINC01078,EDNRB,LMO7DN-IT1,SLC25A30,KBTBD6,ABCC4,ATP7B,PRR20D,NEK3,PROSER1,PCDH9-AS3,MIR3665,TDRD3,LINC00564,VWA8,LINC01052,TGDS,MIR19A,LINC01047,PCDH9,CTAGE11P,SNORA31,MIR3613,LINC00374,IRG1,LINC00433,CAB39L,SLC25A30-AS1,NUFIP1,CTAGE10P,WDFY2,ESD,LINC00284,OR7E37P,DNAJC3-AS1,LINC00358,LINC01055,DGKH,SERP2,SLITRK5,COMMD6,SPERT,MIR16-1,RNF219-AS1,SPRY2,FOXO1,LINC00383,RB1,FAM216B,PHF11,LINC00353,LOC101929657,LINC00363,MZT1,PRR20C,MIR5693,DLEU1,NDFIP2,LINC00331,MIR17,MTRF1,VWA8-AS1,LINC00364,MLNR,SCEL-AS1,PCDH20,LINC00430,MRPS31P5,BTF3P11,LINC01049,RCBTB1,LINC00282,DNAJC15,EDNRB-AS1,LOC101929140,GPALPP1,TPTE2P3,DNAJC3,POU4F1,SUCLA2,CPB2-AS1,DCT,LINC01069,HTR2A-AS1,LINC00446,LINC01075,CYSLTR2,ELF1,RNASEH2B-AS1,LINC00371,LINC00377,LINC00428,LINC00351,COG3,SLITRK1,LOC101927284,CPB2,TPT1-AS1,MIR3169,CLN5,LINC01068,CCDC122,LINC00548,LINC01050,MIR1297,LINC00441,KCTD12,EPSTI1,WBP4,MYCBP2-AS1,SIAH3,FAM124A,DLEU2,VPS36,LINC00397,RBM26-AS1,NHLRC3,ENOX1,MIR4704,LMO7DN,PRR20E,LCP1,EBPL,MIR622,KLF12,SCEL,SNORA107,LINC00410,FNDC3A,CLDN10,DACH1,LINC00458,DLEU1-AS1,MIR17HG,LINC00390,TSC22D1-AS1,MIR320D1,MIR19B1,ARL11,KPNA3,SLITRK6,MIR18A,LOC101929259,PCDH9-AS4,SETDB2,LINC00392,MIR5007,DIAPH3-AS1,LINC00382,THSD1,DHRS12,LINC01040,LOC103191607,ATXN8OS,TUSC8,LINC00376,ST13P4,SPRYD7,LINC00347,SERPINE3,TPTE2P5,KCNRG,CCDC70,LINC00558,UCHL3,MIR4500,GUCY1B2,LINC01080,SUGT1P3,HTR2A,MIR621,RNF219,MIR92A1,LMO7-AS1,TSC22D1,LINC00462,NDFIP2-AS1,CDADC1,LRCH1,LINC00348,LOC102723968,LINC00330,ALG11,LINC00562,BORA,MYCBP2,PRR20B,CKAP2,KLHL1,GPC5,LINC00395,LOC101926897,LINC00378,MRPS31,RCBTB2,PRR20A,LOC100129307,MED4,KBTBD7,SLC25A15,PCDH17,GTF2F2,FREM2,LOC101927248,GPR180,LINC00550,HNRNPA1L2,ENOX1-AS2,KLF5,SOX21-AS1,LINC00598,GPC6-AS1,MIR4500HG,DLEU7,OR7E156P,LINC01038,ERICH6B,MIR5006,MIR4703,DIAPH3-AS2	3	gain
1909	CGTE_24	13	96684081	96743893	13q32.1	HS6ST3,UGGT2	7	gain
1910	CGTE_24	13	97079372	99667944	13q32.3-q32.2	IPO5,LINC00456,STK24,LINC00359,HS6ST3,MBNL2,OXGR1,DOCK9,RAP2A,RNF113B,SLC15A1,MIR3170,MIR4501,FARP1,DOCK9-AS1	3	gain
1911	CGTE_24	13	99738452	99853788	13q32.3	DOCK9,DOCK9-AS2,UBAC2-AS1,UBAC2	1576	gain

1912	CGTE_24	13	99886692	111102557	13q32.3-q33.1	LINC01309,SLC10A2,GGACT,ARGLU1,PCCA,ZIC2,FAM155A,EFNB2,MIR4705,COL4A1,MIR4306,UBAC2,FGF14-AS1,MIR548AN,LINC00449,LOC101927437,NALCN-AS1,METTL21EP,KDELC1,LINC00343,ABHD13,MYO16,FGF14-IT1,LINC00460,METTL21C,MIR2681,MIR1267,MIR623,LIG4,FAM155A-IT1,NALCN,LINC00676,GPR183,PCCA-AS1,CLYBL-AS1,CLYBL-AS2,FKSG29,LINC00554,LINC01039,LINC00399,TPP2,CCDC168,CLYBL,DAOA-AS1,LINC00443,ZIC5,COL4A2,TM9SF2,LINC01232,BIVM,TEX30,LOC105370333,IRS2,MIR548AS,LINC00551,ITGBL1,DAOA,TMTC4,LINC00396,MYO16-AS1,ERCC5,LINC00411,BIVM-ERCC5,MIR8073,FGF14,GPR18,FGF14-AS2,TNFSF13B	3	gain
1913	CGTE_24	13	111102577	111111243	13q34	COL4A2	5	gain
1914	CGTE_24	13	111114344	115091802	13q34	ARHGEF7-AS2,TMEM255B,ATP11A,TUBGCP3,F7,ARHGEF7,GAS6-AS2,LINC00368,COL4A2-AS1,UPF3A,LINC00346,TFDP1,F10,ING1,ATP11AUN,TEX29,LINC00403,COL4A2,CHAMP1,ANKRD10,ATP4B,LINC00431,GRK1,GAS6,LINC01070,ARHGEF7-AS1,LOC101060553,LINC00354,DCUN1D2,SPACA7,PROZ,MCF2L,RASA3,MIR548AR,MCF2L-AS1,ADPRHL1,LOC101928730,LINC01044,LINC00552,LAMP1,CDC16,RAB20,LINC00452,SOX1,MIR4502,ATP11A-AS1,GRTP1-AS1,GAS6-AS1,CARS2,TMCO3,F10-AS1,PCID2,LOC101928841,CUL4A,CARKD,GRTP1,LINC00565,MIR8075	3	gain
1915	CGTE_24	15	42145403	42152991	15q15.1	SPTBN5	0	loss
1916	CGTE_24	16	97450	2088475	16p13.3	HN1L,CCDC154,TPSB2,PRR35,SNORA78,TMEM8A,SNHG9,HBA1,LINC00254,BAIAP3,PIGQ,UBE2I,NPRL3,CCDC78,RGS11,AXIN1,CLCN7,SSTR5-AS1,HBA2,LOC100134368,HBM,MIR3177,SNRNP25,C16orf13,NPW,MIR3176,HS3ST6,MIR5587,SYNGR3,PTX4,IGFALS,C1QTNF8,NOXO1,RPL3L,TSR3,SPSB3,NUBP2,HBZ,TBL3,FAHD1,LUC7L,LMF1-AS1,C16orf91,UNKL,EME2,FAM195A,MSRB1,RHOT2,SNORA10,MIR662,MSLN,MEIOB,PDIA2,TPSAB1,LMF1,RNF151,TPSG1,CACNA1H,HAGH,CHTF18,RHBDL1,ZNF598,NHLRC4,POLR3K,STUB1,GFER,RPUSD1,FAM173A,WDR24,GNPTG,RPS2,SSTR5,HAGHL,ARHGDIG,NME3,TELO2,TPSD1,RAB11FIP3,NME4,LINC00235,SOX8,FBXL16,JMJD8,CAPN15,MAPK8IP3,WFIKKN1,NARFL,DECR2,MRPS34,SLC9A3R2,HBQ1,MPG,WDR90,RAB40C,FAM234A,GNG13,TMEM204,NDUFB10,CRAMP1,RHBDF1,MRPL28,PRR25,SNORA64,IFT140,METRN	1	loss
1917	CGTE_24	16	57994343	57996956	16q21	CNGB1	6	gain
1918	CGTE_24	16	88520007	88552561	16q24.2	MIR5189,ZFPM1	13	gain
1919	CGTE_24	16	88555355	88593422	16q24.2	ZFPM1	0	loss
1920	CGTE_24	16	88594349	88598319	16q24.2	ZFPM1	1409	gain
1921	CGTE_24	16	88598343	88873882	16q24.2-q24.3	IL17C,SNAI3,MVD,MIR4722,PIEZO1,ZFPM1,CYBA,RNF166,LOC100289580,CDT1,ZC3H18,LOC339059,CTU2,SNAI3-AS1	0	loss
1922	CGTE_24	16	88874525	88877007	16q24.3	APRT,CDT1	6	gain

1923	CGTE_24	17	6733602	21320014	17p13.1-p11.2	PIK3R5,ALOX12B,ZSWIM7,MYO15A,ALOX15B,TMEM95,DHRS7B,CDRT8,FBXW10,CDRT15L2,SNORD49A,TRIM16L,COX10,FXR2,TMEM256-PLSCR3,TVP23C,MYOCD,SPEM1,SNORD49B,LOC100128288,TBC1D26,CYB5D1,CDRT15,SAT2,SLC2A4,WRAP53,LOC388436,USP32P1,TMEM220,LOC101928567,STX8,SLC16A11,TVP23C-CDRT4,USP22,RCVRN,SNORD65,MIEF2,FLJ35934,MIR6883,NDEL1,RASD1,LOC100128006,LGALS9B,LGALS9C,GUCY2D,LOC101928266,ELAC2,FAM106B,GPS2,MIR1288,LOC284023,KCNJ12,TRAPPC1,TRPV2,CCDC144NL,CFAP52,FAM83G,AKAP10,B9D1,CCDC42,SCARNA21,EIF5A,PIGL,DNAH2,NTN1,CCDC144NL-AS1,MIR1180,EIF4A1,MIR744,LINC01563,HES7,PFAS,LINC00675,MGC12916,MPDU1,DVL2,TNFSF12,TVP23B,GLP2R,CLDN7,TEKT3,FGF11,ODF4,SNORA59B,ACADVL,MFAP4,ALDH3A2,SLC47A1,TNFRSF13B,C17orf49,LINC00324,RNF222,FAM106A,MIR4314,MIR195,SNORA59A,PHF23,ARHGAP44,TNK1,GRAPL,MAGOH2P,LRRC75A-AS1,CENPV,MIR497HG,CHRNB1,MIR324,PLSCR3,HS3ST3B1,GRAP,FLCN,RPL26,GAS7,KRT16P2,PIRT,CTDNEP1,CDRT15P1,MYH13,ZNF18,ZBTB4,CDRT7,SLC25A35,UBB,CNTROB,CDRT15P2,ASGR2,TRIM16,MIR1269B,KCNJ18,ULK2,USP32P2,LLGL1,KCNAB3,HS3ST3A1,SLC5A10,SPECC1,KRT17P5,MIR4521,SLC16A13,FLII,MAP2K4,ALKBH5,ALOX12,MAP2K3,PMP22,ADORA2B,MYH8,VAMP2,AURKB,CCDC144A,RAI1-AS1,CDRT4,CTC1,CCDC144CP,TBC1D28,POLR2A,TOM1L2,NAA38,RAI1,ACAP1,KRT16P3,TNFSF13,MEIS3P1,GSG1L2,ALDH3A1,RNASEK,NCOR1,CLEC10A,RNF112,SOX15,ALOX12P2,RPL29P2,LRRC75A,FOXO3B,MAPK7,TTC19,PIK3R6,ASGR1,MYH3,SLC35G6,ZNF286B,SNORD10,TNFSF12-TNFSF13,SLC47A2,SENP3,PRPSAP2,SNORA48,YBX2,TMEM88,PEMT,NATD1,MYH2,EPN2-AS1,TMEM256,NEURL4,MYH1,SENP3-EIF4A1,LOC79999,PLD6,ZNF287,ADPRM,MIR6778,LOC339260,BORCS6,ALOXE3,SCO1,COPS3,CDRT1,SMCR5,MFSD6L,NLGN2,DHRS7C,ZNF286A,C17orf74,ELP5,FAM106CP,KCTD11,TMEM220-AS1,LINC00670,SREBF1,DNAH9,MYH4,DRC3,GABARAP,LOC100287072,EPN2-IT1,SNORA67,EVPLL,ATP1B2,MIR497,GID4,SPDYE4,MED9,SHISA6,MYHAS,CHD3,PER1,ATPAF2,BCL6B,SHBG,ARHGEF15,EPN2,TEKT1,SMCR2,TMEM102,MYH10,SHMT1,LOC101928418,RNASEK-C17orf49,DRG2,TMEM107,EFNB3,MPRIP,SMCR8,USP43,DLG4,CD68,CCDC144B,RANGRF,KRBA2,MIR6777,TP53,TMEM11,TOP3A,ALOX12-AS1,KDM6B,MIR33B,COX10-AS1,NT5M,MIR4731,KRT16P1,ZNF624	1	loss
1924	CGTE_24	17	21438538	21826324	17p11.2	C17orf51,FAM27L	4	gain
1925	CGTE_24	17	79092145	79508104	17q25.3	FAAP100,BAHCC1,MIR3065,SLC38A10,FSCN2,LOC100130370,ACTG1,MIR3186,ENTHD2,AATK-AS1,MIR1250,MIR338,TMEM105,AATK,MIR4740,C17orf89,LINC00482,MIR657,CEP131	0	loss
1926	CGTE_24	17	79508329	79512882	17q25.3	FAAP100	10	gain
1927	CGTE_24	18	44772780	44775202	18q21.1	SKOR2	17	gain
1928	CGTE_24	18	47800519	47802343	18q21.1	MBD1	9	gain
1929	CGTE_24	18	47811305	47813259	18q21.1	CXXC1	4	gain
1930	CGTE_24	18	66509274	66513765	18q22.1	CCDC102B	5	gain
1931	CGTE_24	18	77160324	77170736	18q23	NFATC1	11	gain

1932	CGTE_24	19	281411	1980402	19p13.3	CDC34,MBD3,MADCAM1,NDUFS7,SHC2,HMHA1,ABHD17A,R3HDM4,TMEM259,RPS15,CBARP,PTBP1,RNU6-2,MIDN,GZMM,PRSS57,POLR2E,UQCR11,PLPPR3,REEP6,MIR4745,AZU1,ARID3A,POLRMT,KLF16,PLPP2,REXO1,ATP5D,CIRBP,GAMT,FSTL3,ABCA7,GRIN3B,PALM,APC2,CFD,THEG,LOC100288123,ODF3L2,C19orf25,RNF126,C2CD4C,FGF22,MUM1,CSNK1G2-AS1,MISP,ONECUT3,KISS1R,TCF3,MIR3187,WDR18,EFNA2,MED16,PCSK4,PLK5,ADAMTSL5,CNN2,MIR1909,MIER2,DAZAP1,CSNK1G2,C19orf24,BSG,ATP8B3,STK11,GPX4,SBNO2,ELANE,HCN2,CIRBP-AS1,ADAT3,TPGS1,PRTN3,MEX3D,SCAMP4	0	loss
1933	CGTE_24	19	1986461	7128962	19p13.2-p13.3	NCLN,TMIGD2,GNA15,SH2D3A,AMH,VMAC,PLIN5,STAP2,MIR4321,SAFB,LSM7,INSR,THOP1,FUT3,ARRDC5,MIR1268A,NDUFA11,PIP5K1C,MIR3940,SLC39A3,CD70,TRIP10,CCDC94,EBI3,ZNF555,UBXN6,TMPRSS9,ZNF556,PLEKHJ1,C19orf35,MFSD12,HSD11B1L,SHD,TUBB4A,PLIN4,CRB3,MBD3L4,MATK,C19orf70,MIR6885,LOC100996351,LOC101928844,SLC25A23,FLJ25758,TNFSF9,CACTIN-AS1,HMG20B,LONP1,SF3A2,SAFB2,MIR7-3HG,ZNF57,MAP2K2,CACTIN,ZFR2,MIR4747,CREB3L3,S1PR4,ACER1,SIRT6,SLC25A41,LRG1,MRPL54,MOB3A,GNG7,MIR4746,LMNB2,PIAS4,SEMA6B,TNFSF14,UHRF1,HDGFRP2,LINGO3,SMIM24,TNFAIP8L1,ZNF557,DAPK3,GIPC3,TICAM1,DENND1C,DPP9,DOHH,GADD45B,NRTN,GTF2F1,C3,FSD1,FUT5,RPL36,TLE2,PRR22,TBXA2R,MIR7850,TINCR,PLIN3,SPPL2B,BTBD2,OAZ1,FZR1,RAX2,EEF2,MBD3L2,MYDGF,RANBP3,KDM4B,CHAF1A,ADGRE1,MIR6791,PSPN,CELF5,ADGRE4P,ZNF554,KHSRP,TJP3,SGTA,ALKBH7,RFX2,JSRP1,DUS3L,CATSPERD,LOC100128568,ZBTB7A,TIMM13,AES,GNA11,MLLT1,SNORD37,MIR7-3,ZNRF4,MIR6789,C19orf71,CAPS,NFIC,NMRK2,MBD3L3,MKNK2,CLPP,DPP9-AS1,ATCAY,IZUMO4,MIR1227,TLE6,MIR637,ACSBG2,ANKRD24,AP3D1,VAV1,APBA3,PTPRS,SH3GL1,MPND,MIR7108,FUT6,GPR108,DOT1L,DIRAS1,MBD3L5,ZNF77,FEM1A,MIR6790	1	loss

1934	CGTE_24	19	7132089	16301692	19p13.13-p13.12	WIZ,PLPPR2,SYDE1,ZNF177,DNASE2,ZNF564,FDX1L,ZNF442,ARHGEF18,ADGRL1,FCER2,ICAM4,CYP4F11,DAND5,TYK2,SLC44A2,ZNF443,C19orf43,CARM1,DNAJB1,ZNF266,OR7E24,AKAP8,MIR1199,LOC105372280,DNM2,OLFM2,ZNF561,WDR83OS,LDLR,KEAP1,DCAF15,OR1I1,GIPC1,DOCK6,MBD3L1,COL5A3,PRR36,PRDX2,ZNF317,MIR639,MUC16,AP1M2,RAB11B-AS1,ZNF559,MIR638,ZNF358,TRMT1,ADAMTS10,CD209,SNORD41,RAB3D,DHPS,DNMT1,CASP14,BRD4,QTRT1,AKAP8L,CALR,RAVER1,SLC1A6,HSH2D,ZGLP1,OR7D4,CYP4F24P,OR10H4,SWSAP1,MAST1,C19orf53,ICAM1,ZNF812,MAN2B1,P2RY11,LOC100507373,MIR1238,ICAM5,MIR27A,ZNF333,TGFBR3L,ZNF625,ZNF136,MIR181D,RAB8A,OR7G2,CLEC4M,C19orf66,C19orf52,ZNF20,OR7A10,TRAPPC5,ADGRE2,OR7C1,HNRNPM,ELAVL1,ZNF791,NANOS3,PET100,ANGPTL4,S1PR5,PRKACA,CYP4F8,ZNF121,PRKCSH,FBXW9,FBXL12,EPHX3,RFX1,ZNF788,ZNF846,NDUFB7,FBN3,GADD45GIP1,XAB2,ZNF878,ZNF490,ADGRE3,RPS28,SNORA104,MIR4999,LINC00905,CCDC105,PEX11G,ELAVL3,LOC100128573,ZNF763,CYP4F3,PIN1,ZNF439,OR1M1,ZNF833P,ZNF562,RASAL3,RAD23A,INSR,CLEC4G,UBL5,STXBP2,ZNF699,TECR,TMED1,MIR24-2,MIR199A1,MIR6793,WDR83,HOOK2,CLEC17A,RLN3,NFIX,C19orf45,KLF1,NACC1,NOTCH3,RDH8,BEST2,MCOLN1,SNORD105B,KANK3,ADGRE5,OR7A5,UCA1,ZNF653,CC2D1A,MIR6792,ANGPTL8,ZNF414,KRI1,NDUFA7,OR2Z1,CACNA1A,SAMD1,ZNF823,ZNF44,MAP2K7,ZNF625-ZNF20,ILF3-AS1,OR7A17,C19orf67,DDX39A,TNPO2,LYL1,MIR7974,PODNL1,ANGPTL6,C3P1,LOC102724279,TIMM44,MARCH2,CLEC4GP1,MRI1,ELOF1,ZNF433,IER2,LOC100289333,SNORD135,CDKN2D,RGL3,ZNF426,CIB3,SPC24,MRPL4,ASF1B,MIR1470,OR10H3,ZNF709,ZNF844,LRRC8E,MIR6886,S1PR2,ZNF560,CTXN1,ZNF700,ECSIT,MIR4748,PRAM1,CAMSAP3,OR10H5,CD320,CYP4F2,PGLYRP2,C19orf57,CYP4F12,CYP4F22,MIR5589,ZNF563,CCDC130,IL27RA,OR10H2,PNPLA6,ZNF69,PDE4A,SMARCA4,ZSWIM4,ZNF561-AS1,ATG4D,FAM32A,GCDH,RAB11B,ZNF559-ZNF177,EIF3G,ILVBL,ICAM3,RETN,C19orf38,SNAPC2,CDC37,TMEM205,JUNB,EVI5L,PPAN,MCEMP1,ZNF440,CCDC159,KANK2,CNN1,RNASEH2A,ACP5,CCDC151,ZNF441,MIR6794,TSPAN16,OR7G1,SNORD105,ZNF799,OR10H1,PTGER1,MIR4322,CERS4,PPAN-P2RY11,OR7D2,MIR1181,ZNF491,MIR6795,ASNA1,LINC00661,MIR181C,LOC113230,STX10,CCL25,ILF3,MIR23A,LYPLA2P2,FARSA,YIPF2,ZNF558,MYO1F,EPOR,ZNF627,SYCE2,MIR6515,RTBDN,PCP2,TPM4,OR7C2,ACTL9,PALM3,PKN1,LOC284454,OR7G3	3	gain

1935	CGTE_24	20	68259	14307008	20p12.1-p12.2	SNORD119,MRPS26,GNRH2,SIGLEC1,VPS16,SNORD57,UBOX5,MACROD2,TRIB3,MIR6869,SNPH,LINC00687,LINC01428,LRRN4,GPCPD1,LOC339593,LOC100505515,SOX12,PTPRA,PCNA-AS1,CDC25B,NDUFAF5,NSFL1C,SEL1L2,CRLS1,PRND,CHGB,LOC101929413,DEFB125,NOP56,MAVS,C20orf194,MIR6870,BMP2,FERMT1,SNORD86,SLX4IP,PLCB4,LINC00654,ADRA1D,LOC101929486,C20orf96,CENPB,SRXN1,PRNP,SDCBP2-AS1,PDYN,FKBP1A-SDCBP2,SIRPD,GFRA4,DEFB132,PANK2,JAG1,C20orf196,LOC388780,LOC101929312,PLCB1,SLC4A11,SIRPB1,MIR1292,CSNK2A1,HSPA12B,AP5S1,SLC23A2,LOC100289473,PROKR2,SLC52A3,RBCK1,SIRPG-AS1,SDCBP2,RNF24,MIR8062,MKKS,TMEM239,PRNT,TGM6,DEFB127,EBF4,LINC01433,LAMP5,DEFB126,FLRT3,SNRPB,CPXM1,FKBP1A,OXT,CDS2,SNORD110,DEFB129,LOC101929395,PSMF1,NRSN2-AS1,ITPA,SPEF1,FAM110A,IDH3B,LOC102606466,TMX4,ATRN,BTBD3,LOC101929207,C20orf27,SNORD56,TGM3,LOC643406,LAMP5-AS1,TMEM74B,SMOX,UBOX5-AS1,RSPO4,SPTLC3,C20orf141,PCNA,ESF1,SIRPB2,TMEM230,CASC20,TMC2,ISM1-AS1,DDRGK1,ISM1,LINC00658,TRMT6,TBC1D20,ZNF343,RASSF2,STK35,MIR103A2,PCED1A,SNORA51,DEFB128,C20orf202,HAO1,RAD21L1,SIRPG,NRSN2,MIR103B2,SIRPA,ADAM33,LOC101929125,PAK7,MCM8-AS1,LZTS3,SCRT2,LOC101929371,AVP,SNAP25,ANKEF1,TCF15,SNAP25-AS1,ZCCHC3,MCM8,FASTKD5,TASP1,ANGPT4	4	gain

1936	CGTE_24	20	14307011	60883807	20p11.21-q13.11	AAR2,RBM39,SYS1-DBNDD2,EDEM2,SPINT3,LOC339568,LOC100506384,LOC729296,PRELID3B,BPIFA1,DEFB121,BMP7,FRG1DP,NEURL2,DEFB119,PHF20,CDH22,PPP1R16B,RPRD1B,NAA20,RBM38,NPEPL1,ELMO2,BFSP1,NKX2-2,RBL1,CSRP2BP,PTPRT,ZNF337-AS1,ADA,MIR1289-1,E2F1,LOC101926935,OCSTAMP,ARHGAP40,BCL2L1,LINC00261,HM13-AS1,CST5,C20orf85,KIF16B,HNF4A-AS1,PCSK2,SNHG11,DNMT3B,CSTF1,FAM209B,SYS1,FAM217B,OTOR,LINC01620,DEFB115,SNORA71A,TTI1,ABALON,DEFB118,RBPJL,ZNF341,LINC00494,LINC01270,CTSZ,MIR3192,STK4,LOC101927932,GCNT7,CEBPB,C20orf24,CST9L,OSER1-AS1,DZANK1,EDN3,KIAA1755,LPIN3,FITM2,C20orf78,LINC01260,SNX21,NKX2-4,NCOA3,MIR6812,CRNKL1,MACROD2-AS1,C20orf173,ASIP,PKIG,LINC01441,DNTTIP1,LOC100134868,MIR4325,DSN1,MIR296,MIR4755,TOMM34,LINC01523,FAM83C-AS1,DEFB124,LINC01432,PAX1,ZSWIM3,DOK5,SPINT4,STK4-AS1,ENTPD6,CST11,CSE1L,LOC284798,AHCY,SNTA1,VSX1,DYNLRB1,POLR3F,SLC32A1,MIR4533,LINC01524,BPI,DDX27,TPX2,ZNF663P,TUBB1,ADIG,LINC00657,TSHZ2,ANKRD60,OSER1,MAP1LC3A,CHMP4B,TMEM189-UBE2V1,ZNF334,POFUT1,HM13,UQCC1,MIR646,PABPC1L,CDH26,APMAP,VSTM2L,RALY,TP53TG5,LOC101929698,CCM2L,ID1,WFDC5,SLC12A5,BPIFB3,ADRM1,LINC00028,ZHX3,FAM65C,FAM209A,FOXS1,EYA2,TGIF2-C20orf24,APCDD1L,FAM83C,TRPC4AP,PLTP,LINC00652,PLCG1-AS1,SNX5,GSS,NCOR1P1,MROH8,CST8,NOL4L,SSTR4,CST7,EPB41L1,SNHG17,TGIF2,PCIF1,ZFP64,AURKA,TGM2,LOC101929625,MIR646HG,RAE1,BPIFA3,SPAG4,UBE2C,ZFAS1,STX16,CD40,MIR499A,SNORA71D,SALL4,SUMO1P1,FLRT3,MTRNR2L3,ARFGEF2,ZBP1,LINC01431,MMP24,LOC101926889,SNAI1,NNAT,ACTL10,CTNNBL1,C20orf197,MIR3646,PLAGL2,FER1L4,FLJ33581,MC3R,DLGAP4,SS18L1,SNRPB2,LOC100506175,SCAND1,REM1,CDH4,TTPAL,HRH3,EPPIN,MANBAL,MIR3616,CST9,INSM1,MIR298,SLMO2-ATP5E,PXMP4,UBE2V1,BPIFB4,ERGIC3,ZNF337,GNAS,ACOT8,FOXA2,XRN2,OSBPL2,ZNFX1,DBNDD2,LOC100131496,SNORD12C,TSPY26P,GTSF1L,BCAS1,RAB22A,MLLT10P1,CPNE1,MIR645,LOC100506470,LOC284788,LINC00851,LOC149950,WFDC10A,PLCG1,DEFB123,MIR1257,MYBL2,NANP,CHD6,CST2,BANF2,MIR4532,RNF114,KCNB1,ATP9A,NFS1,PIGU,ACSS2,MIR3194,LINC00489,ATP5E,SDC4,NCOA5,BPIFB2,GHRH,SERINC3,GZF1,CST4,LINC01427,DUSP15,CTCFL,LINC01598,CSTL1,MIR3617,LINC01370,SCP2D1,FAM182A,LOC101929526,MAFB,SLA2,GGT7,MYLK2,TOX2,MTG2,MGME1,C20orf144,COMMD7,GGTLC1,CNBD2,CEP250,BPIFB6,HNF4A,MIR3195,MIR6871,ZNF217,PIGT,DEFB122,SNORA71E,PREX1,PYGB,FAM182B,LOC101927159,CST13P,RBBP9,IFT52,TFAP2C,PPP1R3D,WFDC13,SNORD17,KIZ-AS1,LINC01429,CTSA,LINC01273,CYP24A1,RALGAPA2,WFDC2,ZNF341-AS1,SEC23B,KIF3B,SAMHD1,ZSWIM1,SEMG2,PFDN4,THBD,BMP7-AS1,MIR3193,EIF2S2,MIR7641-2,SNORA71C,EIF6,SYCP2,MIR644A,KCNK15-AS1,PROCR,SPATA25,MIR663AHG,ZNF831,MIR499B,PET117,WFDC9,TTLL9,EMILIN3,LOC100287792,HCK,SPO11,CST3,GNAS-AS1,DHX35,LOC100270804,MIR4756,LINC00656,DEFB116,ADNP-AS1,ZNF133,MOCS3,SNORA71B,DPM1,LBP,SRC,LSM14B,NINL,HMGB3P1,SNORD12,ITCH,	3	gain
1937	CGTE_24	20	60884380	60910360	20q13.33	LAMA5,MIR4758	0	loss
1938	CGTE_24	20	60911223	60912710	20q13.33	LAMA5	14	gain
1939	CGTE_24	20	60912712	60937710	20q13.33	LAMA5,LAMA5-AS1	0	loss
1940	CGTE_24	20	60941793	61936919	20q13.33	MIR4326,GID8,NKAIN4,RBBP8NL,OGFR-AS1,MIR1-1,FLJ16779,GATA5,LINC00659,OGFR,SLCO4A1,BHLHE23,BIRC7,DIDO1,COL20A1,COL9A3,LOC63930,NTSR1,HAR1A,LINC01056,DPH3P1,CABLES2,MIR124-3,C20orf166-AS1,SLCO4A1-AS1,SLC17A9,MIR1-1HG,LAMA5,TCFL5,YTHDF1,MIR133A2,MRGBP,LINC00029,RPS21,HAR1B,ARFGAP1,MIR3196	3	gain

1941	CGTE_24	20	61937229	61940070	20q13.33	COL20A1	23	gain
1942	CGTE_24	20	61940584	62046404	20q13.33	CHRNA4,COL20A1,LOC100130587,KCNQ2	0	loss
1943	CGTE_24	20	62046409	62120512	20q13.33	KCNQ2,EEF1A2	6	gain
1944	CGTE_24	20	62121728	62222147	20q13.33	PPDPF,PTK6,EEF1A2,SRMS,HELZ2,GMEB2,C20orf195	0	loss
1945	CGTE_24	20	62223390	62905019	20q13.33	MIR941-2,MYT1,ZBTB46-AS1,ZGPAT,ARFRP1,TCEA2,DNAJC5,MIR941-3,STMN3,LOC100505771,NPBWR2,MIR1914,MIR941-4,TPD52L2,RTEL1,SOX18,SAMD10,ZBTB46,UCKL1-AS1,PCMTD2,LKAAEAR1,LIME1,MIR647,LINC00176,SLC2A4RG,GMEB2,RTEL1-TNFRSF6B,MIR941-1,PRPF6,UCKL1,TNFRSF6B,MIR6813,RGS19,ABHD16B,ZNF512B,MIR941-5,OPRL1	3	gain
1946	CGTE_24	21	45971009	45978279	21q22.3	TSPEAR,KRTAP10-2,KRTAP10-3	565	gain
1947	CGTE_24	21	46906607	47331095	21q22.3	SLC19A1,PCBP3,LOC100129027,COL18A1	0	loss
1948	CGTE_24	21	47558427	47602866	21q22.3	FTCD,SPATC1L	0	loss
1949	CGTE_24	22	23656744	23917406	22q11.23	BCR,LOC101929374,IGLL1,ZDHHC8P1,CES5AP1,LOC388882	15	gain
1950	CGTE_24	X	152864479	152937170	Xq28	FAM58A,DUSP9,PNCK,LOC105373383	4	gain
1951	CGTE_24	X	152937334	152938839	Xq28	PNCK	0	loss
1952	CGTE_24	X	152939442	152958189	Xq28	PNCK,SLC6A8	8	gain
1953	CGTE_24	X	152958246	153040877	Xq28	BCAP31,ABCD1,PLXNB3,SLC6A8	0	loss
1954	CGTE_24	X	153040982	153041924	Xq28	PLXNB3	8	gain
1955	CGTE_24	X	153677986	153690623	Xq28	FAM50A,MIR6858,PLXNA3	3	gain
1956	CGTE_24	Y	2655076	15505873	Yp11.2-Yq11.221	PCDH11Y,TSPY3,TTTY16,TTTY12,TTTY23B,TTTY2,SRY,GYG2P1,RBMY1A3P,LINC00280,TTTY1,TTTY21,TTTY20,UTY,USP9Y,TSPY4,TTTY15,TTTY8,TTTY21B,DDX3Y,TTTY19,AMELY,TTTY7B,TSPY2,TTTY2B,TGIF2LY,TTTY23,TTTY7,FAM197Y5P,TTTY22,RBMY3AP,TBL1Y,PRKY,TSPY1,RPS4Y1,ZFY,TTTY8B,TSPY10,TTTY18,TTTY1B,TTTY11,FAM197Y2P,LINC00278,TSPY8	0	loss
1957	CGTE_24	Y	15526398	20826588	Yq11.222-Yq11.221	FAM41AY1,NLGN4Y,VCY1B,TMSB4Y,FAM41AY2,VCY,HSFY2,XKRY2,HSFY1,TTTY9B,CDY2A,CDY2B,NLGN4Y-AS1,TTTY9A,FAM224A,UTY,XKRY,FAM224B	0	loss
1958	CGTE_24	Y	21154393	24460980	Yq11.222-Yq11.223	PRY,RBMY1D,PRORY,RBMY2EP,TTTY5,KDM5D,TTTY14,EIF1AY,TTTY6,RPS4Y2,RBMY1B,RBMY1F,RBMY1E,CD24,RBMY1J,LOC101929148,TTTY13,PRY2,RBMY1A1,TTTY10,TXLNGY,RBMY2FP,BCORP1,TTTY6B	0	loss
1959	CGTE_25	1	79356757	82456857	1p31.1	LOC101927412,ADGRL4,ADGRL2,LOC101927434	3	gain
1960	CGTE_25	1	101490748	104068999	1p21.1-p21.2	COL11A1,S1PR1,DNAJA1P5,LOC102606465,DPH5,LINC01307,LOC101928370,OLFM3,RNPC3,LOC101928436	3	gain
1961	CGTE_25	1	152006202	153233498	1q21.3	FLG,LCE1C,LCE2C,LCE3C,LOC101928009,LCE4A,LOC100131107,FLG2,LCE1A,SPRR2B,SPRR1B,SPRR2A,SPRR2F,HRNR,RPTN,SPRR2D,LCE3A,LCE1B,LCE3D,SMCP,SPRR3,LCE3B,S100A11,LCE1D,PRR9,SPRR4,KPRP,SPRR1A,LCE1E,LCE2A,SPRR2G,CRCT1,LCE2D,TCHHL1,SPRR2C,IVL,TCHH,LOR,LCE6A,SPRR2E,LCE2B,LCE3E,LCE5A,FLG-AS1,CRNN,LELP1,LCE1F,C1orf68	3	gain
1962	CGTE_25	1	156937804	158817855	1q23.1	CD1E,OR6K2,CYCSP52,OR6Y1,CD1A,OR10K2,OR10R2,OR10T2,ETV3,OR10Z1,MNDA,OR6K3,OR6K6,LOC646268,CD1D,ETV3L,FCRL5,OR6P1,OR10X1,FCRL3,CD5L,CD1B,FCRL2,FCRL1,OR10K1,OR6N2,KIRREL,OR6N1,SPTA1,ARHGEF11,FCRL4,CD1C	3	gain
1963	CGTE_25	1	185878471	200311913	1q32.1-q31.2	CFH,DENND1B,CRB1,PRG4,LINC01036,CDC73,LOC440704,ATP6V1G3,NEK7,LHX9,MIR181A1HG,ZBTB41,F13B,LOC102724919,RGS21,ASPM,NR5A2,LINC01031,TROVE2,LINC01222,CFHR3,C1orf53,UCHL5,BRINP3,C1orf27,MIR4426,LINC01037,LINC01221,KCNT2,RGS2,RGS1,CFHR1,TPR,HMCN1,MIR1278,PTPRC,B3GALT2,MIR4735,RGS18,RGS13,LINC01032,PLA2G4A,CFHR5,PDC,CFHR2,LINC01351,LINC00862,PACERR,OCLM,MIR181A1,CFHR4,GLRX2,MIR181B1,MIR548F1,PTGS2	3	gain

1964	CGTE_25	1	215259719	217955692	1q41	SPATA17,SPATA17-AS1,KCNK2,USH2A,GPATCH2,ESRRG,KCTD3,LOC102723833	3	gain
1965	CGTE_25	1	247614360	249212637	1q44	GCSAML,OR2T29,OR2M3,OR2T10,OR2C3,OR2T6,OR2T11,TRIM58,OR2M4,OR2T2,OR13G1,MIR3124,ZNF692,OR14A16,OR2G2,OR2T5,OR1C1,OR2T12,OR2W3,OR2L5,OR2B11,OR14C36,OR2M1P,LYPD8,OR11L1,OR2W5,OR2L8,OR2L3,OR2G6,OR2T8,OR2G3,ZNF672,OR2T27,OR2M5,OR2L2,OR2L13,OR2T35,GCSAML-AS1,OR2T1,OR2T4,OR2M7,OR2AK2,OR2T34,SH3BP5L,OR2M2,OR6F1,LOC101928226,OR14I1,OR2T33,PGBD2,OR2L1P,OR2T3	3	gain
1966	CGTE_25	2	71597011	71623350	2p13.2	ZNF638	9	gain
1967	CGTE_25	2	75916149	83084264	2p12	REG3G,REG3A,SNAR-H,LOC100507201,LOC1720,MIR8080,REG1CP,GCFC2,CTNNA2,LRRTM4,LOC101927987,REG1A,MIR4264,LOC101927926,LRRTM1,LOC101927907,REG1B,LOC101927948,LOC101927967	3	gain
1968	CGTE_25	2	109086037	109089385	2q12.3	GCC2	12	gain
1969	CGTE_25	2	113033386	113082791	2q13	ZC3H6	5	gain
1970	CGTE_25	2	136633824	149260106	2q22.2-q23.1	PABPC1P2,LINC01412,THSD7B,MCM6,ZEB2-AS1,NXPH2,DARS-AS1,MIR7157,HNMT,ORC4,YY1P2,LOC101928386,CXCR4,ARHGAP15,DARS,ZEB2,LRP1B,MBD5,LOC101928273,GTDC1,SPOPL,KYNU,TEX41,ACVR2A	3	gain
1971	CGTE_25	2	161137832	168812249	2q24.2-q24.3	LOC101929512,LOC100506124,XIRP2-AS1,LOC100996579,LOC101929633,SNORA70F,IFIH1,GCA,SCN7A,RBMS1,TTC21B-AS1,KCNH7,SCN3A,LOC101929680,SCN1A,B3GALT1,SLC4A10,TTC21B,SLC38A11,FIGN,SCN9A,TBR1,MIR4785,GRB14,DPP4,LOC105616981,AHCTF1P1,XIRP2,LOC101929570,PSMD14,STK39,COBLL1,GALNT3,SCN2A,TANK,CSRNP3,GCG,LOC101929532,LOC102724058,FAP	3	gain
1972	CGTE_25	2	179485373	197649695	2q32.3-q32.1	TMEFF2,ITGA4,CCDC150,GULP1,LOC101927482,FAM171B,PCGEM1,LOC101927406,UBE2E3,ORMDL1,DNAJC10,GTF3C3,PDE1A,DIRC1,TFPI,TTN,FSIP2,HIBCH,ZSWIM2,SLC39A10,SLC40A1,OSGEPL1,OSGEPL1-AS1,LOC105747689,GLS,MIR1258,ITGAV,WDR75,SESTD1,MIR3129,C2orf88,STK17B,CCDC141,LOC101927156,INPP1,ZNF804A,ANKAR,LOC101927196,CWC22,COL3A1,PMS1,HECW2,COL5A2,STAT4,NUP35,FRZB,NEUROD1,SCHLAP1,LINC01090,SSFA2,ZC3H15,DUSP19,MIR561,CALCRL,NAB1,MIR4437,MIR548N,ASNSD1,LOC100130452,LINC01473,ZNF385B,MFSD6,MSTN,MIR548AE1,MIR1245A,MYO1B,MIR3606,LOC101927055,NEMP2,NCKAP1,PPP1R1C,STAT1,CERKL,MIR1245B,NABP1,SDPR,DNAH7,LOC101927431	3	gain
1973	CGTE_25	2	209189950	216293109	2q35-q34	FN1,PIKFYVE,LOC101928103,CPS1,MIR548F2,MIR4776-1,IKZF2,LOC100130451,ERBB4,LANCL1-AS1,LOC101928020,PTH2R,LOC102725079,MAP2,LANCL1,BARD1,ATIC,SPAG16,VWC2L,CPS1-IT1,UNC80,KANSL1L,RPE,ABCA12,MYL1,VWC2L-IT1,ACADL,MIR4776-2,MIR4438	3	gain
1974	CGTE_25	3	77571935	89521805	3p11.2-p11.1	LOC101927374,HTR1F,GBE1,LINC00971,MIR4795,EPHA3,ZNF654,ROBO2,MIR5688,LINC00506,ROBO1,CADM2-AS2,LOC728290,VGLL3,C3orf38,POU1F1,MIR3923,CADM2,CHMP2B,SNORA95,CGGBP1	3	gain
1975	CGTE_25	4	493077	1379815	4p16.3	GAK,DGKQ,LOC100130872,PCGF3,CTBP1,ATP5I,MYL5,FGFRL1,CTBP1-AS2,LOC100129917,CTBP1-AS,MAEA,IDUA,UVSSA,MFSD7,TMEM175,TMED11P,SLC26A1,PIGG,CPLX1,SPON2,RNF212,PDE6B	1	loss
1976	CGTE_25	4	62845326	67142606	4q13.2-q13.1	LOC401134,ADGRL3,EPHA5,ADGRL3-AS1,EPHA5-AS1,MIR1269A,TECRL	4	gain

1977	CGTE_25	4	68201868	70803639	4q13.3-q13.2	LOC550113,SULT1B1,UGT2B17,LOC101927237,TMPRSS11D,UGT2A1,UGT2B4,TMPRSS11GP,YTHDC1,FTLP10,CSN1S1,TMPRSS11BNL,SYT14P1,UGT2B7,UGT2B10,UGT2B15,TMPRSS11B,UGT2A3,UBA6,UGT2B11,UGT2B28,UGT2A2,UBA6-AS1,TMPRSS11E,TMPRSS11A,SULT1E1,GNRHR,STAP1,TMPRSS11F,CENPC	3	gain
1978	CGTE_25	4	70804746	70945497	4q13.3	CSN1S1,CSN2,HTN1,STATH,HTN3,CSN1S2AP	4	gain
1979	CGTE_25	5	80756811	110837817	5q21.1-q21.2	CAST,LOC100289673,NUDT12,MBLAC2,STARD4,FBXL17,TMEM167A,ARSK,ERAP2,RPS23,LOC100505878,MIR548F3,LOC101929380,CCNH,LOC102546226,LOC644285,LOC731157,SLCO6A1,ARRDC3-AS1,RFESD,ATP6AP1L,PPIP5K2,LNPEP,VCAN,PCSK1,TMEM232,SPATA9,LOC100133050,CTD-2151A2.1,LOC100289230,SLC25A46,MEF2C,LINC00491,SLCO4C1,EFNA5,POLR3G,ADGRV1,ATG10,FAM172A,SLF1,RHOBTB3,LOC101929710,MCTP1,MAN2A1,GLRX,RASA1,POU5F2,FAM174A,LINC01023,LINC00492,SSBP2,MIR3607,GPR150,RAB9BP1,CHD1,PJA2,FAM81B,XRCC4,GIN1,LYSMD3,MIR548AO,LOC55338,HAPLN1,SCARNA18,LOC102467212,MEF2C-AS1,RGMB-AS1,RGMB,MIR583,LINC01340,LINC01339,MIR3660,MIR548P,MIR2277,C5orf30,ERAP1,NR2F1-AS1,COX7C,RIOK2,NBPF22P,ARRDC3,LINC00461,EDIL3,MIR4280,MIR9-2,TSLP,LINC01554,LOC102467213,TTC37,CAMK4,TMEM161B,TMEM161B-AS1,KIAA0825,ELL2,ST8SIA4,LIX1,MIR3977,LINC01338,WDR36,NR2F1,LUCAT1,FER,PAM,CETN3	3	gain
1980	CGTE_25	5	126674797	129521485	5q23.3-q23.2	LINC01184,MEGF10,KIAA1024L,ADAMTS19,ISOC1,ADAMTS19-AS1,SLC27A6,CHSY3,MIR4460,FBN2,SLC12A2,MIR4633,CTXN3,PRRC1	3	gain
1981	CGTE_25	5	160721095	167638822	5q34	GABRG2,GABRB2,CCNG1,LINC01202,HMMR,LOC102546299,CTB-7E3.1,GABRA1,MAT2B,TENM2,NUDCD2,GABRA6,LOC101927835,HMMR-AS1	3	gain
1982	CGTE_25	6	25452230	29571433	6p22.1-p22.2	TRIM27,HIST1H4K,ZNF391,HIST1H2AA,ZSCAN9,ZSCAN31,HIST1H3I,SLC17A2,HIST1H2BE,OR2J3,BTN3A2,HIST1H2BJ,SLC17A1,HIST1H4F,HIST1H3H,OR2J2,HIST1H4E,HIST1H1B,HIST1H2AD,HIST1H3F,GPX5,HIST1H4C,LRRC16A,HIST1H2BL,BTN2A2,POM121L2,TOB2P1,ZBED9,LOC100129636,SLC17A4,OR5V1,HIST1H1C,ZKSCAN4,HFE,TRIM38,LINC00240,HIST1H4H,MAS1L,HIST1H2AJ,HIST1H4L,HIST1H2AE,HIST1H3D,UBD,HIST1H2APS1,ZSCAN12P1,PGBD1,OR2H1,HIST1H2BI,ZNF322,LINC01623,OR2W1,ZSCAN23,ZNF165,OR2B6,HIST1H3G,HIST1H2BD,HIST1H2BF,OR12D3,HIST1H2AM,HIST1H3C,LINC01556,GABBR1,HIST1H2BC,HIST1H4A,OR2B3,ZNF311,SNORD32B,ABT1,HIST1H2AL,ZSCAN16,HIST1H2BH,HIST1H2BO,HIST1H2BM,HIST1H4J,HIST1H1E,HCG11,HIST1H3A,MIR3143,HIST1H2BB,ZNF184,BTN3A1,GPX6,LOC100270746,HIST1H4D,HIST1H2AI,SCGN,LINC01012,HIST1H2AG,HMGN4,ZSCAN16-AS1,HIST1H4I,HIST1H3J,ZKSCAN8,HIST1H2BN,LOC285819,HIST1H1D,HIST1H2AH,ZKSCAN3,HCG14,HIST1H2AB,PRSS16,OR12D2,BTN2A1,NKAPL,ZSCAN26,HIST1H2BG,LOC100131289,BTN1A1,OR2B2,HIST1H3E,OR14J1,HIST1H2BK,ZNF192P1,OR2H2,LINC01015,OR11A1,HIST1H1A,HIST1H2AC,SLC17A3,ZNF204P,HIST1H2BA,GUSBP2,HIST1H3B,OR10C1,ZSCAN12,VN1R10P,HIST1H2AK,HIST1H1T,HIST1H4B,BTN2A3P,BTN3A3,HIST1H4G	3	gain
1983	CGTE_25	6	144837336	148050973	6q24.2-q24.3	ADGB,KATNBL1P6,EPM2A,UTRN,FBXO30,STXBP5,SAMD5,LOC101928661,STXBP5-AS1,SHPRH,RAB32,GRM1,LUADT1,LOC100507557	3	gain

1984	CGTE_25	7	7106427	20739767	7p21.1-p22.1	C1GALT1,LOC100131257,LOC101927630,FERD3L,THSD7A,PER4,TMEM196,TMEM106B,LOC100505921,AGR2,MEOX2,DGKB,ANKMY2,ISPD-AS1,MIR3683,NXPH1,MIR1302-6,MIR3146,AHR,PHF14,LOC100505938,SOSTDC1,SCIN,COL28A1,ITGB8,AGR3,TSPAN13,ABCB5,ETV1,BZW2,LOC101927354,HDAC9,LOC101927668,MACC1,SNX13,MIOS,GLCCI1,LOC101927769,LOC101927391,AGMO,NDUFA4,UMAD1,VWDE,MACC1-AS1,PRPS1L1,LRRC72,ISPD,LOC101927811,ARL4A,RPA3,ICA1,TWIST1,MEOX2-AS1,TWISTNB,KCCAT333	3	gain
1985	CGTE_25	7	77754993	86814437	7q21.12-q21.11	LOC101927356,MAGI2,KIAA1324L,MIR548M,SEMA3A,SEMA3E,LOC101927378,MAGI2-AS2,MAGI2-AS3,LOC101927269,LOC100128317,SEMA3D,RPL13AP17,GRM3,CACNA2D1,PCLO,DMTF1,CD36,GNAI1,HGF,SEMA3C,GNAT3	3	gain
1986	CGTE_25	8	21946698	22064499	8p21.3	NUDT18,FAM160B2,LGI3,SFTPC,REEP4,HR,BMP1	1	loss

1987	CGTE_25	8	43159834	120575339	8q21.3-q12.2	CHCHD7,MIR3610,NCALD,MIR7705,RGS22,PLAG1,FAM92A1,LACTB2-AS1,SAMD12-AS1,SLC30A8,C8orf59,RGS20,MIR2052,CALB1,C8orf22,NPBWR1,GDAP1,WWP1,MAL2,LINC01607,TNFRSF11B,CRH,PKIA,ZFHX4-AS1,NCOA2,TPD52,OPRK1,OTUD6B-AS1,RB1CC1,SNX31,CHD7,CNGB3,SNHG6,SLC25A32,TRIM55,NDUFAF6,LINC01592,FLJ46284,MCMDC2,MIR5681A,RALYL,PAG1,C8orf46,TSPYL5,E2F5,PREX2,MIR5681B,ODF1,LOC102724612,BAALC-AS2,FABP12,LOC101929528,ADHFE1,LINC00535,RDH10,LOC101929415,SLCO5A1,CDH17,RUNX1T1,C8orf37,TOX,SDR16C5,TP53INP1,SPIDR,CA3,ZFPM2-AS1,ATP6V0D2,LINC01289,LINC01419,HNF4G,EFCAB1,SPAG1,RRS1,IMPA1,SULF1,CLVS1,KIAA1429,PKHD1L1,CA8,LRRCC1,SBF1P1,LINC01609,LINC01030,BAALC,TMEM55A,ERICH5,KLF10,SOX17,PENK,UTP23,CTHRC1,EIF3H,SDR16C6P,CNBD1,STMN2,TCEB1,LOC104054148,MIR124-2HG,C8orf37-AS1,FABP9,LINC01299,XKR4,SDC2,C8orf87,FABP5,LOC101927040,UQCRB,ZC2HC1A,LINC01602,LOC101929217,MIR1273A,NACAP1,MRPL15,LINC00536,FABP4,CSPP1,ABRA,MIR2052HG,ENY2,MIR599,PTDSS1,SNORA72,LINC01603,IMPAD1,TRPA1,LOC102724874,DECR1,DPYS,LOC401463,RMDN1,PMP2,STAU2-AS1,CA1,TRHR,ZBTB10,LYN,TCEA1,DNAJC5B,SAMD12,MOS,TRAM1,MMP16,RDH10-AS1,SDCBP,MIR7641-2,NUDCD1,CA3-AS1,ZNF704,UBR5,ARFGEF1,UBXN2B,NIPAL2,SNAI2,SGK3,RPL30,COPS5,JPH1,AZIN1,LINC00588,UG0898H09,KCNV1,LOC102724623,ASPH,YTHDF3-AS1,TGS1,MIR4661,SLC26A7,FAM150A,ARMC1,RAD21,RAD21-AS1,FSBP,EXT1,LOC392232,LOC105375650,RIMS2,RPL7,HEY1,RIPK2,ANGPT1,ENPP2,STK3,HRSP12,CPQ,YWHAZ,CASC9,MTFR1,MIR378D2,MIR3150A,LOC286177,NBN,CA2,ESRP1,MIR4470,PXDNL,C8orf89,LRRC69,UBE2W,POLR2K,LACTB2,LINC00967,CYP7A1,ATP6V1C1,UBE2V2,LINC01608,LINC00293,MIR3151,LOC100130298,SNTG1,MIR3150B,OXR1,PRKDC,RAD54B,CCNE2,C8orf34,GEM,CHMP4C,AARD,LOC101926908,LOC101241902,ZFPM2,PRDM14,MIR4471,SBSPON,EMC2,MTERF3,PDP1,LINC01298,INTS8,MIR8084,LOC101929268,MRPS28,MED30,AZIN1-AS1,REXO1L2P,MTDH,FAM110B,DCSTAMP,GRHL2,LINC01301,C8orf44-SGK3,C8orf34-AS1,MATN2,RRS1-AS1,MIR5680,RP1,SNORD54,RBM12B-AS1,SNORD87,LINC01111,C8orf88,RPS20,LOC101926892,LY96,OSGIN2,MIR2053,UBR5-AS1,NKAIN3,TMEM68,CRISPLD1,PDE7A,NOV,TTPA,KCNB2,SLC7A13,TCF24,MIR3149,OSR2,DCAF4L2,TMEM67,FLJ42969,CPNE3,PKIA-AS1,EBAG9,TERF1,LOC100287846,LOC101927066,TMEM74,C8orf44,LINC01606,RSPO2,MIR875,LINC00251,GGH,FZD6,LAPTM4B,CEBPD,ZFHX4,DCAF13,COLEC10,GDF6,PTTG3P,TRIQK,CPA6,SLC10A5,PI15,MIR124-2,ZNF706,PSKH2,PLEKHF2,VCPIP1,RAB2A,SNX16,FBXO43,ANKRD46,DPY19L4,LRP12,BAALC-AS1,PEX2,CA13,MYBL1,TRPS1,POP1,VPS13B,OTUD6B,NECAB1,COX6C,ST18,NSMAF,PABPC1,MCM4,LOC101929709,IL7,PPP1R42,LOC101929488,LOC102724804,POTEA,CYP7B1,YTHDF3,RBM12B,TMEM64,XKR9,MIR5708,LINC00968,PCMTD1,MSC-AS1,LOC102724710,MSC,LOC100288748,KCNS2,LOC100500773,EIF3E,RRM2B,ZFAND1,EYA1,SYBU,STAU2,RNF19A,LYPLA1,CSMD3,BHLHE22,ATP6V1H,LINC00534,TMEM70	3	gain
1988	CGTE_25	9	8437031	21078002	9p22.1-p22.2	BNC2,MPDZ,MIR4474,TYRP1,DENND4C,SCARNA8,PSIP1,HACD4,FLJ41200,RRAGA,ZDHHC21,CNTLN,CCDC171,C9orf92,CER1,TTC39B,ADAMTSL1,SNORD137,LURAP1L,MIR4473,PTPRD,PTPRD-AS2,NFIB,SLC24A2,FREM1,FOCAD,SH3GL2,LINC00583,ACER2,MIR3152,MIR491,SAXO1,RPS6,LURAP1L-AS1,SNAPC3,IFNB1,HAUS6,PLIN2,LOC389705,MLLT3,PTPRD-AS1,FOCAD-AS1	3	gain
1989	CGTE_25	9	21140925	21409569	9p21.3	IFNA6,IFNA8,IFNA21,IFNW1,IFNA13,IFNA14,KLHL9,IFNA2,IFNA10,IFNA7,IFNA5,IFNA22P,IFNA4,IFNA16,IFNA17	4	gain

1990	CGTE_25	9	71840940	84267304	9q21.13-q21.32	TRPM6,TLE1,MAMDC2,LINC01504,ALDH1A1,PCSK5,TRPM3,GNA14,GCNT1,TJP2,ZFAND5,CEP78,LINC01507,TLE4,MIR6130,C9orf135-AS1,SMC5-AS1,LOC101927450,RORB,C9orf40,SMC5,PSAT1,OSTF1,PCA3,FOXB2,PRUNE2,CARNMT1,RORB-AS1,MIR548H3,FAM189A2,KLF9,ABHD17B,GNA14-AS1,MAMDC2-AS1,GNAQ,C9orf85,TMC1,RFK,VPS13A,RPSAP9,VPS13A-AS1,TMEM2,GDA,LINC01474,C9orf135,ANXA1,NMRK1,C9orf41-AS1,APBA1,BANCR,LOC101927358,MIR204,C9orf57,PTAR1	3	gain
1991	CGTE_25	9	103348097	107517045	9q31.1	CYLC2,OR13C9,BAAT,TMEM246,ALDOB,SMC2-AS1,OR13F1,OR13C5,OR13C8,LOC101928523,PLPPR1,LINC00587,TMEM246-AS1,NIPSNAP3A,MURC,OR13C2,LINC01492,MRPL50,ZNF189,OR13C4,PPP3R2,GRIN3A,OR13C3,OR13D1,RNF20,SMC2	3	gain
1992	CGTE_25	9	117109874	122075634	9q32-q33.1	C9orf91,ATP6V1G1,ASTN2-AS1,DFNB31,LOC101928775,SNORA70C,TNC,TLR4,LOC100505478,LOC101928797,TNFSF15,PAPPA,LOC101928748,TRIM32,DEC1,AKNA,BRINP1,PAPPA-AS1,LINC00474,ASTN2,TNFSF8	3	gain
1993	CGTE_25	9	130474826	130482683	9q34.11	CFAP157,PTRH1,TTC16	0	loss
1994	CGTE_25	9	130485462	130490014	9q34.11	TTC16	16	gain
1995	CGTE_25	9	133759579	133769257	9q34.12	ABL1,QRFP	7	gain
1996	CGTE_25	9	136913138	137734125	9q34.2-q34.3	WDR5,RXRA,BRD3,RNU6ATAC,MIR4669,LOC101448202,COL5A1	1	loss
1997	CGTE_25	9	137742454	137775248	9q34.3	FCN2,LOC101448202,MIR3689F,MIR3689A,MIR3689E	5	gain
1998	CGTE_25	9	139092400	139101168	9q34.3	LHX3,QSOX2	76	gain
1999	CGTE_25	9	139257347	139261361	9q34.3	CARD9,DNLZ	8	gain
2000	CGTE_25	9	139658757	139686522	9q34.3	TMEM141,LCN15	8	gain
2001	CGTE_25	9	140344368	140346954	9q34.3	MIR7114,NSMF	23	gain
2002	CGTE_25	9	140349971	140379208	9q34.3	PNPLA7,NSMF	0	loss
2003	CGTE_25	10	52002917	58121135	10q21.1-q11.23	SGMS1-AS1,SGMS1,ZWINT,A1CF,ASAH2,MBL2,MTRNR2L5,PRKG1,LINC01468,PCDH15,LOC102724719,PRKG1-AS1,CSTF2T,MIR605,ASAH2B,DKK1	3	gain
2004	CGTE_25	10	93242715	93257967	10q23.32	HECTD2,HECTD2-AS1	7	gain
2005	CGTE_25	10	105752277	105759890	10q24.33	SLK	8	gain
2006	CGTE_25	10	133948481	135216461	10q26.3	MIR202,MIR3944,FUOM,KNDC1,ECHS1,CALY,ZNF511,LINC01167,ADGRA1,INPP5A,UTF1,JAKMIP3,LRRC27,VENTX,MIR202HG,MTG1,NKX6-2,LINC01166,TUBGCP2,ADGRA1-AS1,PWWP2B,PRAP1,CFAP46,PAOX,C10orf91,LINC01168,ADAM8,STK32C,DPYSL4	1	loss

2007	CGTE_25	11	193051	3380822	11p15.5-p15.4	CDKN1C,SCGB1C2,RIC8A,CDHR5,TALDO1,LSP1,SLC22A18AS,NAP1L4,TSPAN4,RASSF7,FAM99A,DUSP8,MRPL23,BRSK2,MIR210HG,KRTAP5-AS1,MUC5B,IRF7,MIR4686,LOC143666,MIR210,PKP3,KCNQ1-AS1,MRGPRG-AS1,TNNI2,TSSC4,TH,LRRC56,SCGB1C1,ANO9,IFITM3,LOC171391,SLC22A18,CD151,ODF3,MRGPRE,MUC2,IGF2,MUC6,C11orf21,TOLLIP,MIR6744,KCNQ1OT1,IGF2-AS,MIR675,IFITM2,INS,PNPLA2,KCNQ1,MRPL23-AS1,MRGPRG,KRTAP5-2,IFITM10,IFITM1,MIR7847,PHRF1,IFITM5,DRD4,SLC25A22,LINC01219,LINC01150,TNNT3,TSPAN32,POLR2L,SNORA54,SCT,OSBPL5,SNORD131,CEND1,KRTAP5-6,HRAS,RPLP2,MIR6743,KRTAP5-3,SYT8,SNORA52,CD81,KCNQ1DN,ASCL2,MUC5AC,PANO1,RNH1,CD81-AS1,MIR4298,KRTAP5-1,KRTAP5-5,TOLLIP-AS1,CTSD,PHLDA2,PDDC1,KRTAP5-4,HOTS,H19,SIGIRR,CARS,MOB2,PSMD13,SIRT3,FAM99B,AP2A2,BET1L,MIR483,ZNF195,LMNTD2,CHID1,TMEM80,INS-IGF2,DEAF1,PIDD1,TRPM5,ATHL1,CRACR2B,B4GALNT4,PTDSS2,NLRP6,EPS8L2	1	loss
2008	CGTE_25	11	3380963	6231840	11p15.4	RHOG,OR52M1,OR52E8,OR56A4,OR51E1,OR51V1,OR51Q1,OR51G1,OR51D1,OR56B4,OR56A5,OLFM5P,HBBP1,OR51T1,UBQLNL,OR52B2,STIM1,OR52B4,OR52K1,OR52A5,TRIM22,TRPC2,OR52W1,OR51F1,OR52H1,OR52D1,ART5,OR51A2,OR52I2,OR51L1,TRIM34,TRIM68,OR52N5,TRIM6,OR56B1,OR7E12P,PGAP2,ART1,LOC101927708,OR52B6,TRIM21,CHRNA10,OR51G2,OR51I2,OR51B6,MIR4687,OR51I1,C11orf40,UBQLN3,HBE1,OR52R1,OR52E4,OR52E6,OR56A3,TRIM5,OR51M1,C11orf42,HBD,OR52N4,BGLT3,RRM1,OR51S1,NUP98,OR51B2,OR56A1,TRIM6-TRIM34,OR51E2,HBB,OR52A1,TSSC2,OR51A4,OR52J3,ZNF195,OR51B4,OR51A7,OR52N1,LOC100506082,OR52I1,OR52E2,OR51F2,HBG2,OR52L1,OR51B5,OR52N2,OR52K2,MMP26,HBG1	3	gain
2009	CGTE_25	11	102710809	106849306	11q22.2-q22.3	CARD18,CASP4,KBTBD3,CASP12,MMP13,CASP5,MMP12,GUCY1A2,DDI1,PDGFD,DCUN1D5,MIR4693,LOC101928535,MSANTD4,CASP1P2,MIR7641-1,CASP1,CARD17,AASDHPPT,DYNC2H1,LOC643733,LOC102723895,CARD16,GRIA4,MMP3	3	gain
2010	CGTE_25	12	32260206	47630279	12q11-p11.1	ALG10B,LRRK2,SYT10,GXYLT1,MUC19,PKP2,CPNE8,YAF2,CNTN1,C12orf40,YARS2,ABCD2,MIR4698,AMIGO2,RACGAP1P,BICD1,TMEM117,RNY5,IRAK4,KIF21A,NELL2,ADAMTS20,SLC2A13,ARID2,PCED1B,SLC38A2,PCED1B-AS1,PUS7L,TWF1,LOC101927058,SLC38A1,PLEKHA8P1,FGD4,DNM1L,PRICKLE1,ANO6,ZCRB1,LOC100288798,PPHLN1,MIR7851,ALG10,LINC00938,DBX2,PDZRN4,SLC38A4,SCAF11	3	gain
2011	CGTE_25	12	59271059	62974202	12q14.1	USP15,SLC16A7,LRIG3,MIR6125,FAM19A2,MON2	3	gain
2012	CGTE_25	12	70507857	93245057	12q15-q21.2	GLIPR1,SYT1,MIR5692B,DCN,PAWR,MYF5,KRR1,TSPAN19,TPH2,LGR5,CLLU1OS,CCDC59,PHLDA1,GALNT4,MGAT4C,LIN7A,PTPRQ,TRHDE,LOC100507377,THAP2,KITLG,LUM,KCNC2,CEP290,OSBPL8,KCNMB4,MIR1252,METTL25,LOC102724663,LINC01481,LOC101928137,TBC1D15,CCER1,EPYC,ATP2B1,PPP1R12A,LINC00615,MIR548AL,ALX1,MIR617,BTG1,GLIPR1L2,MRS2P2,PLEKHG7,NAP1L1,EEA1,LINC01619,MKRN9P,LOC728084,TMTC3,ZDHHC17,TRHDE-AS1,SLC6A15,KERA,PTPRR,BBS10,CAPS2,RASSF9,LRRIQ1,POC1B-GALNT4,PTPRB,ZFC3H1,C12orf74,NAV3,MIR4699,TSPAN8,MIR618,ACSS3,C12orf50,POC1B,CSRP2,LINC01490,LINC00936,DUSP6,OTOGL,TMTC2,RAB21,C12orf29,CLLU1,TMEM19,NTS,E2F7,CNOT2,PPFIA2,GLIPR1L1,MYF6,LOC101928449,ATXN7L3B	3	gain

2013	CGTE_25	12	93245948	93247773	12q22	EEA1	5	gain
2014	CGTE_25	13	33233284	41134195	13q13.1-q14.11	PROSER1,SMAD9,LINC00548,MAB21L1,LINC00332,CCDC169-SOHLH2,LHFP,KL,TRPC4,LINC00445,LINC00457,MIR548F5,EXOSC8,FREM2,SUPT20H,NBEA,FOXO1,POSTN,LINC00547,LINC00437,SPG20,UFM1,COG6,CCNA1,RFXAP,STARD13,ALG5,MIR4305,SPG20-AS1,SERTM1,LINC00598,LINC00423,DCLK1,CSNK1A1L,STOML3,LINC01048,NHLRC3,LINC00366,LINC00571,RFC3,CCDC169,PDS5B,SOHLH2,STARD13-AS	3	gain
2015	CGTE_25	13	58240574	95275580	13q22.2-q21.33	TBC1D4,COMMD6,LOC101926897,FBXL3,SLITRK6,LMO7,LINC00379,GPC5-AS1,GPC5,IRG1,DIAPH3-AS2,LINC00440,LINC00378,LINC01052,MYCBP2-AS1,MIR17HG,LINC00381,DIAPH3,LINC00375,POU4F1,MIR20A,LINC00374,ATXN8OS,RNF219,LINC00380,PIBF1,PCDH9-AS2,PCDH17,NDFIP2,LOC100129307,RBM26-AS1,UCHL3,KLF5,MIR4500,MIR18A,OR7E156P,KCTD12,LINC00410,CTAGE11P,SLAIN1,MIR92A1,LINC00333,LINC01069,LINC00559,LINC00392,KLHL1,LINC00448,LINC00331,RNF219-AS1,LINC00347,EDNRB,BTF3P11,LINC00434,TGDS,DIS3,LMO7-AS1,LOC102723968,LINC01047,SPRY2,GPR180,LINC00430,DACH1,MIR622,LOC105370306,NDFIP2-AS1,LINC00377,LINC00376,LINC00550,MYCBP2,PCDH20,LINC00382,LINC00395,MIR548X2,LINC00363,LINC00358,SCEL,MIR19B1,LINC00446,LINC00383,BORA,MIR19A,SCEL-AS1,LINC01080,LINC01038,LINC00353,SLITRK1,PCDH9,LINC00351,LINC01049,RBM26,PCDH9-AS3,MIR3169,LINC01040,TDRD3,PCDH9-AS4,LMO7DN,GPC6,LMO7DN-IT1,MIR17,CLN5,MIR4500HG,SNORA107,DCT,GPC6-AS1,MZT1,DIAPH3-AS1,EDNRB-AS1,LINC00433,SLITRK5,LINC00564,LINC00348,LINC00364,GPC6-AS2,KLF12,LINC00397,LINC01068,MIR3665,MIR4704,LINC01078,LINC01075,GPC5-AS2	3	gain
2016	CGTE_25	14	21990264	22789098	14q11.2	OR10G3,OR4E1,SALL2,OR4E2,OR10G2	3	gain
2017	CGTE_25	15	49867171	49869886	15q21.2	FAM227B	8	gain
2018	CGTE_25	15	66856213	66857701	15q22.31	LCTL	7	gain
2019	CGTE_25	16	309429	1570362	16p13.3	TPSAB1,GNPTG,SSTR5-AS1,CACNA1H,RHBDL1,FBXL16,MSLN,TMEM8A,C1QTNF8,RPUSD1,SOX8,WFIKKN1,NARFL,LMF1-AS1,FAM234A,TELO2,GNG13,CCDC78,TSR3,FAM195A,BAIAP3,TPSD1,LINC00235,RHOT2,RAB40C,METRN,LOC100134368,MRPL28,CAPN15,WDR90,TPSB2,MIR5587,PTX4,PRR35,HAGHL,IFT140,JMJD8,RAB11FIP3,CCDC154,PRR25,SSTR5,TPSG1,UBE2I,CLCN7,NME4,WDR24,PDIA2,PIGQ,STUB1,ARHGDIG,DECR2,MIR662,AXIN1,C16orf13,NHLRC4,FAM173A,RGS11,C16orf91,LMF1,UNKL,CHTF18,MIR3176	1	loss
2020	CGTE_25	17	8168076	8171191	17p13.1	PFAS	9	gain
2021	CGTE_25	17	38856398	39622249	17q21.2	TMEM99,KRTAP9-1,KRT40,KRTAP1-1,KRT20,KRTAP4-7,KRTAP1-3,KRT38,KRT10,KRTAP2-1,KRTAP4-5,KRT33A,KRT26,LOC100505782,KRTAP4-1,KRTAP4-8,KRTAP3-2,KRT34,KRTAP1-4,KRT12,KRTAP9-4,KRTAP9-3,KRTAP3-3,KRT23,KRTAP4-3,KRT27,KRTAP4-12,KRTAP9-6,KRTAP4-9,KRT32,KRTAP29-1,KRT33B,KRTAP16-1,KRTAP9-8,KRT39,KRTAP4-6,KRT37,KRT24,KRTAP9-7,KRTAP1-5,KRTAP2-2,KRT31,KRTAP4-11,KRTAP4-2,KRTAP17-1,KRTAP9-9,KRT28,KRT25,KRTAP9-2,KRTAP2-3,KRTAP2-4,KRTAP3-1,KRTAP4-4	3	gain
2022	CGTE_25	17	48613895	48668030	17q21.33	SPATA20,EPN3,CACNA1G,CACNA1G-AS1	0	loss

2023	CGTE_25	17	66518859	67310627	17q24.2-q24.3	PRO1804,PRKAR1A,MIR4524B,ABCA6,FAM20A,ABCA8,ABCA9,ABCA5,ABCA9-AS1,LINC01482,MIR4524A,ABCA10	3	gain
2024	CGTE_25	17	79941384	79954060	17q25.3	ASPSCR1	7	gain
2025	CGTE_25	18	712200	743500	18p11.32	YES1,ENOSF1	6	gain
2026	CGTE_25	18	9239420	9255438	18p11.22	ANKRD12	47	gain
2027	CGTE_25	18	22671762	29099970	18q12.1-q11.2	AQP4,DSG3,DSCAS,DSG1-AS1,PSMA8,DSC2,ZNF521,DSG4,MIR302F,AQP4-AS1,MIR8057,DSG2,CHST9,CDH2,DSC3,KCTD1,DSC1,TAF4B,DSG1,PCAT18,LINC01543,SS18	3	gain
2028	CGTE_25	18	30991819	31803214	18q12.1	NOL4,ASXL3,CCDC178	3	gain
2029	CGTE_25	18	45374848	45555738	18q21.1	SMAD2,ZBTB7C	4	gain
2030	CGTE_25	18	48577593	55020278	18q21.31-q21.2	LINC01539,SMAD4,TCF4,TCF4-AS1,TXNL1,DYNAP,LINC01416,LOC100287225,C18orf54,DCC,ST8SIA3,MIR4528,MIR4529,LOC101928167,SNORA37,BOD1L2,LOC102724651,POLI,CCDC68,STARD6,WDR7,MBD2,LOC101927229,LINC-ROR,RAB27B,MEX3C	3	gain
2031	CGTE_25	18	63430205	66355035	18q22.1	TMX3,LOC643542,MIR5011,DSEL,CDH19,CDH7	3	gain
2032	CGTE_25	18	66564393	70520672	18q22.2-q22.1	LOC101060542,GTSCR1,RTTN,NETO1,CBLN2,CCDC102B,CD226,SOCS6,DOK6,LINC01541,LOC101927481,LOC102724913	3	gain
2033	CGTE_25	19	281411	2271826	19p13.3	GRIN3B,AZU1,POLRMT,CFD,GPX4,PLPPR3,EFNA2,TPGS1,SBNO2,MIER2,MUM1,PRSS57,KLF16,CIRBP,BSG,PTBP1,CSNK1G2,REXO1,ARID3A,RPS15,R3HDM4,PALM,PCSK4,MIR1909,RNU6-2,C19orf25,HMHA1,MISP,PLEKHJ1,SF3A2,CDC34,ABCA7,LOC100288123,WDR18,PLK5,STK11,ABHD17A,GAMT,BTBD2,MIR3187,NDUFS7,MIR1227,DOT1L,SCAMP4,MBD3,C2CD4C,FGF22,KISS1R,ODF3L2,TMEM259,HCN2,APC2,IZUMO4,POLR2E,SHC2,MED16,RNF126,AMH,GZMM,MKNK2,MIR4745,AP3D1,PRTN3,MIR6789,ONECUT3,THEG,OAZ1,DAZAP1,FSTL3,CBARP,ADAMTSL5,UQCR11,REEP6,ATP5D,C19orf24,MIDN,ADAT3,CIRBP-AS1,PLPP2,MEX3D,JSRP1,ATP8B3,MADCAM1,MOB3A,ELANE,TCF3,MIR4321,CSNK1G2-AS1,CNN2	1	loss
2034	CGTE_25	19	2324116	2417042	19p13.3	TMPRSS9,LSM7,SPPL2B	3	gain
2035	CGTE_25	19	3964652	3978240	19p13.3	EEF2,DAPK3,MIR1268A	9	gain
2036	CGTE_25	19	5917655	5921411	19p13.3	RANBP3	16	gain
2037	CGTE_25	21	10793919	26946466	21q21.2-q21.3	LINC00317,ANKRD30BP2,USP25,ABCC13,RBM11,MIR99A,LOC339622,MIR3118-1,POTED,CHODL,TPTE,D21S2088E,LOC101927869,LOC388813,LOC102724188,LINC00320,MIR8069-2,MIR548XHG,CYP4F29P,ANKRD20A11P,LINC00158,BAGE3,MIR3156-3,LINC01549,LIPI,HSPA13,BTG3,MIR155HG,CXADR,LINC00308,MIRLET7C,NRIP1,C21orf91-OT1,BAGE,CHODL-AS1,BAGE4,BAGE2,MIR8069-1,MIR99AHG,C21orf91,MIR155,BAGE5,MIR125B2,TMPRSS15,LOC101927843,LINC01425,SAMSN1,SAMSN1-AS1,NCAM2	3	gain
2038	CGTE_25	21	26957999	26966214	21q21.3	MRPL39	6	gain
2039	CGTE_25	21	31538390	32253929	21q22.11	KRTAP19-7,KRTAP26-1,KRTAP19-4,KRTAP8-1,KRTAP19-6,KRTAP6-2,CLDN17,KRTAP6-3,KRTAP13-4,KRTAP19-3,KRTAP22-1,KRTAP22-2,KRTAP19-5,KRTAP13-3,KRTAP19-1,MIR4327,KRTAP20-2,KRTAP21-2,KRTAP7-1,KRTAP13-1,CLDN8,KRTAP21-1,KRTAP24-1,KRTAP27-1,KRTAP13-2,KRTAP11-1,KRTAP20-1,KRTAP20-4,LINC00307,KRTAP23-1,KRTAP15-1,KRTAP19-2,KRTAP6-1,KRTAP20-3,KRTAP21-3,KRTAP25-1	4	gain
2040	CGTE_25	22	19865838	20236757	22q11.21	MIR6816,CCDC188,MIR4761,COMT,LOC284865,ARVCF,ZDHHC8,MIR3618,RANBP1,TANGO2,TXNRD2,LINC00896,MIR1306,RTN4R,DGCR8,MIR1286,TRMT2A,MIR185	1	loss

2041	CGTE_25	X	30846337	37955577	Xp21.2-Xp11.4	DYNLT3,LANCL3,RP11-87M18.2,CYBB,MAGEB16,XK,CFAP47,PRRG1,MIR3915,DMD,TMEM47,FTHL17,HYPM,FAM47C,TAB3,SYTL5,FTH1P18,FAM47B,FAM47A	3	gain
2042	CGTE_25	X	78216274	96194162	Xq21.32-Xq21.2	ITM2A,TBX22,CYLC1,CHMP1B2P,BRWD3,PABPC5,POF1B,HDX,NAP1L3,CHM,TGIF2LX,KLHL4,DACH2,ZNF711,POU3F4,HMGN5,PCDH11X,FAM133A,APOOL,SH3BGRL,PABPC5-AS1,BRDTP1,UBE2DNL,GPR174,MIR4454,FAM46D,LOC101928128,RPS6KA6,DIAPH2,CPXCR1,MIR1321,P2RY10,SATL1,MIR361,RPA4	3	gain
2043	CGTE_25	X	107823776	107976134	Xq22.3	COL4A5,IRS4	3	gain
2044	CGTE_26	1	17248472	17265466	1p36.13	CROCC	3	gain
2045	CGTE_26	1	110754094	110754866	1p13.3	KCNC4	0	loss
2046	CGTE_26	1	115400018	115489998	1p13.2	SYCP1	1	loss
2047	CGTE_26	1	150676532	150689921	1q21.3	HORMAD1	1	loss
2048	CGTE_26	1	196748431	196796227	1q31.3	CFHR3,CFHR1	3	gain
2049	CGTE_26	2	61336321	61344664	2p15	KIAA1841	3	gain
2050	CGTE_26	2	70082038	70092046	2p13.3	GMCL1	1	loss
2051	CGTE_26	2	70528779	70679047	2p13.3	TGFA,FAM136A	1	loss
2052	CGTE_26	2	98280234	98315694	2q11.2	LINC01125,ACTR1B	3	gain
2053	CGTE_26	2	168921880	168997296	2q24.3	STK39	3	gain
2054	CGTE_26	2	179260715	179308166	2q31.2	OSBPL6,PRKRA,LOC101927027,MIR548N	4	gain
2055	CGTE_26	2	179309113	179312382	2q31.2	PRKRA,MIR548N	6	gain
2056	CGTE_26	3	138725034	138762807	3q23	PRR23A,PRR23C,PRR23B	1	loss
2057	CGTE_26	4	337656	366955	4p16.3	ZNF141,MIR571	3	gain
2058	CGTE_26	4	2159527	2172928	4p16.3	POLN	3	gain
2059	CGTE_26	4	13485654	13544271	4p15.33	RAB28,LINC01097,NKX3-2	1	loss
2060	CGTE_26	4	70936114	71062444	4q13.3	ODAM,CSN1S2AP,PRR27,CSN1S2BP	1	loss
2061	CGTE_26	4	184572225	184580072	4q35.1	RWDD4	1	loss
2062	CGTE_26	5	140553759	140558897	5q31.3	PCDHB7,PCDHB8	6	gain
2063	CGTE_26	5	170813667	170818516	5q35.1	MIR3912,NPM1	4	gain
2064	CGTE_26	5	180376274	180429863	5q35.3	BTNL3,BTNL8	0	loss
2065	CGTE_26	6	29855554	29910814	6p22.1	HLA-H,HCG4B,HLA-A	0	loss
2066	CGTE_26	6	31371272	31431879	6p21.33	HCP5,MICA	4	gain
2067	CGTE_26	6	32489819	32498113	6p21.32	HLA-DRB5	5	gain
2068	CGTE_26	6	32546706	32548765	6p21.32	HLA-DRB1	1	loss
2069	CGTE_26	6	32609662	32629904	6p21.32	HLA-DQA1,HLA-DQB1	1	loss
2070	CGTE_26	9	27290935	27294402	9p21.2	EQTN	3	gain
2071	CGTE_26	10	35896546	35929050	10p11.21	FZD8,GJD4	1	loss
2072	CGTE_26	10	104162894	104180918	10q24.32	FBXL15,PSD	3	gain
2073	CGTE_26	11	62287062	62302825	11q12.3	AHNAK	3	gain
2074	CGTE_26	11	85966153	85968554	11q14.2	EED	1	loss
2075	CGTE_26	11	95568377	95598850	11q21	MTMR2	3	gain
2076	CGTE_26	12	27648450	27655110	12p11.23	SMCO2	4	gain
2077	CGTE_26	13	21729143	21751303	13q12.11	MRPL57,SKA3	1	loss
2078	CGTE_26	14	21359911	21388280	14q11.2	ECRP,RNASE3	4	gain
2079	CGTE_26	14	60908697	60945166	14q23.1	C14orf39	1	loss
2080	CGTE_26	14	105415266	105417956	14q32.33	AHNAK2	1	loss

2081	CGTE_26	15	20170004	22833683	15q11.2-q11.1	MIR5701-2,MIR4509-1,CHEK2P2,REREP3,POTEB,MIR3118-2,MIR3118-4,GOLGA8DP,OR4N3P,MIR5701-1,LOC101927079,GOLGA6L22,LOC646214,GOLGA6L1,LINC01193,TUBGCP5,MIR3118-3,HERC2P3,POTEB2,LOC727924,OR4M2,OR4N4,MIR1268A,POTEB3,MIR4509-2,GOLGA6L6,NF1P2,NBEAP1,MIR5701-3,CXADRP2,GOLGA8CP,MIR4509-3	1	loss
2082	CGTE_26	15	41864558	41870539	15q15.1	TYRO3	1	loss
2083	CGTE_26	19	38375470	38386939	19q13.13	WDR87	1	loss
2084	CGTE_26	19	43343466	43576068	19q13.2-q13.31	PSG7,PSG1,PSG10P,PSG2,PSG11,PSG6	3	gain
2085	CGTE_26	19	43674229	43708437	19q13.31	PSG4,PSG5	5	gain
2086	CGTE_26	19	43708920	43716696	19q13.31	PSG4,LOC284344	3	gain
2087	CGTE_26	19	54802017	54804664	19q13.42	LILRA3	1	loss
2088	CGTE_26	19	55246674	55297955	19q13.42	KIR3DL3,KIR2DL1,LOC101928804,KIR2DL3	0	loss
2089	CGTE_26	19	55327978	55351058	19q13.42	KIR3DL1,KIR2DS4	0	loss
2090	CGTE_26	20	1569040	1584001	20p13	SIRPB1	4	gain
2091	CGTE_26	21	36259189	36265344	21q22.12	RUNX1	1	loss
2092	CGTE_26	22	24373191	24384346	22q11.23	GSTT1,GSTT1-AS1,LOC391322	1	loss
2093	CGTE_26	22	32546887	32548081	22q12.3	C22orf42	3	gain
2094	CGTE_28	1	861266	1245321	1p36.33	TNFRSF4,C1orf159,PUSL1,HES4,MIR6726,SAMD11,ACAP3,LINC01342,AGRN,TTLL10,MIR429,PERM1,UBE2J2,MIR200A,PLEKHN1,RNF223,KLHL17,FAM132A,SCNN1D,B3GALT6,TNFRSF18,SDF4,MIR200B,ISG15,NOC2L	0	loss
2095	CGTE_28	1	3382443	3391363	1p36.32	ARHGEF16	5	gain
2096	CGTE_28	1	3392506	3422105	1p36.32	MEGF6,ARHGEF16	0	loss
2097	CGTE_28	1	10754327	11190020	1p36.22	TARDBP,C1orf127,MTOR,EXOSC10,CASZ1,MASP2,SRM	3	gain
2098	CGTE_28	1	27939472	28223713	1p36.11-p35.3	FGR,RPA2,PPP1R8,IFI6,SCARNA1,THEMIS2,FAM76A,STX12	4	gain
2099	CGTE_28	1	43906032	44126151	1p34.2-p34.1	MIR6735,PTPRF,HYI,KDM4A,SZT2	3	gain
2100	CGTE_28	1	228247849	228329554	1q42.13	C1orf35,MIR3620,GUK1,ARF1,WNT3A,MRPL55	5	gain
2101	CGTE_28	1	228333228	228465399	1q42.13	GUK1,GJC2,IBA57,IBA57-AS1,C1orf145,OBSCN	0	loss
2102	CGTE_28	2	96789510	97587408	2q11.1-q11.2	SEMA4C,LOC101927053,NEURL3,CIAO1,CNNM4,LMAN2L,ITPRIPL1,ASTL,CNNM3,KANSL3,ANKRD39,ANKRD23,STARD7,SNRNP200,STARD7-AS1,MIR3127,NCAPH,ARID5A,TMEM127,FAM178B,FER1L5,DUSP2	4	gain
2103	CGTE_28	2	191785785	192548606	2q32.3-q32.2	GLS,MYO1B,STAT4,STAT1,NABP1	3	gain
2104	CGTE_28	3	47029424	47049379	3p21.31	NBEAL2	3	gain
2105	CGTE_28	3	184280095	184298270	3q27.1	EPHB3	6	gain
2106	CGTE_28	4	884322	949821	4p16.3	GAK,TMEM175	3	gain
2107	CGTE_28	4	949946	1019033	4p16.3	DGKQ,SLC26A1,TMEM175,IDUA,FGFRL1	0	loss
2108	CGTE_28	4	1019058	1093838	4p16.3	FGFRL1,RNF212	3	gain
2109	CGTE_28	4	1102015	1211207	4p16.3	LOC100130872,CTBP1-AS,CTBP1,RNF212,TMED11P,SPON2	0	loss
2110	CGTE_28	4	1739332	1804832	4p16.3	TACC3,FGFR3	0	loss
2111	CGTE_28	4	1805388	1806274	4p16.3	FGFR3	12	gain
2112	CGTE_28	4	1806365	1809470	4p16.3	FGFR3	0	loss
2113	CGTE_28	4	8239247	8417468	4p16.1	SH3TC1,HTRA3,ACOX3	3	gain
2114	CGTE_28	4	184567442	184577210	4q35.1	RWDD4	7	gain
2115	CGTE_28	5	140384	1063093	5p15.33	PP7080,EXOC3-AS1,SDHA,ZDHHC11,HRAT5,PDCD6,PLEKHG4B,TPPP,SLC9A3,CCDC127,MIR4635,AHRR,LOC100506688,BRD9,LRRC14B,LOC100288152,SLC12A7,NKD2,CEP72,EXOC3,TRIP13,LOC100996325,MIR4456	4	gain
2116	CGTE_28	5	1063891	1093812	5p15.33	SLC12A7	0	loss
2117	CGTE_28	5	1094263	1488642	5p15.33	SLC12A7,TERT,CTD-3080P12.3,SLC6A18,SLC6A3,SLC6A19,LPCAT1,LINC01511,MIR4457,CLPTM1L	4	gain
2118	CGTE_28	5	172386735	172421971	5q35.1	ATP6V0E1,RPL26L1	6	gain

2119	CGTE_28	6	36938197	43646440	6p21.2-p21.1	ZNF318,CUL9,MRPS10,TREM1,TFEB,TREML2,BTBD9,MRPL2,BYSL,POLH,UNC5CL,TRERF1,ZFAND3,TBC1D22B,MRPS18A,TREM2,TREML3P,TOMM6,DNAH8,GUCA1A,GLO1,SRF,YIPF3,PTCRA,LOC100131047,NFYA,TMEM217,PRICKLE4,PTK7,MED20,FGD2,ADCY10P1,MIR4462,KLC4,DLK2,TREML5P,MEA1,OARD1,LOC101929555,MTCH1,ABCC10,MIR4641,GLTSCR1L,LRRC73,MDGA1,LRFN2,CCDC167,FOXP4,MIR6780B,CUL7,TDRG1,TTBK1,RPL7L1,CRIP3,LOC100505635,TBCC,CMTR1,MOCS1,KCNK5,MDFI,TREML4,C6orf132,FRS3,LOC100505530,PRPH2,UBR2,CCND3,KIF6,GLP1R,FOXP4-AS1,DNPH1,TREML1,POLR1C,PIM1,LINC00951,KCNK16,TAF8,GTPBP2,XPO5,RRP36,C6orf226,NCR2,PPP2R5D,MAD2L1BP,PGC,GUCA1B,LINC01276,RNF8,PEX6,APOBEC2,CNPY3,GNMT,KLHDC3,SLC22A7,RSPH9,ATP6V0CP3,DAAM2,SAYSD1,TJAP1,TSPO2,KCNK17,USP49	3	gain
2120	CGTE_28	6	43648775	43969869	6p21.1	LOC101929705,VEGFA,C6orf223,MRPS18A,LINC01512	5	gain
2121	CGTE_28	7	195557	1476661	7p22.3	FAM20C,LOC101927000,HRAT92,C7orf50,DNAAF5,MICALL2,LOC101927021,SUN1,PRKAR1B,GPR146,UNCX,ZFAND2A,ADAP1,GET4,GPER1,LOC442497,PDGFA,WI2-2373I1.2,CYP2W1,COX19,MIR339,LOC101926963	3	gain
2122	CGTE_28	7	1476930	1533604	7p22.3	INTS1,MICALL2	0	loss
2123	CGTE_28	7	1533622	1542681	7p22.3	INTS1	6	gain
2124	CGTE_28	7	1542685	1785900	7p22.3	TMEM184A,ELFN1-AS1,PSMG3-AS1,MAFK,TFAMP1,PSMG3,INTS1,ELFN1	0	loss
2125	CGTE_28	7	1883690	2420119	7p22.3	SNX8,MIR6836,NUDT1,MAD1L1,MIR4655,FTSJ2,EIF3B	3	gain
2126	CGTE_28	7	2433420	2582545	7p22.3	CHST12,BRAT1,LOC101927181,LFNG,MIR4648,GRIFIN	0	loss
2127	CGTE_28	7	4824996	4826088	7p22.1	AP5Z1	6	gain
2128	CGTE_28	7	4827097	4856995	7p22.1	RADIL,MIR4656,AP5Z1	0	loss
2129	CGTE_28	7	44260175	44266221	7p13	CAMK2B	10	gain
2130	CGTE_28	7	98628194	99058270	7q22.1	SMURF1,ARPC1A,LOC101927550,BUD31,ATP5J2-PTCD1,ATP5J2,ARPC1B,CPSF4,MYH16,PDAP1,KPNA7,PTCD1	4	gain
2131	CGTE_28	7	142564633	142566926	7q34	EPHB6	8	gain
2132	CGTE_28	8	11400715	11711034	8p23.1	CTSB,LINC00208,BLK,FDFT1,NEIL2,SNORA99,C8orf49,GATA4	5	gain
2133	CGTE_28	8	119936671	126448739	8q24.12-q24.13	DERL1,LOC101927588,NOV,DEPTOR,FER1L6-AS2,MIR4662A,RNF139-AS1,COL14A1,KLHL38,TAF2,KIAA0196,FER1L6,ZHX1,HAS2,MTBP,NDUFB9,TRIB1,NSMCE2,ENPP2,ZHX2,COLEC10,ANXA13,TBC1D31,C8orf76,DSCC1,TRMT12,FBXO32,WDYHV1,ATAD2,ZNF572,LINC01151,TMEM65,FAM83A-AS1,FAM83A,TNFRSF11B,LOC101927543,MIR4663,LINC00964,MAL2,SNTB1,HAS2-AS1,MTSS1,LOC105375734,TATDN1,MIR4662B,MRPL13,FER1L6-AS1,ZHX1-C8orf76,RNF139,SQLE,FAM91A1,MIR6844	5	gain
2134	CGTE_28	8	127568702	136659394	8q24.22-q24.21	FAM49B,KHDRBS3,MIR30B,LOC101927798,KCNQ3,EFR3A,LRRC6,CASC11,TMEM75,ZFAT-AS1,MIR1207,ASAP1-IT1,PVT1,MIR7848,MIR5194,MIR3686,ST3GAL1,OC90,LOC101927845,SLA,PHF20L1,NCRNA00250,ADCY8,MIR1208,CASC19,LINC01591,MIR30D,ASAP1,MYC,LINC00824,CASC21,PCAT1,ZFAT,POU5F1B,MIR1206,GSDMC,WISP1,MIR1205,TG,LOC101927822,CCAT2,ASAP1-IT2,NDRG1,CCDC26,HPYR1,CASC8,FAM84B,LINC00977,MIR1204,PRNCR1,TMEM71,HHLA1,PCAT2,CCAT1	4	gain

2135	CGTE_28	9	214455	27061103	9p24.2-p21.2	KIAA2026,C9orf92,CAAP1,MIR491,SLC1A1,RCL1,GLIS3-AS1,GLDC,IFNA22P,IFT74,CD274,CDKN2B-AS1,LOC389705,ZDHHC21,CER1,SPATA6L,PUM3,RFX3-AS1,KLHL9,PLPP6,MLLT3,MTAP,SH3GL2,IFNA14,SNAPC3,IFNA2,IFNA16,PLGRKT,MIR101-2,VLDLR,KCNV2,MIR3152,MIR4474,IFNA13,HACD4,CDKN2A,DMRT2,PLIN2,LURAP1L-AS1,LRRC19,IFNA10,FOCAD,IFNA4,CNTLN,TUSC1,MPDZ,IFNA7,SLC24A2,PTPRD,SMARCA2,KANK1,VLDLR-AS1,DOCK8,FLJ41200,CDC37L1,TTC39B,SAXO1,BNC2,IFNA1,ACER2,LINC01241,SCARNA8,LINC01239,MIR31,LOC100506422,GLIS3,INSL4,JAK2,LINC01230,IFT74-AS1,IFNB1,DMRTA1,IFNA17,UHRF2,RPS6,IFNW1,ERMP1,IL33,LURAP1L,ELAVL2,SNORD137,PLAA,CDKN2A-AS1,PDCD1LG2,INSL6,KDM4C,LOC101929563,RRAGA,CDKN2B,DMRT3,RFX3,IFNA5,IFNA21,HAUS6,LINC00583,PSIP1,TMEM261,IZUMO3,CCDC171,CDC37L1-AS1,RLN1,C9orf66,TPD52L3,PTPRD-AS2,DMRT1,ADAMTSL1,MLANA,AK3,DENND4C,LINC01231,IFNA6,TYRP1,IFNA8,PTPRD-AS1,RLN2,IFNE,RANBP6,NFIB,FOCAD-AS1,FREM1,RIC1,MIR31HG,MIR4665,MIR4473	1	loss
2136	CGTE_28	9	127617918	127619187	9q33.3	WDR38	6	gain
2137	CGTE_28	9	139372026	139699148	9q34.3	LOC100128593,TMEM141,AGPAT2,MIR4673,CCDC183-AS1,EGFL7,LCN6,MIR4674,C9orf163,NOTCH1,LCN8,SEC16A,SNHG7,MIR126,SNORA17B,MIR6722,FAM69B,CCDC183,LCN15,NALT1,LCN10,SNORA17A	0	loss
2138	CGTE_28	9	139699153	139726845	9q34.3	CCDC183,MIR4292,RABL6,CCDC183-AS1	3	gain
2139	CGTE_28	9	139728155	139916049	9q34.3	C9orf142,FBXW5,EDF1,LCNL1,C8G,MAMDC4,PTGDS,MIR4479,LCN12,TRAF2,C9orf172,CLIC3,RABL6,PHPT1,ABCA2	0	loss
2140	CGTE_28	9	139916244	139918760	9q34.3	ABCA2	8	gain
2141	CGTE_28	9	139922497	140437994	9q34.3	SLC34A3,FUT7,LRRC26,RNF208,NPDC1,PNPLA7,TOR4A,NRARP,NDOR1,MIR3621,RNF224,C9orf173-AS1,ANAPC2,TMEM210,NOXA1,TMEM203,ENTPD8,UAP1L1,C9orf139,EXD3,NSMF,MIR7114,C9orf173,SSNA1,GRIN1,MAN1B1,ENTPD2,MAN1B1-AS1,FAM166A,TUBB4B,TPRN,NELFB,SAPCD2,ABCA2,DPP7,CYSRT1	0	loss
2142	CGTE_28	10	104158428	104161226	10q24.32	NFKB2	8	gain
2143	CGTE_28	11	193051	1775623	11p15.5	TOLLIP,RASSF7,SCT,IRF7,FAM99A,IFITM2,TMEM80,PKP3,ATHL1,IFITM5,SIRT3,CDHR5,AP2A2,LRRC56,CHID1,BRSK2,RNH1,CD151,PNPLA2,MUC5AC,SCGB1C2,LOC143666,CTSD,KRTAP5-AS1,TOLLIP-AS1,EPS8L2,KRTAP5-6,RIC8A,TSPAN4,MIR6744,POLR2L,ANO9,SLC25A22,KRTAP5-5,IFITM10,LMNTD2,B4GALNT4,MIR210,DEAF1,MUC6,DUSP8,SNORA52,PIDD1,PTDSS2,KRTAP5-2,NLRP6,CEND1,MUC2,IFITM1,SIGIRR,CRACR2B,KRTAP5-3,MUC5B,MIR210HG,KRTAP5-1,PDDC1,DRD4,RPLP2,KRTAP5-4,PANO1,MOB2,BET1L,IFITM3,HRAS,MIR6743,PSMD13,TALDO1,SCGB1C1,ODF3,FAM99B,PHRF1,LOC171391	1	loss
2144	CGTE_28	11	1776093	1782715	11p15.5	CTSD	5	gain

2145	CGTE_28	11	30516766	34460641	11p14.1-p13	CSTF3,HIPK3,DCDC5,WT1-AS,LOC100506675,PAX6,LINC00294,LMO2,ELP4,IMMP1L,CAT,C11orf91,DNAJC24,FBXO3-AS1,DEPDC7,TCP11L1,ABTB2,CCDC73,PAUPAR,DCDC1,FBXO3,WT1,CAPRIN1,CD59,PRRG4,NAT10,DKFZp686K1684,CSTF3-AS1,RCN1,EIF3M,QSER1,KIAA1549L,SNORA88,MPPED2	3	gain
2146	CGTE_28	11	34470772	36669776	11p13-p12	CD44,LDLRAD3,TRAF6,PRR5L,LOC100507144,APIP,MIR3973,EHF,RAG2,TRIM44,PAMR1,SLC1A2,COMMD9,FJX1,PDHX,RAG1,C11orf74,ELF5,CAT,MIR1343	8	gain
2147	CGTE_28	11	36680477	45230860	11p11.2-p12	TTC17,MIR129-2,ALKBH3-AS1,C11orf96,ALKBH3,ACCS,SEC14L1P1,EXT2,PRDM11,C11orf74,LINC01499,API5,TP53I11,CD82,ALX4,LOC221122,MIR670,LRRC4C,TSPAN18,LINC01493,HSD17B12,MIR670HG,ACCSL,LOC103312105,LOC100507205,HNRNPKP3	4	gain
2148	CGTE_28	11	45233965	46529848	11p11.2	DGKZ,MDK,PEX16,LOC100507384,SYT13,MIR3160-2,MAPK8IP1,MIR4688,PRDM11,PHF21A,AMBRA1,CREB3L1,GYLTL1B,LOC101928894,CHRM4,SLC35C1,MIR7154,CHST1,CRY2,LOC101928812,LOC399886,DKFZp779M0652,MIR3160-1,C11orf94	3	gain
2149	CGTE_28	11	46529852	47159285	11p11.2	SNORD67,CKAP5,LRP4,F2,C11orf49,ARHGAP1,HARBI1,ATG13,AMBRA1,LRP4-AS1,MIR5582,ZNF408	4	gain
2150	CGTE_28	11	47176690	48239315	11p11.2	PSMC3,FAM180B,MIR6745,NR1H3,MYBPC3,PTPRJ,OR4B1,NDUFS3,LOC101928943,MIR4487,RAPSN,KBTBD4,SPI1,ARFGAP2,MTCH2,FNBP4,MADD,SLC39A13,PTPMT1,C1QTNF4,C11orf49,PACSIN3,CELF1,AGBL2,ACP2,DDB2,MIR3161,NUP160	3	gain
2151	CGTE_28	11	67169135	67829526	11q13.2	ALDH3B2,PITPNM1,CHKA,PPP1CA,GPR152,CDK2AP2,CABP2,TBX10,FAM86C2P,CABP4,DOC2GP,ALDH3B1,CARNS1,TCIRG1,GSTP1,ACY3,MIR7113,NUDT8,AIP,TBC1D10C,RPS6KB2,PTPRCAP,MIR4691,NDUFV1,C11orf72,MIR6753,UNC93B1,NDUFS8,CORO1B,TMEM134,MIR6752	0	loss
2152	CGTE_28	11	74904299	75168834	11q13.4	SNORD15B,RPS3,MIR326,ARRB1,SLCO2B1,SNORD15A,GDPD5,TPBGL,KLHL35	3	gain
2153	CGTE_28	12	24970877	27066614	12p11.23-p12.1	KRAS,MIR4302,ITPR2,C12orf77,LMNTD1,ASUN,RASSF8-AS1,BCAT1,SSPN,LRMP,RASSF8,BHLHE41,LYRM5,CASC1	8	gain
2154	CGTE_28	12	27066857	27950359	12p11.23-p11.22	FGFR1OP2,REP15,ARNTL2,ARNTL2-AS1,MED21,SMCO2,MRPS35,MANSC4,ASUN,STK38L,TM7SF3,KLHL42,C12orf71,PPFIBP1	4	gain
2155	CGTE_28	12	27950605	33030870	12p11.21-p11.22	YARS2,DDX11,PKP2,OVCH1,TMTC1,DENND5B,FAR2,DNM1L,LINC00941,PTHLH,DDX11-AS1,FGD4,AMN1,KIAA1551,OVCH1-AS1,BICD1,FAM60A,FLJ13224,TSPAN11,CCDC91,ERGIC2,CAPRIN2,H3F3C,DENND5B-AS1,KLHL42,LOC100506606,IPO8,METTL20	3	gain
2156	CGTE_28	12	48370605	48380214	12q13.11	COL2A1	3	gain
2157	CGTE_28	12	112191210	112630976	12q24.13-q24.12	MAPKAPK5,HECTD4,ACAD10,TRAFD1,NAA25,TMEM116,MIR6761,MIR6861,ERP29,MIR3657,ADAM1A,MAPKAPK5-AS1,ALDH2	3	gain
2158	CGTE_28	12	124779984	124857156	12q24.31	FAM101A,MIR6880,ZNF664-FAM101A,NCOR2	0	loss
2159	CGTE_28	13	20657803	20762904	13q12.11	GJA3,GJB2,ZMYM2,LINC01072	4	gain
2160	CGTE_28	13	22070140	22088634	13q12.11	MICU2	6	gain
2161	CGTE_28	13	58206674	70293677	13q21.2-q21.1	LOC102723968,LINC00448,TDRD3,DIAPH3,LINC00434,LINC01052,PCDH20,OR7E156P,LINC00378,LINC00358,LINC00550,MIR4704,PCDH9-AS3,LINC00383,LINC00395,LINC00364,PCDH17,DIAPH3-AS2,LOC101926897,MIR3169,PCDH9,MIR548X2,LINC00374,LINC01075,DIAPH3-AS1,KLHL1,PCDH9-AS2,PCDH9-AS4,LINC00376	1	loss
2162	CGTE_28	13	114766249	115004603	13q34	CDC16,RASA3	3	gain

2163	CGTE_28	14	94038102	104565321	14q32.12-q32.32	MEG9,IFI27,MIR543,SNHG24,LINC00605,SNORD114-6,HSP90AA1,BDKRB2,MIR1193,SNORD114-2,ZNF839,MIR431,TRMT61A,SNORD114-11,LOC100129345,BAG5,MIR337,DIO3OS,BEGAIN,SNORD114-24,FAM181A,MIR300,MIR496,MIR433,MIR323A,MIR379,SNORD113-4,SNORD114-21,MIR889,PPP2R5C,MIR409,MIR369,MIR4506,MIR136,MIR370,DDX24,SNORD114-20,SNORD114-25,MIR6764,SNORA28,SERPINA1,RTL1,MIR4309,LINC00521,MIR758,LOC101929080,SNORD114-27,EML1,MIR665,MIR487B,LOC101929241,CDC42BPB,DIO3,EIF5,CLMN,MIR345,PAPOLA,SERPINA12,C14orf177,MIR1247,DICER1-AS1,SERPINA4,ASB2,ASPG,XRCC3,MARK3,MIR544A,MIR541,SNORD113-6,MIR342,SNORD114-28,MIR151B,ANKRD9,WDR25,SNORD114-3,MIR5698,EXOC3L4,SNORD114-9,MIR656,CKB,MIR1197,HHIPL1,SNHG10,SNORD114-10,MIR495,SERPINA13P,PPP4R4,TECPR2,SLC25A47,MIR376A2,PPP1R13B,LINC00524,AMN,SLC25A29,C14orf132,SNORD114-14,TRAF3,AK7,DLK1,MIR381HG,RCOR1,WDR20,SYNE3,MIR655,MIR299,MEG3,SNORD113-1,SERPINA11,MIR376C,SNORD114-15,MIR770,MIR1185-2,MIR2392,SNORD114-16,PRIMA1,EVL,MIR3173,MIR539,LINC00637,YY1,MIR134,MIR380,MIR485,KLC1,SNORD113-2,LINC00239,APOPT1,TDRD9,MIR323B,MIR329-2,IFI27L1,SNORD113-5,SNORD114-30,MIR493,GSC,TCL1A,FAM181A-AS1,SERPINA10,IFI27L2,SNORD113-7,MIR382,UNC79,SNORD114-4,LINC00341,SNORD114-19,LINC00523,SNORD114-29,MIR376B,ZFYVE21,MIR432,SERPINA3,TCL1B,LINC01550,WARS,LOC730202,MIR411,SNORD114-23,ATG2B,RD3L,MIR376A1,SNORD114-26,SNORD114-7,DICER1,SNORD114-17,SETD3,TUNAR,CYP46A1,MOK,LINC00618,SNORD114-5,SERPINA6,SERPINA5,CCNK,SERPINA2,SNORD114-12,SNORD114-8,C14orf2,SNORD114-22,SNORD114-1,TNFAIP2,MIR410,CCDC85C,GSKIP,MIR668,SERPINA9,MIR494,MIR381,SCARNA13,SNORD113-9,TCL6,BDKRB1,MEG8,BCL11B,MIR329-1,SNORD114-31,CINP,MIR654,MIR154,DYNC1H1,GLRX5,MIR487A,DEGS2,SNORD114-18,MIR1185-1,MIR127,OTUB2,SNORD114-13,MIR377,MIR412,VRK1	3	gain
2164	CGTE_28	14	104569793	105181171	14q32.33	MIR4710,MIR203A,ASPG,INF2,TMEM179,KIF26A,C14orf180,MIR203B	0	loss
2165	CGTE_28	14	105181544	105242184	14q32.33	SIVA1,ADSSL1,AKT1,INF2	4	gain
2166	CGTE_28	14	105242927	105412546	14q32.33	CEP170B,AKT1,AHNAK2,PLD4,ZBTB42,LINC00638	0	loss
2167	CGTE_28	14	105412779	105416196	14q32.33	AHNAK2	11	gain
2168	CGTE_28	14	105417827	105609490	14q32.33	GPR132,CDCA4,AHNAK2,JAG2,C14orf79,LOC102723354	4	gain
2169	CGTE_28	14	105609769	105677039	14q32.33	BRF1,MIR6765,JAG2,NUDT14	0	loss
2170	CGTE_28	14	105677348	105834901	14q32.33	BTBD6,PACS2,BRF1	9	gain
2171	CGTE_28	14	105836124	107283148	14q32.33	MIR7641-2,ELK2AP,KIAA0125,MIR8071-2,ADAM6,LINC00221,CRIP1,MIR4507,MIR4538,CRIP2,MIR4539,LOC100507437,MIR8071-1,PACS2,TMEM121,MIR5195,C14orf80,TEX22,MIR4537,MTA1,LINC00226	3	gain
2172	CGTE_28	15	34060720	34657886	15q14	NOP10,KATNBL1,NUTM1,AVEN,PGBD4,SLC12A6,LPCAT4,EMC4,CHRM5,EMC7,RYR3	3	gain
2173	CGTE_28	15	35274279	35674153	15q14	ANP32AP1,ZNF770,MIR3942,DPH6	3	gain
2174	CGTE_28	15	40630166	40631827	15q15.1	C15orf52	4	gain
2175	CGTE_28	15	42137000	42166625	15q15.1	JMJD7-PLA2G4B,MIR4310,PLA2G4B,SPTBN5	0	loss

2176	CGTE_28	15	42166913	42168853	15q15.1	SPTBN5	7	gain
2177	CGTE_28	15	42168948	42170978	15q15.1	SPTBN5	0	loss
2178	CGTE_28	15	43028035	43733836	15q15.3-q15.2	ZSCAN29,TGM7,CDAN1,TGM5,LCMT2,UBR1,TTBK2,TMEM62,TUBGCP4,CCNDBP1,EPB42,ADAL,TP53BP1	3	gain
2179	CGTE_28	15	68098780	68116354	15q23	MAP2K5,SKOR1	5	gain
2180	CGTE_28	15	75090918	75093312	15q24.1	CSK	7	gain
2181	CGTE_28	15	77325007	77329533	15q24.3	PSTPIP1	5	gain
2182	CGTE_28	15	79063629	79170714	15q25.1	MORF4L1,ADAMTS7	0	loss
2183	CGTE_28	15	90171648	90185750	15q26.1	KIF7	0	loss
2184	CGTE_28	15	90188161	90188851	15q26.1	KIF7	38	gain
2185	CGTE_28	15	101417996	102389808	15q26.3	LRRK1,OR4F13P,PCSK6-AS1,CHSY1,LOC100507472,TM2D3,OR4F15,VIMP,SNRPA1,ALDH1A3,OR4F6,PCSK6,TARSL2	3	gain
2186	CGTE_28	16	309921	313422	16p13.3	FAM234A	7	gain
2187	CGTE_28	16	313640	2098753	16p13.3	TPSG1,FAM234A,TBL3,IFT140,NME4,NPW,CLCN7,TSC2,TPSD1,SNORA10,LOC100134368,SYNGR3,HS3ST6,CACNA1H,RAB40C,WDR90,TPSAB1,RNF151,MEIOB,DECR2,C16orf91,RHBDL1,UNKL,TMEM8A,SSTR5-AS1,NME3,NTHL1,TMEM204,RPL3L,LMF1-AS1,FAM195A,TPSB2,NOXO1,CHTF18,ARHGDIG,IGFALS,LMF1,TSR3,MSRB1,HN1L,MRPL28,CAPN15,MRPS34,PTX4,MSLN,PDIA2,RGS11,JMJD8,RAB11FIP3,MIR3177,FBXL16,PRR35,TELO2,BAIAP3,WFIKKN1,MAPK8IP3,SOX8,STUB1,C1QTNF8,ZNF598,GFER,SLC9A3R2,HAGHL,PIGQ,AXIN1,C16orf13,FAM173A,WDR24,NARFL,RPUSD1,SPSB3,HAGH,LINC00235,NUBP2,CRAMP1,MIR3176,GNG13,CCDC78,LINC00254,GNPTG,NDUFB10,EME2,MIR662,NHLRC4,METRN,SNORA64,FAHD1,MIR5587,RPS2,PRR25,SNHG9,CCDC154,SNORA78,UBE2I,RHOT2,SSTR5	0	loss
2188	CGTE_28	16	2100378	2107366	16p13.3	TSC2	5	gain
2189	CGTE_28	16	4864364	5058552	16p13.3	PPL,UBN1,SEC14L5,GLYR1	3	gain
2190	CGTE_28	16	5058555	5065133	16p13.3	SEC14L5	6	gain
2191	CGTE_28	16	30123407	30124450	16p11.2	GDPD3	7	gain
2192	CGTE_28	16	75276464	75485803	16q23.1	TMEM170A,BCAR1,CFDP1	4	gain
2193	CGTE_28	16	75498110	75564300	16q23.1	TMEM170A,CHST5,CHST6	0	loss
2194	CGTE_28	16	75565505	75794040	16q23.1	TMEM231,CHST5,ADAT1,GABARAPL2,TERF2IP,KARS	3	gain
2195	CGTE_28	16	88792913	88874836	16q24.3	LOC100289580,LOC339059,CDT1,PIEZO1	0	loss
2196	CGTE_28	17	37665878	37871794	17q12	PPP1R1B,STARD3,TCAP,PNMT,CDK12,ERBB2,NEUROD2,PGAP3	45	gain
2197	CGTE_28	17	37871975	37949204	17q12	IKZF3,ERBB2,GRB7,MIEN1,MIR4728	38	gain
2198	CGTE_28	17	48191056	48696127	17q21.33	COL1A1,PPP1R9B,LRRC59,SAMD14,LOC101927230,RSAD1,MRPL27,XYLT2,SGCA,SPATA20,EME1,MYCBPAP,CHAD,CACNA1G,ACSF2,EPN3,HILS1,TMEM92,CACNA1G-AS1,TMEM92-AS1	3	gain
2199	CGTE_28	17	57937667	57952069	17q23.1	TUBD1	13	gain
2200	CGTE_28	18	163296	905504	18p11.32	ENOSF1,CETN1,CLUL1,TYMSOS,COLEC12,YES1,THOC1,TYMS,USP14,ADCYAP1	3	gain
2201	CGTE_28	18	20602088	21107960	18q11.2	RBBP8,C18orf8,CABLES1,TMEM241,RIOK3	3	gain
2202	CGTE_28	18	55317465	55816855	18q21.31	NEDD4L,ATP8B1,LOC100505549	3	gain
2203	CGTE_28	18	77156185	77171501	18q23	NFATC1	0	loss
2204	CGTE_28	18	77193615	77208984	18q23	NFATC1	18	gain
2205	CGTE_28	18	77210916	78005277	18q23	RBFA,NFATC1,PQLC1,HSBP1L1,ADNP2,CTDP1,KCNG2,TXNL4A,PARD6G,RBFADN,PARD6G-AS1	3	gain

2206	CGTE_28	19	281411	1905500	19p13.3	FGF22,POLRMT,HCN2,SCAMP4,MADCAM1,REEP6,APC2,ONECUT3,LOC100288123,CBARP,FSTL3,MBD3,ARID3A,ODF3L2,MIDN,RPS15,CDC34,C19orf24,PLPP2,STK11,REXO1,GZMM,PALM,PLK5,UQCR11,PCSK4,CIRBP-AS1,BSG,EFNA2,SHC2,MIR3187,NDUFS7,C19orf25,CFD,MISP,C2CD4C,MED16,SBNO2,TMEM259,PRTN3,ATP8B3,RNU6-2,WDR18,PLPPR3,ADAMTSL5,ATP5D,TCF3,MIER2,ABHD17A,MUM1,CIRBP,MIR4745,CNN2,HMHA1,PRSS57,RNF126,KISS1R,GRIN3B,PTBP1,ADAT3,MIR1909,GPX4,MEX3D,THEG,TPGS1,ELANE,POLR2E,ABCA7,DAZAP1,AZU1,KLF16,R3HDM4,GAMT	0	loss
2207	CGTE_28	19	15465745	17001375	19p13.12-p13.11	CYP4F12,HSH2D,NWD1,MED26,CYP4F2,SMIM7,CYP4F3,OR10H5,AKAP8L,F2RL3,OR10H1,AP1M1,RAB8A,CIB3,UCA1,EPS15L1,CYP4F22,CYP4F24P,AKAP8,KLF2,WIZ,CALR3,LOC102724279,FAM32A,OR10H2,PGLYRP2,CHERP,SLC35E1,CYP4F11,LINC00905,LINC00661,OR10H3,MIR1470,OR10H4,TMEM38A,TPM4,SIN3B,C19orf44,CYP4F8,RASAL3	3	gain
2208	CGTE_28	19	17003704	17063032	19p13.11	CPAMD8	5	gain
2209	CGTE_28	19	17441585	17732948	19p13.11	BST2,ANO8,NXNL1,BISPR,UNC13A,TMEM221,PLVAP,COLGALT1,GTPBP3,SLC27A1,FAM129C,PGLS,MVB12A	4	gain
2210	CGTE_28	19	30018053	30506560	19q12	PLEKHF1,VSTM2B,C19orf12,URI1,CCNE1,POP4	11	gain
2211	CGTE_28	19	45213713	45494636	19q13.32	APOC1,APOC2,TOMM40,APOE,APOC1P1,BCAM,CLPTM1,CEACAM16,APOC4-APOC2,APOC4,MIR8085,BCL3,PVRL2,CBLC	3	gain
2212	CGTE_28	19	55624763	55991386	19q13.42	PTPRH,HSPBP1,RPL28,FAM71E2,SYT5,PPP6R1,MIR6803,UBE2S,MIR6802,TNNT1,SHISA7,TNNI3,MIR6804,ISOC2,MIR6805,PPP1R12C,MIR7975,TMEM238,TMEM150B,COX6B2,BRSK1,IL11,ZNF628,TMEM86B,KMT5C,TMEM190,DNAAF3	4	gain
2213	CGTE_28	19	56969427	58864860	19q13.43	ZNF749,ZNF419,ZNF264,ZIM2-AS1,A1BG-AS1,ZNF417,ZSCAN4,DUXA,ZIM2,C19orf18,ZNF329,SMIM17,ZNF667,ZNF587B,ZNF154,MIR6806,ZNF549,ZNF814,ZNF135,ZNF773,ZNF671,ZNF835,TRAPPC2B,ZNF552,ZNF304,ZNF543,ZNF550,ZNF547,ZNF776,ZNF211,ZNF667-AS1,ZIK1,USP29,ZNF470,A1BG,FKBP1AP1,ZNF418,ZNF805,VN1R1,ZNF256,ZNF606,ZNF548,PEG3-AS1,ZNF8,PEG3,ZNF530,ZNF587,ZNF586,AURKC,LOC100128398,ZNF460,ZNF71,ZNF416,MIMT1,ZNF471,ZSCAN18,ZNF17,ZNF772,ZNF544,ZSCAN1,ZNF274,ZIM3,ZSCAN22,ZNF551,ZFP28,ZNF134	5	gain
2214	CGTE_28	19	58867388	58898360	19q13.43	ZNF837,MIR4754,ZNF497	0	loss
2215	CGTE_28	19	58899600	59093745	19q13.43	MZF1,ZNF584,ZNF324,ZBTB45,TRIM28,ZNF324B,UBE2M,ZNF446,CHMP2A,RNF225,MZF1-AS1,RPS5,ZNF132,MIR6807,SLC27A5,CENPBD1P1	5	gain
2216	CGTE_28	20	15210540	15480562	20p12.1	MACROD2	9	gain
2217	CGTE_28	20	25011760	25394490	20p11.21	GINS1,LOC284798,LOC101926889,PYGB,ACSS1,VSX1,ENTPD6,ABHD12	3	gain
2218	CGTE_28	20	31885315	32358106	20q11.22-q11.21	CBFA2T2,PXMP4,SNTA1,ZNF341,NECAB3,ACTL10,C20orf144,BPIFB1,E2F1,CDK5RAP1	3	gain
2219	CGTE_28	20	60891582	61290188	20q13.33	LAMA5,SLCO4A1,C20orf166-AS1,MIR1-1,LAMA5-AS1,RBBP8NL,RPS21,MIR1-1HG,GATA5,CABLES2,MIR4758,MIR133A2	0	loss
2220	CGTE_28	20	61937229	61981794	20q13.33	CHRNA4,COL20A1	0	loss
2221	CGTE_28	21	42647170	43414359	21q22.3	C2CD2,FAM3B,MX2,MX1,LINC00112,RIPK4,SNORA91,BACE2,LINC00111,ZBTB21,TMPRSS2,LINC00479,PRDM15,MIR6814	1	loss
2222	CGTE_28	21	45103124	45175654	21q22.3	RRP1B,PDXK	4	gain
2223	CGTE_28	21	45971009	46057457	21q22.3	KRTAP10-5,KRTAP10-6,KRTAP10-9,KRTAP10-7,KRTAP10-10,TSPEAR,KRTAP10-3,KRTAP10-8,KRTAP10-2,KRTAP10-4	0	loss
2224	CGTE_28	21	46057516	46067022	21q22.3	KRTAP10-11,KRTAP10-10,TSPEAR	7	gain
2225	CGTE_28	22	40641922	40803008	22q13.1	TNRC6B,ADSL,SGSM3	5	gain

2226	CGTE_28	22	40803033	41368561	22q13.1-q13.2	ST13,XPNPEP3,SGSM3,MCHR1,MIR4766,MKL1,SLC25A17,DNAJB7,LOC101927257,RBX1	4	gain
2227	CGTE_28	22	46751346	46787801	22q13.31	CELSR1,TRMU	0	loss
2228	CGTE_28	22	46790056	50321250	22q13.31-q13.33	CELSR1,MIR4535,GRAMD4,ALG12,BRD1,CERK,MIR3667,LOC284930,FAM19A5,CRELD2,C22orf34,LOC284933,TBC1D22A,ZBED4,MIR3201,TBC1D22A-AS1,LINC01310,LINC00898,LL22NC03-75H12.2	3	gain
2229	CGTE_28	22	50354564	50470540	22q13.33	IL17REL,PIM3,MIR6821	0	loss
2230	CGTE_28	22	50471620	50599898	22q13.33	MOV10L1,MLC1	3	gain
2231	CGTE_28	22	50609165	50752306	22q13.33	DENND6B,MAPK11,HDAC10,PANX2,MAPK12,SELO,PLXNB2,TRABD,TUBGCP6	0	loss
2232	CGTE_28	22	50752426	50752992	22q13.33	DENND6B	6	gain
2233	CGTE_28	22	50752995	50946884	22q13.33	LMF2,SBF1,NCAPH2,ADM2,DENND6B,PPP6R2,MIOX	0	loss
2234	CGTE_28	22	50954146	50957173	22q13.33	NCAPH2	4	gain
2235	CGTE_28	X	106110009	106114056	Xq22.3	TBC1D8B	3	gain
2236	CGTE_28	X	152806771	152807402	Xq28	ATP2B3	5	gain
2237	CGTE_28	X	152913426	152915194	Xq28	DUSP9	5	gain
2238	CGTE_28	X	152915382	153043159	Xq28	SLC6A8,PLXNB3,BCAP31,DUSP9,ABCD1,PNCK	0	loss
2239	CGTE_28	X	153043399	153045904	Xq28	PLXNB3	6	gain
2240	CGTE_28	X	153046586	153051781	Xq28	SRPK3,IDH3G	0	loss
2241	CGTE_28	X	153220574	153221907	Xq28	HCFC1	0	loss
2242	CGTE_28	X	153590322	153596543	Xq28	FLNA	0	loss
2243	CGTE_28	X	153689414	153697115	Xq28	PLXNA3	0	loss
2244	CGTE_28	Y	14834062	15481254	Yq11.221-Yq11.21	UTY,USP9Y,DDX3Y	0	loss
2245	CGTE_28	Y	15522890	21759666	Yq11.221-Yq11.222	TTTY14,NLGN4Y-AS1,UTY,NLGN4Y,FAM41AY2,CDY2B,VCY1B,CDY2A,XKRY2,HSFY2,FAM224B,FAM224A,TTTY9B,XKRY,VCY,CD24,FAM41AY1,BCORP1,TTTY9A,TMSB4Y,HSFY1,TXLNGY	0	loss
2246	CGTE_28	Y	21765690	24460980	Yq11.222-Yq11.223	TTTY6B,RBMY1D,PRY,TTTY13,RBMY1J,PRY2,RBMY1B,RPS4Y2,RBMY1F,RBMY1E,RBMY2EP,LOC101929148,TTTY6,RBMY1A1,KDM5D,TTTY10,RBMY2FP,TXLNGY,EIF1AY,TTTY5,PRORY	0	loss
2247	CGTE_29	1	1007401	1018387	1p36.33	C1orf159,RNF223	8	gain
2248	CGTE_29	1	1178371	1179432	1p36.33	FAM132A	7	gain
2249	CGTE_29	1	3410927	3413331	1p36.32	MEGF6	5	gain
2250	CGTE_29	1	6266598	6269827	1p36.31	RNF207	6	gain
2251	CGTE_29	1	40366379	40421261	1p34.2	MYCL,MFSD2A	6	gain
2252	CGTE_29	1	115417049	115428307	1p13.2	SYCP1	4	gain
2253	CGTE_29	1	155657760	155658790	1q22	YY1AP1	65	gain
2254	CGTE_29	1	228210513	228247261	1q42.13	WNT3A	5	gain
2255	CGTE_29	1	228247849	228290484	1q42.13	MIR3620,C1orf35,ARF1,WNT3A	1	loss
2256	CGTE_29	1	228463390	228464436	1q42.13	OBSCN	14	gain
2257	CGTE_29	2	26950837	26987373	2p23.3	SLC35F6,KCNK3	5	gain
2258	CGTE_29	2	73052921	73114960	2p13.2	SPR,EXOC6B	34	gain
2259	CGTE_29	2	73492461	73493598	2p13.2	FBXO41	3	gain
2260	CGTE_29	2	73493623	73520865	2p13.2-p13.1	EGR4,FBXO41	1	loss
2261	CGTE_29	2	74741195	74743220	2p13.1	TLX2	0	loss
2262	CGTE_29	2	95540530	95691686	2q11.1	TEKT4,LOC442028,MAL	7	gain
2263	CGTE_29	2	114684854	114688902	2q14.1	ACTR3	6	gain
2264	CGTE_29	2	177036459	177134590	2q31.1	HOXD3,MTX2,HAGLR,HOXD1,HAGLROS,MIR7704	5	gain
2265	CGTE_29	2	219757393	219867844	2q35	LINC01494,LOC100129175,FEV,CFAP65,LINC00608,MIR375,CDK5R2,CRYBA2,WNT10A	3	gain
2266	CGTE_29	2	239009324	239037496	2q37.3	ESPNL	3	gain

2267	CGTE_29	3	48936127	48956549	3p21.31	ARIH2OS,ARIH2,SLC25A20	6	gain
2268	CGTE_29	3	138724504	138739537	3q23	PRR23B,PRR23A	15	gain
2269	CGTE_29	4	960751	961935	4p16.3	DGKQ	0	loss
2270	CGTE_29	4	995981	1006362	4p16.3	IDUA,FGFRL1	1	loss
2271	CGTE_29	4	1807041	1809026	4p16.3	FGFR3	3	gain
2272	CGTE_29	4	2010470	2043840	4p16.3	NELFA,C4orf48	5	gain
2273	CGTE_29	4	8582721	8594854	4p16.1	GPR78,CPZ	4	gain
2274	CGTE_29	4	13485654	13544271	4p15.33	LINC01097,RAB28,NKX3-2	4	gain
2275	CGTE_29	5	2749447	2755292	5p15.33	C5orf38,IRX2	3	gain
2276	CGTE_29	5	92920861	92924208	5q15	NR2F1,MIR548AO	7	gain
2277	CGTE_29	5	140188016	140188988	5q31.3	PCDHA1,PCDHA4,PCDHA3,PCDHA2	6	gain
2278	CGTE_29	5	140208384	140209716	5q31.3	PCDHA4,PCDHA1,PCDHA3,PCDHA5,PCDHA2,PCDHA6	6	gain
2279	CGTE_29	5	140256442	140257143	5q31.3	PCDHA9,PCDHA6,PCDHA2,PCDHA5,PCDHA3,PCDHA8,PCDHA10,PCDHA11,PCDHA1,PCDHA4,PCDHA12,PCDHA7	6	gain
2280	CGTE_29	5	176023753	176025158	5q35.2	GPRIN1	5	gain
2281	CGTE_29	6	29910395	29911267	6p22.1	HLA-A	6	gain
2282	CGTE_29	6	31324110	31371612	6p21.33	MICA,HLA-B	5	gain
2283	CGTE_29	6	37616725	37617791	6p21.2	MDGA1	0	loss
2284	CGTE_29	6	127836618	127837673	6q22.33	SOGA3	51	gain
2285	CGTE_29	7	72722337	72727628	7q11.23	TRIM50,NSUN5	7	gain
2286	CGTE_29	7	100806233	100808125	7q22.1	VGF	13	gain
2287	CGTE_29	8	144662852	144668620	8q24.3	EEF1D	11	gain
2288	CGTE_29	8	145006091	145008335	8q24.3	PLEC	8	gain
2289	CGTE_29	8	145008455	145016663	8q24.3	PLEC	1	loss
2290	CGTE_29	9	124989598	124991118	9q33.2	LHX6	9	gain
2291	CGTE_29	9	139398936	139401157	9q34.3	NOTCH1	6	gain
2292	CGTE_29	9	140120446	140123594	9q34.3	RNF224,CYSRT1	50	gain
2293	CGTE_29	9	140327886	140329812	9q34.3	ENTPD8,NOXA1	9	gain
2294	CGTE_29	10	93002	243989	10p15.3	TUBB8,ZMYND11	4	gain
2295	CGTE_29	10	27702744	27793808	10p12.1	PTCHD3,RAB18	9	gain
2296	CGTE_29	10	35896546	35928369	10p11.21	FZD8,GJD4	11	gain
2297	CGTE_29	10	35928432	35929717	10p11.21	FZD8	0	loss
2298	CGTE_29	10	76994622	77159485	10q22.2	ZNF503-AS1,COMTD1,ZNF503	0	loss
2299	CGTE_29	10	99079047	99080200	10q24.1	FRAT1	34	gain
2300	CGTE_29	10	102586739	102673061	10q24.31	PAX2,SLF2	4	gain
2301	CGTE_29	10	102893995	102988367	10q24.31	LINC01514,LBX1,TLX1NB,TLX1	3	gain
2302	CGTE_29	11	64135895	64139065	11q13.1	MIR1237,RPS6KA4	5	gain
2303	CGTE_29	12	208094	248527	12p13.33	IQSEC3,LOC574538	3	gain
2304	CGTE_29	12	4877557	4919751	12p13.32	GALNT8,KCNA6	5	gain
2305	CGTE_29	12	49363872	49375091	12q13.12	WNT1,WNT10B	3	gain
2306	CGTE_29	12	52699791	52708652	12q13.13	KRT83,KRT86	7	gain
2307	CGTE_29	12	63195555	63328560	12q14.2	PPM1H	4	gain
2308	CGTE_29	12	110883325	110888184	12q24.11	ARPC3	8	gain
2309	CGTE_29	12	132400404	132404156	12q24.33	ULK1	4	gain
2310	CGTE_29	12	133158221	133160392	12q24.33	MIR6763,FBRSL1	7	gain
2311	CGTE_29	13	38934079	39262135	13q13.3	LINC00366,UFM1,LINC00437,FREM2	5	gain
2312	CGTE_29	13	52004448	52026733	13q14.3	INTS6	6	gain
2313	CGTE_29	13	53420381	53421740	13q14.3	PCDH8	0	loss
2314	CGTE_29	13	60737611	61013821	13q21.2	TDRD3,LINC00434,DIAPH3	6	gain
2315	CGTE_29	13	110434503	110438412	13q34	IRS2	0	loss
2316	CGTE_29	14	34268878	34270267	14q13.1	NPAS3	5	gain

2317	CGTE_29	14	70038179	70040340	14q24.1	CCDC177	0	loss
2318	CGTE_29	14	74035917	74036650	14q24.3	ACOT2	1	loss
2319	CGTE_29	14	88789958	88852272	14q31.3	SPATA7,KCNK10	8	gain
2320	CGTE_29	14	89258619	89291210	14q31.3	EML5,TTC8	5	gain
2321	CGTE_29	14	99640502	99642207	14q32.2	BCL11B	0	loss
2322	CGTE_29	14	103388895	103396111	14q32.32	AMN	0	loss
2323	CGTE_29	14	103987620	103988824	14q32.32	CKB	22	gain
2324	CGTE_29	14	104644924	104646154	14q32.33	KIF26A	10	gain
2325	CGTE_29	14	105350013	105353205	14q32.33	CEP170B	7	gain
2326	CGTE_29	14	105941155	105953902	14q32.33	CRIP2,CRIP1	0	loss
2327	CGTE_29	15	62352431	62360851	15q22.2	VPS13C,C2CD4A	5	gain
2328	CGTE_29	16	30537579	30545692	16p11.2	ZNF747,ZNF768	7	gain
2329	CGTE_29	17	4925590	4927393	17p13.2	KIF1C	5	gain
2330	CGTE_29	17	39182910	39334490	17q21.2	KRTAP2-4,KRTAP4-4,KRTAP4-7,KRTAP4-9,KRTAP2-1,KRTAP1-5,KRTAP4-12,KRTAP1-4,KRTAP1-1,KRTAP4-8,KRTAP4-3,KRTAP2-3,KRTAP4-5,KRTAP4-11,KRTAP4-2,KRTAP4-6,KRTAP2-2,KRTAP1-3	3	gain
2331	CGTE_29	17	42634694	42734142	17q21.31	FZD2,LINC01180,MEIOC	5	gain
2332	CGTE_29	17	70117549	70119154	17q24.3	SOX9	6	gain
2333	CGTE_29	17	77926401	77987332	17q25.3	TBC1D16	9	gain
2334	CGTE_29	18	24127679	24128654	18q11.2	KCTD1	5	gain
2335	CGTE_29	18	60052126	60191579	18q21.33	TNFRSF11A,ZCCHC2	0	loss
2336	CGTE_29	18	76740231	76754997	18q23	SALL3	5	gain
2337	CGTE_29	19	42880015	42891632	19q13.2	CNFN,MEGF8	6	gain
2338	CGTE_29	20	3765885	3777513	20p13	CDC25B,CENPB	7	gain
2339	CGTE_29	20	44518858	44520452	20q13.12	NEURL2,CTSA	5	gain
2340	CGTE_29	20	60893431	60894979	20q13.33	LAMA5	6	gain
2341	CGTE_29	20	62153065	62222147	20q13.33	HELZ2,PPDPF,SRMS,PTK6,GMEB2,C20orf195	3	gain
2342	CGTE_29	21	45553492	45681134	21q22.3	ICOSLG,DNMT3L,C21orf33	1	loss
2343	CGTE_29	21	45953454	45978626	21q22.3	TSPEAR,KRTAP10-2,KRTAP10-1,KRTAP10-3	3	gain
2344	CGTE_29	22	17589465	17590650	22q11.1	IL17RA	10	gain
2345	CGTE_29	22	39077900	39078343	22q13.1	TOMM22	8	gain
2346	CGTE_29	X	49019045	49020504	Xp11.23	MAGIX	3	gain
2347	CGTE_29	X	150396052	150565998	Xq28	VMA21	5	gain
2348	CGTE_30	1	1141655	1149611	1p36.33	TNFRSF18,TNFRSF4	3	gain
2349	CGTE_30	1	1534756	1559343	1p36.33	C1orf233,MIB2	10	gain
2350	CGTE_30	1	17281174	17281880	1p36.13	CROCC	8	gain
2351	CGTE_30	1	26487799	26496630	1p36.11	ZNF593,FAM110D	4	gain
2352	CGTE_30	1	228194779	228210633	1q42.13	WNT3A	4	gain
2353	CGTE_30	1	231472977	231473521	1q42.2	EXOC8	4	gain
2354	CGTE_30	2	9770499	9981848	2p25.1	YWHAQ	6	gain
2355	CGTE_30	2	27593013	27593834	2p23.3	EIF2B4,SNX17	4	gain
2356	CGTE_30	2	33952730	36583843	2p22.3	LOC100288911,MYADML,CRIM1,LINC01317,LINC01320,LINC01318	5	gain
2357	CGTE_30	2	73052921	73114960	2p13.2	EXOC6B,SPR	5	gain
2358	CGTE_30	2	73495801	73496753	2p13.2	FBXO41	122	gain
2359	CGTE_30	2	114513817	115822446	2q14.1	DPP10-AS3,LINC01191,ACTR3,LOC100499194,SLC35F5,DPP10,LOC101060091	3	gain
2360	CGTE_30	2	233386764	233387878	2q37.1	PRSS56	4	gain
2361	CGTE_30	3	9988031	9989767	3p25.3	PRRT3,PRRT3-AS1	13	gain
2362	CGTE_30	3	138664424	138763157	3q22.3-q23	PRR23C,PRR23A,FOXL2,PRR23B,FOXL2NB	4	gain
2363	CGTE_30	5	139283827	139422680	5q31.2	NRG2	5	gain
2364	CGTE_30	5	140176158	140263292	5q31.3	PCDHA9,PCDHA12,PCDHA8,PCDHA6,PCDHA1,PCDHA4,PCDHA2,PCDHA7,PCDHA3,PCDHA5,PCDHA11,PCDHA10,PCDHA13	3	gain

2365	CGTE_30	5	140263492	140264088	5q31.3	PCDHA9,PCDHA12,PCDHA8,PCDHA6,PCDHA1,PCDHA7,PCDHA2,PCDHA4,PCDHA5,PCDHA3,PCDHA11,PCDHA10,PCDHA13	9	gain
2366	CGTE_30	5	178770862	178977830	5q35.3	ADAMTS2,RUFY1	4	gain
2367	CGTE_30	6	42945944	42952540	6p21.1	PPP2R5D,PEX6	5	gain
2368	CGTE_30	6	84563047	84569861	6q14.2	RIPPLY2,CYB5R4	4	gain
2369	CGTE_30	6	157099101	157100701	6q25.3	ARID1B	0	loss
2370	CGTE_30	7	5569184	5633203	7p22.1	FSCN1,ACTB	12	gain
2371	CGTE_30	7	100088665	100137217	7q22.1	AGFG2,NYAP1	20	gain
2372	CGTE_30	8	6566183	6582566	8p23.1	AGPAT5	6	gain
2373	CGTE_30	8	10583649	10588092	8p23.1	SOX7	6	gain
2374	CGTE_30	8	28928030	28929920	8p12	KIF13B	4	gain
2375	CGTE_30	8	63161553	63985639	8q12.3	UG0898H09,TTPA,NKAIN3,GGH	3	gain
2376	CGTE_30	8	125334237	125384287	8q24.13	TMEM65	4	gain
2377	CGTE_30	9	138378655	138379511	9q34.3	PPP1R26	5	gain
2378	CGTE_30	9	138391467	138392982	9q34.3	MRPS2,C9orf116	5	gain
2379	CGTE_30	9	139333168	139333910	9q34.3	INPP5E	8	gain
2380	CGTE_30	9	139889157	139890225	9q34.3	CLIC3	8	gain
2381	CGTE_30	10	35896546	36813201	10p11.21	FZD8,PCAT5,GJD4,MIR4683	3	gain
2382	CGTE_30	10	76994850	77159485	10q22.2	COMTD1,ZNF503-AS1,ZNF503	5	gain
2383	CGTE_30	10	105991630	106015089	10q25.1	CFAP43,GSTO1	4	gain
2384	CGTE_30	10	120513904	120790135	10q26.11	CACUL1,NANOS1	6	gain
2385	CGTE_30	11	17742805	17757750	11p15.1	KCNC1,MYOD1	4	gain
2386	CGTE_30	12	49688945	49689674	12q13.12	LOC101927267,PRPH	8	gain
2387	CGTE_30	13	41239774	41302080	13q14.11	FOXO1,MIR320D1	128	gain
2388	CGTE_30	13	53419777	53421145	13q14.3	PCDH8	3	gain
2389	CGTE_30	13	78235542	78272705	13q22.3	MIR3665,LOC100129307,SLAIN1	7	gain
2390	CGTE_30	13	110434503	110436284	13q34	IRS2	0	loss
2391	CGTE_30	13	110436287	110438412	13q34	IRS2	8	gain
2392	CGTE_30	14	35077248	35182849	14q13.1	SNX6,CFL2	1	loss
2393	CGTE_30	14	36986429	36988419	14q13.3	NKX2-1	6	gain
2394	CGTE_30	14	50100386	50110441	14q21.3	DNAAF2,POLE2	9	gain
2395	CGTE_30	14	70038179	70040340	14q24.1	CCDC177	0	loss
2396	CGTE_30	14	77493072	77494102	14q24.3	IRF2BPL	9	gain
2397	CGTE_30	14	91957033	91975970	14q32.12	PPP4R3A	4	gain
2398	CGTE_30	14	105941155	105953902	14q32.33	CRIP1,CRIP2	0	loss
2399	CGTE_30	15	65115868	65134449	15q22.31	PIF1,PLEKHO2	5	gain
2400	CGTE_30	15	80987785	80988424	15q25.1	ABHD17C	17	gain
2401	CGTE_30	15	83735497	83776604	15q25.2	MIR4515,TM6SF1,BTBD1	0	loss
2402	CGTE_30	16	857493	863949	16p13.3	PRR25	4	gain
2403	CGTE_30	16	2835029	2849189	16p13.3	PRSS33,PRSS41	3	gain
2404	CGTE_30	16	30545859	30567352	16p11.2	ZNF764,ZNF747	11	gain
2405	CGTE_30	16	77245896	77246942	16q23.1	SYCE1L	5	gain
2406	CGTE_30	17	1959961	1961491	17p13.3	HIC1	0	loss
2407	CGTE_30	17	79095094	79096622	17q25.3	AATK	6	gain
2408	CGTE_30	18	19751067	19757114	18q11.2	GATA6	8	gain
2409	CGTE_30	18	60052126	60191579	18q21.33	TNFRSF11A,ZCCHC2	0	loss
2410	CGTE_30	19	913102	920838	19p13.3	KISS1R,R3HDM4	4	gain
2411	CGTE_30	19	1978528	1979412	19p13.3	CSNK1G2	5	gain
2412	CGTE_30	19	7676569	7678151	19p13.2	CAMSAP3	11	gain
2413	CGTE_30	19	17006547	17008815	19p13.11	CPAMD8	9	gain
2414	CGTE_30	19	18543973	18545096	19p13.11	SSBP4	8	gain

2415	CGTE_30	19	38876088	38877786	19q13.2	GGN	5	gain
2416	CGTE_30	19	56125290	56126424	19q13.42	ZNF865	4	gain
2417	CGTE_30	20	33460371	33464768	20q11.22	ACSS2,GGT7	5	gain
2418	CGTE_30	20	61637404	61810017	20q13.33	MIR124-3,LOC63930,HAR1B,HAR1A,LINC00029,BHLHE23,LINC01056	4	gain
2419	CGTE_30	20	62272717	62275763	20q13.33	STMN3	3	gain
2420	CGTE_30	22	20130396	20131241	22q11.21	ZDHHC8	4	gain
2421	CGTE_30	22	21983160	21984303	22q11.21	YDJC	4	gain
2422	CGTE_30	22	39239086	39263273	22q13.1	CBX6,NPTXR	4	gain
2423	CGTE_31	1	146756900	151542262	1q21.1-q21.3	C1orf54,GJA5,BOLA1,MTMR11,GOLPH3L,GPR89B,RPRD2,MRPS21,NBPF8,LOC101060524,PPIAL4F,CA14,ZNF687,SCNM1,BNIPL,SEMA6C,MLLT11,MIR6077,RNVU1-20,CGN,FALEC,PPIAL4A,FAM231D,HIST2H4B,FCGR1C,CTSK,VPS45,OTUD7B,PPIAL4E,ECM1,RFX5,PIP5K1A,PLEKHO1,LOC101927468,NBPF25P,PSMD4,PDZK1P1,TNFAIP8L2,FAM63A,HIST2H3D,RNVU1-19,PI4KB,FCGR1A,GJA8,TNFAIP8L2-SCNM1,HIST2H2AA4,APH1A,ENSA,MIR554,NBPF9,SETDB1,PPIAL4C,MIR4257,PSMB4,LOC103091866,PRPF3,NBPF15,ANXA9,MIR6878,ANP32E,MIR5087,HIST2H2BC,NBPF11,LYSMD1,SF3B4,HIST2H2AC,LOC645166,LINC00624,HIST2H2AB,GABPB2,CERS2,ADAMTSL4-AS1,HORMAD1,TARS2,HIST2H3A,PPIAL4D,ARNT,PRUNE,HIST2H3C,MCL1,SELENBP1,CDC42SE1,SV2A,CTSS,ACP6,NBPF14,VPS72,CIART,CHD1L,C1orf56,LOC100132057,GBAT2,HIST2H4A,POGZ,LINC00623,LINC00869,HIST2H2BF,TMOD4,HIST2H2BE,BCL9,ADAMTSL4,TUFT1,LOC388692,DRD5P2,HIST2H2AA3	3	gain
2424	CGTE_31	1	151546630	151630864	1q21.3	SNX27,TUFT1	4	gain
2425	CGTE_31	1	151633223	152130451	1q21.3	TCHHL1,LOC100132111,S100A11,LINGO4,RORC,THEM5,S100A10,RPTN,NBPF18P,RIIAD1,LOC100131107,OAZ3,TDRKH,TCHH,C2CD4D,MRPL9,SNX27,CELF3,THEM4	3	gain
2426	CGTE_31	1	152185549	152328837	1q21.3	FLG,FLG-AS1,HRNR,FLG2	4	gain
2427	CGTE_31	1	152328873	152692945	1q21.3	LCE3E,LCE3A,FLG2,LCE3C,LCE5A,CRNN,LCE3B,LCE2D,LCE2B,LCE2A,C1orf68,LCE2C,FLG-AS1,LCE3D,CRCT1,LCE4A	3	gain
2428	CGTE_31	6	26020713	26033814	6p22.2	HIST1H3A,HIST1H4B,HIST1H4A,HIST1H3B,HIST1H2AB	3	gain
2429	CGTE_31	6	30917180	30919329	6p21.33	DPCR1	4	gain
2430	CGTE_31	6	30995155	30997476	6p21.33	MUC22	4	gain
2431	CGTE_31	6	31237820	31431879	6p21.33	MIR6891,MICA,HCP5,HLA-B,HLA-C	3	gain
2432	CGTE_31	6	32489819	32725875	6p21.32	HLA-DQA2,HLA-DQB2,MIR3135B,HLA-DQB1,HLA-DRB6,HLA-DRB1,HLA-DRB5,HLA-DQA1	3	gain
2433	CGTE_31	7	94285852	95739493	7q21.3	ASB4,PDK4,DYNC1I1,PON2,PPP1R9A,PON1,PON3,PEG10	3	gain
2434	CGTE_31	7	97481717	99081751	7q21.3-q22.1	ARPC1A,PDAP1,NPTX2,PTCD1,TMEM130,BAIAP2L1,ATP5J2-PTCD1,ZNF789,CPSF4,ATP5J2,ASNS,MIR5692A1,MYH16,LMTK2,BUD31,ARPC1B,LOC101927550,BRI3,BHLHA15,MIR3609,KPNA7,SCARNA28,TRRAP,TECPR1,SMURF1,MIR5692A2,MGC72080,OCM2	3	gain
2435	CGTE_31	7	99082729	99972221	7q22.1	MCM7,ZKSCAN1,CNPY4,CYP3A5,ZSCAN21,GPC2,AP4M1,CYP3A4,PILRA,ZSCAN25,GAL3ST4,MIR6840,MIR93,AZGP1P1,STAG3,PVRIG,STAG3L5P-PVRIG2P-PILRB,ZNF394,OR2AE1,CYP3A43,AZGP1,PMS2P1,MIR106B,ZNF655,TAF6,LAMTOR4,PVRIG2P,MIR4658,COPS6,CYP3A7,MIR25,TRIM4,MBLAC1,PILRB,STAG3L5P,GATS,GJC3,C7orf43,ZNF789,ZNF3,SPDYE3,CYP3A7-CYP3A51P,ZKSCAN5,FAM200A,GS1-259H13.2	4	gain
2436	CGTE_31	7	99987536	100007332	7q22.1	ZCWPW1,PILRA	3	gain
2437	CGTE_31	7	100675877	100685166	7q22.1	MUC17	3	gain
2438	CGTE_31	11	62287532	62299606	11q12.3	AHNAK	3	gain

2439	CGTE_31	11	68701933	71293983	11q13.4-q13.3	CTTN,LOC101928443,MIR548K,PPFIA1,FGF4,KRTAP5-11,MIR6754,KRTAP5-8,SHANK2-AS3,SHANK2-AS1,MRGPRD,KRTAP5-9,FGF3,KRTAP5-7,LINC01488,FLJ42102,ANO1-AS2,DHCR7,MIR3164,NADSYN1,TPCN2,MRGPRF,IGHMBP2,SHANK2,LOC338694,CCND1,FADD,ORAOV1,MRGPRF-AS1,ANO1,KRTAP5-10,MIR3664,MYEOV,FGF19	3	gain
2440	CGTE_31	11	72794634	73100442	11q13.4	RELT,P2RY2,FCHSD2,ARHGEF17,P2RY6	3	gain
2441	CGTE_31	12	40876490	40885045	12q12	MUC19	4	gain
2442	CGTE_31	14	105408121	105419610	14q32.33	AHNAK2	3	gain
2443	CGTE_31	15	40645341	40648559	15q15.1	PHGR1	3	gain
2444	CGTE_31	18	19353558	19757114	18q11.2	MIB1,GATA6-AS1,MIR1-2,MIR133A1,GATA6,MIR133A1HG	3	gain
2445	CGTE_31	18	19761364	19994754	18q11.2	CTAGE1,GATA6	4	gain
2446	CGTE_31	18	19995474	19997832	18q11.2	CTAGE1	5	gain
2447	CGTE_31	18	30538011	31319235	18q12.1	CCDC178,ASXL3	3	gain
2448	CGTE_31	18	31319236	31326643	18q12.1	ASXL3	4	gain
2449	CGTE_31	18	31432808	32920681	18q12.2-q12.1	NOL4,ZNF271P,ZNF24,ZNF397,MAPRE2,DTNA,ZSCAN30	3	gain
2450	CGTE_31	19	20002477	20003372	19p12	ZNF253	4	gain
2451	CGTE_31	19	43093813	43772343	19q13.31-q13.2	PSG4,PSG10P,PSG9,PSG1,LOC284344,PSG7,PSG5,LIPE-AS1,PSG11,PSG3,PSG2,LOC100289650,PSG8,CEACAM8,PSG6	3	gain
2452	CGTE_31	19	55241162	55385916	19q13.42	KIR2DL4,KIR3DL2,KIR2DL3,KIR2DS4,FCAR,LOC101928804,KIR3DL1,KIR2DL1,KIR3DL3	3	gain
2453	CGTE_31	20	33343888	35155619	20q11.23-q11.22	AAR2,MIR499B,TRPC4AP,UQCC1,ROMO1,SCAND1,MMP24-AS1,CPNE1,EPB41L1,ACSS2,EDEM2,GSS,DLGAP4,ERGIC3,MIR1289-1,DLGAP4-AS1,RBM12,CEP250,GGT7,HMGB3P1,MYH7B,PHF20,MMP24,EIF6,RBM39,MIR499A,FER1L4,NCOA6,LINC00657,SPAG4,PROCR,FAM83C,FAM83C-AS1,CNBD2,GDF5,C20orf173,NFS1	3	gain
2454	CGTE_31	20	57599028	61598860	20q13.32-q13.33	LOC729296,OGFR,ADRM1,OGFR-AS1,NTSR1,MIR4758,SS18L1,EDN3,COL9A3,MIR3195,MIR4533,TAF4,PRELID3B,SLCO4A1-AS1,CABLES2,LAMA5,MTG2,LOC100506384,MIR646,LSM14B,FAM217B,PSMA7,LOC101928048,C20orf197,GID8,MIR1257,MIR1-1HG,SLC17A9,SYCP2,LAMA5-AS1,RBBP8NL,C20orf166-AS1,OSBPL2,GATA5,MRGBP,HRH3,DPH3P1,SLCO4A1,PPP1R3D,MIR133A2,PHACTR3,ZNF831,LINC00659,MIR1-1,MIR548AG2,DIDO1,LOC100506470,TCFL5,MIR646HG,RPS21,CDH4,TUBB1,ATP5E,CDH26,SLMO2-ATP5E	3	gain
2455	CGTE_31	21	45970507	45971322	21q22.3	TSPEAR,KRTAP10-2	3	gain
2456	CGTE_31	21	47862306	47863873	21q22.3	PCNT	3	gain
2457	CGTE_31	22	20130396	20229420	22q11.21	LOC284865,RTN4R,CCDC188,LINC00896,ZDHHC8	1	loss
2458	CGTE_31	22	29885087	29886598	22q12.2	NEFH	3	gain
2459	CGTE_31	X	37003718	37027779	Xp21.1	FAM47C	3	gain
2460	CGTE_31	X	124454824	124456486	Xq25	LOC100129520	3	gain
2461	CGTE_32	1	861266	1564479	1p36.33	LINC01342,MIR429,B3GALT6,MIR6726,PLEKHN1,HES4,UBE2J2,ATAD3C,FAM132A,MXRA8,TTLL10,MIR200B,SSU72,SCNN1D,LOC102724312,ATAD3A,SDF4,CPSF3L,ACAP3,AGRN,CPTP,PERM1,DVL1,MIB2,MIR6727,C1orf159,ANKRD65,TMEM240,MIR6808,ISG15,TMEM88B,TNFRSF18,ATAD3B,VWA1,MIR200A,NOC2L,PUSL1,TNFRSF4,MRPL20,CCNL2,TAS1R3,LOC148413,C1orf233,RNF223,SAMD11,KLHL17,AURKAIP1	0	loss
2462	CGTE_32	1	3410264	3417222	1p36.32	MEGF6	0	loss

2463	CGTE_32	1	26670248	26673154	1p36.11	AIM1L	6	gain
2464	CGTE_32	1	109810627	109814527	1p13.3	CELSR2	7	gain
2465	CGTE_32	2	128381736	128388817	2q14.3	MYO7B	6	gain
2466	CGTE_32	2	239006590	239056757	2q37.3	UBE2F-SCLY,SCLY,KLHL30,ESPNL	0	loss
2467	CGTE_32	3	47040866	47049379	3p21.31	NBEAL2	3	gain
2468	CGTE_32	3	48602227	48610403	3p21.31	COL7A1	3	gain
2469	CGTE_32	3	52263090	52265282	3p21.2	TWF2	16	gain
2470	CGTE_32	3	52521197	52559166	3p21.1	NISCH,NT5DC2,STAB1	3	gain
2471	CGTE_32	3	127387137	127413943	3q21.3	PODXL2,MGLL,ABTB1	3	gain
2472	CGTE_32	3	196669188	196675344	3q29	PIGZ,NCBP2,NCBP2-AS2	12	gain
2473	CGTE_32	4	952203	1211207	4p16.3	CTBP1-AS,LOC100130872,RNF212,SLC26A1,SPON2,TMED11P,IDUA,CTBP1,FGFRL1,TMEM175,DGKQ	0	loss
2474	CGTE_32	4	3432335	3450074	4p16.3	HGFAC,RGS12	0	loss
2475	CGTE_32	4	26321380	26322732	4p15.2	RBPJ	12	gain
2476	CGTE_32	5	1268653	1272473	5p15.33	TERT	7	gain
2477	CGTE_32	5	54522286	54710104	5q11.2	SKIV2L2,MCIDAS,CCNO,DHX29	1	loss
2478	CGTE_32	5	140175794	140264088	5q31.3	PCDHA7,PCDHA8,PCDHA5,PCDHA12,PCDHA13,PCDHA3,PCDHA2,PCDHA9,PCDHA4,PCDHA10,PCDHA6,PCDHA1,PCDHA11	0	loss
2479	CGTE_32	6	30860711	30863284	6p21.33	DDR1	11	gain
2480	CGTE_32	6	32029097	32030067	6p21.33	TNXB	55	gain
2481	CGTE_32	6	33416529	33419732	6p21.32	SYNGAP1	11	gain
2482	CGTE_32	6	33641890	33656195	6p21.31	ITPR3	4	gain
2483	CGTE_32	6	37622059	37664608	6p21.2	MDGA1	4	gain
2484	CGTE_32	6	64288878	64390087	6q12	PTP4A1,PHF3	5	gain
2485	CGTE_32	6	133827157	134350968	6q23.2	SLC2A12,LINC01312,TARID,TBPL1,TCF21,EYA4	3	gain
2486	CGTE_32	6	134373515	134436545	6q23.2	HMGA1P7,SLC2A12	5	gain
2487	CGTE_32	7	75615415	75617780	7q11.23	POR,TMEM120A	10	gain
2488	CGTE_32	8	40010915	43160039	8p11.1-p11.21	POLB,C8orf4,KAT6A,MIR4469,GINS4,MIR486-2,HGSNAT,AP3M2,DKK4,LOC102723729,RNF170,POMK,ZMAT4,GOLGA7,POTEA,THAP1,NKX6-3,SLC20A2,CHRNA6,ANK1,CHRNB3,GPAT4,SFRP1,MIR486-1,SMIM19,LOC101929897,HOOK3,VDAC3,PLAT,FNTA,IKBKB	1	loss
2489	CGTE_32	8	144460882	145009356	8q24.3	MIR4664,NRBP2,PUF60,MIR937,FAM83H-AS1,MAPK15,EEF1D,EPPK1,SCRIB,ZNF623,GSDMD,MAFA,ZNF707,MIR6845,FAM83H,MAFA-AS1,ZC3H3,NAPRT,CCDC166,PLEC,MROH6,PYCRL,BREA2,TSTA3,TIGD5,RHPN1	0	loss
2490	CGTE_32	8	145009357	145012931	8q24.3	PLEC	5	gain
2491	CGTE_32	8	145013436	145138426	8q24.3	GRINA,PLEC,EXOSC4,MIR6847,PARP10,SPATC1,GPAA1,OPLAH,MIR661,MIR6846	0	loss
2492	CGTE_32	8	145138598	145140844	8q24.3	GPAA1	5	gain
2493	CGTE_32	9	79634862	79952484	9q21.2	FOXB2,VPS13A,VPS13A-AS1	0	loss
2494	CGTE_32	9	139390521	139409036	9q34.3	NOTCH1	0	loss
2495	CGTE_32	9	139409039	139414075	9q34.3	MIR4673,NOTCH1	4	gain
2496	CGTE_32	9	139417295	139568413	9q34.3	NOTCH1,AGPAT2,MIR4674,NALT1,MIR126,EGFL7	0	loss
2497	CGTE_32	9	139569120	139572046	9q34.3	AGPAT2	4	gain
2498	CGTE_32	9	139581622	139618097	9q34.3	AGPAT2,FAM69B	0	loss
2499	CGTE_32	9	139730048	139744026	9q34.3	C9orf172,RABL6,PHPT1	0	loss
2500	CGTE_32	9	139744469	139747079	9q34.3	MAMDC4,PHPT1	8	gain
2501	CGTE_32	9	139747560	139943727	9q34.3	C9orf142,LCNL1,FUT7,C9orf139,ENTPD2,NPDC1,MIR4479,C8G,FBXW5,MAMDC4,EDF1,LCN12,CLIC3,PTGDS,TRAF2,ABCA2	0	loss

2502	CGTE_32	9	139944327	139946874	9q34.3	ENTPD2	6	gain
2503	CGTE_32	9	139948165	139973116	9q34.3	SAPCD2,ENTPD2,UAP1L1	0	loss
2504	CGTE_32	9	140001694	140151481	9q34.3	SSNA1,FAM166A,C9orf173,TMEM210,CYSRT1,NELFB,TPRN,C9orf173-AS1,ANAPC2,NDOR1,TMEM203,RNF224,SLC34A3,GRIN1,MAN1B1,RNF208,LRRC26,DPP7,MIR3621,TUBB4B	0	loss
2505	CGTE_32	9	140157475	140160447	9q34.3	NELFB	8	gain
2506	CGTE_32	9	140160776	140389610	9q34.3	NOXA1,EXD3,TOR4A,NELFB,MIR7114,NSMF,NRARP,PNPLA7,ENTPD8	0	loss
2507	CGTE_32	9	140391507	140395360	9q34.3	PNPLA7	11	gain
2508	CGTE_32	10	94608232	94835040	10q23.33	CYP26C1,CYP26A1,EXOC6	0	loss
2509	CGTE_32	11	1025974	1080490	11p15.5	MUC6,MUC2	0	loss
2510	CGTE_32	11	1080494	1081152	11p15.5	MUC2	7	gain
2511	CGTE_32	11	17631172	17633837	11p15.1	OTOG	17	gain
2512	CGTE_32	12	20896185	21242958	12p12.2	SLCO1B7,SLCO1B3,SLCO1C1	1	loss
2513	CGTE_32	12	48185020	48190116	12q13.11	HDAC7	0	loss
2514	CGTE_32	12	57584558	57605616	12q13.3	LRP1,MIR1228	3	gain
2515	CGTE_32	12	110032746	110182148	12q24.11	FAM222A,FAM222A-AS1,MVK	8	gain
2516	CGTE_32	12	122212749	122217601	12q24.31	RHOF,TMEM120B	7	gain
2517	CGTE_32	12	124779984	124873314	12q24.31	ZNF664-FAM101A,NCOR2,FAM101A,MIR6880	0	loss
2518	CGTE_32	12	132326120	132394614	12q24.33	ULK1,MMP17	0	loss
2519	CGTE_32	12	132394741	132399028	12q24.33	ULK1	4	gain
2520	CGTE_32	12	132399441	132404156	12q24.33	ULK1	0	loss
2521	CGTE_32	14	104552082	105412546	14q32.33	MIR4710,C14orf180,AKT1,ADSSL1,TMEM179,CEP170B,ASPG,AHNAK2,SIVA1,MIR203A,INF2,LINC00638,ZBTB42,KIF26A,MIR203B,PLD4	0	loss
2522	CGTE_32	14	105412779	105418727	14q32.33	AHNAK2	5	gain
2523	CGTE_32	15	56243637	56999359	15q21.3	RFX7,MNS1,TEX9,NEDD4,ZNF280D	1	loss
2524	CGTE_32	16	97450	920066	16p13.3	NPRL3,RHOT2,PRR25,HBA1,FAM234A,PIGQ,GNG13,HBM,PDIA2,POLR3K,LINC00235,DECR2,FAM195A,RAB40C,CCDC78,LOC100134368,HAGHL,FBXL16,METRN,LMF1,MIR662,RHBDL1,HBQ1,PRR35,STUB1,SNRNP25,MSLN,FAM173A,JMJD8,MPG,ARHGDIG,HBA2,MIR3176,RGS11,NME4,MIR5587,RAB11FIP3,AXIN1,HBZ,RHBDF1,WDR90,WFIKKN1,NARFL,CAPN15,CHTF18,NHLRC4,WDR24,TMEM8A,MRPL28,C16orf13,RPUSD1,LUC7L	1	loss
2525	CGTE_32	16	920676	943180	16p13.3	LMF1	6	gain
2526	CGTE_32	16	943201	2097939	16p13.3	SNORA10,EME2,RPS2,MIR3177,MAPK8IP3,TPSB2,RPL3L,GNPTG,MEIOB,SLC9A3R2,TBL3,LINC00254,SPSB3,MSRB1,SSTR5-AS1,LMF1-AS1,UBE2I,HAGH,BAIAP3,TSR3,SNHG9,C1QTNF8,UNKL,NTHL1,TELO2,TPSG1,LMF1,SNORA78,SYNGR3,NUBP2,TMEM204,TPSD1,IGFALS,HS3ST6,RNF151,SNORA64,CCDC154,FAHD1,TPSAB1,SSTR5,NME3,HN1L,CRAMP1,SOX8,PTX4,NOXO1,ZNF598,MRPS34,NPW,CACNA1H,C16orf91,IFT140,GFER,CLCN7,NDUFB10	1	loss
2527	CGTE_32	17	79077744	79080034	17q25.3	BAIAP2	9	gain
2528	CGTE_32	17	79080500	79508104	17q25.3	SLC38A10,TMEM105,MIR657,MIR1250,C17orf89,ENTHD2,ACTG1,AATK,BAIAP2,MIR338,BAHCC1,CEP131,LINC00482,MIR3065,MIR4740,AATK-AS1,FSCN2,FAAP100,LOC100130370,MIR3186	0	loss
2529	CGTE_32	17	79508329	79512882	17q25.3	FAAP100	58	gain
2530	CGTE_32	17	80018432	80051264	17q25.3	DUS1L,SNORD134,FASN	0	loss
2531	CGTE_32	18	25756893	28574468	18q12.1	DSC3,CDH2,MIR302F	9	gain
2532	CGTE_32	18	55102921	55104114	18q21.31	ONECUT2	0	loss
2533	CGTE_32	18	66351606	66513765	18q22.1	TMX3,CCDC102B	0	loss
2534	CGTE_32	18	77160324	77170736	18q23	NFATC1	325	gain
2535	CGTE_32	18	77170739	77208984	18q23	NFATC1	5	gain

2536	CGTE_32	19	281411	1980402	19p13.3	POLRMT,SHC2,PCSK4,C2CD4C,CFD,ATP8B3,SCAMP4,ONECUT3,PTBP1,CIRBP-AS1,HMHA1,RNF126,RPS15,RNU6-2,TCF3,ODF3L2,UQCR11,ADAMTSL5,GAMT,MED16,MADCAM1,PALM,CSNK1G2,R3HDM4,MEX3D,REXO1,ELANE,MBD3,BSG,PLPPR3,MISP,CNN2,KISS1R,CIRBP,TPGS1,GRIN3B,C19orf24,HCN2,NDUFS7,CSNK1G2-AS1,CDC34,MIR4745,EFNA2,GPX4,PLK5,CBARP,MIDN,ABHD17A,KLF16,LOC100288123,STK11,ATP5D,FSTL3,WDR18,SBNO2,ADAT3,REEP6,GZMM,PLPP2,THEG,POLR2E,DAZAP1,AZU1,APC2,ABCA7,MUM1,MIR3187,TMEM259,PRTN3,C19orf25,MIR1909,FGF22,ARID3A,PRSS57,MIER2	0	loss
2537	CGTE_32	19	4212526	4217438	19p13.3	ANKRD24	5	gain
2538	CGTE_32	20	3211162	3214966	20p13	SLC4A11	8	gain
2539	CGTE_32	20	62050934	62078219	20q13.33	KCNQ2	4	gain
2540	CGTE_32	20	62103503	62222147	20q13.33	C20orf195,PPDPF,HELZ2,KCNQ2,PTK6,EEF1A2,SRMS,GMEB2	0	loss
2541	CGTE_32	21	45843428	46057457	21q22.3	KRTAP10-2,KRTAP10-10,LRRC3-AS1,TSPEAR-AS1,KRTAP10-4,KRTAP10-9,TSPEAR,KRTAP10-3,KRTAP10-1,KRTAP10-8,TRPM2,KRTAP10-6,TSPEAR-AS2,TRPM2-AS,KRTAP10-7,KRTAP10-5,LRRC3	1	loss
2542	CGTE_32	21	46057516	46074692	21q22.3	KRTAP10-11,TSPEAR,KRTAP12-4,KRTAP10-10	6	gain
2543	CGTE_32	22	50870426	50904405	22q13.33	SBF1,PPP6R2	0	loss
2544	CGTE_32	X	152864479	152937170	Xq28	PNCK,FAM58A,LOC105373383,DUSP9	6	gain
2545	CGTE_32	X	152937334	153040877	Xq28	SLC6A8,PLXNB3,PNCK,ABCD1,BCAP31	0	loss
2546	CGTE_32	X	153040982	153041924	Xq28	PLXNB3	24	gain
2547	CGTE_32	X	153577716	153592724	Xq28	FLNA	3	gain
2548	CGTE_33	1	16344220	16359049	1p36.13	HSPB7,CLCNKA	1	loss
2549	CGTE_33	1	22148651	22217218	1p36.12	LDLRAD2,HSPG2	1	loss
2550	CGTE_33	1	151254447	151263835	1q21.3	ZNF687	1	loss
2551	CGTE_33	1	156814179	156894812	1q23.1	PEAR1,LRRC71,NTRK1,INSRR	1	loss
2552	CGTE_33	1	228495764	228645579	1q42.13	MIR6742,HIST3H2A,TRIM11,TRIM17,OBSCN,HIST3H3	1	loss
2553	CGTE_33	2	17877577	17884643	2p24.2	SMC6	0	loss
2554	CGTE_33	2	32353447	32368518	2p22.3	SPAST	1	loss
2555	CGTE_33	2	42980402	43571392	2p21	OXER1,ZFP36L2,THADA,LINC01126,MTA3,HAAO,LOC102723854	3	gain
2556	CGTE_33	2	74641252	74649545	2p13.1	WDR54,C2orf81	0	loss
2557	CGTE_33	2	74779477	74783763	2p13.1	DOK1,LOXL3	0	loss
2558	CGTE_33	2	95538507	95715441	2q11.1	LOC442028,MAL,TEKT4	1	loss
2559	CGTE_33	2	128381736	128431411	2q14.3	GPR17,MYO7B,LIMS2	1	loss
2560	CGTE_33	2	170734973	170751876	2q31.1	UBR3	0	loss
2561	CGTE_33	2	201397638	201401012	2q33.1	SGOL2	0	loss
2562	CGTE_33	2	219883704	219897456	2q35	CFAP65	1	loss
2563	CGTE_33	2	232234610	232236298	2q37.1	ARMC9	0	loss
2564	CGTE_33	2	233200895	233410501	2q37.1	PRSS56,ECEL1P2,ECEL1,CHRND,CHRNG,ALPI,ALPPL2,ALPP,DIS3L2	1	loss
2565	CGTE_33	2	234728295	234731652	2q37.1	MROH2A	0	loss
2566	CGTE_33	3	46720622	46729876	3p21.31	ALS2CL	1	loss
2567	CGTE_33	3	52525405	52540102	3p21.1	NISCH,STAB1	1	loss
2568	CGTE_33	3	135969225	136136842	3q22.3	PCCB,STAG1	3	gain
2569	CGTE_33	4	8234277	8293237	4p16.1	SH3TC1,HTRA3	0	loss
2570	CGTE_33	5	175956083	175992764	5q35.2	CDHR2,RNF44	1	loss
2571	CGTE_33	6	26252156	26273561	6p22.2	HIST1H3G,HIST1H2BH,HIST1H2BI	1	loss
2572	CGTE_33	6	31935970	32061354	6p21.33	DXO,TNXA,C4B,CYP21A1P,CYP21A2,C4A,TNXB,STK19,C4B_2,SKIV2L	1	loss
2573	CGTE_33	6	33132621	33143435	6p21.32	COL11A2	1	loss
2574	CGTE_33	6	80749375	80750444	6q14.1	TTK	0	loss
2575	CGTE_33	7	208660	905701	7p22.3	LOC442497,HRAT92,DNAAF5,LOC101927000,PDGFA,FAM20C,WI2-2373I1.2,LOC101926963,PRKAR1B,SUN1	3	gain

2576	CGTE_33	7	2966242	2998181	7p22.2	CARD11	3	gain
2577	CGTE_33	7	99811465	99821325	7q22.1	STAG3,GATS,PVRIG	0	loss
2578	CGTE_33	7	149476529	149528378	7q36.1	SSPO	1	loss
2579	CGTE_33	7	150557486	150699239	7q36.1	NOS3,KCNH2,AOC1	1	loss
2580	CGTE_33	8	143396292	143624848	8q24.3	TSNARE1,ADGRB1	1	loss
2581	CGTE_33	8	144802384	145831031	8q24.3	FBXL6,MIR6849,MAPK15,HGH1,HSF1,PLEC,WDR97,NRBP2,TONSL-AS1,FAM83H-AS1,MIR937,MROH1,CYHR1,DGAT1,FOXH1,ARHGAP39,SCRT1,RECQL4,C8orf82,MIR6848,FAM83H,VPS28,EXOSC4,MIR7112,EPPK1,GPAA1,SCRIB,SPATC1,TMEM249,MIR4664,PARP10,LRRC24,GPT,SCX,MIR6893,SLC39A4,ADCK5,MIR6846,PUF60,MFSD3,MIR6845,TONSL,MAF1,PPP1R16A,GRINA,MIR6847,SHARPIN,SLC52A2,BOP1,KIFC2,CYC1,OPLAH,CPSF1,MIR939,LRRC14,MIR661	1	loss
2582	CGTE_33	9	117165396	117169191	9q32	DFNB31	0	loss
2583	CGTE_33	9	133944257	133961092	9q34.12	LAMC3	1	loss
2584	CGTE_33	10	33014200	33136894	10p11.22	CCDC7	1	loss
2585	CGTE_33	10	72195085	72542177	10q22.1	NODAL,TBATA,ADAMTS14,PRF1,PALD1	1	loss
2586	CGTE_33	10	73565497	73573207	10q22.1	CDH23	1	loss
2587	CGTE_33	10	75843086	75898150	10q22.2	VCL,AP3M1	4	gain
2588	CGTE_33	10	75984162	76468298	10q22.2	ADK,LOC102723439	4	gain
2589	CGTE_33	10	99258053	99337663	10q24.1-q24.2	ANKRD2,UBTD1,MMS19	0	loss
2590	CGTE_33	10	104158428	104164540	10q24.32	PSD,NFKB2	1	loss
2591	CGTE_33	10	105232981	105363697	10q24.33	CALHM3,NEURL1,SH3PXD2A,NEURL1-AS1	0	loss
2592	CGTE_33	10	112333439	112337260	10q25.2	SMC3	1	loss
2593	CGTE_33	10	112337564	112350898	10q25.2	SMC3	3	gain
2594	CGTE_33	11	62384726	62603010	11q12.3	BSCL2,MIR6748,GANAB,SNORA57,GNG3,UQCC3,ZBTB3,TAF6L,LRRN4CL,MIR6514,LBHD1,NXF1,POLR2G,METTL12,TMEM179B,TMEM223,TTC9C,STX5,HNRNPUL2-BSCL2,C11orf98,B3GAT3,INTS5,UBXN1,HNRNPUL2,WDR74	3	gain
2595	CGTE_33	11	67058826	67371994	11q13.2	GPR152,CABP4,GSTP1,SSH3,CORO1B,CARNS1,C11orf72,POLD4,TMEM134,TBC1D10C,RPS6KB2,CABP2,LOC100130987,RAD9A,AIP,MIR6752,PPP1CA,CLCF1,CDK2AP2,PITPNM1,ANKRD13D,PTPRCAP	1	loss
2596	CGTE_33	12	319002	330768	12p13.33	SLC6A13,SLC6A12	0	loss
2597	CGTE_33	12	40874156	40888021	12q12	MUC19	1	loss
2598	CGTE_33	12	48133942	48137975	12q13.11	RAPGEF3	1	loss
2599	CGTE_33	12	52282272	52285104	12q13.13	ANKRD33	1	loss
2600	CGTE_33	12	57556550	57626395	12q13.3	NXPH4,MIR1228,SHMT2,LRP1	1	loss
2601	CGTE_33	12	109998859	109999692	12q24.11	MMAB	0	loss
2602	CGTE_33	12	113835087	113909315	12q24.13	SDS,SDSL,LHX5	1	loss
2603	CGTE_33	13	46820594	46841128	13q14.13	LRRC63	0	loss
2604	CGTE_33	13	48938921	48953798	13q14.2	RB1	0	loss
2605	CGTE_33	13	53237221	53261896	13q14.3	SUGT1	1	loss
2606	CGTE_33	13	99092111	99093075	13q32.2	FARP1	1	loss
2607	CGTE_33	13	111155431	111160613	13q34	COL4A2,COL4A2-AS1	0	loss
2608	CGTE_33	14	67878442	69445837	14q24.1-q23.3	ARG2,ACTN1,RAD51B,PLEK2,PLEKHH1,TMEM229B,RDH11,PIGH,RDH12,ZFYVE26,VTI1B,ZFP36L1	3	gain
2609	CGTE_33	15	40630739	40656741	15q15.1	DISP2,PHGR1,C15orf52	1	loss
2610	CGTE_33	15	41148449	41508090	15q15.1	RHOV,CHAC1,EXD1,VPS18,INO80,DLL4,SPINT1	3	gain
2611	CGTE_33	15	42113991	42179584	15q15.1	MAPKBP1,MIR4310,JMJD7,JMJD7-PLA2G4B,PLA2G4B,SPTBN5	1	loss
2612	CGTE_33	15	74244031	74281138	15q24.1	LOXL1,STOML1	0	loss
2613	CGTE_33	15	75498193	75652645	15q24.2	COMMD4,C15orf39,NEIL1,MAN2C1,GOLGA6D,MIR631,GOLGA6C	1	loss
2614	CGTE_33	15	77322836	77329533	15q24.3	PSTPIP1	0	loss
2615	CGTE_33	16	67325235	67416070	16q22.1	LRRC36,KCTD19	3	gain

2616	CGTE_33	16	67913236	67986370	16q22.1	EDC4,LCAT,SLC12A4,PSKH1,NRN1L,CTRL,PSMB10	1	loss
2617	CGTE_33	16	68679202	69294288	16q22.1	CHTF8,TANGO6,SNTB2,CDH3,MIR7641-2,CDH1,HAS3,UTP4	3	gain
2618	CGTE_33	17	4348308	4446596	17p13.2	MYBBP1A,SPNS2,SPNS3	1	loss
2619	CGTE_33	17	42327763	42461200	17q21.31	FAM171A2,GRN,SLC25A39,RUNDC3A,SLC4A1,ITGA2B,RUNDC3A-AS1	1	loss
2620	CGTE_33	17	73485660	73844037	17q25.1	SAP30BP,ITGB4,SMIM6,WBP2,MIR4738,UNK,MYO15B,MIR6785,RECQL5,GALK1,H3F3B,CASKIN2,UNC13D,TSEN54,SMIM5,TMEM94,LLGL2	1	loss
2621	CGTE_33	17	79857662	80017938	17q25.3	STRA13,GPS1,LRRC45,NPB,PYCR1,ASPSCR1,DUS1L,PCYT2,RFNG,NOTUM,SIRT7,MAFG,MAFG-AS1,RAC3,DCXR,MYADML2,ANAPC11	1	loss
2622	CGTE_33	18	9239420	9256628	18p11.22	ANKRD12	1	loss
2623	CGTE_33	18	19120546	19146224	18q11.2	ESCO1	1	loss
2624	CGTE_33	18	34773515	34850847	18q12.2	CELF4,KIAA1328	3	gain
2625	CGTE_33	18	46145781	46163065	18q21.1	CTIF	0	loss
2626	CGTE_33	19	50882979	50940056	19q13.33	SPIB,MYBPC2,POLD1,NR1H2	1	loss
2627	CGTE_33	20	33572422	33592385	20q11.22	TRPC4AP,MIR499A,MIR499B,MYH7B	1	loss
2628	CGTE_33	20	44587750	44596689	20q13.12	ZNF335	0	loss
2629	CGTE_33	20	44985141	44987393	20q13.12	SLC35C2	0	loss
2630	CGTE_33	20	61441727	61459462	20q13.33	OGFR,COL9A3	0	loss
2631	CGTE_33	21	45708156	45745153	21q22.3	AIRE,PFKL	1	loss
2632	CGTE_33	22	21345974	21402382	22q11.21	LRRC74B,THAP7,P2RX6P,MIR649,THAP7-AS1,SLC7A4,LZTR1,TUBA3FP,P2RX6	1	loss
2633	CGTE_33	22	24981803	25007299	22q11.23	GGT1,LRRC75B	1	loss
2634	CGTE_33	22	36681763	36691205	22q12.3	MYH9,MIR6819	0	loss
2635	CGTE_33	22	38492981	38522549	22q13.1	PLA2G6,BAIAP2L2	1	loss
2636	CGTE_33	22	50685145	50723353	22q13.33	PLXNB2,HDAC10,MAPK11,MAPK12	1	loss
2637	CGTE_33	X	9881976	9905426	Xp22.2	SHROOM2	0	loss
2638	CGTE_33	X	37003718	37027779	Xp21.1	FAM47C	0	loss
2639	CGTE_33	X	53279319	53284129	Xp11.22	IQSEC2	0	loss
2640	CGTE_33	X	153578469	153599424	Xq28	FLNA	0	loss
2641	CGTE_33	Y	15435539	15466793	Yq11.221	UTY	0	loss
2642	CGTE_34	1	861266	866507	1p36.33	SAMD11	5	gain
2643	CGTE_34	1	871064	1564067	1p36.33	UBE2J2,ATAD3A,MIR6726,ISG15,RNF223,VWA1,SDF4,LOC102724312,MIB2,MXRA8,C1orf233,TAS1R3,PUSL1,FAM132A,LINC01342,ACAP3,MIR6808,CPTP,PERM1,ATAD3B,MRPL20,TNFRSF4,ATAD3C,AURKAIP1,LOC148413,CPSF3L,NOC2L,SAMD11,TTLL10,C1orf159,MIR200B,PLEKHN1,TMEM88B,DVL1,KLHL17,SCNN1D,B3GALT6,HES4,TMEM240,TNFRSF18,MIR200A,CCNL2,MIR429,AGRN,MIR6727,SSU72,ANKRD65	0	loss
2644	CGTE_34	1	1895183	3650151	1p36.32-p36.33	GABRD,MIR4251,TPRG1L,ACTRT2,TTC34,ARHGEF16,LOC100129534,LINC00982,PANK4,MMEL1,PRDM16,LOC100996583,TP73,MEGF6,FAM213B,PRKCZ,TNFRSF14,HES5,LOC115110,FAAP20,PLCH2,RER1,SKI,WRAP73,CFAP74,MIR551A,PEX10,MORN1	1	loss
2645	CGTE_34	1	17248472	17328872	1p36.13	CROCC,ATP13A2,MFAP2	0	loss
2646	CGTE_34	1	228333228	228468459	1q42.13	GUK1,IBA57-AS1,IBA57,GJC2,OBSCN,C1orf145	0	loss
2647	CGTE_34	1	228469459	228471439	1q42.13	OBSCN	8	gain
2648	CGTE_34	1	228524589	228526140	1q42.13	OBSCN	6	gain
2649	CGTE_34	1	228526548	228597061	1q42.13	MIR6742,TRIM11,OBSCN,TRIM17	1	loss
2650	CGTE_34	1	228598667	228645168	1q42.13	HIST3H2A,HIST3H3,TRIM17	4	gain
2651	CGTE_34	2	170734973	170751876	2q31.1	UBR3	16	gain
2652	CGTE_34	2	220347788	220355707	2q35	SPEG	11	gain
2653	CGTE_34	2	233243426	233321475	2q37.1	ALPPL2,ALPP,ALPI,ECEL1P2	6	gain
2654	CGTE_34	2	239006590	239059847	2q37.3	SCLY,UBE2F-SCLY,KLHL30,ESPNL	0	loss
2655	CGTE_34	2	241508080	241627403	2q37.3	GPR35,CAPN10-AS1,CAPN10,AQP12B,RNPEPL1	0	loss

2656	CGTE_34	2	241816287	241932646	2q37.3	AGXT,LOC200772,C2orf54	0	loss
2657	CGTE_34	3	52553409	52558572	3p21.1	NT5DC2,STAB1	5	gain
2658	CGTE_34	3	57846400	57851195	3p14.3	SLMAP	6	gain
2659	CGTE_34	3	148777122	148778694	3q24	HLTF	10	gain
2660	CGTE_34	3	196669188	196674753	3q29	NCBP2-AS2,PIGZ,NCBP2	22	gain
2661	CGTE_34	5	42762552	42779753	5p12	CCDC152	7	gain
2662	CGTE_34	5	140188016	140257143	5q31.3	PCDHA12,PCDHA8,PCDHA5,PCDHA10,PCDHA1,PCDHA4,PCDHA6,PCDHA11,PCDHA3,PCDHA9,PCDHA7,PCDHA2	0	loss
2663	CGTE_34	5	172386735	172421971	5q35.1	ATP6V0E1,RPL26L1	5	gain
2664	CGTE_34	5	176860624	176936797	5q35.3	PRR7-AS1,DOK3,GRK6,DBN1,PDLIM7,PRR7	0	loss
2665	CGTE_34	6	30862283	30863284	6p21.33	DDR1	43	gain
2666	CGTE_34	6	33416529	33419732	6p21.32	SYNGAP1	30	gain
2667	CGTE_34	6	33650996	33653610	6p21.31	ITPR3	25	gain
2668	CGTE_34	6	41552454	41652981	6p21.1	MIR4641,MDFI,FOXP4,TFEB	0	loss
2669	CGTE_34	6	80749375	80750444	6q14.1	TTK	12	gain
2670	CGTE_34	6	126298763	126319529	6q22.32	TRMT11,HINT3	7	gain
2671	CGTE_34	6	128840188	129204607	6q22.33	LAMA2,PTPRK	7	gain
2672	CGTE_34	6	134373515	134436545	6q23.2	SLC2A12,HMGA1P7	5	gain
2673	CGTE_34	7	149493398	149497153	7q36.1	SSPO	4	gain
2674	CGTE_34	7	149497396	149521882	7q36.1	SSPO	0	loss
2675	CGTE_34	7	149521925	149523373	7q36.1	SSPO	12	gain
2676	CGTE_34	8	21977237	21997080	8p21.3	REEP4,HR	0	loss
2677	CGTE_34	8	143605476	143763559	8q24.3	ADGRB1,PSCA,ARC,JRK	0	loss
2678	CGTE_34	8	143763564	143832748	8q24.3	PSCA,SLURP1,THEM6,LOC100288181,LYPD2,LY6K	4	gain
2679	CGTE_34	8	143833743	143867988	8q24.3	LYNX1,LYPD2,LY6D	0	loss
2680	CGTE_34	8	144240163	145009356	8q24.3	CCDC166,MROH6,MAFA-AS1,MINCR,RHPN1-AS1,NAPRT,ZC3H3,ZNF696,MIR6845,TIGD5,EEF1D,ZNF623,TSTA3,BREA2,PYCRL,GLI4,MAPK15,FAM83H,MAFA,TOP1MT,ZFP41,ZNF707,EPPK1,MIR937,PLEC,LY6H,GSDMD,SCRIB,GPIHBP1,MIR4664,NRBP2,PUF60,RHPN1,FAM83H-AS1	0	loss
2681	CGTE_34	8	145009357	145012485	8q24.3	PLEC	8	gain
2682	CGTE_34	8	145012533	145138426	8q24.3	MIR6847,SPATC1,PARP10,PLEC,GPAA1,EXOSC4,MIR661,OPLAH,MIR6846,GRINA	0	loss
2683	CGTE_34	8	145138598	145140502	8q24.3	GPAA1	4	gain
2684	CGTE_34	9	139378808	139568413	9q34.3	EGFL7,C9orf163,MIR4673,MIR4674,NOTCH1,NALT1,AGPAT2,MIR126	0	loss
2685	CGTE_34	9	139730048	139744657	9q34.3	RABL6,C9orf172,PHPT1	0	loss
2686	CGTE_34	9	139744851	139747079	9q34.3	PHPT1,MAMDC4	5	gain
2687	CGTE_34	9	139747560	139976585	9q34.3	SAPCD2,FBXW5,FUT7,ENTPD2,C8G,EDF1,LCNL1,C9orf142,CLIC3,ABCA2,MIR4479,PTGDS,TRAF2,NPDC1,C9orf139,UAP1L1,LCN12,MAMDC4	0	loss
2688	CGTE_34	9	140001694	140389610	9q34.3	C9orf173,PNPLA7,CYSRT1,LRRC26,TMEM203,RNF208,FAM166A,MAN1B1,NRARP,TPRN,ANAPC2,TOR4A,SLC34A3,NOXA1,NELFB,C9orf173-AS1,TMEM210,ENTPD8,RNF224,GRIN1,EXD3,TUBB4B,DPP7,NDOR1,MIR3621,MIR7114,NSMF,SSNA1	0	loss
2689	CGTE_34	9	140391507	140392723	9q34.3	PNPLA7	26	gain
2690	CGTE_34	11	67184774	67821587	11q13.2	NDUFS8,CORO1B,CARNS1,MIR6753,C11orf72,FAM86C2P,TMEM134,CHKA,PITPNM1,ALDH3B1,GSTP1,PTPRCAP,NUDT8,ACY3,GPR152,UNC93B1,CABP4,MIR4691,TCIRG1,AIP,RPS6KB2,NDUFV1,ALDH3B2,TBX10,MIR6752,DOC2GP,MIR7113,CDK2AP2,CABP2	0	loss
2691	CGTE_34	12	40876490	40885045	12q12	MUC19	4	gain
2692	CGTE_34	12	124779984	124915378	12q24.31	ZNF664-FAM101A,NCOR2,MIR6880,FAM101A	0	loss
2693	CGTE_34	13	21170268	21200006	13q12.11	IFT88	4	gain

2694	CGTE_34	13	25404580	25418092	13q12.12	RNF17	12	gain
2695	CGTE_34	13	96684081	96743252	13q32.1	HS6ST3,UGGT2	0	loss
2696	CGTE_34	14	103576202	103596472	14q32.32	EXOC3L4,TNFAIP2	4	gain
2697	CGTE_34	14	104618116	105363146	14q32.33	INF2,AKT1,LINC00638,SIVA1,ZBTB42,TMEM179,ADSSL1,KIF26A,MIR4710,CEP170B,C14orf180	0	loss
2698	CGTE_34	14	105393282	105412546	14q32.33	PLD4,AHNAK2	1	loss
2699	CGTE_34	14	105412779	105418727	14q32.33	AHNAK2	4	gain
2700	CGTE_34	15	42139373	42164151	15q15.1	MIR4310,SPTBN5,PLA2G4B,JMJD7-PLA2G4B	0	loss
2701	CGTE_34	16	97450	2284104	16p13.3	TPSB2,CACNA1H,TSR3,E4F1,GFER,TPSAB1,NPRL3,RAB26,AXIN1,HBQ1,CCDC78,CAPN15,SOX8,MPG,WDR90,LMF1-AS1,FAM173A,NHLRC4,ARHGDIG,BRICD5,NTHL1,HN1L,TPSD1,MIR3177,SNORA10,RPS2,PTX4,GNG13,RPUSD1,SNRNP25,SLC9A3R2,MIR6511B1,MSRB1,PKD1,FAHD1,SYNGR3,JMJD8,LUC7L,MIR6511B2,MRPL28,RAB11FIP3,HBA2,UBE2I,TPSG1,RAB40C,PRR35,SNHG9,UNKL,RHOT2,PDIA2,HBM,WFIKKN1,FBXL16,MIR662,HAGH,LINC00235,HBA1,IGFALS,MIR5587,NME4,CRAMP1,RHBDF1,FAM195A,C16orf13,TSC2,SNORD60,MRPS34,SSTR5-AS1,HAGHL,ZNF598,C16orf91,HBZ,CLCN7,SNHG19,PGP,EME2,SPSB3,TRAF7,MEIOB,MIR3176,MIR3180-5,RHBDL1,HS3ST6,NDUFB10,TMEM8A,C1QTNF8,FAM234A,CHTF18,NPW,LOC100134368,TELO2,NUBP2,MIR1225,GNPTG,PIGQ,MLST8,RNF151,RPL3L,TMEM204,METRN,POLR3K,SNORA64,RGS11,IFT140,MSLN,STUB1,MIR4516,BAIAP3,TBL3,CASKIN1,WDR24,SSTR5,NARFL,MAPK8IP3,SNORA78,PRR25,CCDC154,NME3,LINC00254,NOXO1,DECR2,LMF1	1	loss
2702	CGTE_34	17	80038173	80051264	17q25.3	SNORD134,FASN	0	loss
2703	CGTE_34	17	80051469	80065616	17q25.3	FASN,CCDC57	4	gain
2704	CGTE_34	18	9239420	9256307	18p11.22	ANKRD12	5	gain
2705	CGTE_34	18	9256378	9279684	18p11.22	ANKRD12	3	gain
2706	CGTE_34	19	281411	1622622	19p13.3	DAZAP1,C2CD4C,ATP5D,HCN2,STK11,C19orf24,SBNO2,AZU1,PRSS57,BSG,RNF126,NDUFS7,MIDN,GAMT,REEP6,RPS15,PALM,PLPPR3,GPX4,GRIN3B,SHC2,UQCR11,MUM1,ELANE,CNN2,THEG,PLK5,MISP,MIR3187,ABCA7,PLPP2,PTBP1,WDR18,CIRBP,POLR2E,PRTN3,TMEM259,FSTL3,MADCAM1,EFNA2,CBARP,MED16,RNU6-2,KISS1R,ADAMTSL5,APC2,MBD3,C19orf25,CIRBP-AS1,CDC34,FGF22,ODF3L2,TCF3,GZMM,CFD,MIR4745,POLRMT,HMHA1,R3HDM4,MIER2,ARID3A,MEX3D,TPGS1,PCSK4	0	loss
2707	CGTE_34	19	19905849	22785695	19p13.11-p12	ZNF90,ZNF486,ZNF93,ZNF708,ZNF43,ZNF626,ZNF738,ZNF737,ZNF257,GOLGA2P9,ZNF506,ZNF429,LINC01233,ZNF714,ZNF98,MIR1270,ZNF493,ZNF208,ZNF431,ZNF85,LOC101929124,ZNF729,ZNF826P,LOC641367,ZNF253,ZNF100,ZNF682,ZNF430,ZNF676,LINC00664	3	gain
2708	CGTE_34	20	60891582	60937710	20q13.33	LAMA5,LAMA5-AS1,MIR4758	0	loss
2709	CGTE_34	21	46057516	46074404	21q22.3	KRTAP10-11,KRTAP10-10,TSPEAR,KRTAP12-4	6	gain
2710	CGTE_34	21	47532625	47536771	21q22.3	COL6A2	7	gain
2711	CGTE_34	21	47537235	47539894	21q22.3	COL6A2	0	loss
2712	CGTE_34	22	50609165	50904830	22q13.33	MAPK12,SBF1,HDAC10,SELO,TUBGCP6,DENND6B,PANX2,MAPK11,PLXNB2,TRABD,PPP6R2	0	loss
2713	CGTE_34	X	53253865	53264360	Xp11.22	KDM5C,IQSEC2	0	loss
2714	CGTE_34	X	83402159	83442937	Xq21.1	RPS6KA6	4	gain
2715	CGTE_34	X	105921317	105970749	Xq22.3	RNF128,CXorf57	10	gain
2716	CGTE_34	X	152935817	152991671	Xq28	BCAP31,ABCD1,SLC6A8,PNCK	0	loss
2717	CGTE_34	X	153005560	153050632	Xq28	SRPK3,ABCD1,PLXNB3	0	loss
2718	CGTE_34	X	153648238	153649079	Xq28	TAZ	5	gain
2719	CGTE_34	X	153692811	153697115	Xq28	PLXNA3	0	loss

2720	CGTE_35	1	115456586	115524130	1p13.2	SYCP1	4	gain
2721	CGTE_35	1	150684156	150687224	1q21.3	HORMAD1	4	gain
2722	CGTE_35	1	247051364	247054451	1q44	AHCTF1	3	gain
2723	CGTE_35	2	233386764	233387878	2q37.1	PRSS56	4	gain
2724	CGTE_35	3	9988031	9989767	3p25.3	PRRT3,PRRT3-AS1	3	gain
2725	CGTE_35	3	33633855	33638305	3p22.3	CLASP2	3	gain
2726	CGTE_35	3	97499430	97583230	3q11.2	ARL6,CRYBG3	3	gain
2727	CGTE_35	3	146234673	146239452	3q24	PLSCR1	3	gain
2728	CGTE_35	5	89910020	89915035	5q14.3	ADGRV1	3	gain
2729	CGTE_35	7	5569184	5633203	7p22.1	ACTB,FSCN1	0	loss
2730	CGTE_35	7	73038481	73084437	7q11.23	MLXIPL,VPS37D	3	gain
2731	CGTE_35	7	89887301	89894630	7q21.13	CFAP69	4	gain
2732	CGTE_35	7	150783817	150784315	7q36.1	AGAP3	5	gain
2733	CGTE_35	8	16927165	16976333	8p22	MICU3	3	gain
2734	CGTE_35	8	125515964	125516798	8q24.13	TATDN1	4	gain
2735	CGTE_35	9	72920176	72933714	9q21.12	SMC5	3	gain
2736	CGTE_35	9	139333168	139333910	9q34.3	INPP5E	0	loss
2737	CGTE_35	10	32742222	32807010	10p11.22	CCDC7	3	gain
2738	CGTE_35	10	117185682	117228911	10q25.3	ATRNL1	3	gain
2739	CGTE_35	11	614801	617770	11p15.5	IRF7,CDHR5	4	gain
2740	CGTE_35	11	9438507	9442181	11p15.4	IPO7	3	gain
2741	CGTE_35	11	17742805	17757750	11p15.1	MYOD1,KCNC1	4	gain
2742	CGTE_35	13	53419777	53421740	13q14.3	PCDH8	0	loss
2743	CGTE_35	13	78235542	78272705	13q22.3	LOC100129307,SLAIN1,MIR3665	4	gain
2744	CGTE_35	14	50100386	50110441	14q21.3	POLE2,DNAAF2	0	loss
2745	CGTE_35	14	50120701	50121603	14q21.3	POLE2	4	gain
2746	CGTE_35	15	49833877	49867340	15q21.2	FAM227B	3	gain
2747	CGTE_35	17	6545443	6546407	17p13.1	TXNDC17	3	gain
2748	CGTE_35	17	6547736	6555205	17p13.1	C17orf100,TXNDC17,MED31	1	loss
2749	CGTE_35	17	79095094	79096622	17q25.3	AATK	23	gain
2750	CGTE_35	18	19751067	19757114	18q11.2	GATA6	0	loss
2751	CGTE_35	19	14073337	14074669	19p13.12	RFX1	4	gain
2752	CGTE_35	19	29567062	30314665	19q12	C19orf12,LOC284395,UQCRFS1,CCNE1,PLEKHF1,POP4,VSTM2B	3	gain
2753	CGTE_35	19	56125290	56128296	19q13.42	ZNF865	0	loss
2754	CGTE_35	20	58452978	58461095	20q13.33	SYCP2	3	gain
2755	CGTE_35	21	10793919	10907034	21p11.1-p11.2	TPTE	1	loss
2756	CGTE_35	21	10908781	10910238	21p11.1	TPTE	3	gain
2757	CGTE_35	21	38468894	38480696	21q22.13	TTC3	4	gain
2758	CGTE_35	22	20130396	20136478	22q11.21	CCDC188,ZDHHC8	0	loss
2759	CGTE_35	22	20784758	20791856	22q11.21	SCARF2	3	gain
2760	CGTE_35	22	30821666	30822063	22q12.2	MTFP1	4	gain

2761	CGTE_36	1	865582	6676969	1p36.31-p36.33	PLEKHG5,UBE2J2,B3GALT6,ANKRD65,CHD5,PERM1,TTLL10,WRAP73,PRKCZ,VWA1,HES4,CALML6,GPR153,CDK11A,ATAD3B,GNB1,NOL9,FAM213B,LINC01134,MIR4417,KLHL17,MEGF6,GABRD,PUSL1,ATAD3A,TMEM240,CFAP74,TNFRSF25,TTC34,SLC35E2B,TMEM88B,TMEM52,LOC102724450,MIR200B,ISG15,MIR4689,NPHP4,AJAP1,TAS1R1,MIR200A,MIR6808,MIR4252,MIR6726,LOC284661,KLHL21,LINC00982,SLC35E2,ZBTB48,CEP104,SKI,LOC100996583,RPL22,MIR4251,LOC148413,LOC115110,RNF223,MIB2,HES2,CDK11B,ESPN,SMIM1,LRRC47,LINC01342,RER1,PHF13,FAM132A,LOC100129534,KCNAB2,CPTP,ACAP3,MIR429,FAAP20,MRPL20,PLEKHN1,SCNN1D,MXRA8,PLCH2,C1orf159,C1orf174,RNF207,CCNL2,DVL1,MMEL1,SSU72,DFFB,C1orf233,MMP23B,LOC102724312,HES5,SAMD11,AURKAIP1,CCDC27,TP73-AS1,CPSF3L,MIR6727,AGRN,LINC01346,SDF4,ICMT,TNFRSF18,TAS1R3,ACOT7,MIR551A,TNFRSF14,ARHGEF16,MORN1,PRDM16,LINC00337,TNFRSF4,ATAD3C,MMP23A,TPRG1L,PANK4,ACTRT2,PEX10,HES3,NOC2L,NADK,TP73	1	loss
2762	CGTE_36	1	33944807	34678006	1p35.1-p34.3	C1orf94,ZSCAN20,CSMD2-AS1,CSMD2,HMGB4	3	gain
2763	CGTE_36	1	101343053	108247699	1p13.3-p21.2	DPH5,SLC30A7,AMY2B,LOC100129138,LOC101928370,EXTL2,NTNG1,AMY2A,PRMT6,RNPC3,LOC101928476,LOC102606465,AMY1A,ACTG1P4,OLFM3,AMY1C,COL11A1,LOC101928436,DNAJA1P5,VAV3,AMY1B,LINC01307,S1PR1	3	gain
2764	CGTE_36	1	152382018	153122682	1q21.3	LCE2D,IVL,LCE2B,LCE1C,LCE6A,SPRR1B,LCE2C,LCE3A,LCE1B,LCE1D,C1orf68,LCE3C,SPRR2A,LCE4A,CRNN,SPRR2B,SPRR2C,SMCP,LCE3E,SPRR2F,LCE5A,SPRR2D,SPRR2G,LCE3B,KPRP,SPRR1A,SPRR3,LCE1F,CRCT1,SPRR2E,LCE3D,LCE1A,SPRR4,LCE2A,LCE1E	3	gain
2765	CGTE_36	1	158368314	158817855	1q23.1	OR6K3,OR6K2,OR10X1,OR10R2,OR10Z1,MNDA,OR6Y1,OR10K1,SPTA1,OR6N1,OR6K6,OR10K2,OR6N2,OR10T2,OR6P1	3	gain
2766	CGTE_36	1	185267183	200183405	1q31.1-q31.2	LINC01031,B3GALT2,TROVE2,DENND1B,RGS13,LINC01036,LHX9,HMCN1,MIR4735,TPR,PDC,PTGS2,CRB1,ASPM,PLA2G4A,MIR548F1,MIR181A1,RGS18,LINC01037,IVNS1ABP,ZBTB41,CFHR5,F13B,NR5A2,CFHR3,NEK7,C1orf53,MIR181B1,PTPRC,UCHL5,LINC01222,CFHR1,LINC01351,C1orf27,LOC440704,ATP6V1G3,CDC73,GS1-279B7.1,KCNT2,PRG4,LINC01032,MIR1278,OCLM,GLRX2,BRINP3,CFHR4,MIR4426,LOC102724919,PACERR,CFHR2,RGS2,LINC01350,LINC01221,RGS21,CFH,MIR181A1HG,RGS1	3	gain
2767	CGTE_36	1	214828613	219385063	1q41	LYPLAL1,KCTD3,SPATA17-AS1,LINC00210,MIR548F3,TGFB2-AS1,GPATCH2,ESRRG,LOC101929631,KCNK2,CENPF,LOC102723833,USH2A,RRP15,LYPLAL1-AS1,TGFB2,TGFB2-OT1,SPATA17	3	gain
2768	CGTE_36	1	227181987	227843289	1q42.13	ZNF678,CDC42BPA	3	gain
2769	CGTE_36	1	227843445	228649876	1q42.13	MIR4666A,ZNF678,SNAP47,MIR3620,IBA57-AS1,TRIM11,JMJD4,TRIM17,MIR6742,ZNF847P,WNT3A,GJC2,HIST3H2BB,PRSS38,C1orf35,MIR5008,IBA57,GUK1,MRPL55,HIST3H2A,C1orf145,OBSCN,LOC100130093,WNT9A,HIST3H3,ARF1	1	loss
2770	CGTE_36	2	136960251	149247707	2q23.1-q22.1	THSD7B,ZEB2-AS1,LOC101928273,MIR7157,ACVR2A,ORC4,LINC01412,SPOPL,HNMT,KYNU,ZEB2,ARHGAP15,PABPC1P2,GTDC1,MBD5,LOC101928386,LRP1B,YY1P2,NXPH2,TEX41	3	gain

2771	CGTE_36	2	160980216	170131769	2q24.2-q31.1	LOC101929532,COBLL1,RBMS1,GRB14,SLC38A11,FIGN,KCNH7,MIR4774,SPC25,AHCTF1P1,FAP,STK39,XIRP2,TBR1,GALNT3,LOC100996579,LOC101929570,DPP4,GCG,TTC21B,SCN1A,G6PC2,CERS6-AS1,ABCB11,CERS6,LRP2,SCN9A,IFIH1,LOC100505984,NOSTRIN,SCN2A,LOC105616981,LOC100506124,TANK,LOC102724058,MIR4785,SCN7A,ITGB6,CSRNP3,LOC101929512,LOC101929633,B3GALT1,SCN3A,LOC101929680,PSMD14,SNORA70F,SLC4A10,TTC21B-AS1,DHRS9,XIRP2-AS1,GCA	3	gain
2772	CGTE_36	2	179480026	190334221	2q32.1-q32.2	ZSWIM2,SCHLAP1,TFPI,NUP35,GULP1,LINC01473,FRZB,CWC22,CERKL,WDR75,TTN-AS1,DIRC1,MIR1245B,NEUROD1,ITGAV,LOC101927055,TTN,CALCRL,SSFA2,ZNF804A,FSIP2,SESTD1,ZC3H15,MIR561,DUSP19,PDE1A,UBE2E3,ZNF385B,MIR4437,FAM171B,PPP1R1C,MIR548N,DNAJC10,ITGA4,COL3A1,COL5A2,MIR1258,LOC101927156,CCDC141,MIR548AE1,MIR3606,MIR3129,NCKAP1,LOC101927196,MIR1245A,LINC01090	3	gain
2773	CGTE_36	2	209219891	216974384	2q34-q35	LOC100130451,CPS1,LINC00607,LOC101928103,MIR4438,MYL1,ATIC,RPE,TMEM169,LANCL1,BARD1,ABCA12,LOC102724849,KANSL1L,UNC80,MIR548F2,MREG,LOC101928020,MIR4776-2,PECR,VWC2L-IT1,LINC01614,SPAG16,IKZF2,PTH2R,LANCL1-AS1,MIR4776-1,VWC2L,ACADL,LOC102725079,FN1,PIKFYVE,ERBB4,XRCC5,CPS1-IT1,MAP2	3	gain
2774	CGTE_36	2	220307632	220771476	2q35	LOC100996693,SPEG,OBSL1,MIR3132,MIR4268,INHA,ASIC4,GMPPA,TMEM198,SLC4A3,STK11IP,CHPF	1	loss
2775	CGTE_36	2	233164682	233413598	2q37.1	ALPI,ALPPL2,ECEL1P2,CHRND,ALPP,CHRNG,DIS3L2,TIGD1,ECEL1,PRSS56	1	loss
2776	CGTE_36	2	238934000	242842580	2q37.3	STK25,RTP5,SEPT2,AQP12B,ILKAP,MIR4441,PRR21,MIR2467,PASK,MYEOV2,GAL3ST2,FLJ43879,BOK-AS1,PDCD1,HES6,TWIST2,NDUFA10,ING5,LINC01107,PP14571,KLHL30,MIR4786,LOC643387,MIR3133,THAP4,LOC200772,MIR4440,LOC150935,LINC01237,RNPEPL1,OR6B3,DTYMK,KIF1A,AGXT,MIR149,SNED1,GPR35,LOC151174,CAPN10-AS1,FAM132B,BOK,ANO7,UBE2F,C2orf54,NEU4,HDLBP,MIR4269,CAPN10,OTOS,ASB1,ESPNL,SCLY,D2HGDH,MGC16025,PER2,PPP1R7,DUSP28,AQP12A,OR6B2,FARP2,GPC1,ATG4B,UBE2F-SCLY,HDAC4,ANKMY1,MTERF4,TRAF3IP1	1	loss
2777	CGTE_36	3	33906672	36547347	3p22.3-p22.2	ARPP21,LOC101928135,PDCD6IP,MIR128-2,STAC	3	gain
2778	CGTE_36	3	46712440	47050219	3p21.31	MYL3,PRSS42,TMIE,PRSS45,NBEAL2,PRSS46,PRSS50,CCDC12,PTH1R,ALS2CL	1	loss
2779	CGTE_36	3	51975786	52930157	3p21.2-p21.1	POC1A,DNAH1,ITIH4-AS1,BAP1,SMIM4,ACY1,STAB1,SNORD19,NT5DC2,TMEM110-MUSTN1,PPM1M,GLYCTK,SNORD69,RPL29,PARP3,TNNC1,SEMA3G,GLYCTK-AS1,SNORD19B,NEK4,GNL3,PHF7,TLR9,PCBP4,DUSP7,TWF2,LINC00696,ITIH3,SNORD136,NISCH,GPR62,TMEM110,PBRM1,ALAS1,GLT8D1,ITIH1,ABHD14B,MIR8064,ABHD14A-ACY1,SPCS1,ITIH4,MIR135A1,RRP9,MIRLET7G,ABHD14A,WDR82,MUSTN1	1	loss
2780	CGTE_36	3	77147119	93722803	3p11.2-p12.2	CGGBP1,MIR4795,LOC101927374,ROBO2,CHMP2B,C3orf38,LINC00506,ARL13B,GBE1,ROBO1,LINC00971,CADM2,MIR3923,PROS1,CADM2-AS2,ZNF654,SNORA95,HTR1F,VGLL3,EPHA3,POU1F1,MIR5688,LOC728290	3	gain
2781	CGTE_36	3	113775201	118866432	3q13.32-q13.31	LSAMP,IGSF11,ZBTB20,IGSF11-AS1,TIGIT,DRD3,LSAMP-AS1,MIR4447,LINC00901,ZNF80,LOC101929754,ZBTB20-AS4,ZBTB20-AS3,MIR4796,MIR568,QTRTD1,TUSC7,ZBTB20-AS1,GAP43,C3orf30,CCDC191	3	gain
2782	CGTE_36	3	123983395	124210456	3q21.2	MIR6083,KALRN	3	gain
2783	CGTE_36	3	151105542	151129263	3q25.1	MED12L	5	gain
2784	CGTE_36	3	161220793	165555309	3q26.1	LINC01324,MIR1263,SLITRK3,BCHE,LINC01322,LINC01192,OTOL1,SI	4	gain

2785	CGTE_36	3	186978491	192973631	3q29-q28	MGC2889,LPP,MIR28,TP63,LINCR-0002,MB21D2,MASP1,TPRG1-AS2,CCDC50,TPRG1-AS1,IL1RAP,FLJ42393,OSTN-AS1,BCL6,LPP-AS2,LOC100131635,UTS2B,GMNC,SST,OSTN,SNAR-I,CLDN1,P3H2,RTP4,TPRG1,FGF12,LPP-AS1,FGF12-AS1,MIR944,PYDC2,HRASLS,TMEM207,RTP2,CLDN16,P3H2-AS1	3	gain
2786	CGTE_36	4	437910	1659301	4p16.3	SPON2,SLC26A1,PDE6B,LOC100130872,ABCA11P,TMED11P,PIGG,MYL5,MFSD7,RNF212,TMEM175,LOC100129917,FGFRL1,ZNF721,CRIPAK,NKX1-1,CTBP1-AS2,DGKQ,MAEA,CTBP1-AS,GAK,PCGF3,CTBP1,CPLX1,UVSSA,FAM53A,ATP5I,IDUA	1	loss
2787	CGTE_36	4	17835931	24578314	4p15.31-p15.2	SLIT2,LOC100505912,MIR548AJ2,PACRGL,LCORL,GBA3,SLIT2-IT1,MIR218-1,DHX15,MIR573,KCNIP4-IT1,ADGRA3,MIR7978,PPARGC1A,NCAPG,KCNIP4	3	gain
2788	CGTE_36	4	26417126	36296676	4p14-p15.2	LOC102723778,PCDH7,LOC102723828,TBC1D19,LOC439933,LOC101929199,STIM2,CCKAR,DTHD1,ARAP2,RBPJ,LOC101928622,MIR4275	3	gain
2789	CGTE_36	4	62362908	76439480	4q21.1-q13.1	PARM1,NPFFR2,CXCL1,HTN1,HTN3,LOC550113,PF4V1,PF4,UGT2A2,CSN2,UGT2B10,SULT1B1,DCK,RCHY1,TECRL,AFP,STAP1,EREG,UGT2A3,TMPRSS11BNL,LOC100507388,MUC7,AFM,ANKRD17,PROL1,AMTN,MOB1B,CSN1S2AP,PPBPP2,ENAM,RASSF6,TMPRSS11F,UBA6,ALB,CSN3,CSN1S1,CXCL8,AMBN,LOC728040,ADAMTS3,UGT2B15,ADGRL3-AS1,UGT2A1,ODAM,BTC,STATH,CXCL5,LOC401134,UGT2B28,ADGRL3,EPGN,EPHA5-AS1,COX18,RUFY3,FTLP10,SULT1E1,AREG,PPBP,UBA6-AS1,EPHA5,CXCL6,GRSF1,UGT2B4,SMR3A,MIR1269A,CENPC,SLC4A4,UTP3,CXCL3,UGT2B7,CABS1,TMPRSS11A,CSN1S2BP,PRR27,TMPRSS11B,FDCSP,JCHAIN,MTHFD2L,LOC441025,TMPRSS11D,UGT2B17,SYT14P1,UGT2B11,YTHDC1,TMPRSS11E,TMPRSS11GP,GNRHR,CXCL2,LOC101927237,GC,SMR3B	3	gain
2790	CGTE_36	4	85654508	87707158	4q21.23-q21.3	LOC101929064,PTPN13,WDFY3-AS2,ARHGAP24,MIR4452,WDFY3,MAPK10,MIR4451	3	gain
2791	CGTE_36	4	90168870	94751351	4q22.2-q22.1	SNCA-AS1,ATOH1,CCSER1,MMRN1,GRID2,SNCA,LOC101929194,GPRIN3	3	gain
2792	CGTE_36	5	140384	3600813	5p15.33	MIR6075,CEP72,ZDHHC11,LINC01511,C5orf38,IRX1,CLPTM1L,IRX2,EXOC3-AS1,AHRR,CTD-3080P12.3,CCDC127,MIR4457,MIR4635,MIR4456,HRAT5,BRD9,LPCAT1,NDUFS6,TPPP,LOC101929034,LINC01019,SLC9A3,PLEKHG4B,SLC6A18,MIR4277,LOC100506688,PDCD6,TERT,LOC728613,MRPL36,NKD2,LOC100288152,CTD-2194D22.4,SLC6A3,SLC12A7,LOC100506858,IRX4,SDHAP3,PP7080,LRRC14B,SDHA,LINC01017,LOC100996325,EXOC3,LINC01377,SLC6A19,TRIP13	1	loss
2793	CGTE_36	5	11236869	11565109	5p15.2	CTNND2	4	gain
2794	CGTE_36	5	17275551	31323297	5p14.2-p14.3	CDH18,SNORA105B,GUSBP1,PMCHL1,LOC101929660,CDH10,LOC401177,LOC101929681,LOC101929645,LSP1P3,CDH12,CDH9,SNORA105A,CDH6,LINC01021,BASP1,C5orf17,LOC340107,PRDM9	3	gain
2795	CGTE_36	5	81608441	94267811	5q15-q14.3	KIAA0825,MCTP1,MIR548AO,LINC01339,FAM172A,EDIL3,HAPLN1,POU5F2,CETN3,LYSMD3,TMEM167A,NR2F1,MIR3660,LOC731157,LINC00461,MIR4280,LOC100505878,ARRDC3,MBLAC2,LINC01338,SLF1,MIR2277,ADGRV1,POLR3G,ARRDC3-AS1,CCNH,LOC55338,NBPF22P,MIR3607,MEF2C-AS1,TMEM161B,LOC102546226,MIR9-2,VCAN,MEF2C,COX7C,ATP6AP1L,NR2F1-AS1,TMEM161B-AS1,SCARNA18,LUCAT1,LOC644285,LOC101929380,XRCC4,MIR3977,RASA1	3	gain
2796	CGTE_36	5	126674797	130883893	5q23.3-q31.1	SLC12A2,LYRM7,CTXN3,FBN2,CDC42SE2,ADAMTS19,MEGF10,KIAA1024L,ADAMTS19-AS1,CHSY3,SLC27A6,ISOC1,LINC01184,RAPGEF6,PRRC1,MIR4460,HINT1,MIR4633	3	gain

2797	CGTE_36	6	53989270	73952276	6q12-q13	PHF3,KIAA1586,LOC101928280,KCNQ5,LINC00680,KHDRBS2,ADGRB3,ZNF451,KHDC1L,BMP5,KHDC1,GFRAL,SMAP1,EVADR,EYS,COL9A1,SDHAF4,RIMS1,PRIM2,LOC100506188,MIR548U,LOC101928307,LINC01626,HMGCLL1,FAM83B,LOC441155,LMBRD1,SLC25A51P1,LOC101927211,KCNQ5-IT1,TINAG,MIR30C2,COL19A1,B3GAT2,MIR4282,COL21A1,OGFRL1,LOC102723883,RAB23,HCRTR2,MTRNR2L9,GUSBP4,LOC101930010,MLIP,FAM135A,PTP4A1,LINC00472,DST,BEND6,BAG2,LGSN,MIR30A,LINC00680-GUSBP4,KCNQ5-AS1	3	gain
2798	CGTE_36	6	90642070	102307409	6q15-q16.2	MANEA-AS1,TSG1,MANEA,LOC101927314,LOC101927365,BACH2,UFL1,KLHL32,FBXL4,PRDM13,MCHR2-AS1,GPR63,USP45,FUT9,PNISR,MIR548H3,MAP3K7,MMS22L,FAXC,CASC6,CCNC,FHL5,EPHA7,GRIK2,COQ3,ASCC3,MIR4643,MCHR2,MIR4464,POU3F2,TSTD3,SIM1,NDUFAF4,MIR2113	3	gain
2799	CGTE_36	6	102337330	102516484	6q16.3	GRIK2	4	gain
2800	CGTE_36	7	7283156	20199925	7p22.1-p21.1	NXPH1,THSD7A,NDUFA4,BZW2,MIOS,TMEM196,COL28A1,AHR,AGMO,MEOX2,ISPD,VWDE,LOC101927668,ETV1,AGR2,SCIN,DGKB,TWISTNB,GLCCI1,PER4,TMEM106B,MIR1302-6,LOC100505938,LOC101927354,PRPS1L1,LOC101927630,SOSTDC1,LOC100505921,TWIST1,TSPAN13,MIR3146,ANKMY2,FERD3L,RPA3,MACC1-AS1,HDAC9,C1GALT1,AGR3,UMAD1,ICA1,KCCAT333,PHF14,ISPD-AS1,ARL4A,LOC101927391,MEOX2-AS1,SNX13,LRRC72,MACC1	3	gain
2801	CGTE_36	7	77756570	86781691	7q21.11-q21.12	GNAI1,DMTF1,LOC101927356,LOC101927269,CD36,SEMA3D,LOC101927378,PCLO,MAGI2-AS2,SEMA3E,RPL13AP17,SEMA3C,LOC100128317,KIAA1324L,MIR548M,GRM3,SEMA3A,MAGI2-AS3,HGF,MAGI2,GNAT3,CACNA2D1	3	gain
2802	CGTE_36	7	112407268	126698333	7q31.1-q31.31	WASL,PTPRZ1,ASB15,LOC101928211,HRAT17,SLC13A1,MIR6132,CADPS2,LINC01392,C7orf60,LOC102724555,TMEM229A,POT1,LINC01510,IQUB,MIR3666,PPP1R3A,LOC102724434,HYALP1,WNT16,ASZ1,MET,CTTNBP2,KCND2,ST7,LOC101928254,SPAM1,GRM8,ING3,LMOD2,GPR37,CAV1,LOC101928283,ST7-AS1,ST7-OT3,GPR85,TFEC,C7orf77,TMEM168,ST7-AS2,FEZF1,CAV2,LINC01393,WNT2,NDUFA5,RNF133,LSM8,POT1-AS1,TAS2R16,MIR592,LINC00998,ANKRD7,ST7-OT4,MDFIC,TSPAN12,FOXP2,RNF148,FEZF1-AS1,CAPZA2,HYAL4,RNU6-2,CPED1,FAM3C,LVCAT5,CFTR,AASS,TES	3	gain
2803	CGTE_36	7	143174822	148112765	7q36.1-q35	TPK1,CTAGE15,OR2A1-AS1,CNTNAP2,EPHA1-AS1,CTAGE6,OR2A2,OR2A9P,OR2A14,TCAF2P1,CTAGE8,ARHGEF35,ARHGEF34P,OR2A12,OR2A42,TCAF2,OR6B1,TCAF1,OR2A7,MIR548T,OR2A1,OR2A20P,OR2F1,LOC101928605,OR2F2,CTAGE4,ARHGEF5,LOC154761,MIR548I4,LOC101928700,NOBOX,TAS2R41,MIR548F4,OR2A25,OR2A5	3	gain
2804	CGTE_36	8	21861413	22105826	8p21.3	FAM160B2,POLR3D,XPO7,BMP1,DMTN,FGF17,LGI3,HR,PHYHIP,REEP4,NUDT18,MIR320A,SFTPC,NPM2	1	loss
2805	CGTE_36	8	92407257	93115056	8q21.3	SLC26A7,RUNX1T1	4	gain
2806	CGTE_36	8	104444910	113293598	8q23.3-q22.3	PKHD1L1,LINC01609,EIF3E,EBAG9,EMC2,ABRA,TRHR,TMEM74,ANGPT1,LRP12,DCAF13,DCSTAMP,CSMD3,RIMS2,SYBU,LINC01608,RSPO2,ZFPM2-AS1,KCNV1,DPYS,NUDCD1,ZFPM2,OXR1,ENY2	3	gain
2807	CGTE_36	8	113299272	116680159	8q23.3	CSMD3,TRPS1,MIR2053	4	gain

2808	CGTE_36	8	142165921	145831031	8q24.3	RHPN1-AS1,MIR7112,GPR20,MIR4472-1,LY6D,THEM6,PPP1R16A,MINCR,MIR4664,HSF1,RHPN1,MAFA-AS1,ZNF696,SLC39A4,MIR939,CDC42P3,JRK,OPLAH,TOP1MT,TSNARE1,LOC100288181,MIR6847,MIR4539,BREA2,CPSF1,MIR937,SLC52A2,LY6H,SCRIB,ZC3H3,SLC45A4,TMEM249,C8orf82,TONSL-AS1,GSDMD,PARP10,EPPK1,LINC00051,ADCK5,TONSL,DGAT1,MIR6849,DENND3,ZFP41,RECQL4,TSTA3,GPAA1,PYCRL,SCRT1,MAFA,GLI4,CYP11B1,SLURP1,MROH6,C8orf31,MAF1,LY6K,FOXH1,FBXL6,TIGD5,PSCA,KIFC2,LRRC14,PLEC,MROH1,LINC01300,ARC,SPATC1,NRBP2,CYHR1,MIR6893,LYNX1,WDR97,NAPRT,PUF60,GML,PTP4A3,LOC100133669,MIR661,ARHGAP39,MIR6846,EEF1D,ZNF623,BOP1,FAM83H-AS1,CCDC166,MIR6848,HGH1,CYC1,EXOSC4,GPIHBP1,FAM83H,SCX,MFSD3,LRRC24,LY6E,CYP11B2,GRINA,SHARPIN,ADGRB1,GPT,MAPK15,LYPD2,MIR6845,MIR1302-7,VPS28,MROH5,ZNF707	1	loss
2809	CGTE_36	9	117389051	122011495	9q32-q33.1	LOC101928775,PAPPA-AS1,TLR4,ASTN2,C9orf91,BRINP1,PAPPA,TNC,LOC100505478,LINC00474,LOC101928797,TNFSF15,DEC1,ASTN2-AS1,LOC101928748,SNORA70C,TNFSF8,TRIM32	3	gain
2810	CGTE_36	9	134019632	134021763	9q34.13	NUP214	6	gain
2811	CGTE_36	9	135820981	140510590	9q34.2-q34.13	MIR4673,SURF1,TMEM203,EXD3,LOC101448202,GRIN1,MAMDC4,SOHLH1,MIR4292,GFI1B,MAN1B1-AS1,TMEM141,SLC2A6,OBP2B,MED22,LCN8,LRRC26,LCNL1,NSMF,TOR4A,DKFZP434A062,TMEM8C,C9orf116,RNU6ATAC,MIR3689B,ADAMTS13,ENTPD2,NACC2,C8G,SNORA17B,RPL7A,EDF1,INPP5E,LOC101928525,PTGDS,COL5A1,MIR6722,GTF3C5,ARRDC1-AS1,SNORD141A,CCDC183-AS1,GLT6D1,DNLZ,PMPCA,NELFB,SNORD24,LCN1,MIR3689E,ZMYND19,STKLD1,MIR3689C,MIR4674,NRARP,QSOX2,MIR3689D2,LOC401557,MIR4479,MIR7114,TMEM210,SNHG7,MIR548AW,FAM163B,UAP1L1,FCN2,CYSRT1,PPP1R26,TUBB4B,C9orf62,ABO,OLFM1,SURF2,UBAC1,MAN1B1,LINC00094,RABL6,LHX3,C9orf69,TPRN,CEL,ARRDC1,LINC01502,MRPS2,NOXA1,GBGT1,SNORD36A,SNAPC4,MIR6877,LCN12,MIR3689A,LOC100128593,MIR3689D1,MIR3689F,SARDH,SURF6,MIR3621,RNF208,PAEP,NPDC1,SNORD141B,ENTPD8,PPP1R26-AS1,VAV2,SDCCAG3,SEC16A,C9orf172,DBH-AS1,OBP2A,DBH,MIR126,SAPCD2,SLC34A3,CAMSAP1,C9orf142,NDOR1,SNORD36C,AGPAT2,ABCA2,PHPT1,ADAMTSL2,FAM166A,WDR5,TRAF2,MIR4669,CLIC3,C9orf163,CCDC183,NALT1,C9orf173-AS1,LCN15,KCNT1,LCN6,CELP,MRPL41,FCN1,RNF224,C9orf173,RALGDS,C9orf139,LCN9,SSNA1,SURF4,BRD3,NOTCH1,FAM69B,REXO4,FBXW5,GPSM1,ANAPC2,DPP7,FUT7,LCN10,CARD9,SNORD36B,DPH7,CACFD1,SNORA17A,RXRA,PNPLA7,EGFL7	1	loss
2812	CGTE_36	10	52002917	59956192	10q21.1-q11.23	PCDH15,PRKG1,CSTF2T,LINC01468,ZWINT,LOC102724719,ASAH2,A1CF,PRKG1-AS1,MTRNR2L5,DKK1,MIR3924,IPMK,ASAH2B,MIR605,SGMS1,SGMS1-AS1,MBL2	3	gain
2813	CGTE_36	10	65383534	69571375	10q21.3	CTNNA3,MIR7151,LOC101928961,DNAJC12,LRRTM3,REEP3,LINC01515,ANXA2P3	3	gain

2814	CGTE_36	10	131557385	135373691	10q26.3	PAOX,MIR378C,MIR4297,FUOM,KNDC1,SCART1,ZNF511,UTF1,CTAGE7P,SPRN,MIR3944,NKX6-2,LINC01167,TUBGCP2,PPP2R2D,C10orf91,ECHS1,SYCE1,STK32C,ADGRA1-AS1,MTG1,BNIP3,TCERG1L,LINC01164,JAKMIP3,DPYSL4,EBF3,VENTX,PWWP2B,CALY,LINC01166,MIR202HG,LINC01168,ADAM8,TCERG1L-AS1,MIR202,ADGRA1,PRAP1,GLRX3,INPP5A,LRRC27,CYP2E1,LINC00959,CFAP46,MGMT	1	loss
2815	CGTE_36	11	193051	3382192	11p15.4-p15.5	BET1L,PHLDA2,TOLLIP-AS1,NAP1L4,MIR6743,ODF3,SLC22A18AS,TH,MRGPRG,SCT,KCNQ1,ZNF195,MUC5B,KCNQ1-AS1,CD81-AS1,CDHR5,MIR483,KRTAP5-3,TOLLIP,PSMD13,RIC8A,IGF2,PIDD1,LMNTD2,PTDSS2,B4GALNT4,MUC2,ANO9,LOC143666,SNORD131,IFITM1,NLRP6,ASCL2,TNNI2,MOB2,KRTAP5-1,IFITM2,KRTAP5-4,EPS8L2,CD81,IFITM5,DUSP8,SYT8,KCNQ1OT1,CDKN1C,TSPAN32,KRTAP5-6,OSBPL5,INS-IGF2,CD151,MIR210HG,CHID1,PANO1,SCGB1C2,INS,HRAS,CEND1,MIR7847,MIR210,TSSC4,MRGPRG-AS1,IFITM3,PDDC1,C11orf21,SNORA54,TMEM80,BRSK2,DEAF1,DRD4,CRACR2B,KRTAP5-2,LINC01219,SLC22A18,SIGIRR,RNH1,KRTAP5-5,CTSD,ATHL1,MRPL23-AS1,SCGB1C1,RASSF7,LSP1,MIR4298,MRPL23,TNNT3,IGF2-AS,H19,RPLP2,HOTS,IRF7,MIR6744,PHRF1,MIR675,SNORA52,FAM99B,TSPAN4,TALDO1,PKP3,SIRT3,MUC6,MIR4686,LRRC56,MUC5AC,MRGPRE,FAM99A,KCNQ1DN,CARS,LINC01150,LOC171391,IFITM10,AP2A2,POLR2L,TRPM5,KRTAP5-AS1,PNPLA2,SLC25A22	1	loss
2816	CGTE_36	11	4411231	6221397	11p15.4	HBB,OR52A1,HBE1,TRIM6-TRIM34,OR52N5,OR51F1,HBG1,OR52W1,OR52E8,OR52E6,OR51V1,TRIM5,OR52K1,OR52B6,OR51E2,OR51B5,UBQLN3,OR51A4,OR52R1,OR51B6,OR56A4,OR51I1,HBBP1,OR51L1,OR51B4,OR51S1,OR52M1,OR51T1,OR51Q1,TRIM22,OR52N1,OR51G2,TRIM68,OR52J3,OR51G1,OR52I2,OR56A5,OR52A5,OR56B1,OR52D1,UBQLNL,OR52K2,TRIM21,OR51F2,OR51M1,OR56B4,OR51B2,OR51I2,OR52N2,OR52B2,OR52E2,TRIM6,OR52E4,C11orf40,OR52N4,OR51E1,HBD,MMP26,OR51A7,OR56A1,HBG2,OR56A3,TRIM34,OR52H1,OLFM5P,OR52I1,OR51A2,OR52L1,OR51D1,BGLT3	3	gain
2817	CGTE_36	11	16010658	16760572	11p15.1-p15.2	C11orf58,SOX6	4	gain
2818	CGTE_36	11	21250782	31812602	11p13-p14.3	DCDC1,LINC01495,DCDC5,ANO5,BBOX1-AS1,LIN7C,FIBIN,MPPED2,DNAJC24,IMMP1L,ELP4,FANCF,SLC5A12,BBOX1,SVIP,FSHB,ANO3,LINC01616,BDNF,BDNF-AS,MUC15,KIF18A,GAS2,LOC105376671,ARL14EP,SLC17A6,CCDC34,LUZP2,NELL1,MIR8054,KCNA4,CCDC179,MIR8068,LINC00678,LOC105376599,MIR610,PAX6,METTL15,LGR4,MIR8087	3	gain
2819	CGTE_36	11	48238466	56805710	11p11.12-q12.1	OR4A16,OR5AR1,LOC646813,OR8U8,OR5T2,OR5AS1,TRIM49B,OR4A47,OR5M8,LOC441601,OR5I1,OR4X2,OR9G4,OR5AK4P,OR4S1,OR8H2,LOC440040,OR4C6,OR5M9,LOC101927120,OR5D16,OR4C11,OR4B1,OR5L2,OR5D13,OR9G9,OR5F1,OR5M10,TRIM48,OR8I2,OR8K3,OR4A15,MIR6128,OR4C16,TRIM51HP,OR8H1,OR4A5,OR8J3,OR4S2,OR4C46,OR8U1,TRIM51,FOLH1,OR5T1,OR8K5,OR5AP2,OR4P4,OR5AK2,OR5L1,OR4C13,OR4C12,OR5M11,OR4C15,OR5D14,OR4C45,OR4C3,OR5T3,TRIM64C,OR8K1,OR5M3,OR4X1,OR9G1,OR5W2,OR5M1,OR8H3,OR5R1,OR5J2,OR7E5P,OR5D18,OR8J1,OR10AG1	3	gain

2820	CGTE_36	11	57596534	58722829	11q12.1	OR5B12,OR5B17,OR9Q1,OR5B21,OR5B2,OR10W1,LOC283194,OR1S2,OR6Q1,CNTF,ZFP91-CNTF,GLYATL1,LPXN,GLYATL2,OR9Q2,OR1S1,OR9I1,OR5B3,GLYAT,OR10Q1,ZFP91	3	gain
2821	CGTE_36	11	62848412	63274057	11q12.3	HRASLS5,SLC22A10,SLC22A24,SLC22A9,SLC22A25,LGALS12	3	gain
2822	CGTE_36	11	76169176	76261291	11q13.5	EMSY	3	gain
2823	CGTE_36	11	102820827	106810853	11q22.3-q22.2	GRIA4,LOC101928535,DCUN1D5,MIR7641-1,GUCY1A2,DDI1,KBTBD3,CASP12,CARD18,CASP1,CASP4,CARD16,LOC102723895,PDGFD,MSANTD4,MIR4693,CARD17,LOC643733,MMP13,DYNC2H1,CASP1P2,CASP5,AASDHPPT	3	gain
2824	CGTE_36	11	123600383	124440854	11q24.1-q24.2	OR8G1,OR8G5,OR8B3,OR10G4,OR10G9,TMEM225,OR8D1,VWA5A,OR6X1,OR10G8,OR8B12,OR8B8,OR8D4,OR10S1,OR8B4,OR8A1,OR6T1,OR6M1,OR8D2,ZNF202,OR8G2,OR4D5,OR8B2,OR10G7	3	gain
2825	CGTE_36	12	936240	1519654	12p13.33	RAD52,ERC1,WNK1	3	gain
2826	CGTE_36	12	7457142	7656317	12p13.31	ACSM4,CD163L1,CD163	3	gain
2827	CGTE_36	12	21712538	24971102	12p12.1	C2CD5,CMAS,GYS2,LOC101928441,BCAT1,LINC00477,KCNJ8,ST8SIA1,LOC101928471,LDHB,ABCC9,ETNK1,MIR920,SOX5	3	gain
2828	CGTE_36	12	32974273	40869542	12q12-p11.1	CPNE8,C12orf40,ABCD2,KIF21A,ALG10,SYT10,ALG10B,PKP2,MUC19,SLC2A13,LRRK2	3	gain
2829	CGTE_36	12	40898772	40899184	12q12	MUC19	6	gain
2830	CGTE_36	12	48131314	48419029	12q13.11	SLC48A1,RAPGEF3,HDAC7,COL2A1,TMEM106C,VDR	1	loss
2831	CGTE_36	12	55233149	56031597	12q13.2	TESPA1,OR6C2,OR6C4,OR2AP1,NEUROD4,OR9K2,OR6C1,OR10A7,OR6C68,OR6C3,OR10P1,OR6C76,OR6C6,OR6C65,OR6C70,MUCL1,OR6C75,OR6C74	3	gain
2832	CGTE_36	12	57556550	57631604	12q13.3	NDUFA4L2,SHMT2,NXPH4,MIR1228,LRP1	1	loss
2833	CGTE_36	12	59197019	62861194	12q14.1	MON2,MIR6125,LRIG3,LOC100506869,FAM19A2,SLC16A7,USP15	3	gain
2834	CGTE_36	12	70507857	93245057	12q21.33-q22	SLC6A15,ATP2B1,LOC728084,LINC01481,POC1B,LUM,TMEM19,LOC101928137,LINC01619,OSBPL8,KCNC2,ATXN7L3B,DUSP6,BBS10,LOC100507377,RAB21,MYF5,NAV3,C12orf74,PTPRQ,MIR5692B,THAP2,NTS,E2F7,CCDC59,LOC101928449,MIR4699,KRR1,C12orf29,POC1B-GALNT4,METTL25,PLEKHG7,MIR548AL,PAWR,DCN,MKRN9P,TPH2,MRS2P2,GLIPR1,GLIPR1L2,LINC00936,TMTC3,MIR618,ACSS3,TSPAN8,PPP1R12A,ZFC3H1,PHLDA1,LINC01490,PTPRR,TMTC2,TRHDE,MIR617,C12orf50,ZDHHC17,OTOGL,KERA,CLLU1,MGAT4C,NAP1L1,LRRIQ1,CLLU1OS,RASSF9,MYF6,LINC00615,MIR1252,BTG1,SYT1,EPYC,LIN7A,ALX1,CNOT2,CEP290,LGR5,TBC1D15,TRHDE-AS1,CSRP2,TSPAN19,PPFIA2,CAPS2,KITLG,KCNMB4,GALNT4,CCER1,GLIPR1L1,PTPRB,EEA1,LOC102724663	3	gain
2835	CGTE_36	12	115120858	117161082	12q24.22-q24.21	C12orf49,LINC00173,MED13L,MAP1LC3B2,MIR4472-2,TBX3,MIR620	3	gain
2836	CGTE_36	13	37582861	41134195	13q14.11-q13.3	LINC00548,LINC01048,LINC00571,COG6,LINC00332,STOML3,FOXO1,FREM2,PROSER1,NHLRC3,CSNK1A1L,LINC00547,LHFP,SUPT20H,LINC00598,TRPC4,LINC00437,POSTN,EXOSC8,UFM1,LINC00366,MIR4305	3	gain
2837	CGTE_36	13	61986973	72147771	13q21.33-q21.32	PCDH9-AS4,LINC00376,DACH1,MIR548X2,OR7E156P,LINC01052,PCDH9,PCDH20,LINC00364,LINC00448,LOC102723968,PCDH9-AS2,LINC01075,PCDH9-AS3,KLHL1,MIR4704,LINC00395,LINC00550,LINC00348,ATXN8OS,LINC00383,LINC00358	3	gain
2838	CGTE_36	13	75886936	75900570	13q22.2	TBC1D4	6	gain

2839	CGTE_36	13	75901833	95673924	13q22.2-q31.1	LINC01047,SOX21-AS1,LMO7,EDNRB-AS1,LMO7-AS1,LINC00564,LINC00382,MIR4500HG,GPC6,SLAIN1,LINC01068,LOC100129307,GPC6-AS2,LINC01038,TGDS,LOC101927248,MIR17HG,MIR92A1,SOX21,LINC00380,RBM26-AS1,LINC00377,MIR4500,GPR180,RNF219-AS1,RBM26,MIR17,LOC105370306,SPRY2,LINC00353,LINC00379,ABCC4,LINC01069,LINC00410,LINC00363,MIR19A,FBXL3,LINC00333,TBC1D4,LINC00430,COMMD6,MYCBP2,MIR3665,GPC5-AS1,GPC5,NDFIP2-AS1,IRG1,SLITRK5,SCEL-AS1,SLITRK6,LINC00351,LINC00440,BTF3P11,EDNRB,LINC01040,GPC6-AS1,LINC01080,LINC00557,SNORA107,LINC00433,MIR18A,LOC101927284,UCHL3,MIR19B1,SLITRK1,LMO7DN-IT1,MIR622,CLN5,RNF219,LINC00559,LINC00331,GPC5-AS2,KCTD12,LINC01049,MYCBP2-AS1,LMO7DN,NDFIP2,LINC00397,MIR20A,SCEL,LINC00375,LINC00446,DCT,POU4F1	3	gain
2840	CGTE_36	14	20201784	20757992	14q11.2	OR4K14,OR4K5,OR4M1,OR11H4,OR4K2,OR4N5,OR4Q3,OR4K1,OR4N2,OR11H6,OR4K13,TTC5,OR4L1,OR4K15,OR4K17,OR11G2	3	gain
2841	CGTE_36	14	21969002	22891639	14q11.2	SALL2,METTL3,OR4E1,OR4E2,OR10G3,OR10G2	3	gain
2842	CGTE_36	14	24911804	31085566	14q12	GZMH,LOC102724890,LINC00645,MIR4307,NOVA1,GZMB,FOXG1,PRKD1,MIR4307HG,SDR39U1,MIR548AI,CMA1,MIR3171,FOXG1-AS1,LOC101927045,LINC01551,G2E3,LOC101927062,STXBP6,CTSG	3	gain
2843	CGTE_36	14	47120321	50065272	14q21.3-q21.2	MDGA2,LINC00648,MIR548Y,RPL10L,RPS29	3	gain
2844	CGTE_36	14	56099901	57075890	14q22.3	KTN1,TMEM260,RPL13AP3,PELI2,LINC00520	3	gain
2845	CGTE_36	14	60756531	60903758	14q23.1	C14orf39,PPM1A	4	gain
2846	CGTE_36	14	67431989	67463001	14q23.3	GPHN	4	gain
2847	CGTE_36	14	71444173	71462720	14q24.2	PCNX	5	gain
2848	CGTE_36	14	71476313	72176523	14q24.2	PCNX,SIPA1L1,LOC145474,SNORD56B	3	gain
2849	CGTE_36	14	79111527	80677881	14q31.1-q24.3	DIO2,DIO2-AS1,NRXN3	3	gain
2850	CGTE_36	14	104565101	106437088	14q32.33	CRIP1,ZBTB42,MIR8071-2,BTBD6,NUDT14,MIR203A,CRIP2,CDCA4,C14orf180,ADAM6,LOC102723354,TMEM179,TMEM121,KIAA0125,ELK2AP,MIR8071-1,KIF26A,PLD4,AKT1,LINC00638,BRF1,PACS2,LOC100507437,CEP170B,ASPG,MIR6765,MIR4710,MIR203B,C14orf79,MIR4507,SIVA1,ADSSL1,MTA1,GPR132,JAG2,TEX22,MIR4537,MIR4539,MIR4538,AHNAK2,C14orf80,INF2	1	loss
2851	CGTE_36	15	25415952	25584361	15q11.2	SNORD115-15,SNORD115-34,SNORD115-28,UBE3A,SNORD115-8,SNORD115-31,SNORD115-12,SNORD115-2,SNORD115-26,SNORD115-46,SNORD115-36,SNORD115-37,SNORD115-5,SNORD115-47,SNORD115-10,SNORD115-33,SNORD115-21,SNORD115-7,SNORD115-6,SNORD115-32,SNORD115-3,SNORD115-29,SNORD115-27,SNORD115-20,SNORD115-14,SNORD115-44,SNORD115-25,SNORD115-30,SNORD115-13,SNORD115-39,SNORD115-45,SNORD115-4,SNORD115-17,SNORD115-24,SNORD115-18,SNORD109A,SNORD115-23,SNORD115-22,SNORD115-9,SNORD115-41,SNORD115-40,SNORD115-11,SNORD115-42,SNORD115-19,SNORD115-16,SNORD115-48,PWAR4,SNORD115-43,SNORD109B,SNORD115-38,SNORD115-35	1	loss

2852	CGTE_36	16	97450	3141587	16p13.3	MIR6511B2,TPSD1,TELO2,E4F1,CCDC78,NUBP2,CHTF18,TSC2,GNPTG,PRSS21,LINC00514,MMP25-AS1,SNRNP25,MIR5587,CCDC64B,WDR90,AXIN1,TBC1D24,MRPS34,FLYWCH1,MIR6768,SNHG9,LOC652276,RPL3L,NHLRC4,PRSS30P,RAB11FIP3,HAGH,NOXO1,NTHL1,CLCN7,JMJD8,POLR3K,CLDN9,PRR25,ZG16B,C16orf91,LUC7L,MEIOB,FAM173A,LOC100134368,CRAMP1,WDR24,C1QTNF8,PRR35,C16orf59,RAB40C,LMF1,CCNF,RNF151,METRN,PKD1,MSLN,FLJ42627,MIR1225,LOC100128770,MIR662,THOC6,MIR3180-5,TPSB2,PDPK1,TBL3,CACNA1H,KCTD5,UBE2I,HBA1,ATP6V0C,FAM195A,MRPL28,IL32,NDUFB10,RAB26,RPUSD1,NME4,TRAF7,MSRB1,RNPS1,DECR2,SNORA64,LOC106660606,TCEB2,DNASE1L2,UNKL,SLC9A3R2,RHOT2,EME2,TSR3,SYNGR3,TPSAB1,SOX8,PAQR4,MIR3177,PTX4,MIR3677,NTN3,NME3,TMEM204,RPS2,NPW,MIR3178,HN1L,HCFC1R1,BRICD5,HAGHL,LOC101929613,ERVK13-1,MIR6511B1,AMDHD2,RGS11,ARHGDIG,HBZ,PRSS27,MIR6767,LINC00235,ZNF598,TMEM8A,SRRM2-AS1,CCDC154,PDIA2,C16orf13,SPSB3,STUB1,PKMYT1,RHBDL1,HBM,PRSS22,PIGQ,MLST8,NPRL3,ABCA17P,IFT140,TPSG1,MAPK8IP3,IGFALS,CASKIN1,MIR4516,BAIAP3,MMP25,CEMP1,SNHG19,HS3ST6,KREMEN2,PRSS33,RHBDF1,GNG13,SNORA10,WFIKKN1,HBQ1,LINC00254,GFER,MPG,CAPN15,TNFRSF12A,PGP,SNORA78,FBXL16,FLYWCH2,HBA2,SSTR5,ECI1,MIR3176,MIR4717,SNORD60,CLDN6,SSTR5-AS1,PRSS41,SRRM2,ZSCAN10,MIR940,NARFL,FAHD1,ABCA3,LMF1-AS1,FAM234A	1	loss
2853	CGTE_36	16	52473073	52498014	16q12.1	TOX3	4	gain
2854	CGTE_36	16	59772750	66432016	16q21	LOC729159,MIR4426,LINC00922,APOOP5,CDH5,CDH11,LOC101927580,CDH8,LOC101927650	3	gain
2855	CGTE_36	16	76311542	76592589	16q23.1	CNTNAP4	3	gain
2856	CGTE_36	16	85109414	90142246	16q24.3-q24.1	LOC400558,TUBB3,ACSF3,GAS8-AS1,ZNF276,MVD,VPS9D1-AS1,MIR5093,LOC100287036,TCF25,ZFPM1,FOXL1,DBNDD1,TRAPPC2L,SPATA33,LOC100289580,SLC7A5,FOXC2-AS1,CDH15,LOC101928682,EMC8,RNF166,FBXO31,GINS2,GAS8,LOC101928737,ZNF469,CDT1,BANP,ZC3H18,CHMP1A,LOC100129697,PABPN1L,ZNF778,MIR6774,VPS9D1,SNAI3-AS1,LOC101928708,LOC146513,SPATA2L,LOC400548,CA5A,ZCCHC14,LINC00917,PIEZO1,MIR4722,MTHFSD,FAM92B,FOXF1,KLHDC4,LOC339059,DPEP1,LOC400553,SPIRE2,MIR1910,C16orf95,CDK10,SNORD68,LINC00311,LINC01082,LINC00304,JPH3,AFG3L1P,MC1R,SPG7,FOXC2,RPL13,LOC440390,APRT,DEF8,MIR5189,CPNE7,PRDM7,FANCA,LOC101928880,LOC101927817,SLC22A31,CTU2,FLJ30679,GALNS,MIR6775,KIAA0513,LINC01081,COX4I1,CENPBD1,URAHP,CYBA,GSE1,FENDRR,C16orf74,MAP1LC3B,CBFA2T3,LOC102724467,SNAI3,IRF8,ANKRD11,IL17C	1	loss
2857	CGTE_36	17	39140011	39502449	17q21.2	KRTAP4-3,KRTAP9-8,KRTAP4-6,KRTAP3-3,KRTAP9-2,KRTAP4-5,KRTAP9-3,KRTAP4-7,KRTAP1-4,KRTAP17-1,KRT33A,KRTAP4-8,KRTAP1-3,KRTAP4-9,KRTAP9-1,KRT40,KRTAP4-2,KRTAP4-4,KRTAP4-12,KRTAP9-9,KRTAP1-5,KRTAP4-11,KRTAP16-1,KRTAP1-1,KRTAP9-6,KRTAP2-3,KRTAP3-2,KRTAP2-2,KRTAP9-7,KRTAP3-1,KRTAP2-4,KRTAP4-1,KRTAP9-4,KRTAP29-1,KRTAP2-1	4	gain
2858	CGTE_36	17	48606345	48699162	17q21.33	MYCBPAP,CACNA1G,EPN3,SPATA20,CACNA1G-AS1	1	loss
2859	CGTE_36	17	61086885	61483755	17q23.2-q23.3	TANC2,MIR548W	3	gain

2860	CGTE_36	17	78831399	81006711	17q25.3	OGFOD3,AATK,DCXR,ACTG1,FN3KRP,ARL16,ENTHD2,BAIAP2-AS1,MIR3065,FAM195B,AATK-AS1,SNORD134,ASPSCR1,UTS2R,ANAPC11,NPB,FN3K,NOTUM,WDR45B,SLC16A3,PPP1R27,CD7,STRA13,BAHCC1,FASN,MIR4740,PYCR1,HEXDC,TEX19,MRPL12,SLC38A10,PDE6G,MIR657,GCGR,MYADML2,FAAP100,RPTOR,TMEM105,RAC3,SECTM1,MIR1250,MIR6787,MAFG,SLC25A10,MIR338,B3GNTL1,CEP131,RAB40B,LRRC45,C17orf62,P4HB,DUS1L,C17orf89,HGS,CHMP6,TSPAN10,MIR4525,NPLOC4,FOXK2,MAFG-AS1,OXLD1,FSCN2,PCYT2,ALYREF,RFNG,SIRT7,MIR6786,GPS1,ARHGDIA,ZNF750,BAIAP2,CSNK1D,LOC100130370,NARF,LINC00482,CCDC137,CCDC57,TBCD,MIR3186	1	loss
2861	CGTE_36	18	37256610	43311155	18q12.3	LOC105667213,SLC14A2-AS1,LINC01477,KC6,SLC14A2,MIR4319,LINC01478,SLC14A1,MIR924HG,PIK3C3,MIR5583-1,SYT4,MIR5583-2,SETBP1,LINC00907,RIT2	3	gain
2862	CGTE_36	18	48575631	55020278	18q21.31-q21.2	LOC102724651,SNORA37,LOC101928167,LINC-ROR,TXNL1,MIR4529,CCDC68,RAB27B,TCF4-AS1,MIR4528,DYNAP,LOC100287225,ST8SIA3,TCF4,POLI,LINC01416,LOC101927229,SMAD4,WDR7,BOD1L2,MBD2,C18orf54,LINC01539,DCC,STARD6,MEX3C	3	gain
2863	CGTE_36	18	59212212	59972858	18q21.33	PIGN,LINC01544,RNF152,CDH20,KIAA1468	3	gain
2864	CGTE_36	18	63430205	64239437	18q22.1	CDH19,CDH7	4	gain
2865	CGTE_36	19	281411	2255477	19p13.3	PCSK4,MIR4745,GAMT,C19orf24,REXO1,POLR2E,KLF16,MOB3A,EFNA2,ADAT3,ABHD17A,ATP5D,GPX4,SHC2,AZU1,CIRBP,PRTN3,RPS15,CNN2,R3HDM4,GRIN3B,CBARP,ADAMTSL5,ARID3A,MKNK2,JSRP1,MISP,MIR1227,RNF126,ODF3L2,MIR4321,MADCAM1,WDR18,MED16,NDUFS7,SBNO2,BSG,MEX3D,PLPP2,TPGS1,ONECUT3,SCAMP4,TCF3,THEG,HCN2,CSNK1G2-AS1,PRSS57,PTBP1,CIRBP-AS1,DOT1L,UQCR11,PLPPR3,FSTL3,KISS1R,REEP6,MUM1,MBD3,IZUMO4,TMEM259,PLEKHJ1,MIER2,MIR6789,LOC100288123,CFD,APC2,AMH,MIR1909,FGF22,GZMM,POLRMT,HMHA1,ATP8B3,ABCA7,CDC34,AP3D1,C2CD4C,MIR3187,SF3A2,DAZAP1,ELANE,C19orf25,STK11,BTBD2,PALM,RNU6-2,CSNK1G2,PLK5,MIDN	1	loss
2866	CGTE_36	19	15222068	15586246	19p13.12	PGLYRP2,ILVBL,AKAP8,SYDE1,RASAL3,WIZ,MIR6795,EPHX3,MIR1470,NOTCH3,BRD4,AKAP8L	1	loss
2867	CGTE_36	19	16059846	16060573	19p13.12	OR10H4	5	gain
2868	CGTE_36	19	41403474	41622705	19q13.2	CYP2B6,CYP2G1P,CYP2B7P,CYP2A13,CYP2F1	3	gain
2869	CGTE_36	19	44663724	45127285	19q13.31	ZNF180,ZNF229,ZNF112,IGSF23,ZNF235,ZNF285,CEACAM20,ZNF227,ZNF234,CEACAM22P,ZNF233,ZNF226	3	gain
2870	CGTE_36	20	3640520	3845406	20p13	CENPB,MAVS,SIGLEC1,ADAM33,CDC25B,GFRA4,AP5S1,LOC101929125,HSPA12B,C20orf27,SPEF1	1	loss

2871	CGTE_36	20	59827521	62905019	20q13.33	ZGPAT,SRMS,TAF4,MIR1914,RBBP8NL,LOC63930,YTHDF1,SLCO4A1-AS1,BIRC7,LKAAEAR1,BHLHE23,LIME1,PRPF6,SAMD10,NTSR1,SLCO4A1,HELZ2,LAMA5,COL9A3,MIR124-3,TNFRSF6B,CDH4,LOC100505771,NPBWR2,MIR941-5,MIR3196,HAR1A,OGFR-AS1,HAR1B,DNAJC5,FLJ16779,ADRM1,MIR941-3,MIR6813,CHRNA4,MIR3195,NKAIN4,MIR1-1,PCMTD2,GMEB2,PPDPF,MTG2,STMN3,KCNQ2,OPRL1,RPS21,UCKL1,MIR1257,GATA5,LINC00659,RTEL1-TNFRSF6B,ZNF512B,MIR133A2,CABLES2,LAMA5-AS1,MIR4326,UCKL1-AS1,OSBPL2,MIR941-1,ARFGAP1,SLC2A4RG,TCFL5,LINC01056,LINC00176,PTK6,ABHD16B,MYT1,OGFR,MIR4758,TPD52L2,MIR941-4,RGS19,MRGBP,PSMA7,DPH3P1,HRH3,RTEL1,LOC100130587,ZBTB46-AS1,SLC17A9,TCEA2,LSM14B,DIDO1,ZBTB46,C20orf195,LINC00029,C20orf166-AS1,GID8,MIR1-1HG,SOX18,ARFRP1,COL20A1,MIR941-2,MIR647,SS18L1,EEF1A2	1	loss
2872	CGTE_36	21	10793919	26946466	21q21.2-q11.2	USP25,ANKRD30BP2,CHODL,BAGE3,ANKRD20A11P,SAMSN1-AS1,LINC00158,MIR548XHG,LINC00317,MIR99A,CXADR,LOC388813,POTED,NCAM2,MIR155,MIR155HG,LINC01425,C21orf91-OT1,LOC339622,MIR99AHG,LOC101927869,NRIP1,BAGE4,ABCC13,LINC00320,BAGE2,LOC101927843,BAGE,MIR3156-3,LIPI,C21orf91,HSPA13,TPTE,LOC102724188,BTG3,MIRLET7C,LINC00308,LINC01549,MIR8069-2,CYP4F29P,MIR8069-1,RBM11,CHODL-AS1,MIR3118-1,D21S2088E,BAGE5,SAMSN1,TMPRSS15,MIR125B2	3	gain
2873	CGTE_36	21	31538390	32410703	21q22.11	KRTAP7-1,KRTAP21-3,KRTAP22-1,KRTAP6-1,KRTAP21-2,KRTAP20-4,KRTAP13-3,KRTAP20-1,KRTAP21-1,KRTAP19-7,KRTAP19-3,KRTAP20-3,KRTAP19-5,KRTAP13-1,KRTAP19-6,KRTAP23-1,CLDN17,KRTAP27-1,MIR4327,KRTAP6-3,KRTAP19-2,KRTAP8-1,KRTAP19-8,KRTAP19-4,KRTAP25-1,KRTAP24-1,KRTAP13-4,KRTAP15-1,KRTAP13-2,LINC00307,CLDN8,KRTAP11-1,KRTAP26-1,KRTAP22-2,KRTAP6-2,KRTAP19-1,KRTAP20-2	4	gain
2874	CGTE_36	21	32410708	33083875	21q22.11	KRTAP19-8,TIAM1,SOD1,SCAF4	3	gain
2875	CGTE_36	21	43982195	47865338	21q22.3	PCBP3,LRRC3-AS1,ITGB2-AS1,C21orf58,C21orf33,TCONS_00029157,MIR6815,KRTAP12-2,LOC101928796,TRPM2,U2AF1,KRTAP12-4,PWP2,MCM3AP-AS1,COL18A1,KRTAP10-8,KRTAP12-3,KRTAP10-7,TSPEAR-AS1,HSF2BP,FAM207A,RRP1,PTTG1IP,ADARB1,LOC101928233,KRTAP12-1,LINC00162,FRGCA,KRTAP10-9,FTCD,CSTB,CRYAA,SSR4P1,LOC106780825,PKNOX1,YBEY,LINC01547,LOC102724652,TSPEAR-AS2,COL18A1-AS2,KRTAP10-1,WDR4,PCNT,MCM3AP,KRTAP10-2,PFKL,KRTAP10-5,UBE2G2,RRP1B,COL18A1-AS1,ICOSLG,KRTAP10-4,SIK1,KRTAP10-10,CBS,NDUFV3,AGPAT3,MIR6070,AATBC,TRPM2-AS,SPATC1L,KRTAP10-3,SUMO3,LINC01424,LINC00319,LSS,ERVH48-1,LINC00313,PDE9A,SLC19A1,TSPEAR,LOC642852,PDXK,LINC00316,KRTAP10-12,C21orf2,TRAPPC10,LINC00322,SLC37A1,COL6A2,COL6A1,KRTAP10-6,LINC00163,KRTAP10-11,LOC100129027,ITGB2,AIRE,DNMT3L,LRRC3,POFUT2	1	loss

2876	CGTE_36	22	19503841	20735045	22q11.21	TANGO2,SEPT5-GP1BB,LINC00896,RIMBP3,LINC00895,GP1BB,LOC284865,DGCR6L,PI4KAP1,TRMT2A,MIR1306,MIR185,TBX1,C22orf29,CLDN5,RANBP1,ARVCF,TXNRD2,DGCR8,GNB1L,MIR3618,MIR1286,ZDHHC8,CCDC188,COMT,TMEM191B,RTN4R,SEPT5,MIR4761,MIR6816,CDC45	1	loss
2877	CGTE_36	22	21303902	22327184	22q11.21-q11.22	YPEL1,LOC101928891,MIR649,PPIL2,PI4KAP2,POM121L8P,P2RX6P,THAP7,LZTR1,HIC2,RIMBP3B,P2RX6,BCRP2,CCDC116,FAM230B,TOP3B,RIMBP3C,CRKL,THAP7-AS1,UBE2L3,MAPK1,AIFM3,LRRC74B,TUBA3FP,YDJC,PPM1F,SDF2L1,SLC7A4,MIR130B,TMEM191C,MIR301B	1	loss
2878	CGTE_36	22	46239411	51178618	22q13.32-q13.31	TBC1D22A-AS1,SYCE3,PRR34,ODF3B,PLXNB2,PPP6R2,ACR,TYMP,BRD1,MIR6821,MIOX,LL22NC03-75H12.2,LINC00898,TBC1D22A,ZBED4,SELO,MIRLET7BHG,CHKB-CPT1B,PKDREJ,LOC284933,FAM19A5,CELSR1,SCO2,NCAPH2,MIR3667,SHANK3,LINC00899,LINC01310,TRMU,C22orf34,CRELD2,MAPK11,IL17REL,ATXN10,MAPK12,ALG12,PRR34-AS1,DENND6B,CPT1B,ADM2,MIR4535,TRABD,LOC730668,MAPK8IP2,MIRLET7A3,SBF1,KLHDC7B,TUBGCP6,GTSE1-AS1,CHKB,PIM3,GTSE1,MIR4763,ARSA,LOC284930,MIR3619,CHKB-AS1,CDPF1,MOV10L1,PPARA,GRAMD4,PANX2,TTC38,LMF2,CERK,WNT7B,MLC1,HDAC10,MIR3201,MIRLET7B	1	loss
2879	CGTE_36	X	35937726	35938206	Xp21.1	CFAP47	3	gain
2880	CGTE_37	1	865582	1154087	1p36.33	PERM1,LINC01342,TNFRSF4,AGRN,HES4,SAMD11,MIR200A,KLHL17,SDF4,PLEKHN1,ISG15,NOC2L,MIR429,C1orf159,TNFRSF18,TTLL10,RNF223,MIR200B	1	loss
2881	CGTE_37	1	1847080	1861804	1p36.33	CFAP74,TMEM52,CALML6	1	loss
2882	CGTE_37	1	3385306	3512096	1p36.32	ARHGEF16,MIR551A,MEGF6	1	loss
2883	CGTE_37	1	16057077	16074126	1p36.21	TMEM82,SLC25A34,PLEKHM2	0	loss
2884	CGTE_37	1	21903838	21938079	1p36.12	ALPL,RAP1GAP	1	loss
2885	CGTE_37	1	91447822	91782074	1p22.2	ZNF644,HFM1	1	loss
2886	CGTE_37	1	109810418	109814923	1p13.3	CELSR2	1	loss
2887	CGTE_37	1	150528674	150533880	1q21.3	ADAMTSL4-AS1,ADAMTSL4	1	loss
2888	CGTE_37	1	152009228	152328837	1q21.3	FLG,FLG2,LOC100131107,HRNR,S100A11,FLG-AS1,TCHH,RPTN,TCHHL1	3	gain
2889	CGTE_37	1	160053853	160093246	1q23.2	ATP1A2,IGSF8,KCNJ9	1	loss
2890	CGTE_37	1	228459619	228476571	1q42.13	OBSCN	1	loss
2891	CGTE_37	2	73487473	73491115	2p13.2	FBXO41	0	loss
2892	CGTE_37	2	97529581	97544275	2q11.2	SEMA4C,FAM178B	1	loss
2893	CGTE_37	2	128381736	128394207	2q14.3	MYO7B	1	loss
2894	CGTE_37	2	201397638	201401012	2q33.1	SGOL2	0	loss
2895	CGTE_37	2	233194365	233405564	2q37.1	DIS3L2,ECEL1,ALPI,ALPP,CHRND,PRSS56,CHRNG,ALPPL2,ECEL1P2	1	loss
2896	CGTE_37	2	241627709	241979828	2q37.3	AGXT,C2orf54,AQP12A,KIF1A,SNED1,LOC200772	1	loss
2897	CGTE_37	3	13659607	13896292	3p25.1	LINC00620,WNT7A,FBLN2,SNORA93	1	loss
2898	CGTE_37	3	46722558	46729876	3p21.31	ALS2CL	1	loss
2899	CGTE_37	3	47018137	47049379	3p21.31	NBEAL2,CCDC12	1	loss
2900	CGTE_37	3	49688283	49701348	3p21.31	BSN	1	loss
2901	CGTE_37	3	52521197	52523688	3p21.1	NISCH	0	loss
2902	CGTE_37	3	52523945	52562997	3p21.1	STAB1,NISCH,NT5DC2	1	loss
2903	CGTE_37	3	126707504	126741135	3q21.3	PLXNA1	1	loss
2904	CGTE_37	3	195596345	195609438	3q29	TNK2,MIR6829	1	loss
2905	CGTE_37	3	196669188	196674753	3q29	NCBP2-AS2,PIGZ,NCBP2	0	loss
2906	CGTE_37	4	299052	338213	4p16.3	ZNF141	3	gain
2907	CGTE_37	4	2824596	2835540	4p16.3	SH3BP2	1	loss

2908	CGTE_37	4	68396539	68411251	4q13.2	CENPC	0	loss
2909	CGTE_37	5	37003351	37014908	5p13.2	NIPBL	1	loss
2910	CGTE_37	5	70800444	70808243	5q13.2	BDP1	3	gain
2911	CGTE_37	5	175956083	176008588	5q35.2	CDHR2,RNF44	1	loss
2912	CGTE_37	6	26027069	26240851	6p22.2	HIST1H1E,HIST1H2BB,HIST1H3D,HIST1H2BE,HIST1H3C,HIST1H3B,HIST1H1C,HIST1H3E,HIST1H1T,HIST1H4B,HIST1H4E,HIST1H4D,HIST1H2BD,HIST1H2AD,HIST1H4C,HFE,HIST1H2BC,HIST1H1D,HIST1H2AC,HIST1H2BF,HIST1H2AE,HIST1H2AB,HIST1H4F,HIST1H2BG	3	gain
2913	CGTE_37	6	30994645	30997476	6p21.33	MUC22	4	gain
2914	CGTE_37	6	33131385	33136735	6p21.32	COL11A2	1	loss
2915	CGTE_37	6	126298763	126320800	6q22.32	TRMT11,HINT3	1	loss
2916	CGTE_37	7	73245481	73279795	7q11.23	WBSCR27,WBSCR28,CLDN4	1	loss
2917	CGTE_37	7	100675346	100685166	7q22.1	MUC17	3	gain
2918	CGTE_37	7	149190028	149522466	7q36.1	KRBA1,SSPO,ZNF767P,ZNF746,ZNF467	1	loss
2919	CGTE_37	8	21957266	21976835	8p21.3	NUDT18,FAM160B2,HR	1	loss
2920	CGTE_37	8	143808726	143832748	8q24.3	THEM6,SLURP1,LYPD2	1	loss
2921	CGTE_37	8	144775803	145770785	8q24.3	CYHR1,SLC39A4,MROH1,HGH1,PLEC,MIR6846,MFSD3,HSF1,MAF1,MIR6847,MIR661,FAM83H-AS1,MIR6893,BREA2,EXOSC4,MIR6845,ZNF707,WDR97,SCRT1,BOP1,DGAT1,EPPK1,MIR4664,CPSF1,CCDC166,PPP1R16A,MIR7112,SCX,ARHGAP39,LRRC14,OPLAH,GPT,C8orf82,RECQL4,TONSL,LRRC24,SPATC1,KIFC2,PUF60,SHARPIN,MIR937,FBXL6,PARP10,GPAA1,MAPK15,FOXH1,SLC52A2,SCRIB,MIR939,NRBP2,TONSL-AS1,VPS28,MIR6848,GRINA,FAM83H,ADCK5,MIR6849,TMEM249,CYC1	1	loss
2922	CGTE_37	9	139820118	139983406	9q34.3	PTGDS,LCNL1,UAP1L1,SAPCD2,C8G,CLIC3,MAN1B1-AS1,MAN1B1,ENTPD2,FUT7,ABCA2,LCN12,FBXW5,TRAF2,C9orf139,C9orf142,NPDC1	1	loss
2923	CGTE_37	9	139983411	139997798	9q34.3	MAN1B1	3	gain
2924	CGTE_37	10	43597796	43604660	10q11.21	RET	0	loss
2925	CGTE_37	10	59986854	60029367	10q21.1	IPMK,CISD1	0	loss
2926	CGTE_37	10	72298053	72493560	10q22.1	ADAMTS14,PRF1,PALD1	1	loss
2927	CGTE_37	10	73565497	73573207	10q22.1	CDH23	1	loss
2928	CGTE_37	10	104159961	104162092	10q24.32	NFKB2	0	loss
2929	CGTE_37	10	134008268	134039199	10q26.3	DPYSL4,STK32C	1	loss
2930	CGTE_37	10	135137692	135165001	10q26.3	PRAP1,CALY	1	loss
2931	CGTE_37	11	32636090	32697629	11p13	CCDC73	0	loss
2932	CGTE_37	11	46387777	46398075	11p11.2	DGKZ,MIR4688	1	loss
2933	CGTE_37	12	40876490	40885045	12q12	MUC19	4	gain
2934	CGTE_37	12	57572621	57615163	12q13.3	NXPH4,LRP1,MIR1228	1	loss
2935	CGTE_37	12	110032746	110221650	12q24.11	TRPV4,MVK,FAM222A,FAM222A-AS1	0	loss
2936	CGTE_37	12	123339418	123346372	12q24.31	HIP1R	1	loss
2937	CGTE_37	12	132328501	132404706	12q24.33	ULK1,MMP17	1	loss
2938	CGTE_37	12	133104486	133202381	12q24.33	MIR6763,LRCOL1,P2RX2,FBRSL1,POLE	1	loss
2939	CGTE_37	13	50321059	50467064	13q14.2	CTAGE10P,KPNA3	4	gain
2940	CGTE_37	14	73712217	73735366	14q24.2	PAPLN	1	loss
2941	CGTE_37	14	103523331	103600330	14q32.32	EXOC3L4,CDC42BPB,TNFAIP2	1	loss
2942	CGTE_37	15	20456562	22489980	15q11.2-q11.1	OR4N4,MIR3118-4,MIR3118-3,LOC101927079,MIR5701-2,OR4M2,LOC646214,POTEB,HERC2P3,POTEB3,GOLGA6L6,POTEB2,LOC727924,MIR5701-1,GOLGA8CP,MIR3118-2,MIR5701-3,CXADRP2,NBEAP1,LINC01193,OR4N3P,NF1P2,CHEK2P2	3	gain
2943	CGTE_37	15	75090918	75105399	15q24.1	LMAN1L,CSK	1	loss

2944	CGTE_37	15	76630150	76633639	15q24.3	ISL2	0	loss
2945	CGTE_37	15	90174779	90196148	15q26.1	KIF7	1	loss
2946	CGTE_37	16	309921	1312249	16p13.3	SSTR5,SSTR5-AS1,CACNA1H,MIR3176,FAM195A,TPSAB1,AXIN1,FBXL16,MSLN,WDR24,MRPL28,TPSG1,CAPN15,MIR662,GNG13,NHLRC4,DECR2,RHBDL1,STUB1,C1QTNF8,PRR35,LOC100134368,WDR90,FAM173A,RAB11FIP3,WFIKKN1,ARHGDIG,SOX8,PDIA2,PIGQ,HAGHL,TPSD1,PRR25,RPUSD1,CHTF18,MIR5587,C16orf13,TPSB2,JMJD8,NARFL,LMF1-AS1,RAB40C,FAM234A,LINC00235,RGS11,METRN,RHOT2,CCDC78,LMF1,TMEM8A,NME4	1	loss
2947	CGTE_37	16	4256719	4445424	16p13.3	PAM16,TFAP4,CORO7-PAM16,SRL,GLIS2-AS1,VASN,GLIS2,CORO7,LINC01569	1	loss
2948	CGTE_37	16	67232982	67245101	16q22.1	ELMO3,MIR328,LRRC29	1	loss
2949	CGTE_37	16	88763963	88808622	16q24.3	LOC100289580,CTU2,MIR4722,RNF166,PIEZO1	1	loss
2950	CGTE_37	16	89760538	89784529	16q24.3	CDK10,SPATA2L,VPS9D1-AS1,VPS9D1	1	loss
2951	CGTE_37	18	8824627	8826420	18p11.22	MTCL1	0	loss
2952	CGTE_37	19	51324816	51327088	19q13.33	KLK1	0	loss
2953	CGTE_37	20	3208382	3214283	20p13	SLC4A11	1	loss
2954	CGTE_37	20	57276037	57288771	20q13.32	STX16-NPEPL1,NPEPL1	1	loss
2955	CGTE_37	20	60882313	61150950	20q13.33	CABLES2,C20orf166-AS1,MIR4758,LAMA5,ADRM1,RBBP8NL,LAMA5-AS1,RPS21,MIR1-1HG,GATA5	1	loss
2956	CGTE_37	20	62316696	62332718	20q13.33	RTEL1,RTEL1-TNFRSF6B,ARFRP1,TNFRSF6B	1	loss
2957	CGTE_37	20	62698249	62738309	20q13.33	LKAAEAR1,TCEA2,OPRL1,RGS19,NPBWR2,MIR6813	1	loss
2958	CGTE_37	21	45714266	45755728	21q22.3	AIRE,PFKL,C21orf2	1	loss
2959	CGTE_37	21	45970688	46117586	21q22.3	KRTAP10-5,KRTAP10-6,KRTAP10-11,KRTAP10-12,TSPEAR,KRTAP12-2,KRTAP10-10,KRTAP10-4,KRTAP12-1,KRTAP10-9,KRTAP12-3,KRTAP10-7,KRTAP10-3,KRTAP10-8,KRTAP10-2,KRTAP12-4	1	loss
2960	CGTE_37	21	47531374	47575502	21q22.3	FTCD,COL6A2	1	loss
2961	CGTE_37	X	114413987	114422944	Xq23	LRCH2	0	loss
2962	CGTE_37	X	153677986	153697856	Xq28	PLXNA3,FAM50A,MIR6858	1	loss
2963	CGTE_38	1	861266	1564479	1p36.33	MIR429,DVL1,PLEKHN1,ACAP3,NOC2L,TTLL10,LINC01342,TAS1R3,PERM1,SAMD11,ATAD3A,MIR200B,SCNN1D,TMEM88B,CPSF3L,ATAD3C,ATAD3B,C1orf159,MIR6727,C1orf233,MRPL20,LOC102724312,HES4,AGRN,ISG15,TMEM240,VWA1,RNF223,TNFRSF4,KLHL17,SDF4,CCNL2,MIB2,AURKAIP1,MIR6726,LOC148413,SSU72,ANKRD65,UBE2J2,PUSL1,CPTP,B3GALT6,TNFRSF18,MXRA8,FAM132A,MIR6808,MIR200A	0	loss
2964	CGTE_38	1	2066625	2118703	1p36.33	PRKCZ,FAAP20	32	gain
2965	CGTE_38	1	2121090	3692052	1p36.32-p36.33	MORN1,TTC34,MIR551A,LOC100129534,PANK4,ARHGEF16,RER1,PEX10,MMEL1,MEGF6,TP73,LOC100996583,LINC00982,LOC115110,SMIM1,TNFRSF14,PRDM16,PLCH2,MIR4251,FAM213B,ACTRT2,SKI,CCDC27,FAAP20,WRAP73,HES5,TP73-AS1,TPRG1L	1	loss
2966	CGTE_38	1	152191503	153309975	1q21.3	CRCT1,LCE2D,LCE1A,KPRP,LCE3B,LCE3E,LCE3C,LCE5A,LCE1C,LCE1D,SPRR2C,C1orf68,LCE3D,LCE1B,SPRR2E,CRNN,IVL,SPRR1B,FLG,LCE6A,SPRR2A,LCE1F,LCE4A,PGLYRP4,FLG-AS1,LOR,SPRR2F,SPRR2G,LCE2C,PGLYRP3,SPRR2B,FLG2,PRR9,LELP1,LCE1E,LOC101928009,SPRR2D,LCE3A,SPRR4,HRNR,LCE2B,SPRR1A,LCE2A,SPRR3,SMCP	3	gain

2967	CGTE_38	1	157488484	159558334	1q23.2-q23.1	MNDA,CD5L,OR10J1,APCS,CADM3,PYHIN1,CD1B,FCRL4,OR6N1,OR10X1,OR6Y1,OR10R2,OR6K3,FCRL5,OR6K2,LOC646268,OR10J3,KIRREL,FCER1A,CD1A,ACKR1,OR10J5,AIM2,OR10K1,FCRL3,CD1E,OR6K6,CADM3-AS1,FCRL1,SPTA1,OR10K2,IFI16,OR10Z1,CD1D,OR6P1,OR10T2,FCRL2,OR6N2,CD1C	3	gain
2968	CGTE_38	2	41449	217544070	2q11.1-p14	LINC01122,LOC100507201,TP53I3,MEMO1,MIR6071,POLE4,CAPN14,RRM2,RHOQ,FAM95A,ZRANB3,FAM161A,GGCX,MIR4432,HAGLROS,YIPF4,RBM43,LINC01249,FOXD4L1,GPR45,NOP58,MIR4430,MOB1A,C2orf74,MIR4265,FAR2P2,PPP1R1C,ZC3H6,LOC105373609,NEMP2,MARCH7,GTF3C2,C2orf16,LOC389033,CYP1B1,LOC100996579,SF3B1,LOC101927948,C2orf68,LOC400997,RAB6C-AS1,POTEF,MPHOSPH10,RGPD2,LOC101927055,COA5,CLIP4,HSPE1,ANXA4,IL1RN,MIR4776-1,RND3,KIAA1841,MAP2,ABCG8,GORASP2,SNORD89,REV1,LOC101929723,LINC01123,LOC151121,MIR217,DPP4,KANSL3,INO80D,TMEM17,C2orf40,SLC8A1-AS1,GCFC2,ACTR1B,MIR4435-1,BCL2L11,WDR43,PDK1,FANCL,LOC285074,LOC644838,ZNF806,STAT1,UNC80,LIMS3,MPV17,PRR30,RPE,LOC654342,SNORD132,CYS1,LINC00309,RNF144A-AS1,FAM228B,AMMECR1L,CCDC148-AS1,UBE2E3,LOC100506124,SCN9A,AOX1,MIR1245A,ALK,LOC102723824,POTEI,DNAJC27,C2orf70,CENPO,MIR3127,SNTG2,LOC100132215,UPP2,DARS-AS1,MARCH4,BBS5,CNGA3,MALL,GPAT2,LINC00954,ST3GAL5,MDH1B,HSPE1-MOB4,XIRP2-AS1,SPATS2L,GCA,LINC01159,ERMN,KCMF1,LOC101927406,TTC32,NRP2,CD8A,LINC01105,ELMOD3,LOC284950,TMEM163,FRZB,MIR3130-1,CLASP1,LINC01118,MIR3681,MRPL19,THADA,PSMD14,IWS1,LINC01090,MIR4784,TMEM182,LOC101929715,TGOLN2,PLEKHA3,SPOPL,PTCD3,TTC27,MIR4436B1,ADGRF3,KCNH7,PNPT1,PAPOLG,KCNK12,RNF181,LOC100507140,PPP1CB,FBXO41,SUPT7L,RIF1,DYNC2LI1,NXPH2,PDCL3,LOC100507006,LOC101060091,PLGLB1,GLI2,MIR216A,LINC00607,BCL11A,ADAM17,TSPYL6,ACTR2,ARHGAP25,MEIS1,STON1,MATN3,MRPS5,RAB3GAP1,PPIL3,PCBP1-AS1,THNSL2,LOC101927285,LOC440910,PGM5P3-AS1,LINC01318,SNORA70B,ATF2,RGPD3,HNMT,WDR75,R3HDM1,SIX3,MIR4434,FKBP7,POLR2D,ORC2,MDH1,MIR1258,LINC01628,UGGT1,C2orf66,LOC101929282,TRMT61B,USP39,SOCS5,VIT,LOC646743,RMDN2,LOC101929633,SRBD1,KLHL29,THUMPD2,AFTPH,SIX3-AS1,KCNJ3,HOXD4,MIR4773-1,MIR559,SMYD5,LOC100288570,SLC25A12,RPL23AP32,LOC101927043,TMEM177,ACVR1,GCKR,ICA1L,GPR75,REG1A,LOC101927865,ATP6V1C2,EN1,GRB14,NOSTRIN,PKP4,EPT1,LOC101927053,CIR1,VWA3B,GULP1,UTAT33,RBMS1,DCDC2C,LCT,CASP8,CYP27C1,NCKAP5,CYBRD1,GRHL1,ERCC3,AGBL5,LINC01127,SLC35F5,CREB1,RAPGEF4-AS1,CDC42EP3,ALMS1-IT1,STK39,TRIM54,LOC101927907,REG1B,LOC728730,EIF2B4,MIR7845,FAHD2A,TISP43,PELI1,RHOB,RMDN2-AS1,PROM2,TPO,DQX1,ALS2CR11,GPR75-ASB3,NABP1,RPS7,AGPS,ST6GAL2,DYSF,MIR548BA,SFXN5,NIFK,RBM45,LINC01291,NEURL3,LHCGR,NAGK,PFN4,SSB,MRPL30,MTX2,LINC00486,PTH2R,ADCY3,MIR4444-1,ACP1,ORMDL1,ATRAID,LOC339807,LINC01125,ZNF804A,GKN1,PCGEM1,TMEM214,SNORD92,C2orf27B,DCAF17,APOB,LOC101928103,CHAC2,SEMA4F,KCTD18,LINC00487,LINC01317,LOC440895,NIF3L1,MIR4772,CKAP2L,MTA3,SCN1A,CXCR4,PMS1,PTPN18,FAM150B,FAM98A,SIX2,NCK2,IL36G,CFC1B,SUMO1,TANK,MIR4444-2,MIR1245B,SPAST,EVA1A,TUBA3E,POTEI,TRIM43B,CTLA4,DNAH6,LOC101929532,LOC1	3	gain
2969	CGTE_38	2	220402668	220427428	2q35	ASIC4,TMEM198,MIR3132,CHPF,OBSL1	0	loss

2970	CGTE_38	2	220428092	239005541	2q37.1-q36.3	GIGYF2,SPP2,NPPC,SCARNA6,UGT1A8,SLC4A3,MIR4439,KCNJ13,PRLH,DIS3L2,HTR2B,ALPPL2,OBSL1,DOCK10,UBE2F-SCLY,PTMA,UGT1A1,MIR1244-1,UGT1A4,SNORD20,ECEL1P2,WDFY1,NCL,CAB39,AP1S3,RAB17,C2orf72,LOC151484,MIR1244-3,UGT1A10,CCDC140,SNORD82,UGT1A6,MFF,AGAP1-IT1,NMUR1,COPS8,KCNE4,IQCA1,MIR4777,LINC00471,ACKR3,SAG,SERPINE2,SPATA3-AS1,LOC646736,IRS1,SGPP2,PDE6D,SLC19A3,MROH2A,SP140L,CUL3,PRSS56,NYAP2,ECEL1,SP110,PSMD1,MIR1471,B3GNT7,LINC01173,RAMP1,INPP5D,CHRNG,UGT1A9,ALPP,RHBDD1,UBE2F,MIR1244-4,TRPM8,LOC100286922,MIR6811,MIR5001,COL4A4,DGKD,MIR5703,MIR1244-2,SP140,MIR4268,NEU2,PAX3,MRPL44,LOC654841,PID1,ITM2C,EFHD1,TM4SF20,DAW1,C2orf57,CHRND,COPS7B,INHA,ALPI,AGAP1,COL6A3,SCARNA5,DNAJB3,SCG2,CCL20,MOGAT1,SLC16A14,STK11IP,FAM124B,ASB18,HJURP,MIR5702,RBM44,ARL4C,MLPH,TIGD1,DNER,AGFG1,SNORA75,UGT1A7,FBXO36,SCLY,LRRFIP1,LOC151475,USP40,UGT1A5,ATG16L1,NGEF,C2orf82,GBX2,SH3BP4,SP100,C2orf83,GPR55,EPHA4,EIF4E2,TRIP12,MSL3P1,SPATA3,ARMC9,FARSB,UGT1A3,ACSL3,SPHKAP,COL4A3	3	gain
2971	CGTE_38	2	239006590	239059847	2q37.3	KLHL30,UBE2F-SCLY,SCLY,ESPNL	0	loss
2972	CGTE_38	4	493077	1732469	4p16.3	CTBP1,MAEA,PCGF3,MYL5,FAM53A,LOC100130872,TMEM129,FGFRL1,RNF212,ATP5I,CPLX1,GAK,IDUA,PDE6B,DGKQ,CRIPAK,SPON2,LOC100129917,SLBP,PIGG,TACC3,SLC26A1,TMED11P,MFSD7,TMEM175,NKX1-1,CTBP1-AS,UVSSA,CTBP1-AS2	1	loss
2973	CGTE_38	4	1732597	1737105	4p16.3	TACC3	5	gain
2974	CGTE_38	4	1737384	1809470	4p16.3	FGFR3,TACC3	0	loss
2975	CGTE_38	4	3432335	3450074	4p16.3	HGFAC,RGS12	0	loss
2976	CGTE_38	4	8582721	8608625	4p16.1	CPZ,GPR78	0	loss
2977	CGTE_38	5	477376	3600446	5p15.33	TERT,LINC01017,CLPTM1L,IRX4,CTD-2194D22.4,LOC100506688,MIR6075,TPPP,SLC6A19,IRX1,BRD9,IRX2,NDUFS6,C5orf38,MRPL36,SLC9A3,CEP72,LPCAT1,TRIP13,MIR4457,MIR4277,SLC6A3,LINC01019,SLC12A7,MIR4635,LOC728613,MIR4456,LOC101929034,LINC01377,SDHAP3,LOC100996325,SLC6A18,LOC100506858,CTD-3080P12.3,ZDHHC11,NKD2,LOC100288152,LINC01511	1	loss
2978	CGTE_38	5	125906198	126141447	5q23.2	ALDH7A1,TEX43,PHAX,LMNB1	1	loss
2979	CGTE_38	5	126145835	130867922	5q31.1-q23.3	SLC27A6,MIR4633,MARCH3,CTXN3,LMNB1,RAPGEF6,CDC42SE2,LINC01184,SLC12A2,HINT1,ISOC1,KIAA1024L,C5orf63,MIR4460,LYRM7,MEGF10,PRRC1,FBN2,ADAMTS19-AS1,CHSY3,ADAMTS19	3	gain
2980	CGTE_38	5	140175794	140257143	5q31.3	PCDHA11,PCDHA4,PCDHA9,PCDHA10,PCDHA5,PCDHA3,PCDHA1,PCDHA6,PCDHA12,PCDHA2,PCDHA7,PCDHA8	0	loss
2981	CGTE_38	5	176859739	176860869	5q35.3	GRK6	5	gain
2982	CGTE_38	5	176860965	176936797	5q35.3	PRR7-AS1,DBN1,DOK3,PDLIM7,PRR7,GRK6	0	loss
2983	CGTE_38	6	25515845	26108369	6p22.2	HIST1H2BB,HIST1H4A,HIST1H4C,HIST1H2APS1,HIST1H4B,HIST1H3A,HIST1H1A,SLC17A1,HIST1H2AB,SLC17A3,HIST1H3B,HFE,HIST1H1C,HIST1H1T,HIST1H3C,TRIM38,SLC17A2,SCGN,SLC17A4,HIST1H2AA,LRRC16A,HIST1H2BA	3	gain
2984	CGTE_38	6	26123839	26273561	6p22.2	HIST1H4G,HIST1H3D,HIST1H2BH,HIST1H3G,HIST1H2BC,HIST1H2BE,HIST1H3E,HIST1H2AE,HIST1H3F,HIST1H4D,HIST1H1D,HIST1H4E,HIST1H2BD,HIST1H2AD,HIST1H2BG,HIST1H2BF,HIST1H4F,HIST1H2AC,HIST1H1E,HIST1H2BI	4	gain
2985	CGTE_38	6	26285443	27215913	6p22.1-p22.2	MIR3143,BTN2A1,BTN2A2,GUSBP2,HCG11,BTN3A3,ABT1,BTN2A3P,HIST1H2AG,PRSS16,HIST1H4I,HIST1H2BJ,LINC00240,HIST1H2AH,LOC100270746,HIST1H2BK,ZNF322,LOC285819,BTN3A1,HMGN4,HIST1H4H,BTN3A2,BTN1A1	3	gain
2986	CGTE_38	6	33131385	33132190	6p21.32	COL11A2	6	gain

2987	CGTE_38	6	33416529	33419732	6p21.32	SYNGAP1	32	gain
2988	CGTE_38	6	33650996	33653610	6p21.31	ITPR3	29	gain
2989	CGTE_38	6	41554683	41652981	6p21.1	FOXP4,MDFI,MIR4641,TFEB	0	loss
2990	CGTE_38	6	128840188	129204607	6q22.33	LAMA2,PTPRK	16	gain
2991	CGTE_38	6	134373515	134436545	6q23.2	SLC2A12,HMGA1P7	9	gain
2992	CGTE_38	7	80374232	86781691	7q21.11-q21.12	SEMA3A,KIAA1324L,GRM3,SEMA3E,LOC101927378,SEMA3C,LOC100128317,DMTF1,PCLO,HGF,CACNA2D1,SEMA3D,LOC101927356	3	gain
2993	CGTE_38	7	149497396	149521882	7q36.1	SSPO	0	loss
2994	CGTE_38	7	149521925	149523373	7q36.1	SSPO	10	gain
2995	CGTE_38	7	154861952	154876068	7q36.2	HTR5A-AS1,HTR5A	10	gain
2996	CGTE_38	8	75937624	76459911	8q21.11	CRISPLD1,CASC9,HNF4G	5	gain
2997	CGTE_38	8	143425279	143763559	8q24.3	JRK,PSCA,ADGRB1,ARC,TSNARE1	0	loss
2998	CGTE_38	8	143763564	143832748	8q24.3	LYPD2,LOC100288181,LY6K,PSCA,SLURP1,THEM6	5	gain
2999	CGTE_38	8	143833743	143867988	8q24.3	LYNX1,LY6D,LYPD2	0	loss
3000	CGTE_38	8	144240163	145009356	8q24.3	MAPK15,RHPN1-AS1,ZNF696,MIR6845,GSDMD,NRBP2,CCDC166,FAM83H,GPIHBP1,MIR937,PUF60,ZNF623,RHPN1,LY6H,MAFA,EPPK1,MAFA-AS1,TIGD5,BREA2,MROH6,PLEC,GLI4,ZC3H3,MIR4664,MINCR,PYCRL,TSTA3,NAPRT,ZNF707,FAM83H-AS1,TOP1MT,SCRIB,ZFP41,EEF1D	0	loss
3001	CGTE_38	8	145009357	145012485	8q24.3	PLEC	6	gain
3002	CGTE_38	8	145012533	145139129	8q24.3	MIR6847,MIR6846,GPAA1,PARP10,OPLAH,PLEC,MIR661,SPATC1,EXOSC4,GRINA	0	loss
3003	CGTE_38	8	145139198	145140502	8q24.3	GPAA1	7	gain
3004	CGTE_38	8	145140564	145618711	8q24.3	SCX,CYC1,FBXL6,CPSF1,GPAA1,BOP1,SCRT1,SLC52A2,MIR7112,MIR6848,MAF1,DGAT1,SHARPIN,HGH1,TMEM249,HSF1,MROH1,ADCK5,WDR97	1	loss
3005	CGTE_38	8	145618716	145739940	8q24.3	TONSL-AS1,PPP1R16A,CPSF1,TONSL,MFSD3,MIR939,VPS28,GPT,MIR6849,KIFC2,MIR6893,RECQL4,SLC39A4,FOXH1,CYHR1	0	loss
3006	CGTE_38	9	139378808	140389610	9q34.3	TOR4A,LOC100128593,GRIN1,MIR6722,C9orf163,EGFL7,TMEM141,LCN6,ENTPD8,LCN15,FUT7,TUBB4B,CYSRT1,SNHG7,NSMF,FAM69B,NRARP,PNPLA7,LRRC26,UAP1L1,MIR3621,CCDC183,ABCA2,TMEM210,NELFB,C9orf172,C9orf173-AS1,MIR4674,RNF224,SSNA1,NPDC1,MIR4292,FBXW5,SNORA17A,C9orf139,C8G,MIR7114,C9orf173,LCN8,NOTCH1,DPP7,RABL6,EXD3,AGPAT2,ANAPC2,MIR4479,TMEM203,RNF208,NOXA1,SLC34A3,CCDC183-AS1,C9orf142,ENTPD2,EDF1,MIR4673,PHPT1,MAMDC4,MAN1B1,TRAF2,MAN1B1-AS1,MIR126,FAM166A,SAPCD2,NALT1,NDOR1,LCN12,LCN10,LCNL1,TPRN,SNORA17B,PTGDS,CLIC3	0	loss
3007	CGTE_38	10	52002917	60124704	10q11.23-q21.1	ASAH2B,MIR605,SGMS1,A1CF,IPMK,UBE2D1,MBL2,PRKG1-AS1,LINC01468,LOC102724719,DKK1,MIR3924,CSTF2T,ZWINT,MTRNR2L5,PCDH15,ASAH2,SGMS1-AS1,PRKG1,CISD1	3	gain
3008	CGTE_38	10	60127633	60145411	10q21.1	TFAM,UBE2D1	6	gain
3009	CGTE_38	10	118030298	118138134	10q25.3	CCDC172,GFRA1	5	gain
3010	CGTE_38	10	131675899	135381428	10q26.3	PAOX,C10orf91,BNIP3,INPP5A,TUBGCP2,TCERG1L,UTF1,CTAGE7P,TCERG1L-AS1,MIR202HG,SPRN,KNDC1,MIR202,PPP2R2D,SPRNP1,PWWP2B,LRRC27,ECHS1,ADGRA1-AS1,ZNF511,DPYSL4,FUOM,LINC01168,MIR3944,JAKMIP3,ADGRA1,LINC00959,MTG1,CALY,CFAP46,GLRX3,STK32C,SCART1,CYP2E1,SYCE1,ADAM8,LINC01167,EBF3,PRAP1,MIR378C,VENTX,LINC01166,LINC01164,NKX6-2	1	loss

3011	CGTE_38	11	1023920	1080490	11p15.5	MUC2,MUC6	0	loss
3012	CGTE_38	11	1080494	1081152	11p15.5	MUC2	10	gain
3013	CGTE_38	11	1081415	3249981	11p15.5-p15.4	LSP1,TNNT3,TOLLIP,TRPM5,BRSK2,MRPL23,LINC01150,MUC5AC,TOLLIP-AS1,SYT8,C11orf21,KCNQ1DN,MIR675,NAP1L4,MIR4298,IGF2,OSBPL5,MIR7847,KRTAP5-6,CD81,KRTAP5-5,FAM99A,CD81-AS1,DUSP8,TSPAN32,MIR483,IGF2-AS,KRTAP5-AS1,ASCL2,MIR6744,KRTAP5-4,MUC5B,HOTS,KCNQ1OT1,FAM99B,MIR4686,MUC2,SLC22A18,MRPL23-AS1,SLC22A18AS,TNNI2,CTSD,LINC01219,MOB2,IFITM10,TSSC4,MRGPRG,CDKN1C,TH,H19,KRTAP5-3,INS,KRTAP5-2,SNORA54,SNORD131,MRGPRE,MRGPRG-AS1,KCNQ1,PHLDA2,KCNQ1-AS1,CARS,KRTAP5-1,INS-IGF2	1	loss
3014	CGTE_38	11	4730882	6341077	11p15.4	OR51V1,OR52R1,OR51G2,BGLT3,OR56A1,OR52W1,OLFM5P,HBG2,OR52N5,OR51L1,CNGA4,OR51I1,OR51A7,OR51F1,OR51B4,TRIM34,OR56B1,OR51I2,OR52E2,OR52N4,OR51F2,OR52A1,PRKCDBP,TRIM6,OR52B2,OR52J3,OR52A5,HBBP1,OR56B4,OR51S1,OR56A3,OR52N2,C11orf42,OR52D1,OR52L1,CCKBR,OR51A2,HBE1,OR51G1,UBQLNL,OR52N1,OR51Q1,HBD,OR51M1,OR52E6,HBG1,OR51B5,OR52B6,TRIM5,OR56A5,TRIM6-TRIM34,OR52H1,UBQLN3,FAM160A2,OR51A4,TRIM22,OR52E4,HBB,OR56A4,OR51T1,OR51B2,OR51B6,MMP26,OR52E8	3	gain
3015	CGTE_38	11	57799116	58377388	11q12.1	OR1S2,OR5B2,OR9Q2,OR10Q1,ZFP91,OR5B21,OR5B3,OR1S1,OR10W1,ZFP91-CNTF,LPXN,OR6Q1,OR5B12,OR5B17,OR9I1,OR9Q1	3	gain
3016	CGTE_38	11	64083180	64139065	11q13.1	ESRRA,TRMT112,CCDC88B,PRDX5,MIR1237,RPS6KA4,MIR7155	0	loss
3017	CGTE_38	11	64216947	64323730	11q13.1	LOC100996455,SLC22A11	7	gain
3018	CGTE_38	11	67184774	68154169	11q13.2	NDUFV1,C11orf24,KMT5B,TCIRG1,AIP,UNC93B1,ACY3,MIR6753,CABP4,RPS6KB2,TBX10,PTPRCAP,CARNS1,CDK2AP2,DOC2GP,NDUFS8,CHKA,PITPNM1,FAM86C2P,NUDT8,C11orf72,CORO1B,GPR152,ALDH3B1,GSTP1,TMEM134,CABP2,LRP5,MIR4691,MIR6752,MIR7113,ALDH3B2	1	loss
3019	CGTE_38	11	68157351	68214059	11q13.2	LRP5	3	gain
3020	CGTE_38	12	7343745	7655405	12p13.31	ACSM4,CD163,CD163L1,PEX5	3	gain
3021	CGTE_38	13	25459690	25463557	13q12.12	CENPJ	35	gain
3022	CGTE_38	13	45556210	45563877	13q14.12	NUFIP1,GPALPP1	7	gain
3023	CGTE_38	13	46357511	46358239	13q14.13	SIAH3	6	gain
3024	CGTE_38	14	21971280	22772042	14q11.2	SALL2,OR10G3,OR4E1,METTL3,OR4E2,OR10G2	3	gain
3025	CGTE_38	14	24911249	29237307	14q12	MIR4307,MIR4307HG,CTSG,MIR3171,SDR39U1,STXBP6,GZMH,NOVA1,FOXG1,LOC101927045,LINC00645,LOC102724890,FOXG1-AS1,LOC101927062,GZMB,CMA1	3	gain
3026	CGTE_38	14	50930686	51027123	14q22.1	ATL1,MAP4K5	5	gain
3027	CGTE_38	14	99640502	99697954	14q32.2	BCL11B	0	loss
3028	CGTE_38	14	103576202	103596472	14q32.32	TNFAIP2,EXOC3L4	5	gain
3029	CGTE_38	14	104552082	105363146	14q32.33	MIR4710,MIR203B,AKT1,LINC00638,ADSSL1,SIVA1,CEP170B,C14orf180,ZBTB42,INF2,MIR203A,KIF26A,TMEM179,ASPG	0	loss
3030	CGTE_38	14	105393282	105412546	14q32.33	AHNAK2,PLD4	1	loss
3031	CGTE_38	14	105412779	105418727	14q32.33	AHNAK2	4	gain
3032	CGTE_38	14	105418796	106330007	14q32.33	MIR4507,MIR6765,AHNAK2,CRIP2,C14orf80,PACS2,CRIP1,MIR8071-1,MIR4537,NUDT14,ELK2AP,BTBD6,MIR4539,MTA1,MIR8071-2,GPR132,LOC102723354,C14orf79,TMEM121,LOC100507437,TEX22,MIR4538,BRF1,JAG2,CDCA4	1	loss

3033	CGTE_38	15	20170004	22489980	15q11.1-q11.2	LOC101927079,OR4M2,MIR5701-2,MIR3118-2,MIR3118-3,OR4N4,POTEB3,LINC01193,MIR3118-4,POTEB,CHEK2P2,LOC727924,HERC2P3,NF1P2,MIR5701-3,CXADRP2,LOC646214,OR4N3P,MIR5701-1,POTEB2,NBEAP1,GOLGA8CP,GOLGA6L6	3	gain
3034	CGTE_38	15	42143069	42164751	15q15.1	SPTBN5,MIR4310	0	loss
3035	CGTE_38	15	65828077	66857701	15q22.31	HACD3,MIR4512,VWA9,SLC24A1,MIR4311,SCARNA14,DIS3L,SNORD18A,MAP2K1,SNAPC5,TIPIN,LCTL,MIR4511,RPL4,SNORD18B,MEGF11,DENND4A,ZWILCH,SNORD16,RAB11A,SNORD18C	3	gain
3036	CGTE_38	16	97450	920066	16p13.3	AXIN1,HBZ,NPRL3,PDIA2,WFIKKN1,HBQ1,MIR5587,SNRNP25,RPUSD1,PRR35,C16orf13,PRR25,RHBDL1,FAM173A,LINC00235,CHTF18,MRPL28,RHBDF1,MSLN,RHOT2,ARHGDIG,METRN,RAB40C,MIR662,HBA1,FAM234A,CAPN15,CCDC78,HBA2,JMJD8,NHLRC4,PIGQ,TMEM8A,MPG,FBXL16,POLR3K,RGS11,STUB1,WDR24,LUC7L,LMF1,GNG13,HBM,WDR90,DECR2,LOC100134368,HAGHL,MIR3176,FAM195A,RAB11FIP3,NME4,NARFL	1	loss
3037	CGTE_38	16	920676	943180	16p13.3	LMF1	9	gain
3038	CGTE_38	16	943201	2328107	16p13.3	SPSB3,TPSAB1,NDUFB10,RNPS1,BAIAP3,SNHG9,NOXO1,IGFALS,MRPS34,HAGH,C1QTNF8,ABCA3,SLC9A3R2,GFER,MIR1225,GNPTG,RNF151,SNORA78,TPSB2,BRICD5,RPL3L,CASKIN1,CCDC154,RPS2,PGP,TELO2,NUBP2,CRAMP1,SOX8,E4F1,MLST8,MIR6511B1,PKD1,SNORA10,SSTR5-AS1,MIR3677,NTHL1,CACNA1H,MIR4717,NPW,UNKL,ECI1,SNORD60,IFT140,TPSD1,TMEM204,TPSG1,NME3,SNORA64,MIR4516,MSRB1,EME2,MIR3177,TSC2,MIR6511B2,LMF1-AS1,RAB26,TSR3,SYNGR3,CLCN7,C16orf91,MIR940,MEIOB,LOC106660606,SSTR5,TBL3,FAHD1,DNASE1L2,MAPK8IP3,LMF1,SNHG19,LINC00254,PTX4,UBE2I,HS3ST6,TRAF7,HN1L,MIR3180-5,ZNF598	1	loss
3039	CGTE_38	16	85741580	85768895	16q24.1	C16orf74	8	gain
3040	CGTE_38	17	38801891	39637322	17q21.2	KRTAP9-8,KRTAP9-3,KRTAP2-3,KRTAP4-3,KRT40,TMEM99,KRT37,KRT38,KRT27,KRTAP9-1,KRTAP4-5,KRTAP9-2,KRT20,KRT28,KRTAP4-4,KRT35,KRT12,KRTAP9-4,KRT34,LOC100505782,KRTAP4-8,KRT23,KRTAP3-1,KRTAP1-1,KRT33A,KRTAP4-12,SMARCE1,KRTAP16-1,KRTAP4-6,KRT32,KRT26,KRTAP2-4,KRTAP4-7,KRTAP4-1,KRTAP9-6,KRT222,KRTAP1-3,KRTAP3-3,KRTAP3-2,KRT24,KRTAP9-7,KRT33B,KRTAP17-1,KRT10,KRTAP4-2,KRTAP2-1,KRTAP2-2,KRTAP1-5,KRTAP29-1,KRTAP4-9,KRT25,KRTAP1-4,KRTAP4-11,KRTAP9-9,KRT31,KRT39	3	gain
3041	CGTE_38	17	77078856	77080160	17q25.3	ENGASE	6	gain
3042	CGTE_38	18	29848070	32621646	18q12.1	KLHL14,WBP11P1,MAPRE2,GAREM1,CCDC178,DTNA,NOL4,ASXL3	3	gain
3043	CGTE_38	18	67872791	67993430	18q22.2	SOCS6,RTTN	4	gain

3044	CGTE_38	19	281411	4210456	19p13.3	DOT1L,ABHD17A,MIR1909,THEG,TCF3,MIDN,BSG,MED16,JSRP1,C19orf25,LINGO3,DAPK3,SIRT6,ZFR2,OAZ1,PRTN3,APBA3,AP3D1,CIRBP,CSNK1G2-AS1,C19orf35,ZNF554,LOC100996351,MISP,REXO1,THOP1,TMEM259,C2CD4C,MADCAM1,RPS15,C19orf24,MIR1268A,ZBTB7A,GZMM,ATCAY,CDC34,RNU6-2,CIRBP-AS1,NDUFS7,ZNF556,GNA11,MIR637,MIR7108,ABCA7,HCN2,HMG20B,R3HDM4,LOC100288123,RNF126,SMIM24,GAMT,CBARP,MFSD12,GIPC3,MBD3,MIER2,CFD,SPPL2B,GNG7,PIP5K1C,GNA15,FZR1,TBXA2R,CNN2,POLR2E,UQCR11,AZU1,FGF22,TLE6,TMPRSS9,S1PR4,PTBP1,NFIC,HMHA1,MEX3D,POLRMT,PLPP2,MKNK2,DIRAS1,GPX4,NMRK2,REEP6,CREB3L3,CACTIN-AS1,BTBD2,CELF5,MOB3A,CSNK1G2,SBNO2,PIAS4,RAX2,MRPL54,APC2,MAP2K2,STK11,MIR6789,TLE2,ELANE,GRIN3B,ZNF555,CACTIN,ONECUT3,AES,PLPPR3,PCSK4,TJP3,SGTA,LMNB2,LSM7,NCLN,ATP8B3,TIMM13,PLEKHJ1,ATP5D,KLF16,MIR7850,KISS1R,PALM,SCAMP4,MIR1227,DOHH,MIR4321,ZNF77,ARID3A,IZUMO4,EFNA2,FSTL3,AMH,PRSS57,MATK,C19orf71,ADAT3,ANKRD24,MUM1,MIR3187,ZNF57,SLC39A3,TPGS1,SHC2,ODF3L2,PLK5,DAZAP1,WDR18,ADAMTSL5,GADD45B,MIR4745,EEF2,SF3A2,SNORD37	1	loss

3045	CGTE_38	19	24269970	42754849	19q13.12-q13.13	CD22,B3GNT8,ZNF383,SELV,LOC728752,LYPD4,MED29,HPN,ZNF780A,ZFP36,ZNF345,IGFLR1,GAPDHS,OVOL3,RAB4B-EGLN2,ATP1A3,ZNF780B,PLD3,HIPK4,LINC01480,ZNF565,MIR4323,ZFP30,LINC00665,LOC102724958,AKT2,LOC102723617,GSK3A,NCCRP1,HPN-AS1,LGALS4,IFNL3,DYRK1B,NFKBID,DPF1,MIA,LOC400684,LOC100505622,RGS9BP,NPHS1,BLVRB,LINC00662,ZNF30-AS1,CYP2G1P,CYP2B6,TMEM91,EIF3K,GRAMD1A,ZNF571,ACTN4,PLEKHG2,FBXO17,EXOSC5,KCTD15,ZNF382,DEDD2,TDRD12,NFKBIB,GPATCH1,ZNF567,SNRPA,CYP2A6,FBL,SPINT2,RYR1,KIRREL2,ZNF574,CEACAM6,IFNL2,CLC,PRX,PPP1R14A,TTC9B,HAVCR1P1,TBCB,CEACAM7,ZNF571-AS1,MIR6887,ECH1,LINC00906,CNTD2,LINC01532,UBA2,HCST,HNRNPL,SLC7A10,CCER2,FBXO27,PLEKHF1,ZFP14,ZNF573,ZNF526,MIA-RAB4B,ZNF792,LOC100631378,APLP1,PDCD2L,LSR,ZNF569,ZNF607,ZBTB32,SUPT5H,RPS19,ZNF790-AS1,SYCN,FCGBP,ZNF829,ALKBH6,CEBPG,DLL3,ADCK4,SDHAF1,PCAT19,THEG5,PROSER3,LINC01533,EID2,SPTBN4,PAF1,FAM98C,LGALS14,HNRNPUL1,LINC01529,ZNF30,LINC01534,ZNF585B,POU2F2,ERF,SERTAD3,COX7A1,GPI,MIR6719,SCGB2B3P,SAMD4B,ZNF793,MIR641,LOC100134317,LIN37,CLIP3,ZNF793-AS1,MRPS12,SHKBP1,MIR6796,LRFN3,SCGB1B2P,LINC01531,ZNF181,TIMM50,GGN,PSENEN,LOC400685,C19orf47,FXYD5,LOC644189,ARHGEF1,CAPN12,CD79A,ZNF568,GRIK5,TMEM147-AS1,LGALS16,LOC101927151,CEACAM5,ARHGAP33,LOC100420587,HKR1,LSM14A,ITPKC,SBSN,C19orf12,EGLN2,RPS16,DMKN,RBM42,WTIP,ZNF850,ZNF790,ZNF529-AS1,KIAA0355,ATP5SL,LGI4,LOC284412,PSMD8,ZNF260,PEPD,ZNF146,TMEM147,MAP4K1,TGFB1,FXYD7,LOC284395,WDR87,ZNF254,ZNF302,YIF1B,KCNK6,B9D2,LOC728485,PDCD5,POP4,ZNF546,URI1,RAB4B,CEP89,LEUTX,LTBP4,C19orf54,LOC101927411,ETV2,CYP2B7P,ZNF585A,MIR5196,SLC7A9,CHST8,RASGRP4,MIR4530,USF2,PSMC4,COX6B1,DMRTC2,WDR62,CYP2A13,LGALS7,DPY19L3,U2AF1L4,ANKRD27,ZFP82,CEBPA,ZNF570,LOC644554,FAAP24,CCDC97,IFNL4,RHPN2,ZNF529,MIR6797,SPRED3,IFNL1,KRTDAP,MAG,ERICH4,EID2B,WDR88,HAUS5,SCN1B,POLR2I,FFAR1,RABAC1,CCNE1,NUMBL,CEACAM21,PRODH2,UQCRFS1,LOC101927572,UPK1A-AS1,ZNF566,FAM187B,TSHZ3,RINL,ZNF420,LRFN1,LGALS17A,SARS2,VSTM2B,LINC01535,MAP3K10,CYP2A7,LGALS13,CEBPA-AS1,FXYD3,ATP4A,SIRT2,SIPA1L3,ZNF599,LOC100129935,FFAR3,TYROBP,LGALS7B,BCKDHA,ZNF461,ACP7,THAP8,HSPB6,UPK1A,LINC00904,ZNF527,CYP2S1,SCGB2B2,C19orf33,ZNF536,CATSPERG,FXYD1,KMT2B,FFAR2,CEACAM3,HAMP,ZNF540,CEACAM4,CYP2F1,NUDT19,SERTAD1,LRP3,AXL,CAPNS1,SYNE4,PAK4,ZNF507,ZNF781,GMFG	4	gain
3046	CGTE_38	19	42758982	42928483	19q13.2	LIPE,PRR19,CNFN,MEGF8,ERF,CIC,TMEM145,MIR8077,PAFAH1B3,LOC101930071,LIPE-AS1	3	gain
3047	CGTE_38	19	42928488	45314763	19q13.32-q13.31	PSG2,PSG10P,ZNF233,PSG3,ZNF226,PSG11,PSG7,LOC100289650,PRG1,PLAUR,BCAM,IRGC,PSG6,SMG9,ZNF112,ZNF576,ZNF45,LYPD3,CXCL17,IRGQ,CEACAM1,CEACAM8,MIR4531,PINLYP,TEX101,ZNF234,PSG9,LIPE-AS1,CEACAM22P,ZNF284,CD177,MIR8085,LYPD5,ZNF428,LIPE,SRRM5,ZNF283,PHLDB3,LOC100379224,KCNN4,ZNF235,ZNF575,ZNF221,PSG5,IGSF23,ZNF225,PVR,ZNF230,BCL3,CADM4,CEACAM16,CEACAM19,ZNF227,ZNF180,PSG8,CBLC,ZNF229,LOC101928063,ZNF404,LOC100505715,LOC284344,ZNF224,ZNF285,ZNF222,PSG4,CEACAM20,PSG1,XRCC1,ETHE1,ZNF155,ZNF223	5	gain

3048	CGTE_38	19	45315469	55543993	19q13.33-q13.32	C19orf48,SIGLEC8,TRPM4,VRK3,ZNF808,PPP1R37,MIR518F,SAE1,SNAR-G1,SIGLEC17P,MED25,TARM1,ZNF611,ASPDH,TULP2,IZUMO2,C19orf81,NLRP7,MIR1283-1,DPRX,SIGLEC12,PPP5D1,ZNF350,MIR517C,KLK12,LILRB1,GLTSCR1,PTGIR,MYADM,SYNGR4,MIR516B1,ZNF677,TMC4,SNAR-A10,CGB1,LHB,GPR32,CALM3,MYBPC2,ZNF836,CARD8-AS1,CKM,KLK10,MAMSTR,ZNF880,ZNF888,MYH14,MIR519A1,SIGLEC16,KLK8,MIR4324,DBP,CCDC9,GRWD1,AP2A1,SNORD34,KIR2DL1,NUCB1-AS1,TOMM40,HRC,EHD2,MIR935,MIR518A2,DACT3,GPR4,FOSB,LOC105372441,ZNF347,LILRA2,NANOS2,FCGRT,MIR372,GNG8,MIR6088,EML2-AS1,FTL,ZNF175,SYT3,DHX34,ERCC2,SNORD35B,SNAR-C4,ZNF321P,ZNF665,MIR371B,PPP2R1A,SNAR-A9,SNAR-B2,NAPA,KPTN,GIPR,SNAR-C3,CGB,FBXO46,VN1R2,NR1H2,CACNG8,EML2,PIH1D1,MIRLET7E,SULT2A1,SIGLEC10,TPM3P9,MIR8061,LAIR2,LOC105447645,KLK2,DMPK,INAFM1,FGF21,KIR3DL1,APOC4,PLA2G4C-AS1,NDUFA3,PLEKHA4,NTF4,EMP3,MIR512-2,DKFZp434J0226,AP2S1,MIR520F,CTU1,MIR520H,C19orf68,FKRP,MIR520D,ZNF701,MIR518A1,MIR520C,APOC2,MYPOP,SNORD32A,SIGLEC11,LIG1,SIGLEC5,NAPSA,LILRA5,TEAD2,CD3EAP,ZNF613,IGFL4,MIR525,ADM5,SNAR-A14,DHDH,CRX,SULT2B1,ERCC1,MIR517A,LOC101928804,RPL18,ZNF28,MIR642A,BSPH1,MIR3191,MIR6798,KLC3,MIR769,SPIB,SNORD23,MIR520B,KIR3DL3,MIR518E,EXOC3L2,FAM71E1,SNAR-A4,FUZ,SNAR-A13,SNAR-A6,SIX5,DMWD,ZNF525,RPL13AP5,ALDH16A1,C19orf73,FUT2,LOC101059948,LOC101928517,MIR6800,PPP1R13L,TBC1D17,MGC45922,TPRX1,KLKP1,VASP,ZNF610,IRF3,NKPD1,MIR518D,CACNG6,SNAR-F,GYS1,SEC1P,SPHK2,VSTM1,ETFB,RTN2,ERVV-2,MIR5088,LILRB3,EMC10,RSPH6A,LOC400706,FOXA3,HSD17B14,CARD8,MIR643,SNORD88C,MIR1283-2,IGFL1,APOC1P1,ZNF614,CYTH2,QPCTL,KLK11,LIN7B,PNMAL2,ZNF432,PNKP,MIR520E,SNAR-D,KCNC3,TFPT,NTN5,MIR6801,LMTK3,PRKD2,ZNF541,KIR2DL4,ZNF296,ZNF473,SNAR-A7,FAM83E,NAPA-AS1,GFY,MIR8074,ZNF616,MIR371A,SEPW1,FCAR,SHANK1,ZNF480,SIGLEC14,SNAR-C1,CLEC11A,CCDC155,RUVBL2,MIR642B,MIR519E,LENG8,PRRG2,PNMAL1,LILRA4,ZNF331,KLK6,NUCB1,NKG7,MIR3190,SNAR-C5,C5AR2,RPS11,FPR3,NOSIP,CABP5,CLASRP,IZUMO1,ZNF114,MIR526A1,KIR3DX1,SIGLEC9,PRMT1,LRRC4B,PPM1N,PRR12,IL4I1,KCNA7,FAM90A27P,ZNF83,GEMIN7,PGLYRP1,MIR320E,ZNF841,KLK1,KLK7,FPR2,SPACA4,ERVV-1,CDC42EP5,SNAR-A5,KCNJ14,FPR1,AKT1S1,SIGLEC6,LAIR1,ZNF816,ZNF816-ZNF321P,SNORD35A,IGFL2,ZNF320,SLC8A2,FUT1,NLRP2,SLC1A5,CD33,LINC01530,C5AR1,MARK4,MIR4750,BCAT2,SNAR-C2,MIR519C,VSIG10L,CACNG7,ZNF702P,SNAR-G2,PTH2,BCL2L12,LILRP2,MIR516A1,ARHGAP35,CCDC61,GP6,ELSPBP1,SNAR-A2,KIR3DL2,HAS1,PRPF31,CPT1C,MIR522,SNAR-E,NUP62,ZNF528,FLJ26850,NPAS1,LENG9,TMEM160,LOC100129083,LOC101928295,PVRL2,MIR4752,MIR519B,RPS9,LILRA3,BIRC8,SNRPD2,MIR526B,MBOAT7,LILRB2,TRAPPC6A,BAX,IGFL3,CNOT3,MIR99B,ZNF468,ZNF818P,ZC3H4,SPACA6P-	4	gain
3049	CGTE_38	19	55549296	56222936	19q13.42	HSPBP1,EPS8L1,GP6,SSC5D,ISOC2,EPN1,RPL28,SBK2,MIR7975,ZNF865,ZNF579,NAT14,RDH13,TMEM150B,PPP6R1,BRSK1,ZNF628,PTPRH,TNNI3,UBE2S,FIZ1,COX6B2,SBK3,U2AF2,DNAAF3,TMEM86B,ZNF524,MIR6804,PPP1R12C,MIR6803,TMEM190,TMEM238,CCDC106,KMT5C,MIR6805,MIR6802,SHISA7,ZNF580,NLRP9,IL11,ZNF581,TNNT1,SYT5,ZNF784,FAM71E2	3	gain

3050	CGTE_38	19	56223183	59093745	19q13.43-q13.42	ZNF154,ZNF587B,ZNF773,ZIM2-AS1,ZIM2,A1BG-AS1,SMIM17,LOC101928886,C19orf18,ZNF135,ZNF530,ZNF419,GALP,ZNF547,ZNF544,LOC100128398,UBE2M,ZNF835,ZSCAN4,ZNF772,ZIM3,FKBP1AP1,ZNF776,ZSCAN18,CENPBD1P1,ZSCAN22,ZNF584,MZF1,ZNF470,ZNF582,USP29,MIMT1,ZNF264,CHMP2A,TRIM28,ZNF667-AS1,ZNF814,ZNF416,NLRP8,ZNF71,ZSCAN5B,PEG3,ZNF671,ZNF551,ZNF549,NLRP5,NLRP11,NLRP13,RPS5,TRAPPC2B,ZNF211,ZNF471,MIR6806,RFPL4AL1,ZNF274,MIR4754,ZNF587,ZNF805,ZNF418,ZNF134,ZSCAN5A,ZNF304,ZNF324B,ZBTB45,ZNF460,ZNF324,ZNF446,ZNF586,ZSCAN1,NLRP9,A1BG,ZNF543,RNF225,ZNF256,ZIK1,ZNF548,MIR6807,ZNF550,ZNF582-AS1,ZNF132,ZNF542P,ZNF667,ZNF17,MZF1-AS1,DUXA,SLC27A5,ZNF583,ZNF417,ZNF787,NLRP4,ZNF749,ZNF497,PEG3-AS1,RFPL4A,ZNF552,AURKC,VN1R1,ZNF8,ZFP28,ZNF837,ZNF444,ZNF606,ZNF329	4	gain
3051	CGTE_38	20	68259	3210951	20p13	SNORD56,CPXM1,ITPA,DDRGK1,EBF4,VPS16,LZTS3,SLC52A3,SNRPB,RAD21L1,SDCBP2,PCED1A,NSFL1C,UBOX5,SNORD86,NOP56,RSPO4,SIRPD,TGM3,OXT,SIRPB1,FASTKD5,ANGPT4,DEFB126,ZNF343,NRSN2,SNORD119,DEFB125,C20orf96,FKBP1A-SDCBP2,SLC4A11,PTPRA,TRIB3,ZCCHC3,SIRPB2,DEFB128,SIRPG-AS1,LOC388780,DEFB129,IDH3B,NRSN2-AS1,SIRPA,UBOX5-AS1,DEFB132,SCRT2,LOC100289473,STK35,DEFB127,GNRH2,TMEM74B,SIRPG,TGM6,CSNK2A1,FKBP1A,SNORD57,TMC2,SRXN1,FAM110A,SNORD110,MRPS26,TMEM239,TCF15,RBCK1,MIR1292,TBC1D20,C20orf141,SOX12,PSMF1,SNORA51,PDYN,C20orf202,AVP,SDCBP2-AS1,MIR6869,SNPH	3	gain
3052	CGTE_38	20	3211162	3214966	20p13	SLC4A11	15	gain
3053	CGTE_38	20	3215205	21494375	20p12.2-p11.23	SNORD17,SEC23B,JAG1,LOC101929395,PRND,LOC101929312,MKKS,DTD1,SLC4A11,SLC23A2,LINC00658,BMP2,RRBP1,KIZ-AS1,TRMT6,BTBD3,LOC101929486,LOC100505515,LAMP5,CSRP2BP,RNF24,SLX4IP,SMOX,PCSK2,C20orf27,LAMP5-AS1,MACROD2-IT1,ADAM33,PCNA-AS1,MIR8062,CHGB,XRN2,LOC100270804,NKX2-4,KIF16B,MGME1,FLRT3,PRNT,SIGLEC1,LOC101929125,LOC613266,MIR103A2,MACROD2,LINC00493,RALGAPA2,ISM1,LINC00687,PRNP,CDS2,ZNF133,LOC100130264,LOC643406,BFSP1,TMX4,RBBP9,CASC20,C20orf196,SPTLC3,DZANK1,PROKR2,OVOL2,PAK7,LOC101929413,CRLS1,LINC01428,LRRN4,ISM1-AS1,MIR6870,SEL1L2,SCP2D1,GFRA4,HSPA12B,MCM8-AS1,MIR3192,LOC339593,SNX5,C20orf78,INSM1,LINC01433,LINC00652,CFAP61,LOC101929371,PLCB4,MIR103B2,POLR3F,NDUFAF5,PCNA,OTOR,SNAP25,NKX2-2,ADRA1D,HAO1,ATRN,MACROD2-AS1,SNRPB2,PET117,ESF1,SNAP25-AS1,KIZ,CENPB,FERMT1,BANF2,LINC00851,PANK2,CRNKL1,RASSF2,RIN2,AP5S1,TASP1,PLCB1,MAVS,SPEF1,SLC24A3,CDC25B,GPCPD1,LOC101929207,DSTN,TMEM230,C20orf194,LOC101929526,MCM8,ANKEF1,NAA20,LOC102606466,LINC00654	3	gain
3054	CGTE_38	20	21494396	23030432	20p11.22-p11.21	LOC101929625,FOXA2,THBD,LOC101929608,LINC00261,LINC01384,LINC01432,NKX2-2,LINC01427,SSTR4,PAX1,LOC284788	0	loss

3055	CGTE_38	20	23064445	60883807	20p11.21-p11.1	WFDC10B,ZSWIM1,CST1,COX4I2,CST4,BPIFA3,WFDC8,RBPJL,KCNK15,FRG1DP,CD40,ZFAS1,HNF4A,MC3R,MIR3194,LOC729296,SNORA71B,UQCC1,MIR644A,RPN2,MIR1289-1,PABPC1L,ADA,RBM39,MTG2,SLPI,FAM65C,NEURL2,SOGA1,TUBB1,SCAND1,CNBD2,CST7,DLGAP4-AS1,CHD6,MOCS3,PHACTR3,EPPIN,TSPY26P,MAPRE1,CTSZ,SNX21,MIR645,GCNT7,DEFB119,ZNF337-AS1,PLAGL2,RAB22A,SAMHD1,HM13-AS1,PIGU,EYA2,SYS1,ZHX3,LSM14B,ZNF335,CSTF1,STK4-AS1,MIR3616,CPNE1,RTFDC1,CST3,ADNP,RBM38,WFDC9,CASS4,C20orf203,EDEM2,ASIP,NOL4L,CSE1L,MIR298,VSTM2L,ANKRD60,ADNP-AS1,BCL2L1,NCOR1P1,NANP,MIR1257,FAM182A,MANBAL,MLLT10P1,OSER1,LINC01260,SLC32A1,TTPAL,E2F1,WFDC11,ARFGEF2,MMP24-AS1,GDAP1L1,NCOA3,ITCH,LOC101928048,PLCG1,TGIF2,GNAS-AS1,FAM209B,OCSTAMP,FAM182B,DYNLRB1,APCDD1L,NINL,CTNNBL1,LINC01524,SNORD12B,NDRG3,LINC00489,MIR3195,EPB41L1,LOC100506175,TP53TG5,SNHG17,RPRD1B,TTLL9,MKRN7P,TRPC4AP,SRC,TP53INP2,DHX35,PSMA7,NPEPL1,BPIFB3,LINC01620,PKIG,C20orf24,PPP1R16B,STX16-NPEPL1,SGK2,PTPRT,LOC100506470,PTPN1,BPIFB1,SUMO1P1,GZF1,ROMO1,ARHGAP40,TRERNA1,SUN5,SPATA2,PARD6B,NCOA5,NCOA6,ZNF217,TLDC2,MIR3193,LBP,DEFB122,ACSS2,LINC00657,SLC2A10,CEP250,KIAA1755,TGIF2-C20orf24,WFDC12,DOK5,TPX2,SLC12A5,LINC01441,RALY-AS1,RNF114,MATN4,SNORA71D,BCAS1,KCNB1,CST9,DLGAP4,MIR646HG,NFS1,GHRH,LOC149684,TSHZ2,TP53RK,MIR663A,DEFB121,PPP4R1L,DUSP15,MIR7641-2,ZMYND8,AURKA,RALGAPB,MIR4533,SALL4,MIR6871,LINC01598,CYP24A1,MIR4755,APMAP,DEFB118,APCDD1L-AS1,CST2,EDN3,LINC00494,MIR4532,ACOT8,SULF2,LINC01270,PLTP,SNTA1,CDH26,ERGIC3,JPH2,LOC101926955,C20orf197,DEFB115,TOX2,BMP7-AS1,TAF4,MIR296,KCNS1,HCK,LINC00028,FAM210B,AAR2,KCNK15-AS1,NECAB3,MIR1302-5,ACSS1,FITM2,LPIN3,ADRM1,GGT7,STK4,MIR4325,MIR3617,MIR548AG2,ASXL1,L3MBTL1,SRSF6,ZNF334,LINC00656,TNNC2,ATP9A,PTGIS,NKILA,RBM12,CEBPB-AS1,CST5,LINC01440,LINC01431,NELFCD,FER1L4,KCNG1,RIMS4,EIF6,SERINC3,OSBPL2,ADIG,ACTR5,ZNF831,FLJ33581,SNAI1,CST11,LOC101929698,IFT52,ID1,BMP7,LOC100506384,LOC101926935,DPM1,RAE1,MIR6812,SPATA25,HRH3,SLA2,HNF4A-AS1,DEFB116,LOC100131496,ZBP1,MCTS2P,DEFB124,PPP1R3D,LINC01522,LINC01273,MROH8,BPIFB6,GGTLC1,STX16,MMP24,FAM83D,GNAS,CSE1L-AS1,EIF2S2,UBE2C,NXT1,ZNFX1,DBNDD2,BPIFA4P,SNORA60,ZNF663P,MTRNR2L3,VAPB,ZNF337,CEBPB,COMMD7,TOP1,LINC01523,MYH7B,DNMT3B,WFDC10A,SNORD12C,MIR4756,SNORD12,CST8,SS18L1,SLC9A8,LINC01272,MAP1LC3A,SYCP2,PREX1,ABALON,YWHAB,LINC01370,PMEPA1,PLCG1-AS1,LOC101926889,LOC101927770,FAM83C,GSS,FAM209A,DEFB123,CD93,PHF20,C20orf85,ELMO2,ZFP64,LINC01430,WISP2,LOC339568,ABHD12,LOC149950,MYLK2,GINS1,LOC100287792,TTI1,C20orf144,LOC101927159,CBLN4,FRG1BP,STAU1,TMEM189,SNORA71A,BLCAP,CST9L,FAM217B,LINC01429,MIR1825,SLMO2-ATP5E,OSER1-AS1,LOC101927932,PIGT,UBE2V1,MAFB,SEMG2,FAM83C-	3	gain
3056	CGTE_38	20	60884380	61510671	20q13.33	LAMA5,GATA5,MRGBP,MIR4758,DPH3P1,RPS21,MIR1-1HG,CABLES2,LAMA5-AS1,NTSR1,OGFR,MIR1-1,OGFR-AS1,TCFL5,C20orf166-AS1,DIDO1,SLCO4A1,SLCO4A1-AS1,LINC00659,RBBP8NL,COL9A3,MIR133A2	0	loss
3057	CGTE_38	20	61510754	61597106	20q13.33	DIDO1,SLC17A9,GID8	3	gain
3058	CGTE_38	20	61597848	62046569	20q13.33	MIR3196,LOC63930,KCNQ2,CHRNA4,YTHDF1,BHLHE23,ARFGAP1,LINC01056,NKAIN4,MIR4326,FLJ16779,MIR124-3,HAR1B,COL20A1,LOC100130587,LINC00029,HAR1A,BIRC7,SLC17A9	0	loss
3059	CGTE_38	20	62050934	62120512	20q13.33	KCNQ2,EEF1A2	5	gain

3060	CGTE_38	21	31311664	32410828	21q22.11-q21.3	KRTAP22-2,KRTAP20-3,KRTAP15-1,KRTAP13-1,KRTAP21-1,KRTAP19-4,KRTAP6-3,KRTAP25-1,KRTAP19-1,KRTAP21-3,KRTAP19-3,KRTAP13-3,KRTAP19-6,KRTAP21-2,KRTAP24-1,KRTAP20-4,KRTAP20-2,KRTAP6-2,LINC00307,CLDN17,KRTAP7-1,KRTAP20-1,KRTAP27-1,GRIK1,KRTAP13-4,KRTAP11-1,KRTAP19-8,CLDN8,KRTAP19-7,KRTAP19-5,MIR4327,KRTAP6-1,KRTAP26-1,KRTAP13-2,KRTAP8-1,KRTAP23-1,KRTAP22-1,KRTAP19-2	3	gain
3061	CGTE_38	21	46057516	46074404	21q22.3	KRTAP10-11,KRTAP10-10,KRTAP12-4,TSPEAR	6	gain
3062	CGTE_38	21	46702267	47424671	21q22.3	COL18A1-AS2,COL18A1-AS1,PCBP3,SLC19A1,COL18A1,LOC101928796,LOC642852,LOC100129027,LINC00316,POFUT2,COL6A1,MIR6815	1	loss
3063	CGTE_38	21	47531374	47536771	21q22.3	COL6A2	6	gain
3064	CGTE_38	22	22316729	23214235	22q11.22	BMS1P20,POM121L1P,PRAME,ZNF280A,GGTLC2,MIR650,LL22NC03-63E9.3,VPREB1,ZNF280B,TOP3B	3	gain
3065	CGTE_38	22	50609165	50904830	22q13.33	PANX2,TRABD,SELO,PPP6R2,PLXNB2,SBF1,MAPK12,DENND6B,TUBGCP6,MAPK11,HDAC10	0	loss
3066	CGTE_38	X	152864479	152915194	Xq28	FAM58A,LOC105373383,DUSP9	6	gain
3067	CGTE_38	X	152915382	153629353	Xq28	TMEM187,IDH3G,MIR3202-2,MIR718,DUSP9,PNCK,MIR3202-1,SRPK3,SNORA70,ABCD1,PLXNB3,OPN1MW,RENBP,OPN1MW2,PDZD4,TKTL1,FLNA,RPL10,MECP2,ARHGAP4,LCA10,NAA10,L1CAM,SSR4,TEX28,HCFC1-AS1,HCFC1,EMD,IRAK1,BCAP31,AVPR2,OPN1LW,SLC6A8	1	loss
3068	CGTE_39	1	985576	987203	1p36.33	AGRN	5	gain
3069	CGTE_39	1	1132786	1139644	1p36.33	TTLL10,TNFRSF18	5	gain
3070	CGTE_39	1	1139768	1148902	1p36.33	TNFRSF18,TNFRSF4	1	loss
3071	CGTE_39	1	1178191	1179883	1p36.33	FAM132A	6	gain
3072	CGTE_39	1	1563392	1565870	1p36.33	MIB2	9	gain
3073	CGTE_39	1	3410927	3413331	1p36.32	MEGF6	5	gain
3074	CGTE_39	1	228463390	228464436	1q42.13	OBSCN	23	gain
3075	CGTE_39	2	43451377	43452948	2p21	ZFP36L2	5	gain
3076	CGTE_39	2	45169390	45171964	2p21	SIX3	5	gain
3077	CGTE_39	2	73052921	73114960	2p13.2	SPR,EXOC6B	87	gain
3078	CGTE_39	2	73492461	73493598	2p13.2	FBXO41	3	gain
3079	CGTE_39	2	73493623	73496753	2p13.2	FBXO41	0	loss
3080	CGTE_39	2	74741195	74743220	2p13.1	TLX2	13	gain
3081	CGTE_39	2	95540530	95691686	2q11.1	LOC442028,TEKT4,MAL	7	gain
3082	CGTE_39	2	101767605	101870015	2q11.2	CNOT11,TBC1D8	4	gain
3083	CGTE_39	2	219757660	219825892	2q35	CDK5R2,WNT10A,LINC01494	25	gain
3084	CGTE_39	2	219857691	219867844	2q35	CFAP65,CRYBA2,MIR375,LOC100129175	7	gain
3085	CGTE_39	2	233386764	233390070	2q37.1	PRSS56	1	loss
3086	CGTE_39	2	239009324	239013579	2q37.3	ESPNL	5	gain
3087	CGTE_39	2	242707092	242754901	2q37.3	D2HGDH,NEU4,GAL3ST2	3	gain
3088	CGTE_39	2	242755175	242757743	2q37.3	NEU4	0	loss
3089	CGTE_39	2	242757746	242842580	2q37.3	RTP5,LINC01237,PDCD1,NEU4	3	gain
3090	CGTE_39	3	9987907	9989646	3p25.3	PRRT3,PRRT3-AS1	0	loss
3091	CGTE_39	3	50312770	50314222	3p21.31	SEMA3B	12	gain
3092	CGTE_39	3	138724504	138738804	3q23	PRR23B,PRR23A	7	gain
3093	CGTE_39	4	954750	960069	4p16.3	DGKQ	3	gain
3094	CGTE_39	4	961815	981740	4p16.3	DGKQ,IDUA,SLC26A1	3	gain
3095	CGTE_39	4	982642	985752	4p16.3	SLC26A1,IDUA	5	gain
3096	CGTE_39	4	1807041	1808710	4p16.3	FGFR3	3	gain
3097	CGTE_39	4	2043007	2044383	4p16.3	C4orf48	9	gain
3098	CGTE_39	4	3432335	3445893	4p16.3	HGFAC,RGS12	3	gain

3099	CGTE_39	4	3447510	3502527	4p16.3	DOK7,HGFAC	3	gain
3100	CGTE_39	4	3768419	3769730	4p16.3	ADRA2C	10	gain
3101	CGTE_39	4	13485654	13543845	4p15.33	LINC01097,NKX3-2,RAB28	3	gain
3102	CGTE_39	5	2749447	2752641	5p15.33	IRX2,C5orf38	3	gain
3103	CGTE_39	5	92920861	92924208	5q15	MIR548AO,NR2F1	5	gain
3104	CGTE_39	5	140188016	140188988	5q31.3	PCDHA2,PCDHA3,PCDHA1,PCDHA4	4	gain
3105	CGTE_39	5	140207766	140216026	5q31.3	PCDHA2,PCDHA5,PCDHA3,PCDHA4,PCDHA1,PCDHA6,PCDHA7	3	gain
3106	CGTE_39	5	140256442	140257143	5q31.3	PCDHA8,PCDHA7,PCDHA2,PCDHA3,PCDHA5,PCDHA6,PCDHA4,PCDHA1,PCDHA11,PCDHA12,PCDHA10,PCDHA9	5	gain
3107	CGTE_39	5	176023753	176025158	5q35.2	GPRIN1	4	gain
3108	CGTE_39	6	3227755	3255416	6p25.2	TUBB2B	3	gain
3109	CGTE_39	6	31324110	31324996	6p21.33	HLA-B	3	gain
3110	CGTE_39	6	37616725	37617791	6p21.2	MDGA1	0	loss
3111	CGTE_39	6	43227286	43251172	6p21.1	TTBK1	4	gain
3112	CGTE_39	6	43251177	43253116	6p21.1	TTBK1	3	gain
3113	CGTE_39	6	127836618	127837673	6q22.33	SOGA3	3	gain
3114	CGTE_39	7	44924502	45002733	7p13	PURB,MYO1G	33	gain
3115	CGTE_39	7	72722337	72727628	7q11.23	NSUN5,TRIM50	6	gain
3116	CGTE_39	7	100806022	100816785	7q22.1	NAT16,VGF	9	gain
3117	CGTE_39	8	144662852	144668620	8q24.3	EEF1D	12	gain
3118	CGTE_39	8	145017839	145024901	8q24.3	MIR661,PLEC	4	gain
3119	CGTE_39	9	124414069	124461707	9q33.2	DAB2IP	5	gain
3120	CGTE_39	9	139089140	139090912	9q34.3	LHX3	3	gain
3121	CGTE_39	9	139091449	139096861	9q34.3	LHX3	13	gain
3122	CGTE_39	9	139271962	139273930	9q34.3	SNAPC4	7	gain
3123	CGTE_39	9	139276405	139277985	9q34.3	SNAPC4	5	gain
3124	CGTE_39	9	139390778	139397802	9q34.3	NOTCH1	3	gain
3125	CGTE_39	9	139398936	139401157	9q34.3	NOTCH1	6	gain
3126	CGTE_39	9	139731757	139748169	9q34.3	MAMDC4,PHPT1,RABL6,C9orf172	3	gain
3127	CGTE_39	9	139748172	139750824	9q34.3	MAMDC4	6	gain
3128	CGTE_39	9	139820461	139838494	9q34.3	FBXW5,TRAF2	4	gain
3129	CGTE_39	9	139913294	139916049	9q34.3	ABCA2	7	gain
3130	CGTE_39	9	140120446	140123594	9q34.3	RNF224,CYSRT1	11	gain
3131	CGTE_39	9	140127612	140130900	9q34.3	SLC34A3	7	gain
3132	CGTE_39	9	140167176	140174478	9q34.3	TOR4A,NELFB	0	loss
3133	CGTE_39	9	140327886	140329812	9q34.3	NOXA1,ENTPD8	12	gain
3134	CGTE_39	9	140351824	140353591	9q34.3	NSMF	5	gain
3135	CGTE_39	10	27702744	27793808	10p12.1	RAB18,PTCHD3	7	gain
3136	CGTE_39	10	76994622	77159485	10q22.2	ZNF503-AS1,ZNF503,COMTD1	0	loss
3137	CGTE_39	10	99079047	99080200	10q24.1	FRAT1	47	gain
3138	CGTE_39	10	120513904	120790135	10q26.11	NANOS1,CACUL1	0	loss
3139	CGTE_39	11	788141	792998	11p15.5	CEND1,SLC25A22	7	gain
3140	CGTE_39	11	824170	825018	11p15.5	PNPLA2	7	gain
3141	CGTE_39	11	1247658	1255724	11p15.5	MUC5B	3	gain
3142	CGTE_39	11	64135895	64139065	11q13.1	RPS6KA4,MIR1237	6	gain
3143	CGTE_39	11	67205051	67206480	11q13.2	PTPRCAP,CORO1B	6	gain
3144	CGTE_39	12	132400404	132404156	12q24.33	ULK1	4	gain
3145	CGTE_39	12	133067154	133158159	12q24.33	FBRSL1	3	gain
3146	CGTE_39	12	133158221	133160392	12q24.33	MIR6763,FBRSL1	8	gain
3147	CGTE_39	13	78235542	78272705	13q22.3	LOC100129307,MIR3665,SLAIN1	0	loss
3148	CGTE_39	13	100637323	100741487	13q32.3	LOC105370333,ZIC2,LINC00554,PCCA	1	loss

3149	CGTE_39	13	110434381	110438412	13q34	IRS2	0	loss
3150	CGTE_39	14	103987620	103988824	14q32.32	CKB	102	gain
3151	CGTE_39	14	104569793	104578087	14q32.33	ASPG	3	gain
3152	CGTE_39	14	104633030	104641459	14q32.33	KIF26A	3	gain
3153	CGTE_39	14	104641463	104645839	14q32.33	KIF26A	0	loss
3154	CGTE_39	14	105349358	105353205	14q32.33	CEP170B	7	gain
3155	CGTE_39	14	105611106	105613102	14q32.33	JAG2	4	gain
3156	CGTE_39	14	105931405	105936492	14q32.33	MTA1	4	gain
3157	CGTE_39	15	41136241	41137285	15q15.1	SPINT1	0	loss
3158	CGTE_39	15	48623728	48624688	15q21.1	DUT	0	loss
3159	CGTE_39	15	52849382	52861044	15q21.2	ARPP19	1	loss
3160	CGTE_39	15	68119103	68121600	15q23	SKOR1	0	loss
3161	CGTE_39	15	80987785	80988424	15q25.1	ABHD17C	0	loss
3162	CGTE_39	17	40705158	40706686	17q21.2	HSD17B1	4	gain
3163	CGTE_39	17	61548870	61554711	17q23.3	ACE	4	gain
3164	CGTE_39	17	76113303	76114031	17q25.3	TMC6	5	gain
3165	CGTE_39	17	77983991	77987332	17q25.3	TBC1D16	14	gain
3166	CGTE_39	17	79093114	79095093	17q25.3	AATK	5	gain
3167	CGTE_39	17	79095094	79096622	17q25.3	AATK	0	loss
3168	CGTE_39	17	79096926	79165170	17q25.3	AATK-AS1,AATK,CEP131,MIR657,MIR338,MIR3065,MIR1250	3	gain
3169	CGTE_39	17	79409175	79412217	17q25.3	BAHCC1	0	loss
3170	CGTE_39	17	79412344	79414900	17q25.3	BAHCC1	8	gain
3171	CGTE_39	17	79977584	79981825	17q25.3	LRRC45,STRA13	20	gain
3172	CGTE_39	18	19751067	19757114	18q11.2	GATA6	0	loss
3173	CGTE_39	18	24127679	24171532	18q11.2	KCTD1,MIR8057	4	gain
3174	CGTE_39	18	76740231	76754997	18q23	SALL3	5	gain
3175	CGTE_39	19	7795141	7796111	19p13.2	CLEC4G	7	gain
3176	CGTE_39	19	39660172	39666158	19q13.2	PAK4	5	gain
3177	CGTE_39	19	45649064	45662373	19q13.32	NKPD1,PPP1R37	6	gain
3178	CGTE_39	20	3765656	3777513	20p13	CENPB,CDC25B	6	gain
3179	CGTE_39	20	60882313	60893689	20q13.33	LAMA5,ADRM1	3	gain
3180	CGTE_39	20	62165533	62195221	20q13.33	SRMS,PTK6,C20orf195,HELZ2	4	gain
3181	CGTE_39	20	62195225	62197261	20q13.33	HELZ2	10	gain
3182	CGTE_39	21	45970507	46101952	21q22.3	KRTAP10-9,TSPEAR,KRTAP10-5,KRTAP10-3,KRTAP10-2,KRTAP12-1,KRTAP10-10,KRTAP12-2,KRTAP12-3,KRTAP10-6,KRTAP10-8,KRTAP10-4,KRTAP12-4,KRTAP10-11,KRTAP10-7	3	gain
3183	CGTE_39	22	17589465	17590650	22q11.1	IL17RA	9	gain
3184	CGTE_39	22	19709756	19712059	22q11.21	GP1BB,SEPT5-GP1BB,SEPT5	6	gain
3185	CGTE_39	22	20130396	20136478	22q11.21	ZDHHC8,CCDC188	0	loss
3186	CGTE_39	22	20136679	20137238	22q11.21	CCDC188	6	gain
3187	CGTE_39	22	50705363	50706398	22q13.33	MAPK11	8	gain
3188	CGTE_39	22	50892927	50898014	22q13.33	SBF1	3	gain
3189	CGTE_39	22	50898019	50898760	22q13.33	SBF1	0	loss
3190	CGTE_39	22	50942911	50946884	22q13.33	NCAPH2,LMF2	4	gain
3191	CGTE_39	X	153599508	153608165	Xq28	EMD,FLNA	10	gain
3192	CGTE_40	1	861266	1245321	1p36.33	MIR200A,UBE2J2,MIR429,MIR6726,TNFRSF18,NOC2L,AGRN,B3GALT6,TNFRSF4,LINC01342,SAMD11,PERM1,HES4,RNF223,MIR200B,ACAP3,PLEKHN1,SDF4,C1orf159,TTLL10,PUSL1,KLHL17,SCNN1D,FAM132A,ISG15	0	loss

3193	CGTE_40	1	1245890	3692052	1p36.32-p36.33	AURKAIP1,TNFRSF14,MRPL20,ARHGEF16,CDK11B,GABRD,SKI,MMP23A,LOC100129534,MIR4251,NADK,CALML6,FAAP20,MIR6727,SSU72,WRAP73,MIB2,CDK11A,TP73,CPTP,ATAD3A,LOC102724312,SLC35E2,PEX10,ATAD3B,TP73-AS1,PRDM16,MORN1,RER1,LOC148413,VWA1,MXRA8,PRKCZ,ACTRT2,C1orf233,LINC00982,TMEM240,ANKRD65,SMIM1,LOC100996583,MIR551A,TPRG1L,CPSF3L,PLCH2,FAM213B,TMEM88B,CCDC27,MIR6808,PANK4,TTC34,PUSL1,TMEM52,MMEL1,LOC115110,MEGF6,TAS1R3,CCNL2,MMP23B,CFAP74,DVL1,GNB1,HES5,SLC35E2B,ATAD3C	1	loss
3194	CGTE_40	1	228333228	228588972	1q42.13	GJC2,MIR6742,IBA57-AS1,IBA57,TRIM11,GUK1,C1orf145,OBSCN	1	loss
3195	CGTE_40	2	109086037	109088020	2q12.3	GCC2	7	gain
3196	CGTE_40	2	239006590	239059847	2q37.3	SCLY,KLHL30,UBE2F-SCLY,ESPNL	0	loss
3197	CGTE_40	2	239067499	239076271	2q37.3	FAM132B	9	gain
3198	CGTE_40	3	47042499	47049379	3p21.31	NBEAL2	3	gain
3199	CGTE_40	3	126193786	126207145	3q21.3	ZXDC,UROC1	10	gain
3200	CGTE_40	4	858828	860338	4p16.3	GAK	11	gain
3201	CGTE_40	4	860648	1805559	4p16.3	MAEA,TMED11P,SLBP,TMEM175,CTBP1,RNF212,DGKQ,FGFRL1,SLC26A1,SPON2,IDUA,CRIPAK,TMEM129,FAM53A,LOC100130872,TACC3,GAK,FGFR3,NKX1-1,CTBP1-AS2,UVSSA,CTBP1-AS	1	loss
3202	CGTE_40	4	1806029	1806485	4p16.3	FGFR3	14	gain
3203	CGTE_40	4	1806505	1816262	4p16.3	LETM1,FGFR3	0	loss
3204	CGTE_40	4	3432335	3475372	4p16.3	HGFAC,DOK7,RGS12	0	loss
3205	CGTE_40	4	56329784	70800563	4q13.3-q13.2	UGT2B10,IGFBP7,TMPRSS11D,KIAA1211,NMU,EPHA5,ADGRL3,TMPRSS11GP,MIR7641-2,PPAT,HOPX,UBA6-AS1,UGT2B28,SPINK2,UGT2A1,NOA1,LOC644145,SYT14P1,CLOCK,LOC550113,CSN1S1,TMPRSS11F,MIR1269A,PDCL2,UBA6,CENPC,UGT2A2,THEGL,MIR548AG1,CEP135,UGT2B7,TMPRSS11A,LOC401134,SULT1B1,TMPRSS11BNL,TMPRSS11E,LOC101928851,AASDH,UGT2B17,ADGRL3-AS1,LOC101927237,SULT1E1,ARL9,EXOC1,UGT2B4,UGT2A3,REST,GNRHR,UGT2B15,STAP1,POLR2B,FTLP10,SRP72,PAICS,IGFBP7-AS1,UGT2B11,TMPRSS11B,EPHA5-AS1,YTHDC1,TECRL	1	loss
3206	CGTE_40	4	70801612	70803639	4q13.3	CSN1S1	37	gain
3207	CGTE_40	4	70804746	79856425	4q21.21-q21.1	CABS1,NUP54,CCNI,CSN2,GC,SOWAHB,SCARB2,CSN3,CXCL2,PAQR3,HTN3,MIR4450,AFM,PPBP,BMP2K,CXCL8,FDCSP,STBD1,ART3,LOC728040,USO1,CXCL9,GRSF1,SMR3A,MIR548AH,CXCL13,LOC100507388,AMBN,ODAM,CXCL1,RASSF6,PF4,RUFY3,ALB,SLC4A4,MRPL1,LOC441025,BTC,RCHY1,AMTN,ADAMTS3,AFP,CSN1S2AP,PARM1,SHROOM3,EREG,CXCL3,CXCL6,PF4V1,C4orf26,MUC7,LINC01094,CXCL10,UTP3,SDAD1,JCHAIN,CSN1S2BP,MIR5096,ANXA3,MTHFD2L,SEPT11,NPFFR2,FRAS1,CDKL2,PPBPP2,FAM47E-STBD1,PPEF2,PROL1,CSN1S1,CXCL11,AREG,MOB1B,G3BP2,EPGN,STATH,CXCL5,COX18,CCNG2,CNOT6L,THAP6,SMR3B,FAM47E,ANKRD17,NAAA,HTN1,DCK,ENAM,PRR27,CCDC158,LOC101928809	1	loss
3208	CGTE_40	4	122853377	123091993	4q27	KIAA1109,TRPC3	8	gain
3209	CGTE_40	5	1574310	3600446	5p15.33	IRX1,SDHAP3,LINC01017,LINC01019,LOC100506858,IRX4,MRPL36,CTD-2194D22.4,LOC728613,LOC101929034,NDUFS6,IRX2,MIR4277,LINC01377,C5orf38	0	loss
3210	CGTE_40	5	140167086	140257143	5q31.3	PCDHA9,PCDHA3,PCDHA2,PCDHA5,PCDHA8,PCDHA7,PCDHA6,PCDHA10,PCDHA4,PCDHA1,PCDHA11,PCDHA12	0	loss
3211	CGTE_40	5	172386735	172421778	5q35.1	RPL26L1,ATP6V0E1	10	gain
3212	CGTE_40	6	32156204	32159627	6p21.32	PBX2,GPSM3	6	gain
3213	CGTE_40	6	32552039	32629461	6p21.32	HLA-DQA1,HLA-DRB1,HLA-DQB1	4	gain
3214	CGTE_40	6	33131385	33136735	6p21.32	COL11A2	10	gain

3215	CGTE_40	6	33650282	33651916	6p21.31	ITPR3	7	gain
3216	CGTE_40	6	33995889	34030051	6p21.31	GRM4	4	gain
3217	CGTE_40	6	121447473	121481286	6q22.31	TBC1D32	9	gain
3218	CGTE_40	6	134373515	134436545	6q23.2	SLC2A12,HMGA1P7	17	gain
3219	CGTE_40	7	195557	228854	7p22.3	FAM20C	6	gain
3220	CGTE_40	7	142563387	142566926	7q34	EPHB6	10	gain
3221	CGTE_40	8	21959246	21960152	8p21.3	FAM160B2	21	gain
3222	CGTE_40	8	22052895	22067207	8p21.3	BMP1	4	gain
3223	CGTE_40	8	144240163	144922047	8q24.3	BREA2,MAFA-AS1,ZNF696,MIR937,NRBP2,GSDMD,ZNF623,GLI4,ZNF707,NAPRT,RHPN1,TIGD5,MIR4664,MAPK15,LY6H,EEF1D,PYCRL,ZFP41,PUF60,MIR6845,GPIHBP1,MROH6,RHPN1-AS1,SCRIB,FAM83H-AS1,ZC3H3,MINCR,FAM83H,TSTA3,TOP1MT,MAFA,CCDC166	0	loss
3224	CGTE_40	8	144922051	144941151	8q24.3	NRBP2,EPPK1	12	gain
3225	CGTE_40	8	144941154	145139387	8q24.3	OPLAH,PLEC,EPPK1,EXOSC4,MIR6846,PARP10,MIR6847,GPAA1,MIR661,SPATC1,GRINA	0	loss
3226	CGTE_40	8	145540214	145758721	8q24.3	SLC52A2,ADCK5,MIR6849,FBXL6,MFSD3,RECQL4,VPS28,LRRC14,TONSL-AS1,CYHR1,CPSF1,MIR6893,SLC39A4,KIFC2,DGAT1,C8orf82,TMEM249,LRRC24,MIR6848,PPP1R16A,TONSL,FOXH1,SCRT1,MIR939,ARHGAP39,GPT	0	loss
3227	CGTE_40	9	32384647	32635556	9p21.1	DDX58,TAF1L,NDUFB6,TOPORS-AS1,TOPORS,ACO1	3	gain
3228	CGTE_40	9	138903487	140353055	9q34.3	LCNL1,FUT7,SNORA17B,NELFB,NSMF,LCN15,LCN10,PTGDS,FBXW5,C9orf173,C9orf172,NOXA1,C9orf163,FAM166A,MIR7114,C9orf142,LCN12,C9orf139,MIR4292,LCN8,LOC100128593,SNAPC4,NPDC1,NACC2,MAN1B1,FAM69B,PMPCA,NOTCH1,AGPAT2,CCDC183-AS1,NRARP,MIR6722,ABCA2,MIR4674,GPSM1,TMEM141,EGFL7,ENTPD8,SSNA1,LHX3,RNF208,MIR4479,UAP1L1,QSOX2,ENTPD2,RABL6,CCDC183,TOR4A,C9orf173-AS1,ANAPC2,CARD9,DKFZP434A062,SNHG7,LCN6,TMEM210,EXD3,PHPT1,TRAF2,NDOR1,SAPCD2,INPP5E,MAN1B1-AS1,TMEM203,MIR126,DPP7,LRRC26,MIR3621,SEC16A,SDCCAG3,CLIC3,NALT1,EDF1,RNF224,GRIN1,C9orf69,TUBB4B,MAMDC4,TPRN,MIR4673,C8G,DNLZ,SNORA17A,SLC34A3,CYSRT1	0	loss
3229	CGTE_40	9	140353421	140356100	9q34.3	NSMF,PNPLA7	6	gain
3230	CGTE_40	10	48370726	48382328	10q11.22	ZNF488,RBP3	4	gain
3231	CGTE_40	10	94708004	94715540	10q23.33	EXOC6	6	gain
3232	CGTE_40	10	124344643	124351472	10q26.13	DMBT1	7	gain
3233	CGTE_40	10	134218298	134230635	10q26.3	PWWP2B	8	gain
3234	CGTE_40	11	64033899	64375632	11q13.1	RPS6KA4,PRDX5,ESRRA,KCNK4-TEX40,NRXN2,SLC22A12,BAD,KCNK4,MIR1237,PLCB3,MIR7155,CCDC88B,SLC22A11,GPR137,LOC100996455,TEX40,TRMT112	0	loss
3235	CGTE_40	11	64593981	64606708	11q13.1	CDC42BPG	0	loss
3236	CGTE_40	11	67172841	67378686	11q13.2	PITPNM1,AIP,GSTP1,NDUFV1,MIR6752,C11orf72,TBC1D10C,RPS6KB2,TMEM134,CABP2,PTPRCAP,CABP4,GPR152,CARNS1,CORO1B,CDK2AP2	0	loss
3237	CGTE_40	11	68157351	68181524	11q13.2	LRP5	7	gain
3238	CGTE_40	12	48370605	48372655	12q13.11	COL2A1	6	gain
3239	CGTE_40	12	124779984	124873314	12q24.31	FAM101A,ZNF664-FAM101A,NCOR2,MIR6880	0	loss
3240	CGTE_40	12	132271024	132396615	12q24.33	MMP17,SFSWAP,ULK1	0	loss
3241	CGTE_40	12	132397725	132399028	12q24.33	ULK1	10	gain
3242	CGTE_40	12	132399441	132404156	12q24.33	ULK1	0	loss
3243	CGTE_40	12	132504556	132505835	12q24.33	EP400	22	gain
3244	CGTE_40	13	22070140	22088634	13q12.11	MICU2	63	gain

3245	CGTE_40	13	113795187	113803926	13q34	F10	6	gain
3246	CGTE_40	13	114778596	114782883	13q34	RASA3	7	gain
3247	CGTE_40	14	23991141	24003806	14q11.2	ZFHX2,THTPA	3	gain
3248	CGTE_40	14	58862651	58869520	14q23.1	TOMM20L	7	gain
3249	CGTE_40	14	103576202	103589054	14q32.32	EXOC3L4	12	gain
3250	CGTE_40	14	104575535	105196547	14q32.33	ASPG,MIR203B,MIR203A,MIR4710,INF2,ADSSL1,KIF26A,TMEM179,C14orf180	0	loss
3251	CGTE_40	14	105241164	105334963	14q32.33	CEP170B,AKT1,ZBTB42,LINC00638	0	loss
3252	CGTE_40	14	105342541	105344863	14q32.33	CEP170B	5	gain
3253	CGTE_40	14	105346566	105412546	14q32.33	CEP170B,PLD4,AHNAK2	0	loss
3254	CGTE_40	14	105412779	105416196	14q32.33	AHNAK2	5	gain
3255	CGTE_40	14	105846377	106318638	14q32.33	ELK2AP,TEX22,MIR8071-2,CRIP2,MIR8071-1,CRIP1,MTA1,TMEM121,C14orf80,LOC100507437,PACS2	0	loss
3256	CGTE_40	15	42135798	42167033	15q15.1	PLA2G4B,MIR4310,SPTBN5,JMJD7-PLA2G4B	0	loss
3257	CGTE_40	15	42167050	42168853	15q15.1	SPTBN5	12	gain
3258	CGTE_40	15	50897021	50902159	15q21.2	TRPM7	8	gain
3259	CGTE_40	15	68064962	68114456	15q23	MAP2K5,SKOR1	9	gain
3260	CGTE_40	16	97450	685051	16p13.3	PRR35,RAB11FIP3,MRPL28,PIGQ,NHLRC4,HBA2,ARHGDIG,FAM234A,LUC7L,TMEM8A,LOC100134368,AXIN1,RHBDF1,C16orf13,CAPN15,HBM,RGS11,HBA1,NPRL3,SNRNP25,MIR5587,RAB40C,LINC00235,PDIA2,HBQ1,POLR3K,WFIKKN1,MPG,MIR3176,NME4,DECR2,HBZ	1	loss
3261	CGTE_40	16	685267	717892	16p13.3	FAM195A,C16orf13,WDR90	0	loss
3262	CGTE_40	16	718100	720234	16p13.3	RHOT2	10	gain
3263	CGTE_40	16	720237	728251	16p13.3	RHOT2,RHBDL1	0	loss
3264	CGTE_40	16	730522	732433	16p13.3	JMJD8,STUB1	9	gain
3265	CGTE_40	16	732438	2105597	16p13.3	SNORA78,TMEM204,FBXL16,NDUFB10,C16orf91,LMF1,SSTR5,HAGH,SOX8,NTHL1,ZNF598,SPSB3,TSR3,HAGHL,RPL3L,CLCN7,BAIAP3,IFT140,EME2,TELO2,MIR3177,NARFL,MEIOB,IGFALS,LMF1-AS1,WDR24,MIR662,CACNA1H,MAPK8IP3,RPUSD1,CCDC154,CHTF18,TPSB2,MSLN,TPSG1,GFER,NPW,LINC00254,TPSAB1,NOXO1,METRN,SYNGR3,RPS2,GNG13,SLC9A3R2,FAHD1,PTX4,FAM173A,GNPTG,NME3,HN1L,MRPS34,TSC2,JMJD8,SSTR5-AS1,CCDC78,C1QTNF8,UBE2I,TPSD1,UNKL,STUB1,PRR25,SNORA64,SNHG9,CRAMP1,SNORA10,HS3ST6,MSRB1,RNF151,NUBP2,TBL3	0	loss
3266	CGTE_40	16	2106087	2107366	16p13.3	TSC2	21	gain
3267	CGTE_40	16	2108768	2891476	16p13.3	MIR6768,MIR6511B2,TRAF7,PRSS41,ABCA17P,PKD1,SNORD60,ZG16B,TCEB2,NTN3,BRICD5,ABCA3,RAB26,PRSS21,MIR4516,TSC2,PGP,ECI1,FLJ42627,SNHG19,KCTD5,ATP6V0C,MIR6767,SRRM2,E4F1,MIR3178,MIR1225,AMDHD2,CEMP1,MIR3677,CASKIN1,SRRM2-AS1,MIR940,CCNF,LOC106660606,PRSS33,PDPK1,PRSS27,TBC1D24,DNASE1L2,MIR6511B1,MIR3180-5,C16orf59,MLST8,PRSS30P,ERVK13-1,LOC652276,MIR4717,RNPS1	1	loss
3268	CGTE_40	16	2891563	3196169	16p13.3	ZSCAN10,ZNF213,IL32,MMP25,PRSS22,LINC00514,CCDC64B,PKMYT1,TNFRSF12A,THOC6,HCFC1R1,CASP16P,FLYWCH1,MMP25-AS1,LOC100128770,CLDN6,FLYWCH2,CLDN9,PAQR4,PRSS30P,ZNF205,LOC101929613,KREMEN2,ZNF205-AS1,ZNF213-AS1	0	loss
3269	CGTE_40	16	85741580	85768895	16q24.1	C16orf74	11	gain

3270	CGTE_40	16	85775088	89043308	16q24.1-q24.2	LOC440390,SNAI3,FENDRR,MIR1910,MVD,LOC339059,CDT1,LOC100289580,ZC3H18,SLC7A5,FOXC2,LOC101928682,TRAPPC2L,LINC01081,CYBA,JPH3,ZNF469,LINC00917,SNAI3-AS1,ZCCHC14,FOXC2-AS1,MIR6774,COX4I1,BANP,APRT,EMC8,ZFPM1,C16orf95,LOC400553,RNF166,CA5A,KLHDC4,LOC100129697,PABPN1L,MAP1LC3B,LOC102724467,PIEZO1,LOC101928880,FOXF1,LOC101928708,LOC101928737,IRF8,LOC146513,C16orf74,MIR5189,CBFA2T3,LINC01082,MIR6775,IL17C,MIR4722,CTU2,FBXO31,MTHFSD,FOXL1,FLJ30679,GALNS	1	loss
3271	CGTE_40	16	89114151	89188915	16q24.3	ACSF3	8	gain
3272	CGTE_40	17	80022515	80051264	17q25.3	DUS1L,SNORD134,FASN	0	loss
3273	CGTE_40	18	23738091	23772484	18q11.2	PSMA8	7	gain
3274	CGTE_40	18	77156185	77171501	18q23	NFATC1	0	loss
3275	CGTE_40	18	77193615	77208984	18q23	NFATC1	16	gain
3276	CGTE_40	19	281411	3959620	19p13.3	MIR6789,MKNK2,LOC100288123,MIER2,GAMT,PRSS57,THOP1,PALM,MUM1,SHC2,ADAMTSL5,GPX4,LSM7,MBD3,PCSK4,S1PR4,GZMM,CACTIN,R3HDM4,POLR2E,CELF5,TBXA2R,PRTN3,ZNF555,POLRMT,AES,MATK,DIRAS1,ELANE,ZNF556,ABHD17A,FGF22,MIR7850,ODF3L2,TLE6,RAX2,EFNA2,JSRP1,DOT1L,MIR1909,OAZ1,PTBP1,C2CD4C,AZU1,CIRBP-AS1,PLPPR3,THEG,GNA15,CDC34,GIPC3,ADAT3,HMG20B,NMRK2,ARID3A,ZNF77,FSTL3,MFSD12,PLPP2,MADCAM1,SGTA,STK11,SMIM24,KLF16,BSG,C19orf24,PIP5K1C,IZUMO4,ABCA7,LOC100996351,ONECUT3,NDUFS7,GADD45B,ATP8B3,NFIC,LMNB2,SPPL2B,MOB3A,AP3D1,FZR1,HCN2,ATCAY,MIR7108,REEP6,KISS1R,NCLN,TPGS1,ZNF554,GRIN3B,C19orf25,CSNK1G2-AS1,DAZAP1,HMHA1,GNG7,CACTIN-AS1,C19orf35,RNU6-2,CSNK1G2,MIR1227,PLEKHJ1,MED16,REXO1,LINGO3,CFD,UQCR11,DOHH,ZFR2,ZNF57,CIRBP,CBARP,GNA11,MIDN,MEX3D,RNF126,DAPK3,TCF3,APBA3,TLE2,TMEM259,PLK5,TMPRSS9,TIMM13,MIR3187,SBNO2,MRPL54,AMH,CNN2,SCAMP4,MISP,RPS15,WDR18,MIR4745,MIR1268A,APC2,BTBD2,ATP5D,SF3A2,C19orf71,TJP3,MIR4321,SLC39A3	0	loss
3277	CGTE_40	20	60891582	60910360	20q13.33	MIR4758,LAMA5	0	loss
3278	CGTE_40	21	45565244	46057457	21q22.3	KRTAP10-6,KRTAP10-3,KRTAP10-9,TRPM2-AS,AIRE,C21orf33,LRRC3-AS1,ICOSLG,DNMT3L,KRTAP10-7,KRTAP10-8,C21orf2,TSPEAR-AS2,TRPM2,KRTAP10-4,KRTAP10-2,PFKL,KRTAP10-10,KRTAP10-5,TSPEAR-AS1,TSPEAR,LRRC3,KRTAP10-1	1	loss
3279	CGTE_40	21	46057516	46067022	21q22.3	KRTAP10-10,TSPEAR,KRTAP10-11	9	gain
3280	CGTE_40	22	17063382	20708975	22q11.1-q11.21	MIR6816,LOC284865,RANBP1,MIR1306,C22orf29,DGCR14,BCL2L13,CECR5-AS1,SLC25A18,DGCR11,LINC00895,USP18,DGCR8,TPTEP1,LOC100996342,DGCR5,DGCR10,FLJ41941,CECR6,GAB4,CECR1,DGCR6,LINC00896,HIRA,CECR7,GSC2,TXNRD2,MIR1286,TBX1,LOC101929372,SEPT5,CCDC188,SLC25A1,ANKRD62P1-PARP4P3,ZDHHC8,BID,SEPT5-GP1BB,COMT,CLTCL1,CCT8L2,GGT3P,TUBA8,HSFY1P1,RTN4R,PRODH,PI4KAP1,GNB1L,TRMT2A,TSSK2,LINC00528,RIMBP3,UFD1L,GP1BB,MIR4761,LINC01311,CECR3,PEX26,MICAL3,MIR648,DGCR6L,ATP6V1E1,ARVCF,CDC45,C22orf39,DGCR9,CECR2,CLDN5,CECR5,MIR185,MIR3198-1,XKR3,DGCR2,IL17RA,MRPL40,TMEM191B,MIR3618,TANGO2	1	loss
3281	CGTE_40	22	20734915	21304136	22q11.21	KLHL22,TMEM191A,MED15,SNAP29,POM121L4P,PI4KA,SCARF2,CRKL,ZNF74,SERPIND1	28	gain
3282	CGTE_40	22	21311502	21328752	22q11.21	AIFM3,LOC101928891	0	loss

3283	CGTE_40	22	21328758	22024738	22q11.21	TMEM191C,THAP7,YDJC,RIMBP3C,P2RX6,UBE2L3,LRRC74B,FAM230B,PI4KAP2,TUBA3FP,MIR649,SDF2L1,AIFM3,LZTR1,SLC7A4,THAP7-AS1,CCDC116,PPIL2,RIMBP3B,MIR130B,POM121L8P,BCRP2,HIC2,P2RX6P,MIR301B	12	gain
3284	CGTE_40	22	22024834	23613798	22q11.22-q11.23	YPEL1,MIR5571,GNAZ,LL22NC03-63E9.3,RAB36,ZNF280A,MAPK1,GGTLC2,TOP3B,PPIL2,VPREB1,BMS1P20,ZNF280B,BCR,PPM1F,POM121L1P,IGLL5,RSPH14,PRAME,FBXW4P1,MIR650	8	gain
3285	CGTE_40	22	29438359	29706582	22q12.1-q12.2	C22orf31,GAS2L1,RHBDD3,KREMEN1,EMID1,ZNRF3,EWSR1	8	gain
3286	CGTE_40	22	29706587	29710030	22q12.2	GAS2L1,RASL10A	0	loss
3287	CGTE_40	22	29710963	29928039	22q12.2	RASL10A,RFPL1S,AP1B1,MIR3653,SNORD125,NEFH,RFPL1,THOC5	8	gain
3288	CGTE_40	22	50609165	51178618	22q13.33	SBF1,DENND6B,TYMP,ARSA,CHKB-AS1,SELO,PPP6R2,LMF2,MIOX,ODF3B,CHKB,HDAC10,MAPK12,CHKB-CPT1B,CPT1B,MAPK11,SCO2,PANX2,KLHDC7B,SHANK3,TUBGCP6,TRABD,SYCE3,MAPK8IP2,ADM2,NCAPH2,ACR,PLXNB2	0	loss
3289	CGTE_40	X	152913426	152915194	Xq28	DUSP9	10	gain
3290	CGTE_40	X	152915382	153051781	Xq28	PNCK,ABCD1,SLC6A8,PLXNB3,BCAP31,IDH3G,DUSP9,SRPK3	0	loss
3291	CGTE_40	X	153688885	153690623	Xq28	PLXNA3	5	gain
3292	CGTE_40	X	153691606	153697115	Xq28	PLXNA3	0	loss
3293	CGTE_41	1	861266	1585764	1p36.33	TNFRSF18,ACAP3,AURKAIP1,RNF223,TTLL10,CPSF3L,MIR6808,MIR200A,ATAD3B,LOC102724312,MIR429,VWA1,MMP23B,DVL1,TMEM240,SDF4,MRPL20,B3GALT6,TAS1R3,SSU72,ANKRD65,MXRA8,C1orf159,ATAD3C,KLHL17,FAM132A,MMP23A,SCNN1D,PERM1,TNFRSF4,MIB2,C1orf233,NOC2L,CPTP,TMEM88B,CCNL2,LINC01342,MIR6726,PUSL1,MIR6727,AGRN,LOC148413,ATAD3A,CDK11B,SAMD11,ISG15,MIR200B,HES4,UBE2J2,PLEKHN1	0	loss
3294	CGTE_41	1	3383677	3391363	1p36.32	ARHGEF16	4	gain
3295	CGTE_41	1	3392506	3422105	1p36.32	MEGF6,ARHGEF16	0	loss
3296	CGTE_41	2	233194365	233321475	2q37.1	ALPP,ALPI,ALPPL2,ECEL1P2,DIS3L2	3	gain
3297	CGTE_41	2	239006590	239059847	2q37.3	UBE2F-SCLY,SCLY,KLHL30,ESPNL	0	loss
3298	CGTE_41	2	241508080	241662245	2q37.3	RNPEPL1,AQP12B,CAPN10,KIF1A,AQP12A,CAPN10-AS1,GPR35	0	loss
3299	CGTE_41	2	241816287	241969819	2q37.3	LOC200772,C2orf54,SNED1,AGXT	0	loss
3300	CGTE_41	3	46722558	46723688	3p21.31	ALS2CL	176	gain
3301	CGTE_41	3	47042499	47049379	3p21.31	NBEAL2	3	gain
3302	CGTE_41	3	48510005	48511291	3p21.31	SHISA5	6	gain
3303	CGTE_41	3	52553409	52558572	3p21.1	NT5DC2,STAB1	8	gain
3304	CGTE_41	3	52558576	52559347	3p21.1	NT5DC2	0	loss
3305	CGTE_41	3	126193786	126207145	3q21.3	UROC1,ZXDC	7	gain
3306	CGTE_41	3	148777122	148778694	3q24	HLTF	20	gain
3307	CGTE_41	3	193366471	193382833	3q29	OPA1	3	gain
3308	CGTE_41	4	1806029	1809470	4p16.3	FGFR3	0	loss
3309	CGTE_41	5	1880833	3600446	5p15.33	C5orf38,LINC01377,CTD-2194D22.4,LINC01017,LOC100506858,IRX1,IRX2,LINC01019,IRX4	0	loss
3310	CGTE_41	5	140174526	140264088	5q31.3	PCDHA6,PCDHA10,PCDHA13,PCDHA7,PCDHA11,PCDHA1,PCDHA3,PCDHA9,PCDHA5,PCDHA12,PCDHA4,PCDHA2,PCDHA8	1	loss
3311	CGTE_41	5	172386735	172421778	5q35.1	RPL26L1,ATP6V0E1	6	gain
3312	CGTE_41	5	176859739	176860869	5q35.3	GRK6	5	gain
3313	CGTE_41	5	176860965	176919698	5q35.3	PRR7,DBN1,PDLIM7,GRK6,PRR7-AS1	0	loss
3314	CGTE_41	6	13591744	13595834	6p23	SIRT5	11	gain
3315	CGTE_41	6	33131385	33136735	6p21.32	COL11A2	3	gain
3316	CGTE_41	6	34003308	34027119	6p21.31	GRM4	4	gain
3317	CGTE_41	6	80749871	80752036	6q14.1	TTK	5	gain
3318	CGTE_41	6	126298763	126319529	6q22.32	TRMT11,HINT3	8	gain

3319	CGTE_41	6	134373515	134436545	6q23.2	HMGA1P7,SLC2A12	7	gain
3320	CGTE_41	7	100033406	100285885	7q22.1	PCOLCE-AS1,LRCH4,PPP1R35,GNB2,FBXO24,MOSPD3,NYAP1,GIGYF1,AGFG2,C7orf61,SAP25,TFR2,PCOLCE,ZASP,ACTL6B,TSC22D4	1	loss
3321	CGTE_41	8	144240163	145138426	8q24.3	MAFA,NAPRT,PUF60,ZFP41,RHPN1,FAM83H-AS1,PARP10,MIR6845,PLEC,MIR6846,RHPN1-AS1,MIR661,GRINA,LY6H,BREA2,ZNF696,TSTA3,MROH6,MAFA-AS1,MIR6847,GLI4,NRBP2,EXOSC4,TIGD5,ZNF623,MIR937,MIR4664,TOP1MT,MAPK15,EPPK1,CCDC166,PYCRL,MINCR,GPIHBP1,FAM83H,GSDMD,ZNF707,GPAA1,SPATC1,OPLAH,ZC3H3,EEF1D,SCRIB	0	loss
3322	CGTE_41	8	145138598	145140502	8q24.3	GPAA1	6	gain
3323	CGTE_41	9	139230277	139252616	9q34.3	GPSM1	0	loss
3324	CGTE_41	9	139378808	139916049	9q34.3	LCN8,LCN6,LCN12,LCN10,SNORA17A,NOTCH1,MIR4673,PHPT1,LCNL1,MIR4292,C9orf172,FBXW5,FAM69B,C9orf142,MIR126,MIR4479,LCN15,SNORA17B,MIR4674,SNHG7,RABL6,EGFL7,TMEM141,CCDC183,C8G,ABCA2,CLIC3,EDF1,TRAF2,AGPAT2,MIR6722,C9orf163,LOC100128593,NALT1,CCDC183-AS1,PTGDS,MAMDC4	0	loss
3325	CGTE_41	9	139916244	139918760	9q34.3	ABCA2	7	gain
3326	CGTE_41	9	139922497	140344192	9q34.3	ENTPD8,C9orf173-AS1,LRRC26,EXD3,C9orf139,NRARP,TOR4A,FAM166A,SAPCD2,DPP7,SLC34A3,SSNA1,NSMF,TMEM203,NELFB,NOXA1,RNF208,MAN1B1-AS1,FUT7,ENTPD2,TPRN,RNF224,UAP1L1,TUBB4B,MIR3621,MAN1B1,TMEM210,C9orf173,ANAPC2,NDOR1,CYSRT1,GRIN1,ABCA2,NPDC1	0	loss
3327	CGTE_41	10	134915762	134940873	10q26.3	ADGRA1	5	gain
3328	CGTE_41	10	134941652	135020329	10q26.3	ADGRA1,KNDC1	0	loss
3329	CGTE_41	11	193051	609691	11p15.5	SIRT3,PSMD13,ATHL1,SCGB1C2,LMNTD2,NLRP6,HRAS,LOC143666,SIGIRR,RIC8A,B4GALNT4,RNH1,IFITM2,IFITM3,PTDSS2,PKP3,SCGB1C1,LRRC56,MIR6743,PHRF1,MIR210,ANO9,RASSF7,IFITM1,IFITM5,ODF3,MIR210HG,BET1L	1	loss
3330	CGTE_41	11	610111	611160	11p15.5	PHRF1	6	gain
3331	CGTE_41	11	611582	621675	11p15.5	CDHR5,IRF7,PHRF1	0	loss
3332	CGTE_41	11	1023920	1080614	11p15.5	MUC2,MUC6	0	loss
3333	CGTE_41	11	1103818	1263783	11p15.5	MUC5B,MUC2,MUC5AC	0	loss
3334	CGTE_41	11	1272346	1274240	11p15.5	MUC5B	6	gain
3335	CGTE_41	11	1275302	2972586	11p15.5-p15.4	DUSP8,KRTAP5-2,TNNT3,LSP1,PHLDA2,CTSD,ASCL2,KRTAP5-3,MRPL23-AS1,TSSC4,SLC22A18,TOLLIP,KRTAP5-AS1,KRTAP5-1,KRTAP5-4,INS-IGF2,MIR7847,C11orf21,SLC22A18AS,FAM99B,LINC01219,IGF2,SYT8,INS,TRPM5,MOB2,IGF2-AS,KRTAP5-5,LINC01150,TOLLIP-AS1,MIR4686,MIR4298,MIR6744,FAM99A,KCNQ1-AS1,KRTAP5-6,TSPAN32,MIR483,MIR675,IFITM10,NAP1L4,KCNQ1,H19,HOTS,CDKN1C,SNORD131,KCNQ1OT1,MRPL23,BRSK2,TH,TNNI2,CD81-AS1,KCNQ1DN,CD81,MUC5B	0	loss
3336	CGTE_41	11	64083180	64139065	11q13.1	MIR7155,RPS6KA4,MIR1237,CCDC88B,ESRRA,TRMT112,PRDX5	0	loss
3337	CGTE_41	11	64216947	64323730	11q13.1	LOC100996455,SLC22A11	6	gain
3338	CGTE_41	11	64825752	64834550	11q13.1	NAALADL1	6	gain
3339	CGTE_41	11	67160919	67164800	11q13.2	RAD9A	4	gain
3340	CGTE_41	11	67164802	68154169	11q13.2	NDUFS8,PTPRCAP,MIR7113,UNC93B1,NUDT8,CDK2AP2,AIP,PITPNM1,PPP1CA,ACY3,CORO1B,C11orf24,GSTP1,LRP5,MIR6753,CABP2,CARNS1,RAD9A,MIR4691,ALDH3B1,TBX10,MIR6752,TMEM134,DOC2GP,RPS6KB2,TBC1D10C,CABP4,KMT5B,ALDH3B2,CHKA,TCIRG1,NDUFV1,C11orf72,GPR152,FAM86C2P	1	loss
3341	CGTE_41	11	68157351	68193660	11q13.2	LRP5	3	gain

3342	CGTE_41	11	124766066	124767808	11q24.2	ROBO4	8	gain
3343	CGTE_41	12	122212749	122213828	12q24.31	TMEM120B	10	gain
3344	CGTE_41	12	124779984	124873314	12q24.31	FAM101A,ZNF664-FAM101A,NCOR2,MIR6880	0	loss
3345	CGTE_41	12	132504556	132505835	12q24.33	EP400	7	gain
3346	CGTE_41	12	133144042	133148071	12q24.33	FBRSL1	24	gain
3347	CGTE_41	13	21179076	21200006	13q12.11	IFT88	7	gain
3348	CGTE_41	13	25404580	25416301	13q12.12	RNF17	18	gain
3349	CGTE_41	13	60565265	60590161	13q21.2	DIAPH3-AS1,DIAPH3	5	gain
3350	CGTE_41	13	98640209	98642530	13q32.2	IPO5	6	gain
3351	CGTE_41	13	109793592	110438412	13q33.3-q34	IRS2,MYO16-AS1,LINC00399,MYO16,LINC00676	1	loss
3352	CGTE_41	14	105057729	105412546	14q32.33	ZBTB42,INF2,SIVA1,TMEM179,PLD4,AKT1,ADSSL1,AHNAK2,CEP170B,MIR4710,LINC00638	0	loss
3353	CGTE_41	14	105412779	105416196	14q32.33	AHNAK2	5	gain
3354	CGTE_41	15	42139373	42167033	15q15.1	PLA2G4B,MIR4310,JMJD7-PLA2G4B,SPTBN5	0	loss
3355	CGTE_41	15	42167050	42168554	15q15.1	SPTBN5	7	gain
3356	CGTE_41	16	97450	2304119	16p13.3	NUBP2,MIR1225,CACNA1H,PRR35,HN1L,HBA1,SPSB3,DECR2,NOXO1,FAM195A,CASKIN1,TPSB2,UNKL,SNRNP25,LMF1-AS1,TPSG1,UBE2I,MAPK8IP3,LOC100134368,FAHD1,PRR25,FBXL16,POLR3K,MSLN,TSR3,CRAMP1,SNORA64,SNORD60,IFT140,MPG,SNHG19,PIGQ,JMJD8,MIR3177,NME3,ZNF598,CHTF18,NDUFB10,MIR662,NPRL3,TMEM204,PTX4,MLST8,FAM234A,NHLRC4,TPSAB1,LUC7L,TMEM8A,STUB1,EME2,LINC00254,PDIA2,METRN,MIR6511B2,SYNGR3,LINC00235,RPL3L,GNG13,ECI1,CCDC78,HBM,MIR4516,MEIOB,SNORA78,IGFALS,BAIAP3,MIR6511B1,TRAF7,GFER,TELO2,CCDC154,RNPS1,RAB11FIP3,GNPTG,MRPS34,C16orf91,HBA2,WDR24,MSRB1,C16orf13,SOX8,SNHG9,RHBDL1,CAPN15,RNF151,C1QTNF8,RHOT2,MIR3176,DNASE1L2,HBZ,BRICD5,RGS11,HAGHL,SNORA10,TPSD1,FAM173A,NME4,SSTR5-AS1,HS3ST6,HAGH,WDR90,RAB40C,RAB26,NPW,E4F1,RHBDF1,TSC2,RPS2,PGP,AXIN1,MRPL28,ARHGDIG,MIR3180-5,RPUSD1,WFIKKN1,SSTR5,MIR5587,HBQ1,NARFL,TBL3,PKD1,LMF1,SLC9A3R2,CLCN7,NTHL1	0	loss
3357	CGTE_41	17	79092145	79170825	17q25.3	MIR338,CEP131,MIR3065,AATK,MIR657,AATK-AS1,MIR1250	0	loss
3358	CGTE_41	17	80013620	80051264	17q25.3	GPS1,SNORD134,FASN,DUS1L	0	loss
3359	CGTE_41	19	281411	1257277	19p13.3	CFD,CDC34,GPX4,HMHA1,RNU6-2,SHC2,PALM,MIR3187,WDR18,ABCA7,ODF3L2,MIR4745,AZU1,PRTN3,GRIN3B,PLPPR3,CNN2,RNF126,TMEM259,PRSS57,SBNO2,THEG,KISS1R,GZMM,MIDN,POLRMT,TPGS1,FGF22,FSTL3,POLR2E,HCN2,BSG,ELANE,ARID3A,PLPP2,PTBP1,STK11,MADCAM1,R3HDM4,ATP5D,MISP,MIER2,MED16,CBARP,C2CD4C	0	loss
3360	CGTE_41	19	1390911	2042872	19p13.3	MEX3D,ONECUT3,CSNK1G2,ABHD17A,NDUFS7,REXO1,MBD3,PCSK4,MKNK2,MIR1909,TCF3,GAMT,UQCR11,KLF16,LOC100288123,ADAMTSL5,PLK5,DAZAP1,BTBD2,CSNK1G2-AS1,REEP6,APC2,ADAT3,SCAMP4,RPS15,C19orf25,ATP8B3	0	loss
3361	CGTE_41	19	2214331	2456773	19p13.3	SPPL2B,SF3A2,TMPRSS9,OAZ1,MIR4321,C19orf35,DOT1L,LINGO3,LMNB2,LSM7,MIR7108,AMH,TIMM13,MIR1227,MIR6789,PLEKHJ1,JSRP1	0	loss
3362	CGTE_41	19	3178819	3611339	19p13.3	CELF5,NFIC,S1PR4,GIPC3,SMIM24,MFSD12,FZR1,HMG20B,CACTIN-AS1,TBXA2R,CACTIN,NCLN,C19orf71,DOHH	0	loss
3363	CGTE_41	19	3611653	3614594	19p13.3	CACTIN,CACTIN-AS1	12	gain

3364	CGTE_41	19	3618867	4212716	19p13.3	NMRK2,ZFR2,SNORD37,ANKRD24,PIP5K1C,TJP3,CREB3L3,MAP2K2,MATK,ZBTB7A,MRPL54,PIAS4,DAPK3,EEF2,APBA3,MIR1268A,ATCAY,CACTIN,MIR637,SIRT6,RAX2	1	loss
3365	CGTE_41	19	4212727	4217438	19p13.3	ANKRD24	4	gain
3366	CGTE_41	20	21494396	23030432	20p11.22-p11.21	LOC284788,LINC00261,THBD,LINC01427,PAX1,NKX2-2,LINC01432,LOC101929608,SSTR4,FOXA2,LINC01384,LOC101929625	0	loss
3367	CGTE_41	20	60884380	60937710	20q13.33	LAMA5-AS1,MIR4758,LAMA5	0	loss
3368	CGTE_41	20	61939865	61981794	20q13.33	CHRNA4,COL20A1	0	loss
3369	CGTE_41	20	62128921	62293905	20q13.33	PTK6,SRMS,PPDPF,RTEL1,C20orf195,GMEB2,HELZ2,STMN3,EEF1A2,RTEL1-TNFRSF6B,LOC100505771	0	loss
3370	CGTE_41	20	62293910	62316955	20q13.33	RTEL1,RTEL1-TNFRSF6B	3	gain
3371	CGTE_41	20	62317058	62322371	20q13.33	RTEL1-TNFRSF6B,RTEL1	0	loss
3372	CGTE_41	21	45970688	45977987	21q22.3	TSPEAR,KRTAP10-2,KRTAP10-3	18	gain
3373	CGTE_41	21	45978159	46057457	21q22.3	KRTAP10-10,KRTAP10-8,KRTAP10-9,KRTAP10-3,TSPEAR,KRTAP10-6,KRTAP10-4,KRTAP10-7,KRTAP10-5	0	loss
3374	CGTE_41	21	46057516	46074404	21q22.3	TSPEAR,KRTAP10-11,KRTAP12-4,KRTAP10-10	9	gain
3375	CGTE_41	21	47531374	47536771	21q22.3	COL6A2	6	gain
3376	CGTE_41	21	47537235	47540582	21q22.3	COL6A2	0	loss
3377	CGTE_41	22	50718930	50946884	22q13.33	SBF1,MIOX,ADM2,LMF2,DENND6B,PPP6R2,NCAPH2,PLXNB2	0	loss
3378	CGTE_41	X	12994450	13056704	Xp22.2	TMSB4X,FAM9C	10	gain
3379	CGTE_41	X	30709284	30712746	Xp21.2	GK	13	gain
3380	CGTE_41	X	47486113	47489351	Xp11.23	CFP	6	gain
3381	CGTE_41	X	53253865	53265053	Xp11.22	IQSEC2,KDM5C	0	loss
3382	CGTE_41	X	72667067	72899264	Xq13.2	CHIC1,MAP2K4P1,CDX4	4	gain
3383	CGTE_41	X	83401983	83442937	Xq21.1	RPS6KA6	5	gain
3384	CGTE_41	X	106110009	106114056	Xq22.3	TBC1D8B	9	gain
3385	CGTE_41	X	114399972	114468488	Xq23	RBMXL3,LRCH2	0	loss
3386	CGTE_41	X	152806771	152807402	Xq28	ATP2B3	5	gain
3387	CGTE_41	X	152864479	152915194	Xq28	LOC105373383,FAM58A,DUSP9	7	gain
3388	CGTE_41	X	152937334	153043159	Xq28	SLC6A8,PLXNB3,ABCD1,PNCK,BCAP31	0	loss
3389	CGTE_41	X	153043399	153045904	Xq28	PLXNB3	6	gain
3390	CGTE_41	X	153046586	153050632	Xq28	SRPK3	0	loss
3391	CGTE_41	X	153216772	153220569	Xq28	HCFC1	0	loss
3392	CGTE_41	X	153678337	153690623	Xq28	MIR6858,PLXNA3,FAM50A	4	gain
3393	CGTE_41	X	153691606	153697115	Xq28	PLXNA3	0	loss
3394	CGTE_42	1	17281174	17281880	1p36.13	CROCC	10	gain
3395	CGTE_42	1	19992034	19992925	1p36.13	HTR6	4	gain
3396	CGTE_42	1	26487799	26496630	1p36.11	FAM110D,ZNF593	5	gain
3397	CGTE_42	1	50886633	50887388	1p32.3	DMRTA2	6	gain
3398	CGTE_42	1	76081458	76190555	1p31.1	ACADM	5	gain
3399	CGTE_42	1	181057849	181058958	1q25.3	IER5	4	gain
3400	CGTE_42	1	228289680	228290983	1q42.13	C1orf35	12	gain
3401	CGTE_42	1	234040726	234350401	1q42.2	SLC35F3	5	gain
3402	CGTE_42	2	9731439	9983721	2p25.1	TAF1B,YWHAQ	7	gain
3403	CGTE_42	2	16082129	16083029	2p24.3	MYCN	4	gain
3404	CGTE_42	2	27593013	27593834	2p23.3	EIF2B4,SNX17	6	gain
3405	CGTE_42	2	73495801	73496753	2p13.2	FBXO41	4	gain
3406	CGTE_42	2	87016597	87018114	2p11.2	CD8A	6	gain
3407	CGTE_42	2	171608341	171627719	2q31.1	ERICH2,LOC101926913	12	gain
3408	CGTE_42	2	182543546	182757409	2q31.3	NEUROD1,SSFA2	7	gain
3409	CGTE_42	3	138664424	138739537	3q23-q22.3	FOXL2,PRR23B,FOXL2NB,PRR23A	4	gain

3410	CGTE_42	4	54930191	54966932	4q12	GSX2,CHIC2	3	gain
3411	CGTE_42	5	10649461	10650237	5p15.2	ANKRD33B	10	gain
3412	CGTE_42	5	14487522	14488599	5p15.2	TRIO	7	gain
3413	CGTE_42	5	72742711	72794634	5q13.2	FOXD1,LINC01386,BTF3	4	gain
3414	CGTE_42	5	122372167	122426343	5q23.2	PPIC,PRDM6	5	gain
3415	CGTE_42	5	140167086	140263292	5q31.3	PCDHA2,PCDHA3,PCDHA1,PCDHA11,PCDHA7,PCDHA4,PCDHA12,PCDHA10,PCDHA5,PCDHA13,PCDHA6,PCDHA9,PCDHA8	3	gain
3416	CGTE_42	5	140263492	140264088	5q31.3	PCDHA10,PCDHA12,PCDHA5,PCDHA8,PCDHA6,PCDHA13,PCDHA9,PCDHA11,PCDHA1,PCDHA2,PCDHA3,PCDHA4,PCDHA7	9	gain
3417	CGTE_42	5	140799042	140812605	5q31.3	PCDHGB5,PCDHGA1,PCDHGA6,PCDHGB4,PCDHGA5,PCDHGB7,PCDHGB1,PCDHGA8,PCDHGA10,PCDHGA2,PCDHGA11,PCDHGA3,PCDHGB6,PCDHGB8P,PCDHGA9,PCDHGB3,PCDHGA4,PCDHGB2,PCDHGA7,PCDHGA12	3	gain
3418	CGTE_42	5	170735324	170737270	5q35.1	TLX3	5	gain
3419	CGTE_42	6	26017358	26252277	6p22.2	HIST1H1C,HIST1H2BD,HIST1H4A,HIST1H2AC,HIST1H4G,HIST1H3D,HIST1H1T,HIST1H2AB,HIST1H4F,HIST1H2BB,HIST1H4C,HIST1H4E,HIST1H2AD,HIST1H2AE,HIST1H3B,HIST1H3F,HIST1H3A,HIST1H2BH,HIST1H1D,HIST1H4D,HFE,HIST1H1E,HIST1H2BE,HIST1H1A,HIST1H2BC,HIST1H2BF,HIST1H3C,HIST1H4B,HIST1H3E,HIST1H2BG	3	gain
3420	CGTE_42	6	29795542	29796392	6p22.1	HLA-G	6	gain
3421	CGTE_42	6	31238987	31324231	6p21.33	HLA-B,MIR6891,HLA-C	3	gain
3422	CGTE_42	6	31324289	31371612	6p21.33	HLA-B,MICA	4	gain
3423	CGTE_42	6	157099101	157100701	6q25.3	ARID1B	0	loss
3424	CGTE_42	7	1543466	1544271	7p22.3	INTS1	6	gain
3425	CGTE_42	7	5569184	5633203	7p22.1	FSCN1,ACTB	39	gain
3426	CGTE_42	7	26192097	26192768	7p15.2	NFE2L3	17	gain
3427	CGTE_42	7	27238756	27239757	7p15.2	HOXA13	39	gain
3428	CGTE_42	7	29605904	29923577	7p14.3	PRR15,ZNRF2P2,LOC646762,MIR550A3,DPY19L2P3,WIPF3	3	gain
3429	CGTE_42	7	43946062	43967798	7p13	UBE2D4,URGCP,URGCP-MRPS24	5	gain
3430	CGTE_42	7	44923990	44924956	7p13	PURB	5	gain
3431	CGTE_42	7	73038481	73084437	7q11.23	MLXIPL,VPS37D	3	gain
3432	CGTE_42	7	105925227	106301346	7q22.3	CCDC71L,NAMPT	6	gain
3433	CGTE_42	7	139477394	139478387	7q34	HIPK2,TBXAS1	8	gain
3434	CGTE_42	7	150783817	150784315	7q36.1	AGAP3	7	gain
3435	CGTE_42	8	10583649	10588092	8p23.1	SOX7	6	gain
3436	CGTE_42	8	37553489	37555902	8p11.23	ZNF703	0	loss
3437	CGTE_42	8	37822857	37963281	8p11.23	ADRB3,ASH2L,EIF4EBP1	3	gain
3438	CGTE_42	8	54791774	54852510	8q11.23	RGS20	4	gain
3439	CGTE_42	8	141468396	141522193	8q24.3	TRAPPC9,CHRAC1	4	gain
3440	CGTE_42	9	138378655	138379511	9q34.3	PPP1R26	5	gain
3441	CGTE_42	9	138391467	138392982	9q34.3	MRPS2,C9orf116	5	gain
3442	CGTE_42	9	139333168	139333910	9q34.3	INPP5E	204	gain
3443	CGTE_42	10	35896546	35929717	10p11.21	FZD8,GJD4	4	gain
3444	CGTE_42	10	76994622	77159485	10q22.2	ZNF503,COMTD1,ZNF503-AS1	5	gain
3445	CGTE_42	11	720626	721390	11p15.5	EPS8L2	0	loss
3446	CGTE_42	11	17742805	17757750	11p15.1	KCNC1,MYOD1	6	gain
3447	CGTE_42	11	66056398	66063236	11q13.2	YIF1A,TMEM151A	6	gain
3448	CGTE_42	12	40498899	40619162	12q12	LRRK2,SLC2A13	6	gain
3449	CGTE_42	12	53471856	53491795	12q13.13	IGFBP6,SPRYD3	7	gain
3450	CGTE_42	12	63195555	64061946	12q14.2	DPY19L2,AVPR1A,PPM1H	3	gain
3451	CGTE_42	13	21750515	21948609	13q12.11	SKA3,LINC01046,MRPL57,LINC00539,ZDHHC20,MIPEPP3	3	gain
3452	CGTE_42	13	28366835	28498721	13q12.2	PDX1-AS1,PDX1,GSX1	3	gain

3453	CGTE_42	13	28674553	28713255	13q12.2	PAN3-AS1,PAN3,FLT3	4	gain
3454	CGTE_42	13	38934079	39262135	13q13.3	FREM2,UFM1,LINC00437,LINC00366	3	gain
3455	CGTE_42	13	52004448	52026733	13q14.3	INTS6	4	gain
3456	CGTE_42	13	53419777	53421145	13q14.3	PCDH8	3	gain
3457	CGTE_42	13	78235542	78272705	13q22.3	SLAIN1,MIR3665,LOC100129307	6	gain
3458	CGTE_42	13	95248187	95254357	13q32.1	TGDS,GPR180	3	gain
3459	CGTE_42	13	96684081	96743252	13q32.1	UGGT2,HS6ST3	4	gain
3460	CGTE_42	13	100637323	100638366	13q32.3	ZIC2	3	gain
3461	CGTE_42	13	110434503	110436284	13q34	IRS2	0	loss
3462	CGTE_42	13	110436287	110438412	13q34	IRS2	23	gain
3463	CGTE_42	14	36986429	36988419	14q13.3	NKX2-1	18	gain
3464	CGTE_42	14	50100386	50110441	14q21.3	DNAAF2,POLE2	13	gain
3465	CGTE_42	14	50154724	50160086	14q21.3	POLE2,KLHDC1	8	gain
3466	CGTE_42	14	70038179	70040340	14q24.1	CCDC177	0	loss
3467	CGTE_42	14	105941155	105946033	14q32.33	CRIP2	0	loss
3468	CGTE_42	15	48623320	48624688	15q21.1	DUT	8	gain
3469	CGTE_42	15	68119103	68121600	15q23	SKOR1	5	gain
3470	CGTE_42	15	80189109	80215612	15q25.1	MTHFS,ST20-AS1,ST20,ST20-MTHFS	3	gain
3471	CGTE_42	16	278923	284844	16p13.3	LUC7L,FAM234A	0	loss
3472	CGTE_42	16	857493	863949	16p13.3	PRR25	6	gain
3473	CGTE_42	16	1660699	1664793	16p13.3	CRAMP1,IFT140	5	gain
3474	CGTE_42	16	67196921	67200080	16q22.1	HSF4,FBXL8	3	gain
3475	CGTE_42	17	7491714	7492935	17p13.1	SOX15	4	gain
3476	CGTE_42	17	39149959	39471948	17q21.2	KRTAP4-6,KRTAP4-7,KRTAP4-5,KRTAP9-7,KRTAP4-2,KRTAP17-1,KRTAP4-8,KRTAP9-4,KRTAP16-1,KRTAP1-5,KRTAP1-1,KRTAP29-1,KRTAP9-1,KRTAP4-1,KRTAP1-4,KRTAP9-8,KRTAP9-9,KRTAP3-2,KRTAP4-11,KRTAP4-4,KRTAP1-3,KRTAP9-2,KRTAP2-4,KRTAP9-3,KRTAP9-6,KRTAP2-2,KRTAP3-3,KRTAP2-1,KRTAP3-1,KRTAP4-9,KRTAP2-3,KRTAP4-3,KRTAP4-12	3	gain
3477	CGTE_42	17	42580496	42734142	17q21.31	MEIOC,LINC01180,GPATCH8,FZD2	3	gain
3478	CGTE_42	17	79095094	79096622	17q25.3	AATK	13	gain
3479	CGTE_42	17	79917309	79935624	17q25.3	ASPSCR1,NOTUM	0	loss
3480	CGTE_42	17	79987801	79990295	17q25.3	LRRC45,RAC3	5	gain
3481	CGTE_42	18	19751067	19757114	18q11.2	GATA6	26	gain
3482	CGTE_42	18	24127679	24128654	18q11.2	KCTD1	4	gain
3483	CGTE_42	18	51748740	51796246	18q21.2	SNORA37,POLI,MBD2	4	gain
3484	CGTE_42	19	913102	920564	19p13.3	R3HDM4,KISS1R	8	gain
3485	CGTE_42	19	18543973	18544680	19p13.11	SSBP4	74	gain
3486	CGTE_42	19	19368684	19369662	19p13.11	HAPLN4	5	gain
3487	CGTE_42	19	48183449	48198463	19q13.33	GLTSCR1	5	gain
3488	CGTE_42	19	48997307	49002628	19q13.33	LMTK3	9	gain
3489	CGTE_42	19	51601784	51607961	19q13.41	CTU1	6	gain
3490	CGTE_42	19	56125290	56128296	19q13.42	ZNF865	0	loss
3491	CGTE_42	19	58908008	58920735	19q13.43	RNF225,ZNF584	4	gain
3492	CGTE_42	20	18037279	18118800	20p11.23	OVOL2,PET117	5	gain
3493	CGTE_42	21	34399131	34442669	21q22.11	LINC00945,OLIG2,OLIG1	4	gain
3494	CGTE_42	22	20130396	20136478	22q11.21	ZDHHC8,CCDC188	10	gain
3495	CGTE_42	22	21212839	21213514	22q11.21	PI4KA,SNAP29	5	gain
3496	CGTE_42	22	21983160	21984303	22q11.21	YDJC	4	gain
3497	CGTE_42	22	38051369	38055620	22q13.1	PDXP,SH3BP1,LOC101927051	4	gain
3498	CGTE_42	22	39239086	39268019	22q13.1	NPTXR,CBX6	3	gain
3499	CGTE_42	X	11445482	11776999	Xp22.2	MSL3,ARHGAP6	3	gain

3500	CGTE_42	X	48689663	48693975	Xp11.23	PCSK1N	0	loss
3501	CGTE_42	X	152686435	152710636	Xq28	ZFP92,TREX2	4	gain
3502	CGTE_43	2	73339428	73430213	2p13.2	NOTO,RAB11FIP5	11	gain
3503	CGTE_43	2	175200794	175202321	2q31.1	SP9	0	loss
3504	CGTE_43	3	45187553	45267477	3p21.31	CDCP1,TMEM158	0	loss
3505	CGTE_43	4	88238199	88243979	4q22.1	HSD17B13	3	gain
3506	CGTE_43	6	29691247	29692020	6p22.1	HLA-F	6	gain
3507	CGTE_43	6	157099101	157100457	6q25.3	ARID1B	0	loss
3508	CGTE_43	7	6193575	6194505	7p22.1	USP42	0	loss
3509	CGTE_43	7	6691942	6692742	7p22.1	ZNF316	4	gain
3510	CGTE_43	8	37554602	37555902	8p11.23	ZNF703	0	loss
3511	CGTE_43	9	139739969	139741111	9q34.3	C9orf172	0	loss
3512	CGTE_43	12	54635512	54639926	12q13.13	CBX5	3	gain
3513	CGTE_43	13	21999695	22069460	13q12.11	MICU2,ZDHHC20	4	gain
3514	CGTE_43	13	22070140	22096833	13q12.11	MICU2	1	loss
3515	CGTE_43	13	45971350	45983198	13q14.13	SLC25A30	3	gain
3516	CGTE_43	13	110434503	110436284	13q34	IRS2	4	gain
3517	CGTE_43	14	70038876	70040340	14q24.1	CCDC177	3	gain
3518	CGTE_43	15	43691244	43699691	15q15.3	TP53BP1,TUBGCP4	3	gain
3519	CGTE_43	15	48807636	48813075	15q21.1	FBN1	1	loss
3520	CGTE_43	15	83735497	83776604	15q25.2	TM6SF1,BTBD1,MIR4515	0	loss
3521	CGTE_43	16	50186856	50188126	16q12.1	PAPD5	0	loss
3522	CGTE_43	16	75018785	75034014	16q23.1	WDR59,ZNRF1	1	loss
3523	CGTE_43	17	17046032	17050495	17p11.2	MPRIP	3	gain
3524	CGTE_43	17	36734703	36858597	17q12	C17orf96,SRCIN1,MIR4734	1	loss
3525	CGTE_43	17	79917309	79935624	17q25.3	ASPSCR1,NOTUM	4	gain
3526	CGTE_43	18	60052126	60191296	18q21.33	ZCCHC2,TNFRSF11A	0	loss
3527	CGTE_43	19	643193	646974	19p13.3	FGF22	1	loss
3528	CGTE_43	22	50636777	50640002	22q13.33	SELO,TRABD	4	gain
3529	CGTE_44	1	983567	985381	1p36.33	AGRN	0	loss
3530	CGTE_44	1	985576	986416	1p36.33	AGRN	5	gain
3531	CGTE_44	1	1007231	1018182	1p36.33	C1orf159,RNF223	6	gain
3532	CGTE_44	1	1141655	1148902	1p36.33	TNFRSF18,TNFRSF4	0	loss
3533	CGTE_44	1	17263187	17266650	1p36.13	CROCC	3	gain
3534	CGTE_44	1	27059147	27333608	1p36.11	GPN2,NR0B2,PIGV,SFN,ZDHHC18,FAM46B,ARID1A,KDF1,GPATCH3,TRNP1,NUDC	1	loss
3535	CGTE_44	1	40366379	40421261	1p34.2	MFSD2A,MYCL	6	gain
3536	CGTE_44	1	110754094	110754866	1p13.3	KCNC4	7	gain
3537	CGTE_44	1	228463390	228464436	1q42.13	OBSCN	5	gain
3538	CGTE_44	2	73052921	73114960	2p13.2	SPR,EXOC6B	6	gain
3539	CGTE_44	2	73491651	73492824	2p13.2	FBXO41	5	gain
3540	CGTE_44	2	74741195	74743597	2p13.1	TLX2	7	gain
3541	CGTE_44	2	219745728	219757656	2q35	WNT10A	3	gain
3542	CGTE_44	2	219757660	219825892	2q35	WNT10A,LINC01494,CDK5R2	6	gain
3543	CGTE_44	2	219857691	219868021	2q35	CRYBA2,MIR375,CFAP65,LOC100129175	4	gain
3544	CGTE_44	2	224462288	225265399	2q36.1-q36.2	WDFY1,MRPL44,SCG2,FAM124B,SERPINE2,AP1S3	1	loss
3545	CGTE_44	2	239037238	239040249	2q37.3	ESPNL	10	gain
3546	CGTE_44	2	242755629	242756421	2q37.3	NEU4	19	gain
3547	CGTE_44	3	9987907	9989646	3p25.3	PRRT3,PRRT3-AS1	0	loss
3548	CGTE_44	3	138725034	138739537	3q23	PRR23B,PRR23A	6	gain

3549	CGTE_44	3	163889172	169501389	3q26.1-q26.2	MYNN,ZBBX,LOC100507661,BCHE,SERPINI2,ACTRT3,LINC01330,LINC01322,MIR1263,LINC01327,MIR551B,EGFEM1P,TERC,SLITRK3,LINC01324,MECOM,GOLIM4,PDCD10,SERPINI1,SI,WDR49	3	gain
3550	CGTE_44	3	169501461	169525376	3q26.2	LRRC34,MYNN	5	gain
3551	CGTE_44	3	169539750	169899538	3q26.2	GPR160,LRRC31,PHC3,LRRIQ4,SEC62,SAMD7,LOC100128164	5	gain
3552	CGTE_44	3	169940471	169998232	3q26.2	PRKCI	4	gain
3553	CGTE_44	3	169999015	170584559	3q26.2	SKIL,PRKCI,CLDN11,MIR6828,SLC7A14,RPL22L1	5	gain
3554	CGTE_44	3	170585847	190930460	3q27.2-q26.2	LOC102724699,MIR548AY,LOC101928882,SPATA16,KCCAT211,P3H2,AHSG,IGF2BP2,DNAJB11,LOC344887,EHHADH-AS1,NAALADL2-AS1,AP2M1,MAGEF1,LOC100505609,DGKG,RTP1,LOC253573,DVL3,GMNC,KCNMB2-AS1,MIR569,MIR28,RNU6-2,NLGN1,PIK3CA,CLDN1,HTR3E-AS1,C3orf70,TPRG1-AS1,DCUN1D1,YEATS2,EPHB3,MRPL47,FETUB,LINC00888,SNORA4,FLJ42393,MIR944,RTP2,ECT2,NLGN1-AS1,ACTL6A,SOX2-OT,TP63,B3GNT5,DNAJC19,KLHL6,MIR5588,CHRD,NDUFB5,TPRG1,LIPH,HTR3E,MCCC1,TPRG1-AS2,GNB4,VPS8,RPL22L1,ADIPOQ,MASP1,IL1RAP,ABCF3,FLJ46066,MIR7977,MIR548AQ,MFN1,USP13,FAM131A,EIF5A2,HTR3D,ATP11B,KLHL6-AS1,LOC100131635,EIF4A2,SLC2A2,POLR2H,TBCCD1,LINC00578,NAALADL2,LINC00501,EHHADH,ETV5,ALG3,NCEH1,ECE2,RTP4,KCNMB2,LINC01209,TMEM41A,LAMP3,CRYGS,LPP-AS1,SNORA63,GHSR,TMEM212-AS1,SNORD66,LINC01206,LOC101928739,THPO,MCF2L2,LINC01208,LOC101928992,ZNF639,MIR4789,TBL1XR1,VWA5B2,SST,PLD1,SNAR-I,EIF2B5-AS1,CLCN2,HTR3C,BCL6,PARL,KNG1,TMEM207,P3H2-AS1,EIF2B5,ABCC5,MIR1248,TTC14,SOX2,SNORA81,RFC4,SNORD2,CLDN16,CAMK2N2,ABCC5-AS1,ADIPOQ-AS1,EIF4G1,LPP,MIR1224,KLHL24,TNIK,MAP3K13,PSMD2,CCDC39,MAP6D1,LOC102724604,KCNMB3,SENP2,HRG,TNFSF10,RPL39L,ZMAT3,NAALADL2-AS3,LOC101929106,PEX5L,LPP-AS2,NAALADL2-AS2,OSTN,FXR1,LINC01014,FNDC3B,TRA2B,TMEM212,ST6GAL1,IGF2BP2-AS1,PEX5L-AS2	3	gain
3555	CGTE_44	4	960751	961935	4p16.3	DGKQ	0	loss
3556	CGTE_44	4	995726	997473	4p16.3	IDUA	3	gain
3557	CGTE_44	4	1015856	1018445	4p16.3	FGFRL1	3	gain
3558	CGTE_44	4	1807041	1808710	4p16.3	FGFR3	3	gain
3559	CGTE_44	4	140201108	140217070	4q31.1	NDUFC1,MGARP	5	gain
3560	CGTE_44	4	183720522	183721509	4q35.1	TENM3	5	gain
3561	CGTE_44	5	139283827	139422514	5q31.2	NRG2	0	loss
3562	CGTE_44	5	140188239	140188988	5q31.3	PCDHA2,PCDHA4,PCDHA1,PCDHA3	4	gain
3563	CGTE_44	5	140228160	140229460	5q31.3	PCDHA8,PCDHA7,PCDHA3,PCDHA9,PCDHA2,PCDHA5,PCDHA4,PCDHA6,PCDHA1	3	gain
3564	CGTE_44	6	31324726	31371612	6p21.33	MICA,HLA-B	5	gain
3565	CGTE_44	6	43225455	43251342	6p21.1	TTBK1	3	gain
3566	CGTE_44	6	127836618	127837673	6q22.33	SOGA3	6	gain
3567	CGTE_44	7	195557	2685532	7p22.3	TTYH3,MIR4648,WI2-2373I1.2,SUN1,DNAAF5,MIR6836,LOC101927181,GPER1,LOC101927000,SNX8,FTSJ2,HRAT92,CHST12,BRAT1,FAM20C,CYP2W1,GET4,MIR4655,MAD1L1,GPR146,LOC442497,LOC101927021,LOC101926963,MIR339,TFAMP1,NUDT1,PDGFA,MAFK,PSMG3,IQCE,PRKAR1B,COX19,INTS1,C7orf50,PSMG3-AS1,LFNG,ADAP1,EIF3B,GRIFIN,UNCX,MICALL2,TMEM184A,ELFN1-AS1,ELFN1,ZFAND2A	3	gain

3568	CGTE_44	7	2686284	2689366	7p22.3	TTYH3	8	gain
3569	CGTE_44	7	2689426	4871912	7p22.2-p22.1	GNA12,MIR4656,AP5Z1,FOXK1,AMZ1,CARD11,TTYH3,SDK1,LOC100129603,RADIL	3	gain
3570	CGTE_44	7	4874185	4876257	7p22.1	RADIL	209	gain
3571	CGTE_44	7	4899512	44924497	7p22.1-p21.3	MIR148A,UBE2D4,THSD7A,DKFZP586I1420,FAM126A,EEPD1,PMS2CL,NEUROD6,EVX1-AS,ZMIZ2,SUGCT,OSBPL3,SNX10,PGAM2,ZNF12,UMAD1,ARL4A,NPY,POLM,KBTBD2,HOXA7,LINC01449,TAX1BP1,TMEM196,SCRN1,TWIST1,MYL7,ISPD,LOC100506178,GPNMB,FERD3L,EVX1,AIMP2,FBXL18,MIR4657,POLD2,HOXA2,POU6F2-AS1,GARS,LINC01448,LOC101927769,HDAC9,MACC1,TSPAN13,SNX13,CCDC129,C7orf26,RNF216-IT1,ZNRF2P2,PMS2,SPDYE1,DAGLB,MIR6874,ANKMY2,HOXA10,HOXA6,LOC101927391,MIR550A2,HOTTIP,DPY19L2P3,CYCS,ZNF815P,GLI3,CAMK2B,TMEM106B,DBNL,PER4,SP4,TBX20,BBS9,MEOX2,C1GALT1,TARP,DPY19L2P1,HOXA11-AS,DDX56,AOAH-IT1,TRA2A,ANLN,SFRP4,PLEKHA8,HOXA4,RAC1,CPVL,OGDH,TMED4,RNF216P1,PSMA2,FAM188B,SCIN,SNORD93,NUDCD3,SLC29A4,LINC00265,ZDHHC4,LOC101928618,YKT6,COL28A1,HOTAIRM1,ZNRF2,LINC01176,GHRHR,WIPF3,STK17A,NT5C3A,CCZ1,BLVRA,LOC101927354,NPSR1-AS1,IGF2BP3,DNAH11,RSPH10B2,NUPL2,RADIL,MIR6838,NME8,RPS2P32,TWISTNB,LOC101928168,AOAH,C7orf31,HOXA1,TRG-AS1,AGMO,PRR15,BMPER,POU6F2,KIAA0087,DPY19L1P2,C7orf71,LOC101927630,MEOX2-AS1,MIR6837,HOXA10-AS,LOC100130673,EPDR1,KDELR2,MIR3683,AGR2,ZNF316,ZNRF2P1,DFNA5,WIPI2,HOXA10-HOXA9,IL6,SOSTDC1,AGR3,JAZF1-AS1,VPS41,LOC221946,HERPUD2,RPA3,RNF216,MIR589,FKBP14,TNRC18,MPLKIP,LOC401324,NPVF,EIF2AK1,GCK,TSL,NFE2L3,LOC102724484,LOC541472,COA1,MIR1302-6,ICA1,FAM221A,USP42,CCZ1B,HOXA5,MIR1183,ISPD-AS1,PDE1C,HOXA3,HOXA-AS2,TOMM7,LOC441204,LRRC72,INMT,RSPH10B,KLHL7,HOXA11,MIR550A3,HOXA-AS3,CCDC126,KIAA0895,ADCYAP1R1,MIR3943,INHBA,MIR550B1,HECW1,MRPL32,DPY19L1,PRPS1L1,MIR1200,INMT-FAM188B,ITGB8,LINC00957,OCM,ETV1,LOC100505938,AMPH,ZNF890P,CREB5,LINC01450,MIR3146,LSM5,YAE1D1,SKAP2,LINC01162,CLK2P1,MRPS24,RBAK,HOXA9,URGCP-MRPS24,HNRNPA2B1,LOC100131257,MPP6,AEBP1,AVL9,SEPT7-AS1,NOD1,CHN2,SP8,FAM183BP,LOC100505921,CRHR2,LOC101927668,H2AFV,NPC1L1,INHBA-AS1,MMD2,MIR550A1,CYTH3,ELMO1-AS1,CBX3,LOC401312,TRIL,PURB,C7orf25,MIOS,NPSR1,POLR2J4,STARD3NL,LOC401320,STK31,LOC646762,NDUFA4,LOC100506725,ACTB,KLHL7-AS1,LINC00997,DGKB,CDK13,BZW2,URGCP,RPL23P8,MTURN,RP9,HOXA13,CDCA7L,DPY19L1P1,FSCN1,PPP1R17,ABCB5,RAPGEF5,AQP1,RBAK-RBAKDN,FKBP9,ANKRD61,MIR4649,MACC1-AS1,NXPH1,PHF14,MIR196B,RP9P,LOC100506895,PAPOLB,GPR141,LOC100506497,ELMO1,GRID2IP,JAZF1,LOC101927811,GGCT,PPIA,GLCCI1,STEAP1B,VWDE,HIBADH,KCCAT333,RASA4CP,MALSU1,ZNF853,SEPT7,AHR,FAM220A,RALA,RBAKDN,MIR550B2	3	gain
3572	CGTE_44	7	44924502	45002733	7p13	MYO1G,PURB	7	gain

3573	CGTE_44	7	45003455	72721809	7q11.22-p12.2	SNORA9,RABGEF1,PHKG1,LOC401357,TNS3,TYW1B,PSPH,ZNF713,LOC102723427,GTF2IRD1P1,HPVC1,C7orf65,CCT6A,LINC01447,ASL,MIR3914-2,SEPT7P2,ZNF117,WBSCR17,GTF2IP4,CRCP,LINC00525,MIR4283-1,LOC441242,ELDR,VWC2,STAG3L4,UPP1,RAMP3,IKZF1,ERV3-1,YWHAEP1,LOC100287834,ADCY1,LOC100996654,NUPR2,SNORA5C,IGFBP1,PKD1L1,POM121L12,STAG3L3,MIR4650-2,C7orf72,VKORC1L1,SBDS,SNHG15,LINC01446,GRB10,LINC01005,GTF2IP1,DKFZp434L192,NCF1B,TYW1,SNORA15,MIR4650-1,GUSB,CCT6P1,SBDSP1,ABCA13,FIGNL1,LOC100101148,LOC541473,TMEM248,ZNF92,LOC100130849,MRPS17,MIR3914-1,SUN3,LOC100128885,FKBP9P1,KCTD7,CALN1,SNORA5A,LOC101928401,LOC650226,MIR6839,PMS2P7,LOC100996437,ZPBP,LOC100240728,ZNF138,SPDYE8P,LINC01372,VSTM2A,GUSBP10,MYO1G,IGFBP3,DDC,COBL,CDC14C,DDC-AS1,C7orf69,VOPP1,GS1-124K5.11,SUMF2,ZNF733P,ZNF679,VSTM2A-OT1,PMS2P2,ZNF107,NACAD,TBRG4,LOC641746,AUTS2,SNORA22,LOC100287704,ZNF735,ZNF727,SNORA5B,HUS1,MIR4283-2,MIR3147,NSUN5,STAG3L1,ZNF479,CCT6P3,ZNF680,LINC01445,CCM2,LOC100507468,POM121,C7orf57,GBAS,PMS2P4,CHCHD2,ZNF716,ZNF736,SEC61G,PMS2P5,ZNF273,TPST1,NSUN5P2,EGFR-AS1,EGFR,INTS4P2,SEPT14,LINC00174,LANCL2,SPDYE7P	3	gain
3574	CGTE_44	7	72722337	72738695	7q11.23	TRIM50,NSUN5	5	gain
3575	CGTE_44	7	72743384	73030591	7q11.23	BCL7B,MLXIPL,FKBP6,TBL2,BAZ1B,FZD9	3	gain
3576	CGTE_44	7	73038481	73082449	7q11.23	MLXIPL,VPS37D	0	loss
3577	CGTE_44	7	73083777	99032964	7q22.1-q21.3	ABCB4,LOC101927446,CLIP2,MIR5692A1,SEMA3C,MAGI2-AS3,PDAP1,CCL26,LOC101927378,POM121C,DBF4,SPDYE5,STEAP2,ARPC1A,VPS37D,KIAA1324L,STX1A,POR,GTF2IP1,GNAT3,MDH2,FZD1,SGCE,PDK4,HSPB1,ABHD11-AS1,SRRM3,TRRAP,MIR4651,ZP3,HGF,CROT,MYH16,TFPI2,ABCB1,YWHAG,PTPN12,BUD31,KPNA7,LOC100128317,DLX5,MGC16142,CLDN4,LOC101927269,SMURF1,LOC100133091,GNG11,MIR489,CYP51A1-AS1,NCF1C,LIMK1,BHLHA15,C7orf62,RSBN1L,TMEM130,NSUN5P1,TMEM60,ANKIB1,ABHD11,KRIT1,LOC101927497,CACNA2D1,CDK14,LMTK2,GSAP,ASB4,CCDC146,FGL2,GATSL2,AKAP9,PEX1,DYNC1I1,TMEM243,GRM3,C7orf76,FDPSP2,TP53TG1,SEMA3A,GTF2I,STEAP2-AS1,CASD1,GNAI1,GTF2IP4,MIR4652,BRI3,SDHAF3,RFC2,LOC101926943,SRI,SCARNA28,ATP5J2-PTCD1,HIP1,DMTF1,GATAD1,CALCR,ELN,MIR591,DLX6-AS1,PCLO,SAMD9,RUNDC3B,APTR,OCM2,DTX2P1-UPK3BP1-PMS2P11,PON1,ASNS,MIR5692A2,TMEM120A,DLX6,CLDN3,SEMA3E,MIR653,VPS50,COL1A2,PMS2P5,SNORA14A,SHFM1,BAIAP2L1,MAGI2-AS2,STYXL1,LOC101927550,SEMA3D,LOC102723885,NCF1,BET1,SAMD9L,STAG3L1,PHTF2,PMS2P9,MIR4284,LAT2,PEG10,CLDN12,DNAJC30,TRIM74,MIR548M,MAGI2,CYP51A1,CFAP69,SLC25A40,MGC72080,TAC1,STAG3L2,NPTX2,UPK3B,STEAP1,LRRD1,STEAP4,ZNF804B,GTF2IRD1,GTPBP10,RHBDD2,LOC101927356,SLC25A13,RPL13AP17,POMZP3,CD36,LOC101409256,CCL24,WBSCR22,HEPACAM2,MIR590,GNGT1,PPP1R9A,PON2,PTCD1,EIF4H,PMS2P3,LOC101927243,ADAM22,SSC4D,SPDYE8P,MTERF1,DTX2,PMS2P7,PON3,GTF2IRD2B,WBSCR16,FAM133DP,TRIM73,DPY19L2P4,CDK6,RBM48,TECPR1,GTF2IRD2,ARPC1B,FAM133B,LOC100506136,WBSCR27,MIR3609,WBSCR28	3	gain
3578	CGTE_44	7	100806022	100816785	7q22.1	VGF,NAT16	6	gain
3579	CGTE_44	7	115857781	116436190	7q31.2	MET,LOC102724434,CAV2,LINC01510,TES,CAV1	5	gain

3580	CGTE_44	7	116502589	119914775	7q31.31-q31.2	MIR6132,CFTR,ANKRD7,KCND2,CTTNBP2,CAPZA2,WNT2,ST7-AS1,LSM8,ST7-AS2,ST7-OT3,ST7-OT4,ST7,LVCAT5,ASZ1	4	gain
3581	CGTE_44	7	119914778	158937439	7q32.2-q31.32	CNTNAP2,WEE2,SMO,MGAM2,ACTR3C,MTRNR2L6,OPN1SW,TMEM229A,FAM131B,RNF133,LOC101928605,TNPO3,ZNF398,HTR5A,WNT16,C7orf55-LUC7L2,LOC101928861,ZBED6CL,TMEM209,NUB1,LINC01287,LINC00689,TTC26,KIAA1147,MIR595,IMPDH1,ARF5,TRPV6,LOC100506585,LOC285889,PTN,OR2F1,NRF1,TMEM176B,LOC101927914,PAX4,NDUFB2-AS1,ATP6V0E2,FAM180A,OR2A14,RNF148,PRRT4,NDUFA5,LINC00244,SLC35B4,EN2,KEL,NOM1,TBXAS1,FASTK,GALNT11,PRSS3P2,GPR37,OR2A25,SPAM1,VIPR2,IQUB,TPI1P2,ZNF746,LINC01000,FABP5P3,C7orf55,GIMAP1-GIMAP5,MKLN1-AS,LOC102724555,BPGM,NCAPG2,MEST,MIR593,COPG2,CPA1,SSBP1,AGAP3,LOC100129148,ZC3HC1,MIR548T,PIP,TPK1,LUZP6,GIMAP8,FSCN3,RNU6-2,KRBA1,CNPY1,EXOC4,ZNF777,AGK,OR2A5,TAS2R5,PODXL,LMBR1,PAXIP1,HILPDA,ZNF767P,REPIN1,ASB15,OR2F2,PRSS37,WDR60,SMARCD3,LOC101928211,LINC01003,MIR6892,OR9A2,FAM71F1,LOC101928782,PDIA4,OR6V1,GSTK1,LOC100130880,ZNF467,RBM33,DENND2A,SND1,CREB3L2,KCP,KCNH2,CHRM2,PRKAG2,ZYX,LINC00996,OR2A9P,ADCK2,PRSS58,ZNF212,WASL,PRSS1,MIR548I4,LOC407835,CEP41,SLC37A3,KMT2C,ATG9B,TRIM24,CNOT4,ASB10,MRPS33,LMOD2,OR2A2,LOC101928333,GBX1,CDK5,CUL1,MIR3907,AKR1B1,MIR129-1,BLACE,CTAGE15,CASP2,MOXD2P,CALU,MNX1,ASIC3,ARHGEF34P,PTPRN2,SLC13A1,WEE2-AS1,CHPF2,LOC154761,ZNF775,TRPV5,KLHDC10,ARHGEF5,CTAGE8,CADPS2,MNX1-AS1,AHCYL2,SSMEM1,TAS2R38,MIR153-2,ZNF783,CCDC136,C7orf13,HYALP1,KLF14,RHEB,MIR5707,GIMAP2,LOC101928254,JHDM1D-AS1,MESTIT1,MIR96,LINC01022,SMKR1,DNAJB6,WDR91,CTAGE4,MGC27345,EPHA1,CTAGE6,LOC100130705,KCND2,WDR86-AS1,MIR6509,NOBOX,FAM3C,TSGA13,C7orf34,ZNF800,ABCB8,TCAF1,CLEC2L,LOC100506860,AKR1D1,LOC728743,MIR182,MIR592,TCAF2,STRIP2,CRYGN,GHET1,C7orf33,TMEM139,CLEC5A,MIR6133,IRF5,KIAA1549,OR6B1,GIMAP6,ATP6V0A4,MIR29B1,ATP6V0E2-AS1,OR2A1,LRRC4,C7orf73,MIR490,MTPN,TAS2R39,MIR29A,TMEM140,METTL2B,ZNF425,PARP12,MGAM,SHH,NUP205,TMEM178B,MIR4468,EZH2,ABCF2,GRM8,LOC101928700,OR2A1-AS1,RNF32,AKR1B10,ZNF862,MIR548F4,HTR5A-AS1,LRRC61,MIR183,LRGUK,POT1,CHCHD3,OR9A4,AASS,C7orf49,TAS2R40,FLJ40288,SSPO,GIMAP4,DPP6,TSPAN12,LOC101928283,MIR3654,TMEM213,HIPK2,PAXIP1-AS1,OR2A12,SLC13A4,EPHA1-AS1,ZC3HAV1,UBE3C,LOC155060,LINC01006,LUC7L2,STRA8,LINC-PINT,ACTR3B,AGBL3,NOS3,BRAF,RBM28,ZNF282,KDM7A,AOC1,PAXIP1-AS2,ATP6V1F,GIMAP7,PLXNA4,UBE2H,GALNTL5,ZNF786,TAS2R4,MKLN1,CPED1,FEZF1-AS1,OR6W1P,OR2A42,GIMAP1,ARHGEF35,HYAL4,KLRG2,PRKAG2-AS1,EPHB6,MKRN1,LOC389602,LOC349160,C7orf77,TRY2P,SND1-IT1,MIR671,OR2A7,FLNC,LOC100506302,FAM71F2,TAS2R16,ZC3HAV1L,TSPAN33,NDUFB2,RARRES2,INSIG1,CPA4,CPA2,TAS2R60,POT1-AS1,TMUB1,LOC100506682,IQCA1L,CALD1,UBN2,RAB19,TAS2R41,TCAF2P1,AKR1B15,GCC1,GIMAP5,DGKI,TAS2R3,CPA5,SVOPL,FEZF1,OR2A20P,ESYT2,ING3,XRCC2,TMEM176A,WDR86,SLC4A2,MIR335,LEP,CLCN1,PTPRZ1	3	gain

3582	CGTE_44	8	54791774	145001260	8q21.11-q11.23	BREA2,CASC19,SNORD87,RMDN1,SLA,PSKH2,LINC01608,POP1,C8orf44,ODF1,TRAM1,MIR5681B,ST3GAL1,SDC2,LY6H,REXO1L2P,MIR1206,MIR3150B,STK3,LINC01592,COX6C,LINC01603,LINC00535,POLR2K,INTS8,SLC26A7,RPL7,UBR5-AS1,TERF1,SLC10A5,RAD21,YTHDF3,ERICH5,DPYS,IL7,MIR3150A,MIR5708,CLVS1,ZFP41,COPS5,CA8,LOC102724710,LINC01030,MIR8084,MIR124-2,TRMT12,LINC01609,FABP4,MIR1205,LOC101929528,SNHG6,AGO2,TMEM67,LOC101927040,ASAP1-IT1,MIR6845,ABRA,C8orf34-AS1,ENY2,FAM83A,DERL1,MRPS28,MIR599,LINC01111,ZFAT-AS1,BHLHE22,SOX17,ATP6V0D2,LINC01298,GPIHBP1,TIGD5,ASAP1,RNF19A,COLEC10,HRSP12,HEY1,NDUFAF6,ZFHX4-AS1,MIR7641-2,MIR4472-1,ZBTB10,SAMD12-AS1,C8orf37-AS1,DCAF4L2,PREX2,E2F5,LY6E,CALB1,ZHX1,SULF1,PENK,ZNF572,LYNX1,COL14A1,LOC102724623,TCF24,PSCA,PDP1,PCAT1,MIR4470,ANGPT1,TSNARE1,LINC00251,SLC45A4,MSC,YTHDF3-AS1,EIF3E,DPY19L4,MOS,LINC01602,LOC100288748,CASC8,ZHX1-C8orf76,BAALC,TATDN1,CSMD3,RBM12B-AS1,RHPN1-AS1,ZNF706,NDRG1,CPNE3,RPS20,LINC00588,BAALC-AS1,RNF139-AS1,SLC30A8,TOX,GGH,C8orf37,MAPK15,MTFR1,FABP9,PKIA-AS1,LOC102724874,MIR875,EIF3H,CASC11,CCDC166,FBXO32,ZC2HC1A,LINC01607,TMEM71,LOC401463,KCNV1,CYP7B1,RDH10-AS1,LINC00964,EFR3A,ZNF623,FAM49B,TCEA1,VPS13B,RPL30,LINC01151,CDC42P3,TG,LOC105375650,ADCY8,LOC102724804,UQCRB,NBN,MTBP,LOC101929488,KCNQ3,LRRCC1,AZIN1,TMEM55A,MIR2052,EBAG9,SLC25A32,PDE7A,ATAD2,AZIN1-AS1,EYA1,TMEM68,MIR1204,ZFAT,NOV,EPPK1,PI15,LOC101929709,HNF4G,MROH6,LOC101927822,WWP1,THEM6,MIR4663,FAM84B,CA2,SNTB1,ZFAND1,MMP16,KCNB2,SBSPON,MYC,LACTB2-AS1,SDCBP,GRHL2,FLJ46284,LOC101241902,TP53INP1,LOC100500773,HAS2-AS1,CCAT2,GLI4,TAF2,CTHRC1,RSPO2,PEX2,STMN2,LINC01301,FAM92A1,LOC101927657,CRH,ZFPM2-AS1,GML,TRIB1,NCALD,MIR124-2HG,CRISPLD1,ZHX2,RDH10,CHD7,LRP12,GEM,LOC101929415,TMEM65,NCRNA00250,CCDC26,LINC01289,MIR5681A,CASC9,FAM135B,TRIQK,SCRIB,MIR1208,GDAP1,FAM91A1,MIR6844,MIR3610,SBF1P1,PTK2,NRBP2,CPA6,MIR2052HG,UG0898H09,PAG1,TRPS1,LOC104054148,TRHR,XKR4,DNAJC5B,PYCRL,LACTB2,ADGRB1,C8orf46,LYN,RAD21-AS1,RRS1,RP1,MSC-AS1,MINCR,MIR1207,PRNCR1,C8orf34,TSTA3,HAS2,OSGIN2,RGS22,LOC105375734,UBE2W,MIR4661,TSPYL5,VCPIP1,TMEM64,LINC00967,CSPP1,XKR9,NSMCE2,CPQ,GDF6,LRRC69,LINC00968,SLURP1,MIR4471,C8orf31,HPYR1,TCEB1,C8orf44-SGK3,YWHAZ,LOC100133669,LY6K,PTTG3P,MTDH,BAALC-AS2,RUNX1T1,LINC01299,SNORD54,LOC286177,KCNK9,CA3,TPD52,ZC3H3,EXT1,FBXO43,POU5F1B,UTP23,LRRC6,LINC01606,RRS1-AS1,ATP6V1C1,MIR5680,SDR16C6P,RGS20,LYPLA1,CYP7A1,ZNF704,MIR30D,C8orf59,LINC00536,C8orf87,KHDRBS3,FABP12,TRAPPC9,ENPP2,FLJ42969,ZNF696,FZD6,KCNS2,SNX16,MCMDC2,MIR1273A,LINC01419,MIR2053,CYP11B2,PLAG1,GSDMD,MIR5194,DECR1,LOC392232,KLHL38,SQLE,PMP2,ZFHX4,NIPAL2,UBXN2B,LOC101926892,LOC100288181,SGK3,JRK,ANXA13,COL22A1,STAU2,SLC7A13,MIR4662A,FABP5,LINC00534,TTPA,CYP11B1,TOP1MT,RRM2B,TNFRSF11B,OC90,TMEM74,PLEC,MROH5,PVT1,RAB2A,MATN2,TMEM70,LAPTM4	3	gain
3583	CGTE_44	8	145001406	145004577	8q24.3	PLEC	47	gain
3584	CGTE_44	8	145004581	146279543	8q24.3	HGH1,KIFC2,ZNF16,GRINA,CPSF1,WDR97,ZNF251,PPP1R16A,ARHGAP39,COMMD5,SLC39A4,MROH1,MIR6850,HSF1,TONSL-AS1,ZNF252P,ADCK5,GPAA1,ZNF250,SHARPIN,C8orf82,C8orf33,TMEM249,MIR661,VPS28,PLEC,FOXH1,MIR6847,CYHR1,MIR6848,SLC52A2,EXOSC4,MIR6893,ZNF7,FBXL6,BOP1,OPLAH,CYC1,MIR6846,LRRC14,MFSD3,SCX,MIR6849,TONSL,ZNF34,MIR7112,LRRC24,ZNF517,MAF1,RECQL4,DGAT1,SPATC1,TMED10P1,SCRT1,ZNF252P-AS1,GPT,MIR939,RPL8,PARP10	3	gain
3585	CGTE_44	9	138378655	138379511	9q34.3	PPP1R26	0	loss

3586	CGTE_44	9	140120446	140123594	9q34.3	RNF224,CYSRT1	7	gain
3587	CGTE_44	9	140127612	140130900	9q34.3	SLC34A3	5	gain
3588	CGTE_44	10	13698596	13699513	10p13	FRMD4A	0	loss
3589	CGTE_44	10	27702744	27793808	10p12.1	PTCHD3,RAB18	5	gain
3590	CGTE_44	10	35896546	35929717	10p11.21	FZD8,GJD4	7	gain
3591	CGTE_44	10	64564771	69990938	10q21.3	LINC01515,SIRT1,JMJD1C,LOC101928961,MIR7151,JMJD1C-AS1,ADO,ATOH7,POU5F1P5,LRRTM3,DNAJC12,CTNNA3,ANXA2P3,HERC4,REEP3,EGR2,MYPN,MIR1296,NRBF2	1	loss
3592	CGTE_44	10	70286913	76994457	10q21.3-q22.2	DUPD1,LRRC20,CFAP70,SUPV3L1,USP54,TSPAN15,FAM149B1,KAT6B,GLUD1P3,ZSWIM8,MYOZ1,SPOCK2,VCL,DUSP13,VPS26A,HK1,C10orf35,BMS1P4,DDX50,SLC29A3,MRPS16,DNAJC9-AS1,DDX21,EIF4EBP2,MSS51,NUDT13,PALD1,NODAL,PPP3CB-AS1,P4HA1,LOC102723439,COMTD1,SLC25A16,DDIT4,MIR7152,TACR2,TYSND1,H2AFY2,AIFM2,AP3M1,SAR1A,ANAPC16,DNAJB12,NPFFR1,LOC101928994,CAMK2G,CCAR1,PCBD1,DNAJC9,PLA2G12B,SYNPO2L,ECD,SGPL1,LOC102723377,ASCC1,AGAP5,STOX1,C10orf105,NDST2,PLAU,MICU1,UNC5B-AS1,SAMD8,MIR1254-1,VDAC2,TET1,FUT11,COL13A1,MIR4676,SRGN,PPP3CB,C10orf55,SNORD98,CDH23,ADK,PPA1,ZSWIM8-AS1,OIT3,KIAA1279,SEC24C,CHCHD1,CHST3,HKDC1,NEUROG3,C10orf54,TBATA,ANXA7,ADAMTS14,UNC5B,PRF1,PSAP,MCU	1	loss
3593	CGTE_44	10	76994622	77159485	10q22.2	ZNF503,COMTD1,ZNF503-AS1	0	loss
3594	CGTE_44	10	77159489	91201103	10q22.3-q23.2	KCNMA1,MIR606,DLG5,SH2D4B,LOC101929574,SFTPD,DLG5-AS1,TMEM254,CFL1P1,LINC01520,FAS-AS1,SFTPA1,CH25H,LIPN,RPS24,GRID1-AS1,SLC16A12,MAT1A,MMRN2,LINC00864,ZNF503-AS2,MBL1P,LIPF,MIR4678,LINC00858,NRG3,LOC439994,PTEN,NUTM2B,ZMIZ1,BEND3P3,ZNF503,OPN4,IFIT2,MINPP1,CCSER2,LIPJ,ANXA11,PLAC9,GRID1,ZMIZ1-AS1,STAMBPL1,ACTA2,LRIT2,LIPA,FAM35A,LDB3,KCNMA1-AS3,RGR,LOC101929646,AGAP11,WAPL,IFIT3,RNLS,LIPM,LINC00856,C10orf99,FAM213A,ACTA2-AS1,LRIT1,SNCG,IFIT1,C10orf11,TSPAN14,NUTM2B-AS1,SFTPA2,KCNMA1-AS1,NUTM2D,PAPSS2,LINC01519,TMEM254-AS1,KLLN,LINC00595,LOC102723703,MIR4679-2,EIF5AL1,IFIT1B,BMS1P21,FAM25A,GLUD1,ADIRF,POLR3A,LOC642361,PPIF,BMPR1A,LOC105378367,IFIT5,ZCCHC24,LINC00857,NUTM2A-AS1,MIR4679-1,MIR346,LOC101929662,DYDC1,LOC101929234,NRG3-AS1,ATAD1,KCNMA1-AS2,GHITM,CDHR1,DYDC2,NUTM2A,ANKRD22,LIPK,FAS	1	loss
3595	CGTE_44	10	120513904	120790135	10q26.11	CACUL1,NANOS1	0	loss
3596	CGTE_44	11	562088	568192	11p15.5	MIR210,RASSF7,MIR210HG	3	gain
3597	CGTE_44	11	823474	825018	11p15.5	PNPLA2	4	gain
3598	CGTE_44	11	1029174	1030826	11p15.5	MUC6	4	gain
3599	CGTE_44	11	13030596	13032245	11p15.2	RASSF10	9	gain
3600	CGTE_44	11	64126712	64137887	11q13.1	MIR1237,RPS6KA4	3	gain
3601	CGTE_44	12	54332790	54349298	12q13.13	HOXC12,HOXC13-AS,HOXC13	5	gain

3602	CGTE_44	13	24557988	76457367	13q12.2-q21.32	SIAH3,SMIM2-IT1,LINC01053,SNORA27,NUDT15,ATP5EP2,KPNA3,NAA16,NEK5,DLEU1,PDX1,LINC00347,PAN3-AS1,OR7E37P,CAB39L,LINC00463,MIR5006,MIR3168,SPATA13,ESD,RPL21P28,SERTM1,LINC00395,MIR1297,CTAGE10P,MTMR6,ATP12A,MIR4703,ZC3H13,CPB2,WASF3,TSC22D1,DIAPH3-AS1,MTRF1,CENPJ,LINC00282,OLFM4,TPTE2P1,LINC00572,ATXN8OS,INTS6-AS1,LINC00563,CDADC1,MEDAG,SMIM2-AS1,LACC1,SUGT1,RNF6,CSNK1A1L,LINC00426,NEK3,MZT1,FLT1,THSD1,EXOSC8,GSX1,RXFP2,SUPT20H,LINC00398,LINC00548,CDX2,CCDC169,STARD13-AS,TEX26-AS1,ATP7B,PHF11,DLEU7-AS1,MIR548F5,DNAJC15,COG6,N4BP2L2,SUCLA2,KBTBD6,MIR4704,HNRNPA1L2,LOC102723968,UBL3,DHRS12,HMGB1,NUP58,SPERT,LINC01198,SLC46A3,PRR20C,TRIM13,LINC00423,BORA,LNX2,LINC00371,GTF2F2,ENOX1-AS2,LINC00558,PRR20A,CDK8,LINC00284,EPSTI1,PCDH9-AS4,LOC101929259,FAM124A,LRCH1,POSTN,DLEU2,EBPL,TUSC8,LINC01052,ST13P4,ZAR1L,ITM2B,LINC01050,LINC00571,MIR621,COG3,LINC00392,LOC103191607,SPG20,SLC25A30,WBP4,MRPS31P5,LINC00366,MIR3613,TPTE2P5,C1QTNF9,LINC00598,VPS36,FRY,DLEU7,ALG11,FAM216B,LINC00364,DACH1,LINC00348,LINC00358,TRPC4,NUFIP1,TEX26,LOC101926897,GPALPP1,DLEU1-AS1,LINC00445,MRPS31,RCBTB1,CKAP2,LINC00390,PRR20B,PIBF1,LINC00457,SNORD102,LINC00562,ALG5,UCHL3,DGKH,RCBTB2,MIR759,SERPINE3,CCDC122,LCP1,LINC00383,MIR320D1,MED4-AS1,LINC00545,KCNRG,B3GLCT,USP12-AS2,LOC101929140,SPATA13-AS1,LINC00458,LINC00412,RNASEH2B,LINC00400,LHFP,CCDC169-SOHLH2,LPAR6,PCDH9-AS2,PRR20E,PCDH8,PAN3,GUCY1B2,CTAGE11P,POMP,TPT1,UTP14C,LINC01078,MIR8079,NBEA,PDX1-AS1,TPTE2P6,LINC01055,KATNAL1,LINC00547,FOXO1,TBC1D4,MTIF3,MINOS1P1,LINC01065,PCDH20,VWA8-AS1,DIAPH3,RNF17,PRR20D,MIR15A,POLR1D,ARL11,LINC00544,MIR5007,FLT3,PCDH9,DIS3,PDS5B,SMIM2,RFC3,LMO7-AS1,MLNR,CPB2-AS1,LMO7DN-IT1,RGCC,KLHL1,SLC25A30-AS1,LINC00374,TSC22D1-AS1,RNASEH2B-AS1,CCDC70,ALOX5AP,LINC00434,SLC25A15,MTUS2,SNORA31,SLC7A1,LINC00437,LINC01058,SHISA2,KIAA0226L,LINC00378,PCDH9-AS3,MIR3169,PROSER1,EEF1DP3,STARD13,ERICH6B,KLF5,ATP8A2,USP12-AS1,SPG20-AS1,FRY-AS1,N4BP2L2-IT2,GPR12,LINC00462,LINC00550,TDRD3,TPT1-AS1,FREM2,ELF1,USPL1,PABPC3,LINC00297,AKAP11,LINC00332,LINC00566,MAB21L1,RFXAP,MTUS2-AS1,RPL21,LINC00441,INTS6,LOC101929657,DCLK1,MIR548X2,MIR4305,PCDH17,VWA8,LINC00428,MIR5693,LINC00365,RB1,SERP2,DIAPH3-AS2,SPRYD7,N4BP2L1,KL,LINC00448,GTF3A,LRRC63,LECT1,BRCA2,SMAD9,URAD,ENOX1,UFM1,KCTD4,USP12,CCNA1,OR7E156P,SOHLH2,STOML3,AMER2,WDFY2,MIR16-1,NHLRC3,MIR2276,HSPH1,HTR2A-AS1,LINC00330,LMO7DN,HTR2A,LINC00381,SUGT1P3,CYSLTR2,TPTE2P3,LINC00376,FNDC3A,MED4,SETDB2,PARP4,TNFSF11,COMMD6,LINC01048,KLF12,LINC01075,RASL11A,LM	3	gain

3603	CGTE_44	13	77522565	95279494	13q31.1-q31.3	GPR180,NDFIP2,LINC00446,LINC00363,LINC01038,LOC105370306,MIR19B1,GPC5-AS1,LINC00382,LINC00564,RBM26,SLITRK1,MIR18A,CLN5,LINC01040,MIR20A,LOC100129307,LINC01047,LINC01069,MIR17,MIR4500,SCEL-AS1,LINC00353,SPRY2,MIR92A1,LINC00559,SNORA107,MIR17HG,LINC01049,TGDS,NDFIP2-AS1,SLITRK6,LINC00433,LINC00397,GPC5,LINC00379,EDNRB,POU4F1,GPC6-AS1,IRG1,LINC00331,SLAIN1,SCEL,LINC01080,LINC00440,GPC6,MYCBP2-AS1,MIR3665,LINC01068,MIR19A,EDNRB-AS1,LINC00430,LINC00333,GPC6-AS2,LINC00375,MIR622,MYCBP2,SLITRK5,LINC00351,LINC00380,GPC5-AS2,FBXL3,MIR4500HG,RNF219-AS1,LINC00377,RBM26-AS1,DCT,LINC00410,RNF219	3	gain
3604	CGTE_44	13	95364255	102105464	13q32.1-q32.3	PCCA,ABCC4,IPO5,DNAJC3,OXGR1,RAP2A,LOC105370333,FARP1,RNF113B,TMTC4,LINC00557,STK24,UBAC2,LOC101927284,LINC00554,NALCN,MIR4306,DZIP1,ZIC5,LINC00449,LOC101927437,LINC00456,UBAC2-AS1,HS6ST3,UGGT2,LINC00411,LINC00359,MIR548AN,PCCA-AS1,DOCK9,MIR623,ITGBL1,GGACT,LINC01232,DOCK9-AS1,GPR18,FKSG29,SOX21-AS1,CLYBL-AS1,LINC01039,CLDN10-AS1,SOX21,CLYBL-AS2,MIR3170,MBNL2,TM9SF2,DOCK9-AS2,NALCN-AS1,MIR4501,DNAJC3-AS1,CLYBL,ZIC2,CLDN10,GPR183,SLC15A1	3	gain
3605	CGTE_44	13	110434503	110438412	13q34	IRS2	0	loss
3606	CGTE_44	14	79933574	81994126	14q31.1	LOC100506700,SEL1L,CEP128,STON2,TSHR,DIO2-AS1,DIO2,SNORA79,NRXN3,GTF2A1	1	loss
3607	CGTE_44	14	103390059	103396111	14q32.32	AMN	1	loss
3608	CGTE_44	14	103987257	103988824	14q32.32	CKB	7	gain
3609	CGTE_44	14	105350013	105354322	14q32.33	CEP170B	5	gain
3610	CGTE_44	14	105609769	105614263	14q32.33	JAG2	3	gain
3611	CGTE_44	15	68119103	68121600	15q23	SKOR1	98	gain
3612	CGTE_44	16	704987	707949	16p13.3	WDR90	5	gain
3613	CGTE_44	16	1660699	1664793	16p13.3	CRAMP1,IFT140	11	gain
3614	CGTE_44	16	1828394	1842341	16p13.3	IGFALS,NUBP2,SPSB3	4	gain
3615	CGTE_44	16	23724343	23766536	16p12.2	ERN2,CHP2	8	gain
3616	CGTE_44	16	31439533	31447600	16p11.2	COX6A2,ZNF843	8	gain
3617	CGTE_44	17	32908006	32953651	17q12	TMEM132E	25	gain
3618	CGTE_44	17	79095094	79096622	17q25.3	AATK	0	loss
3619	CGTE_44	17	79412344	79427800	17q25.3	BAHCC1,MIR3186	3	gain

3620	CGTE_44	18	163296	18540259	18p11.23-p11.31	MIR6788,MIR3976HG,ZNF519,ADCYAP1,THOC1,YES1,EMILIN2,MC2R,MIR6718,TGIF1,NDC80,GACAT2,CETN1,PTPRM,ANKRD30B,LOC644669,PRELID3A,LOC100996324,L3MBTL4-AS1,NDUFV2-AS1,CHMP1B,PPP4R1,CBX3P2,ANKRD62,MIR4526,LDLRAD4,NAPG,DLGAP1-AS5,LDLRAD4-AS1,TWSG1,LOC100192426,VAPA,LOC104968399,LINC00667,MYOM1,MYL12A,NDUFV2,METTL4,RNMT,LINC01444,APCDD1,LINC00526,SMCHD1,CIDEA,MIR3976,SLC35G4,C18orf42,LOC727896,CYP4F35P,TYMSOS,RALBP1,ANKRD12,LAMA1,MIR3156-2,L3MBTL4,TMEM200C,LINC01443,DLGAP1-AS3,LOC101927410,PTPN2,ANKRD20A5P,CEP192,LINC01254,LINC00668,GNAL,DLGAP1-AS2,CXADRP3,ENOSF1,ZBTB14,AFG3L2,FAM210A,MTCL1,LINC01387,PPP4R1-AS1,LRRC30,TYMS,ROCK1,DLGAP1-AS1,TXNDC2,LPIN2,CLUL1,DLGAP1-AS4,GAPLINC,USP14,EPB41L3,MIR4317,POTEC,RAB31,C18orf61,LINC00470,IMPA2,MIR5190,CEP76,LINC01255,RAB12,PSMG2,MYL12B,ARHGAP28,DLGAP1,SPIRE1,PIEZO2,LOC101927188,TUBB6,MIR7153,COLEC12,MC5R,SEH1L,MPPE1	1	loss
3621	CGTE_44	18	19751067	19757114	18q11.2	GATA6	0	loss
3622	CGTE_44	18	24127679	24128654	18q11.2	KCTD1	4	gain
3623	CGTE_44	18	25568397	78005277	18q21.33-q23	MIR924HG,KIAA1328,SERPINB2,PMAIP1,LOC339298,TCEB3B,ME2,LMAN1,LOC101927481,ASXL3,CELF4,SERPINB7,ALPK2,LINC01539,BOD1L2,RAX,RPL17-C18orf32,CFAP53,LINC01538,CCDC178,NOL4,CDH20,SEC11C,MBP,MALT1,SLC14A2-AS1,SNORD58C,GRP,HAUS1,LOC105667213,MIR4318,MRO,SLC25A52,MIR4319,ZNF532,CCDC102B,LINC01541,CDH19,TPGS2,ADNP2,SERPINB8,LINC00908,MIR3929,KCNG2,LOC101927606,LINC00909,STARD6,MAPRE2,SCARNA17,WDR7,SNHG22,SNORA37,ZCCHC2,FHOD3,MBD1,LOC400661,CNDP1,CDH7,ZNF397,MIR302F,CCBE1,LIPG,RPL17,FBXO15,SOCS6,MIR4527,DSG3,ZADH2,LINC01416,ATP5A1,KATNAL2,ZNF236,SETBP1,CNDP2,WBP11P1,EPG5,ZNF271P,RNF138,ELP2,TXNL4A,ST8SIA5,ONECUT2,LOC100505817,SIGLEC15,MIR4744,OACYLP,ZNF516,TXNL1,DSG1,ACAA2,TMX3,SLC14A2,RAB27B,DSC3,LOC102724913,PSTPIP2,LOC284294,MIR4320,ZSCAN30,LOC101060542,C18orf65,PIK3C3,SERPINB5,PIGN,SMAD7,SYT4,MIR3591,VPS4B,CBLN2,DSG1-AS1,GALNT1,SMIM21,TSHZ1,LOC400655,DSCAS,SERPINB4,DSG4,TCF4-AS1,MC4R,RBFA,MEX3C,PQLC1,LOC100505549,DYM,SKOR2,SLC39A6,MEP1B,ELAC1,LINC01029,MIR5011,LOC101928167,RNF165,ST8SIA3,DSG2,LINC00305,LINC01477,SMAD2,LINC00683,MOCOS,TCF4,PARD6G,LINC01478,MIR122,CXXC1,BCL2,GTSCR1,MIR4743,C18orf32,C18orf25,FECH,GALR1,KC6,CDH2,ZNF396,MIR5583-1,MIR4529,TNFRSF11A,DSC1,DSEL,MIR3975,ZNF407,ZNF24,PIAS2,LINC01544,MAPK4,NARS,SMAD4,MIR187,POLI,B4GALT6,SLC14A1,DTNA,LOC101927651,DYNAP,C18orf21,DCC,LOC101927229,ATP8B1,RIT2,SERPINB12,HDHD2,TIMM21,RNF125,SERPINB10,LOC100287225,CYB5A,HSBP1L1,HMSD,KDSR,RTTN,DSC2,CD226,CTIF,SERPINB3,RBFADN,LINC-ROR,TCEB3CL2,GAREM1,ZBTB7C,SKA1,MIR5583-2,LOC643542,SNORD58B,SALL3,C18orf63,LOXHD1,NFATC1,RPRD1A,TCEB3CL,SNORA108,C18orf54,KLHL14,LOC105372071,LINC00907,MIR924,LOC100131655,TTR,MIR4528,SERPINB13,DOK6,IER3IP1,MYO5B,PHLPP1,CCDC68,RNF152,ATP9B,NEDD4L,SERPINB11,MBD2,FAM69C,NETO1,TCEB3C,CPLX4,SNORA111,DSG2-AS1,PARD6G-AS1,SNORD58A,LOC101927322,CTDP1,MIR1539,LOC102724651,TRAPPC8,INO80C,KIAA1468	1	loss

3624	CGTE_44	19	1108294	1109781	19p13.3	SBNO2	276	gain
3625	CGTE_44	19	1233395	1257156	19p13.3	ATP5D,CBARP,MIDN	3	gain
3626	CGTE_44	19	10528440	10543355	19p13.2	PDE4A	6	gain
3627	CGTE_44	19	18901558	18902255	19p13.11	COMP	5	gain
3628	CGTE_44	19	48182423	48197493	19q13.33	GLTSCR1	0	loss
3629	CGTE_44	19	48673389	48686003	19q13.33	LIG1,C19orf68	6	gain
3630	CGTE_44	19	58879130	58880780	19q13.43	ZNF837	18	gain
3631	CGTE_44	20	60897209	60898961	20q13.33	LAMA5	10	gain
3632	CGTE_44	20	60908052	60909856	20q13.33	LAMA5	5	gain
3633	CGTE_44	20	62152879	62223558	20q13.33	HELZ2,C20orf195,PPDPF,GMEB2,PTK6,SRMS	3	gain
3634	CGTE_44	22	17589804	17590775	22q11.1	IL17RA	5	gain
3635	CGTE_44	22	19511121	19702380	22q11.21	CLDN5,SEPT5,LINC00895	4	gain
3636	CGTE_44	22	20130396	20136478	22q11.21	ZDHHC8,CCDC188	0	loss
3637	CGTE_44	22	20136679	20137238	22q11.21	CCDC188	4	gain
3638	CGTE_44	22	21983160	21984303	22q11.21	YDJC	12	gain
3639	CGTE_44	22	50699579	50705789	22q13.33	MAPK12,MAPK11	3	gain
3640	CGTE_44	22	50705791	50708746	22q13.33	MAPK11	7	gain
3641	CGTE_44	22	50898019	50898760	22q13.33	SBF1	13	gain
3642	CGTE_44	Y	2655076	14475518	Yq11.1-Yq11.21	TTTY23B,TGIF2LY,TTTY18,TTTY12,TSPY8,TTTY23,RBMY3AP,TSPY10,TTTY21B,TTTY11,PCDH11Y,TTTY7B,LINC00280,TTTY16,LINC00278,TTTY22,TTTY20,TTTY1,TSPY2,TBL1Y,AMELY,TTTY21,TTTY19,FAM197Y5P,RBMY1A3P,ZFY,TSPY3,PRKY,TTTY2,TSPY4,RPS4Y1,TTTY1B,TTTY8,TTTY2B,TSPY1,SRY,FAM197Y2P,TTTY7,TTTY8B	0	loss
3643	CGTE_44	Y	14503837	16953276	Yq11.221-Yq11.21	GYG2P1,NLGN4Y,VCY1B,VCY,UTY,USP9Y,DDX3Y,NLGN4Y-AS1,TTTY15,TMSB4Y	0	loss
3644	CGTE_44	Y	21628782	24460980	Yq11.222-Yq11.223	TXLNGY,RBMY1E,RPS4Y2,RBMY1F,LOC101929148,RBMY1A1,BCORP1,RBMY1B,KDM5D,RBMY1J,RBMY1D,PRY2,PRORY,TTTY6B,TTTY5,TTTY13,PRY,TTTY10,TTTY6,RBMY2EP,RBMY2FP,EIF1AY	0	loss
3645	CGTE_45	1	1178371	1179432	1p36.33	FAM132A	0	loss
3646	CGTE_45	1	1560821	1564479	1p36.33	MIB2	0	loss
3647	CGTE_45	1	110452951	110716732	1p13.3	CSF1,SLC6A17,STRIP1,AHCYL1,LINC01397,UBL4B,ALX3	3	gain
3648	CGTE_45	2	202402087	202412425	2q33.1	ALS2CR11	1	loss
3649	CGTE_45	3	73096187	73110159	3p13	PPP4R2	1	loss
3650	CGTE_45	3	138724504	138738804	3q23	PRR23A,PRR23B	1	loss
3651	CGTE_45	3	148889875	148894245	3q24	CP,HPS3	4	gain
3652	CGTE_45	3	150332256	150341046	3q25.1	SELT	3	gain
3653	CGTE_45	3	182897751	183029573	3q27.1	B3GNT5,MCF2L2	3	gain
3654	CGTE_45	3	192980699	193386346	3q29	OPA1-AS1,HRASLS,OPA1,ATP13A4-AS1,ATP13A5-AS1,ATP13A4,ATP13A5	3	gain
3655	CGTE_45	4	2010470	2043840	4p16.3	C4orf48,NELFA	1	loss
3656	CGTE_45	4	8021325	8031534	4p16.1	ABLIM2	3	gain
3657	CGTE_45	5	145144367	145214819	5q32	PRELID2	3	gain
3658	CGTE_45	5	176647987	176662944	5q35.3	NSD1	4	gain
3659	CGTE_45	6	43112915	43535163	6p21.1	CRIP3,PTK7,POLR1C,SLC22A7,TJAP1,ZNF318,YIPF3,TTBK1,MIR6780B,XPO5,CUL9,LRRC73,DNPH1,DLK2,SRF,ABCC10	3	gain
3660	CGTE_45	7	81588522	83246009	7q21.11	SEMA3E,PCLO,LOC101927356,CACNA2D1	3	gain
3661	CGTE_45	7	84628699	84751238	7q21.11	SEMA3D	3	gain
3662	CGTE_45	7	86273982	86577291	7q21.11-q21.12	GRM3,KIAA1324L	5	gain
3663	CGTE_45	7	86594166	87229552	7q21.12	CROT,TMEM243,ABCB4,KIAA1324L,TP53TG1,ABCB1,DMTF1	8	gain
3664	CGTE_45	7	87257966	87913528	7q21.12	RUNDC3B,ADAM22,SLC25A40,SRI,ABCB1,LOC102723885,DBF4,STEAP4	9	gain
3665	CGTE_45	9	5335198	5339445	9p24.1	RLN1	4	gain
3666	CGTE_45	9	139271962	139273930	9q34.3	SNAPC4	1	loss

3667	CGTE_45	9	139274248	139276315	9q34.3	SNAPC4	3	gain
3668	CGTE_45	9	139276405	139277985	9q34.3	SNAPC4	1	loss
3669	CGTE_45	9	139398936	139401157	9q34.3	NOTCH1	1	loss
3670	CGTE_45	9	139747560	139753293	9q34.3	MAMDC4	1	loss
3671	CGTE_45	9	139915103	139916049	9q34.3	ABCA2	0	loss
3672	CGTE_45	9	140127612	140130900	9q34.3	SLC34A3	0	loss
3673	CGTE_45	10	78317094	78977353	10q22.3	KCNMA1,C10orf11,KCNMA1-AS2,KCNMA1-AS1	3	gain
3674	CGTE_45	10	79553553	80310736	10q22.3	POLR3A,DLG5-AS1,LINC00595,RPS24,DLG5,LINC00856	3	gain
3675	CGTE_45	10	80761920	82045401	10q22.3-q23.1	PLAC9,PPIF,EIF5AL1,ZMIZ1-AS1,SFTPA2,SFTPA1,MAT1A,SFTPD,MBL1P,ANXA11,TMEM254,BEND3P3,TMEM254-AS1,LINC00857,BMS1P21,LOC642361,ZCCHC24,NUTM2B,NUTM2B-AS1,ZMIZ1	4	gain
3676	CGTE_45	10	82048994	89263804	10q23.1-q23.2	LOC101929646,LRIT2,MMRN2,AGAP11,DYDC2,GRID1-AS1,LINC00864,TSPAN14,CCSER2,FAM25A,SNCG,GLUD1,NRG3,RGR,LDB3,MAT1A,LOC439994,WAPL,NUTM2A,MIR4678,DYDC1,LOC101929574,NUTM2A-AS1,FAM35A,ADIRF,BMPR1A,LINC01519,SH2D4B,MIR346,LINC01520,LOC102723703,C10orf99,GHITM,LINC00858,OPN4,NUTM2D,FAM213A,LOC101929662,GRID1,LRIT1,NRG3-AS1,CDHR1	3	gain
3677	CGTE_45	10	90034545	90683079	10q23.31	LIPM,STAMBPL1,LIPJ,ANKRD22,LIPF,LIPN,RNLS,LIPK	3	gain
3678	CGTE_45	10	123233863	124344888	10q26.13	TACC2,MIR3941,FGFR2,HTRA1,ATE1-AS1,PLEKHA1,DMBT1,ATE1,BTBD16,ARMS2,NSMCE4A	3	gain
3679	CGTE_45	10	124345575	124402860	10q26.13	DMBT1	4	gain
3680	CGTE_45	11	64126712	64138222	11q13.1	MIR1237,RPS6KA4	1	loss
3681	CGTE_45	11	67205051	67206480	11q13.2	CORO1B,PTPRCAP	1	loss
3682	CGTE_45	12	42838838	42840172	12q12	PPHLN1	4	gain
3683	CGTE_45	13	21729783	21736073	13q12.11	SKA3	3	gain
3684	CGTE_45	14	103388895	103396383	14q32.32	AMN	1	loss
3685	CGTE_45	15	25442805	25616963	15q11.2	SNORD115-30,SNORD115-27,SNORD115-24,SNORD115-38,UBE3A,SNORD115-25,SNORD115-33,SNORD115-20,SNORD115-32,SNORD115-44,SNORD115-31,SNORD109B,SNORD115-11,SNORD115-26,SNORD115-36,SNORD115-17,SNORD115-39,SNORD115-46,SNORD115-43,SNORD115-29,SNORD115-22,SNORD115-19,SNORD115-35,SNORD115-28,SNORD115-21,SNORD115-47,SNORD115-15,SNORD115-37,SNORD115-34,SNORD115-18,PWAR4,SNORD115-41,SNORD115-42,SNORD109A,SNORD115-23,SNORD115-48,SNORD115-10,SNORD115-45,SNORD115-40,SNORD115-16	3	gain
3686	CGTE_45	15	90135257	90818965	15q26.1	NGRN,CIB1,MIR3174,SEMA4B,KIF7,PEX11A,TICRR,ANPEP,MESP2,AP3S2,C15orf38-AP3S2,GDPGP1,MESP1,MIR5094,WDR93,IDH2,TTLL13P,PLIN1,ZNF710,ARPIN	3	gain
3687	CGTE_45	15	90892065	90905896	15q26.1	ZNF774,GABARAPL3	4	gain
3688	CGTE_45	15	90931466	91163127	15q26.1	CRTC3,IQGAP1	3	gain
3689	CGTE_45	16	1832841	1841830	16p13.3	NUBP2,IGFALS	1	loss
3690	CGTE_45	16	67211927	67212627	16q22.1	KIAA0895L	0	loss
3691	CGTE_45	17	41454972	42018603	17q21.31	ARL4D,MEOX1,LINC00910,ETV4,SOST,DHX8,DUSP3,FAM215A,C17orf105,CD300LG,PPY,MIR2117,MPP3,MPP2	3	gain
3692	CGTE_45	18	9124945	9134187	18p11.22	NDUFV2-AS1,NDUFV2	0	loss
3693	CGTE_45	18	14106097	14124564	18p11.21	ZNF519	4	gain
3694	CGTE_45	19	29698974	30308466	19q12	CCNE1,VSTM2B,C19orf12,POP4,UQCRFS1,PLEKHF1,LOC284395	4	gain
3695	CGTE_45	19	30311552	30506560	19q12	URI1,CCNE1	5	gain
3696	CGTE_45	19	30934490	31619490	19q12	ZNF536	3	gain
3697	CGTE_45	21	27124655	27130550	21q21.3	GABPA	4	gain

3698	CGTE_46	1	861266	3732963	1p36.32-p36.33	CPSF3L,VWA1,CALML6,PRKCZ,UBE2J2,PERM1,LOC115110,LINC00982,NADK,CFAP74,TTLL10,SMIM1,TTC34,FAAP20,PRDM16,MMP23B,TNFRSF14,CDK11A,NOC2L,ACTRT2,TMEM52,SKI,TNFRSF18,TP73,ACAP3,PLEKHN1,MIR200B,MMP23A,WRAP73,GABRD,ATAD3B,LOC100129534,TAS1R3,MRPL20,TMEM240,LOC148413,LINC01342,KLHL17,AURKAIP1,MIR429,GNB1,SSU72,MIR6808,MORN1,PLCH2,AGRN,FAM132A,CCNL2,TNFRSF4,PANK4,MIR6727,SLC35E2,MIR4251,MXRA8,MIB2,CDK11B,TPRG1L,LOC100996583,MIR551A,CPTP,C1orf233,MEGF6,ANKRD65,MIR6726,MMEL1,RNF223,CEP104,LRRC47,ARHGEF16,LOC102724312,HES5,SLC35E2B,TMEM88B,PEX10,SDF4,PUSL1,TP73-AS1,SCNN1D,B3GALT6,DVL1,FAM213B,ISG15,HES4,SAMD11,RER1,ATAD3A,CCDC27,MIR200A,C1orf159,ATAD3C	0	loss
3699	CGTE_46	1	206240053	225742803	1q32.1-q42.11	MIR4742,SERTAD4,CNIH4,LYPLAL1,PFKFB2,LINC00538,NSL1,MIR215,MAPKAPK2,SMYD2,LOC101929541,IKBKE,KCTD3,LINC00467,CD55,LOC102723833,CR1,CR1L,RCOR3,TGFB2,TRAF5,WDR26,MIR548F3,ANGEL2,C1orf116,CTSE,SRGAP2,MIR320B2,ESRRG,GTF2IP20,MIR6769B,HHIPL2,PROX1-AS1,TAF1A,MIR205HG,PROX1,INTS7,CENPF,CAPN2,ENAH,BROX,TGFB2-OT1,G0S2,USH2A,DIEXF,NVL,IL20,DYRK3,LOC101927143,EPRS,TRAF3IP3,SNORA16B,IL19,DISP1,KCNK2,IARS2,KCNH1,SRGAP2C,LBR,LOC105748977,MIR3122,VASH2,TAF1A-AS1,MIR194-1,YOD1,RRP15,DTL,SPATA17,MIR29B2,MARC2,FLVCR1,MIR205,LINC01352,PIGR,NEK2,HHAT,MARC1,GPATCH2,BATF3,ATF3,HSD11B1,RPS6KC1,FLVCR1-AS1,LOC148696,RNU5F-1,CAMK1G,EIF2D,C4BPA,C1orf140,HLX,CCDC185,SYT14,TMEM206,FCMR,SRGAP2D,CAPN8,C1orf115,MIR4260,SLC30A10,MARK1,TP53BP2,AIDA,FAM71A,SUSD4,LPGAT1,TLR5,C1orf186,RD3,PPP2R5A,TGFB2-AS1,RAB3GAP2,DEGS1,IL10,SPATA45,LOC101929771,C1orf74,CNIH3,AURKAPS1,DNAH14,FAM177B,MIA3,HLX-AS1,SERTAD4-AS1,LOC101929631,LOC101927164,SLC30A1,PLXNA2,RASSF5,SNORA36B,SPATA17-AS1,LINC00210,NENF,BPNT1,IRF6,FCAMR,PTPN14,LAMB3,TATDN3,C4BPB,LOC101929565,CD46,MIR29C,MIR664A,FBXO28,CR2,IL24,CD34,LYPLAL1-AS1,DUSP10	3	gain
3700	CGTE_46	1	228108968	229568892	1q42.13	ARF1,DUSP5P1,RAB4A,MIR4666A,TRIM17,CCSAP,WNT9A,MIR7641-2,MIR4454,IBA57,BTNL10,WNT3A,TRIM11,HIST3H2BB,C1orf145,ACTA1,GUK1,MIR6742,RHOU,RNF187,MIR5008,IBA57-AS1,MIR3620,SPHAR,C1orf35,MRPL55,HIST3H3,HIST3H2A,GJC2,OBSCN	0	loss
3701	CGTE_46	2	74783075	85097496	2p11.2-p12	FUNDC2P2,REG3A,LOC101927926,REG3G,GCFC2,LRRTM1,SEMA4F,SNAR-H,TACR1,EVA1A,LOC101927967,DNAH6,TRABD2A,REG1A,LINC01291,LOC101927907,LRRTM4,LOC101927948,MRPL19,SUCLG1,REG1B,MIR5000,M1AP,POLE4,CTNNA2,MIR4264,DOK1,LOC101927987,REG1CP,LOC1720,MIR8080,LOC101927884,LOC100507201,HK2	3	gain
3702	CGTE_46	2	122516226	127659559	2q14.3	GYPC,TSN,CNTNAP5	3	gain

3703	CGTE_46	2	131100624	170336166	2q24.2-q24.1	LOC100507600,WTH3DI,LYPD6,TBR1,MARCH7,RPRM,LOC102724058,ACVR1C,LOC101928273,MIR4785,SLC38A11,CCDC148-AS1,TMEM163,RND3,NCKAP5,LOC101929231,LOC101929570,ANKRD30BL,SCN1A,FIGN,C2orf27A,MAP3K19,LOC101929260,PRPF40A,POTEI,CERS6-AS1,CFC1B,TANC1,GALNT5,FMNL2,GPR148,PABPC1P2,CCNT2-AS1,STAM2,HNMT,ZEB2,DARS,SPC25,MBD5,MIR7853,MIR663B,LOC101929319,UBXN4,ACVR2A,CERS6,MZT2A,LOC101929680,KIF5C,ERMN,LINC01412,MIR3679,MIR5590,SLC4A10,FAR2P2,C2orf27B,THSD7B,LOC643072,MIR4773-2,CYP4F62P,MIR7157,LCT,BAZ2B,LY75,POTEE,ARL5A,LOC100144595,DPP4,POTEKP,CYTIP,LOC101929532,RIF1,LRP1B,KCNJ3,UPP2,PLEKHB2,MIR4773-1,TTC21B,LY75-CD302,GCG,KYNU,TISP43,PKP4,LOC101928386,CCDC74A,RBMS1,DHRS9,ABCB11,PSMD14,FAP,ARHGAP15,LOC646743,POTEJ,LOC100506124,XIRP2,BBS5,PTPN18,B3GALT1,TTC21B-AS1,CCNT2,WDSUB1,TEX41,MCM6,NXPH2,PLA2R1,SCN7A,LYPD1,ORC4,IFIH1,R3HDM1,NR4A2,CACNB4,TNFAIP6,SNORA70F,SCN3A,GRB14,ZNF806,STK39,MGAT5,CD302,KCNH7,NEB,IMP4,CSRNP3,DARS-AS1,ITGB6,MIR4774,LINC01120,ACMSD,ACVR1,MIR128-1,COBLL1,NOSTRIN,GTDC1,GALNT13,LOC101928161,SCN2A,SPOPL,LOC101929512,AMER3,DAPL1,G6PC2,LOC401010,CCDC148,AHCTF1P1,ZEB2-AS1,TUBA3D,GPD2,LOC150776,SCN9A,LINC01087,CFC1,MIR6888,LOC105616981,LOC101929378,LOC100996579,ARHGEF4,ARL6IP6,EPC2,CYP4F30P,GALNT3,LOC101929633,MMADHC,YY1P2,LOC100505984,ZRANB3,NMI,RAB3GAP1,GCA,MIR4784,RBM43,GPR39,CXCR4,LYPD6B,LOC101929282,FAM168B,LOC440910,TANK,LRP2,XIRP2-AS1	3	gain

3704	CGTE_46	3	77530054	125899472	3p11.1-p12.3	SEC22A,MIR5092,EAF2,EPHA6,ARHGAP31-AS1,LSAMP,ADGRG7,MIR548AB,LINC00506,MIR6083,LRRC58,CD200R1L,KIAA1524,MTHFD2P1,HTR1F,CADM2,CSTA,ILDR1,B4GALT4,BTLA,RETNLB,OR5K1,LOC101927374,MAATS1,STX19,PROS1,ST3GAL6,LOC101929694,ARHGAP31,TAGLN3,DZIP3,TMEM30C,PLCXD2-AS1,NXPE3,OR5H6,MUC13,MIR8076,DRD3,ST3GAL6-AS1,SEMA5B,MYH15,NSUN3,PLA1A,LSAMP-AS1,SENP7,FAM86JP,IGSF11,TMEM39A,MIR4447,POPDC2,EPHA3,ZNF80,OR5H14,LINC00488,MIR567,MIR544B,GAP43,MIR4795,MIR3921,OSBPL11,CD86,TMEM45A,MYLK-AS1,ABHD10,MIR5688,SNORA95,PLCXD2,TRMT10C,ARL13B,MYLK,ZBTB11-AS1,PVRL3-AS1,GOLGB1,SLC9C1,MIR3923,ZDHHC23,LOC101929579,PHLDB2,FILIP1L,BOC,LINC00971,ZBTB20,LOC102723582,ADCY5,OR5AC2,LOC101929754,NDUFB4,IQCB1,CCDC58,ARL6,KALRN,GPR15,ALG1L,SLC12A8,STXBP5L,NAA50,MIR7110,SNX4,C3orf52,CHMP2B,KPNA1,GPR156,TMPRSS7,C3orf30,CADM2-AS2,MIR8060,CD200,VGLL3,LINC00879,IFT57,COX17,PCNP,LOC728290,GRAMD1C,LINC01215,GCSAM,DIRC2,TBC1D23,GABRR3,ZNF654,ZBTB11,FAM172BP,LOC152225,ROPN1,NR1I2,PARP15,LINC01205,ALDH1L1-AS2,USF3,C3orf38,ZBTB20-AS4,OR5H2,MIR4445,NFKBIZ,CCDC54,FLJ22763,RABL3,DUBR,TOMM70A,CD96,DPPA2,LINC00882,HSPBAP1,HHLA2,LINC01279,CASR,CBLB,OR5K4,ROPN1B,NIT2,MINA,LNP1,DPPA4,B4GALT4-AS1,ITGB5,MIR5682,MIR4446,DHFRL1,BBX,CMSS1,LOC101927056,HEG1,PVRL3,CCDC80,SLC35A5,ZBTB20-AS1,ARGFX,CD200R1,CFAP44-AS1,GUCA1C,IGSF11-AS1,ZNF148,CPOX,PARP14,ATP6V1A,C3orf17,ZBED2,GTF2E1,CD80,MYLK-AS2,SIDT1,CCDC191,ALCAM,PDIA5,LINC00635,CFAP44,HCLS1,FBXO40,ZBTB20-AS3,ZPLD1,LOC100129550,IMPG2,LOC101929717,OR5K3,ABI3BP,COL8A1,HACD2,TUSC7,SPICE1,PDCL3P4,SLC41A3,MIR548A3,ALDH1L1-AS1,LINC00901,OR5H1,ROBO2,CRYBG3,ALDH1L1,MIR6730,WDR5B,FAM162A,QTRTD1,MIR5002,DCBLD2,PARP9,MIR548G,POU1F1,ROBO1,OR5H15,MIR198,POGLUT1,MIR4796,LINC00636,CGGBP1,GSK3B,HGD,RPL24,MIR568,CD47,GTPBP8,UPK1B,SLC15A2,UMPS,DTX3L,TRAT1,OR5K2,ATG3,HP09053,TIMMDC1,FSTL1,CCDC14,CEP97,CLDND1,GBE1,POLQ,TIGIT,MIR548I1,ADPRH,MORC1,TFG,LOC101929607	3	gain
3705	CGTE_46	3	125899702	126070881	3q21.3	KLF15,ALDH1L1,ALDH1L1-AS2	6	gain

3706	CGTE_46	3	157160160	182614605	3q26.32-q25.33	MIR6828,MIR3919,KCCAT211,FXR1,WDR49,OTOL1,TRIM59,PDCD10,MYNN,ZNF639,MECOM,MIR548AY,ECT2,LXN,EIF5A2,ARL14,SPTSSB,LINC01322,IQCJ-SCHIP1,SPATA16,PHC3,SHOX2,NAALADL2-AS2,MIR1263,ZBBX,LOC100507661,NAALADL2-AS3,TMEM212,KPNA4,TNIK,SLC7A14,LRRC31,TNFSF10,USP13,KCNMB2,GHSR,RNU6-2,LINC01330,CCDC39,IQCJ,FNDC3B,SERPINI1,LRRIQ4,VEPH1,SKIL,LOC101928739,IFT80,MLF1,GNB4,SAMD7,LINC01206,IL12A,GFM1,KCNMB2-AS1,GOLIM4,FLJ46066,SLITRK3,LOC100128164,ATP11B,C3orf80,ZMAT3,PRKCI,MIR7977,PEX5L-AS2,BCHE,NAALADL2-AS1,PQLC2L,SOX2-OT,LINC01327,SLC2A2,SOX2,LINC01014,LINC00501,PIK3CA,LOC100996447,GPR160,MFSD1,SERPINI2,LOC101243545,MFN1,MIR551B,PLD1,B3GALNT1,SMC4,MIR16-2,TBL1XR1,CLDN11,ACTL6A,NAALADL2,NMD3,LINC01324,TERC,RSRC1,IQCJ-SCHIP1-AS1,DNAJC19,NDUFB5,ACTRT3,LINC00578,NCEH1,PPM1L,LOC102724604,LOC100505609,KCNMB3,EGFEM1P,PEX5L,LRRC34,SEC62,IL12A-AS1,RARRES1,SI,NLGN1-AS1,NLGN1,LINC01208,MIR4789,PTX3,MRPL47,MIR569,LOC101928882,TTC14,SCARNA7,LINC01209,TMEM212-AS1,SCHIP1,LINC01192,LINC01100,RPL22L1,MIR15B	3	gain
3707	CGTE_46	3	185369753	193386346	3q27.2-q29	GMNC,FGF12-AS1,LPP,MIR548AQ,MB21D2,ADIPOQ,BCL6,CRYGS,LOC101929106,MIR1248,RTP4,MIR28,SNORA4,FLJ42393,TPRG1-AS1,P3H2,TBCCD1,LINCR-0002,FGF12,MASP1,OPA1,SNORD2,DGKG,ATP13A4,LOC344887,SNAR-I,DNAJB11,OSTN,HRASLS,MGC2889,P3H2-AS1,OPA1-AS1,TRA2B,LOC253573,RTP2,IGF2BP2-AS1,UTS2B,EIF4A2,CCDC50,SST,RPL39L,CLDN1,LOC100131635,ST6GAL1,LPP-AS1,RFC4,IGF2BP2,ETV5,FETUB,ATP13A5,RTP1,SNORA63,AHSG,ATP13A5-AS1,MIR944,TPRG1,KNG1,TPRG1-AS2,PYDC2,IL1RAP,LPP-AS2,TP63,ADIPOQ-AS1,CLDN16,TMEM207,OSTN-AS1,HRG,ATP13A4-AS1,SNORA81,LOC102724699	3	gain
3708	CGTE_46	4	517309	2074216	4p16.3	CPLX1,CTBP1-AS,ATP5I,FGFR3,PCGF3,PIGG,UVSSA,SLC26A1,NKX1-1,MIR943,IDUA,NELFA,SLBP,CTBP1,CTBP1-AS2,GAK,LOC100130872,TMED11P,WHSC1,MYL5,MAEA,SPON2,PDE6B,MFSD7,POLN,CRIPAK,RNF212,TMEM129,FGFRL1,LETM1,DGKQ,C4orf48,SCARNA22,TMEM175,FAM53A,LOC100129917,TACC3,NAT8L	1	loss
3709	CGTE_46	4	3432335	3502527	4p16.3	HGFAC,DOK7,RGS12	0	loss
3710	CGTE_46	4	13628927	15006006	4p15.33	MIR5091,LINC01182,BOD1L1,LINC01085,CPEB2-AS1,CPEB2,LINC00504	0	loss

3711	CGTE_46	4	15008874	93218842	4p11-p13	SRD5A3-AS1,UGT2B4,SULT1B1,SNCA,CXCL6,QDPR,LOC100507388,FBXL5,PTPN13,PDS5A,RHOH,TBC1D19,MED28,C4orf36,CXCL13,CXCL8,LOC101927179,LCORL,EPHA5-AS1,LINC01088,CDKL2,N4BP2,LOC401134,CXCL2,MIR218-1,TMPRSS11E,GUF1,RPL9,SEPSECS-AS1,TMPRSS11GP,FAM200B,PYURF,MIR4451,SLC4A4,LOC100505912,LINC00989,MIR548AH,GPRIN3,ZCCHC4,KIAA1211,CXCL5,SNCA-AS1,MAPK10,MIR7978,AASDH,USO1,CLOCK,DCAF4L1,NMU,POLR2B,ENAM,ANTXR2,IGFBP7,LOC100506444,NAP1L5,PDCL2,EREG,MEPE,NKX6-1,SLC34A2,SCD5,MIR548AJ2,CCDC158,CLRN2,LOC101928978,SRD5A3,PACRGL,SPARCL1,PARM1,NAA11,CCKAR,SNORD143,LOC101927237,UGT2B15,HNRNPD,C1QTNF7,STIM2,GC,LOC728040,LOC550113,CCDC149,MIR7641-2,PPBPP2,CPEB2,CWH43,ANKRD17,CSN1S2BP,SULT1E1,HOPX,UBE2K,TLR10,KCTD8,BMP2K,SDAD1,MIR1255B1,MIR4450,LINC00575,LOC101928942,FIP1L1,MIR5705,RCHY1,CSN2,DSPP,AFP,AFF1,UGT2A1,PROL1,AREG,UGT2B7,TXK,CDS1,SEC31A,LIN54,CHRNA9,PF4,LOC102723778,DCK,LOC344967,UGT2B17,PGM2,CC2D2A,ENOPH1,GABRA2,HTN1,PDGFRA,SMIM20,UGT2A2,CENPC,TMPRSS11BNL,ARHGAP24,JCHAIN,LOC101927157,LIMCH1,SLC30A9,NUDT9,CXCL1,SMR3A,UGDH-AS1,ADGRL3-AS1,C4orf19,RBM47,CCNG2,HELQ,CSN1S2AP,BST1,BMP3,TMPRSS11B,TMPRSS11D,MIR4801,SHROOM3,MIR8053,KLB,MUC7,PPBP,THAP9-AS1,ABCG2,FAM13A,IGFBP7-AS1,KIT,MIR1273H,CSN3,NFXL1,NUP54,HERC6,MIR5096,LRRC66,GABRA4,USP46,HNRNPDL,PF4V1,USP46-AS1,SLC10A4,UGT2B10,FAM175A,EXOC1,RELL1,GABRG1,SHISA3,G3BP2,SNORA26,ALB,SLC10A6,UBA6-AS1,GK2,HTN3,UGT2B11,SLIT2,NCAPG,DHX15,RPL21P44,HSD17B11,RBPJ,NOA1,LOC101928622,FAM13A-AS1,SOWAHB,KCNIP4-IT1,PPAT,CNOT6L,APBB2,SEL1L3,KLF3-AS1,FAM47E-STBD1,CD38,THAP9,ADAMTS3,IBSP,SPATA18,BTC,FAM47E,YTHDC1,LNX1,STBD1,C4orf22,PI4K2B,UCHL1-AS1,CXCL11,FAM184B,TAPT1,LOC101928851,MIR4449,SLIT2-IT1,ARL9,KLHL5,PPM1K,ADGRL3,SLAIN2,MIR574,CHIC2,MIR575,TECRL,PKD2,DMP1,MIR548AG1,MIR548AX,RASGEF1B,TBC1D1,TMEM165,MRPS18C,PRKG2,MIR5591,ADGRA3,LOC401127,RASSF6,PCAT4,WDR19,OCIAD1,UCHL1,DCUN1D4,CORIN,SCFD2,COQ2,CCSER1,COX18,LOC439933,LOC101928809,HSD17B13,PRR27,CXCL10,PPARGC1A,BEND4,PLAC8,TIGD2,PIGY,NIPAL1,MIR1269A,CCNI,ANXA3,GABRB1,TMPRSS11A,PHOX2B,LOC101929199,PCDH7,STATH,MIR4275,THAP6,LOC441025,KLHL8,LNX1-AS1,ART3,THEGL,UGT2B28,MMRN1,GSX2,DANCR,DCAF16,SYT14P1,UGDH,UGT2A3,NPFFR2,REST,PROM1,FDCSP,TMPRSS11F,FGFBP1,EPGN,WDFY3-AS2,DTHD1,ZAR1,C4orf26,RFC1,LVCAT1,RUFY3,MTHFD2L,SMIM14,PRDM8,LOC101929064,SEPSECS,FRAS1,KCNIP4,CEP135,LDB2,FAM114A1,HERC3,KLF3,SNORD144,LOC100506746,OCIAD2,TLR1,TMEM33,TMEM156,CXCL9,TLR6,SGCB,LGI2,PAICS,ANAPC4,TMEM150C,SOD3,PAQR3,GPAT3,COX7B2,LOC102723828,STAP1,CABS1,RASL11B,MOB1B,NSUN7,HERC5,PTTG2,LIAS,ARAP2,NWD2,AMBN,SEPT11,COMMD8,SPINK2,CNGA1,GRSF1,CSN1S1,TEC,LOC101929194,GRXCR1,FGF5,ERVMER34-	3	gain
3712	CGTE_46	5	140384	3600446	5p15.33	LINC01017,AHRR,PLEKHG4B,LRRC14B,LINC01377,LOC100506858,EXOC3-AS1,MIR6075,LOC101929034,PDCD6,IRX2,SLC6A19,TERT,CCDC127,TPPP,HRAT5,C5orf38,MIR4277,SLC9A3,SLC6A18,LOC100506688,NKD2,MIR4457,LINC01019,LOC728613,IRX1,NDUFS6,IRX4,LPCAT1,MRPL36,CTD-2194D22.4,SLC12A7,MIR4456,BRD9,LOC100996325,SDHAP3,SDHA,LINC01511,CEP72,CLPTM1L,EXOC3,LOC100288152,PP7080,CTD-3080P12.3,TRIP13,MIR4635,ZDHHC11,SLC6A3	1	loss

3713	CGTE_46	5	3600447	54516583	5p15.2-p14.3	SUB1,DNAJC21,MIR7641-2,LOC442132,MIR4637,GDNF,RAI14,RICTOR,MIR581,ADAMTS12,CDH9,SNORD123,MIR4459,OSMR-AS1,C7,NIPBL,LOC100506639,HSPB3,MIR4454,LOC285696,LOC101929505,LINC01033,RXFP3,SNORD141A,FASTKD3,DAB2,SEPP1,OXCT1-AS1,LOC340107,BRCAT107,SNORD141B,TTC33,CCDC152,BRCAT54,LOC102467081,MIR6131,ICE1,GHR,FLJ33360,CDC20B,LOC100130744,LIFR,C1QTNF3,SLC45A2,UBE2QL1,NDUFS4,IRX1,AMACR,WDR70,PMCHL1,LOC101929645,NIPBL-AS1,GZMK,ADAMTS16,CDH12,CDH18,C9,SEMA5A,LINC01194,OTULIN,ITGA1,FST,LSP1P3,FLJ32255,NPR3,C5orf42,SNHG18,EGFLAM,OSMR,EGFLAM-AS2,LINC01265,MARCH11,PRLR,PRKAA1,MTMR12,MIR4458HG,FAM173B,FAM105A,GOLPH3,LOC101929153,LINC01020,C5orf34,UGT3A2,LOC257396,C5orf51,C5orf17,SLC1A3,LOC100132356,LOC642366,EGFLAM-AS4,MIR449B,DNAH5,C5orf28,PTGER4,CTNND2,MTRR,GDNF-AS1,LOC100287592,LOC101926940,LOC100506548,PAIP1,ZNF622,SNORA105B,LINC00603,ZNF131,CAPSL,MIR579,LOC153684,GZMA,LOC401177,ISL1,FGF10,MIR4636,FBXO4,LOC101929412,MCIDAS,MIR3650,CTD-2201E9.1,SNORA105A,ESM1,MIR449A,ANKH,NIM1K,C1QTNF3-AMACR,LOC285692,MIR4279,MED10,LOC648987,LOC101929454,DROSHA,TRIO,ROPN1L,TAS2R1,NADK2,LIFR-AS1,CCT5,C6,LOC102467080,BASP1,FBXL7,ANKRD33B,MOCS2,MIR449C,SNORD72,LOC101929524,PLCXD3,SNX18,ROPN1L-AS1,MIR887,DAP,SKP2,LOC101929681,CTD-2350J17.1,PAPD7,GUSBP1,MARCH6,ADCY2,RAD1,C5orf49,BRIX1,AGXT2,CTD-2297D10.2,ARL15,MIR580,TTC23L,OXCT1,CMBL,HMGCS1,NNT-AS1,MIR4278,RPL37,LOC340113,UGT3A1,RANBP3L,HCN1,LOC101929660,LINC01018,ITGA2,LINC01021,CDH6,MYO10,LOC100506406,CDH10,NNT,PELO,FYB,SRD5A1,NUP155,ANXA2R,PARP8,MRPS30,MROH2B,NSUN2,C5orf22,PDZD2,ZFR,GPX8,CARD6,PRDM9,IL7R,LOC100505625,SPEF2,MIR4458,EMB,LOC729506,LOC101929284,CCL28,LOC101926960,FAM134B,FGF10-AS1,TARS,LMBRD2	3	gain
3714	CGTE_46	5	140167086	140257143	5q31.3	PCDHA12,PCDHA11,PCDHA5,PCDHA9,PCDHA7,PCDHA2,PCDHA6,PCDHA1,PCDHA4,PCDHA10,PCDHA8,PCDHA3	0	loss
3715	CGTE_46	6	44374086	57372415	6p11.2-p21.1	IL17F,BMP5,CLIC5,PRIM2,GSTA5,ADGRF1,GSTA4,HCRTR2,CD2AP,ICK,LOC101927020,ANKRD66,CRISP1,KIAA1586,GSTA1,LOC101927136,TRAM2,SUPT3H,DEFB110,MIR586,MUT,RPS16P5,DST,DEFB133,LINCMD1,MIR548U,LOC100506188,ENPP5,GSTA3,CDC5L,GSTA7P,LOC101927082,LOC101927048,OPN5,KLHL31,CENPQ,C6orf141,HMGCLL1,PTCHD4,LOC101927211,TFAP2D,MLIP,LOC730101,MIR4642,MCM3,RAB23,PAQR8,RHAG,LRRC1,BEND6,MLIP-IT1,GFRAL,EFHC1,DEFB113,FAM83B,CRISP2,MIR5685,MIR133B,LOC101926915,FBXO9,TINAG,GSTA2,ADGRF4,PLA2G7,LOC101930010,RCAN2,ZNF451,GCLC,ENPP4,GLYATL3,BAG2,MEP1A,LOC101926898,LOC101926962,LINC01564,MIR206,LOC101927189,COL21A1,SLC25A27,CRISP3,TDRD6,CYP39A1,PKHD1,TRAM2-AS1,TFAP2B,DEFB114,TNFRSF21,PGK2,ADGRF2,GCM1,ADGRF5,ELOVL5,DEFB112,IL17A,TMEM14A,RUNX2	3	gain
3716	CGTE_46	7	193220	932031	7p22.3	GET4,SUN1,WI2-2373I1.2,FAM20C,LOC442497,DNAAF5,LOC101927000,LOC101926963,PDGFA,HRAT92,PRKAR1B	1	loss

3717	CGTE_46	7	933307	1786688	7p22.3	TFAMP1,CYP2W1,UNCX,LOC101927021,TMEM184A,MICALL2,ADAP1,ZFAND2A,ELFN1-AS1,COX19,MIR339,INTS1,GPR146,ELFN1,C7orf50,PSMG3-AS1,PSMG3,GET4,MAFK,GPER1	0	loss
3718	CGTE_46	7	7278153	23227726	7p21.3-p22.1	LOC401312,PER4,AGR3,IL6,LOC541472,AHR,SOSTDC1,RAPGEF5,RPL23P8,DGKB,LRRC72,MIOS,ABCB5,MIR1302-6,SNORD93,KLHL7-AS1,FAM126A,NUPL2,TOMM7,LOC101927630,LOC101927811,ICA1,LOC100506178,MIR3146,THSD7A,STEAP1B,AGR2,HDAC9,ISPD,TWISTNB,MEOX2-AS1,NXPH1,TSPAN13,AGMO,SP8,MEOX2,KLHL7,LOC101927391,LOC101927354,DNAH11,ITGB8,ANKMY2,SCIN,SNX13,PRPS1L1,ETV1,BZW2,LOC100505921,TMEM196,LINC01162,PHF14,KCCAT333,CDCA7L,RPA3,LOC100505938,LOC101927769,ISPD-AS1,COL28A1,C1GALT1,GLCCI1,VWDE,MACC1,ARL4A,MACC1-AS1,MIR1183,TMEM106B,UMAD1,TWIST1,NDUFA4,LOC101927668,FERD3L,SP4	3	gain
3719	CGTE_46	7	76825553	96747252	7q11.23-q21.13	ABCB1,STEAP2,PON1,C7orf76,PEX1,GNG11,MAGI2-AS2,LOC101927446,MAGI2,STEAP1,CDK14,RBM48,PHTF2,BET1,FGL2,ANKIB1,LOC101927269,LOC101409256,LOC101927497,LOC102723885,GATAD1,RUNDC3B,PPP1R9A,PEG10,SAMD9,CCDC146,LOC101927356,C7orf62,DMTF1,DLX5,PDK4,LOC101927378,SEMA3A,CYP51A1-AS1,SLC25A40,ADAM22,GTPBP10,HGF,SAMD9L,MIR548M,MIR591,LRRD1,MAGI2-AS3,CACNA2D1,KRIT1,GNGT1,SDHAF3,CDK6,SRI,APTR,DLX6-AS1,SGCE,LOC100128317,DYNC1I1,CD36,TP53TG1,LOC100506136,RPL13AP17,GSAP,MIR489,SEMA3D,PTPN12,ABCB4,DLX6,KIAA1324L,GNAI1,MIR4652,ZNF804B,GNAT3,SHFM1,STEAP2-AS1,CLDN12,CASD1,SLC25A13,AKAP9,CYP51A1,MIR653,TMEM243,CROT,DPY19L2P4,RSBN1L,VPS50,ASB4,GRM3,CALCR,MTERF1,CFAP69,TMEM60,HEPACAM2,FAM133DP,TFPI2,STEAP4,LOC101927243,DBF4,MGC16142,PCLO,PON3,FAM133B,PON2,SEMA3E,SEMA3C,COL1A2,FZD1	3	gain
3720	CGTE_46	7	100017559	102113545	7q22.1	MIR548O,EPHB4,ZASP,PRKRIP1,GIGYF1,GNB2,C7orf61,MIR4285,ZCWPW1,AP1S1,SPDYE6,LRWD1,ZNHIT1,PLOD3,PCOLCE,MYL10,UFSP1,ZAN,ALKBH4,AGFG2,ORAI2,MIR5090,NYAP1,MIR4653,SERPINE1,TRIM56,COL26A1,MUC12,SAP25,IFT22,ACTL6B,LOC101927746,FBXO24,MIR6875,MIR4467,POP7,TFR2,MEPCE,MOGAT3,CUX1,LOC102724094,MUC17,PPP1R35,MUC3A,TRIP6,LINC01007,EPO,TSC22D4,ACHE,LRCH4,SH2B2,NAT16,CLDN15,VGF,SRRT,SLC12A9,MOSPD3,LOC100630923,LOC100289561,PCOLCE-AS1,FIS1	1	loss
3721	CGTE_46	7	128470626	128544548	7q32.1	ATP6V1F,KCP,LOC100130705,FLNC	0	loss
3722	CGTE_46	7	148700064	150911137	7q36.1	LOC155060,GIMAP8,ZNF282,GIMAP1,ZNF862,AOC1,ABCB8,KRBA1,GIMAP1-GIMAP5,RARRES2,ATG9B,ASIC3,FASTK,CDK5,GIMAP5,ZNF777,LRRC61,AGAP3,ZNF398,ASB10,ZNF746,GIMAP7,PDIA4,TMEM176B,ABCF2,ZNF786,GBX1,KCNH2,REPIN1,SLC4A2,ZNF775,LINC00996,ZNF767P,ATP6V0E2-AS1,GIMAP6,GIMAP2,TMEM176A,GIMAP4,SSPO,IQCA1L,ATP6V0E2,LOC728743,ZNF467,TMUB1,ACTR3C,ZNF783,ZNF425,NOS3,ZNF212,ZBED6CL	1	loss
3723	CGTE_46	8	21959246	22006520	8p21.3	REEP4,HR,FAM160B2,NUDT18,LGI3	0	loss

3724	CGTE_46	8	67925179	142203036	8q22.2-q23.1	RNF139-AS1,LINC01591,DSCC1,TMEM71,PMP2,HAS2,NIPAL2,VPS13B,LAPTM4B,PTK2,EBAG9,LOC105375650,RRM2B,C8orf37,C8orf59,LINC01603,FABP4,KCNS2,PLEKHF2,CRISPLD1,LOC102724804,TCEB1,GDF6,MIR3150B,MIR5708,EIF3E,AZIN1-AS1,ATAD2,RBM12B,LOC100500773,MTSS1,FLJ46284,DPY19L4,ZHX1,TMEM75,RBM12B-AS1,ZFPM2-AS1,MRPL13,PCAT1,MIR3151,CCDC26,FABP5,LOC101927915,ZHX1-C8orf76,TERF1,SLC30A8,MIR5681A,SAMD12,STAU2-AS1,GEM,MIR1205,ESRP1,MIR4471,FBXO43,PDP1,CNGB3,OXR1,LINC00534,MIR8084,LINC01151,FABP12,LOC101927040,PHF20L1,ZFHX4-AS1,POU5F1B,SNORA72,LINC01030,FZD6,GDAP1,PKIA,C8orf88,TP53INP1,NOV,MIR3610,DENND3,RNF139,WISP1,SLCO5A1,NACAP1,PPP1R42,PSKH2,LINC01609,FER1L6-AS2,RIPK2,FAM83A-AS1,ENPP2,LOC104054148,DCSTAMP,AARD,SLC26A7,MIR4662A,PAG1,RIMS2,HPYR1,HHLA1,STK3,GSDMC,NSMCE2,LINC01608,MYC,LOC101927822,COPS5,RAD21-AS1,TRHR,ZFHX4,RAD21,TPD52,WWP1,UQCRB,MIR7848,SLC25A32,CCAT1,DCAF13,NCALD,C8orf76,TMEM64,TRPS1,EXT1,BAALC,TMEM70,MIR3150A,LOC101927543,ERICH5,NDUFB9,KLF10,C8orf89,MIR6844,STAU2,EMC2,LRP12,SULF1,CA13,C8orf37-AS1,ST3GAL1,MSC-AS1,FER1L6,SAMD12-AS1,SDC2,LOC101241902,NCRNA00250,PRNCR1,FBXO32,CSPP1,LRRCC1,HAS2-AS1,REXO1L2P,YWHAZ,MIR5194,FAM91A1,TSPYL5,LOC102724710,DECR1,HEY1,ZFAT,MIR5681B,FABP9,NCOA2,PRDM14,SQLE,LINC00861,DPYS,RPL30,ABRA,LINC01607,CA2,MAL2,HRSP12,LOC100288748,MMP16,LINC00964,FAM49B,LINC00535,TBC1D31,DEPTOR,OSGIN2,LINC00977,PKHD1L1,COL22A1,ZFPM2,FAM83A,LOC101927066,TRAPPC9,KCNB2,ATP6V1C1,BAALC-AS1,LRRC6,CHRAC1,SYBU,ZFAND1,CNBD1,LACTB2-AS1,CA3-AS1,ZNF572,WDYHV1,C8orf87,MIR875,ZBTB10,TAF2,ARFGEF1,ANGPT1,JPH1,PVT1,MIR2053,CASC9,ENY2,MTBP,TMEM74,LOC101926908,MIR2052HG,TRAM1,COLEC10,MIR599,COL14A1,TRMT12,TG,FER1L6-AS1,PABPC1,CA3,RMDN1,ODF1,LACTB2,CASC19,MIR4661,LINC01111,MIR7641-2,KCNK9,RPL7,E2F5,MIR1208,LINC00824,EYA1,MIR1204,KIAA1429,BAALC-AS2,LOC101927657,STMN2,ZC2HC1A,TMEM65,C8orf34-AS1,ASAP1,SNX16,SPAG1,SBSPON,ASAP1-IT2,LY96,DCAF4L2,CASC8,GRHL2,FAM84B,PEX2,CCAT2,RDH10-AS1,PTDSS1,RGS22,NBN,KIAA0196,COX6C,FAM135B,MIR1206,KCNQ3,LRRC69,MIR4663,CPA6,LINC01419,TMEM55A,ANKRD46,CTHRC1,C8orf34,RNF19A,UTP23,CHMP4C,LINC01592,RUNX1T1,MIR3149,NUDCD1,LOC105375734,IMPA1,RAD54B,EIF3H,MIR30D,LOC101926892,MTDH,CASC21,CALB1,KLHL38,CPQ,SLA,LOC101927845,IL7,MIR3686,MIR5680,TRIQK,ZFAT-AS1,FLJ42969,KCNV1,ZHX2,ADCY8,PCAT2,HNF4G,AGO2,ASAP1-IT1,OTUD6B-AS1,LINC00536,AZIN1,TMEM67,POP1,LINC01298,CPNE3,SLC7A13,MIR1273A,UBE2W,MSC,LOC101927798,FSBP,PREX2,MIR7705,CCNE2,MRPS28,UBR5-AS1,FAM92A1,LOC392232,OC90,INTS8,ZNF704,OSR2,MED30,TRIB1,MIR2052,CA1,UBR5,CASC11,RSPO2,SNX31,MATN2,LOC102724874,MTERF3,SLC10A5,RDH10,TATDN1,EFR3A,OTUD6B,RALYL,MIR4662B,NECAB1,ATP6V0D2,MIR378D2,MIR30B,ZNF706,ANXA13,SNTB1,KHDRBS3,NDRG1,XKR9,DERL1,MIR1207,CDH17,TRPA1,PKIA-	3	gain

3725	CGTE_46	8	142204169	145773883	8q24.3	LY6H,MIR6893,NRBP2,SHARPIN,GSDMD,SLC45A4,MIR6845,ARHGAP39,MIR4664,NAPRT,CYP11B2,CDC42P3,MIR7112,ZC3H3,FOXH1,MINCR,PLEC,LY6E,PTP4A3,SCRIB,DENND3,MIR661,KIFC2,SLURP1,LRRC14,SCX,GPT,EEF1D,MIR6846,MIR6848,SLC39A4,ZNF623,FAM83H,MAFA,MROH6,FAM83H-AS1,LINC01300,EPPK1,LY6D,ARC,GRINA,SPATC1,SLC52A2,ADCK5,ZFP41,MIR4472-1,HGH1,LOC100288181,JRK,CPSF1,MIR6847,LINC00051,CYC1,C8orf82,LOC100133669,MIR6849,TONSL,DGAT1,RHPN1,LYPD2,GLI4,PSCA,TONSL-AS1,LRRC24,THEM6,MAF1,HSF1,MIR1302-7,PYCRL,LY6K,BREA2,TMEM249,ADGRB1,ZNF696,ZNF707,PPP1R16A,OPLAH,MIR937,MROH5,MIR939,TSTA3,TSNARE1,VPS28,MFSD3,GPIHBP1,LYNX1,GML,FBXL6,BOP1,CYHR1,PUF60,TIGD5,CYP11B1,TOP1MT,MAPK15,PARP10,GPAA1,MIR4539,EXOSC4,RECQL4,WDR97,C8orf31,RHPN1-AS1,GPR20,MAFA-AS1,SCRT1,MROH1,CCDC166	0	loss
3726	CGTE_46	8	145780937	146035749	8q24.3	ARHGAP39,MIR6850,ZNF251,ZNF517,RPL8,ZNF34	1	loss
3727	CGTE_46	9	69002258	90499732	9q21.13-q21.31	PCSK5,MIR6130,MIR548H3,SPATA31D5P,UBQLN1,PGM5-AS1,LOC101927358,LOC440896,ALDH1A1,ANKRD20A4,FAM122A,CBWD3,TMEM2,GAS1,LINC01507,FOXD4L3,GNA14-AS1,SMC5,RASEF,MIR1299,C9orf153,TMC1,LOC100133920,FOXD4L5,LINC01474,RMI1,RORB,GNAQ,OSTF1,GNA14,PGM5,BANCR,FOXB2,PSAT1,FRG1HP,SMC5-AS1,LOC494127,C9orf57,TRPM6,C9orf41-AS1,LOC389765,LOC101927502,IDNK,ZFAND5,LOC101927450,NAA35,MAMDC2,C9orf135,MIR204,TLE1,CARNMT1,GAS1RR,PIP5K1B,FOXD4L4,PCA3,ZCCHC6,NMRK1,AGTPBP1,APBA1,ANXA1,LOC101927623,LOC101928381,FAM189A2,GCNT1,SPATA31D1,CEP78,PRUNE2,ABHD17B,SPATA31E1,SLC28A3,MAMDC2-AS1,CBWD5,DAPK1,FRMD3,SPATA31D3,LOC101927575,SPATA31D4,RFK,CTSLP8,C9orf85,GKAP1,HNRNPK,LINC01506,C9orf170,LOC440173,FXN,KLF9,C9orf40,KIF27,VPS13A-AS1,LOC392364,RPSAP9,PRKACG,C9orf135-AS1,TJP2,PGM5P2,VPS13A,NTRK2,FOXD4L6,CBWD6,ISCA1,GDA,RORB-AS1,TMEM252,C9orf64,LOC101927069,TLE4,MIR7-1,LINC01504,TRPM3,PTAR1,CTSL3P,CTSL,GOLM1	3	gain
3728	CGTE_46	9	138377911	139372146	9q34.3	SNAPC4,GPSM1,LINC01502,INPP5E,DKFZP434A062,KCNT1,LOC101928525,UBAC1,LCN9,QSOX2,C9orf116,LCN1,LHX3,PPP1R26,MRPS2,OBP2A,GLT6D1,PAEP,CARD9,NACC2,C9orf69,DNLZ,SDCCAG3,PMPCA,SOHLH1,CAMSAP1,SEC16A	1	loss
3729	CGTE_46	9	139378808	140395360	9q34.3	TMEM210,AGPAT2,NRARP,FAM166A,RNF224,CYSRT1,TRAF2,NOXA1,RABL6,LOC100128593,NSMF,MIR4479,MIR4673,NOTCH1,FAM69B,TMEM141,MAN1B1-AS1,GRIN1,MIR6722,EXD3,NDOR1,SAPCD2,TPRN,MIR7114,C9orf139,C8G,C9orf173-AS1,C9orf142,LCN10,C9orf173,LCN8,CLIC3,PNPLA7,TUBB4B,PHPT1,PTGDS,LCN6,DPP7,SNHG7,FUT7,NPDC1,MIR4292,ABCA2,SLC34A3,LRRC26,NALT1,MIR3621,EGFL7,SSNA1,EDF1,TOR4A,LCN12,FBXW5,UAP1L1,ANAPC2,TMEM203,C9orf163,CCDC183-AS1,RNF208,LCNL1,MAMDC4,C9orf172,CCDC183,MAN1B1,NELFB,SNORA17B,LCN15,ENTPD8,MIR126,SNORA17A,ENTPD2,MIR4674	0	loss

3730	CGTE_46	10	50944082	65281422	10q21.3-q21.2	PCDH15,NCOA4,CCDC6,UBE2D1,MIR1296,FAM13C,TMEM26,RTKN2,JMJD1C-AS1,ASAH2B,FAM133CP,LINC01553,NRBF2,ADO,EGR2,ARID5B,FAM21EP,ANK3,PRKG1,RHOBTB1,ZNF365,MIR548AV,PARGP1,CCEPR,TMEM26-AS1,TIMM23,PARG,CSTF2T,SGMS1,SGMS1-AS1,LINC00844,MBL2,TIMM23B,LOC102724719,LINC01468,A1CF,FAM21A,MRLN,BICC1,SLC16A9,PRKG1-AS1,TFAM,IPMK,MIR3924,REEP3,OGDHL,CISD1,MTRNR2L5,ZWINT,AGAP7P,MSMB,PHYHIPL,CDK1,LOC283045,JMJD1C,DKK1,AGAP6,MIR605,ASAH2,LINC00845,C10orf107	3	gain
3731	CGTE_46	10	102505931	104836906	10q24.31-q24.32	ELOVL3,SEMA4G,KAZALD1,LOC101927419,SFXN2,PITX3,LBX1-AS1,MIR3158-1,SFXN3,MIR146B,ARL3,LDB1,SUFU,RPARP-AS1,TLX1,MIR3158-2,FGF8,PSD,C10orf2,FBXW4,NOLC1,CYP17A1,TLX1NB,HPS6,LBX1,MFSD13A,WBP1L,KCNIP2,CUEDC2,AS3MT,C10orf95,MGEA5,LZTS2,BORCS7,POLL,ACTR1A,MRPL43,MIR608,PDZD7,PAX2,BTRC,SLF2,C10orf76,DPCD,CNNM2,FBXL15,TRIM8,LINC01514,NPM3,GBF1,PPRC1,BORCS7-ASMT,KCNIP2-AS1,NFKB2	1	loss
3732	CGTE_46	10	133967144	135381963	10q26.3	CFAP46,SPRNP1,MIR3944,KNDC1,JAKMIP3,FUOM,ADGRA1-AS1,STK32C,LINC01167,UTF1,NKX6-2,PRAP1,VENTX,PAOX,MIR202HG,LINC01168,ZNF511,C10orf91,SPRN,ECHS1,ADAM8,PWWP2B,CALY,SCART1,LINC01166,INPP5A,CYP2E1,MTG1,TUBGCP2,SYCE1,DPYSL4,LRRC27,MIR202,ADGRA1	1	loss
3733	CGTE_46	11	193051	2466762	11p15.5	KRTAP5-6,CRACR2B,TALDO1,PDDC1,IFITM1,LRRC56,CHID1,MOB2,IFITM10,LSP1,MUC6,TSSC4,MIR675,MUC5B,MRPL23-AS1,LINC01219,TNNT3,SYT8,MIR4686,MIR7847,IRF7,IFITM5,KRTAP5-3,NLRP6,SCGB1C2,TRPM5,HRAS,MIR210HG,TMEM80,C11orf21,TSPAN32,CDHR5,PIDD1,MUC2,H19,IFITM3,ODF3,KRTAP5-1,BRSK2,AP2A2,PKP3,SCGB1C1,ANO9,LOC171391,B4GALNT4,TOLLIP-AS1,CEND1,KRTAP5-AS1,DUSP8,TOLLIP,IGF2,PANO1,DEAF1,FAM99A,TNNI2,RPLP2,EPS8L2,MRPL23,CD151,SIGIRR,SCT,CD81,PTDSS2,MIR6744,RNH1,SLC25A22,CD81-AS1,PNPLA2,MIR210,IFITM2,KRTAP5-4,LINC01150,MIR483,KCNQ1,MIR4298,MUC5AC,BET1L,PSMD13,KRTAP5-2,RIC8A,MIR6743,TH,SIRT3,FAM99B,INS,HOTS,SNORD131,PHRF1,KRTAP5-5,TSPAN4,INS-IGF2,LOC143666,ATHL1,IGF2-AS,CTSD,ASCL2,SNORA52,DRD4,LMNTD2,RASSF7,POLR2L	0	loss

3734	CGTE_46	11	3380963	8704461	11p15.4	MRPL17,OR52E4,OR51I1,TRIM34,OR10A2,OR10A6,TIMM10B,RHOG,MIR4687,OR51G1,LOC100506258,ZNF214,BGLT3,ILK,RRP8,OR56A1,OR51B2,CASC23,ART5,OR52B4,OR5P2,OR51I2,CNGA4,OR52A5,TUB-AS1,LMO1,OR56B4,UBQLN3,RPL27A,OR52K2,OR5E1P,RBMXL2,TRIM6-TRIM34,CCKBR,OR51E2,OR51B5,PPFIBP2,OLFML1,OR56A3,TRIM6,OLFM5P,OR51V1,CYB5R2,TRIM5,PRKCDBP,OR52D1,ART1,OR2AG2,OR2D3,OR52J3,TRIM3,OR51B4,HPX,OR51A2,OR52R1,OR52W1,LOC283299,OVCH2,OR52E6,OR51A7,OR51F1,PGAP2,MMP26,OR51T1,OR51F2,OR51E1,UBQLNL,TPP1,OR51B6,OR6A2,OR51A4,ZNF195,OR51S1,OR7E12P,OR2AG1,HBBP1,OR51M1,TRIM66,OR52N1,LOC101927708,MIR302E,ARFIP2,OR2D2,OR56A4,ZNF215,STK33,TUB,SMPD1,OR52B6,HBE1,RRM1,NUP98,OR51D1,LOC100506082,OR52H1,SYT9,C11orf42,OR56B1,CHRNA10,C11orf40,TRIM21,OR52M1,OR52N4,DCHS1,OR52K1,OR5P3,OR52B2,OR10A5,HBB,STIM1,FAM160A2,HBG1,OR52I2,NLRP10,EIF3F,OR51Q1,OR51L1,OR56A5,NLRP14,OR52A1,OR52N2,OR10A3,TRPC2,GVINP1,OR52E2,OR52E8,TSSC2,HBD,RIC3,OR51G2,TRIM68,OR10A4,OR52N5,OR52I1,DNHD1,APBB1,HBG2,TRIM22,OR52L1,TAF10	3	gain
3735	CGTE_46	11	63671203	67818329	11q13.1-q13.2	KCNK4,PPP2R5B,BANF1,DPF2,MIR194-2,AP5B1,TBX10,SYT12,PPP1R14B,NAALADL1,MIR6879,CARNS1,SF1,PC,CTSW,LOC100996455,TMEM134,B4GAT1,MALAT1,VEGFB,MRPL11,SPDYC,SNX15,CFL1,SSSCA1-AS1,EHD1,KCNK4-TEX40,ANKRD13D,C11orf80,DNAJC4,FLRT1,MIR3163,RELA,RHOD,SCYL1,NUDT8,CCS,SART1,TSGA10IP,CLCF1,TM7SF2,PCNXL3,EFEMP2,UNC93B1,NAA40,FAM86C2P,C11orf85,SF3B2,FIBP,MIR192,NPAS4,MIR612,RBM4,MACROD1,SLC25A45,CD248,ALDH3B2,CAPN1,DOC2GP,CATSPER1,GPHA2,TRPT1,RPS6KB2,OVOL1,ZFPL1,MIR6860,PPP1CA,DRAP1,RNASEH2C,C11orf72,FOSL1,LTBP3,FKBP2,PTPRCAP,VPS51,NDUFV1,DPP3,GAL3ST3,CNIH2,POLD4,RCOR2,NEAT1,MIR6752,RAD9A,MRPL49,FAM89B,PLCB3,EIF1AD,CCDC87,MARK2,CDC42BPG,CTSF,SLC22A20,KLC2,TIGD3,SSH3,KDM2A,OTUB1,RBM14-RBM4,MEN1,GPR137,PRDX5,MIR7155,MIR4690,TMEM262,CABP4,BRMS1,C11orf68,MIR6751,RAB1B,KAT5,MUS81,OVOL1-AS1,ATG2A,GPR152,PACS1,ADRBK1,ACY3,MIR6749,SLC29A2,MAP4K2,TBC1D10C,SAC3D1,CST6,NRXN2,AIP,FRMD8,ZDHHC24,SNX32,MAP3K11,CABP2,MIR4489,RBM14,STIP1,COX8A,FAU,LOC100130987,TCIRG1,SPTBN2,LOC101928069,LRFN4,TEX40,POLA2,TMEM151A,SIPA1,MIR7113,ACTN3,YIF1A,ESRRA,ZNHIT2,SYVN1,ARL2-SNX15,NUDT22,CORO1B,PITPNM1,BAD,SLC22A11,MIR4691,CDC42EP2,RASGRP2,CDK2AP2,PELI3,RPS6KA4,ARL2,MIR1234,MIR1237,GSTP1,FERMT3,RIN1,CCDC85B,C11orf86,RBM4B,KCNK7,MIR194-2HG,BATF2,MIR6753,TRMT112,SSSCA1,EHBP1L1,MIR6750,BBS1,NDUFS8,PYGM,SLC22A12,ALDH3B1,CCDC88B,RCE1,CDCA5	1	loss

3736	CGTE_46	12	7285589	49164831	12p13.31-p12.2	GDF3,SCAF11,PRH1-PRR4,FAM234B,ADCY6,RERG,CASC1,BCAT1,TAS2R8,CPNE8,C12orf77,ARNTL2-AS1,PEX5,SENP1,YARS2,DENND5B-AS1,PDE3A,ZNF705A,CCDC184,TAS2R31,LOC101928162,MRPS35,LALBA,DERA,SMCO3,PPFIBP1,PZP,CLEC2D,NANOG,CNTN1,BCL2L14,ANP32D,TMEM52B,DDX47,FAM90A1,LOC642846,SMIM10L1,TAS2R42,PLBD1-AS1,SLCO1B7,CLEC4A,SKP1P2,KIAA1551,ATF7IP,MIR7851,PLEKHA5,STK38L,LMNTD1,TAS2R50,LOC101927905,MIR1244-4,CD163L1,MUC19,TAS2R30,LINC01252,ETV6,CCDC91,LRP6,ANO6,CD163,LINC00987,LINC01489,PYROXD1,PRICKLE1,MIR3974,ARNTL2,LYRM5,SLCO1B3,MIR613,C12orf54,HDAC7,PPHLN1,RACGAP1P,HIST4H4,HTR7P1,TMEM106C,ABCD2,BHLHE41,ETNK1,MIR4302,KLRC1,LOC101928441,LOC101928100,TMTC1,RPAP3,CLEC1B,REP15,AEBP2,WBP11,MIR6505,SLCO1B1,ALG10B,MIR1244-2,PRB4,SLCO1C1,PHC1,PLCZ1,M6PR,FAM66C,SLC48A1,MIR1291,BORCS5,AMN1,GUCY2C,LINC00612,CLEC4D,C12orf40,SLC38A4,LINC00938,SMCO2,LOC101930452,RAPGEF3,BICD1,TSPAN11,AICDA,LINC00477,SLCO1A2,PFKM,CLEC7A,PUS7L,RPL13AP20,YAF2,DDX11,CMAS,GPRC5D,CD69,MIR1244-1,OR10AD1,SNORA2B,RECQL,APOLD1,KRAS,TMEM117,MANSC4,TWF1,OVCH1,SYT10,CLEC4E,LOC100506159,A2ML1,PKP2,CLEC4C,LINC00941,CCNT1,CLEC12A,LOC101928030,PDE6H,PCED1B,ASB8,PRH1,PDZRN4,FLJ13224,TM7SF3,MIR1244-3,TAS2R43,KIF21A,C2CD5,HEBP1,SNORA2C,LRMP,MGST1,NANOGNB,PRR4,RASSF8-AS1,ALG10,FAM60A,MGP,KLRK1,LOC374443,ASUN,GABARAPL1,ST8SIA1,GXYLT1,DDX12P,ARHGDIB,ITPR2,METTL20,RERG-AS1,LINC00937,PRH2,PRB2,MANSC1,ZCRB1,SLC2A14,LDHB,SLC2A13,KANSL2,GYS2,A2M,KLRD1,ABCC9,A2MP1,MED21,MIR920,KLRC4,POU5F1P3,MIR614,C12orf71,DNM1L,LOC100506314,TAS2R10,KCNJ8,SLC38A2,GPRC5A,IPO8,MIR4494,LOC102724020,MFAP5,AMIGO2,RERGL,H1FNT,KLRC4-KLRK1,TAS2R9,MIR7641-2,IRAK4,TAS2R13,RASSF8,PTPRO,APOBEC1,CDKN1B,STYK1,CLEC12B,LOC100506606,ADAMTS20,TAS2R20,SSPN,LMO3,PRH1-TAS2R14,GRIN2B,DDX11-AS1,KLRF1,OVCH1-AS1,CLEC2B,DBX2,CLEC1A,MIR4698,ACSM4,TAS2R7,GPR19,KLRB1,C3AR1,STRAP,ERGIC2,CLEC2A,SLC38A1,ART4,DENND5B,KLRG1,CLECL1,ARID2,LOC100288798,PRB3,GOLT1B,TAS2R14,ERP27,LOH12CR2,EMP1,TAS2R19,LOC101927058,OLR1,DPPA3,PRB1,LOC101928471,CAPZA3,SOX5,FGFR1OP2,LOC100506393,FOXJ2,LRRK2,PLBD1,CAPRIN2,ENDOU,FAM86FP,KLRC2,PIK3C2G,A2M-AS1,SNORA2A,PCED1B-AS1,SLC15A5,YBX3,KLRC3,RIMKLB,COL2A1,EPS8,ZNF641,KLRAP1,CLSTN3,LINC00935,VDR,IAPP,H2AFJ,DUSP16,H3F3C,KLRF2,NECAP1,NELL2,LINC01559,TAS2R46,RNY5,C12orf60,CLEC6A,PLEKHA8P1,FAR2,FGD4,MAGOHB,GSG1,SPX,CREBL2,SLC2A3,PTHLH,KLHL42,OR8S1,CLEC9A	3	gain
3737	CGTE_46	12	132271024	132414668	12q24.33	MMP17,ULK1,PUS1,SFSWAP	0	loss
3738	CGTE_46	13	53419777	53422629	13q14.3	PCDH8	0	loss
3739	CGTE_46	14	104552082	106382699	14q32.33	MIR8071-2,SIVA1,MIR8071-1,TEX22,MIR4710,ELK2AP,ADSSL1,LOC100507437,AHNAK2,PLD4,MIR203B,C14orf79,MIR203A,ASPG,LOC102723354,INF2,TMEM121,CDCA4,AKT1,C14orf80,CRIP1,LINC00638,MIR6765,BTBD6,MIR4539,MTA1,MIR4538,CEP170B,C14orf180,TMEM179,MIR4507,GPR132,CRIP2,PACS2,KIF26A,MIR4537,BRF1,ZBTB42,NUDT14,JAG2	0	loss
3740	CGTE_46	15	41864558	41865251	15q15.1	TYRO3	66	gain

3741	CGTE_46	15	42134665	42174975	15q15.1	PLA2G4B,SPTBN5,MIR4310,JMJD7-PLA2G4B	0	loss
3742	CGTE_46	15	74244257	74281138	15q24.1	STOML1,LOXL1	8	gain
3743	CGTE_46	15	77327771	77329533	15q24.3	PSTPIP1	22	gain
3744	CGTE_46	15	91418941	91448648	15q26.1	FES,FURIN,MAN2A2	0	loss
3745	CGTE_46	16	97450	135924	16p13.3	NPRL3,RHBDF1,MPG,SNRNP25,POLR3K	1	loss
3746	CGTE_46	16	136718	674941	16p13.3	RGS11,NPRL3,RAB40C,HBZ,NME4,DECR2,HBA2,RAB11FIP3,PDIA2,LOC100134368,MIR3176,LUC7L,MRPL28,FAM234A,MIR5587,TMEM8A,HBQ1,PIGQ,ARHGDIG,PRR35,CAPN15,LINC00235,HBA1,HBM,AXIN1,NHLRC4	0	loss
3747	CGTE_46	16	674952	676112	16p13.3	RAB40C	4	gain
3748	CGTE_46	16	677226	3199803	16p13.3	MSRB1,CLDN9,C16orf91,MIR3677,SSTR5-AS1,PRSS22,CASKIN1,TNFRSF12A,RPL3L,TPSB2,RPS2,TELO2,GNG13,TSC2,MRPS34,IL32,BAIAP3,STUB1,MIR6511B1,ERVK13-1,TMEM204,UNKL,LINC00514,ZNF213-AS1,IGFALS,MIR4516,LOC652276,CRAMP1,PRSS21,WFIKKN1,HAGH,SLC9A3R2,MIR3177,ABCA17P,ZSCAN10,TCEB2,UBE2I,MMP25,TBL3,BRICD5,ATP6V0C,DNASE1L2,LMF1,KREMEN2,LINC00254,LMF1-AS1,PDPK1,MSLN,C1QTNF8,CCDC64B,FAM173A,PRR25,NDUFB10,CEMP1,NARFL,RHBDL1,WDR90,LOC106660606,CCDC154,THOC6,EME2,SRRM2-AS1,TSR3,SNORA64,CLDN6,MMP25-AS1,SYNGR3,C16orf13,MIR940,MIR3180-5,TRAF7,NTN3,FBXL16,ZNF205,JMJD8,HCFC1R1,CACNA1H,HS3ST6,MIR4717,PTX4,RAB40C,PKD1,LOC100128770,ZNF205-AS1,NUBP2,ZNF598,METRN,SNORA10,C16orf59,E4F1,CCDC78,KCTD5,ECI1,FAM195A,SRRM2,MIR6511B2,RNF151,SNHG19,PRSS41,PRSS30P,ZG16B,GNPTG,MIR3178,MLST8,TPSD1,MIR6768,GFER,NTHL1,NME3,WDR24,RPUSD1,PRSS27,PRSS33,SOX8,PAQR4,MEIOB,SNORD60,PGP,SSTR5,FLJ42627,NPW,FAHD1,IFT140,MIR1225,FLYWCH2,TBC1D24,SNORA78,TPSAB1,SNHG9,RHOT2,TPSG1,ABCA3,LOC101929613,RNPS1,HN1L,RAB26,ZNF213,CASP16P,CCNF,CHTF18,HAGHL,MIR662,CLCN7,FLYWCH1,SPSB3,NOXO1,AMDHD2,PKMYT1,MAPK8IP3,MIR6767	0	loss
3749	CGTE_46	16	4644630	4749159	16p13.3	C16orf96,NUDT16L1,ANKS3,MIR6769A,UBALD1,MGRN1	0	loss
3750	CGTE_46	16	28222361	34578611	16p11.2	ZNF668,BOLA2B,YPEL3,QPRT,FUS,LAT,HSD3B7,MIR4517,LOC730183,RABEP2,PHKG2,CLUHP3,BCL7C,MIR3680-2,ITGAD,ZNF764,MIR3680-1,CD2BP2,ZNF843,CORO1A,IL27,ORAI3,TMEM265,SMG1P5,SMG1P2,SPN,SLC5A2,ZNF48,ITGAM,XPO6,KCTD13,LOC390705,NFATC2IP,UBE2MP1,STX4,TRIM72,FBXL19-AS1,CDIPT,SNX29P2,LOC613037,SULT1A4,GDPD3,ALDOA,TP53TG3C,SRCAP,SH2B1,CLN3,AHSP,ZNF629,ZNF785,NUPR1,MIR4518,VKORC1,EIF3CL,PYCARD-AS1,C16orf54,RNF40,KAT8,YBX3P1,MIR6862-2,DOC2A,SLC7A5P1,ZNF771,PYDC1,HERC2P4,SEZ6L2,TMEM219,TP53TG3D,ZG16,LINC00273,SEPHS2,RRN3P2,NPIPB8,ENPP7P13,ARMC5,CCDC189,ATP2A1,LOC613038,FAM57B,CTF1,ZNF646,ZNF267,TBX6,CDIPT-AS1,LOC606724,SULT1A1,TBC1D10B,ZNF689,BOLA2,FBRS,SLX1A-SULT1A3,MIR4519,PRSS36,MIR4721,ITGAX,PAGR1,TAOK2,ATXN2L,SLC6A10P,PYCARD,MAPK3,SLX1B-SULT1A4,FBXL19,ZNF768,NPIPB6,MIR762HG,ZNF720,SNORA30,HIRIP3,MYLPF,CD19,PRRT2,KIF22,SULT1A3,MVP,PPP4C,ZNF747,C16orf58,APOBR,NPIPB11,PRSS8,PRSS53,ZNF688,MIR6862-1,LOC388242,NPIPB9,STX1B,ITGAL,INO80E,TUFM,SLX1B,BCKDK,SULT1A2,C16orf92,EIF3C,DCTPP1,TP53TG3B,PRR14,MIR762,ASPHD1,SEPT1,SPNS1,MAZ,TGFB1I1,SBK1,ATP2A1-AS1,COX6A2,SETD1A,TP53TG3,SGF29,SLX1A	1	loss

3751	CGTE_46	16	67180054	67245101	16q22.1	NOL3,E2F4,ELMO3,KIAA0895L,MIR328,C16orf70,TRADD,EXOC3L1,LRRC29,B3GNT9,HSF4,FBXL8	0	loss
3752	CGTE_46	16	70697469	70726923	16q22.1	MTSS1L,VAC14	0	loss
3753	CGTE_46	16	75498110	75600484	16q23.1	GABARAPL2,TMEM231,CHST5,CHST6,TMEM170A	0	loss
3754	CGTE_46	16	75600732	84165041	16q23.1-q23.2	MIR4719,SLC38A8,SDR42E1,LINC01227,GCSH,HSDL1,LOC102724163,GABARAPL2,NECAB2,MIR7854,PKD1L2,MIR8058,CDYL2,LOC100129617,CNTNAP4,CDH13,MIR3182,CENPN,MIR6504,VAT1L,CMC2,KARS,HSD17B2,MAF,ADAT1,PLCG2,OSGIN1,BCO1,ADAMTS18,LOC101928446,LINC01229,TERF2IP,MIR4720,LOC101928203,MAFTRR,GAN,LOC101928417,CLEC3A,MPHOSPH6,NUDT7,LOC102724084,MLYCD,CMIP,PRCAT47,ATMIN,DYNLRB2,MON1B,MBTPS1,SYCE1L,C16orf46,HSBP1,WWOX	3	gain
3755	CGTE_46	16	88052065	89268072	16q24.2-q24.3	MIR5189,MIR4722,ZC3H18,LINC00304,GALNS,CDT1,SNAI3-AS1,PIEZO1,LOC101928880,BANP,RNF166,CTU2,PABPN1L,CYBA,APRT,MVD,ZNF469,TRAPPC2L,ZFPM1,ACSF3,CDH15,LOC339059,IL17C,CBFA2T3,LOC100289580,LOC100129697,LOC400558,SNAI3,LOC400553,SLC22A31	0	loss
3756	CGTE_46	16	90076311	90142246	16q24.3	URAHP,GAS8-AS1,DBNDD1,GAS8,PRDM7	3	gain
3757	CGTE_46	17	6944845	8137805	17p13.1	ZBTB4,TMEM256,ALOX15B,ACADVL,GUCY2D,SNORA48,MPDU1,YBX2,CLDN7,TMEM95,TP53,CYB5D1,LOC284023,NLGN2,PHF23,TNFSF12,FXR2,SOX15,EFNB3,SAT2,CTDNEP1,ALOXE3,SPEM1,TMEM88,TMEM102,SLC16A11,TNK1,SHBG,KCNAB3,ATP1B2,WRAP53,SLC2A4,SENP3,DLG4,KDM6B,TNFSF12-TNFSF13,NEURL4,MIR324,SCARNA21,SNORD10,NAA38,GABARAP,LINC00324,EIF5A,CHD3,CNTROB,MIR4314,KCTD11,GPS2,POLR2A,ELP5,ALOX12B,MIR4521,TNFSF13,DNAH2,VAMP2,TMEM107,FGF11,CD68,SLC35G6,EIF4A1,MIR6883,SNORA67,SENP3-EIF4A1,RPL29P2,AURKB,ASGR1,BORCS6,CTC1,CHRNB1,TRAPPC1,ACAP1,C17orf74,PER1,CLEC10A,HES7,TMEM256-PLSCR3,ASGR2,DVL2,PLSCR3	1	loss
3758	CGTE_46	17	27893041	27949938	17q11.2	ABHD15,GIT1,TP53I13,CORO6,ANKRD13B	0	loss

3759	CGTE_46	17	72764824	81083625	17q25.2-q25.1	MIR4739,UNK,SLC26A11,USH1G,UBE2O,C17orf62,SLC9A3R1,GALR2,USP36,MXRA7,TIMP2,PCYT2,ARHGDIA,SUMO2,ARMC7,RECQL5,C17orf89,CDR2L,HN1,ACOX1,MYADML2,ANAPC11,HP09025,SNORD134,PGS1,SGSH,NOTUM,DNAH17-AS1,LOC100294362,RBFOX3,NARF,FASN,STRA13,WBP2,SNHG16,OTOP3,FAM195B,SIRT7,LGALS3BP,SLC16A3,WDR45B,CYTH1,FDXR,SECTM1,GAA,LOC100287042,GCGR,MIR4316,MIF4GD,SEC14L1,SLC25A19,FN3K,CCDC137,SOCS3,LOC105274304,CEP131,SRSF2,PPP1R27,TMC8,TMEM235,MIR657,ZNF750,ATP5H,RPTOR,EIF4A3,NUP85,TNRC6C-AS1,SMIM6,RNF213,EXOC7,OGFOD3,MIR3678,CBX8,BAIAP2-AS1,MIR6785,MGAT5B,PRCD,ICT1,CEP295NL,UTS2R,ENTHD2,SCARNA16,TEN1,ZACN,PDE6G,MRPL38,NT5C,SAP30BP,CHMP6,C17orf99,BAIAP2,MIR4538,DCXR,SYNGR2,HGS,MIR338,CASKIN2,ARL16,TMEM94,ASPSCR1,MIR6786,ALYREF,MIR4738,TMEM104,HEXDC,CBX4,CANT1,SNHG20,GRIN2C,TRIM65,SMIM5,MFSD11,UNC13D,GRB2,OTOP2,MAFG,SNORD1A,OXLD1,LOC101928674,BAHCC1,CSNK1D,TBC1D16,FN3KRP,CDK3,MIR636,MRPL12,MIR6787,MIR4730,TSEN54,FAAP100,ACTG1,LRRC45,FBF1,LOC100132174,AFMID,ENDOV,CARD14,FSCN2,ST6GALNAC2,GALK1,MRPS7,MAFG-AS1,NPB,ENGASE,LOC101928514,FLJ45079,MIR3065,CCDC40,RAB40B,NPLOC4,QRICH2,CD7,MIR1250,HID1-AS1,MIR6516,FADS6,AATK,KCTD2,TNRC6C,TMEM105,LOC100996291,HID1,NPTX1,GPS1,METRNL,UBALD2,LINC00482,RNF157-AS1,NAT9,MYO15B,SPHK1,LOC101928710,CYGB,LOC101928855,TRIM47,SEPT9,SNORD1C,EVPL,RFNG,SLC25A10,LLGL2,TEN1-CDK3,CCDC57,PRPSAP1,TSPAN10,H3F3B,GGA3,SLC16A5,MIR4525,AANAT,BIRC5,RNF157,SRP68,LOC100507351,TEX19,LOC100130370,C1QTNF1,DUS1L,MIR3186,AATK-AS1,ENPP7,LOC101928738,P4HB,PYCR1,B3GNTL1,FOXK2,ITGB4,DNAH17,RHBDF2,FOXJ1,MIR6868,METTL23,RAC3,SNORD1B,TMC6,CBX2,TBCD,C1QTNF1-AS1,MIR4740,ST6GALNAC1,JMJD6,TK1,LOC101928766,SLC38A10	1	loss
3760	CGTE_46	19	281411	3820466	19p13.3	CIRBP-AS1,SCAMP4,TBXA2R,ADAMTSL5,AMH,PLPPR3,MIR6789,C19orf71,ATP8B3,ZNF555,ADAT3,MIER2,MIDN,HMG20B,TPGS1,PCSK4,THOP1,TCF3,HCN2,OAZ1,ELANE,APC2,GADD45B,CELF5,MIR4321,C2CD4C,ODF3L2,NDUFS7,MIR1268A,MED16,MEX3D,MIR7850,STK11,MADCAM1,ARID3A,AES,SGTA,PRTN3,ZNF57,AP3D1,SBNO2,GNA11,TLE2,REXO1,CBARP,TMEM259,ZNF554,UQCR11,MIR7108,FZR1,CACTIN-AS1,WDR18,PLPP2,PLEKHJ1,KISS1R,LOC100996351,RAX2,ZNF556,C19orf35,ABCA7,CSNK1G2-AS1,BSG,TIMM13,LOC100288123,THEG,TLE6,PLK5,RPS15,MIR1227,GNA15,TMPRSS9,MATK,PTBP1,ZNF77,DOHH,CFD,MISP,NCLN,KLF16,REEP6,GRIN3B,PALM,POLR2E,TJP3,ONECUT3,C19orf24,FGF22,MKNK2,ZFR2,HMHA1,CNN2,C19orf25,PIP5K1C,DOT1L,MBD3,POLRMT,JSRP1,LINGO3,NFIC,ATP5D,SHC2,APBA3,SF3A2,MOB3A,GIPC3,CACTIN,DIRAS1,IZUMO4,DAZAP1,MIR1909,ABHD17A,RNU6-2,MIR4745,FSTL3,GNG7,R3HDM4,S1PR4,EFNA2,PRSS57,MUM1,MIR3187,LMNB2,MFSD12,CDC34,LSM7,CIRBP,GPX4,CSNK1G2,SMIM24,MRPL54,AZU1,GAMT,RNF126,SLC39A3,SPPL2B,GZMM,BTBD2	0	loss

3761	CGTE_46	19	3821363	6670132	19p13.3	MIR4747,CREB3L3,UHRF1,ARRDC5,TICAM1,TUBB4A,SIRT6,CRB3,PLIN3,MPND,TNFAIP8L1,ACER1,ALKBH7,DPP9-AS1,DPP9,TNFSF9,CATSPERD,MAP2K2,MIR6885,SAFB,PRR22,MIR1268A,DUS3L,C19orf70,LOC101928844,RPL36,SEMA6B,SLC25A41,KHSRP,MIR3940,HDGFRP2,STAP2,TMIGD2,PTPRS,MIR7-3HG,NRTN,FSD1,TNFSF14,CAPS,TINCR,LRG1,MLLT1,PIAS4,SLC25A23,ZNRF4,EEF2,HSD11B1L,SHD,EBI3,UBXN6,PLIN5,CHAF1A,MIR7-3,MIR6790,ZBTB7A,FEM1A,DENND1C,MIR4746,SAFB2,MIR637,CD70,FUT3,LOC100128568,NDUFA11,SH3GL1,DAPK3,PLIN4,ATCAY,KDM4B,LONP1,NMRK2,ACSBG2,FUT5,RANBP3,GTF2F1,VMAC,ZFR2,ANKRD24,RFX2,PSPN,FUT6,SNORD37,CLPP,CCDC94,MYDGF	1	loss
3762	CGTE_46	19	7524671	8601494	19p13.2	PRR36,EVI5L,MCOLN1,TRAPPC5,ELAVL1,XAB2,FCER2,CLEC4M,C19orf45,MIR6792,TGFBR3L,CTXN1,TIMM44,NDUFA7,ZNF414,MIR4999,LOC100128573,PCP2,CD320,ARHGEF18,ANGPTL4,CLEC4G,CLEC4GP1,FBN3,PEX11G,CD209,ZNF358,RAB11B,RETN,CERS4,PNPLA6,SNAPC2,RPS28,RAB11B-AS1,KANK3,STXBP2,MAP2K7,MARCH2,CCL25,MCEMP1,PET100,LYPLA2P2,CAMSAP3,PRAM1,MYO1F,LRRC8E,HNRNPM	1	loss

3763	CGTE_46	19	45494459	51358327	19q13.32-q13.33	BBC3,CARD8-AS1,NKPD1,C19orf48,RASIP1,HSD17B14,ARHGAP35,SULT2B1,CARD8,SNORD35A,C5AR1,TRAPPC6A,RRAS,ASPDH,RPL18,SNAR-G1,TRPM4,MIR642A,SNORD35B,IGFL3,GLTSCR2,NOSIP,CPT1C,SULT2A1,GIPR,FOXA3,SNAR-A4,RPS11,CGB1,GLTSCR2-AS1,SNAR-A5,RSPH6A,PNKP,SPACA4,PNMAL2,IL4I1,SNAR-A12,MIR6799,RUVBL2,ADM5,BLOC1S3,CLPTM1,POLD1,BHMG1,EXOC3L2,SNORD32A,FCGRT,CGB7,KLK15,SYMPK,LIN7B,KLK1,SNAR-A9,FOSB,SNAR-A10,PRKD2,NR1H2,LOC400706,EML2-AS1,SNAR-A1,DKFZp434J0226,SNAR-A2,SNAR-F,FTL,MIR642B,SNAR-A13,FGF21,NTF4,NAPSA,CGB2,TPRX1,BCAT2,MAMSTR,CD3EAP,SYNGR4,SHANK1,NOVA2,GPR32,NAPA,SNAR-A6,FAM71E1,IGFL1,KLC3,MIR6088,FUZ,FUT1,NAPSB,SLC6A16,PRRG2,PPP5C,PTOV1-AS2,ACPT,SNAR-G2,MYH14,CCDC155,SNAR-A11,PIH1D1,LOC101928295,EMC10,SIGLEC16,NUCB1-AS1,GEMIN7,SNAR-A8,HRC,NTN5,SIGLEC11,ALDH16A1,EHD2,SNORD88C,PRR12,NANOS2,PLA2G4C,MIR769,GRWD1,CCDC61,DACT3,SNAR-C1,STRN4,GYS1,PLEKHA4,DMPK,MIR3191,KCNC3,KCNJ14,PGLYRP1,CABP5,SNRPD2,CLASRP,SNORD88B,CRX,CALM3,TSKS,ELSPBP1,OPA3,SLC1A5,TBC1D17,BSPH1,C19orf68,SPHK2,GNG8,LOC101059948,FLT3LG,IRF3,KLK3,PNMAL1,KDELR1,DHDH,FAM83E,PTGIR,CYTH2,ZC3H4,SNORD33,CGB8,SNAR-A7,CA11,MIR6798,CCDC9,SLC8A2,KCNA7,SNAR-A14,ZNF541,MIR3190,SLC17A7,GPR4,DMWD,LHB,QPCTL,SEPW1,KPTN,MIR4324,TMEM160,MIR320E,SNAR-C5,BCL2L12,PPM1N,SEC1P,ATF5,NPAS1,SNORD34,LRRC4B,AP2A1,PTH2,MIR4751,EMP3,NUCB1,C19orf73,SNAR-D,RPL13AP5,FUT2,TULP2,FKRP,SNAR-E,NAPA-AS1,SCAF1,LOC105447645,IGFL4,ZNF473,SNAR-B2,SNAR-C2,SNAR-C4,SIX5,VASP,NUP62,RTN2,BAX,PRMT1,PPP5D1,PTOV1-AS1,AP2S1,MIR330,RELB,MGC45922,ZNF296,INAFM1,PPP1R37,DACT3-AS1,SYT3,IGFL2,MYBPC2,FBXO46,VRK3,MARK4,CGB,SNAR-B1,PPFIA3,SNORD88A,CD37,TEAD2,DBP,PPP1R15A,PTOV1,CLEC11A,ERCC2,GLTSCR1,IRF2BP1,RPL13A,ZNF114,MYPOP,MIR5088,FLJ26850,LMTK3,MIR4749,AKT1S1,CGB5,SAE1,LIG1,DKKL1,DHX34,PLA2G4C-AS1,SNORD23,MIR150,C5AR2,MED25,SNAR-C3,MIR4750,SNRNP70,RCN3,IZUMO1,SNAR-A3,SPIB,IZUMO2,JOSD2,TMEM143,C19orf81,HIF3A,LOC105372441,CCDC114,CKM,GRIN2D,ERCC1,CCDC8,MIR6800,GFY,EML2,MEIS3,PPP1R13L	1	loss
3764	CGTE_46	19	55720776	56172064	19q13.42	HSPBP1,ZNF524,MIR6804,TMEM150B,COX6B2,ZNF579,ZNF784,NAT14,CCDC106,TMEM190,FIZ1,SHISA7,IL11,ZNF628,UBE2S,TMEM238,PPP6R1,SSC5D,TMEM86B,FAM71E2,U2AF2,MIR6805,MIR6802,SBK3,ZNF581,ISOC2,ZNF865,MIR6803,ZNF580,KMT5C,PTPRH,SBK2,RPL28,BRSK1	0	loss
3765	CGTE_46	19	58858071	59093745	19q13.43	CHMP2A,ZNF584,MIR4754,ZBTB45,ZNF324B,ZNF497,RPS5,UBE2M,ZNF446,ZNF132,MZF1-AS1,MIR6807,ZNF837,CENPBD1P1,MZF1,TRIM28,ZNF324,RNF225,A1BG,SLC27A5,A1BG-AS1	1	loss
3766	CGTE_46	20	21376484	23168707	20p11.22-p11.21	LINC01427,FOXA2,LINC01432,PAX1,LOC101929625,LINC01384,NKX2-4,THBD,LOC101929608,LINC00656,NKX2-2,LINC00261,CD93,SSTR4,LOC284788	0	loss

3767	CGTE_46	20	60587887	62905019	20q13.33	CHRNA4,PPDPF,TCEA2,ZGPAT,RPS21,TNFRSF6B,MIR124-3,OGFR,SLCO4A1,LINC00659,LOC100505771,ARFRP1,C20orf195,HAR1A,ZNF512B,MIR4758,MIR3196,PCMTD2,MIR941-3,PTK6,UCKL1-AS1,ARFGAP1,KCNQ2,ZBTB46-AS1,MIR6813,MIR647,LINC00029,RTEL1-TNFRSF6B,MIR941-4,SLC2A4RG,BHLHE23,RBBP8NL,SRMS,LKAAEAR1,YTHDF1,HRH3,SLCO4A1-AS1,SAMD10,LAMA5,GMEB2,DPH3P1,GID8,DNAJC5,MTG2,FLJ16779,LOC63930,OGFR-AS1,TAF4,MIR4326,HAR1B,EEF1A2,MIR3195,GATA5,LAMA5-AS1,SS18L1,NPBWR2,LINC01056,ADRM1,MIR941-2,UCKL1,MYT1,COL20A1,PRPF6,SLC17A9,OSBPL2,DIDO1,TPD52L2,MIR1-1,LIME1,RGS19,NTSR1,BIRC7,RTEL1,OPRL1,CABLES2,STMN3,C20orf166-AS1,TCFL5,ABHD16B,LSM14B,MIR941-5,MIR1914,ZBTB46,LINC00176,NKAIN4,MIR941-1,MRGBP,LOC100130587,MIR133A2,PSMA7,MIR1-1HG,COL9A3,HELZ2,SOX18	0	loss
3768	CGTE_46	21	9825720	33765785	21q22.11-p11.1	C21orf91,BAGE,C21orf91-OT1,LOC101927869,SCAF4,LINC00161,CHODL,SOD1,USP16,SAMSN1,LINC01549,GABPA,LIPI,MIR155,KRTAP19-5,KRTAP21-2,LINC00113,KRTAP20-1,KRTAP27-1,MIR8069-2,KRTAP7-1,CLDN17,MIR8069-1,BAGE4,KRTAP19-3,KRTAP13-2,ADAMTS1,CYP4F29P,MIR3156-3,KRTAP6-3,MIR4327,LINC00308,MIR155HG,LINC00317,GRIK1-AS1,KRTAP19-2,LINC00314,KRTAP15-1,MIR3687-2,ANKRD30BP2,MRAP,KRTAP19-7,KRTAP25-1,POTED,BACH1,CCT8,NCAM2,KRTAP20-2,USP25,LOC339622,KRTAP20-3,MIR548XHG,MAP3K7CL,LOC284825,SAMSN1-AS1,MIR99AHG,MIR125B2,NRIP1,CLDN8,LOC102724188,URB1,LINC00320,MIR3648-1,KRTAP13-4,MIR3118-1,KRTAP19-6,BTG3,MIRLET7C,GRIK1,RBM11,MIR4759,LOC101927973,HUNK,KRTAP23-1,KRTAP13-1,KRTAP22-2,RWDD2B,CXADR,ATP5J,CHODL-AS1,MIS18A,SNORA80A,ANKRD20A11P,HSPA13,JAM2,LOC388813,KRTAP6-1,BAGE3,MRPL39,KRTAP19-1,URB1-AS1,KRTAP11-1,KRTAP8-1,BAGE2,KRTAP24-1,KRTAP21-3,CYYR1,BAGE5,KRTAP20-4,TPTE,LINC00158,LTN1,BACH1-IT2,KRTAP21-1,LINC00189,TMPRSS15,MIR99A,LOC101927843,KRTAP6-2,TIAM1,LINC01425,LINC00515,N6AMT1,LINC00307,KRTAP26-1,KRTAP19-8,KRTAP13-3,KRTAP22-1,D21S2088E,TEKT4P2,GRIK1-AS2,ABCC13,MIR3687-1,APP,MIR5009,KRTAP19-4,ADAMTS5,LINC00159,MIR3648-2	3	gain
3769	CGTE_46	21	44185334	47331095	21q22.3	PWP2,C21orf2,LOC642852,TRPM2,FAM207A,AIRE,KRTAP10-2,SLC19A1,TRPM2-AS,AGPAT3,LOC106780825,TSPEAR-AS2,PKNOX1,MIR6070,SUMO3,ITGB2-AS1,KRTAP10-12,KRTAP10-4,KRTAP10-10,PDXK,LINC00163,ICOSLG,UBE2G2,RRP1,SSR4P1,KRTAP10-1,LOC100129027,LINC01424,LRRC3-AS1,CBS,COL18A1-AS1,WDR4,KRTAP10-5,TCONS_00029157,COL18A1-AS2,TSPEAR,HSF2BP,U2AF1,NDUFV3,RRP1B,SIK1,TRAPPC10,LOC102724652,KRTAP10-6,LINC00319,TSPEAR-AS1,PCBP3,PDE9A,POFUT2,LINC00322,DNMT3L,LRRC3,KRTAP12-4,C21orf33,FRGCA,KRTAP10-9,ITGB2,COL18A1,LINC00316,KRTAP12-3,AATBC,KRTAP12-1,KRTAP10-7,LINC01547,PTTG1IP,ADARB1,CRYAA,LINC00313,MIR6815,KRTAP10-3,KRTAP10-8,PFKL,KRTAP10-11,KRTAP12-2,ERVH48-1,CSTB,LINC00162	1	loss
3770	CGTE_46	21	47349727	47602866	21q22.3	COL6A2,FTCD,PCBP3,LOC101928796,COL6A1,SPATC1L	0	loss

3771	CGTE_46	22	37415852	38084971	22q12.3-q13.1	SH3BP1,LGALS2,CARD10,TMPRSS6,KCTD17,GGA1,C1QTNF6,TST,LGALS1,LOC100506271,SSTR3,MFNG,NOL12,MPST,LOC101927051,PDXP,CDC42EP1,IL2RB,RAC2,ELFN2,CYTH4	1	loss
3772	CGTE_46	22	50296739	51178618	22q13.33	ADM2,MIOX,HDAC10,TYMP,CHKB-CPT1B,SYCE3,SBF1,TRABD,PPP6R2,CPT1B,CRELD2,KLHDC7B,ODF3B,ACR,PIM3,SELO,NCAPH2,MIR6821,MAPK11,DENND6B,MLC1,CHKB-AS1,TUBGCP6,SHANK3,PANX2,ALG12,MAPK8IP2,IL17REL,MAPK12,MOV10L1,LMF2,SCO2,ARSA,PLXNB2,CHKB	0	loss
3773	CGTE_46	X	49137811	70282873	Xp11.23-Xq13.1	SNORA11D,MAGED1,FOXR2,GAGE2C,MIR502,XAGE5,SPIN4,USP51,FAM155B,RAB41,CCNB3,ASB12,HUWE1,STARD8,SPIN2B,GAGE12G,NUDT10,HSD17B10,OTUD6A,GDPD2,GAGE12E,SPANXN5,SNORA11E,GNL3L,SPIN2A,AWAT2,USP27X,HEPH,GAGE5,XAGE1B,GAGE8,YIPF6,FGD1,EDA,ZC3H12B,SPIN3,MAGEH1,MIR362,LINC01278,KANTR,MIR8088,GAGE4,MIR660,GAGE12H,TSPYL2,USP27X-AS1,XAGE1E,PAGE2,FRMD8P1,MIRLET7F2,MIR6894,LINC01284,MIR500A,EDA2R,TSR2,MAGED4,MIR6895,EFNB1,RIBC1,AR,PDZD11,XAGE2,GAGE2A,KIF4A,ITIH6,PAGE4,MIR223,PHF8,GAGE12D,APEX2,SHROOM4,UBQLN2,MIR4536-1,GAGE12I,KLF8,MSN,SSX2,MAGED4B,MIR501,RRAGB,UQCRBP1,CXorf67,ARR3,PFKFB1,AKAP4,ARHGEF9,IQSEC2,AMER1,FAM156B,SSX8,MIR500B,ALAS2,GAGE13,GAGE6,SNORA11,MTRNR2L10,GPR173,LAS1L,DLG3-AS1,GAGE2B,FAM104B,MIR676,ZXDB,MIR188,BMP15,XAGE3,NUDT11,ZXDA,IGBP1,SMC1A,GAGE12J,SSX2B,LOC100421746,SNX12,KDM5C,SSX7,AWAT1,SLC7A3,LINC01496,LINC00269,PAGE1,ARHGEF9-IT1,TEX11,FAM120C,PAGE2B,DGAT2L6,MAGED2,CENPVP1,GAGE10,GAGE12C,PJA1,GAGE12F,CLCN5,PAGE5,DGKK,GAGE12B,GAGE2D,MIR4536-2,ZC4H2,P2RY4,PPP1R3F,FAAH2,MIR98,PAGE3,LINC01420,CENPVP2,MTMR8,GAGE1,DLG3,GAGE2E,MIR6857,OPHN1,VSIG4,TRO,SNORA109,MIR1468,WNK3,MIR532,GAGE7,GSPT2,FAM156A	3	gain
3774	CGTE_46	X	70287960	70320476	Xq13.1	FOXO4,SNX12	56	gain

3775	CGTE_46	X	71351902	114419153	Xq21.33-Xq21.31	FGF16,KIAA2022,MIR448,NAP1L3,DMRTC1,NRK,TEX13A,PRPS1,XRCC6P5,MIR374C,MIR652,CHIC1,RAB40AL,TAF9B,PABPC1L2B,TMEM164,CHM,FAM46D,ARMCX5,HNRNPH2,TGIF2LX,CHRDL1,SLC16A2,PAK3,MIR361,PSMD10,ITM2A,SH3BGRL,PABPC1L2B-AS1,NXF4,TBX22,BEX2,CSTF2,TCP11X2,CPXCR1,ZCCHC16,MORF4L2,KCNE5,MIR421,MIR1912,TCEAL5,TCEAL3,ZCCHC5,NXF2B,RPL36A-HNRNPH2,ARMCX3,LINC00684,XACT,CDX4,AMMECR1,PGAM4,LOC101928358,IL13RA2,ARMCX6,FTX,TRPC5,HMGN5,IL1RAPL2,POU3F4,DIAPH2,PHKA1,MAGEE1,TEX13B,CXorf57,ARMCX4,MIR1298,NUP62CL,RAB9B,FAM226B,ATRX,DMRTC1B,NXT2,BEX4,PABPC1L2A,GLA,TTC3P1,PABPC5,TBC1D8B,CITED1,MIR4328,RPA4,PABPC5-AS1,SNORD96B,MIR4329,CYSLTR1,NXF3,MIR325HG,DACH2,PGK1,FLJ44635,RPS4X,APOOL,H2BFM,CHMP1B2P,RPL36A,TCEAL6,HDX,GLRA4,LPAR4,MIR6087,FAM199X,GPR174,POF1B,H2BFWT,NHSL2,TDGF1P3,ARL13A,BHLHB9,MIR764,LINC00890,UPRT,TMSB15A,LOC101928335,FAM133A,COL4A6,DIAPH2-AS1,ESX1,LOC100101478,NXF5,TSPAN6,MIR374B,TSIX,ALG13,MORC4,UBE2DNL,TMEM31,PIN4,LRCH2,TRMT2B,MAP2K4P1,RIPPLY1,KLHL4,NOX1,GUCY2F,CAPN6,H2BFXP,ARMCX1,MUM1L1,MIR545,TCEAL8,RPS6KA6,DRP2,SLC25A53,MIR384,ACSL4,WBP5,MIR325,NAP1L2,NXF2,ZMAT1,LHFPL1,ARMCX2,XKRX,PCDH11X,NGFRAP1,TRPC5OS,AMOT,LOC105373300,SYTL4,CENPI,LOC286437,CLDN2,RLIM,MIR1264,XIST,TCEAL1,TCEAL7,HTR2C,PIH1D3,ZDHHC15,IRS4,TAF7L,ABCB7,SRPX2,RNF128,PLP1,FRMPD3,SNORA35,RBM41,ZCCHC18,TCEAL4,GPRASP2,ZNF711,FAM226A,HDAC8,COL4A5,RAB40A,TIMM8A,LOC101928437,TCEAL2,VSIG1,SATL1,BEX5,LOC100132304,ATG4A,MIR1321,NAP1L6,MORF4L2-AS1,LINC00630,ZCCHC13,JPX,FRMPD3-AS1,GPRASP1,MAGT1,BTK,MID2,LOC101928259,MAGEE2,DCX,ERCC6L,TNMD,P2RY10,PBDC1,MIR374A,TSC22D3,MIR1911,BRWD3,MIR3978,LOC101928128,CYLC1,COX7B,TMSB15B,MIR4454,BRDTP1,PCDH19,ATP7A,TMEM35,ARMCX5-GPRASP2,SERPINA7,RGAG1,BEX1	3	gain
3776	CGTE_46	X	114422776	114468801	Xq23	RBMXL3,LRCH2	0	loss
3777	CGTE_46	X	152686435	153133370	Xq28	BGN,ABCD1,PDZD4,ATP2B3,DUSP9,BCAP31,HAUS7,PNCK,TREX2,SSR4,PLXNB3,SRPK3,SLC6A8,LOC105373383,ZFP92,FAM58A,IDH3G,L1CAM	0	loss
3778	CGTE_46	X	153133372	153149465	Xq28	LCA10,L1CAM	4	gain
3779	CGTE_46	X	153149468	153697856	Xq28	HCFC1-AS1,MIR3202-1,EMD,FAM50A,MIR718,MIR6858,LCA10,OPN1LW,DNASE1L1,IRAK1,CH17-340M24.3,TMEM187,TAZ,AVPR2,ARHGAP4,TKTL1,SNORA70,HCFC1,TEX28,MIR3202-2,OPN1MW,L1CAM,GDI1,ATP6AP1,NAA10,RENBP,FLNA,OPN1MW2,MECP2,RPL10,PLXNA3	1	loss
3780	CGTE_46	X	153698024	153698846	Xq28	PLXNA3	4	gain
3781	CGTE_47	1	861266	987203	1p36.33	ISG15,AGRN,NOC2L,PLEKHN1,KLHL17,HES4,PERM1,SAMD11	0	loss
3782	CGTE_47	1	987290	990505	1p36.33	AGRN	6	gain
3783	CGTE_47	1	1018186	1564479	1p36.33	VWA1,SDF4,ACAP3,TMEM240,TNFRSF18,MRPL20,MIR200B,AURKAIP1,TAS1R3,PUSL1,CPSF3L,MXRA8,DVL1,LOC148413,CPTP,MIR429,ANKRD65,ATAD3B,MIB2,MIR200A,ATAD3C,LINC01342,MIR6726,FAM132A,LOC102724312,MIR6808,TMEM88B,UBE2J2,SCNN1D,ATAD3A,TTLL10,SSU72,MIR6727,C1orf233,B3GALT6,C1orf159,TNFRSF4,CCNL2	1	loss
3784	CGTE_47	1	12081759	12089253	1p36.22	MIR6729,MIIP	8	gain
3785	CGTE_47	1	17248472	17294933	1p36.13	CROCC	0	loss
3786	CGTE_47	1	22174160	22178404	1p36.12	HSPG2	15	gain
3787	CGTE_47	1	22191596	22208012	1p36.12	HSPG2	4	gain

3788	CGTE_47	1	101361766	108293869	1p13.3-p21.1	AMY2B,COL11A1,SLC30A7,LINC01307,AMY1B,OLFM3,VAV3,RNPC3,LOC101928370,LOC102606465,S1PR1,DPH5,NTNG1,ACTG1P4,DNAJA1P5,LOC100129138,LOC101928476,PRMT6,LOC101928436,AMY1C,AMY2A,AMY1A	3	gain
3789	CGTE_47	1	109811255	109816453	1p13.3	CELSR2	5	gain
3790	CGTE_47	1	152636618	153122472	1q21.3	SPRR2B,SPRR1A,SPRR3,LCE2C,LCE1D,LCE2A,SMCP,SPRR2D,C1orf68,LCE1A,SPRR2E,SPRR2G,IVL,SPRR1B,LCE1C,SPRR2C,SPRR2F,LCE6A,LCE1B,SPRR2A,LCE1E,LCE2D,LCE4A,KPRP,LCE1F,SPRR4,LCE2B	3	gain
3791	CGTE_47	1	157490037	158908314	1q23.1	OR10Z1,OR6N1,OR6K3,CD1D,FCRL2,FCRL4,OR6K6,SPTA1,CD1E,OR10T2,CD5L,FCRL5,OR6P1,CD1B,MNDA,LOC646268,OR10K1,OR10R2,FCRL1,CD1A,OR6N2,OR6K2,FCRL3,OR10X1,OR6Y1,CD1C,PYHIN1,OR10K2,KIRREL	3	gain
3792	CGTE_47	1	185270003	200183405	1q32.1-q31.2	MIR4735,RGS13,MIR1278,PTGS2,UCHL5,OCLM,CDC73,RGS18,ATP6V1G3,GLRX2,CFHR5,HMCN1,LINC01222,MIR548F1,CFHR3,ASPM,RGS21,IVNS1ABP,KCNT2,F13B,LINC01350,DENND1B,NR5A2,MIR181A1,CFHR4,PDC,LHX9,CFH,MIR181A1HG,PRG4,NEK7,MIR4426,LINC01032,PACERR,RGS2,LINC01036,LINC01031,CFHR2,LOC440704,CRB1,LINC01037,PLA2G4A,C1orf53,GS1-279B7.1,PTPRC,RGS1,ZBTB41,TROVE2,LOC102724919,TPR,LINC01351,LINC01221,BRINP3,B3GALT2,CFHR1,MIR181B1,C1orf27	3	gain
3793	CGTE_47	2	136960101	149447927	2q22.2-q23.1	LOC101928386,LINC01412,MBD5,MIR7157,TEX41,GTDC1,HNMT,ACVR2A,ZEB2,ARHGAP15,ORC4,LRP1B,THSD7B,PABPC1P2,ZEB2-AS1,YY1P2,LOC101928273,NXPH2,EPC2,SPOPL,KYNU	3	gain
3794	CGTE_47	2	209189950	215674173	2q34-q35	CPS1,SPAG16,MIR548F2,LOC101928020,LOC100130451,CPS1-IT1,PTH2R,BARD1,MAP2,MIR4776-2,MIR4776-1,LOC102725079,KANSL1L,VWC2L-IT1,MIR4438,MYL1,VWC2L,LANCL1,PIKFYVE,RPE,LANCL1-AS1,ACADL,UNC80,IKZF2,ERBB4	3	gain
3795	CGTE_47	2	239006590	239056757	2q37.3	UBE2F-SCLY,ESPNL,KLHL30,SCLY	0	loss
3796	CGTE_47	3	75790414	89499505	3p11.1-p12.3	MIR3923,CADM2,ROBO2,GBE1,LOC101927374,C3orf38,VGLL3,EPHA3,CADM2-AS2,ZNF717,ROBO1,ZNF654,CHMP2B,MIR5688,HTR1F,SNORA95,MIR4795,POU1F1,CGGBP1,LOC728290,LINC00971,LINC00506	3	gain
3797	CGTE_47	3	187387861	187419851	3q27.3	RTP2,SST	9	gain
3798	CGTE_47	3	196669188	196675668	3q29	PIGZ,NCBP2-AS2,NCBP2	6	gain
3799	CGTE_47	4	2332567	2341325	4p16.3	ZFYVE28	5	gain
3800	CGTE_47	4	48597888	48605438	4p11	FRYL	6	gain
3801	CGTE_47	4	62383136	70361640	4q13.1-q13.2	UBA6,TMPRSS11A,EPHA5-AS1,ADGRL3-AS1,UGT2B7,TMPRSS11BNL,FTLP10,CENPC,ADGRL3,TECRL,STAP1,UBA6-AS1,EPHA5,UGT2A3,UGT2B15,UGT2B11,TMPRSS11B,LOC550113,YTHDC1,TMPRSS11E,LOC101927237,GNRHR,MIR1269A,TMPRSS11D,SYT14P1,UGT2B28,TMPRSS11F,TMPRSS11GP,LOC401134,UGT2B10,UGT2B17,UGT2B4	3	gain
3802	CGTE_47	4	190858343	190884383	4q35.2	FRG1	4	gain
3803	CGTE_47	6	26020713	26285778	6p22.2	HIST1H2BF,HIST1H4H,HIST1H4G,HIST1H1T,HIST1H3D,HIST1H2BI,HIST1H2BG,HIST1H4B,HIST1H2BH,HIST1H1D,HIST1H2BB,HIST1H3G,HFE,HIST1H3A,HIST1H4E,HIST1H3E,HIST1H2BE,HIST1H2BC,HIST1H2AD,HIST1H4D,HIST1H1E,HIST1H3C,HIST1H2AC,HIST1H4A,HIST1H4F,HIST1H4C,HIST1H2AE,HIST1H2AB,HIST1H3F,HIST1H3B,HIST1H1C,HIST1H2BD	3	gain
3804	CGTE_47	6	32017678	32035471	6p21.33	TNXB	5	gain

3805	CGTE_47	6	53409245	73952276	6q11.1-q12	TINAG,LGSN,PTP4A1,HMGCLL1,MIR30C2,SMAP1,GFRAL,MLIP,LOC101928307,SLC25A51P1,GCLC,KCNQ5-IT1,MIR4282,MIR30A,SDHAF4,LOC101930010,LMBRD1,LINC01626,FAM135A,LINC01564,LINC00472,MIR548U,COL19A1,ZNF451,LRRC1,LOC101928280,B3GAT2,KHDC1L,PHF3,HCRTR2,COL9A1,RAB23,KIAA1586,KHDRBS2,LOC102723883,BAG2,GUSBP4,COL21A1,LOC101927136,LOC441155,DST,BMP5,PRIM2,MTRNR2L9,LOC100506188,ADGRB3,KHDC1,KCNQ5,LINC00680-GUSBP4,EVADR,KLHL31,BEND6,OGFRL1,RIMS1,LINC00680,LOC101927211,KCNQ5-AS1,FAM83B,EYS,LOC101927189,MLIP-IT1	3	gain
3806	CGTE_47	6	82461282	82461928	6q14.1	FAM46A	9	gain
3807	CGTE_47	7	6977453	20782635	7p21.2-p21.3	LOC101927811,RPA3,TWISTNB,KCCAT333,AGR3,MACC1-AS1,GLCCI1,SCIN,LOC101927391,LOC101927354,BZW2,LOC100505938,C1GALT1,ETV1,AGR2,MEOX2,DGKB,UMAD1,COL28A1,MACC1,PRPS1L1,PHF14,ISPD,MIR3146,LOC101927630,LOC100131257,AGMO,LOC100505921,LOC101927769,LRRC72,VWDE,FERD3L,ABCB5,MIR3683,TMEM106B,NDUFA4,TSPAN13,ISPD-AS1,TMEM196,ITGB8,PER4,NXPH1,HDAC9,SNX13,SOSTDC1,MIOS,ANKMY2,LOC101927668,AHR,MEOX2-AS1,ICA1,THSD7A,TWIST1,ARL4A,MIR1302-6	3	gain
3808	CGTE_47	7	77754993	86811669	7q21.12-q21.11	GRM3,LOC100128317,KIAA1324L,CACNA2D1,CD36,HGF,MAGI2-AS2,SEMA3A,LOC101927269,LOC101927378,PCLO,GNAI1,MAGI2-AS3,GNAT3,DMTF1,LOC101927356,SEMA3D,RPL13AP17,SEMA3E,MAGI2,MIR548M,SEMA3C	3	gain
3809	CGTE_47	8	104454893	120809369	8q22.3-q24.11	KCNV1,LINC01609,EBAG9,TRHR,MIR3610,NOV,ZFPM2,DPYS,LINC01608,PKHD1L1,COLEC10,ENY2,EIF3E,TNFRSF11B,SYBU,EMC2,UTP23,RIMS2,LINC00536,SLC30A8,ABRA,MED30,EXT1,RAD21-AS1,ANGPT1,SAMD12-AS1,OXR1,CSMD3,LRP12,TMEM74,TAF2,RSPO2,RAD21,DCSTAMP,TRPS1,ZFPM2-AS1,MAL2,EIF3H,MIR2053,NUDCD1,DCAF13,ENPP2,SAMD12,AARD	3	gain
3810	CGTE_47	8	144460882	145138426	8q24.3	FAM83H-AS1,CCDC166,BREA2,MAFA-AS1,GRINA,FAM83H,MROH6,PUF60,EXOSC4,ZC3H3,GSDMD,NRBP2,RHPN1,EEF1D,MIR6846,MIR4664,ZNF707,SPATC1,MIR6845,EPPK1,ZNF623,PARP10,MAFA,SCRIB,MIR937,MIR6847,OPLAH,MIR661,PYCRL,MAPK15,GPAA1,NAPRT,TSTA3,PLEC,TIGD5	0	loss
3811	CGTE_47	9	21970872	22008749	9p21.3	CDKN2B,CDKN2B-AS1,CDKN2A	6	gain
3812	CGTE_47	9	138838123	140389610	9q34.3	RNF208,PNPLA7,SNORA17B,LCN6,TMEM203,TPRN,PTGDS,C9orf172,C9orf139,C9orf163,DNLZ,RABL6,CLIC3,LCN15,ENTPD8,CYSRT1,INPP5E,DPP7,FBXW5,MAMDC4,LCNL1,NRARP,MAN1B1-AS1,GPSM1,FUT7,MIR4479,LHX3,CCDC183,LRRC26,C9orf173-AS1,MIR4673,C9orf69,MIR3621,UBAC1,SNAPC4,NSMF,C8G,RNF224,NOXA1,CARD9,EDF1,EGFL7,MIR126,SNHG7,TRAF2,TMEM210,CCDC183-AS1,UAP1L1,AGPAT2,ABCA2,FAM69B,MAN1B1,ENTPD2,C9orf173,PMPCA,NOTCH1,FAM166A,MIR4674,LCN12,NPDC1,C9orf142,NELFB,LCN10,SNORA17A,MIR7114,EXD3,SLC34A3,MIR4292,LCN8,SSNA1,TMEM141,NALT1,NACC2,NDOR1,DKFZP434A062,MIR6722,SEC16A,SDCCAG3,SAPCD2,QSOX2,TUBB4B,ANAPC2,LOC100128593,GRIN1,TOR4A,PHPT1	1	loss
3813	CGTE_47	9	140391507	140395360	9q34.3	PNPLA7	6	gain
3814	CGTE_47	10	52486638	61028425	10q21.1-q11.23	PHYHIPL,MTRNR2L5,A1CF,LINC00844,DKK1,LOC102724719,CISD1,CCEPR,TFAM,BICC1,PRKG1,FAM13C,MBL2,CSTF2T,PCDH15,IPMK,MIR3924,UBE2D1,LINC01468,PRKG1-AS1,ASAH2B,ZWINT,FAM133CP,MIR605	3	gain
3815	CGTE_47	10	100167629	100177686	10q24.2	HPS1,PYROXD2	7	gain
3816	CGTE_47	10	134648057	134649786	10q26.3	CFAP46	7	gain

3817	CGTE_47	11	4411231	6238921	11p15.4	OR52N1,HBB,OR51I1,TRIM6,FAM160A2,MMP26,HBE1,OR52H1,OR51E2,OR52K1,OR51G1,OR52A1,UBQLN3,OR51M1,OR52R1,TRIM34,HBD,OR52D1,TRIM6-TRIM34,OR51A2,OR51A7,OR56B4,OR52E8,OR52B6,HBG1,OR52E2,OR51Q1,OR52K2,OR56A3,OR52I1,TRIM68,OR51T1,OR52E6,OR51B2,BGLT3,OR52N4,C11orf42,OR56B1,OR52W1,OR51F2,OR51G2,UBQLNL,HBG2,TRIM22,OR56A5,HBBP1,OR56A4,OR51B6,OR51B4,OR51E1,OR52J3,OR51S1,OR56A1,OR52N2,OR52L1,OR51A4,OR52B2,OR51V1,OR51L1,OR52N5,C11orf40,OR52A5,OR52E4,OR51I2,OR51B5,OR52M1,OLFM5P,TRIM21,OR51D1,OR51F1,TRIM5,OR52I2	3	gain
3818	CGTE_47	12	72148955	93251335	12q21.32-q21.1	MIR4699,BTG1,OSBPL8,LINC00615,MIR1252,KCNC2,CAPS2,TRHDE,C12orf29,PPFIA2,LINC01490,PHLDA1,MGAT4C,PLEKHG7,CCER1,KRR1,TBC1D15,LOC100507377,PPP1R12A,MRS2P2,MIR5692B,GALNT4,NTS,CCDC59,RASSF9,ALX1,POC1B,LINC00936,E2F7,EEA1,NAP1L1,ACSS3,POC1B-GALNT4,TSPAN19,C12orf50,TRHDE-AS1,MKRN9P,ZDHHC17,OTOGL,METTL25,CLLU1,ATP2B1,RAB21,SLC6A15,TMTC3,MYF6,LOC101928137,MIR548AL,LIN7A,PAWR,DCN,LINC01619,TMTC2,KERA,CEP290,PTPRQ,C12orf74,MYF5,GLIPR1L1,TPH2,LOC728084,EPYC,LOC102724663,ATXN7L3B,GLIPR1L2,CLLU1OS,MIR617,BBS10,MIR618,NAV3,LOC101928449,DUSP6,LUM,CSRP2,LRRIQ1,SYT1,KITLG,GLIPR1	3	gain
3819	CGTE_47	12	125434516	125441781	12q24.31	DHX37	13	gain
3820	CGTE_47	13	28494270	28498550	13q12.2	PDX1-AS1,PDX1	73	gain
3821	CGTE_47	13	37605623	38924314	13q13.3	LINC01048,POSTN,UFM1,LINC00571,SUPT20H,LINC00547,TRPC4,CSNK1A1L	3	gain
3822	CGTE_47	13	96743122	96743893	13q32.1	HS6ST3	10	gain
3823	CGTE_47	14	51560769	51710610	14q22.1	TMX1,TRIM9	55	gain
3824	CGTE_47	14	104563765	105196547	14q32.33	INF2,MIR203A,MIR4710,MIR203B,C14orf180,KIF26A,ADSSL1,ASPG,TMEM179	0	loss
3825	CGTE_47	14	105609769	105622291	14q32.33	JAG2,MIR6765	0	loss
3826	CGTE_47	15	60734701	62333636	15q22.2	ICE2,RORA-AS2,VPS13C,RORA,RORA-AS1	3	gain
3827	CGTE_47	15	62336337	62360851	15q22.2	C2CD4A,VPS13C	0	loss
3828	CGTE_47	16	88520007	88552561	16q24.2	MIR5189,ZFPM1	8	gain
3829	CGTE_47	16	88555355	88968161	16q24.3-q24.2	CYBA,MVD,CTU2,LOC100289580,MIR4722,PABPN1L,LOC339059,GALNS,TRAPPC2L,ZC3H18,SNAI3-AS1,CDT1,IL17C,PIEZO1,ZFPM1,RNF166,SNAI3,APRT,CBFA2T3	1	loss
3830	CGTE_47	17	38859512	39619354	17q21.2	KRTAP4-6,KRT40,KRTAP2-2,KRT27,KRTAP9-3,KRTAP4-11,KRTAP2-1,KRT33B,KRTAP1-1,KRTAP9-1,KRTAP9-4,KRTAP4-9,KRT20,KRTAP4-1,KRT37,TMEM99,LOC100505782,KRT10,KRT34,KRTAP9-6,KRT33A,KRTAP29-1,KRTAP4-8,KRT28,KRT32,KRTAP9-7,KRTAP1-4,KRT39,KRTAP4-7,KRT24,KRT12,KRTAP2-4,KRTAP9-8,KRTAP9-2,KRTAP3-2,KRTAP1-5,KRTAP9-9,KRTAP17-1,KRTAP4-12,KRT23,KRTAP16-1,KRTAP4-3,KRT25,KRT38,KRTAP4-4,KRTAP3-3,KRT26,KRTAP4-5,KRT31,KRTAP1-3,KRTAP3-1,KRTAP2-3,KRTAP4-2	3	gain
3831	CGTE_47	17	73615971	73622301	17q25.1	MYO15B	7	gain
3832	CGTE_47	18	8783411	8825974	18p11.22	MTCL1	3	gain
3833	CGTE_47	19	422156	579448	19p13.3	SHC2,GZMM,ODF3L2,MADCAM1,TPGS1,BSG,CDC34	0	loss
3834	CGTE_47	19	579449	582400	19p13.3	BSG	6	gain

3835	CGTE_47	19	582456	1987713	19p13.3	PLPPR3,R3HDM4,ADAT3,ONECUT3,EFNA2,KISS1R,FGF22,MEX3D,CFD,APC2,KLF16,SCAMP4,BTBD2,BSG,RPS15,PRTN3,CIRBP-AS1,CIRBP,ATP5D,PTBP1,AZU1,MUM1,PCSK4,C19orf25,PRSS57,MED16,C19orf24,POLR2E,PLK5,TCF3,HMHA1,ABCA7,MIR3187,TMEM259,MIR4745,MBD3,ADAMTSL5,STK11,HCN2,CSNK1G2-AS1,ELANE,CNN2,POLRMT,MIR1909,CSNK1G2,RNU6-2,WDR18,RNF126,SBNO2,MISP,GPX4,CBARP,REEP6,GAMT,UQCR11,LOC100288123,ARID3A,MIDN,PALM,NDUFS7,ABHD17A,DAZAP1,ATP8B3,REXO1,GRIN3B,FSTL3	1	loss
3836	CGTE_47	19	1989994	1993222	19p13.3	BTBD2	7	gain
3837	CGTE_47	19	15739262	16301692	19p13.11-p13.12	HSH2D,CYP4F2,TPM4,RAB8A,LOC102724279,CIB3,OR10H5,OR10H2,FAM32A,LINC00905,CYP4F8,OR10H1,CYP4F11,OR10H4,UCA1,OR10H3,CYP4F24P,LINC00661,CYP4F12,CYP4F3	3	gain
3838	CGTE_47	19	23938117	24115211	19p12	RPSAP58,ZNF726,ZNF681	10	gain
3839	CGTE_47	20	60884380	60895882	20q13.33	LAMA5	0	loss
3840	CGTE_47	20	60895887	60902062	20q13.33	LAMA5	4	gain
3841	CGTE_47	20	60902187	60911445	20q13.33	MIR4758,LAMA5	0	loss
3842	CGTE_47	20	60911450	60912710	20q13.33	LAMA5	6	gain
3843	CGTE_47	20	61926366	61941419	20q13.33	COL20A1	7	gain
3844	CGTE_47	20	61941511	61987836	20q13.33	CHRNA4,COL20A1	0	loss
3845	CGTE_47	20	61990840	62127466	20q13.33	KCNQ2,EEF1A2,LOC100130587,CHRNA4	3	gain
3846	CGTE_47	20	62128921	62222147	20q13.33	SRMS,PTK6,C20orf195,EEF1A2,GMEB2,HELZ2,PPDPF	0	loss
3847	CGTE_47	21	31311664	32253929	21q21.3-q22.11	KRTAP6-1,KRTAP22-2,CLDN8,KRTAP6-2,KRTAP20-3,KRTAP11-1,KRTAP13-3,KRTAP8-1,KRTAP15-1,KRTAP25-1,KRTAP19-6,KRTAP19-5,MIR4327,KRTAP21-3,KRTAP20-2,KRTAP21-1,KRTAP19-2,LINC00307,KRTAP19-3,KRTAP19-1,KRTAP27-1,KRTAP24-1,KRTAP13-4,KRTAP6-3,KRTAP20-4,KRTAP20-1,KRTAP26-1,CLDN17,KRTAP21-2,KRTAP22-1,KRTAP13-2,KRTAP23-1,KRTAP13-1,GRIK1,KRTAP19-4,KRTAP19-7,KRTAP7-1	3	gain
3848	CGTE_47	21	45959977	45978279	21q22.3	KRTAP10-2,KRTAP10-3,KRTAP10-1,TSPEAR	10	gain
3849	CGTE_47	22	23656744	23917406	22q11.23	BCR,CES5AP1,IGLL1,ZDHHC8P1,LOC388882,LOC101929374	9	gain
3850	CGTE_47	X	24806500	24828984	Xp22.11	POLA1	20	gain
3851	CGTE_47	X	37027879	37028924	Xp21.1	FAM47C	82	gain
3852	CGTE_47	X	72667067	72804436	Xq13.2	MAP2K4P1,CHIC1,CDX4	4	gain
3853	CGTE_48	1	1146913	1149611	1p36.33	TNFRSF4	9	gain
3854	CGTE_48	1	152484018	153122472	1q21.3	SPRR4,SPRR2F,LCE1B,IVL,SPRR2C,LCE1E,LCE1F,CRCT1,SPRR3,SPRR1B,LCE6A,KPRP,LCE1A,LCE3E,LCE2B,LCE1C,LCE4A,C1orf68,LCE5A,LCE3A,LCE2D,SPRR2A,SPRR2E,SMCP,SPRR1A,SPRR2G,LCE3D,LCE3C,SPRR2B,LCE3B,LCE2C,SPRR2D,LCE2A,LCE1D	3	gain
3855	CGTE_48	1	185270003	199998383	1q32.1-q25.3	RGS1,TPR,RGS13,CFHR2,CRB1,B3GALT2,CFHR4,TROVE2,NEK7,DENND1B,LINC01221,PTGS2,OCLM,RGS21,PRG4,MIR181A1HG,C1orf27,RGS2,C1orf53,KCNT2,MIR4426,NR5A2,LINC01222,LINC01036,LHX9,LINC01037,GLRX2,PTPRC,PACERR,LINC01032,LINC01351,CFHR5,RGS18,ATP6V1G3,ZBTB41,LINC01031,MIR1278,PLA2G4A,HMCN1,CDC73,MIR4735,CFHR1,LINC01350,F13B,GS1-279B7.1,IVNS1ABP,LOC102724919,CFHR3,LOC440704,PDC,BRINP3,UCHL5,CFH,MIR181A1,ASPM,MIR548F1,MIR181B1	3	gain
3856	CGTE_48	2	136718886	149247707	2q23.1-q21.3	KYNU,SPOPL,LOC101928386,MBD5,ACVR2A,NXPH2,DARS,HNMT,YY1P2,ARHGAP15,CXCR4,MIR7157,ZEB2-AS1,GTDC1,LRP1B,LINC01412,ZEB2,PABPC1P2,DARS-AS1,TEX41,THSD7B,LOC101928273,ORC4	3	gain

3857	CGTE_48	2	209212534	215876820	2q35-q34	UNC80,LANCL1,MAP2,VWC2L-IT1,PTH2R,VWC2L,CPS1-IT1,LANCL1-AS1,MYL1,BARD1,MIR4776-2,RPE,ABCA12,KANSL1L,MIR4776-1,MIR548F2,SPAG16,IKZF2,LOC101928020,ERBB4,ACADL,LOC100130451,MIR4438,LOC102725079,PIKFYVE,LOC101928103,CPS1	3	gain
3858	CGTE_48	3	138724259	138762807	3q23	PRR23B,PRR23C,PRR23A	4	gain
3859	CGTE_48	3	161221006	165555309	3q26.1	LINC01324,OTOL1,SI,LINC01322,BCHE,MIR1263,SLITRK3,LINC01192	3	gain
3860	CGTE_48	3	180707157	182166175	3q26.33	SOX2-OT,LOC102724604,FLJ46066,LINC01206,SOX2,RNU6-2,DNAJC19	4	gain
3861	CGTE_48	3	186978491	192517485	3q29-q28	SNAR-I,MASP1,IL1RAP,TPRG1-AS1,CLDN16,LPP-AS1,P3H2,BCL6,UTS2B,TPRG1,OSTN-AS1,RTP4,GMNC,LPP,LOC100131635,MIR944,SST,TPRG1-AS2,MIR28,P3H2-AS1,MB21D2,LINCR-0002,FGF12,PYDC2,OSTN,TMEM207,FLJ42393,LPP-AS2,FGF12-AS1,TP63,RTP2,CLDN1,CCDC50	3	gain
3862	CGTE_48	4	62383011	67142606	4q13.1-q13.2	LOC401134,TECRL,MIR1269A,ADGRL3-AS1,EPHA5-AS1,EPHA5,ADGRL3	3	gain
3863	CGTE_48	5	16877663	31323431	5p14.1-p14.2	PMCHL1,LINC01021,BASP1,LOC340107,CDH10,LOC101929681,LOC401177,MYO10,SNORA105A,LSP1P3,CDH9,CDH18,LOC101929645,CDH6,LOC101929660,PRDM9,LOC285696,SNORA105B,GUSBP1,CDH12,C5orf17	3	gain
3864	CGTE_48	5	38944331	52097709	5p12-q11.2	TTC33,PLCXD3,CARD6,MROH2B,NNT-AS1,HMGCS1,LOC100132356,C7,C6,FYB,LOC100287592,ANXA2R,PELO,NIM1K,C5orf28,C5orf51,OXCT1-AS1,ZNF131,LOC101926940,EMB,HCN1,CCDC152,PTGER4,MRPS30,GHR,PAIP1,PARP8,CCL28,FBXO4,SEPP1,BRCAT54,LOC153684,LOC100506639,LOC642366,FGF10,C9,RICTOR,PRKAA1,LOC100506548,ITGA1,LOC101926960,SNORD72,RPL37,LINC00603,FGF10-AS1,FLJ32255,OXCT1,ISL1,DAB2,BRCAT107,LOC648987,NNT,C5orf34	3	gain
3865	CGTE_48	5	140182023	140263947	5q31.3	PCDHA2,PCDHA1,PCDHA7,PCDHA10,PCDHA5,PCDHA11,PCDHA6,PCDHA8,PCDHA3,PCDHA4,PCDHA12,PCDHA9,PCDHA13	3	gain
3866	CGTE_48	6	26017701	26285778	6p22.2	HIST1H2AD,HIST1H4C,HIST1H4H,HIST1H4E,HIST1H2BB,HIST1H2BG,HIST1H3G,HIST1H4A,HIST1H3B,HIST1H3E,HIST1H2BD,HIST1H3F,HIST1H4F,HIST1H1C,HIST1H2BF,HIST1H1D,HIST1H2BE,HIST1H4G,HIST1H4D,HIST1H3C,HIST1H2BC,HFE,HIST1H2BI,HIST1H2AC,HIST1H3A,HIST1H2BH,HIST1H1E,HIST1H1T,HIST1H3D,HIST1H4B,HIST1H1A,HIST1H2AE,HIST1H2AB	3	gain
3867	CGTE_48	6	31324110	31371612	6p21.33	HLA-B,MICA	4	gain
3868	CGTE_48	6	32338253	32632794	6p21.32	HLA-DQB1,BTNL2,HLA-DQA1,HLA-DRB6,HLA-DRB1,HLA-DRB5,HLA-DRA,C6orf10,HCG23	3	gain
3869	CGTE_48	6	102074163	102516484	6q16.3	GRIK2	3	gain
3870	CGTE_48	6	127775016	129723698	6q22.33	THEMIS,C6orf58,SOGA3,LOC101928140,KIAA0408,PTPRK,LAMA2	3	gain
3871	CGTE_48	7	10979522	19813386	7p21.1-p21.3	KCCAT333,SCIN,ETV1,TWISTNB,BZW2,AHR,LOC101927630,TMEM196,MIR3146,TMEM106B,HDAC9,THSD7A,DGKB,PRPS1L1,LRRC72,SOSTDC1,MEOX2,ISPD-AS1,NDUFA4,AGMO,MIR1302-6,FERD3L,ARL4A,MEOX2-AS1,AGR2,VWDE,AGR3,ANKMY2,PHF14,SNX13,TSPAN13,TWIST1,ISPD	3	gain
3872	CGTE_48	7	77648494	86794466	7q21.11-q21.12	PCLO,MAGI2-AS2,KIAA1324L,SEMA3A,LOC101927356,LOC101927378,HGF,LOC101927269,MIR548M,DMTF1,SEMA3D,GNAI1,GNAT3,SEMA3E,MAGI2,CD36,RPL13AP17,CACNA2D1,LOC100128317,SEMA3C,MAGI2-AS3,GRM3	3	gain
3873	CGTE_48	8	2806801	4852016	8p23.2	CSMD1	3	gain
3874	CGTE_48	8	49987035	52233583	8q11.21-q11.22	SNTG1,C8orf22,PXDNL	3	gain
3875	CGTE_48	8	90996557	94179120	8q22.1-q21.3	NBN,TMEM55A,DECR1,LINC01030,TMEM64,LOC102724710,CALB1,NECAB1,MIR7641-2,LINC00534,OTUD6B-AS1,TRIQK,C8orf88,MIR8084,OTUD6B,SLC26A7,MIR4661,LRRC69,C8orf87,RUNX1T1,FLJ46284	3	gain

3876	CGTE_48	8	104336695	117658993	8q23.2-q23.1	MIR2053,RSPO2,DCSTAMP,ZFPM2-AS1,EBAG9,LRP12,SLC25A32,EIF3H,ANGPT1,ZFPM2,TMEM74,NUDCD1,ENY2,LINC01609,PKHD1L1,TRHR,OXR1,SYBU,ABRA,DCAF13,LINC01608,KCNV1,DPYS,CSMD3,RIMS2,EMC2,TRPS1,FZD6,EIF3E,CTHRC1,LINC00536	3	gain
3877	CGTE_48	8	125131842	125384287	8q24.13	FER1L6,LOC101927588,FER1L6-AS2,TMEM65	4	gain
3878	CGTE_48	8	144662852	144668620	8q24.3	EEF1D	5	gain
3879	CGTE_48	8	145001406	145004577	8q24.3	PLEC	7	gain
3880	CGTE_48	8	145638645	145641680	8q24.3	SLC39A4	3	gain
3881	CGTE_48	8	145772472	145773883	8q24.3	ARHGAP39	8	gain
3882	CGTE_48	9	8499700	14179797	9p24.1-p23	MPDZ,LINC00583,LURAP1L-AS1,PTPRD-AS1,TYRP1,NFIB,PTPRD,SNORD137,PTPRD-AS2,FLJ41200,LURAP1L	3	gain
3883	CGTE_48	9	33473770	33676497	9p13.3	ANXA2P2,ANKRD18B,SUGT1P1,NOL6,PTENP1	3	gain
3884	CGTE_48	9	103348520	107535313	9q31.1	GRIN3A,CYLC2,ALDOB,OR13C8,NIPSNAP3A,PLPPR1,OR13C3,TMEM246,ZNF189,RNF20,LINC01492,TMEM246-AS1,PPP3R2,BAAT,OR13C9,MURC,OR13D1,LOC101928523,OR13C2,OR13F1,OR13C5,SMC2,OR13C4,LINC00587,NIPSNAP3B,MRPL50,SMC2-AS1	3	gain
3885	CGTE_48	9	115818743	115913515	9q32	FAM225A,ZFP37,SLC31A2,FAM225B	5	gain
3886	CGTE_48	9	140167176	140174478	9q34.3	TOR4A,NELFB	69	gain
3887	CGTE_48	10	27702744	27793808	10p12.1	PTCHD3,RAB18	5	gain
3888	CGTE_48	10	35896546	38065637	10p11.1-p11.21	GJD4,MTRNR2L7,MIR4683,PCAT5,FZD8,ZNF248,LINC00993,ANKRD30A	3	gain
3889	CGTE_48	10	52103256	60145411	10q11.23-q21.1	PRKG1-AS1,SGMS1,PRKG1,LINC01468,SGMS1-AS1,MBL2,LOC102724719,MTRNR2L5,UBE2D1,DKK1,TFAM,MIR605,ASAH2B,PCDH15,IPMK,MIR3924,CSTF2T,A1CF,ZWINT,CISD1	3	gain
3890	CGTE_48	10	105991630	106015089	10q25.1	CFAP43,GSTO1	5	gain
3891	CGTE_48	10	124591795	124768667	10q26.13	FAM24A,IKZF5,FAM24B-CUZD1,C10orf88,CUZD1,FAM24B,PSTK,ACADSB,LOC399815	3	gain
3892	CGTE_48	11	4411231	6240316	11p15.4	OR52B2,UBQLN3,TRIM34,OR52D1,OR52N1,OR51E1,OR51D1,OR56A3,TRIM5,OR52I1,TRIM6-TRIM34,OR51B6,HBB,OR52M1,OR52E4,OR52A1,OR56B1,OR52L1,OR52R1,HBG2,OR51A4,OR51M1,OR52K2,TRIM6,OR51B2,OR51T1,OR51S1,OR52B6,OR51I2,OR52K1,OR51V1,OR51B5,HBG1,OR51A2,OR56A1,FAM160A2,OR51F2,OR52N4,HBBP1,OR56B4,OR52W1,C11orf40,TRIM22,BGLT3,OR52E8,UBQLNL,OR51Q1,OR51B4,OR52N5,OR52H1,TRIM21,HBD,OR51I1,OR56A5,OR52I2,OLFM5P,OR52E2,MMP26,OR51G1,OR51G2,OR51A7,OR51L1,OR52E6,OR56A4,OR51F1,HBE1,OR51E2,OR52J3,OR52A5,OR52N2,C11orf42,TRIM68	3	gain
3893	CGTE_48	11	48181399	57004521	11p11.12-q12.1	OR5T1,OR5D16,OR5M1,OR4B1,OR4S2,OR10AG1,OR4A47,OR8K3,OR5M11,OR4X2,TRIM48,OR5L1,OR4S1,TRIM64C,OR5T3,OR5AK4P,OR5F1,OR4X1,OR8J3,OR4C6,OR4C16,OR5AP2,PTPRJ,OR5D13,OR9G1,OR9G9,OR9G4,MIR6128,OR5AS1,TRIM51HP,OR4P4,OR5W2,OR4C12,OR4C3,OR5T2,TRIM51,OR8K5,OR8J1,OR5L2,FOLH1,OR4A15,OR5AK2,LOC101927120,APLNR,OR4C15,LOC646813,OR4A5,OR7E5P,OR5M9,OR5M10,OR5D18,OR5R1,OR8H2,OR8H1,OR8U1,OR8U8,OR4C45,OR8K1,OR8H3,OR5I1,OR4A16,OR5D14,OR5J2,OR8I2,OR5AR1,LRRC55,OR4C46,OR5M8,OR4C11,TRIM49B,OR4C13,OR5M3,LOC440040,LOC441601	3	gain
3894	CGTE_48	11	67056906	68855424	11q13.3-q13.2	CABP2,MIR6752,KMT5B,NUDT8,CDK2AP2,TMEM134,FAM86C2P,MIR3164,NDUFV1,CLCF1,MIR4691,RPS6KB2,MRPL21,CORO1B,NDUFS8,LOC100130987,RAD9A,MTL5,CPT1A,MIR6753,GSTP1,ANKRD13D,TPCN2,TBC1D10C,MRGPRF-AS1,CABP4,POLD4,MRGPRD,ALDH3B1,CHKA,PPP6R3,MRGPRF,C11orf24,C11orf72,IGHMBP2,ACY3,GAL,GPR152,TCIRG1,MIR7113,PITPNM1,LRP5,SSH3,PPP1CA,UNC93B1,ALDH3B2,CARNS1,PTPRCAP,AIP,TBX10,DOC2GP	3	gain

3895	CGTE_48	11	73785263	75014723	11q13.4	ARRB1,OR2AT4,SPCS2,TPBGL,POLD3,XRRA1,P4HA3,SLCO2B1,PGM2L1,LIPT2,LOC101928580,CHRDL2,PPME1,KCNE3,RNF169,NEU3,MIR4696,C2CD3	3	gain
3896	CGTE_48	11	77047126	85431995	11q13.5-q14.1	DLG2,NARS2,CCDC89,AQP11,TMEM126A,AAMDC,SYTL2,PAK1,CLNS1A,RSF1,THRSP,LOC101928896,CREBZF,MIR4300,USP35,GAB2,MIR4300HG,KCTD14,LOC101928944,SNORA70E,KCTD21,ANKRD42,NDUFC2-KCTD14,PRCP,TMEM126B,NDUFC2,MIR5579,LOC646029,KCTD21-AS1,INTS4,RAB30-AS1,RAB30,ALG8,LOC101928865,TENM4,MIR708,DDIAS,CCDC90B,FAM181B,PCF11	3	gain
3897	CGTE_48	11	94245587	106810853	11q22.3-q21	CNTN5,BIRC3,JRKL-AS1,CEP126,ARHGAP42,CARD16,MMP3,ANGPTL5,PDGFD,ENDOD1,YAP1,PIWIL4,MIR7641-1,MMP10,MTMR2,MMP12,LOC100129203,CASP12,LOC643733,SRSF8,LOC102723895,PGR,GUCY1A2,CASP1P2,CASP5,AMOTL1,LOC101054525,MMP8,TMEM133,FAM76B,DYNC2H1,KBTBD3,JRKL,KDM4D,MMP27,BIRC2,TMEM123,MSANTD4,KDM4E,MMP20,CWC15,SESN3,MMP1,LOC101928535,DCUN1D5,MMP7,DDI1,MIR3920,LOC100128386,TRPC6,CASP1,WTAPP1,LOC101929295,MMP13,CEP57,C11orf70,MIR4693,AASDHPPT,MIR1260B,CARD18,CCDC82,C11orf97,FUT4,CARD17,MAML2,GRIA4,CASP4	3	gain
3898	CGTE_48	13	35743024	35745581	13q13.3	NBEA	6	gain
3899	CGTE_48	13	37614540	40293963	13q13.3-q14.11	UFM1,LINC00547,CSNK1A1L,POSTN,NHLRC3,STOML3,LINC00437,COG6,TRPC4,LINC00571,LINC01048,SUPT20H,LHFP,LINC00366,FREM2,MIR4305,PROSER1	3	gain
3900	CGTE_48	14	20201784	20767090	14q11.2	OR4K15,OR4K1,OR4L1,OR4M1,OR4Q3,OR4K17,OR4K2,TTC5,OR4N5,OR4K5,OR4N2,OR11H6,OR11H4,OR11G2,OR4K13,OR4K14	3	gain
3901	CGTE_48	14	21971280	22928229	14q11.2	OR10G2,OR4E2,SALL2,METTL3,OR4E1,OR10G3	3	gain
3902	CGTE_48	14	24779761	24785983	14q12	LTB4R2,CIDEB,LTB4R	3	gain
3903	CGTE_48	14	50154724	50160086	14q21.3	KLHDC1,POLE2	43	gain
3904	CGTE_48	16	2286845	2288033	16p13.3	DNASE1L2	17	gain
3905	CGTE_48	16	31439038	31471349	16p11.2	ARMC5,ZNF843,COX6A2	3	gain
3906	CGTE_48	16	55542823	55563878	16q12.2	LPCAT2	4	gain
3907	CGTE_48	16	59785799	65038867	16q21	CDH8,LOC101927580,LOC729159,MIR4426,APOOP5,CDH11	3	gain
3908	CGTE_48	17	39140011	39432574	17q21.2	KRTAP9-8,KRTAP2-2,KRTAP9-3,KRTAP9-2,KRT40,KRTAP3-3,KRTAP4-11,KRTAP1-1,KRTAP4-12,KRTAP4-6,KRTAP1-4,KRTAP4-1,KRTAP9-4,KRTAP3-1,KRTAP9-7,KRTAP9-9,KRTAP4-9,KRTAP4-3,KRTAP4-8,KRTAP9-6,KRTAP4-7,KRTAP4-2,KRTAP9-1,KRTAP3-2,KRTAP2-3,KRTAP2-4,KRTAP1-5,KRTAP2-1,KRTAP4-4,KRTAP1-3,KRTAP4-5	3	gain
3909	CGTE_48	21	31311664	32410703	21q21.3-q22.11	CLDN8,KRTAP19-4,KRTAP19-7,KRTAP15-1,KRTAP20-1,KRTAP13-2,MIR4327,KRTAP6-1,CLDN17,KRTAP23-1,KRTAP22-1,KRTAP20-2,KRTAP6-3,KRTAP8-1,KRTAP20-3,KRTAP19-5,KRTAP13-1,KRTAP19-6,KRTAP13-4,KRTAP20-4,KRTAP21-2,KRTAP11-1,KRTAP25-1,KRTAP21-1,KRTAP6-2,KRTAP19-8,LINC00307,KRTAP26-1,GRIK1,KRTAP13-3,KRTAP19-1,KRTAP21-3,KRTAP22-2,KRTAP19-3,KRTAP24-1,KRTAP27-1,KRTAP19-2,KRTAP7-1	3	gain
3910	CGTE_48	21	45970507	46000487	21q22.3	KRTAP10-3,KRTAP10-2,TSPEAR,KRTAP10-4,KRTAP10-5	3	gain
3911	CGTE_48	22	21983160	21984303	22q11.21	YDJC	6	gain
3912	CGTE_49	1	70291387	70304896	1p31.1	LRRC7	6	gain
3913	CGTE_49	1	74574927	74621554	1p31.1	LRRIQ3	17	gain
3914	CGTE_49	1	150681375	150684368	1q21.3	HORMAD1	6	gain
3915	CGTE_49	1	173495704	173505168	1q25.1	SLC9C2	7	gain
3916	CGTE_49	3	5212065	5215732	3p26.1	ARL8B	0	loss

3917	CGTE_49	3	164712006	164793800	3q26.1	SI	3	gain
3918	CGTE_49	4	949946	1020758	4p16.3	SLC26A1,DGKQ,FGFRL1,IDUA,TMEM175	1	loss
3919	CGTE_49	4	3429947	3769730	4p16.3	RGS12,LINC00955,DOK7,HGFAC,ADRA2C,LRPAP1,LOC100133461	1	loss
3920	CGTE_49	4	48835388	48844764	4p11	OCIAD1	5	gain
3921	CGTE_49	4	68496079	68497694	4q13.2	UBA6	5	gain
3922	CGTE_49	4	146042619	146044607	4q31.21	ABCE1	8	gain
3923	CGTE_49	5	72147148	72157818	5q13.2	TNPO1	3	gain
3924	CGTE_49	6	29759900	29911267	6p22.1	HCG4B,HCG4,LOC554223,HLA-G,HLA-A,HLA-H	1	loss
3925	CGTE_49	7	121548731	121568327	7q31.32	PTPRZ1	5	gain
3926	CGTE_49	10	35896546	35930313	10p11.21	MIR4683,FZD8,GJD4	1	loss
3927	CGTE_49	10	118100217	118138976	10q25.3	CCDC172	3	gain
3928	CGTE_49	10	120070344	120085687	10q26.11	FAM204A	6	gain
3929	CGTE_49	12	40874156	40888021	12q12	MUC19	1	loss
3930	CGTE_49	13	38934079	39262135	13q13.3	LINC00437,UFM1,FREM2,LINC00366	1	loss
3931	CGTE_49	13	60737611	61018931	13q21.2	DIAPH3,TDRD3,LINC00434	1	loss
3932	CGTE_49	13	100622394	100741487	13q32.3	LINC00554,PCCA,LOC105370333,ZIC2,ZIC5	1	loss
3933	CGTE_49	14	57699362	57711142	14q22.3	EXOC5	3	gain
3934	CGTE_49	14	103987257	103988824	14q32.32	CKB	0	loss
3935	CGTE_49	16	596835	615348	16p13.3	PRR35,CAPN15	0	loss
3936	CGTE_49	18	9119347	9125066	18p11.22	NDUFV2,NDUFV2-AS1	1	loss
3937	CGTE_49	18	34854095	35065613	18q12.2	SNORA111,CELF4	1	loss
3938	CGTE_49	19	2247548	2253748	19p13.3	SF3A2,JSRP1,MIR4321,AMH	0	loss
3939	CGTE_49	19	45565494	45720981	19q13.32	GEMIN7,ZNF296,BLOC1S3,CLASRP,EXOC3L2,NKPD1,TRAPPC6A,PPP1R37	1	loss
3940	CGTE_49	20	58476704	58490389	20q13.33	SYCP2	4	gain
3941	CGTE_49	20	58490468	58558139	20q13.33	SYCP2,PPP1R3D,FAM217B,CDH26	3	gain
3942	CGTE_49	20	60881681	60909856	20q13.33	MIR4758,ADRM1,LAMA5	1	loss
3943	CGTE_49	21	34399131	34443066	21q22.11	OLIG2,OLIG1,LINC00945	1	loss
3944	CGTE_49	21	38463681	38480696	21q22.13	TTC3	0	loss
3945	CGTE_49	22	17586335	17603033	22q11.1	IL17RA,LOC100996342,CECR6	1	loss
3946	CGTE_49	22	21983160	21984303	22q11.21	YDJC	0	loss
3947	CGTE_49	22	28192793	28196535	22q12.1	MN1	1	loss
3948	CGTE_49	X	40990457	41007958	Xp11.4	USP9X	3	gain
3949	CGTE_49	X	152686435	152710636	Xq28	ZFP92,TREX2	0	loss
3950	CGTE_50	1	1560821	1565994	1p36.33	MIB2	1	loss
3951	CGTE_50	1	3410264	3422105	1p36.32	MEGF6	1	loss
3952	CGTE_50	1	17263187	17282087	1p36.13	CROCC	1	loss
3953	CGTE_50	1	68944964	70257849	1p31.2-p31.1	DEPDC1-AS1,LRRC7,DEPDC1	3	gain
3954	CGTE_50	1	74574927	74621554	1p31.1	LRRIQ3	17	gain
3955	CGTE_50	1	76313843	76342759	1p31.1	MSH4	5	gain
3956	CGTE_50	1	115400018	115428932	1p13.2	SYCP1	3	gain
3957	CGTE_50	1	150681375	150684368	1q21.3	HORMAD1	8	gain
3958	CGTE_50	1	215741025	215747523	1q41	KCTD3	6	gain
3959	CGTE_50	2	71607298	71632910	2p13.2	ZNF638	3	gain
3960	CGTE_50	2	183586733	183604413	2q32.1	DNAJC10	3	gain
3961	CGTE_50	2	191774955	191785970	2q32.2	GLS	3	gain
3962	CGTE_50	2	197750085	197762011	2q33.1	PGAP1	3	gain
3963	CGTE_50	2	219724753	219868021	2q35	FEV,LOC100129175,WNT10A,LINC01494,WNT6,CFAP65,LINC00608,CRYBA2,MIR375,CDK5R2	1	loss
3964	CGTE_50	3	156638320	156645441	3q25.31	LEKR1	12	gain
3965	CGTE_50	4	57352532	57354249	4q12	SRP72	6	gain
3966	CGTE_50	4	146042619	146046333	4q31.21	ABCE1	9	gain

3967	CGTE_50	5	1878107	3601317	5p15.33	IRX2,CTD-2194D22.4,C5orf38,LINC01017,IRX4,IRX1,LINC01019,LOC100506858,LINC01377	1	loss
3968	CGTE_50	6	43227286	43253116	6p21.1	TTBK1	1	loss
3969	CGTE_50	6	123595745	123600282	6q22.31	TRDN	3	gain
3970	CGTE_50	8	73933006	73942577	8q21.11	TERF1	0	loss
3971	CGTE_50	13	38934079	39262135	13q13.3	UFM1,FREM2,LINC00437,LINC00366	1	loss
3972	CGTE_50	13	60737611	61018931	13q21.2	LINC00434,TDRD3,DIAPH3	1	loss
3973	CGTE_50	14	39587046	39597495	14q21.1	GEMIN2	3	gain
3974	CGTE_50	14	60756531	60933752	14q23.1	PPM1A,C14orf39	3	gain
3975	CGTE_50	14	60935160	60938256	14q23.1	C14orf39	0	loss
3976	CGTE_50	14	60938258	60951791	14q23.1	C14orf39	4	gain
3977	CGTE_50	14	103388895	103396383	14q32.32	AMN	0	loss
3978	CGTE_50	14	104508507	105174379	14q32.33	MIR4710,TDRD9,MIR203A,KIF26A,TMEM179,MIR203B,INF2,ASPG,C14orf180	1	loss
3979	CGTE_50	15	63998909	64005925	15q22.31	HERC1	4	gain
3980	CGTE_50	17	29508408	29557491	17q11.2	NF1	3	gain
3981	CGTE_50	17	79092145	79167999	17q25.3	MIR657,MIR3065,AATK,MIR1250,MIR338,CEP131,AATK-AS1	1	loss
3982	CGTE_50	18	9124945	9134187	18p11.22	NDUFV2,NDUFV2-AS1	0	loss
3983	CGTE_50	19	1257157	1277291	19p13.3	C19orf24,CIRBP,CIRBP-AS1,MIDN	3	gain
3984	CGTE_50	19	18898978	18902255	19p13.11	COMP	0	loss
3985	CGTE_50	20	3765656	3777513	20p13	CDC25B,CENPB	1	loss
3986	CGTE_50	20	21492570	23064646	20p11.22-p11.21	SSTR4,LOC284788,PAX1,THBD,FOXA2,LOC101929608,LINC00261,LINC01432,LOC101929625,LINC01427,LINC01384,NKX2-2,CD93	1	loss
3987	CGTE_50	20	58460902	58496498	20q13.33	SYCP2	3	gain
3988	CGTE_50	21	34224032	34442669	21q22.11	LINC00945,OLIG1,OLIG2	1	loss
3989	CGTE_50	21	38463681	38480696	21q22.13	TTC3	0	loss
3990	CGTE_50	22	21975728	21984303	22q11.21	YDJC,UBE2L3	0	loss

**Supplementary Table S3.** Frequency of genes with driver mutations across different cohorts. The table is sorted according to frequency found in the INCan cohort. The frequency is presented in percentage.			
**Gene**	**INCan**	**TCGA**	**OncoSG**
TP53	41.5	39.7	46.5
CDH1	22.0	6.9	7.5
ARID1A	19.5	21.3	13.9
PIK3CA	12.2	13.6	6.4
LRP1B	9.8	21.8	19.8
ARID1B	7.3	6.3	3.2
BCOR	7.3	5.9	3.2
BRCA2	7.3	7.7	3.7
CDH10	7.3	6.5	4.3
CDKN2A	7.3	4.2	3.2
NOTCH1	7.3	5.9	3.7
RHOA	7.3	4.0	3.2
SVEP1	7.3	6.5	5.9
ARHGAP29	4.9	2.7	1.1
ARID2	4.9	6.1	4.8
ASPM	4.9	7.7	5.9
ATRX	4.9	5.4	3.2
AXIN1	4.9	2.9	3.7
CTC1	4.9	1.7	1.1
ELF3	4.9	2.9	2.1
ERBB2	4.9	4.0	8.0
ERBB3	4.9	7.3	5.3
FAT1	4.9	7.1	5.9
FAT2	4.9	9.8	6.4
FAT3	4.9	15.3	14.4
FAT4	4.9	17.8	11.2
FBXW7	4.9	6.5	5.3
GRM3	4.9	4.2	1.6
INPP4A	4.9	1.5	0.5
ITGAV	4.9	4.0	3.7
MECOM	4.9	2.5	0.5
NBEA	4.9	11.3	11.8
PIK3R1	4.9	2.3	3.2
PLXNB2	4.9	4.0	1.6
POT1	4.9	1.5	1.1
PTEN	4.9	6.3	1.6
PTGS1	4.9	2.3	0.5
PTPRU	4.9	4.8	2.1
RNF43	4.9	0.0	8.6
SETD2	4.9	3.6	2.7
SMAD4	4.9	5.9	5.3
SMO	4.9	2.9	1.6
STK11	4.9	1.3	1.1
ZNF292	4.9	6.9	2.1
AC006064.6	2.4	0.0	0.0
ACACA	2.4	5.4	4.3
ACVR2A	2.4	12.3	8.0
ADCY1	2.4	3.3	3.7
AHCTF1	2.4	3.1	2.7
AKT1	2.4	0.6	0.5
APC	2.4	10.3	8.0
ARFGEF1	2.4	6.1	4.3
ARHGAP35	2.4	3.8	2.7
ARID4A	2.4	4.6	2.7
ASH1L	2.4	7.5	5.9
ASXL1	2.4	4.8	5.3
ATP1A1	2.4	2.7	2.7
AXIN2	2.4	4.4	1.6
BAP1	2.4	2.3	1.6

BCLAF1	2.4	6.7	0.0
BIRC6	2.4	6.9	7.0
BIVM-ERCC5	2.4	0.0	0.0
BMP5	2.4	2.3	1.6
BPTF	2.4	6.3	3.7
BRAF	2.4	3.8	0.5
BRWD1	2.4	5.0	3.7
BUB1B	2.4	2.5	1.1
CASP8	2.4	3.8	2.1
CBLB	2.4	0.4	0.0
CCR7	2.4	0.8	1.1
CD28	2.4	0.2	0.0
CDK4	2.4	0.4	0.0
CEP290	2.4	5.6	7.0
CHD4	2.4	7.7	4.3
CHD8	2.4	4.8	3.2
CHEK2	2.4	2.1	0.5
CIC	2.4	10.7	6.4
CLOCK	2.4	2.5	4.8
CLTC	2.4	2.3	1.6
CNOT4	2.4	1.5	2.1
CR2	2.4	5.0	4.3
CREBBP	2.4	7.9	5.3
CSMD3	2.4	0.0	15.0
CSNK2A1	2.4	1.0	0.0
CTCF	2.4	4.4	2.1
CTNNA2	2.4	5.0	7.5
CTNNB1	2.4	5.4	7.5
CTNND1	2.4	5.0	2.1
CTNND2	2.4	7.3	3.7
CTTN	2.4	2.7	2.1
CUX1	2.4	5.4	3.2
DCAF12L2	2.4	4.8	1.6
DCC	2.4	7.1	5.3
DDX5	2.4	1.3	2.7
DICER1	2.4	2.1	2.7
ELAC2	2.4	1.3	0.0
EPHA1	2.4	2.9	1.1
EPHA2	2.4	5.9	2.1
EPHA4	2.4	3.3	2.7
ERCC2	2.4	1.9	0.5
ERCC3	2.4	1.7	1.6
ERCC6	2.4	4.8	4.8
EXT2	2.4	1.3	1.1
EZH2	2.4	1.0	2.1
FMR1	2.4	1.3	1.6
FN1	2.4	5.6	4.8
FOXA2	2.4	0.8	0.5
GATA1	2.4	0.6	0.0
GATA2	2.4	1.0	1.6
GATA3	2.4	3.8	2.1
GLI1	2.4	4.2	4.8
GNA13	2.4	1.3	0.5
GPS2	2.4	0.6	0.5
H3-3A	2.4	0.6	0.5
HERC2	2.4	9.6	5.9
HLA-A	2.4	4.4	0.0
IKBKB	2.4	2.1	1.6
ING1	2.4	1.3	0.5
INPPL1	2.4	5.0	2.1
ITGA9	2.4	1.9	2.7
ITSN1	2.4	3.1	1.6
KDR	2.4	3.3	3.7
KMT2C	2.4	11.5	10.2
KMT2D	2.4	14.4	7.0

KRAS	2.4	7.7	6.4
LEF1	2.4	1.0	1.1
LRPPRC	2.4	1.9	1.1
MAP3K1	2.4	3.1	1.6
MAP3K13	2.4	2.5	0.5
MED12	2.4	3.1	2.1
MED23	2.4	4.0	2.7
MGA	2.4	5.9	4.8
MGMT	2.4	1.0	0.5
MNDA	2.4	3.1	3.7
MRE11	2.4	1.5	0.0
MSH3	2.4	7.1	3.2
MSH6	2.4	4.4	1.6
MTOR	2.4	5.4	2.7
MUC16	2.4	28.0	18.7
MYH11	2.4	5.2	4.8
MYH14	2.4	4.8	2.7
MYH9	2.4	7.3	5.3
NCOR1	2.4	5.6	2.7
NCOR2	2.4	6.5	5.3
NF1	2.4	6.3	6.4
NOTCH2	2.4	6.9	3.7
NTRK1	2.4	1.9	1.1
NTRK3	2.4	2.3	1.6
OCA2	2.4	6.3	5.3
PABPC1	2.4	1.7	0.5
PDGFRA	2.4	2.5	2.1
PER1	2.4	0.8	1.6
PGR	2.4	3.8	3.2
PHOX2B	2.4	1.0	1.6
PIK3CB	2.4	2.5	0.5
PIK3CD	2.4	3.1	1.6
PLCG1	2.4	3.8	2.1
PMS1	2.4	1.7	1.6
PMS2	2.4	1.3	1.6
POLD1	2.4	2.9	3.2
POLQ	2.4	6.7	5.3
POLR2B	2.4	2.3	0.5
PRDM2	2.4	6.1	5.3
PREX2	2.4	10.5	8.0
PRF1	2.4	2.7	1.1
PRKCZ	2.4	0.6	0.0
PTPN13	2.4	6.1	4.3
PTPRC	2.4	4.0	2.7
PTPRD	2.4	6.5	5.9
PTPRF	2.4	3.6	6.4
RAD23B	2.4	0.8	1.1
RGS3	2.4	3.1	2.1
ROS1	2.4	5.0	4.3
SDHA	2.4	2.7	1.6
SDHAF2	2.4	0.0	0.0
SETBP1	2.4	7.1	5.9
SFRP4	2.4	1.0	3.7
SH2B3	2.4	1.0	1.1
SMAD2	2.4	1.9	0.5
SMARCA4	2.4	5.2	8.0
SPEN	2.4	7.7	2.7
SPTAN1	2.4	4.4	4.8
STAT3	2.4	1.3	0.0
STAT5B	2.4	3.1	0.0
STK4	2.4	0.6	1.1
TAOK1	2.4	2.1	3.7
TBX3	2.4	1.7	0.5
TET2	2.4	2.5	4.3
TGFBR2	2.4	3.3	8.0

TJP1	2.4	4.6	2.7
TNC	2.4	5.6	2.1
TNPO1	2.4	1.5	2.1
TNPO2	2.4	2.5	0.5
TRIO	2.4	8.8	4.3
TRRAP	2.4	10.0	8.0
UBR5	2.4	10.5	8.6
VIM	2.4	2.1	0.5
WASF3	2.4	4.0	3.2
WNK2	2.4	0.0	4.3
WRN	2.4	4.2	2.1
ZEB1	2.4	3.3	1.6
ZFHX3	2.4	8.8	5.9
ZMYM3	2.4	3.1	1.1
